# Rh^I^/Rh^III^ catalyst-controlled divergent aryl/heteroaryl C–H bond functionalization of picolinamides with alkynes†
†Electronic supplementary information (ESI) available: Experimental and computational details as well as spectroscopic and analytical data for new compounds. CCDC 1027406–1027411. For ESI and crystallographic data in CIF or other electronic format see DOI: 10.1039/c5sc01885d


**DOI:** 10.1039/c5sc01885d

**Published:** 2015-06-29

**Authors:** Ángel Manu Martínez, Javier Echavarren, Inés Alonso, Nuria Rodríguez, Ramón Gómez Arrayás, Juan C. Carretero

**Affiliations:** a Universidad Autónoma de Madrid (UAM) , Cantoblanco 28049 , Madrid , Spain . Email: n.rodriguez@uam.es ; Email: ramon.gomez@uam.es ; Email: juancarlos.carretero@uam.es

## Abstract

The ability to establish switchable site-selectivity through catalyst control in the direct functionalization of molecules that contain distinct C–H bonds remains a demanding challenge that would enable the construction of diverse scaffolds from the same starting materials. Herein we describe the realization of this goal, namely a divergent heteroaryl/aryl C–H functionalization of aromatic picolinamide derivatives, targeting two distinct C–H sites, either at the pyridine ring or at the arene unit, to afford isoquinoline or *ortho*-olefinated benzylamine (or phenethylamine) derivatives. This complementary reactivity has been achieved on the basis of a Rh^III^/Rh^I^ switch in the catalyst, resulting in different mechanistic outcomes. Notably, a series of experimental and DFT mechanistic studies revealed important insights about the mechanism of the reaction and reasons behind the divergent regiochemical outcome.

## Abstract 2

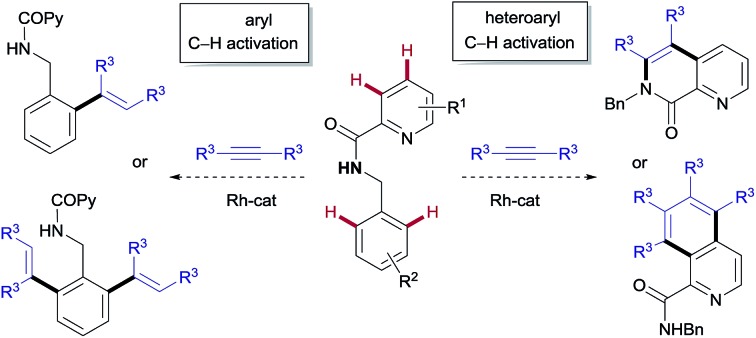
Switchable site-selectivity through catalyst control is achieved in the direct functionalization of picolinamides that contain two distinct C–H sites to construct diverse scaffolds from the same starting material.

## Introduction

The great potential of metal-catalyzed C–H bond functionalization to streamline synthetic schemes has been illustrated with many elegant methods featuring exquisite and predictable site-selectivity in the presence of multiple reactive C–H bonds.^1^ However, despite the fast-paced development of this field, the discovery of procedures capable of divergent functionalization at distinct C–H sites through catalyst control is relatively uncommon,^2^ yet highly appealing. In particular, achieving distinctive positional reactivities by simply varying the ligand environment and oxidation state of the catalytically active metal species could provide a unique opportunity for the construction of diverse scaffolds from the same starting materials.

Rh-catalyzed coupling reactions of alkynes involving C–H cyclometalation/annulation of (hetero)arenes provide an atom- and step-economical route to heterocycles, ubiquitous structural elements in nature, medicinal chemistry and material science.^3^–^7^ Also, the use of alkynes as coupling partners allows access to aromatic compounds with a pendant *ortho*-vinyl group,^6^ that could serve as a versatile synthetic handle. In both contexts, rhodium(iii)-catalysts, most often introduced as Cp*Rh^III^L_*n*_ precursors in combination with the classical Cu^I^/Cu^II^ redox couple, have proven to be particularly useful.^3^–^5^ However, in contrast to the tremendous strides made with functionalized arenes,^3^–^6^ there are few methods for the Rh^III^-catalyzed C–H activation of electron-deficient aza-heterocycles containing a basic nitrogen such as pyridine.^7^ This deficiency is somewhat surprising given that nitrogen-containing heterocyclic compounds are privileged structures in medicinal chemistry.

**Fig. 1:**
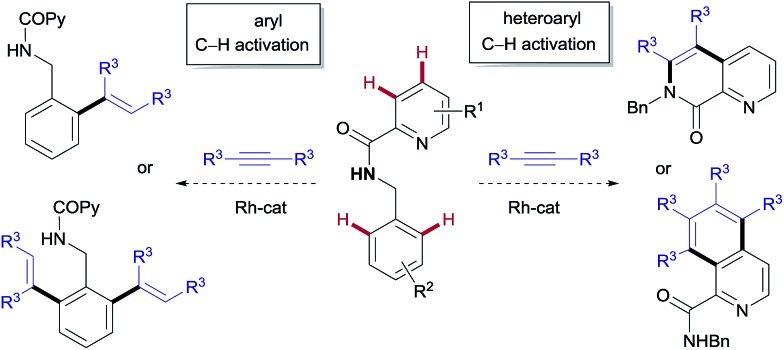
Proposed catalyst-controlled divergent aryl/heteroaryl C–H functionalization.

We envisaged that *N*-benzyl-2-picolinamides would provide an opportunity for developing a divergent C–H functionalization procedure targeting selectively either the pyridyl unit or the benzyl moiety. Our plan is outlined in Fig. 1. There are several challenges behind the choice of this substrate. Firstly, the aminocarbonyl group at C2 might strengthen the interaction between the pyridinic nitrogen and the metal through a bidentate coordination, thereby preventing the catalyst from interacting with the target pyridinic C–H bond.^8^ In fact, the picolinamide (COPy) has been extensively used as a directing group in a variety of C(sp^2^)– and C(sp^3^)–H functionalization reactions.^9^ In contrast, there have only been isolated examples of successful derivatization at the pyridine ring,^10^ thus highlighting the challenging nature of this task. Recently, the groups of Shi^11^ and our own^8^ managed to overcome this difficulty and reported the Rh^III^-catalyzed *ortho*-olefination/annulation of picolinamides with electron-deficient olefins. Secondly, the benzylamine unit embedded in the substrate is prone to dehydrogenation at the benzylic position under the oxidative Rh/Cu^II^ system, potentially leading to imine-type intermediates.12a,b The scarcity of precedents for the functionalization of benzylamine derivatives^12^ compared to the variety of methods available for benzoic acid derivatives^3^ points toward a challenging transformation.

Herein we describe the catalyst-controlled divergent heteroaryl/aryl functionalization of picolinamide derivatives that provides selective straightforward access to either isoquinoline-1-carboxamide or *ortho*-olefinated benzylamine (or phenethylamine) derivatives. This complementary reactivity has been achieved by simply choosing between either a Rh^III^ or a Rh^I^ catalyst.^2^ To our knowledge, Rh^I^/Rh^III^ divergent control in C–H activation on the same substrate remains undocumented.

## Results and discussion

### Optimization studies

The model reaction between *N*-benzylpicolinamide (**1**) and diphenylacetylene was chosen for the optimization studies (Table 1). A low but promising outcome was obtained with [RhCp*Cl_2_]_2_ (2.5 mol%) in conjunction with Cu(OAc)_2_ (2 equiv.), providing a 1 : 3.7 mixture of the isoquinoline-1-carboxamide derivative **2** (ref. 13) and the di-olefinated benzylamine derivative **3**,^13^ both resulting from two C–H activations and two alkyne insertions at either the pyridyl or the benzene unit (entry 1). The replacement of Cu(OAc)_2_ with Cu(TFA)_2_ led to suppression of the catalytic activity, likely due to the lower basicity of trifluoroacetate compared to acetate (entry 2). In fact, the addition of 4 equiv. of NaOAc to the Rh/Cu(TFA)_2_ system restored the catalytic activity (entry 3), suggesting that the oxidant is a source of acetate, necessary for the reaction to proceed. Further investigation (see the ESI†), led us to find that the addition of AgSbF_6_ (10 mol%) to sequester the chloride ligands remarkably improved both the reactivity and the site selectivity, allowing a clean and complete conversion of **1** into isoquinoline **2** as the single coupling product (entry 4). Control experiments determined that the product formation is completely inhibited in the absence of the Rh catalyst (entry 5) or with the omission of the copper salt, even when using O_2_ as an external co-oxidant (entry 6). Interestingly, however, the reactivity was partially restored but lead selectively to the di-olefinated product **3**, albeit with moderate yield, without the Cu^II^ salt but in the presence of NaOAc (entry 7). These optimization studies are evidence for the critical role played by both the Cu(OAc)_2_, as both the oxidant and carboxylate source, and AgSbF_6_, responsible for promoting the ligand exchange at Rh, in determining the catalyst activity and selectivity towards the formation of isoquinoline **2**.

**Table 1:** Optimization studies in the reaction of **1** with diphenylacetylene^*a*^

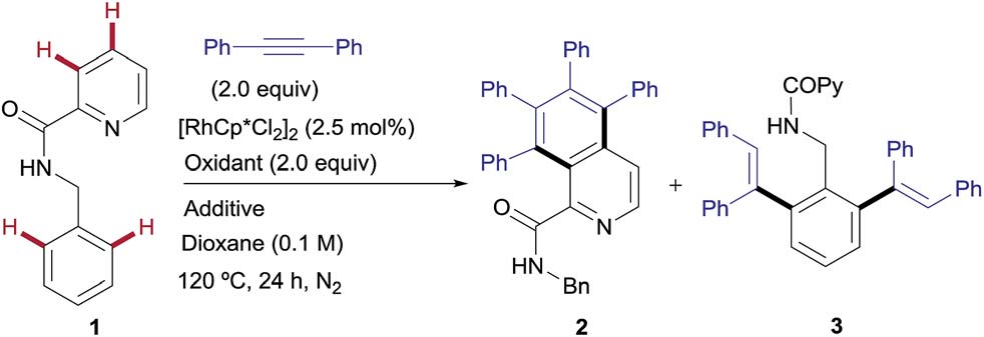				
Entry	Oxidant	Additive	Conversion^*b*^ (%)	**2**/**3**^*c*^ (%)
1	Cu(OAc)_2_	—	47	21/79
2	Cu(TFA)_2_	—	—	—/—
3	Cu(TFA)_2_	NaOAc^*d*^	66	44/56
4	Cu(OAc)_2_	AgSbF_6_^*e*^	>98	>98/<2
5^*f*^	Cu(OAc)_2_	AgSbF_6_^*e*^	—	—/—
6^*g*^	—	AgSbF_6_^*e*^	—	—/—
7	—	NaOAc^*d*^/AgSbF_6_^*e*^	46	<2/>98

^*a*^ **1** (0.15 mmol), alkyne (0.30 mmol), [Rh^III^]-cat. (5 mol%), oxidant (2.0 equiv.), dioxane (0.1 M), 120 °C, 24 h.

^*b*^Determined by ^1^H NMR from the crude mixture.

^*c*^[Rh^III^]-cat. (10 mol%).

^*d*^4.0 equiv.

^*e*^10 mol%.

^*f*^Without Rh-catalyst.

^*g*^Under O_2_.

### Rh^III^-catalyzed pyridyl C–H functionalization: synthesis of isoquinoline derivatives

**Scheme 1:**
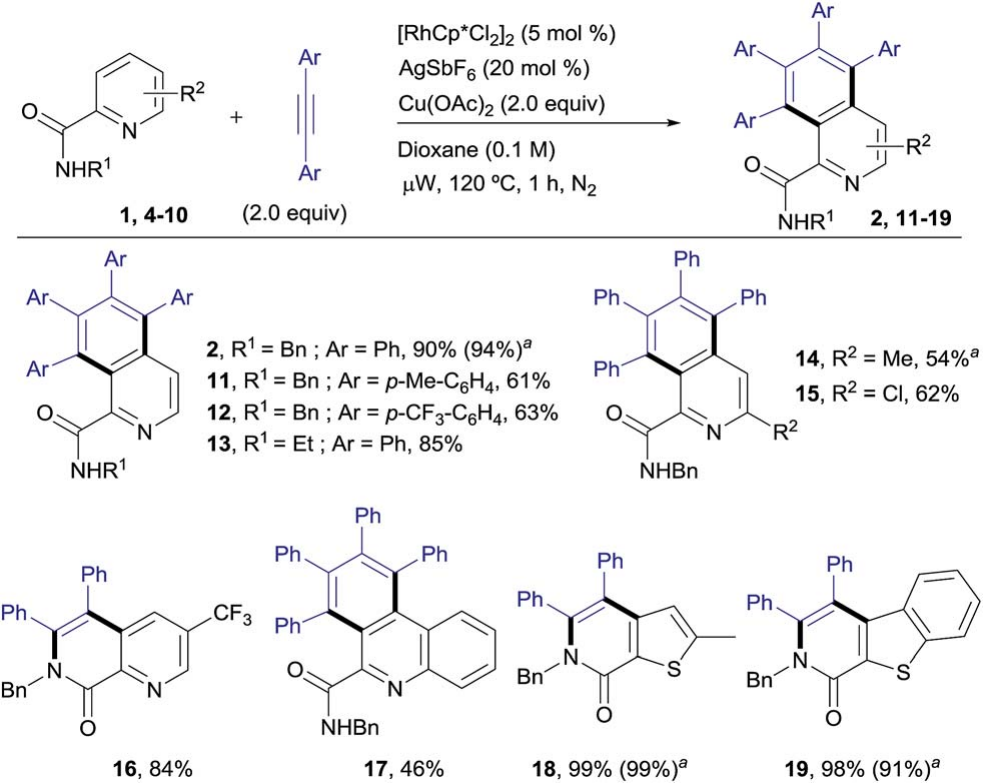
Rh^III^-catalyzed pyridyl C–H functionalization, leading to the isoquinoline derivatives. Conditions: benzylamine derivative (0.15 mmol), alkyne (0.30 mmol), [RhCp*Cl_2_]_2_ (5 mol%), Cu(OAc)_2_ (2.0 equiv.), dioxane (0.1 M), μW, 120 °C, 1 h. ^a^Under conventional heating at 120 °C for 24 h.

The scope of this aromatic homologation method allows for the construction of variously substituted polyarylated isoquinoline derivatives (Scheme 1). It is important to note that the isoquinoline moiety forms the core of many biologically active molecules.^14^ In this study microwave heating was generally applied since it dramatically reduced reaction times (from 24 h to just 1 h) while preserving the high site-selectivity, as exemplified in the isolation of **2** in 90% yield. Both electron-donating and electron-withdrawing substituents at either the pyridine (**14–15**, 54 and 62%) or the diarylacetylene^15^ (**11–12**, 61% and 63%) coupling partners were well tolerated. It is also remarkable that the Cl substituent survived the reaction conditions (**15**, 62%). The use of substituents on the amide nitrogen other than a benzyl group was also tolerated, as demonstrated by the good reactivity displayed by the substrate bearing an ethyl substituent (R = Et), which provided the corresponding isoquinoline derivative **13** in 85% yield.

Interestingly, the presence of a CF_3_ group at the C5 of the pyridine ring, residing in close proximity to one of the reactive C–H bonds, interrupted the aromatic homologation and led exclusively to the 1,7-naphthyridin-8(7*H*)-one derivative **16** (ref. 13) (84% yield), resulting from a double C–H/N–H activation2*c* and only one alkyne insertion. Although factors affecting these reactivity differences remain to be elucidated, this result suggests that the second alkyne insertion/C–H activation is sensitive to steric effects, so that the presence of a substituent in the aryl *ortho*-position to the reactive C–H site may impart a significant steric demand, thereby bypassing the normal reaction outcome and favouring the competitive trapping of the plausible alkenyl rhodium intermediate by the amidic N–H. Extension of this reaction to heteroaryl 2-carboxamides with quinoline, thiophene or benzo[*b*]thiophene skeletons proved successful, but whereas the first substrate evolved to give the expected aromatic homologation product in moderate yield (**17**, 46%), in the other two cases the reaction proceeded through the “interrupted” pathway, leading to the C–H/N–H cyclization products **18** (ref. 13) and **19** in excellent yields (98–99%).

### Rh^I^-catalyzed C–H *ortho*-olefination at the benzylamine unit

**Scheme 2:**
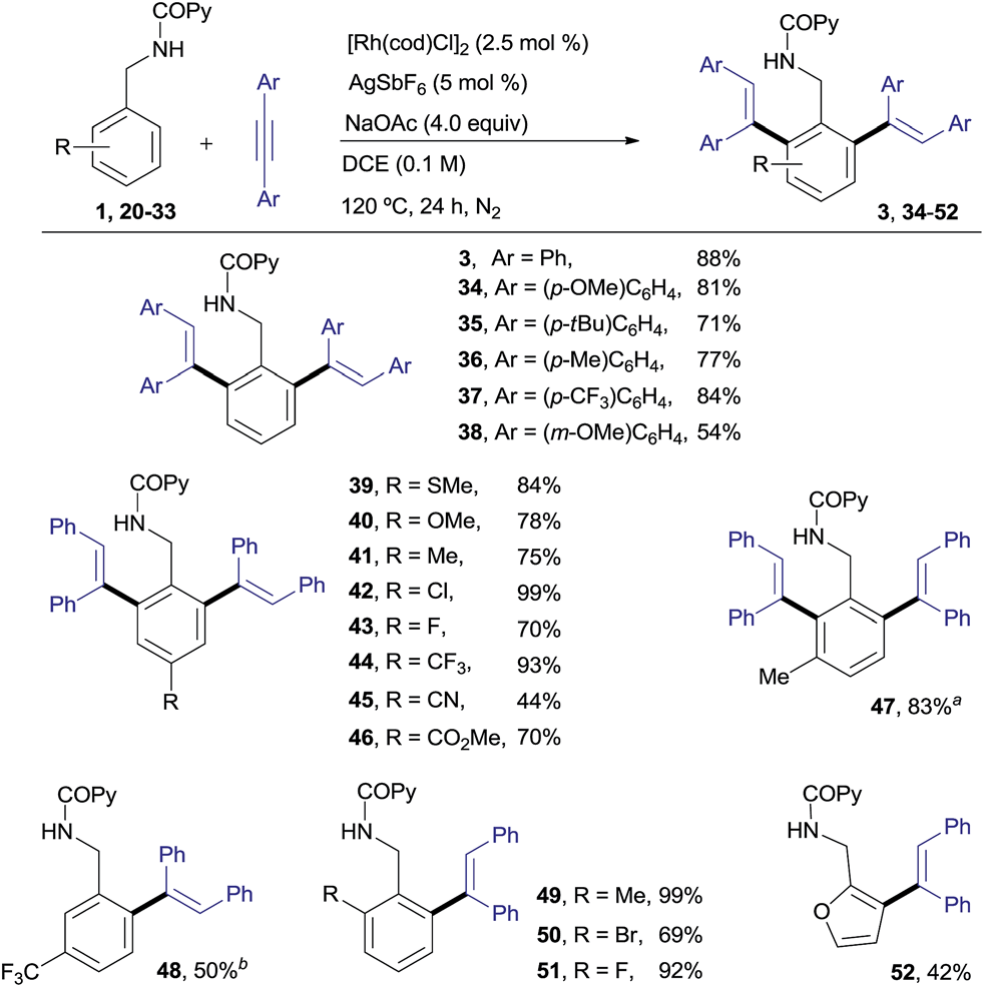
Rh^I^-catalyzed *ortho*-olefination of benzylamine derivatives. ^a^The mono-*ortho*-olefinated product was also isolated in 8% yield. ^b^The di-olefinated product was also isolated in 6% yield.

Our desired goal of developing a divergent C–H functionalization protocol guided us to revisit the low-yielding but encouragingly selective formation of the di-olefinated benzylamine **3**, observed in the reaction of **1** with diphenylacetylene in the absence of Cu(OAc)_2_ but using NaOAc as an acetate ion source (see Table 1, entry 7, 46% NMR conversion). We reasoned that in the absence of the Cu^II^-terminal oxidant, Rh^I^ species,^16^ rather than Rh^III^, could be a competent catalyst leading to the di-*ortho*-olefination product through a distinct mechanistic pathway. To our delight, this was indeed the case and the desired product **3** was obtained in 88% yield when using [Rh(cod)Cl]_2_ (ref. 17) (2.5 mol%) under conditions very similar to those in entry 7 of Table 1 (Scheme 2).

As shown in Scheme 2, *N*-benzylpicolinamide **1** smoothly reacted with a variety of diarylacetylenes^15^ equipped with both electron-rich and electron-poor *para*-substituted aryl groups, to give the corresponding di-olefinated benzylamine derivatives in good yield (**34–37**, 71–84%). *meta*-Substitution at the diaryl acetylene is also possible, albeit with lower efficiency (**38**, 54%), while no reaction was observed with the more sterically hindered *ortho*-substituted diaryl acetylenes (not shown).^18^ A broad range of *para*-, *ortho*- and *meta*-substituents at the benzylamine unit with very different electronic properties proved to be suitable substrates (**39–51**, 44–99% yield). The functional-group compatibility is remarkable, including coordinating functionalities (CN or SMe), and halogens (Cl and, especially, the challenging Br). A *meta*-Me substituent led to the di-olefinated product in good yield (**47**, 83% yield), while a *meta*-CF_3_ resulted mainly in mono-olefination at the sterically less hindered *ortho*-position (**48**, 50% yield). *ortho*-Substitution, which often results in reduced reactivity for steric reasons, was well tolerated (**49–51**, 69–99%). Likewise, the successful use of a heteroaromatic substrate turned out to be viable, albeit in a lower yield (furanyl derivative **52**, 42%).

### Exploration of unsymmetrical alkyl-substituted internal alkynes

**Scheme 3:**
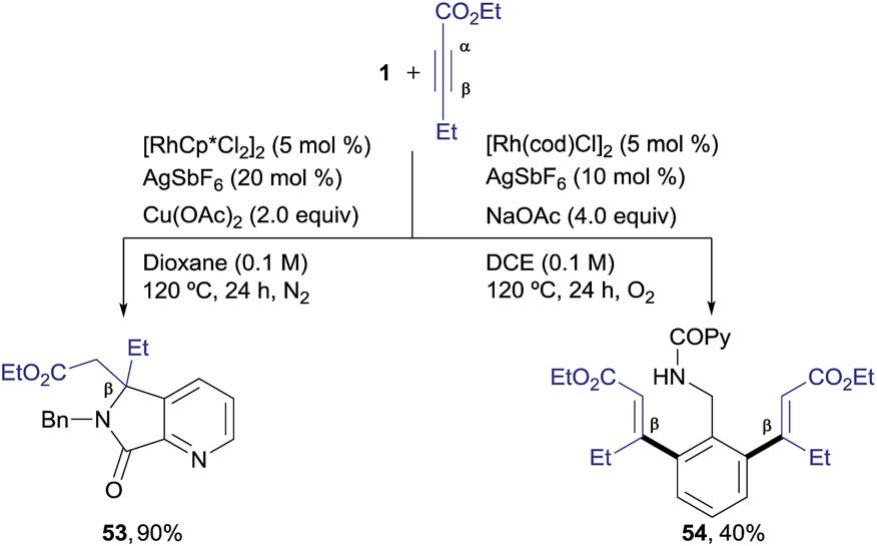
Rhodium-controlled divergent aryl/heteroaryl C–H functionalization in the reaction of **1** with ethyl pent-2-ynoate.

We next explored unsymmetrical alkynes, for which regiocontrol in the insertion step becomes an issue of concern. Unsymmetrical aliphatic-substituted internal alkynes are a more challenging type of substrate due to their diminished reactivity and poor regioselectivity in the 1,2-migratory insertion often observed in the context of rhodium-catalyzed C–H functionalization.4*f* Interestingly, it was found that β-alkyl acetylenic esters did participate in the pyridyl/phenyl divergent C–H functionalization with excellent selectivity, albeit with a different reaction outcome than the diarylalkynes (Scheme 3). For instance, the reaction of **1** with ethyl pent-2-ynoate under the Rh^III^-catalyzed conditions (*i.e.*, the isoquinoline formation conditions) led to an *ortho*-functionalization at the pyridine ring but it did not yield the corresponding isoquinoline. Instead, the 5,5-fused bicyclic ester **53**, with a valuable 6,7-dihydro-5*H*-pyrrolo[3,4-*b*]pyridine architecture holding a quaternary carbon center, was obtained as the sole reaction product in good yield (90%). This compound seems to arise from a competitive evolution of the alkyne insertion complex that prevents the second alkyne insertion/C–H activation. On the other hand, this result demonstrates that the reaction outcome can be significantly influenced by changes in the alkyne substitution. However, when the same two reacting partners (**1** + ethyl 2-pentynoate) were submitted to the Rh^I^-catalyzed conditions, a clean formation of the di-olefinated benzylamine derivative **54** was observed, yet in modest yield (40%). In the latter case, the reaction was found to be accelerated under aerobic conditions (air or a balloon of O_2_). Remarkably, both of the Rh^III^ and Rh^I^ C–H functionalization processes led to products with complete regioselectivity regarding the alkyne insertion (in both cases at the β-position of the ethyl 2-pentynoate).

**Scheme 4:**
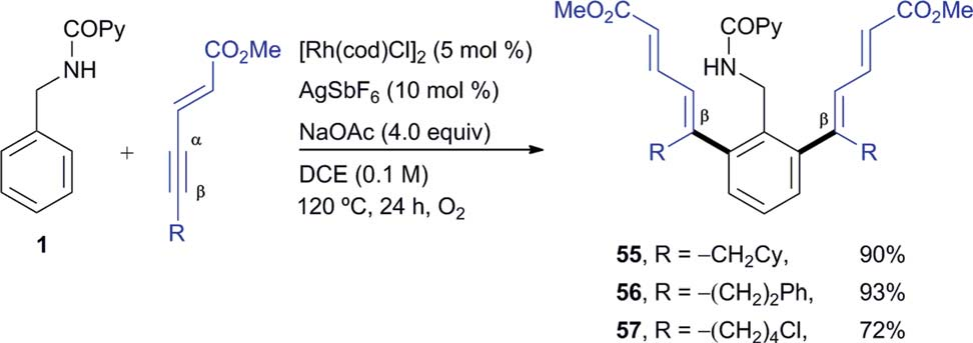
Rh^I^-catalyzed *ortho*-olefination of **1** with 1,3-enynes.

The use of enynes as another type of non-aromatic alkyne coupling partner with an electronic bias for highly regioselective insertion, elegantly introduced by Huestis and co-workers in the context of C–H functionalization,4*q* led us to easily prepare di-*ortho*-dienyl benzylamine derivatives in good yields (products **55–57**, 72–93% yield, Scheme 4). This reaction revealed the tolerance of this catalyst system towards a sensitive alkyl chloride substituent (**57**, 72% yield). As occurred in the case of the acetylenic esters, higher reaction rates were observed under aerobic conditions and in all cases studied the conjugated moiety attached to the alkyne ended up at the vinylic position away from the phenyl ring with complete regiocontrol.

**Scheme 5:**
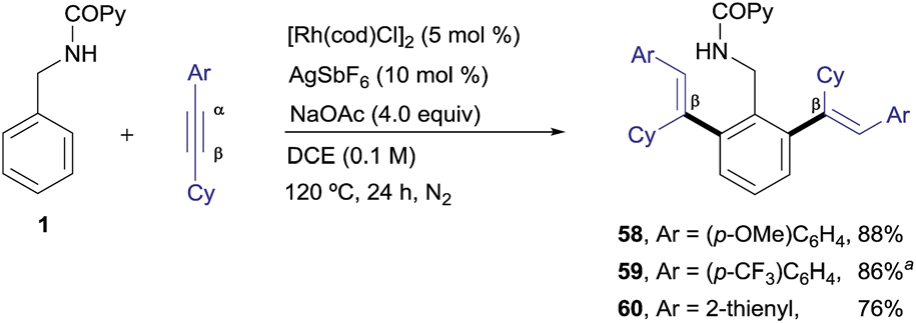
Rh^I^-catalyzed *ortho*-olefination of **1** with aryl-cyclohexyl-acetylenes. ^a^Using 5 mol% [Rh(cod)Cl]_2_ and 10 mol% of AgSbF_6_.

Finally, as shown in Scheme 5, some unsymmetrical alkyl–aryl–alkynes, such as cyclohexyl–aryl–acetylenes, also participated in the Rh^I^-catalyzed cross-coupling reaction, affording the desired di-olefinated products as single regioisomers and stereoisomers (products **58–60**, 76–88%) showing that, as in the previous examples, there is complete regiocontrol in favour of functionalization at the β-position of the starting conjugated alkyne. In contrast, very poor conversion was observed with oct-1-yn-1-ylbenzene while internal dialkyl-alkynes such as 2-butyne resulted in a total lack of reactivity (not shown).

### Extension of the reactions to phenethylamine derivatives

**Scheme 6:**
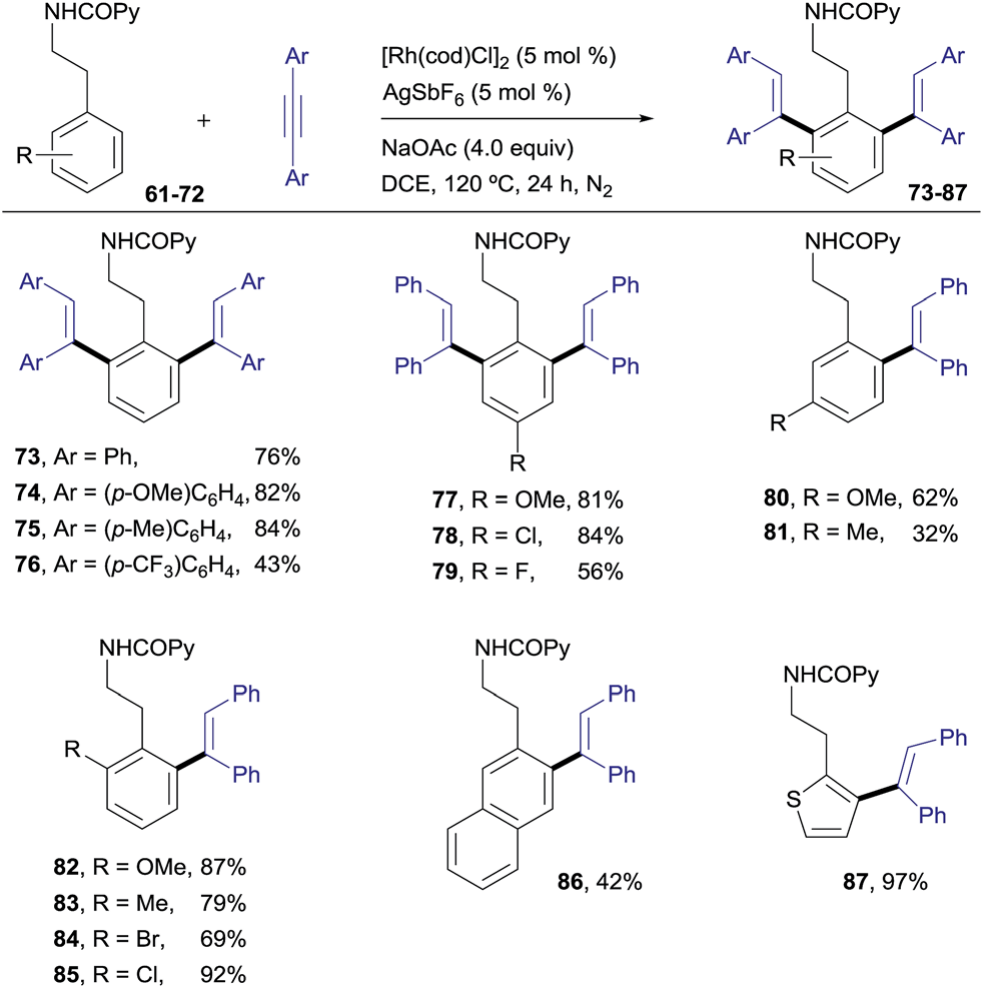
Rh^I^-catalyzed *ortho*-olefination of phenethylamine derivatives.

Pleasingly, this method could be extended to phenethylamine derivatives, which have a tether that is one carbon longer with regard to the directing group. *N*(COPy)-phenethylamine (**61**)^19^ reacted smoothly with diphenylacetylene under the optimized conditions to give the di-olefinated product **73** (ref. 13) in 76% yield (Scheme 6). In terms of scope, the results parallel those found with the benzylamine derivatives, with the applicability to naphthalene (**86**, 42%) and heteroaromatic (**87**, 97%) compounds being of particular relevance. This structural flexibility is noteworthy, since very often the precise tether length of the directing group is found to be crucial for reactivity in C–H functionalizations.^20^

**Scheme 7:**
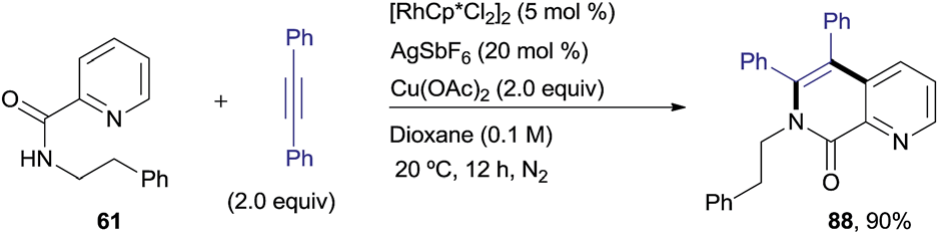
Rh^III^-catalyzed pyridyl C–H functionalization leading to a 1,7-naphthyridin-8(7*H*)-one derivative.

The complementary reactivity of the Rh^III^-catalyzed oxidative alkenylation/annulation was also briefly explored with *N*-(2-picolinamide)-protected phenethylamine substrates (Scheme 7). As in the model reaction, when the parent substrate **61** was submitted to the standard optimized reaction conditions, the 1,7-naphthyridin-8(7*H*)-one **88** was produced as a single product in 90% yield. This product results from a double C–H/N–H activation and only one alkyne insertion (referred to as the “interrupted” pathway) rather than the aromatic homologation *via* the two-fold C–H activation previously observed for the reaction of the analogous benzylamine derivative under identical reaction conditions (product **2**, 94% yield). This result adds additional weight to the noticed sensitivity of this catalyst system to steric hindrance, which appears to strongly influence the reaction outcome.

### Chemoselective deprotection and removal of the auxiliary COPy group

**Scheme 8:**
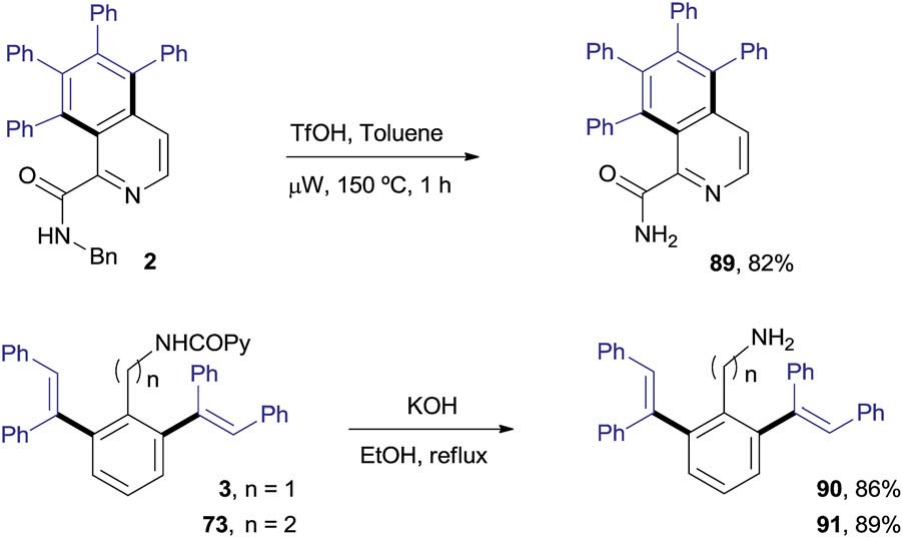
Deprotection of the *N*-benzyl group and removal of the COPy directing group.

Scheme 8 illustrates the chemoselective *N*-deprotection of **2** to give the isoquinoline-2-carboxamide derivative **89** (82%), as well as the facile removal of the auxiliary picolinamide directing group in both the benzyl- and phenethylamine di-olefinated products (**90** and **91**, 86% and 89%, respectively).

### Mechanistic insights

#### Stoichiometric reactions of the isolated Rh-complexes

**Fig. 2:**
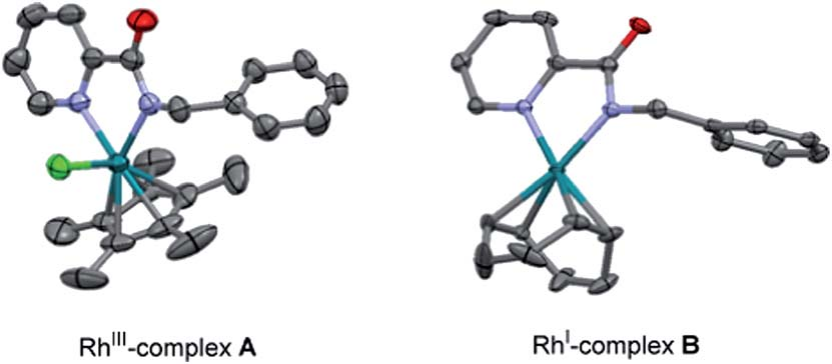
ORTEP view of Rh^III^-complex **A** and Rh^I^-complex **B**. The hydrogen atoms have been removed for simplicity.

**Scheme 9:**
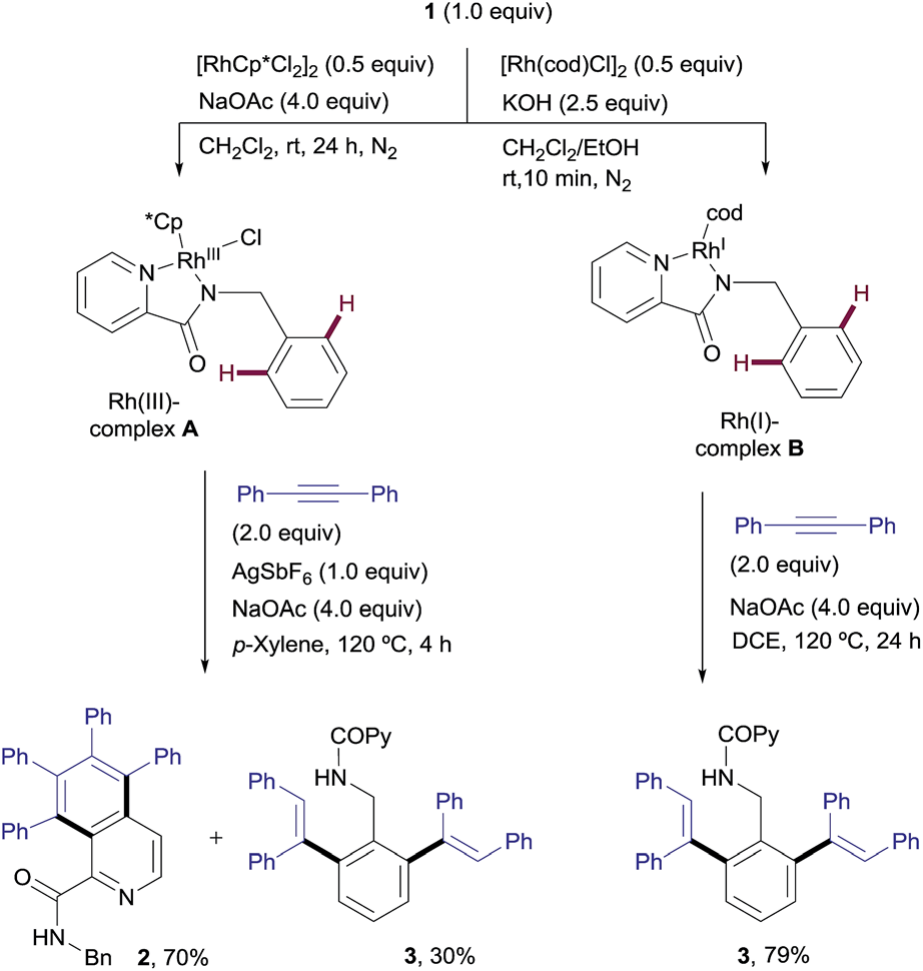
Stoichiometric studies with isolated Rh^III^- and Rh^I^-picolinamide complexes.

To shed light on the basis of this divergent functionalization, we tried to identify a Rh-complex that could be involved in each catalytic cycle. The stoichiometric reaction of the *N*-benzylpicolinamide (**1**) with [RhCp*Cl_2_]_2_, in the presence of NaOAc in CH_2_Cl_2_ at room temperature led to Rh^III^-complex **A**, showing *N*,*N*-coordination of the picolinamide to Rh (see the X-ray structure in Fig. 2).^21^ However, this bidentate coordination does not prevent the metal center from interacting with the target pyridinic C–H bond. In fact, **A** reacted with diphenylacetylene to afford in quantitative yield a 70 : 30 mixture of the isoquinoline derivative **2** and the di-olefinated product **3**, in the presence of NaOAc at 120 °C in only 4 h (Scheme 9). It is worth remarking that no reactivity is observed in the absence of NaOAc.

On the other hand, the stoichiometric reaction of **1** with [Rh(cod)Cl]_2_, under similar conditions to those employed in the formation of complex **A**, provided the Rh^I^-complex **B**, whose X-ray structure showed a similar *N*,*N*-bidentate metal coordination (Fig. 2, see the ESI† for details).^13^ Remarkably, the reaction of complex **B** with diphenylacetylene afforded the di-olefinated product **3** as the only product (Scheme 9). Control experiments confirmed again that NaOAc is crucial for the reaction to proceed.

#### Deuterium labeling studies

**Scheme 10:**
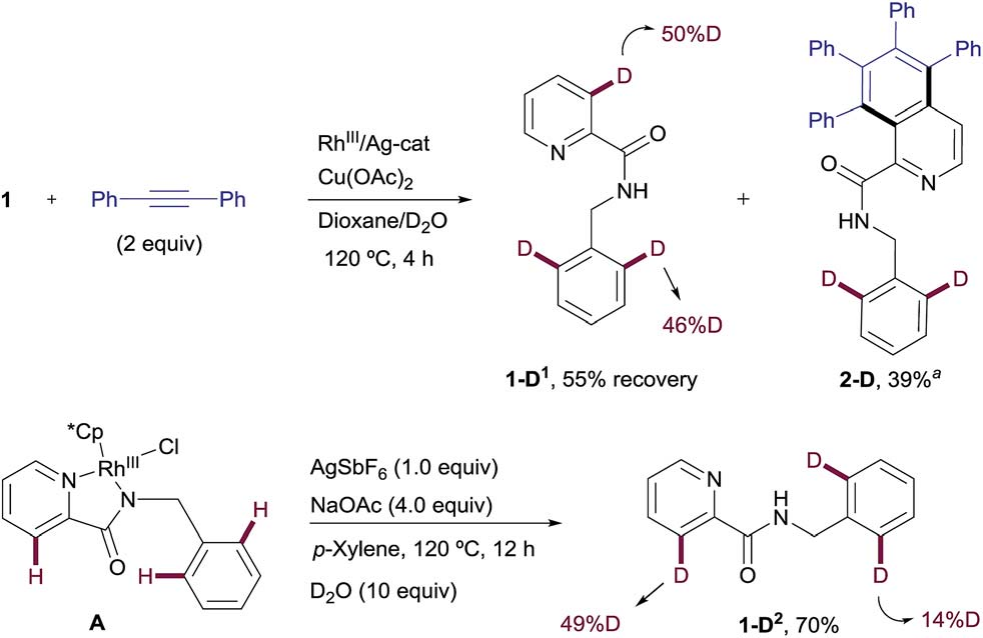
H/D exchange experiments in the Rh^III^-promoted C–H functionalization process. ^a^The H/D exchange was detected using mass spectrometry in this case (the exact deuterium content could not be determined by NMR).

To gain insight into both reaction mechanisms, a series of H/D exchange experiments were carried out next. The results obtained in the Rh^III^-catalyzed C–H functionalization of picolinamides are depicted in Scheme 10. The reaction of **1** with diphenylacetylene in the presence of [RhCp*Cl_2_]_2_ and Cu(OAc)_2_ in a dioxane/D_2_O mixture at 120 °C at incomplete conversion (4 h) gave isoquinoline derivative **2-D** in 39% yield with partial deuterium scrambling at the *ortho*-positions of the benzyl substituent. Meanwhile, the recovered starting material **1-D^1^** (55% yield) showed similar levels of deuterium incorporation at the C3–Py position (50%D) and the benzyl ring (46%D). These data suggest that a reversible metalation/deutero (proto)demetalation takes place prior to the coupling with the alkyne. The fact that the C–H activation is reversible at both the pyridine moiety and the phenyl moiety under catalytic conditions means that neither of them is rate-limiting. It also suggests that the selectivity is controlled not by the site of C–H cyclometalation but by the ease with which the two potential isomeric Rh-complexes undergo subsequent alkyne insertion.

Likewise, when Rh^III^-complex **A** was dissolved in a *p*-xylene/D_2_O mixture and heated at 120 °C in the presence of NaOAc and AgSbF_6_ for 12 h but in the absence of an alkyne, **1-D^2^** was recovered in 70% yield showing 49% of deuterium incorporation at the C3–Py and 14% of H/D scrambling at the *ortho*-positions of the benzyl moiety (Scheme 10). This result seems to indicate that with stoichiometric amounts of Rh, C–H insertion at both the aryl and heteroaryl sites also become reversible in the absence of the alkyne.

**Scheme 11:**
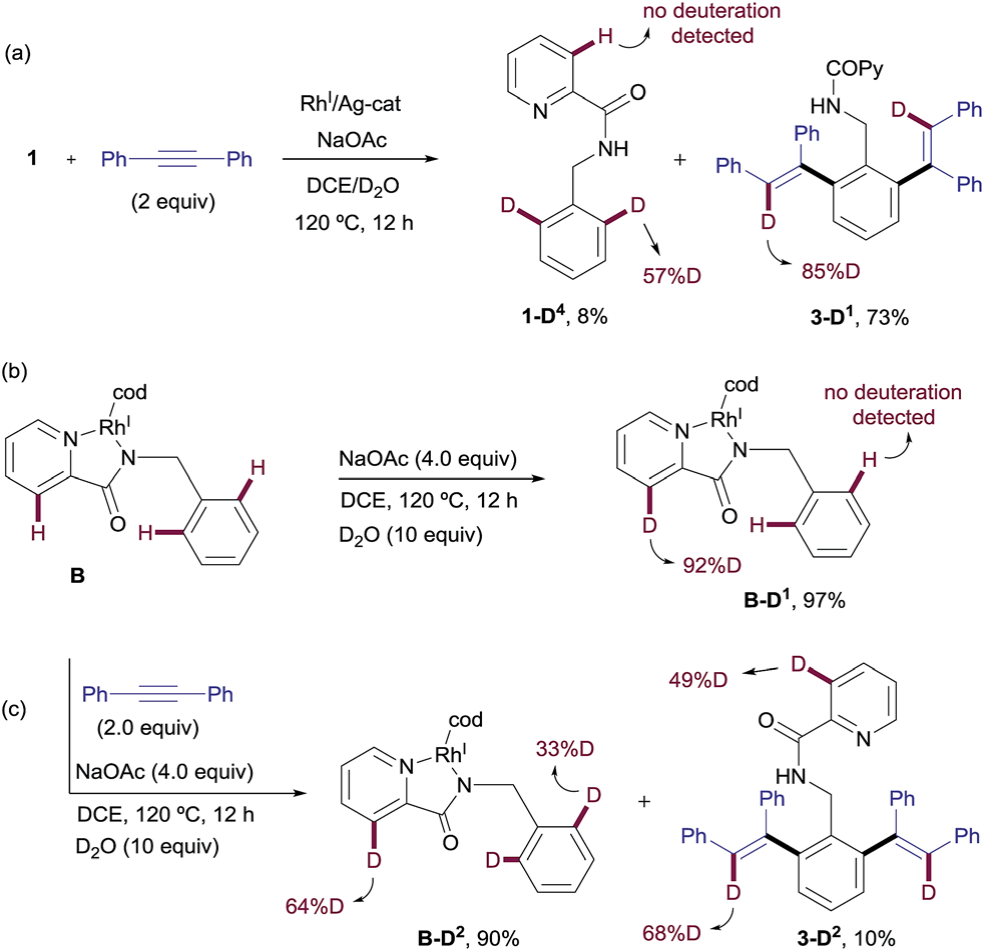
H/D exchange experiments in the Rh^I^-promoted C–H functionalization process.

Similar deuterium labeling studies were performed in the Rh^I^-promoted C–H functionalization process (Scheme 11). When substrate **1** was allowed to react with diphenylacetylene in a DCE/D_2_O mixture at 120 °C for 12 h under otherwise standard Rh^I^-catalyzed conditions {[Rh(cod)Cl]_2_ (2.5 mol%)/AgSbF_6_ (5 mol%) and NaOAc (4 equiv.)}, unreacted **1** was recovered (in 8% yield) with significant deuterium incorporation at the *ortho*-position of the benzylamine moiety (57%D) but no H/D exchange detected at the pyridine ring. The main component of the reaction mixture was the di-olefinated product **3-D^1^** (73% isolated yield), which showed high levels of deuterium incorporation at the vinylic position (85%D, Scheme 11a). This result suggests a reversible metalation/deutero(proto) demetalation at the reactive C–H sites, whereas activation at the pyridine ring appears to be less favorable. The high degree of deuteration at the vinylic positions of product **3-D^1^** is compatible with a mechanism of arene activation *via* oxidative insertion (which should retain the H/D incorporation from the starting material) in which the hydride/deuterium ligand exchange with D_2_O in the Rh^III^-complex, resulting from the oxidative addition of Rh^I^ into the *ortho*-C–H bond of **1**, readily occurs prior to reductive elimination.^22^

The evaluation of the potential of Rh^I^-complex **B** for metalation/deutero(proto) demetalation in the absence of an alkyne using a hydrogen/deuterium exchange process led to almost complete deuteration of the C3–Py position in Rh^I^-complex **B** (**B-D^1^**, 92%D), with no deuteration being observed at the benzylamine part (Scheme 11b). This result was in contrast to the high selectivity towards the benzylamine moiety observed under catalytic Rh^I^ in the presence of an alkyne, where no deuteration was observed at the pyridine ring. Product **B-D^1^** may arise from dissociation of the pyridinic nitrogen ligand from Rh (*e.g.*, through displacement by the acetate ion), followed by metalation/deutero-demetalation at the *ortho* 2-picolinamide moiety. Finally, when Rh^I^-complex **B** was mixed with the diphenylacetylene in a DCE/D_2_O mixture at 120 °C (Scheme 11c), a very low conversion to the dialkenylation product **3-D^2^** was observed (10% isolated yield after 12 h), which showed significant deuterium incorporation at both 3-pyridyl (49%D) and vinylic (68%D) positions. The unreacted complex was recovered in 90% isolated yield with 64% H/D scrambling at the C3–Py position and 33% deuterium incorporation in the benzylic moiety. This result suggests that, as previously observed in the Rh^III^-promoted outcome, the regioselectivity of the reaction is controlled not by the site of the C–H cyclometalation but by the rate at which the two potential isomeric Rh-complexes undergo subsequent alkyne insertion, which turns out to be opposite in the Rh^I^ or Rh^III^ pathways. The reasons behind the lower reactivity of complex **B** in the mixture DCE/D_2_O are not fully understood at the present time.

#### Plausible mechanistic hypothesis

**Scheme 12:**
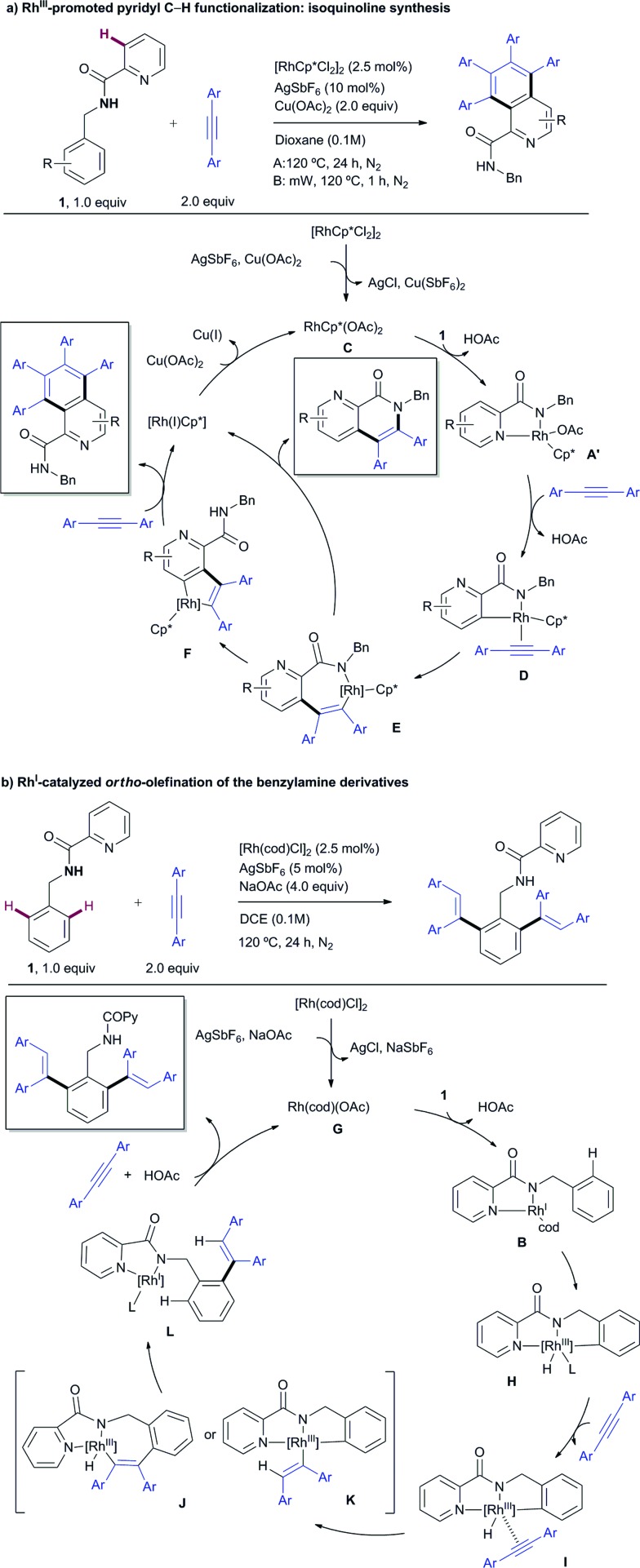
Simplified plausible mechanistic pathways.

Simplified general catalytic cycles for the aromatic homologation towards the isoquinoline formation and the di-*ortho*-olefination are shown in Scheme 12 based on the proposals described in the literature for related annulative processes with internal alkynes.^4^,^16^ The former reaction might proceed through a Rh^III^-catalyzed C–H activation of substrate **1***via* a concerted metalation-deprotonation (CMD) mechanism assisted by the acetate ion (Scheme 12a), while the *ortho*-olefination of the benzylamine derivatives might occur *via* an oxidative addition of Rh^I^ to the C–H bond (Scheme 12b).

The Rh^III^ catalytic pathway depicted in Scheme 12a is proposed to start by forming the highly soluble presumed active catalyst RhCp*(OAc)_2_ (**C**) *via* ligand exchange from [RhCp*Cl_2_]_2_ in the presence of an excess of acetate ions. Then, displacement of an acetate from **C** by the substrate (**1**) would lead to intermediate **A′**, analogous to the X-ray characterized complex **A**. A subsequent “rollover” cyclometalation^23^*via* pyridine decomplexation and rotation around the carbonyl–Py bond and then C–H bond activation, presumably by an acetate-assisted concerted metalation-deprotonation (CMD) pathway with concomitant loss of a second molecule of acetic acid, followed by an alkyne coordination affords **D**. 1,2-Migration of the rhodium–carbon bond across the alkyne results in the formation of the seven-membered rhodacycle **E**, which presumably triggers a second intramolecular C–H activation leading to a more stable five-membered Rh complex **F**. After the coordination and migratory insertion of a second alkyne molecule, a reductive elimination step releases the isoquinoline product while the concomitantly formed Rh^I^ species is oxidized by Cu^II^ acetate to regenerate the Rh^III^Cp*catalyst. Alternatively, if formation of complex **F** from **E** is hampered (for instance by steric crowding next to the reactive C–H site), the direct formation of the carbon–nitrogen bond from **E***via* reductive elimination becomes more favorable to afford the mono-insertion product (previously referred to as the “interrupted” pathway), at which time the metal catalyst is reduced to Rh^I^ and further oxidized to Rh^III^ by Cu^II^ acetate. In the case of using β-alkyl acetylenic esters (such as ethyl 2-pentynoate) as the coupling partner, the formation of the 6,7-dihydro-5*H*-pyrrolo[3,4-*b*]pyridine skeleton (product **53**) may arise from a fast proto-demetalation of the complex type **E** followed by either an intramolecular hydroamination and subsequent oxidation or an oxidative cyclization through electrophilic activation of the olefin, C–N bond formation and subsequent β-hydride elimination.

The first step in the catalytic cycle proposed for the Rh^I^-catalyzed *ortho*-olefination (Scheme 12b) would likely involve the formation of the catalytically active Rh–acetate complex **G***via* chloride displacement of an acetate ion from the Rh^I^-chloride precatalyst [Rh(cod)Cl]_2_. Coordination of substrate **1** in a bidentate fashion would lead to complex **B**,^13^ which has been isolated and structurally characterized by X-ray diffraction analysis. Complex **B** might undergo a reversible oxidative addition of an *ortho* aromatic C–H bond to the Rh^I^ to form hydrometallacycle **H**. Upon metal-coordination of the alkyne to afford complex **I**, a further *syn*-insertion to the rhodium–carbon or rhodium–hydride bond would afford **J** or **K**, respectively. Subsequent reductive elimination from **J** or **K** delivers the mono-alkenylation Rh^I^ complex **L**, primed for subsequent oxidative insertion at the other *ortho* C–H bond followed by alkyne insertion and reductive elimination to afford the di-alkenylated benzylamine product while regenerating the Rh^I^ catalyst.

#### Theoretical DFT calculations

**Fig. 3:**
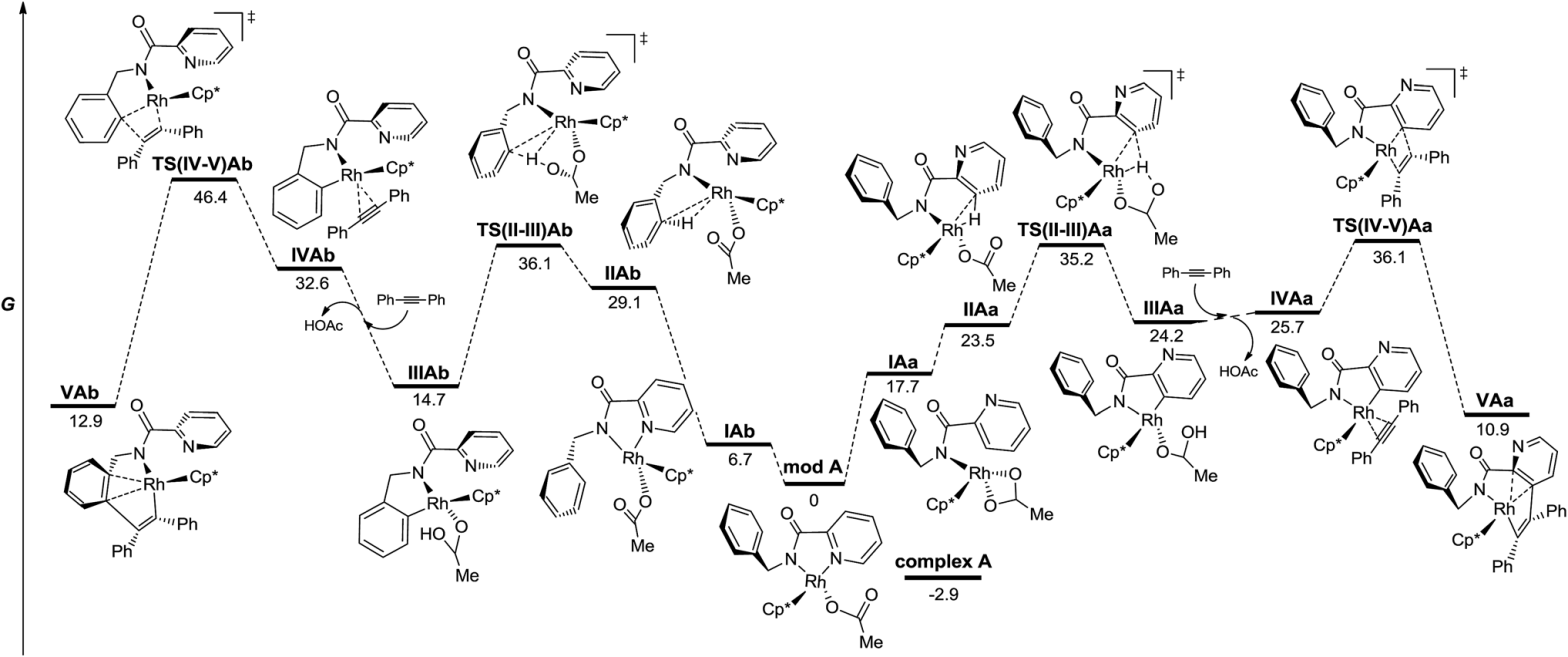
Energy profile for the C–H functionalization pathways of **1** with diphenylacetylene from neutral model Rh^III^ complexes in the gas phase (M06/6-311+G(d,p)(C,H,N,O),SDD (Rh)//6-31G(d)(C,H,N,O), LANL2DZ(Rh). The relative G values are in kcal mol^–1^ at 298 K).

**Fig. 4:**
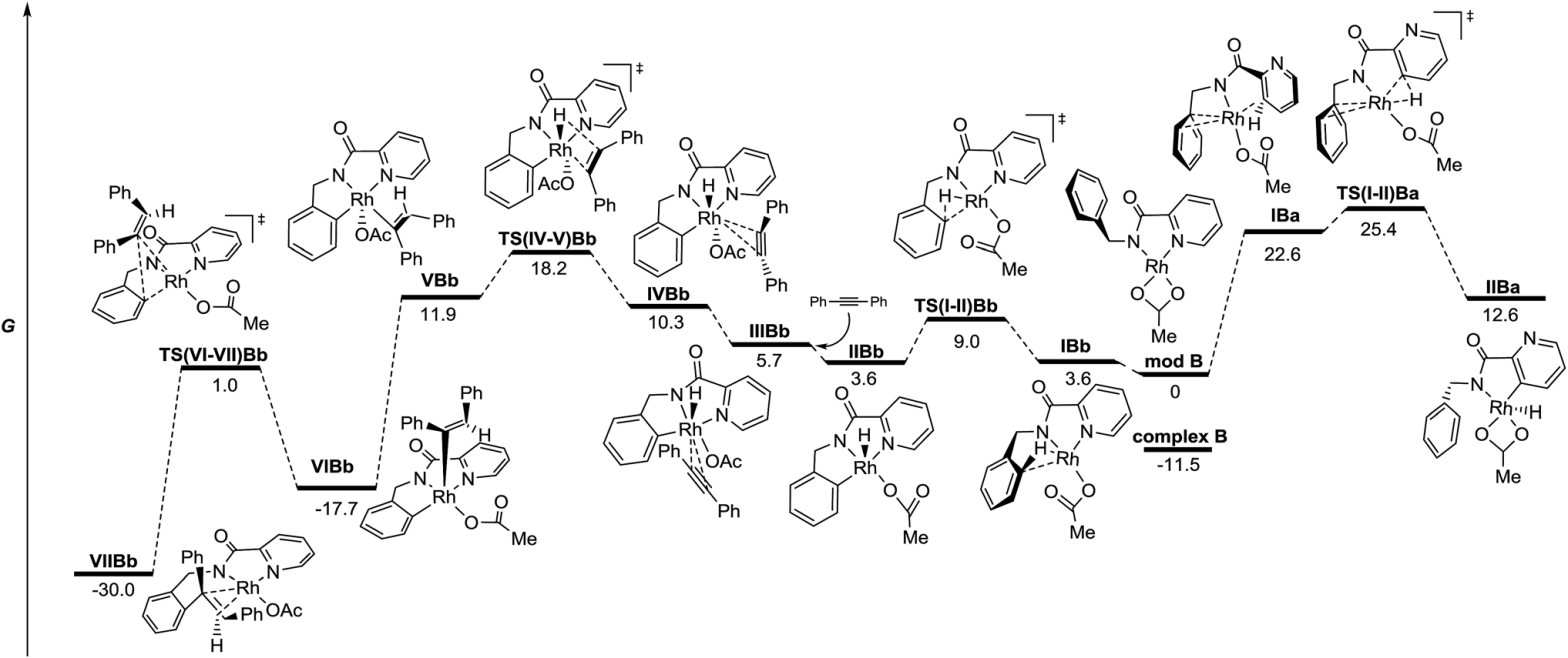
Energy profile for the C–H functionalization pathways of **1** with diphenylacetylene from anionic model Rh^I^ complexes in the gas phase (M06/6-311+G(d,p)(C,H,N,O),SDD (Rh)//6-31G(d)(C,H,N,O), LANL2DZ(Rh). The relative G values are in kcal mol^–1^ at 298 K). For simplicity, the negative charge has been omitted.

On the basis of the structures of the isolated Rh-complexes, Rh^III^-complex **A** and Rh^I^-complex **B**, and these two plausible proposed mechanisms, DFT calculations were performed to provide further insight to explain the observed catalyst-controlled divergent C–H bond activation of picolinamide derivatives (Fig. 3 and 4, see the ESI† for details). Taking into account that the acetate ion is always present and is crucial for the reactions to proceed for both catalysts, neutral model complex **modA** and anionic model **modB**, obtained from complexes **A** and **B** changing the “Cl” and “cod” ligands, respectively, for “OAc”, were selected as the catalytically active species.^24^ From these species the possible intermediates arising from the C–H activation of the benzyl and pyridyl rings (species “b” and “a”, respectively) and the diphenylacetylene insertion in each case have been studied.

Fig. 3 depicts the calculated lowest energy profile for the postulated CMD mechanism assisted by the acetate ion when using the Rh^III^ catalyst. All species show an almost tetrahedral coordination around the Rh atom similar to that found in the solid state for complex **A**. The C–H activation of either the pyridine ring or the arene unit (intermediates **IIA** and **TS(II-III)A**) requires a lack of the stabilizing interaction between the Rh atom and the pyridinic nitrogen and implies an important and quite similar activation barrier (36.1 and 35.2 kcal mol^–1^ for benzyl and pyridyl rings respectively). However, the key step that really determines the selective functionalization of the pyridine ring with the Rh^III^ catalyst is the insertion step for the diphenylacetylene unit. The activation barrier to reach that point after the benzyl C–H activation (**TS(IV-V)Ab**) is almost 10 kcal mol^–1^ higher than that after the pyridyl C–H activation (**TS(IV-V)Aa**). Once species **VAa** is formed, it will be involved in a second C–H activation–insertion sequence to afford the final product.

The energy profile for the reaction catalyzed by Rh^I^*via* oxidative addition across the C–H bond is depicted in Fig. 4. The different species show square planar coordination, similar to that observed in the solid state for complex **B** (**modB** and **VIIBb**), square pyramidal (**IIBb**, **VBb** and **VIBb**) or octahedral coordination (**IIIBb** and **IVBb**), depending on the number of ligands around the Rh atom in each case. The C–H activation step for the benzyl ring *via***TS(I-II)Bb**, which keeps the strong stabilizing interaction between the Rh atom and the pyridine nitrogen, is clearly favored over that of the pyridine ring (**TS(I-II)Ba**) with a lower activation barrier (9.0 compared to 25.4 kcal mol^–1^).^25^ However, analyzing the energy profile, the alkyne insertion step is again the determining step through **TS(IV-V)Bb** in which the new C–H bond is being formed.^26^ Structural reorganization gives species **VIBb** with a geometry suitable for the reductive elimination process (**TS(VI-VII)Bb**). Species **VIIBb** would continue the same reaction sequence: decomplexation and conformational changes to achieve cyclometalation, alkyne insertion and reductive elimination to afford the final product.

According to the energy profiles depicted in Fig. 3 and 4, the reaction catalyzed by Rh^III^ should follow selectively route “a” to afford products coming from pyridyl C–H activation, whereas the reaction catalyzed by Rh^I^ should follow route “b” to afford the *ortho*-olefination of the benzyl ring. Thus, these models would explain the experimental results found in both catalytic processes: the Rh^III^ catalyst affords products of type **2** whereas the Rh^I^ catalyst leads to products of type **3**. The decrease in selectivity found in the stoichiometric reaction of complex **A** (Scheme 9) may be a consequence of the easy reduction of Rh^III^ by the base^27^ in the absence of the usual Cu oxidant.

**Fig. 5:**
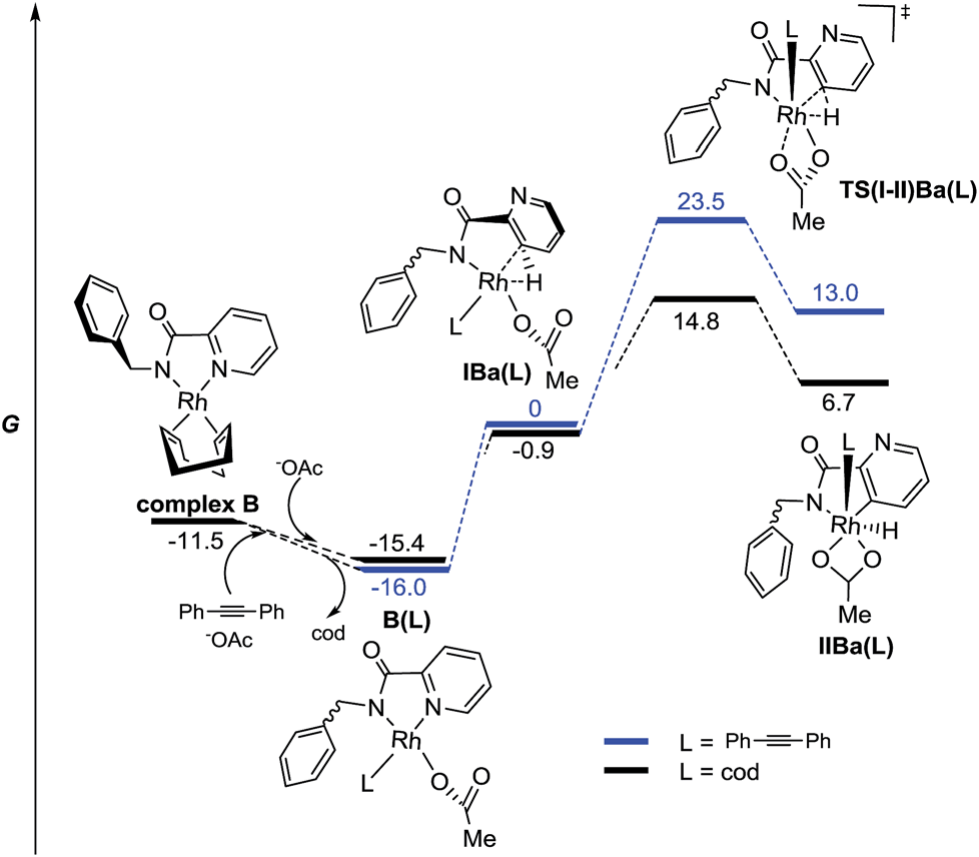
Energy profile for the C–H activation pathways of picolinamides from anionic model Rh^I^ complexes with monocoordinated “cod” or alkyne ligands in the gas phase (M06/6-311+G(d,p)(C,H,N,O),SDD (Rh)//6-31G(d)(C,H,N,O), LANL2DZ(Rh). The relative G values are in kcal mol^–1^ at 298 K).

The results found in the H/D exchange experiments can also be rationalized on the basis of the species depicted in Fig. 3 for the reaction catalyzed by Rh^III^. The C–H functionalization is favored at the C3–Py position and is a reversible process. However, when Rh^I^ is used as catalyst (Scheme 11), the results found in the stoichiometric reaction pointed out the possible role of other ligands such as “cod” and the alkyne partner to reach the catalytically active species or to affect the C–H activation process. To shed some light on this point, complexes including these ligands and the corresponding C–H activation processes were studied taking complex **B** as the starting model (Fig. 5).

From this species, the coordination of an acetate ligand could shift one of the olefin units of “cod” to afford a more stable complex **B(cod)**. Additionally, the resulting monocoordinated cod ligand could be effectively shifted by the alkyne partner to afford complex **B(diphenylacetylene)**, which is even more stable.^28^ This fact could explain the crucial role of the alkyne for the *ortho* C–H metalation reaction to take place because otherwise the “cod” ligand would stay bonded to the Rh atom.

The complexes **IBa(L)**, prior to the C–H activation step, resulted in quite similar energy barriers for both ligands. However, the C–H activation of the pyridyl ring through **TS(I-II)Ba(L)** resulted in being much more favored in the case of the “cod” ligand than in the case of the alkyne one (ΔΔ*G*^‡^ = 7.8 kcal mol^–1^). Thus, a reversible C–H activation of the pyridine ring could be expected in the absence of the alkyne, in agreement with experimental results (Scheme 11b), whereas if the alkyne is present the evolution through **modB** should be favored instead of the pyridyl C–H activation. All attempts to find any intermediate keeping either “cod” or the alkyne ligand bonded to the Rh atom that would be involved in the *ortho* C–H activation of the benzyl ring were unsuccessful, thus reinforcing the hypothesis of **modB** as the catalytically active species for the benzyl ring functionalization.

**Fig. 6:**
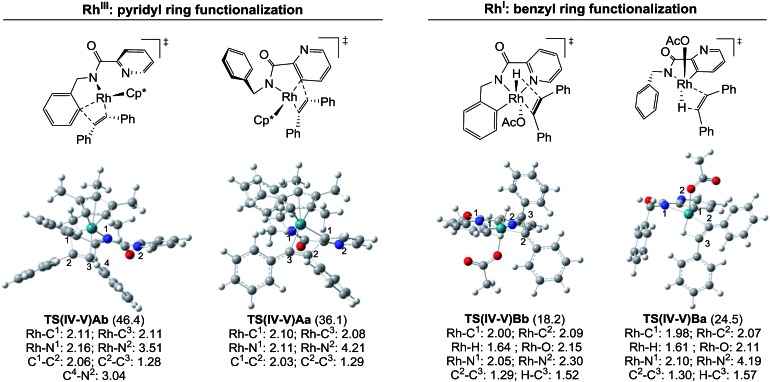
Molecular structures of the key transition states. The bond lengths are given in Å and the relative free energies with respect to **modA** and **modB** are indicated in parentheses (kcal mol^–1^).

The energy differences between each of the coupled key transition states, **TS(IV-V)Ab**/**TS(IV-V)Aa** and **TS(IV-V)Bb**/**TS(IV-V)Ba**, can be attributed to different steric and/or electronic interactions (Fig. 6). In the case of the reaction catalyzed by Rh^III^, transition states **TS(IV-V)Ab** and **TS(IV-V)Aa** show important steric differences. Whereas **TS(IV-V)Aa** is a late transition state that shows a shorter C_1_–C_2_ distance and longer C_2_–C_3_ with the phenyl groups spun around to avoid steric hindrance in the *Z*-alkene that is being formed, in **TS(IV-V)Ab** the pyridine ring does not allow the Ph group to reach an equivalent conformation, giving rise to an early transition state with a very distorted alkyne partner. In the case of the reaction catalyzed by Rh^I^, there are no relevant steric interactions. However, the ligands around the Rh atoms are quite different. Whereas **TS(IV-V)Bb** shows an octahedral coordination with one of the ligands being the pyridine nitrogen, **TS(IV-V)Ba** lacks this stabilizing interaction and only five ligands (instead of six) coordinate to the Rh atom.

#### Role of the base in the Rh^I^-catalyzed *ortho*-olefination of benzylamine derivatives

Based on our experimental studies, the acetate ion has a crucial role in the Rh^I^-catalyzed *ortho*-olefination of benzylamine derivatives (see the ESI† for further experimental details). This observation is supported by the above theoretical studies which suggest that the acetate ion leads to the active anionic species in the catalytic cycle. In order to gain better understanding of the role of the acetate ion, we embarked on synthesizing the new Rh^I^-complex **M**, related to complex **B** but with two monodentate ethylene molecules replacing the bidentate “cod” ligand. We envisaged that the greater lability of the bis(ethylene) complex should facilitate the formation of the postulated anionic Rh^I^-acetate complex.

**Scheme 13:**
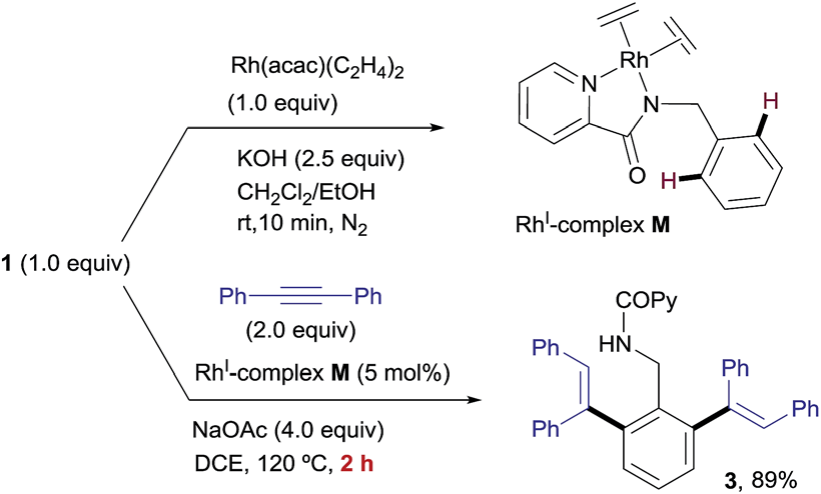
Synthesis and activity of Rh^I^-complex **M**.

**Fig. 7:**
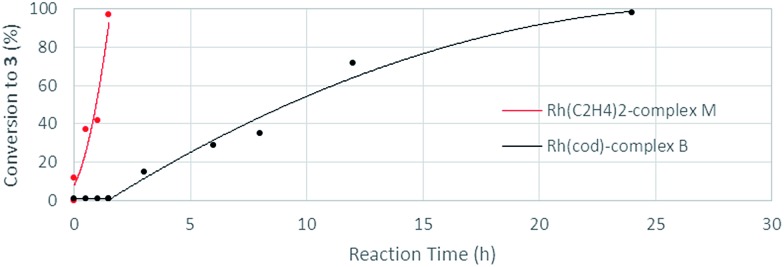
Kinetic profiles of complexes **B** and **M** as catalysts in parallel dialkenylation reactions of **1** with diphenylacetylene.

The stoichiometric reaction of *N*-benzylpicolinamide (**1**) with Rh(acac)(C_2_H_4_)_2_ in the presence of KOH in a CH_2_Cl_2_/EtOH mixture at room temperature allowed the isolation and full characterization of Rh^I^-complex **M** (Scheme 13). All attempts to crystalize Rh^I^(C_2_H_4_)_2_-complex **M** have failed so far due to its moderate stability. The activity of complex **M** was tested in the model reaction between *N*-benzylpicolinamide (**1**) and diphenylacetylene in otherwise standard reaction conditions. In line with our proposal, product **3** was isolated in 89% yield after only 2 h of reaction. In contrast, the reaction with catalytic amounts of Rh^I^-complex **B** was notably slower, observing a similar conversion only after 12 h (see the ESI† for further details). Indeed, as evidenced in the kinetic catalytic profiles of both complexes from parallel reactions shown in Fig. 7, complex **B** requires an activation period (more than 1 h) prior to becoming active, whereas catalyst **M** promoted almost complete conversion within 1.5 h without a noticeable induction period. This stark difference between the activity of complexes **B** and **M** was ascribed to the much easier displacement of the ethylene ligands from Rh^I^ by the acetate compared to the bidentate “cod” group, thereby accelerating the catalyst turnover, along with a loss of the “cod” ligand during the course of the reaction with the generation of vacant coordination sites.

## Conclusions

In conclusion, divergent highly site-selective control in the direct functionalization of both aryl and heteroaryl C–H bonds of *N*-substituted picolinamide substrates has been cleanly achieved by simply using either a Rh^I^ or Rh^III^ catalyst precursor, either using [RhCp*Cl_2_]_2_/AgSbF_6_/Cu(OAc)_2_ or [Rh(cod)Cl]_2_/AgSbF_6_/NaOAc. This method provides access to either isoquinoline derivatives or *ortho*-olefinated benzylamine and phenethylamine derivatives, respectively. Some experimental mechanistic studies based on the isolation of Rh^I^ and Rh^III^ picolinamide complexes, stoichiometric experiments and deuterium labeling studies, as well as DFT theoretical calculations, have been performed to explain this site-selective control for both the Rh^I^ and Rh^III^ catalytic systems and the intimate involvement of the acetate ion in the mechanism of these reactions.

## Supplementary Material

Supplementary information

Crystal structure data
