# Supplementary material for: RhI/RhIII catalyst-controlled divergent aryl/heteroaryl C–H bond functionalization of picolinamides with alkynes
Source: Chem Sci. 2015 Jun 29;6(10):5802–14. doi: 10.1039/c5sc01885d (PMC5950197; doi:10.1039/c5sc01885d)
Supplement: Supplementary file 1 [file SC-006-C5SC01885D-s001.pdf]

Electronic Supplementary Material (ESI) for Chemical Science.
This journal is © The Royal Society of Chemistry 2015

Supporting Information

Rh^{I}/Rh^{III} Catalyst-Controlled Divergent Aryl/Heteroaryl C–H Bond Functionalization of Picolinamides with Alkynes

Ángel Manu Martínez, Javier Echavarren, Inés Alonso, Nuria Rodríguez,* Ramón Gómez Arrayás* and Juan C. Carretero*

Departamento de Química Orgánica. Facultad de Ciencias. Universidad Autónoma de Madrid. Cantoblanco. 28049 Madrid. Spain

General Methods	S2
1. Additional information<br>1.1. Selected screening results<br>1.2. Other tested substrates and reaction conditions<br>1.3. X-ray structure determination	S3
2. Typical procedure for the protection of benzylamine derivatives	S7
3. Typical procedure for the protection of phenethylamine derivatives	S12
4. Typical procedure for the protection of alkylamine derivatives	S15
5. Typical procedure for the synthesis of alkynes	S15
6. Typical procedure for the synthesis of 1,3-enynes	S17
7. Rh(III)-catalyzed heteroaryl C–H functionalization	S19
8. Rh(I)-catalyzed *ortho*-olefination of benzylamine derivatives	S23
9. Rhodium-controlled divergent aryl/heteroaryl C–H functionalization using an alkynyl propiolate	S31
10. Rh(I)-catalyzed *ortho*-olefination of phenethylamine derivatives	S32
11. Typical procedure for the cleavage of the benzyl group	S37
12. Typical procedure for the cleavage of the picolinate group	S37
13. Synthesis of the Rh^{III}-complex **A**	S38
14. Synthesis of the Rh^{I}-complex **B**	S39
15. Synthesis of the Rh^{I}-complex **M**	S39
16. Mechanistic studies<br>16.1. Stoichiometric studies with the isolated Rh^{III} and Rh^{I} picolinamide complexes<br>16.2. H/D exchange experiments using D_2O as deuterium donor<br>16.3. Kinetic studies of the Rh^{I}-catalyzed *ortho*-olefination of *N*-benzylamine<br>16.4. Role of the base in the Rh^{I}-catalyzed *ortho*-olefination of *N*-benzylamine	S40
17. NMR Spectra	S61
18. Theoretical studies	S176

Experimental procedures and data

General Methods. The corresponding starting materials were synthetized using oven-dried glassware under a nitrogen atmosphere containing a teflon-coated stirrer bar and dry septum. All reactions were performed at ambient N_2 pressure in oven-dried 20 mL vessel containing a teflon-coated stirrer bar and dry septum. All microwave irradiation experiments were carried out in a mono mode microwave apparatus equipped with a pressure control system and a vertically-focused IR temperature sensor (CEM).

Solvents were purified by standard procedures prior to use. All other compounds are commercially available and were used without further purification.

Flash column chromatography was performed using 230-400 mesh ultra-pure silica gel. NMR spectra were obtained on 300 and 500 MHz spectrometers using acetone-d_6, chloroform-d and methanol-d_4 as solvents, with proton and carbon resonances at 300/500 MHz and 75/125 MHz, respectively. Mass spectral data were acquired on a VG *AutoSpec* mass spectrometer.

1. Additional information

1.1. Selected screening results (Table S1)

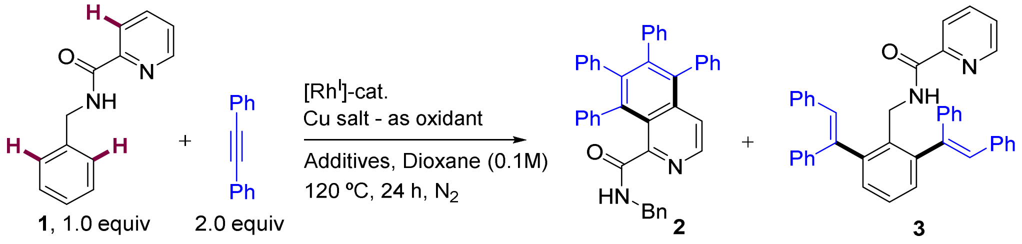

Entry	[Rh]-cat (mol%)		Cu-salt	Additives		Conv. (%)[a]	2/3 ratio (%)[a]
**1**	$[RhCl_2Cp^*]_2$	2.5	$Cu(OAc)_2$	–	–	47	21/79
**2[b]**	$[RhCl_2Cp^*]_2$	5.0	$Cu(OAc)_2$	–	–	63	32/68
**3**	$[RhCl_2Cp^*]_2$	5.0	$Cu(OAc)_2$	–	–	74	14/86
**4**	$[RhCl_2Cp^*]_2$	5.0	$Cu(OAc)_2$	–	$AgSbF_6$	≥ 99	≥ 98/≤ 2
**5**	$[RhCl_2Cp^*]_2$	2.5	$Cu(OAc)_2$	–	$AgSbF_6$	≥ 99	≥ 98/≤ 2
**6**	$[RhCl_2Cp^*]_2$	2.5	–	–	$AgSbF_6$	–	–
**7**	$[RhCl_2Cp^*]_2$	5.0	$Cu(TFA)_2$	–	–	0	–
**8**	$[RhCl_2Cp^*]_2$	5.0	$Cu(OAc)_2{\cdot}H_2O$	–	–	67	42/58
**9**	$[RhCl_2Cp^*]_2$	5.0	$Cu(SO_3CF_3)_2$	–	–	0	–
**10**	$[RhCl_2Cp^*]_2$	5.0	$Cu(TFA)_2$	NaOAc	–	66	44/56
**11**	$[RhCl_2Cp^*]_2$	5.0	–	NaOAc	–	68	≤ 2/≥ 98
**12**	$[RhCl_2Cp^*]_2$	2.5	–	NaOAc	$AgSbF_6$	46	≤ 2/≥ 98
**13**	$[Rh(cod)Cl]_2$	2.5	–	NaOAc	$AgSbF_6$	94	≤ 2/≥ 98
**14**	$Rh(acac)(C_2H_4)_2$	5.0	–	NaOAc	–	60	≤ 2/≥ 98
**15**	$Rh(cod)_2BF_4.H_2O$	5.0	–	NaOAc	–	83	≤ 2/≥ 98
**16**	$Rh(cod)_2PF_6$	5.0	–	NaOAc	–	77	≤ 2/≥ 98

Reaction conditions: **1** (0.15 mmol, 1.00 equiv), diphenylacetylene (0.30 mmol, 2.00 equiv), [Rh^{I}]-cat., additives NaOAc (4.00 equiv), $AgSbF_6$ (1:1 respect to the amount of Cl present in the Rh-cat.), Cu-salt (2.00 equiv), dioxane (0.1M), 120 °C, 24 h. [a] Determined by ^{1}H NMR from the crude mixture. [b] 4 h reaction time.

1.2. Other tested substrates and reaction conditions

- Attempts to control the Rh^{I}-catalyzed *ortho*-mono-olefination of the benzylamine derivatives provided a mixture of mono- and di-olefinated products.

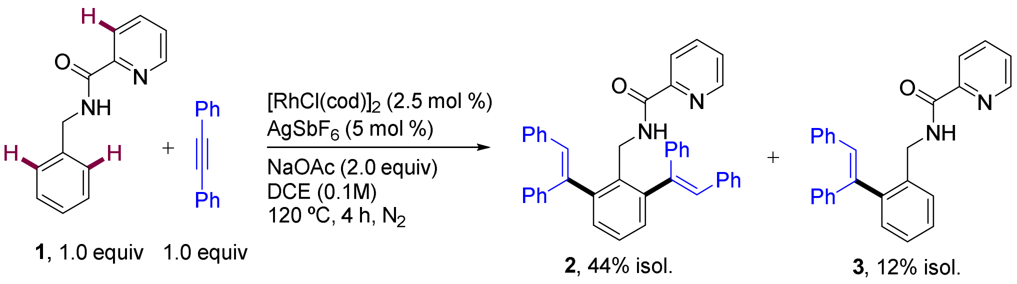

- *N*-alkylation is not tolerated: Tertiary picolinamide derivatives are unreactive.

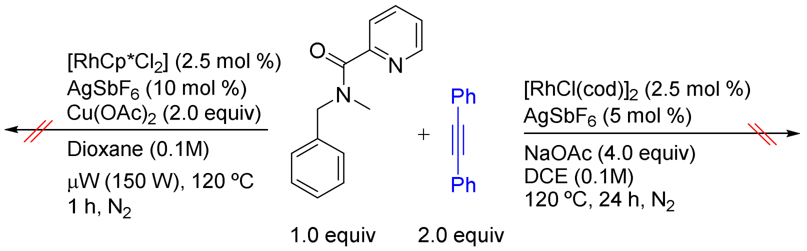

Starting material is recovered unaltered under both reaction systems

1.3. X-ray structure determination

In addition to the NMR and mass spectra, the structure elucidation of a representative example of each structural series has been determined by X-ray diffraction.

N-Benzyl-5,6,7,8-tetraphenylisoquinoline-1-carboxamide (2):

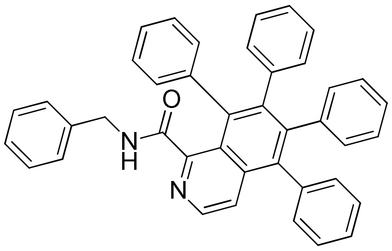

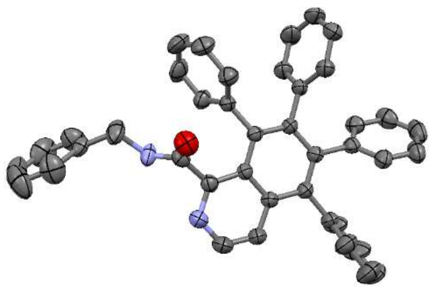

N-Benzyl-5,6,7,8-tetraphenyl-4-(trifluoromethyl)isoquinoline-1-carboxamide (16)

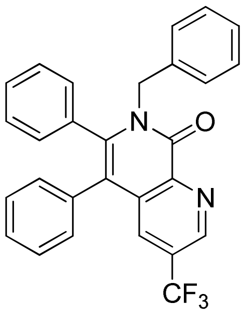

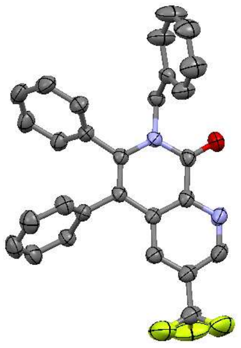

6-Benzyl-2-methyl-4,5-diphenylthieno[2,3-*c*]pyridin-7(6H)-one (18):

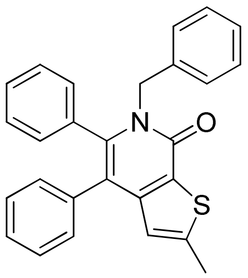

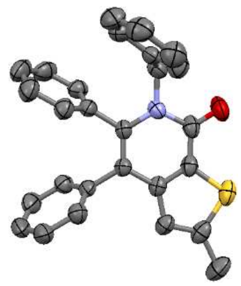

N-(2,6-bis((*E*)-1,2-diphenylvinyl)benzyl)picolinamide (3):

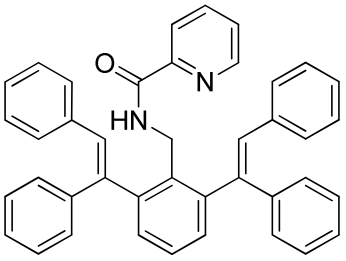

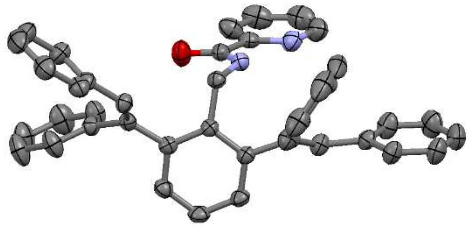

***N*-(2,6-Bis((*E*)-1,2-diphenylvinyl)phenethyl)picolinamide (73):**

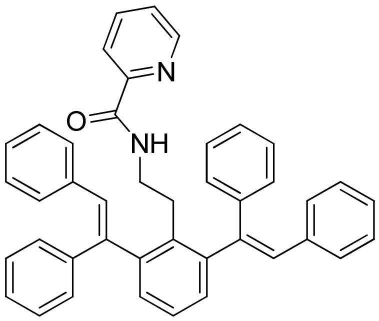

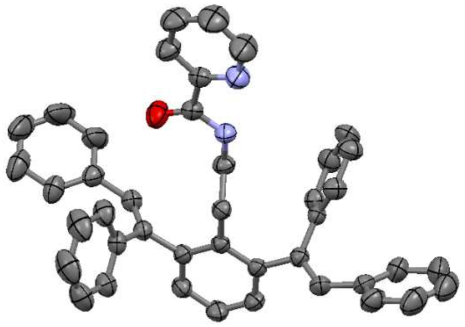

In the ORTEP view of these compounds, hydrogen atoms have been removed for simplicity.

2. Typical procedure for the protection of benzylamine derivatives

2.1. Synthesis of pyridinecarboxamide derivatives

Synthesis of *N*-benzylpicolinamide (1).[1] A 50 mL round-bottomed flask immersed in a 0 °C

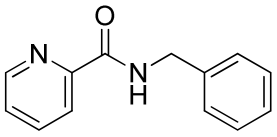

bath (ice and water) was charged with picolinic acid (616 mg, 5.0 mmol, 1.00 equiv) and CH_2Cl_2 (10 mL). To the stirred suspension was added oxalyl chloride (0.47 mL, 5.50 mmol, 1.10 equiv) dropwise over a 15-minute period followed by addition of DMF (0.10 mL, catalytic amount) in one portion, producing a rust-red color and the evolution of a gas. The mixture was kept in the cooling bath for 1 h and then allowed to warm to room temperature. After gas evolution ceased, the mixture was again cooled to 0 °C and NEt_3 (1.40 mL, 10.0 mmol) was added dropwise over a 15-minute period followed by benzylamine (0.60 mL, 5.50 mmol, 1.10 equiv) added dropwise over a 15-minute period. The brown mixture was left in the cooling bath for 30 minutes and then allowed to warm to room temperature. Stirring was continued at room temperature for 2 h. Removal of solvent *in vacuo* gave the crude product as a brown solid that was extracted with H_2O-CH_2Cl_2. The organic phases were combined and concentrated under reduced pressure to give **1** as a white solid; yield: 1.04 g (98%); mp= 219-221 °C. The analytical data (NMR, HRMS analysis) matched those reported in the literature for *N*-benzylpicolinamide [CAS: 18904-38-6]. **^{1}H NMR ($CDCl_3$, 300 MHz)** δ: 8.52 (ddd, *J* = 4.8, 1.7, 0.9 Hz, 1H, py-H^6), 8.39 (s, 1H), 8.24 (dt, *J* = 7.8, 1.1 Hz, 1H, py-H^3), 7.85 (td, *J* = 7.7, 1.7 Hz, 1H, py-H^4), 7.43 - 7.25 (m, 6H, py-H^5), 4.67 (d, 1H), 4.66 (d, 1H). **^{13}C NMR ($CDCl_3$, 75 MHz)** δ: 164.3 (C=O), 149.9 (py-C^1), 148.1 (py-C^6), 138.3, 137.4 (py-C^3), 128.8, 127.9, 127.5, 126.3 (py-C^5), 122.4 (py-C^2), 43.6. **ESI$^+$** calcd. for $C_{13}H_{13}N_2O$ $(M+H)^+$: 213.1022; Found: 213.1028.

***N*-Benzyl-6-methylpicolinamide (5)**. Compound **5** was prepared following the typical

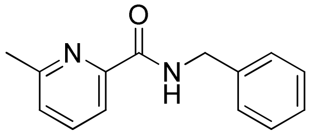

procedure from 6-methylpicolinic acid (685 mg, 5.00 mmol, 1.00 equiv), to give **5** as a pale orange solid; yield: 670 mg (59%); mp= 104-105 °C. **^{1}H NMR ($CDCl_3$, 300 MHz)** δ: 8.46 (s, 1H), 8.05 (d, *J* = 7.7 Hz, 1H), 7.73 (t, *J* = 7.7 Hz, 1H), 7.45 - 7.23 (m, 6H), 4.68 (d, *J* = 6.2 Hz, 2H), 2.55 (s, 3H). **^{13}C NMR ($CDCl_3$, 75 MHz)** δ: 164.5, 157.3, 149.2, 138.6, 137.7, 128.8, 128.0, 127.5, 126.1, 119.6, 43.5, 24.3. **EI$^+$** calcd. for $C_{14}H_{14}N_2O$ $(M)^+$: 226.1106; Found: 226.1112.

***N*-Benzyl-6-chloropicolinamide (6)**. Compound **6** was prepared following the typical

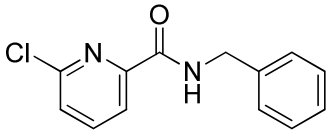

procedure from 6-chloropicolinic acid (788 mg, 5.00 mmol, 1.00 equiv), to give **6** as a pale orange solid; yield: 825 mg (67%); mp= 116-117 °C. **^{1}H NMR ($CDCl_3$, 300 MHz)** δ: 8.25 - 8.09 (m, 1H), 7.80 (t, *J* = 7.8 Hz, 1H), 7.44 (d, *J* = 8.0 Hz, 1H), 7.38 - 7.24 (m, 5H), 4.65 (d, *J* = 6.2 Hz, 2H). **^{13}C NMR ($CDCl_3$, 75 MHz)** δ: 162.9, 150.5, 150.1, 140.1, 138.0, 128.8, 127.9, 127.7, 127.1, 121.2, 43.6. **EI$^+$** calcd. for $C_{13}H_{11}ClN_2O$ $(M)^+$: 246.0560; Found: 246.0558.

---

[1](*a*) A. Jóźwiak, J. Z. Brzeziński, M. W. Płotka, A. K. Szcześniak, Z. Malinowski and J. Epsztajn, *Eur. J. Org. Chem.*, 2004, 3254; (*b*) H. Brunner, B. Nuber and M. Prommesberger, *J. Organomet. Chem.*, 1996, **523**, 179.

***N*-Benzyl-5-(trifluoromethyl)picolinamide (7)**. Compound **7** was prepared following the typical procedure from 5-(trifluoromethyl)picolinic acid (0.34 mL, 2.40 mmol, 1.00 equiv), to give **7** as a yellow solid; yield: 468 mg (71%); mp= 71-72 °C. **^{1}H NMR ($CDCl_3$, 300 MHz)** δ: 8.79 (s, 1H), 8.38 (d, *J* = 8.2 Hz, 1H), 8.33 (s, 1H), 8.11 (d, *J* = 8.2 Hz, 1H), 7.41 - 7.26 (m, 5H), 4.69 (d, *J* = 6.1 Hz, 2H). **^{13}C NMR ($CDCl_3$, 75 MHz)** δ: 163.0, 152.9, 145.3 (q, *J* = 3.9 Hz), 137.9, 134.9 (dd, *J* = 6.8, 3.4 Hz), 129.2, 128.9, 128.7, 127.9, 127.8, 122.3, 43.8. **ESI^{+}** calcd. for $C_{14}H_{12}F_3N_2O$ $(M+H)^+$: 281.0896; Found: 281.0897.

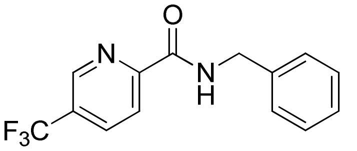

***N*-(4-Methoxybenzyl)-3-methylpicolinamide**. This compound was prepared following the typical procedure from 3-methylpicolinic acid (370 mg, 2.70 mmol, 1.00 equiv) and (4-methoxyphenyl)methanamine, (0.37 mg, 2.70 mmol, 1.00 equiv) to give a yellow oil; yield: 468 mg (71%). **^{1}H NMR ($CDCl_3$, 300 MHz)** δ: 8.48 (s, 1H), 8.35 (s, 1H), 7.57 (d, *J* = 7.3 Hz, 1H), 7.39 - 7.26 (m, 3H), 6.89 (d, *J* = 8.7 Hz, 2H), 4.58 (d, *J* = 5.9 Hz, 2H), 3.79 (s, 3H), 2.79 (s, 3H). **^{13}C NMR ($CDCl_3$, 75 MHz)** δ: 165.7, 158.9, 147.2, 145.3, 140.7, 135.3, 130.6, 129.0, 125.5, 114.0, 55.2, 42.6, 20.4. **EI^{+}** calcd. for $C_{15}H_{16}N_2O_2$ $(M+H)^+$: 256.1212; Found: 256.1220.

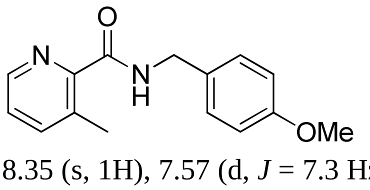

***N*-(4-(Methylthio)benzyl)picolinamide (20)** Compound **20** was prepared following the general protocol from (4-(methylthio)phenyl)methanamine (250 mg, 1.63 mmol, 1.10 equiv), to give **20** as a white solid; yield: 272 mg (65%); mp= 66-68 °C. **^{1}H NMR ($CDCl_3$, 300 MHz)** δ: 8.42 (d, *J* = 4.2 Hz, 1H), 8.24 (s, 1H), 8.13 (d, *J* = 7.8 Hz, 1H), 7.75 (t, *J* = 7.6 Hz, 1H), 7.35 - 7.29 (m, 1H), 7.16 (dd, *J* = 17.8, 8.2 Hz, 1H), 4.52 (d, *J* = 6.0 Hz, 1H), 2.37 (s, 1H). **^{13}C NMR ($CDCl_3$, 75 MHz)** δ: 164.3, 149.8, 148.1, 137.6, 137.4, 135.2, 128.5, 127.0, 126.3, 122.4, 43.1, 16.0. **EI^{+}** calcd. for $C_{14}H_{14}N_2OS$ $(M)^+$: 258.0827; Found: 258.0834.

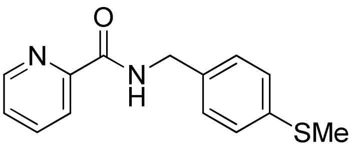

***N*-(4-Methoxybenzyl)picolinamide (21)**. Compound **21** was prepared following the typical procedure from (4-methoxyphenyl)methanamine (0.65 mL, 5.00 mmol, 1.00 equiv), to give **21** as a white solid; yield: 758 mg (63%); mp= 52-53 °C. **^{1}H NMR ($CDCl_3$, 300 MHz)** δ: 8.88 (d, *J* = 4.7 Hz, 1H), 8.71 (s, 1H), 8.60 (d, *J* = 8.6 Hz, 1H), 8.21 (t, *J* = 7.7 Hz, 1H), 7.77 (s, 1H), 7.67 (d, *J* = 8.6 Hz, 2H), 7.25 (d, *J* = 8.7 Hz, 2H), 4.98 (d, *J* = 6.0 Hz, 2H), 4.16 (s, 3H). **^{13}C NMR ($CDCl_3$, 75 MHz)** δ: 164.2, 159.1, 150.0, 148.1, 137.4, 130.4, 129.27, 126.2, 122.4, 114.2, 55.3, 43.0. **ESI^{+}** calcd. for $C_{14}H_{15}N_2O_2$ $(M+H)^+$: 243.1128; Found: 243.1138.

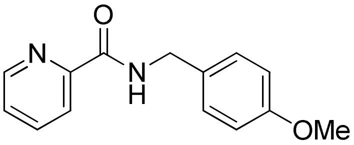

***N*-(4-Methylbenzyl)picolinamide (22)**. Compound **22** was prepared following the typical procedure from *p*-tolylmethanamine (0.70 mL, 5.50 mmol, 1.10 equiv), to give **22** as a pale yellow solid; yield: 926 mg (82%); mp= 87-88 °C. **^{1}H NMR (acetone-d_6, 300 MHz)** δ: 8.75 (s, 1H), 8.57 (d, *J* = 4.7 Hz, 1H), 8.16 (d, *J* = 7.8 Hz, 1H), 7.96 (t, *J* = 7.7 Hz, 1H), 7.57 - 7.50 (m, 1H), 7.29 (d, *J* = 7.7 Hz, 2H), 7.13 (d, *J* = 7.6 Hz, 2H), 4.61 (d, *J* = 6.2 Hz, 2H), 2.29 (s, 3H). **^{13}C NMR (acetone-d_6, 75 MHz)** δ: 164.5, 151.3, 149.1, 138.3, 137.4, 137.2, 129.8, 128.5, 127.1, 122.7, 43.3, 21.0. **EI^{+}** calcd. for $C_{14}H_{14}N_2O$ $(M)^+$: 226.1106; Found: 226.1108.

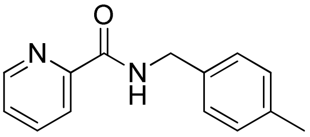

***N*-(4-Chlorobenzyl)picolinamide (23)**. Compound **23** was prepared following the typical procedure from (4-chlorophenyl)methanamine (0.67 mL, 5.50 mmol, 1.10 equiv), to give **23** as a pale yellow solid; yield: 946 mg (70%); mp= 87-88 °C. **^{1}H NMR (acetone-d$_6$, 300 MHz)** δ: 8.90 (s, 1H), 8.59 (d, *J* = 5.5 Hz, 1H), 8.15 (d, *J* = 7.8 Hz, 1H), 7.98 (t, *J* = 7.7 Hz, 1H), 7.55 (ddd, *J* = 7.6, 4.8, 1.3 Hz, 1H), 7.42 (d, *J* = 8.7 Hz, 2H), 7.34 (d, *J* = 8.6 Hz, 2H), 4.65 (d, *J* = 6.5 Hz, 2H).**^{13}C NMR (acetone-d$_6$, 75 MHz)** δ: 164.8, 151.1, 149.2, 139.6, 138.3, 133.0, 130.2, 129.2, 127.2, 122.8, 42.9. **EI$^+$** calcd. for $C_{13}H_{11}ClN_2O$ $(M)^+$: 246.0560; Found: 246.0570.

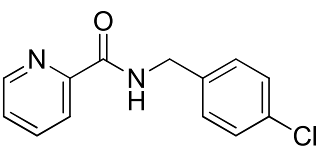

***N*-(4-Fluorobenzyl)picolinamide (24)**. Compound **24** was prepared following the typical procedure from (4-fluorophenyl)methanamine (0.60 mL, 5.50 mmol, 1.10 equiv), to give **24** as a yellow oil; yield: 945 mg (82%); **^{1}H NMR (acetone-d$_6$, 300 MHz)** δ: 8.89 (s, 1H), 8.57 (d, *J* = 4.5 Hz, 1H), 8.17 (d, *J* = 7.8 Hz, 1H), 7.96 (t, *J* = 7.6 Hz, 1H), 7.57 - 7.48 (m, 1H), 7.49 - 7.38 (m, 2H), 7.07 (t, *J* = 8.6 Hz, 2H), 4.65 (d, *J* = 6.4 Hz, 2H). **^{13}C NMR (acetone-d$_6$, 75 MHz)** δ: 164.8, 162.7 (d, *J* = 243.0 Hz), 151.1, 149.1, 138.3, 136.6 (d, *J* = 3.1 Hz), 130.4 (d, *J* = 8.1 Hz), 127.1, 122.8, 115.7 (d, *J* = 21.5 Hz), 42.8. **EI$^+$** calcd. for $C_{13}H_{11}FN_2O$ $(M)^+$: 230.0855; Found: 230.0850.

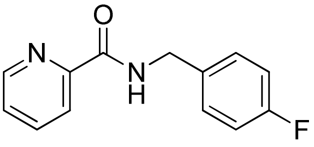

***N*-(4-(Trifluoromethyl)benzyl)picolinamide (25)**. Compound **25** was prepared following the typical procedure from (4-(trifluoromethyl)phenyl)-methanamine (0.78 mL, 5.50 mmol, 1.10 equiv), to give **25** as a yellow solid; yield: 1.02 g (67%); mp= 83-84 °C. **^{1}H NMR ($CDCl_3$, 300 MHz)** δ: 8.61 - 8.41 (m, 1H), 8.22 (d, *J* = 7.8 Hz, 1H), 7.85 (t, *J* = 7.7 Hz, 1H), 7.56 (t, *J* = 10.1 Hz, 2H), 7.53 - 7.38 (m, 3H), 4.72 (d, *J* = 6.2 Hz, 2H). **^{13}C NMR ($CDCl_3$, 75 MHz)** δ: 164.6, 149.7, 148.3, 142.6, 137.6, 128.1, 126.5, 125.7 (q, *J* = 3.8 Hz), 122.5, 43.1. **ESI$^+$** calcd. for $C_{14}H_{12}F_3N_2O$ $(M+H)^+$: 281.0896; Found: 281.0886.

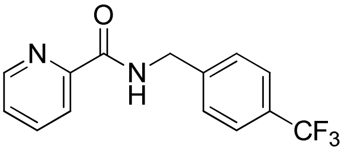

***N*-(4-Cyanobenzyl)picolinamide (26)**. Compound **26** was prepared following the typical procedure from 4-(aminomethyl)benzonitrile (927 mg, 5.50 mmol, 1.10 equiv), to give **26** as a white solid; yield: 785 mg (66%); mp= 128-130 °C. **^{1}H NMR (acetone-d$_6$, 300 MHz)** δ: 9.04 (s, 1H), 8.60 (d, *J* = 4.7 Hz, 1H), 8.15 (d, *J* = 7.8 Hz, 1H), 7.98 (td, *J* = 7.7, 1.7 Hz, 1H), 7.72 (d, *J* = 8.3 Hz, 2H), 7.62 - 7.53 (m, 3H), 4.75 (d, *J* = 6.5 Hz, 2H). **^{13}C NMR (acetone-d$_6$, 75 MHz)** δ: 165.1, 151.0, 149.2, 146.3, 138.4, 133.0, 129.2, 127.3, 122.8, 119.3, 111.4, 43.3. **EI$^+$** calcd. for $C_{14}H_{11}N_3O$ $(M)^+$: 237.0902; Found: 237.0907.

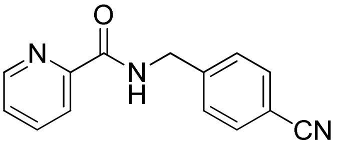

Methyl 4-(picolinamidomethyl)benzoate (27). Compound **27** was prepared following the typical procedure from methyl 4-(aminomethyl)benzoate (908mg, 5.50 mmol, 1.10 equiv), to give **27** as a white solid; yield: 1.08 g (73%)%); mp= 85-86 °C. **^{1}H NMR ($CDCl_3$, 300 MHz)** δ: 8.51 (s, 1H), 8.46 (d, *J* = 4.2 Hz, 1H), 8.17 (d, *J* = 7.8 Hz, 1H), 7.94 (d, *J* = 8.1 Hz, 2H), 7.79 (t, *J* = 7.7 Hz, 1H), 7.36 (d, *J* = 8.1 Hz, 3H), 4.67 (d, *J* = 6.2 Hz, 2H), 3.83 (s, 3H). **^{13}C NMR ($CDCl_3$, 75 MHz)** δ: 166.7, 164.4, 149.6, 148.1, 143.6, 137.3, 129.9, 129.2, 127.4, 126.3, 122.3, 52.0, 43.0. **EI$^+$** calcd. for $C_{15}H_{14}N_2O_3$ $(M)^+$: 270.1004; Found: 270.1011.

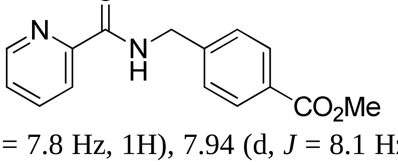

***N*-(3-Methylbenzyl)picolinamide (28)**. Compound **28** was prepared following the typical procedure from *m*-tolylmethanamine (0.69 mL, 5.50 mmol, 1.10 equiv), to give **28** as a white solid; yield: 893 mg (79%); mp= 63-64 °C. **^{1}H NMR (acetone-d$_6$, 300 MHz)** δ: 8.77 (s, 1H), 8.58 (d, *J* = 4.8 Hz, 1H), 8.17 (d, *J* = 7.8 Hz, 1H), 7.97 (t, *J* = 7.7 Hz, 1H), 7.54 (ddd, *J* = 7.6, 4.8, 1.2 Hz, 1H), 7.23 - 7.14 (m, 3H), 7.09 - 7.01 (m, 1H), 4.62 (d, *J* = 6.4 Hz, 2H), 2.29 (s, 3H). **^{13}C NMR (acetone-d$_6$, 75 MHz)** δ: 164.6, 151.3, 149.1, 140.3, 138.6, 138.3, 129.1, 129.1, 128.5, 127.1, 125.5, 122.7, 43.5, 21.4. **EI$^+$** calcd. for $C_{14}H_{14}N_2O$ $(M)^+$: 226.1106; Found: 226.1105.

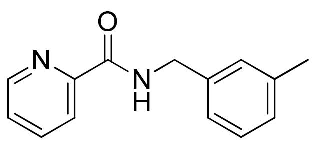

***N*-(3-(Trifluoromethyl)benzyl)picolinamide (29)**. Compound **29** was prepared following the typical procedure from (3-(trifluoromethyl)phenyl)methanamine (0.80 mL, 5.50 mmol, 1.10 equiv), to give **29** as a colorless oil; yield: 952 mg (68%). **^{1}H NMR (acetone-d$_6$, 300 MHz)** δ: 9.04 (s, 1H), 8.60 (d, *J* = 4.7 Hz, 1H), 8.16 (d, *J* = 7.8 Hz, 1H), 7.98 (td, *J* = 7.7, 1.7 Hz, 1H), 7.76 (s, 1H), 7.71 (d, *J* = 6.8 Hz, 1H), 7.62 - 7.51 (m, 3H), 4.76 (d, *J* = 6.5 Hz, 2H). **^{13}C NMR (acetone-d$_6$, 75 MHz)** δ: 165.0, 151.1, 149.2, 142.2, 138.4, 132.4, 132.4, 130.9 (d, *J* = 31.8 Hz), 130.1, 127.2, 125.3 (d, *J* = 271.4 Hz), 125.1 (q, *J* = 3.9 Hz), 124.5 (q, *J* = 3.9 Hz), 122.9, 43.2. **EI$^+$** calcd. for $C_{14}H_{11}F_3N_2O$ $(M)^+$: 280.0823; Found: 280.0811.

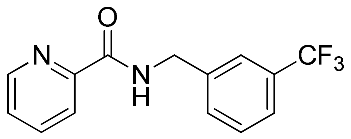

***N*-(2-Methylbenzyl)picolinamide (30)**. Compound **30** was prepared following the typical procedure from *o*-tolylmethanamine (0.68 mL, 5.50 mmol, 1.10 equiv), to give **30** as a pale orange solid; yield: 712 mg (63%); mp= 89-90 °C. **^{1}H NMR (acetone-d$_6$, 300 MHz)** δ: 8.62 (s, 1H), 8.58 (d, *J* = 3.8 Hz, 1H), 8.17 (d, *J* = 7.8 Hz, 1H), 7.96 (t, *J* = 7.7 Hz, 1H), 7.58 - 7.49 (m, 1H), 7.34 (t, *J* = 3.6 Hz, 1H), 7.20 - 7.13 (m, 3H), 4.65 (d, *J* = 6.2 Hz, 2H), 2.37 (s, 3H). **^{13}C NMR (acetone-d$_6$, 75 MHz)** δ: 164.5, 151.2, 149.1, 138.3, 137.8, 136.7, 130.9, 128.7, 127.9, 127.1, 126.7, 122.7, 41.5, 19.1. **EI$^+$** calcd. for $C_{14}H_{14}N_2O$ $(M)^+$: 226.1106; Found: 226.1112.

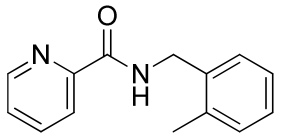

***N*-(2-Bromobenzyl)picolinamide (31).** Compound **31** was prepared following the typical procedure from (2-bromophenyl)methanamine hydrochloride (900 mg, 4.00 mmol, 1.10 equiv), to give **31** as a pale brown solid; yield: 856 mg (59%); mp= 91-92 °C. **^{1}H NMR (acetone-d$_6$, 300 MHz)** δ: 8.88 (s, 1H), 8.61 (d, *J* = 4.7 Hz, 1H), 8.16 (d, *J* = 7.8 Hz, 1H), 7.98 (t, *J* = 7.7 Hz, 1H), 7.51 (m, 2H), 7.44 (d, *J* = 7.6 Hz, 1H), 7.32 (t, *J* = 7.5 Hz, 1H), 7.19 (t, *J* = 7.6 Hz, 1H), 4.73 (d, *J* = 6.4 Hz, 2H). **^{13}C NMR (acetone-d$_6$, 75 MHz)** δ: 164.8, 150.9, 149.2, 138.9, 138.3, 133.3, 130.0, 129.7, 128.5, 127.2, 123.5, 122.8, 44.0. **EI$^+$** calcd. for $C_{13}H_{11}BrN_2O$ $(M)^+$: 290.0055; Found: 290.0055.

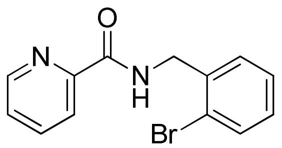

***N*-(2-Fluorobenzyl)picolinamide (32)**. Compound **32** was prepared following the typical procedure from (2-fluorophenyl)methanamine (0.60 mL, 5.50 mmol, 1.10 equiv), to give **32** as an orange oil; yield: 598 mg (52%); **^{1}H NMR (acetone-d$_6$, 300 MHz)** δ: 8.80 (s, 1H), 8.60 (d, *J* = 4.8 Hz, 1H), 8.15 (d, *J* = 7.8 Hz, 1H), 7.98 (td, *J* = 7.7, 1.7 Hz, 1H), 7.55 (ddd, *J* = 7.6, 4.8, 1.3 Hz, 1H), 7.49 - 7.41 (m, 1H), 7.35 - 7.25 (m, 1H), 7.13 (m, 2H), 4.73 (d, *J* = 6.4 Hz, 2H). **^{13}C NMR (acetone-d$_6$, 75 MHz)** δ: 164.8, 161.63 (d, *J* = 244.6 Hz), 151.0, 149.2, 138.3, 130.5 (d, *J* = 4.4 Hz), 129.8 (d, *J* = 8.2 Hz), 127.2, 127.0, 125.1 (d, *J* = 3.6 Hz), 122.8, 115.8 (d, *J* = 21.5 Hz). **EI$^+$** calcd. for $C_{13}H_{11}FN_2O$ $(M)^+$: 230.0855; Found: 230.0857.

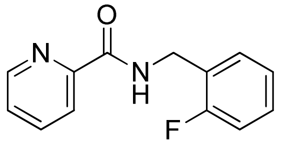

***N*-(Furan-2-ylmethyl)picolinamide (33)**. Compound **33** was prepared following the typical procedure from furan-2-ylmethanamine (0.50 mL, 5.50 mmol, 1.10 equiv), to give **33** as a white solid; yield: 667 mg (60%); mp= 105-107 °C. **^{1}H NMR ($CDCl_3$, 300 MHz)** δ: 8.51 (d, *J* = 4.7 Hz, 1H), 8.33 (s, 1H), 8.19 (d, *J* = 7.8 Hz, 1H), 7.81 (t, *J* = 7.7 Hz, 1H), 7.46 - 7.29 (m, 2H), 6.34 - 6.21 (m, 2H), 4.64 (d, *J* = 5.9 Hz, 2H). **^{13}C NMR ($CDCl_3$, 75 MHz)** δ: 164.2, 151.3, 149.8, 148.1, 142.3, 137.4, 126.3, 122.4, 110.5, 107.5, 36.5. **ESI$^+$** calcd. for $C_{11}H_{11}N_2O_2$ $(M+H)^+$: 203.0815; Found: 203.0823.

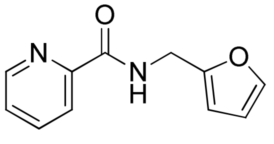

2.2. Synthesis of *N*-benzyl-2-heteroaryl carboxamide derivatives

Synthesis of *N*-benzylquinoline-2-carboxamide (8). Compound **8** was prepared following the typical procedure for the synthesis of pyridinecarboxamide derivatives but from quinoline-2-carboxylic acid (960 mg, 5.00 mmol, 1.00 equiv), to give **8** as a pale orange solid; yield: 720 mg (55%); mp= 123-124 °C. **^{1}H NMR ($CDCl_3$, 300 MHz)** δ: 8.60 (s, 1H), 8.34 (d, *J* = 4.1 Hz, 2H), 8.07 (d, *J* = 8.5 Hz, 1H), 7.88 (d, *J* = 8.2 Hz, 1H), 7.82 - 7.69 (m, 1H), 7.61 (t, *J* = 7.5 Hz, 1H), 7.47 - 7.26 (d, *J* = 55.9 Hz, 5H), 4.75 (d, *J* = 6.1 Hz, 2H). **^{13}C NMR ($CDCl_3$, 75 MHz)** δ: 164.5, 149.7, 146.5, 138.4, 137.5, 130.1, 129.7, 129.3, 128.7, 127.9, 127.7, 127.5, 118.9, 43.6. **ESI$^+$** calcd. for $C_{17}H_{15}N_2O$ $(M+H)^+$: 263.1178; Found: 263.1186.

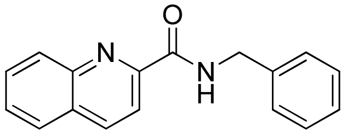

***N*-Benzyl-5-methylthiophene-2-carboxamide (9)**. Compound **9** was prepared following the typical procedure from 5-methylthiophene-2-carboxylic acid (711 mg, 5.00 mmol, 1.00 equiv), to give **9** as a yellow solid; yield: 885 mg (76%); mp= 145-146 °C. **^{1}H NMR (acetone-d_6, 300 MHz)** δ: 8.07 (s, 1H), 7.54 (d, *J* = 3.7 Hz, 1H), 7.41 - 7.15 (m, 6H), 6.89 - 6.66 (m, 1H), 4.54 (d, *J* = 6.1 Hz, 2H), 2.48 (d, *J* = 0.8 Hz, 4H). **^{13}C NMR ($CDCl_3$, 75 MHz)** δ: 162.0, 145.4, 138.4, 136.2, 128.8, 128.6, 127.9, 127.6, 126.1, 43.9, 15.7. **EI$^+$** calcd. for $C_{13}H_{13}NOS$ $(M)^+$: 231.0718; Found: 231.0719.

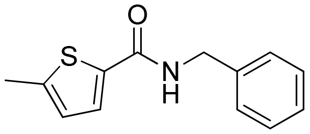

***N*-Benzylbenzo[*b*]thiophene-2-carboxamide (10)**. Compound **10** was prepared following the typical procedure from benzo[*b*]thiophene-2-carboxylic acid (891 mg, 5.00 mmol, 1.00 equiv), to give **10** as a yellow solid; yield: 909 mg (68%); mp= 146-147 °C. **^{1}H NMR ($CDCl_3$, 300 MHz)** δ: 7.89 - 7.75 (m, 3H), 7.47 - 7.27 (m, 7H), 6.44 (s, 1H), 4.67 (d, *J* = 5.7 Hz, 2H). **^{13}C NMR ($CDCl_3$, 75 MHz)** δ: 162.3, 141.0, 139.2, 138.3, 138.0, 129.0, 128.1, 127.9, 126.5, 125.5, 125.2, 125.1, 122.9, 44.4. **EI$^+$** calcd. for $C_{16}H_{13}NOS$ $(M)^+$: 267.0718; Found: 267.0706.

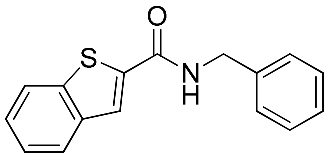

3. Typical procedure for the protection of phenethyl derivatives

Synthesis of *N*-phenethylpicolinamide (61).[1] A 50 mL round-bottomed flask immersed in a 0 °C bath (ice and water) was charged with picolinic acid (616 mg, 5.00 mmol, 1.00 equiv) and CH_2Cl_2 (10 mL). To the stirred suspension was added oxalyl chloride (0.472 mL, 5.50 mmol, 1.10 equiv) dropwise over a 15-minute period followed by addition of DMF (0.10 mL, catalytic amount) in one portion, producing a rust-red color and the evolution of a gas. The mixture was kept in the cooling bath for 1 h and then allowed to warm to room temperature. After gas evolution ceased, the mixture was again cooled to 0 °C and NEt_3 (1.40 mL, 10.0 mmol) was added dropwise over a 15-minute period followed by 2-phenylethanamine (0.63 mL, 5.00 mmol, 1.00 equiv) added dropwise over a 15-minute period. The brown mixture was left in the cooling bath for 30 minutes and then allowed to warm to room temperature. Stirring was continued at room temperature for 2 h. Removal of solvent *in vacuo* gave the crude product as a brown solid that was extracted with H_2O-CH_2Cl_2. The organic phases were combined and concentrated under reduced pressure to give **61**as a yellow oil; yield: 789 mg (70%). **^{1}H NMR ($CDCl_3$, 300 MHz)** δ: 8.52 (d, *J* = 4.7 Hz, 1H), 8.35 - 8.13 (m, 2H), 7.84 (t, *J* = 7.7 Hz, 1H), 7.46 - 7.37 (m, 1H), 7.37 - 7.22 (m, 5H), 3.78 (dd, *J* = 13.6, 7.1 Hz, 2H), 2.98 (t, *J* = 7.3 Hz, 2H). **^{13}C NMR ($CDCl_3$, 75 MHz)** δ: 164.25, 149.89, 147.99, 138.92, 137.22, 128.71, 128.54, 126.39, 126.01, 122.07, 40.70, 35.88. **EI$^+$** calcd. for $C_{14}H_{14}N_2O$ $(M)^+$: 226.1106; Found: 226.1110.

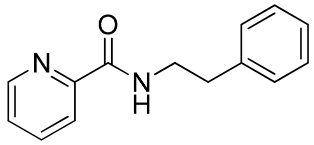

***N*-(4-Methoxyphenethyl)picolinamide (62).** Compound **62** was prepared following the general protocol from 2-(4-methoxyphenyl)ethan-1-amine (0.80 mL, 5.50 mmol, 1.10 equiv),to give **62** as a pale orange solid; yield: 1.06 g (83%); mp= 56-58 °C. **^{1}H NMR (acetone-d_6, 300 MHz)** δ: 8.56 (d, *J* = 4.7 Hz, 1H), 8.39 (s, 1H), 8.13 (d, *J* = 7.8 Hz, 1H), 7.95 (td, *J* = 7.7, 1.7 Hz, 1H), 7.52 (ddd, *J* = 7.6, 4.8, 1.3 Hz, 1H), 7.20 (d, *J* = 8.7 Hz, 2H), 6.86 (d, *J* = 8.7 Hz, 2H), 3.76 (s, 3H), 3.66 (dd, *J* = 14.6, 6.3 Hz, 2H), 2.88 (t, *J* = 7.4 Hz, 2H). **^{13}C NMR (acetone-d_6, 75 MHz)** δ: 164.5, 159.2, 151.3, 149.1, 138.2, 132.2, 130.5, 127.0, 122.6, 114.7, 55.4, 41.6, 35.6. **EI$^+$** calcd. for $C_{15}H_{16}N_2O_2$ $(M)^+$: 256.1212; Found: 256.1215.

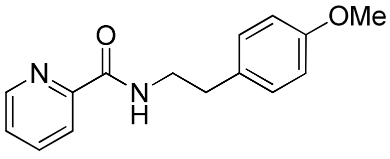

***N*-(4-Chlorophenethyl)picolinamide (63).** Compound **63** was prepared following the general protocol from 2-(4-chlorophenyl)ethan-1-amine (0.77 mL, 5.50 mmol, 1.10 equiv), to give **63** as a white solid; yield: 1.03 g (79%); mp= 87-88 °C. **^{1}H NMR (acetone-d_6, 300 MHz)** δ: 8.56 (d, *J* = 4.1 Hz, 1H), 8.44 (s, 1H), 8.12 (dd, *J* = 7.8, 1.0 Hz, 1H), 7.96 (td, *J* = 7.7, 1.7 Hz, 1H), 7.53 (ddd, *J* = 7.5, 4.8, 1.2 Hz, 1H), 7.31 (s, 4H), 3.69 (dd, *J* = 13.9, 6.8 Hz, 2H), 2.96 (t, *J* = 7.3 Hz, 2H). **^{13}C NMR (acetone-d_6, 75 MHz)** δ: 164.6, 151.2, 149.1, 139.4, 138.3, 132.3, 131.3, 129.2, 127.0, 122.6, 41.2, 35.8. **EI$^+$** calcd. for $C_{14}H_{13}ClN_2O$ $(M)^+$: 260.0716; Found: 260.0706.

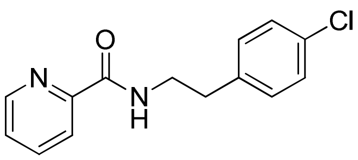

***N*-(4-Fluorophenethyl)picolinamide (64).** Compound **64** was prepared following the general protocol from 2-(4-fluorophenyl)ethan-1-amine (0.70 mL, 5.50 mmol, 1.10 equiv), to give **64** as a white solid; yield: 1060 mg (87%); mp= 58-59 °C. **^{1}H NMR (acetone-d$_6$, 300 MHz)** δ: 8.57 (d, *J* = 3.9 Hz, 1H), 8.44 (s, 1H), 8.12 (d, *J* = 8.8 Hz, 1H), 7.96 (td, *J* = 7.7, 1.7 Hz, 1H), 7.54 (ddd, *J* = 7.5, 4.8, 1.3 Hz, 1H), 7.35 - 7.29 (m, 2H), 7.06 (d, *J* = 34.8 Hz, 2H), 3.68 (dd, *J* = 13.9, 7.0 Hz, 2H), 2.95 (t, *J* = 7.3 Hz, 2H). **^{13}C NMR (acetone-d$_6$, 75 MHz)** δ: 164.6, 162.3 (d, *J* = 241.9 Hz), 151.2, 149.1, 138.3, 136.5 (d, *J* = 3.1 Hz), 131.3 (d, *J* = 7.9 Hz), 127.0, 122.6, 115.8 (d, *J* = 21.2 Hz), 41.4, 35.6. **EI$^+$** calcd. for $C_{14}H_{13}FN_2O$ (M)$^+$: 244.1012; Found: 244.1015.

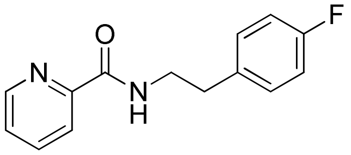

***N*-(3-Methoxyphenethyl)picolinamide (65).** Compound **65** was prepared following the general protocol from 2-(3-methoxyphenyl)ethan-1-amine (0.80 mL, 5.50 mmol, 1.10 equiv), to give **65** as a yellow oil; yield: 922 mg (72%). **^{1}H NMR (acetone-d$_6$, 300 MHz)** δ: 8.59 - 8.53 (m, 1H), 8.42 (s, 1H), 8.14 (d, *J* = 7.8 Hz, 1H), 7.96 (tt, *J* = 7.7, 1.9 Hz, 1H), 7.52 (ddd, *J* = 7.5, 4.8, 1.2 Hz, 1H), 7.21 (t, *J* = 7.9 Hz, 1H), 6.91 - 6.82 (m, 2H), 6.81 - 6.73 (m, 1H), 3.76 (s, 3H), 3.70 (dd, *J* = 13.8, 7.1 Hz, 2H), 2.93 (t, *J* = 7.3 Hz, 2H). **^{13}C NMR (acetone-d$_6$, 75 MHz)** δ: 164.5, 160.8, 151.3, 149.1, 141.9, 138.3, 130.2, 127.0, 122.6, 121.7, 115.1, 112.6, 55.3, 41.3, 36.5. **EI$^+$** calcd. for $C_{15}H_{16}N_2O_2$ (M)$^+$: 256.1212; Found: 256.1204.

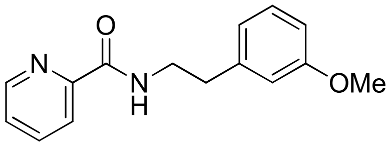

***N*-(3-Methylphenethyl)picolinamide (66).** Compound **66** was prepared following the general protocol from 2-(*m*-tolyl)ethan-1-amine (0.79 mL, 5.50 mmol, 1.10 equiv), to give **66** as a yellow oil; yield: 826 mg (69%). **^{1}H NMR (acetone-d$_6$, 300 MHz)** δ: 8.56 (d, *J* = 4.0 Hz, 1H), 8.41 (s, 1H), 8.13 (d, *J* = 7.8 Hz, 1H), 7.96 (td, *J* = 7.7, 1.7 Hz, 1H), 7.53 (ddd, *J* = 7.6, 4.8, 1.2 Hz, 1H), 7.23 - 6.97 (ddd, *J* = 23.9, 15.7, 7.4 Hz, 4H), 3.68 (dd, *J* = 14.8, 6.3 Hz, 2H), 2.94 - 2.88 (t, *J* = 7.0 Hz, 2H), 2.29 (s, 3H). **^{13}C NMR (acetone-d$_6$, 75 MHz)** δ: 164.5, 151.3, 149.1, 140.3, 138.6, 138.3, 130.3, 129.1, 127.7, 127.0, 126.6, 122.6, 41.4, 36.5, 21.3. **EI$^+$** calcd. for $C_{15}H_{16}N_2O$ (M)$^+$: 240.1263; Found: 240.1267.

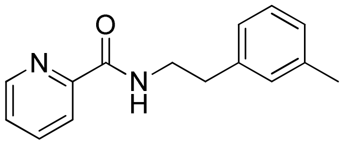

***N*-(2-Methoxyphenethyl)picolinamide (67).** Compound **67** was prepared following the general protocol from 2-(2-methoxyphenyl)ethan-1-amine (0.81 mL, 5.50 mmol, 1.10 equiv), to give **67** as a yellow oil; yield: 691 mg (54%). **^{1}H NMR (acetone-d$_6$, 300 MHz)** δ: 8.58 (d, *J* = 5.3 Hz, 1H), 8.45 (s, 1H), 8.11 (d, *J* = 7.8 Hz, 1H), 7.94 (td, *J* = 7.7, 1.7 Hz, 1H), 7.52 (ddd, *J* = 7.6, 4.8, 1.2 Hz, 1H), 7.25 - 7.15 (dd, *J* = 11.7, 4.5 Hz, 2H), 6.96 (d, *J* = 7.9 Hz, 1H), 6.87 (td, *J* = 7.5, 1.0 Hz, 1H), 3.86 (s, 3H), 3.66 (dd, *J* = 12.8, 7.0 Hz, 2H), 2.96 (t, *J* = 7.0 Hz, 2H). **^{13}C NMR (acetone-d$_6$, 75 MHz)** δ: 164.5, 158.6, 151.4, 149.1, 138.2, 131.1, 128.5, 128.5, 126.9, 122.5, 121.3, 111.2, 55.7, 40.5, 30.8. **EI$^+$** calcd. for $C_{15}H_{16}N_2O_2$ (M)$^+$: 256.1212; Found: 256.1209.

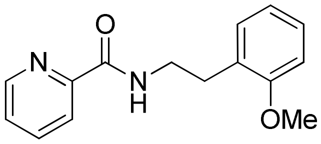

***N*-(2-Methylphenethyl)picolinamide (68).** Compound **68** was prepared following the general protocol from from 2-(*o*-tolyl)ethan-1-amine (0.77 mL, 5.50 mmol, 1.10 equiv), to give **68** as an yellow oil; yield: 804 mg (67%). **^{1}H NMR (acetone-d$_6$, 300 MHz)** δ: 8.61 - 8.44 (m, 2H), 8.15 (d, *J* = 7.8 Hz, 1H), 7.95 (td, *J* = 7.7, 1.7 Hz, 1H), 7.52 (ddd, *J* = 7.5, 4.8, 1.2 Hz, 1H), 7.26 - 7.08 (m, 4H), 3.66 (dd, *J* = 15.4, 6.2 Hz, 2H), 3.02 - 2.91 (m, 2H), 2.38 (s, 3H). **^{13}C NMR (acetone-d$_6$, 75 MHz)** δ: 164.6, 151.3, 149.1, 138.4, 138.2, 137.0, 131.0, 130.1,

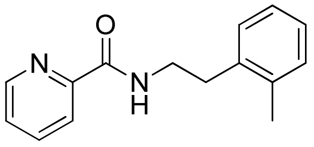

127.1, 126.9, 126.8, 122.6, 40.4, 34.0, 19.3. **EI$^+$** calcd. for $C_{15}H_{16}N_2O$ $(M)^+$: 240.1263; Found: 240.1263.

***N*-(2-Bromophenethyl)picolinamide (69).** Compound **69** was prepared following the general protocol from 2-(2-bromophenyl)ethan-1-amine (0.79 mL, 5.50 mmol, 1.10 equiv), to give **69** as a brown oil; yield: 1.14 g (75%).

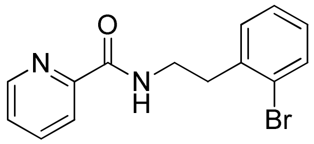

^{1}H NMR (acetone-d$_6$, 300 MHz) δ: 8.61 - 8.43 (m, 2H), 8.14 (d, *J* = 7.8 Hz, 1H), 7.94 (t, *J* = 7.7 Hz, 1H), 7.57 (d, *J* = 7.9 Hz, 1H), 7.55 - 7.48 (m, 1H), 7.38 (d, *J* = 7.6 Hz, 1H), 7.28 (t, *J* = 7.4 Hz, 1H), 7.13 (t, *J* = 7.6 Hz, 1H), 3.75 (dd, *J* = 14.2, 6.6 Hz, 2H), 3.12 (t, *J* = 7.3 Hz, 2H). **^{13}C NMR (acetone-d$_6$, 75 MHz)** δ: 164.6, 151.2, 149.0, 139.6, 138.2, 133.5, 131.8, 129.1, 128.5, 126.9, 125.0, 122.5, 39.7, 36.6. **EI$^+$** calcd. for $C_{14}H_{13}BrN_2O$ $(M)^+$: 304.0211; Found: 304.0207.

***N*-(2-Chlorophenethyl)picolinamide (70).** Compound **70** was prepared following the general protocol from 2-(2-chlorophenyl)ethan-1-amine (0.77 mL, 5.50 mmol, 1.10 equiv), to give **70** as a yellow oil; yield: 910 mg (70%).

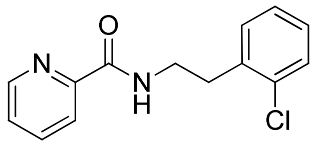

^{1}H NMR (acetone-d$_6$, 300 MHz) δ: 8.57 (d, *J* = 5.5 Hz, 1H), 8.54 - 8.43 (m, 1H), 8.12 (d, *J* = 7.8 Hz, 1H), 7.96 (t, *J* = 7.7 Hz, 1H), 7.57 - 7.49 (d, *J* = 26.2 Hz, 1H), 7.44 - 7.33 (m, 2H), 7.28 - 7.20 (m, 2H), 3.74 (dd, *J* = 13.6, 7.2 Hz, 2H), 3.11 (t, *J* = 7.3 Hz, 2H). **^{13}C NMR (acetone-d$_6$, 75 MHz)** δ: 164.7, 151.3, 149.1, 138.2, 137.9, 134.6, 131.9, 130.2, 128.9, 127.9, 127.0, 122.6, 39.7, 34.1. **EI$^+$** calcd. for $C_{14}H_{13}ClN_2O$ $(M)^+$: 260.0716; Found: 260.0705.

***N*-(2-(Naphthalen-2-yl)ethyl)picolinamide (71).** Compound **71** was prepared following the general protocol from 2-(naphthalen-2-yl)ethanamine (0.77 mL, 5.50 mmol, 1.10 equiv), to give **71** as a brown oil; yield: 1.26 g (83%).

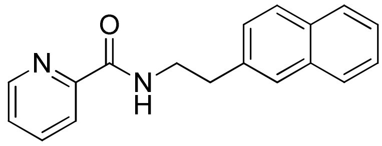

^{1}H NMR (CDCl$_3$, 300 MHz) δ: 8.48 (d, *J* = 4.7 Hz, 1H), 8.23 - 8.11 (m, 2H), 7.87 - 7.75 (m, 2H), 7.71 (s, 1H), 7.48 (s, 2H), 3.90 - 3.77 (m, 1H), 3.12 (t, *J* = 7.1 Hz, 1H). **^{13}C NMR (CDCl$_3$, 75 MHz)** δ: 164.4, 150.0, 148.1, 137.4, 136.5, 133.7, 132.4, 128.3, 127.7, 127.6, 127.3, 127.2, 126.2, 126.1, 125.5, 122.2, 40.7, 36.2. **EI$^+$** calcd. for $C_{18}H_{16}N_2O$ $(M)^+$: 276.1263; Found: 276.1264.

***N*-(2-(Thiophen-2-yl)ethyl)picolinamide (72)**. Compound **72** was prepared following the general protocol from 2-(thiophen-2-yl)ethanamine (0.64 mL, 5.50 mmol, 1.10 equiv), to give **72** as a dark orange oil; yield: 1.14 g (90%).

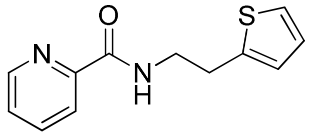

^{1}H NMR (CDCl$_3$, 300 MHz) δ: 8.53 (d, *J* = 3.9 Hz, 1H), 8.26 (s, 1H), 8.21 (d, *J* = 7.8 Hz, 1H), 7.84 (td, *J* = 7.7, 1.7 Hz, 1H), 7.41 (m, 1H), 7.17 (d, *J* = 5.1 Hz, 1H), 6.96 (m, 1H), 6.90 (m, 1H), 3.78 (q, *J* = 6.8 Hz, 2H), 3.18 (t, *J* = 6.9 Hz, 2H). **^{13}C NMR (CDCl$_3$, 75 MHz)** δ: 164.3, 149.9, 148.1, 141.3, 137.3, 127.0, 126.1, 125.3, 123.9, 122.2, 40.9, 30.1. **EI$^+$** calcd. for $C_{12}H_{12}N_2OS$ $(M)^+$: 232.0659; Found: 232.0670.

***N*-(3-Phenylpropyl)picolinamide.** The title compound was prepared following the general protocol from 3-phenylpropan-1-amine (0.78 mL, 5.50 mmol, 1.10 equiv), to give *N*-(3-phenylpropyl)picolinamide as an orange oil; yield: 904 mg (75%).

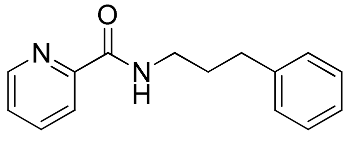

^{1}H NMR (acetone-d$_6$, 300 MHz) δ: 8.58 (d, *J* = 5.0 Hz, 1H), 8.46 (s, 1H), 8.18 (d, *J* = 7.8 Hz, 1H), 7.93 (td, *J* = 7.7, 1.7 Hz, 1H), 7.51 (ddd, *J* = 7.5, 4.8, 1.1 Hz, 1H), 7.31 - 7.11 (m, 5H), 3.51 (dd, *J* = 13.6, 6.7 Hz, 2H), 2.75 - 2.64 (m, 2H), 2.01 - 1.91 (m, 2H). **^{13}C NMR (acetone-d$_6$,**

75 MHz) δ: 164.6, 151.3, 149.0, 142.6, 138.1, 129.1, 129.0, 126.8, 126.5, 122.5, 39.5, 33.8, 32.2. **EI$^+$** calcd. for $C_{15}H_{16}N_2O$ $(M)^+$: 240.1263; Found: 240.1261.

4. Typical procedure for the protection of alkylamine derivatives

Synthesis of *N*-ethylpicolinamide (4). A 50 mL round-bottomed flask immersed in a 0 °C bath (ice and water) was charged with picolinic acid (616 mg, 5.0 mmol, 1.00 equiv) and CH_2Cl_2 (10 mL). To the stirred suspension was added oxalyl chloride (0.47 mL, 5.50 mmol, 1.10 equiv) dropwise over a 15-minute period followed by addition of DMF (0.10 mL, catalytic amount) in one portion, producing a rust-red color and the evolution of a gas. The mixture was kept in the cooling bath for 1 h and then allowed to warm to room temperature. After gas evolution ceased, the mixture was again cooled to 0 °C and NEt_3 (1.40 mL, 10.0 mmol) was added dropwise over a 15-minute period followed by ethylamine solution 2M in THF (2.50 mL, 5.00 mmol, 1.00 equiv) added dropwise over a 15-minute period. The brown mixture was left in the cooling bath for 30 minutes and then allowed to warm to room temperature. Stirring was continued at room temperature for 2 h. Removal of solvent *in vacuo* gave the crude product as a brown solid that was extracted with H_2O-CH_2Cl_2. The organic phases were combined and concentrated under reduced pressure to give **4** as a pale yellow oil; yield: 654 mg (87%). **^{1}H NMR ($CDCl_3$, 300 MHz)** δ: 8.42 (d, J = 4.7 Hz, 1H), 8.09 (d, J = 7.8 Hz, 1H), 7.99 (s, 1H), 7.72 (t, J = 7.7 Hz, 1H), 7.32 - 7.25 (m, 1H), 3.41 (q, J = 7.2 Hz, 2H), 1.15 (t, J = 7.3 Hz, 3H). **^{13}C NMR ($CDCl_3$, 75 MHz)** δ: 164.0, 149.9, 147.8, 137.2, 125.9, 122.0, 34.1, 14.7. **EI$^+$** calcd. for $C_8H_{10}N_2O$ $(M)^+$: 150.0793; Found: 150.0800.

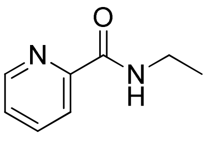

5. Typical procedure for the synthesis of alkynes

5.1. Synthesis of diaryl alkynes

Diphenylacetylene was purchased from Aldrich and used as received.

Synthesis of 1,2-bis(4-methoxyphenyl)ethyne (I).[2] A 50 mL round-bottomed flask was charged with 1-iodo-4-methoxybenzene (468 mg, 2.00 mmol, 1.00 equiv), $PdCl_2$ (3.54 mg, 0.02 mmol), pyrrolidine (0.83 mL, 10.0 mmol) and H_20 (2.50 mL). The mixture was heated to 50 °C for 15 min before the 1-ethynyl-4-methoxybenzene (0.31 mL, 2.40 mmol, 1.20 equiv) was added. The reaction was left stirring for 24h and then allowed to warm to room temperature. The desired product was extracted with H_2O-CH_2Cl_2. The organic phases were combined and concentrated under reduced pressure. The resulting residue was purified by column chromatography (*n*-hexane as only eluent) to give **I** as a white solid; yield: 427 mg (90%); mp= 145-146 °C. **^{1}H NMR (acetone-d_6, 300 MHz)** δ: 7.44 (d, J = 8.9 Hz, 4H), 6.95 (d, J = 8.9 Hz, 4H), 3.83 (s, 6H). **^{13}C NMR (acetone-d_6, 75 MHz)** δ: 160.6, 133.6, 116.4, 115.0, 88.6, 55.6. **EI$^+$** calcd. for $C_{16}H_{14}O_2$ $(M)^+$: 238.0994; Found: 238.0996.

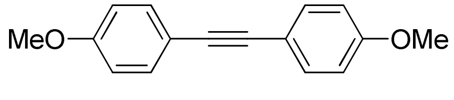

[2] B. Liang, M. Dai, J. Chen and Z. Yang, *J. Org. Chem.*, 2005, **70**, 391.

1,2-Bis(4-(*tert*-butyl)phenyl)ethyne (II). Compound **II** was prepared following the typical

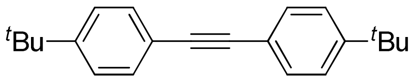

procedure from 1-(*tert*-butyl)-4-iodobenzene (0.35 mg, 2.00 mmol, 1.00 equiv) and 1-(*tert*-butyl)-4-ethynylbenzene (0.43 mL, 2.40 mmol, 1.20 equiv), to give **II** as a yellow solid; yield: 224 mg (39%); mp= 171-172 °C. **^{1}H NMR (acetone-d$_6$, 300 MHz)** δ: 7.46 (s, 8H), 1.33 (s, 18H). **^{13}C NMR (acetone-d$_6$, 75 MHz)** δ: 152.4, 132.0, 126.3, 121.3, 89.5, 35.3, 31.4. **EI$^+$** calcd. for $C_{22}H_{26}$ $(M)^+$: 290.2035; Found: 290.2037.

1,2-Di-*p*-tolylethyne (III). Compound **III** was prepared following the typical procedure from 1-

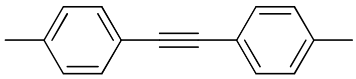

1-iodo-4-methylbenzene (436 mg, 2.00 mmol, 1.00 equiv) and 1-ethynyl-4-methylbenzene (0.30 mL, 2.40 mmol, 1.20 equiv), to give **III** as a white solid; yield: 384 mg (93%); mp= 121-122 °C. **^{1}H NMR (acetone-d$_6$, 300 MHz)** δ: 7.41 (d, *J* = 8.1 Hz, 4H), 7.22 (d, *J* = 7.9 Hz, 4H), 2.35 (s,6H). **^{13}C NMR (acetone-d$_6$, 75 MHz)** δ: 139.3, 132.1, 130.1, 121.2, 89.5, 21.4. **EI$^+$** calcd. for $C_{16}H_{14}$ $(M)^+$: 206.1096; Found: 206.1099.

1,2-Bis(4-(trifluoromethyl)phenyl)ethyne (IV). Compound **IV** was prepared following the

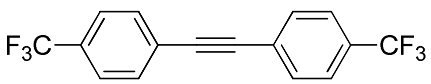

typical procedure from 1-iodo-4-(trifluoromethyl)benzene (0.30 mL, 2.00 mmol, 1.00 equiv) and 1-ethynyl-4-(trifluoromethyl)benzene (0.40 mL, 2.40 mmol, 1.20 equiv), to give **IV** as a white solid; yield: 710 mg (95%); mp= 107-108 °C. **^{1}H NMR (CDCl$_3$, 300 MHz)** δ: 7.67 - 7.61 (m, 8H). **^{13}C NMR (CDCl$_3$, 125 MHz)** δ: 132.1, 130.6 (q, *J* = 32.8 Hz), 126.5 (q, *J* = 1.2 Hz), 125.5 (q, *J* = 3.8 Hz), 124.0 (q, *J* = 272.2 Hz), 90.2. **EI$^+$** calcd. for $C_{16}H_8F_6$ $(M)^+$: 314.0530; Found: 314.0529.

1,2-Bis(3-methoxyphenyl)ethyne (V). Compound **V** was prepared following the typical

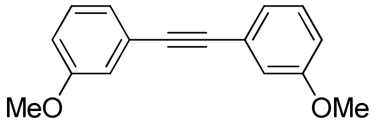

procedure from 1-iodo-3-methoxybenzene (0.23 mL, 2.00 mmol, 1.00 equiv) and 1-ethynyl-3-methoxybenzene (0.30 mL, 2.40 mmol, 1.20 equiv), to give **V** as a yellow solid; yield: 770 mg (81%); mp= 62-63 °C. **^{1}H NMR (acetone-d$_6$, 300 MHz)** δ: 7.32 (t, *J* = 7.9 Hz, 2H), 7.17 - 7.07 (m, 4H), 7.01 - 6.94 (m, 2H), 3.83 (s, 6H). **^{13}C NMR (acetone-d$_6$, 75 MHz)** δ: 160.6, 130.5, 125.0, 124.7, 117.1, 115.8, 89.7, 55.6. **EI$^+$** calcd. for $C_{16}H_{14}O_2$ $(M)^+$: 238.0994; Found: 238.0983.

1,2-Di-*o*-tolylethyne (VI). Compound **VI** was prepared following the typical procedure from 1-

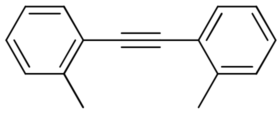

iodo-2-methylbenzene (0.25 mL, 2.00 mmol, 1.00 equiv) and 1-ethynyl-2-methylbenzene (0.30 mL, 2.40 mmol, 1.20 equiv), to give **VI** as a yellow oil; yield: 769 mg (93%). **^{1}H NMR (acetone-d$_6$, 300 MHz)** δ: 7.53 (d, *J* = 7.4 Hz, 2H), 7.34 - 7.20 (m, 6H), 2.52 (s, 6H). **^{13}C NMR (acetone-d$_6$, 75 MHz)** δ: 140.4, 132.5, 130.3, 129.1, 126.5, 124.0, 93.1, 21.1. **EI$^+$** calcd. for $C_{16}H_{14}$ $(M)^+$: 206.1096; Found: 206.1091.

5.2. Synthesis of alkyl-aryl alkynes

Synthesis of 1-(cyclohexylethynyl)-4-methoxybenzene (VII).[3] A 50 mL round-bottomed flask was charged with 1-iodo-4-methoxybenzene (1.17 g, 5.00 mmol, 1.00 equiv), $Pd(PPh_3)_2Cl_2$ (175.5 g, 0.25 mmol) and copper(I) iodide (95.2 mg, 0.5 mmol). The mixture was vacuumed and flushed with Argon for three times. Then Et_3N (10 mL) and the ethynylcyclohexane (0.78 mL, 6.00 mmol, 1.20 equiv) was added. The reaction was left stirring at room temperature until the aryl iodide was consumed. The resulting mixture was diluted with diethyl ether, washed with water and brine, dried with anhydrous $MgSO_4$, concentrated under reduced pressure and purified by column chromatography (*n*-hexane as only eluent) to give **VII** as a yellow oil; yield: 536 mg (50%). **^{1}H NMR ($CDCl_3$, 300 MHz)** δ: 7.33 (d, *J* = 8.8 Hz, 1H), 6.80 (d, *J* = 8.8 Hz, 1H), 3.79 (s, 2H), 2.62 - 2.50 (m, 1H), 1.94 - 1.83 (m, 1H), 1.83 - 1.68 (m, 1H), 1.56 - 1.45 (m, 2H), 1.40 - 1.24 (m, 2H). **^{13}C NMR ($CDCl_3$, 75 MHz)** δ: 159.7, 133.3, 114.7, 113.9, 88.0, 85.9, 81.5, 55.3, 35.2, 26.9, 25.4, 22.8. **EI$^+$** calcd. for $C_{15}H_{18}O$ $(M)^+$: 214.1358; Found: 214.1360.

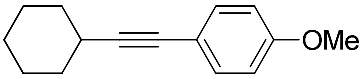

1-(Cyclohexylethynyl)-4-(trifluoromethyl)benzene (VIII). Compound **VIII** was prepared following the typical procedure from 1-bromo-4-(trifluoromethyl)benzene (0.70 mL, 5.00 mmol, 1.00 equiv), to give **VIII** as a yellow oil; yield: 755 mg (60%). **^{1}H NMR ($CDCl_3$, 300 MHz)** δ: 7.50 (q, *J* = 8.5 Hz, 2H), 2.67 - 2.55 (m, 1H), 1.93 - 1.83 (m, 1H), 1.76 (dd, *J* = 8.9, 3.9 Hz, 1H), 1.59 - 1.25 (m, 4H). **^{13}C NMR ($CDCl_3$, 126 MHz)** δ: 132.2, 131.9, 129.3 (q, *J* = 32.6 Hz), 125.1 (q, *J* = 3.8 Hz), 124.1 (q, *J* = 272.0 Hz), 97.3, 79.6, 35.1, 32.6, 29.8, 26.0, 25.0, 22.8. **EI$^+$** calcd. for $C_{15}H_{15}F_3$ $(M)^+$: 252.1126; Found: 252.1126.

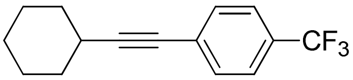

2-(Cyclohexylethynyl)thiophene (IX). Compound **IX** was prepared following the typical procedure from 2-iodothiophene (0.70 mL, 5.00 mmol, 1.00 equiv), to give **IX** as a brown oil; yield: 542 mg (57%). **^{1}H NMR ($CDCl_3$, 300 MHz)** δ: 7.16 (d, *J* = 5.1 Hz, 1H), 7.11 (d, *J* = 3.3 Hz, 1H), 6.97 - 6.89 (m, 1H), 2.67 - 2.54 (m, 1H), 1.95 - 1.80 (m, 2H), 1.80 - 1.67 (m, 2H), 1.62 - 1.48 (m, 3H), 1.40 - 1.26 (m, 3H). **^{13}C NMR ($CDCl_3$, 75 MHz)** δ: 130.9, 126.8, 125.9, 124.4, 98.5, 73.7, 32.6, 30.0, 26.0, 25.0. **EI$^+$** calcd. for $C_{12}H_{14}S$ $(M)^+$: 190.0816; Found: 190.0819.

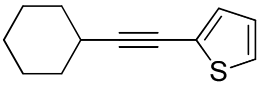

6. Typical procedure for the synthesis of 1,3-enynes

Synthesis of (*E*)-methyl 6-cyclohexylhex-2-en-4-ynoate (X).

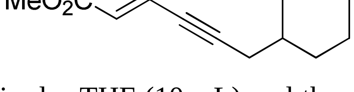

Synthesis of 3-cyclohexylpropiolaldehyde. Following a modified procedure by Larsen *et al.*,[4] prop-2-yn-1-ylcyclohexane (1.16 mL, 8.00 mmol, 1.00 equiv) was dissolved in dry THF (10 mL) and the solution was cooled to -40 °C. A solution of *n*BuLi in hexane 2M (3.20 mL, 8.00 mmol, 1.10 equiv) was added dropwise maintaining the temperature under ¬35 °C. After addition, anhydrous DMF (1.22 mL, 16.0 mmol, 2.00 equiv) was added in one portion and the cold bath was removed. The reaction mixture was allowed to warm to room temperature for 30 min. The THF solution was poured in a vigorously stirred biphasic solution

---

[3] X. Zhang, S. Sarkar and R. C. Larock, *J. Am. Chem. Soc.*, 2010, **132**, 14070.
[4] M. Journet, D. Cai, L. M. DiMichele and R. D. Larsen, *Tetrahedron Lett.*, 1998, **39**, 6427.

prepared from 10% aqueous KH_2PO_4 (43.2 mL) and MTBE(40 mL) cooled over ice. Layers were separated and the organic extract was washed with water. Combined organic layers were dried over Na_2SO_4, filtered and concentrated obtaining an oil which was filtered through silica gel using a mixture of *n*-hexane/AcOEt (9:1) as eluent to give the corresponding aldehyde (3-cyclohexylpropiolaldehyde) as a colorless oil. This product was directly used in the next step in order to obtain the desired product through a Horner-Wadsworth-Emmons reaction.[5]

Thus, a 50 mL round-bottomed flask immersed in a -78 °C bath (CO_2(s) and acetone) was charged with methyl 2-(dimethoxyphosphoryl)acetate (1.18 mL, 7.27 mmol, 1.00 equiv) and THF (15 mL). To the stirred solution *n*BuLi in hexane 2M (3.20 mL, 8.0 mmol, 1.10 equiv) was added dropwise. The reaction mixture was stirred at 0 °C bath (ice and water) for 30 min. After that, the reaction was cooled again at -78 °C for the addition of the obtained α,β-acetylenic aldehyde (1.16 mL, 8.00 mmol, 1.10 equiv) and the mixture was allowed to warm to room temperature for 15 min. The reaction was then quenched with 10 mL of water and the aqueous layer was extracted with AcOEt. The organic layers were combined, dried over Na_2SO_4 and concentrated under reduced pressure to give a dark oil. The obtained oil was purified by chromathography using *n*-hexane as eluent to give **68** as a yellow oil; yield: 1.19 g (72%). **^{1}H NMR ($CDCl_3$, 300 MHz)** δ: 6.77 (d, *J* = 15.8 Hz, 1H), 6.15 (d, *J* = 15.8 Hz, 1H), 3.74 (s, 3H), 2.26 (d, *J* = 6.6 Hz, 2H), 1.84 - 1.60 (m, 5H), 1.59 - 1.44 (m, 1H), 1.30 - 1.12 (m, 3H), 1.07 - 0.92 (m, 2H). **^{13}C NMR ($CDCl_3$, 75 MHz)** δ: 166.7, 128.8, 126.6, 100.2, 78.9, 51.8, 37.3, 32.8, 27.7, 26.3, 26.2. **EI$^+$** calcd. for $C_{13}H_{18}O_2$ $(M)^+$: 206.1307; Found: 206.1310.

(*E*)-Methyl 7-phenylhept-2-en-4-ynoate (XI). Compound **XI** was prepared following the general protocol from but-3-yn-1-ylbenzene (1.12 mL, 8.00 mmol, 2.00 equiv), to give **XI** as a orange oil; yield: 1.09 g (64%). **^{1}H NMR ($CDCl_3$, 300 MHz)** δ: 7.35 - 7.18 (m, 5H), 6.74 (d, *J* = 15.8 Hz, 1H), 6.14 (d, *J* = 15.8 Hz, 1H), 3.75 (s, 3H), 2.87 (t, *J* = 7.4 Hz, 2H), 2.67 (t, *J* = 7.4 Hz, 2H). **^{13}C NMR ($CDCl_3$, 755 MHz)** δ: 166.6, 140.3, 129.2, 128.5, 128.5, 126.6, 126.2, 99.9, 78.7, 51.8, 34.7, 22.0. **EI$^+$** calcd. for $C_{14}H_{14}O_2$ $(M)^+$: 214.0994; Found: 214.0961.

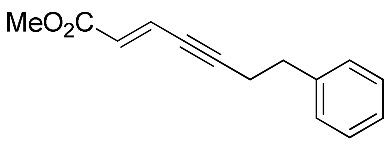

(*E*)-Methyl 9-chloronon-2-en-4-ynoate (XII). Compound **XII** was prepared following the general protocol from 6-chlorohex-1-yne (0.96 mL, 8.00 mmol, 2.00 equiv), to give **XII** as a orange oil; yield: 0.80 g (50%). **^{1}H NMR ($CDCl_3$, 300 MHz)** δ: 6.75 (d, *J* = 15.8 Hz, 1H), 6.16 (d, *J* = 15.9 Hz, 1H), 3.75 (s, 3H), 3.57 (t, *J* = 6.4 Hz, 2H), 2.44 (t, *J* = 6.8 Hz, 2H), 1.99 - 1.85 (m, 2H), 1.81 - 1.67 (m, 2H). **^{13}C NMR ($CDCl_3$, 75 MHz)** δ: 166.6, 129.3, 126.2, 99.8, 78.5, 51.9, 44.4, 31.6, 25.6, 19.2. **EI$^+$** calcd. for $C_{10}H_{13}ClO_2$ $(M)^+$: 200.0604; Found: 200.0601.

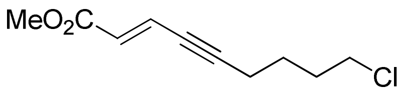

[5] (*a*) L. Horner, H. Hoffmann and H. G. Wippel, *Chem. Ber.* 1958, **91**, 61; (*b*) L. Horner, H. Hoffmann, H. G. Wippel and G. Klahre, *Chem Ber.* 1959, **92**, 2499; (*c*) W. S. Wadsworth and W. D. Emmons, *J. Am. Chem. Soc.* 1961, **83**, 1733.

7. Rh(III)-catalyzed heteroaryl C–H functionalization (Scheme 1)

7.1. Scope with regard to the heteroaryl moiety

Synthesis of *N*-benzyl-5,6,7,8-tetraphenylisoquinoline-1-carboxamide (2).

Method A: Thermal conditions. An oven-dried, nitrogen-flushed 20 mL vessel was charged

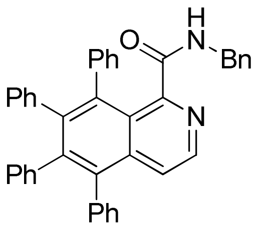

with *N*-benzylpicolinamide (**1**) (31.8 mg, 0.15 mmol, 1.00 equiv), diphenylacetylene (53.3 mg, 0.30 mmol, 2.00 equiv), pentamethylcyclopentadienylrhodium(III) chloride dimer (4.64 mg, 0.0075 mmol, 0.050 equiv), copper (II) acetate (54.5 mg, 0.30 mmol, 2.00 equiv) and silver hexafluoroantimonate(V) (10.7 mg, 0.03 mmol, 0.20 equiv). The reaction vessel was sealed with a Teflon lined cap, then evacuated and flushed with nitrogen three times. Under the atmosphere of nitrogen, 1,4-dioxane (1.00 mL) were added *via* syringe. The resulting mixture was then stirred at 120 °C for 24 h. After the reaction was complete, the volatiles were removed *in vacuo* and the residue was purified by column chromatography (*n*-hexane-EtOAc 2:1), yielding **2** as a pale yellow solid; yield: 80.0 mg (94%); mp= 261-262 °C. **^{1}H NMR (methanol-d$_4$, 500 MHz)** δ: 8.36 (d, *J* = 5.9 Hz, 1H), 7.52 (d, *J* = 5.9 Hz, 1H), 7.33 - 7.13 (m, 16H), 6.90 - 6.77 (m, 11H), 3.78 (s, 2H). **^{13}C NMR (methanol-d$_4$, 125 MHz)** δ: 170.9, 157.2, 145.2, 144.2, 141.6, 140.9, 140.8, 139.7, 139.4, 139.4, 139.3, 139.0, 137.9, 133.9, 132.2, 132.2, 131.8, 129.4, 128.9, 128.8, 128.2, 128.2, 128.1, 127.8, 127.7, 127.6, 126.9, 126.7, 124.9, 122.0, 44.4. **FB$^+$** calcd. for $C_{41}H_{31}N_2O$ $(M+H)^+$: 567.2436; Found: 567.2439. The structure of this compound was confirmed by X-ray diffraction.

Method B: Microwave assisted conditions. An oven-dried, nitrogen-flushed 10 mL microwave vessel was charged with *N*-benzylpicolinamide (**1**) (31.8 mg, 0.15 mmol, 1.00 equiv), diphenylacetylene (53.3 mg, 0.30 mmol, 2.00 equiv), pentamethylcyclopentadienylrhodium(III) chloride dimer (4.64 mg, 0.0075 mmol, 0.05 equiv), copper (II) acetate (54.5 mg, 0.30 mmol, 2.00 equiv) and silver hexafluoroantimonate(V) (10.7 mg, 0.03 mmol, 0.20 equiv). The reaction vessel was sealed with a Teflon lined cap, then evacuated and flushed with nitrogen three times. Under the atmosphere of nitrogen, 1,4-dioxane (1.00 mL) was added *via* syringe. The resulting solution was then stirred for 5 min at room temperature followed by microwave irradiation at 120 °C for 1 h. Removal of solvent *in vacuo* gave the crude product as a brown solid that was extracted with H_2O-CH_2Cl_2. The organic phases were combined and concentrated under reduced pressure. The residue was purified by column chromatography (*n*-hexane-EtOAc 2:1), yielding **2** as a pale yellow solid; yield: 76.4 mg (90%). The structure of this compound was confirmed by X-ray diffraction.

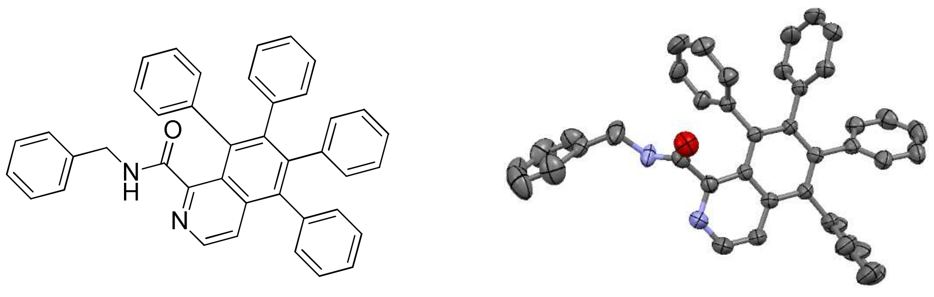

ORTEP view of **2**, hydrogen atoms have been removed for simplicity

***N*-Benzyl-3-methyl-5,6,7,8-tetraphenylisoquinoline-1-carboxamide (14).** In this case, compound **14** was prepared following the general protocol A from *N*-benzyl-6-methylpicolinamide (**5**) (33.9 mg, 0.15 mmol, 1.00 equiv) by conventional heating at 120 °C for 24h to give **14** as a yellow oil; yield: 46.9 mg (54%). **^{1}H NMR ($CDCl_3$, 300 MHz)** δ: 7.48 - 7.33 (m, 14H), 7.05 - 6.92 (m, 7H), 6.92 - 6.80 (m, 5H), 6.58 (s, 1H), 4.08 (d, *J* = 4.9 Hz, 2H), 2.68 (s, 3H). **^{13}C NMR ($CDCl_3$, 126 MHz)** δ: 168.2, 155.0, 149.6, 143.4, 141.2, 139.9, 139.6, 138.4, 138.0, 137.7, 137.6, 137.2, 132.1, 131.3, 130.7, 128.7, 128.3, 127.8, 127.6, 127.0, 126.8, 126.8, 126.7, 126.7, 125.8, 125.6, 122.7, 119.1, 44.2, 23.9. **ESI$^+$** calcd. for $C_{42}H_{33}N_2O$ $(M+H)^+$: 581.2587; Found: 581.2566.

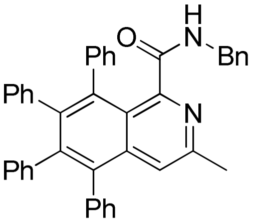

***N*-Benzyl-3-chloro-5,6,7,8-tetraphenylisoquinoline-1-carboxamide (15).** Compound **15** was prepared following the general protocol B from *N*-benzyl-6-chloropicolinamide (**6**) (36.9 mg, 0.15 mmol, 1.00 equiv), to give **15** as a pale yellow oil; yield: 55.9 mg (62%). **^{1}H NMR ($CDCl_3$, 300 MHz)** δ: 7.48 (s, 1H), 7.41 - 7.17 (m, 8H), 7.17 - 7.10 (m, 7H), 6.87 (dd, *J* = 4.2, 2.5 Hz, 6H), 6.76 - 6.68 (m, 4H), 6.37 (t, *J* = 5.0 Hz, 1H), 3.94 (d, *J* = 5.2 Hz, 2H). **^{13}C NMR ($CDCl_3$, 75 MHz)** δ: 166.6, 156.1, 144.6, 143.4, 142.6, 139.3, 139.3, 139.1, 139.0, 138.4, 137.5, 137.3, 137.2, 132.1, 131.1, 131.1, 130.5, 128.8, 128.4, 128.1, 127.7, 127.4, 127.0, 126.9, 126.9, 126.8, 126.1, 125.9, 123.6, 120.4, 44.2. **ESI$^+$** calcd. for $C_{41}H_{30}ClN_2O$ $(M+H)^+$: 601.2041; Found: 601.2051.

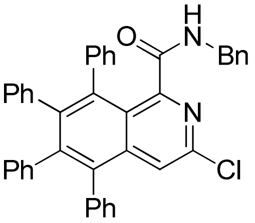

7-Benzyl-5,6-diphenyl-3-(trifluoromethyl)-1,7-naphthyridin-8(7H)-one (16). Compound **16** was prepared following the general protocol B from *N*-benzyl-5-(trifluoromethyl)picolinamide (**7**) (42.0 mg, 0.15 mmol, 1.00 equiv), to give **16** as a yellow solid; yield: 54.7 mg (84%); mp= 213-215 °C. **^{1}H NMR ($CDCl_3$, 300 MHz)** δ: 9.10 (s, 1H), 7.78 (s, 1H), 7.21 - 7.08 (ddd, *J* = 20.6, 14.1, 6.4 Hz, 9H), 7.04 - 6.98 (m, 2H), 6.88 (d, *J* = 7.1 Hz, 4H), 5.29 (s, 2H). **^{13}C NMR ($CDCl_3$, 126 MHz)** δ: 161.0, 145.5 (q, *J* = 3.5 Hz), 144.4, 142.9, 137.0, 134.3, 133.4, 133.0, 131.5 (q, *J* = 3.9 Hz), 131.3, 130.2, 129.3, 128.8, 128.6, 128.4, 128.0, 127.8, 127.5, 127.4, 123.1 (q, *J* = 273.4 Hz), 117.3, 49.8. **^{19}F NMR ($CDCl_3$, 282 MHz)** δ: -62.4. **ESI$^+$** calcd. for $C_{42}H_{30}F_3N_2O$ $(M)^+$: 456.1449; Found: 457.1492. The structure of this compound was confirmed by X-ray diffraction.

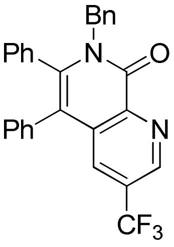

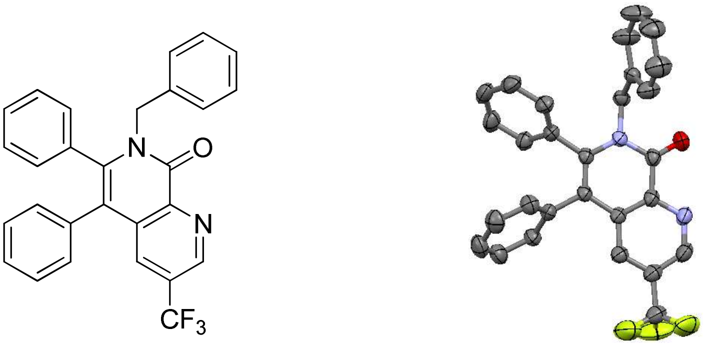

ORTEP view of **14**, hydrogen atoms have been removed for simplicity

***N*-Benzyl-7,8,9,10-tetraphenylphenanthridine-6-carboxamide (17).** Compound **17** was

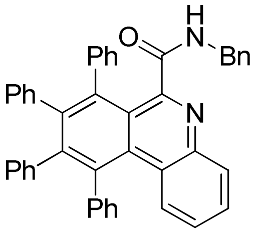

prepared following the general protocol B from *N*-benzylquinoline-2-carboxamide (**8**) (39.3 mg, 0.15 mmol, 1.00 equiv), to give **17** as a yellow solid; yield: 42.5 mg (46%); mp= 250-252 °C. **^{1}H NMR ($CDCl_3$, 300 MHz)** δ: 8.00 (d, *J* = 8.0 Hz, 1H), 7.51 (t, *J* = 7.0 Hz, 1H), 7.40 - 7.04 (s, 17H), 6.93 - 6.82 (d, *J* = 2.8 Hz, 6H), 6.75 - 6.63 (s, 4H), 6.60 (t, *J* = 4.5 Hz, 1H), 4.01 (d, *J* = 5.1 Hz, 2H). **^{13}C NMR ($CDCl_3$, 75 MHz)** δ: 165.6, 151.3, 145.8, 141.1, 139.7, 139.0, 138.4, 137.5, 137.2, 130.8, 130.1, 129.7, 129.6, 129.4, 128.7, 128.6, 128.2, 128.1, 128.0, 127.8, 127.7, 127.4, 127.3, 127.0, 43.6. **ESI$^+$** calcd. for $C_{45}H_{33}N_2O$ $(M)^+$: 617.2587; Found: 617.2580.

6-Benzyl-2-methyl-4,5-diphenylthieno[2,3-*c*]pyridin-7(6H)-one (18). Compound **18** was

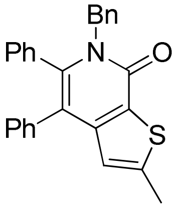

prepared following the general protocol A from *N*-benzyl-5-methylthiophene-2-carboxamide (**9**) (34.7 mg, 0.15 mmol, 1.00 equiv). to give **18** as a yellow solid; yield: 60.2 mg (99%); mp= 174-176 °C. **^{1}H NMR ($CDCl_3$, 300 MHz)** δ: 7.24 - 7.00 (m, 11H), 6.82 - 6.97(s, 4H), 6.57 (s, 1H), 5.24 (s, 2H), 2.54 (s, 3H). **^{13}C NMR ($CDCl_3$, 125 MHz)** δ: 158.4, 148.8, 146.6, 142.6, 137.9, 137.1, 134.1, 130.8, 130.7, 128.4, 128.3, 127.9, 127.7, 127.2, 127.0, 126.8, 123.1, 118.1, 48.9, 16.4. **ESI$^+$** calcd. for $C_{27}H_{22}NOS$ $(M+H)^+$: 408.1416; Found: 408.1405.

Compound **18** was also prepared following method B to give the title compound in 99% yield (60.2 mg). The structure of this compound was confirmed by X-ray diffraction.

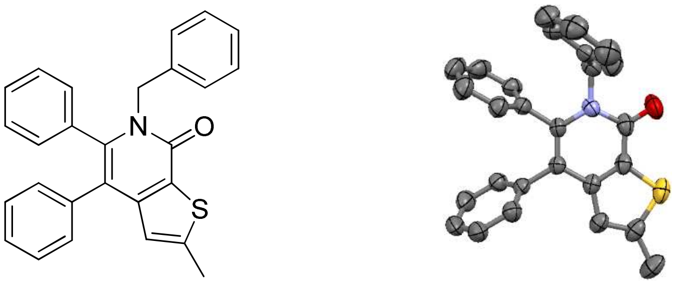

ORTEP view of **16**, hydrogen atoms have been removed for simplicity

2-Benzyl-3,4-diphenylbenzo[4,5]thieno[2,3-*c*]pyridin-1(2H)-one (19). Compound **19** was

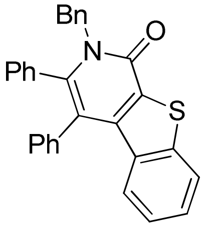

prepared following the general protocol A from *N*-benzylbenzo[*b*]thiophene-2-carboxamide (**10**) (40.1 mg, 0.15 mmol, 1.00 equiv), to give **19** as a yellow solid; yield: 60.3 mg (91%); mp= 226-227 °C. **^{1}H NMR ($CDCl_3$, 300 MHz)** δ: 7.92 (d, *J* = 8.1 Hz, 1H), 7.39 (t, *J* = 7.6 Hz, 1H), 7.26 (s, 11H), 6.91 (d, *J* = 6.1 Hz, 4H), 6.58 (d, *J* = 8.3 Hz, 1H), 5.29 (s, 2H). **^{13}C NMR ($CDCl_3$, 75 MHz)** δ: 159.1, 143.6, 142.9, 140.0, 137.4, 136.6, 135.8, 133.7, 131.2, 130.6, 129.8, 128.4, 128.3, 128.3, 127.7, 127.5, 127.3, 127.2, 127.1, 125.9, 124.3, 123.3, 118.9, 49.3. **ESI$^+$** calcd. for $C_{30}H_{22}NOS$ $(M+H)^+$: 444.1416; Found: 444.1398.

Compound **17** was also prepared following method B to give the title compound in 98% yield (65.5 mg).

7.2. Scope with regard to the *N*-substituent

7-Phenethyl-5,6-diphenyl-1,7-naphthyridin-8(7H)-one (88) (Scheme 5). Compound **88** was

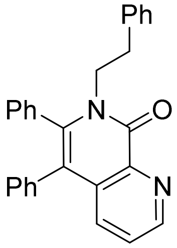

prepared following the general protocol B from with *N*-phenethylpicolinamide (**61**) (61.8 mg, 0.15 mmol, 2.00 equiv) to give **88** as a pale yellow solid; yield: 54.3 mg (90%); mp= 229-230 °C. **^{1}H NMR ($CDCl_3$, 300 MHz)** δ: 8.91 (d, *J* = 2.2 Hz, 1H), 7.53 (dd, *J* = 8.3, 2.2 Hz, 1H), 7.45 (dd, *J* = 8.3, 1H), 7.19 (m, 9H), 7.04 (m, 4H), 6.92 - 6.83 (m, 2H), 4.16 - 4.09 (m, 2H), 3.03 - 2.88 (m, 2H). **^{13}C NMR ($CDCl_3$, 75 MHz)** δ: 161.2, 149.6, 142.4, 141.1, 138.5, 135.4, 134.2, 133.9, 133.5, 131.5, 130.2, 129.0, 128.6, 128.5, 128.2, 128.2, 127.3, 126.5, 117.3, 48.5, 34.6. **FB$^+$** calcd. for $C_{28}H_{23}N_2O$ $(M+H)^+$: 403.1810; Found: 403.1818.

***N*-Ethyl-5,6,7,8-tetraphenylisoquinoline-1-carboxamide (13) (Scheme 1).** Compound **13** was

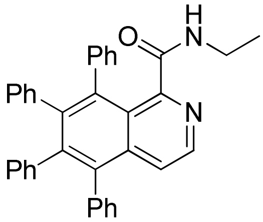

prepared following the general protocol B from *N*-ethylpicolinamide (**4**) (22.5 mg, 0.15 mmol, 2.00 equiv) to give **13** as a yellow oil; yield: 64.2 mg (85%). **^{1}H NMR ($CDCl_3$, 300 MHz)** δ: 9.11 (d, *J* = 5.7 Hz, 1H), 8.24 (d, *J* = 5.8 Hz, 1H), 8.03 (dd, *J* = 12.1, 4.9 Hz, 3H), 7.97 - 7.86 (m, 7H), 7.65 (s, 6H), 7.56 - 7.45 (m, 4H), 6.93 (s, 1H), 3.71 - 3.55 (m, 2H), 1.80 (t, *J* = 7.3 Hz, 3H). **^{13}C NMR ($CDCl_3$, 75 MHz)** δ: 168.2, 168.2, 156.0, 143.6, 142.2, 140.5, 139.7, 139.5, 139.3, 138.3, 138.2, 137.8, 137.0, 132.2, 131.2, 131.2, 130.7, 127.9, 127.1, 126.8, 126.8, 126.7, 125.9, 125.7, 121.1, 34.7, 14.2. **FB$^+$** calcd. for $C_{36}H_{29}N_2O$ $(M+H)^+$: 505.2280; Found: 505.2277.

7.3. Scope with regard to the alkyne

Synthesis of *N*-benzyl-5,6,7,8-tetra-*p*-tolylisoquinoline-1-carboxamide (11). Compound **11**

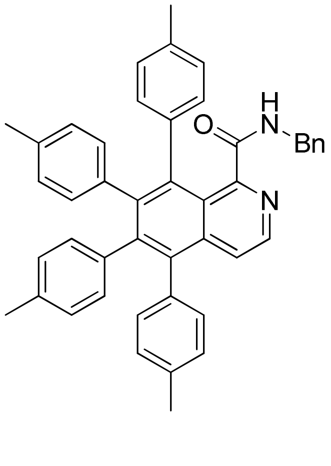

was prepared following the general protocol B from 1,2-di-*p*-tolylethyne (**III**) (61.8 mg, 0.30 mmol, 2.00 equiv) to give **11** as a pale yellow oil; yield: 57.0 mg (61%). **^{1}H NMR ($CDCl_3$, 300 MHz)** δ: 8.26 (s, 1H), 7.36 - 7.22 (m, 6H), 7.10 - 6.90 (m, 9H), 6.73 - 6.54 (m, 9H), 6.34 (s, 1H), 3.98 (s, 2H), 2.31 (s, 6H), 2.13 (s, 3H), 2.10 (s, 3H). **^{13}C NMR ($CDCl_3$, 75 MHz)** δ: 167.1, 154.8, 144.7, 144.7, 143.0, 138.5, 138.5, 137.9, 137.6, 137.4, 136.7, 136.6, 136.4, 136.2, 135.2, 135.1, 135.0, 132.2, 131.1, 130.9, 130.5, 128.7, 128.6, 128.4, 127.7, 127.6, 127.6, 127.5, 124.7, 121.7, 44.3, 29.8, 21.4, 21.3, 21.2. **ESI$^+$** calcd. for $C_{45}H_{39}N_2O$ $(M+H)^+$: 623.3056; Found: 623.3054.

***N*-Benzyl-5,6,7,8-tetrakis(4-(trifluoromethyl)phenyl)isoquinoline-1-carboxamide (12).**

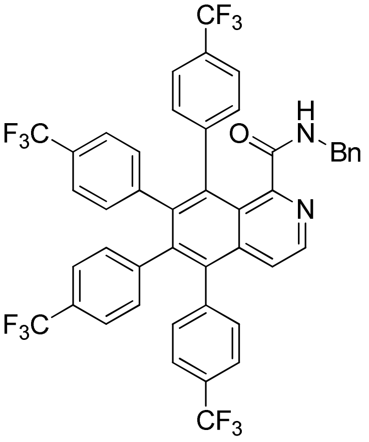

Compound **12** was prepared following the general protocol B from 1,2-bis(4-(trifluoromethyl)yphenyl)ethyne (**IV**) (94.2 mg, 0.30 mmol, 2.00 equiv), to give **12** as a pale yellow oil; yield: 79.3 mg (63%). **^{1}H NMR (CDCl$_3$, 500 MHz)** δ: 8.41 (s, 1H), 7.57 (d, *J* = 8.0 Hz, 2H), 7.43 (t, *J* = 8.4 Hz, 2H), 7.26 (s, 13H), 6.86 (dd, *J* = 14.0, 8.0 Hz, 4H), 6.65 (s, 1H), 3.96 (s, 2H). **^{13}C NMR (CDCl$_3$, 125 MHz)** δ: 170.3, 155.0, 143.6, 142.2, 140.4, 139.6, 139.6, 139.4, 138.1, 138.1, 137.8, 137.0, 132.3, 131.2, 131.2, 130.7, 127.9, 127.1, 127.0, 126.9, 126.9, 126.8, 126.0, 125.8, 121.4, 29.8. **ESI$^+$** calcd. for $C_{45}H_{27}F_{12}N_2O$ $(M+H)^+$: 839.1926; Found: 839.1944.

8. Rh(I)-catalyzed *ortho*-olefination of the benzylamine derivatives (Scheme 2)

8.1. Scope with regard to the benzylamine

Synthesis of *N*-(2,6-bis((*E*)-1,2-diphenylvinyl)benzyl)picolinamide (3). An oven-dried,

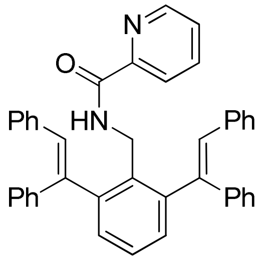

nitrogen-flushed 20 mL vessel was charged with *N*-benzylpicolinamide (**1**) (31.8 mg, 0.15 mmol, 1.00 equiv), diphenylacetylene (53.3 mg, 0.30 mmol, 2.00 equiv), chloro(1,5-cyclooctadiene)rhodium dimer (1.85 mg, 0.00375 mmol, 0.025 equiv), sodium acetate (49.8 mg, 0.60 mmol, 4.00 equiv), and silver hexafluoroantimonate(V) (2.58 mg, 0.0075 mmol, 0.05 equiv). The reaction vessel was sealed with a Teflon lined cap, then evacuated and flushed with nitrogen three times. Under the atmosphere of nitrogen, 1,2-dichloroethane (1.00 mL) were added *via* syringe. The resulting mixture was then stirred at 120 °C for 24 h. After the reaction was complete, the volatiles were removed *in vacuo* and the residue was purified by column chromatography (*n*-hexane-EtOAc 5:1), yielding **3** as a pale yellow solid; yield: 64.9 mg (88%); mp= 186-188 °C. **^{1}H NMR (acetone-d$_6$, 500 MHz)** δ: 8.57 (d, *J* = 4.7 Hz, 1H), 8.06 (s, 1H), 7.99 (d, *J* = 7.7 Hz, 1H), 7.93 (td, *J* = 7.7, 1.5 Hz, 1H), 7.59 - 7.50 (m, 1H), 7.46 - 7.40 (m, 1H), 7.39 (s, 1H), 7.37 (d, *J* = 1.3 Hz, 1H), 7.25 - 7.22 (m, 4H), 7.22 - 7.11 (m, 13H), 7.11 - 7.08 (m, 3H), 6.71 (s, 2H), 4.54 (d, *J* = 5.4 Hz, 2H). **^{13}C NMR (acetone-d$_6$, 125 MHz)** δ: 163.3, 151.1, 149.0, 146.6, 142.8, 140.9, 138.2, 138.0, 134.8, 132.0, 131.0, 130.5, 130.2, 129.1, 128.7, 128.3, 128.2, 127.7, 127.0, 122.4, 39.6. **FB$^+$** calcd. for $C_{41}H_{33}N_2O$ $(M+H)^+$: 569.2593; Found: 569.2596. The structure of this compound was confirmed by X-ray diffraction.

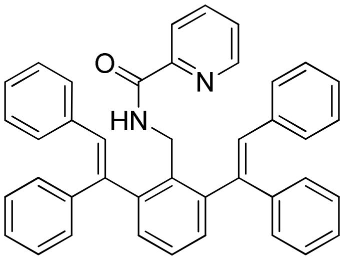

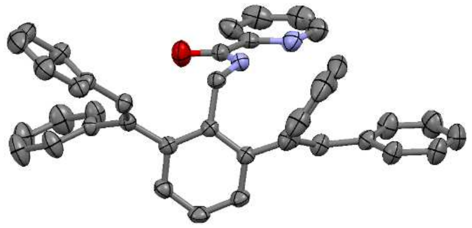

ORTEP view of **3**, hydrogen atoms have been removed for simplicity

***N*-(2,6-Bis((*E*)-1,2-diphenylvinyl)-4-(methylthio)benzyl)picolinamide (39).** Compound **39** was prepared following the general protocol from *N*-(4-(methylthio)benzyl)picolinamide (**20**) (38.7 mg, 0.15 mmol, 1.00 equiv),to give **39** as a yellow solid; yield: 78.0 mg (84%); mp= 83-84 °C. **^{1}H NMR (CDCl$_3$, 300 MHz)** δ: 8.42 (d, *J* = 4.3 Hz, 1H), 8.00 (d, *J* = 7.8 Hz, 1H), 7.79 - 7.65 (m, 2H), 7.35 (dd, *J* = 6.5, 4.9 Hz, 1H), 7.25 - 6.97 (m, 22H), 6.67 (s, 2H), 4.36 (d, *J* = 5.0 Hz, 2H), 2.52 (s, 3H). **^{13}C NMR (CDCl$_3$, 75 MHz)** δ: 162.9, 150.0, 147.7, 146.4, 141.5, 139.4, 137.9, 137.0, 131.4, 130.4, 129.8, 129.4, 128.4, 128.0, 127.7, 127.4, 127.0, 125.7, 121.9, 39.1, 15.6. **ESI$^+$** calcd. for $C_{42}H_{35}N_2OS$ (M+H)$^+$: 615,2464; Found: 615.2472.

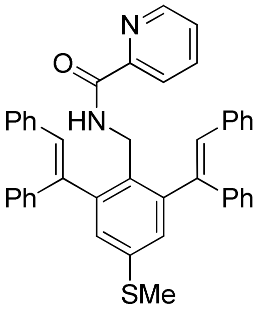

***N*-(2,6-Bis((*E*)-1,2-diphenylvinyl)-4-methoxybenzyl)picolinamide (40).** Compound **40** was prepared following the general protocol from *N*-(4-methoxybenzyl)picolinamide (**21**) (31.8 mg, 0.15 mmol, 1.00 equiv), to give **40** as a white solid; yield: 70.0 mg (78%); mp= 143-144 °C. **^{1}H NMR (acetone-d$_6$, 300 MHz)** δ: 8.57 (d, *J* = 4.6 Hz, 1H), 8.01 - 7.84 (m, 3H), 7.52 (dd, *J* = 6.7, 5.4 Hz, 1H), 7.29 - 7.04 (m, 20H), 7.00 (s, 2H), 6.71 (s, 2H), 4.44 (d, *J* = 5.0 Hz, 2H), 3.87 (s, 3H). **^{13}C NMR (CDCl$_3$, 75 MHz)** δ: 163.2, 159.5, 151.1, 148.9, 147.9, 142.8, 140.7, 138.2, 137.9, 131.9, 130.5, 130.2, 129.1, 128.7, 128.2, 127.7, 126.9, 126.8, 122.4, 116.3, 55.7, 39.1. **ESI$^+$** calcd. for $C_{42}H_{35}N_2O_2$ (M)$^+$: 599.2693; Found: 599.2707.

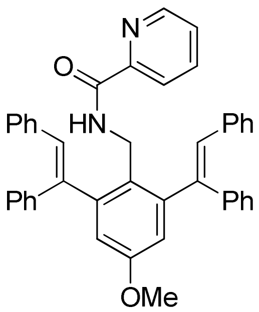

***N*-(2,6-Bis((*E*)-1,2-diphenylvinyl)-4-methylbenzyl)picolinamide (41).** Compound **41** was prepared following the general protocol from *N*-(4-methylbenzyl)picolinamide (**22**) (33.9 mg, 0.15 mmol, 1.00 equiv), to give **41** as a pale yellow solid; yield: 66.7 mg (75%); mp= 159-160 °C. **^{1}H NMR (acetone-d$_6$, 300 MHz)** δ: 8.56 (d, *J* = 4.8 Hz, 1H), 8.04 - 7.87 (m, 3H), 7.53 (ddd, *J* = 7.3, 4.8, 1.4 Hz, 1H), 7.29 - 7.01 (m, 22H), 6.68 (s, 2H), 4.48 (d, *J* = 5.4 Hz, 2H), 2.39 (s, 3H). **^{13}C NMR (CDCl$_3$, 75 MHz)** δ: 162.9, 150.1, 147.7, 145.8, 142.0, 139.8, 137.2, 137.1, 137.0, 131.1, 131.0, 130.6, 129.8, 129.4, 128.3, 128.0, 127.3, 126.8, 125.7, 121.9, 39.3, 21.1. **ESI$^+$** calcd. for $C_{42}H_{35}N_2O$ (M+H)$^+$: 583.2743; Found: 583.2730.

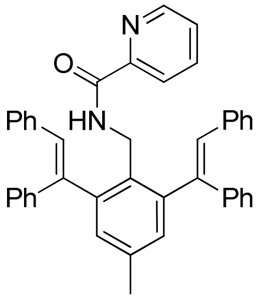

***N*-(2,6-Bis((E)-1,2-diphenylvinyl)benzyl)picolinamide (42).** Compound **42** was prepared following the general protocol from *N*-(4-chlorobenzyl)picolinamide (**23**) (36.9 mg, 0.15 mmol, 1.00 equiv), to give **42** as a pale yellow solid; yield: 89.2 mg (99%); mp= 160-162 °C. **^{1}H NMR (acetone-d$_6$, 300 MHz)** δ: 8.56 (d, *J* = 4.1 Hz, 1H), 8.10 (s, 1H), 8.00 (d, *J* = 7.4 Hz, 1H), 7.93 (td, *J* = 7.6, 1.7 Hz, 1H), 7.54 (ddd, *J* = 7.3, 4.8, 1.5 Hz, 1H), 7.38 (s, 2H), 7.29 - 7.08 (m, 20H), 6.75 (s, 2H), 4.53 (d, *J* = 5.5 Hz, 2H). **^{13}C NMR (acetone-d$_6$, 75 MHz)** δ: 163.5, 150.9, 149.0, 148.4, 141.5, 140.3, 138.3, 137.7, 134.1, 133.3, 132.8, 130.6, 130.4, 130.2, 129.3, 128.8, 128.5, 128.0, 127.0, 122.5, 39.2. **ESI$^+$** calcd. for $C_{41}H_{32}ClN_2O$ (M+H)$^+$: 603.2197; Found: 603.2184.

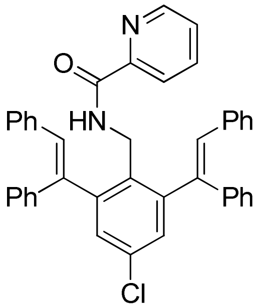

N-(2,6-Bis((E)-1,2-diphenylvinyl)-4-fluorobenzyl)picolinamide (43). Compound **43** was prepared following the general protocol from *N*-(4-fluorobenzyl)picolinamide (**24**) (34.5 mg, 0.15 mmol, 1.00 equiv),to give **43** as a pale yellow solid; yield: 61.4 mg (70%); mp= 134-136 °C. **^{1}H NMR (CDCl$_3$, 300 MHz)** δ: 8.43 (d, *J* = 4.4 Hz, 1H), 8.01 (d, *J* = 7.8 Hz, 1H), 7.76 (dd, *J* = 7.5, 6.2 Hz, 2H), 7.37 (dd, *J* = 6.7, 5.0 Hz, 1H), 7.12 (ddt, *J* = 11.4, 8.8, 4.9 Hz, 22H), 6.67 (s, 2H), 4.43 (d, *J* = 5.1 Hz, 2H). **^{13}C NMR (CDCl$_3$, 75 MHz)** δ: 163.0, 161.4 (d, *J* = 248.5 Hz), 150.0, 147.9 (d, *J* = 7.8 Hz), 147.7, 140.9 (d, *J* = 1.6 Hz), 139.2, 137.0, 136.7, 131.8, 129.8, 129.7, 129.4, 128.5, 128.0, 127.6, 127.1, 125.8, 122.0, 117.0 (d, *J* = 20.8 Hz), 38.9. **ESI$^+$** calcd. for $C_{41}H_{32}FN_2O$ (M+H)$^+$: 587.2493; Found: 587.2479.

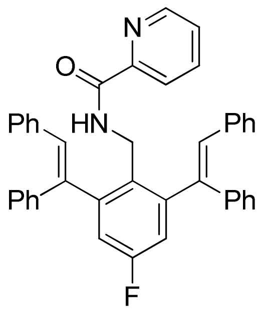

N-(2,6-Bis((E)-1,2-diphenylvinyl)-4-(trifluoromethyl)benzyl)picolinamide (44). Compound **44** was prepared following the general protocol from *N*-(4-(trifluoromethyl)benzyl)picolinamide (**25**) (42.0 mg, 0.15 mmol, 1.00 equiv), to give **44** as a pale yellow solid; yield: 88.7 mg (93%); mp= 157-158 °C. **^{1}H NMR (acetone-d$_6$, 300 MHz)** δ: 8.56 (d, *J* = 3.9 Hz, 1H), 8.23 (s, 1H), 8.02 (d, *J* = 7.8 Hz, 1H), 7.94 (td, *J* = 7.6, 1.5 Hz, 1H), 7.68 (s, 2H), 7.57 - 7.49 (m, 1H), 7.32 - 7.08 (m, 20H), 6.80 (s, 2H), 4.62 (d, *J* = 5.6 Hz, 2H). **^{13}C NMR (acetone-d$_6$, 75 MHz)** δ: 163.6, 150.8, 149.0, 147.5, 141.4, 140.1, 139.8, 138.3, 137.6, 133.1, 130.6, 130.2, 129.3, 128.8, 128.6, 128.0, 127.3 (q, *J* =3.5 Hz), 127.1, 122.5, 39.5. **ESI$^+$** calcd. for $C_{42}H_{32}F_3N_2O$ (M+H)$^+$: 637.2461; Found: 637.2459.

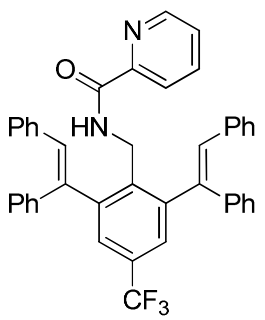

N-(4-Cyano-2,6-bis((E)-1,2-diphenylvinyl)benzyl)picolinamide (45). Compound **45** was prepared following the general protocol from *N*-(4-cyanobenzyl)picolinamide (**26**) (35.6 mg, 0.15 mmol, 1.00 equiv), to give **45** as a pale yellow solid; yield: 39.2 mg (44%); mp= 177-179 °C. **^{1}H NMR (CDCl$_3$, 300 MHz)** δ: 8.42 (d, *J* = 4.4 Hz, 1H), 8.09 - 7.82 (m, 2H), 7.79 (td, *J* = 7.7, 1.6 Hz, 1H), 7.63 (s, 2H), 7.39 (dd, *J* = 6.9, 5.3 Hz, 1H), 7.22 - 7.11 (m, 16H), 7.08 - 7.01 (m, 4H), 6.66 (s, 2H), 4.45 (d, *J* = 5.5 Hz, 2H). **^{13}C NMR (CDCl$_3$, 75 MHz)** δ: 163.2, 149.8, 147.9, 147.0, 140.0, 139.8, 138.8, 137.2, 136.4, 133.4, 132.6, 129.8, 129.5, 128.7, 128.2, 128.0, 127.5, 126.1, 122.1, 118.5, 111.5, 39.4. **ESI$^+$** calcd. for $C_{42}H_{32}N_3O$ (M+H)$^+$: 594.2539; Found: 594.2525.

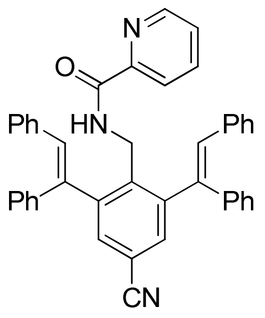

Methyl 3,5-bis((E)-1,2-diphenylvinyl)-4-(picolinamidomethyl)benzoate (46). Compound **46** was prepared following the general protocol from methyl 4-(picolinamidomethyl)benzoate (**27**) (40.5 mg, 0.15 mmol, 1.00 equiv), to give **46** as a pale yellow oil; yield: 70.1 mg (70%). **^{1}H NMR (CDCl$_3$, 300 MHz)** δ: 8.42 (d, *J* = 4.8 Hz, 1H), 8.06 - 7.96 (m, 3H), 7.84 (s, 1H), 7.77 (td, *J* = 7.7, 1.6 Hz, 1H), 7.40 - 7.34 (m, 1H), 7.23 - 7.09 (m, 14H), 7.08 - 7.01 (m, 5H), 6.67 (s, 2H), 4.41 (d, *J* = 5.3 Hz, 2H), 3.93 (s, 3H). **^{13}C NMR (CDCl$_3$, 75 MHz)** δ: 166.9, 163.1, 149.9, 147.8, 146.2, 141.1, 139.3, 139.1, 137.1, 136.9, 131.9, 131.3, 129.8, 129.5, 129.3, 128.5, 128.1, 127.6, 127.1, 125.9, 122.0, 52.4, 39.4. **ESI$^+$** calcd. for $C_{43}H_{34}N_2O_3$ (M+H)$^+$: 627.2569; Found: 627.2666.

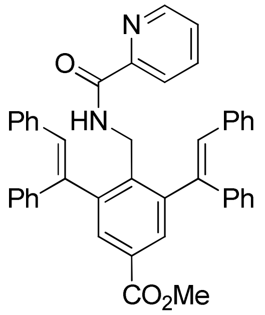

(*E*)-*N*-(2-(1,2-Diphenylvinyl)-5-methylbenzyl)picolinamide (47a). Compound **47a** was prepared following the general protocol from *N*-(3-methylbenzyl)picolinamide (**28**) (33.9 mg, 0.15 mmol, 1.00 equiv), to give **47a** as a pale yellow oil; yield: traces. **^{1}H NMR ($CDCl_3$, 300 MHz)** δ: 8.48 (d, *J* = 4.3 Hz, 1H), 8.14 (d, *J* = 7.8 Hz, 1H), 8.03 (s, 1H), 7.81 (td, *J* = 7.7, 1.6 Hz, 1H), 7.39 (dd, *J* = 6.5, 4.8 Hz, 1H), 7.29 - 7.07 (s, 13H), 6.70 (s, 1H), 4.43 (d, *J* = 6.1 Hz, 2H), 2.35 (s, 3H). **ESI$^+$** calcd. for $C_{28}H_{25}N_2O$ $(M+H)^+$: 405.1961; Found: 405.1964.

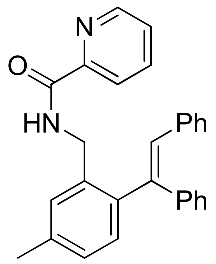

In this experiment, ***N*-(2,6-bis((*E*)-1,2-diphenylvinyl)-3-methylbenzyl)picolinamide (47b)** was also obtained as a pale yellow oil; yield: 72.5 mg (83%). **^{1}H NMR ($CDCl_3$, 300 MHz)** δ: .35 (d, *J* = 4.4 Hz, 1H), 7.92 (d, *J* = 7.8 Hz, 1H), 7.68 (td, *J* = 7.7, 1.5 Hz, 1H), 7.60 (s, 1H), 7.31 - 6.94 (m, 23H), 6.64 (s, 1H), 6.53 (s, 1H), 4.46 (ddd, *J* = 44.9, 14.0, 4.9 Hz, 2H), 2.36 (s, 3H). **^{13}C NMR ($CDCl_3$, 75 MHz)** δ: 162.9, 150.1, 147.6, 145.1, 143.5, 142.1, 140.0, 139.4, 138.6, 137.3, 137.3, 137.0, 136.7, 133.6, 131.6, 130.9, 130.0, 129.8, 129.8, 129.7, 129.4, 129.3, 128.4, 128.1, 128.0, 127.3, 127.3, 126.9, 126.8, 125.7, 121.9, 39.9, 20.8. **ESI$^+$** calcd. for $C_{42}H_{35}N_2O$ $(M+H)^+$: 583.2743; Found: 583.2739.

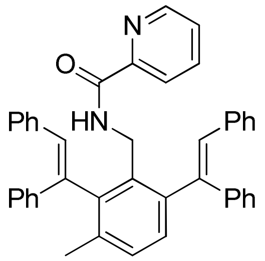

(*E*)-*N*-(2-(1,2-Diphenylvinyl)-5-(trifluoromethyl)benzyl)picolinamide (48). Compound **48** was prepared following the general protocol from *N*-(3-(trifluoromethyl)benzyl)picolinamide (**29**) (42.0 mg, 0.15 mmol, 1.00 equiv). In this experiment a mixture of Hexane:AcOEt:$CHCl_3$ (8:1:1) was used in the chromatography column to give to give **48** as a pale yellow oil; yield: 34.4 mg (50%). **^{1}H NMR ($CDCl_3$, 300 MHz)** δ: 8.52 (d, *J* = 4.6 Hz, 1H), 8.19 (d, *J* = 7.8 Hz, 1H), 8.15 (s, 1H), 7.85 (td, *J* = 7.7, 1.5 Hz, 1H), 7.72 (s, 1H), 7.61 (d, *J* = 8.1 Hz, 1H), 7.52 (d, *J* = 7.9 Hz, 1H), 7.44 (dd, *J* = 7.0, 5.3 Hz, 1H), 7.31 -7.15 (m, 10H), 6.76 (s, 1H), 4.55 (d, *J* = 6.2 Hz, 2H). **^{13}C NMR ($CDCl_3$, 75 MHz)** δ: 164.1, 149.7, 148.0, 147.3, 147.3, 140.7, 139.1, 137.5, 137.4, 136.6, 132.0, 131.2, 130.2 (d, *J* = 32.5 Hz), 129.8, 129.6, 128.8, 128.2, 128.0, 127.5, 126.1 (q, *J* = 3.7 Hz), 124.4 (q, *J* = 3.3 Hz), 124.4 (q, *J* = 293.4 Hz), 122.3, 41.3. **EI$^+$** calcd. for $C_{28}H_{21}F_3N_2O$ $(M)^+$: 458.1606; Found: 458.1590.

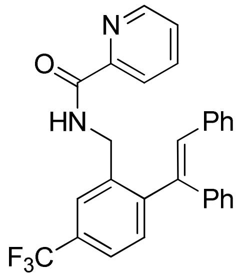

(*E*)-*N*-(2-(1,2-Diphenylvinyl)-6-methylbenzyl)picolinamide (49). Compound **49** was prepared following the general protocol from *N*-(2-methylbenzyl)picolinamide (**30**) (33.9 mg, 0.15 mmol, 1.00 equiv), to give **49** as a pale yellow solid; yield: 60.0 mg (99%); mp= 147-148 °C. **^{1}H NMR (acetone-d_6, 300 MHz)** δ: 8.53 (d, *J* = 4.7 Hz, 1H), 8.04 (dd, *J* = 7.8, 1.0 Hz, 1H), 7.93 (td, *J* = 7.7, 1.7 Hz, 1H), 7.66 (s, 1H), 7.52 (ddd, *J* = 7.5, 4.8, 1.3 Hz, 1H), 7.32 - 7.10 (m, 13H), 6.74 (s, 1H), 4.57 (d, *J* = 5.6 Hz, 2H), 2.39 (s, 3H). **^{13}C NMR (acetone-d_6, 75 MHz)** δ: 163.6, 150.9, 149.0, 146.3, 143.5, 141.2, 139.1, 138.3, 138.1, 135.0, 131.4, 130.9, 130.4, 130.2, 129.4, 128.9, 128.5, 128.3, 127.8, 127.0, 122.4, 39.0, 19.8. **ESI$^+$** calcd. for $C_{28}H_{25}N_2O$ $(M+H)^+$: 405.1961; Found: 405.1967.

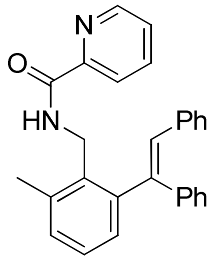

(*E*)-*N*-(2-Bromo-6-(1,2-diphenylvinyl)benzyl)picolinamide (50). Compound **50** was prepared following the general protocol from *N*-(2-bromobenzyl)picolinamide (**31**) (43.5 mg, 0.15 mmol, 1.00 equiv), to give **50** as a pale yellow solid; yield: 48.4 mg (69%); mp= 67-68 °C. **^{1}H NMR (acetone-d_6, 300 MHz)** δ: 8.54 (d, *J* = 4.8 Hz, 1H), 8.06 (d, *J* = 7.8 Hz, 1H), 8.01 - 7.91 (m, 2H), 7.67 (dd, *J* = 7.9, 1.4 Hz, 1H), 7.53 (ddd, *J* = 7.5, 4.8, 1.3 Hz, 1H), 7.44 - 7.28 (m, 2H), 7.25 - 7.11 (m, 10H), 6.75 (s, 1H), 4.75 (d, *J* = 5.4 Hz, 2H). **^{13}C NMR (acetone-d_6, 75 MHz)** δ: 163.6, 150.8, 149.0, 148.2, 142.0, 140.6, 138.3, 137.7, 136.1, 133.3, 132.5, 131.1, 130.5, 130.2, 130.2, 129.3, 128.8, 128.4, 128.0, 127.1, 126.7, 122.5, 42.1. **ESI$^+$** calcd. for $C_{27}H_{22}BrN_2O$ $(M+H)^+$: 469.0910; Found: 469.0903.

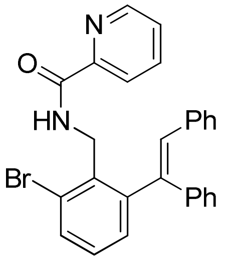

(*E*)-*N*-(2-(1,2-Diphenylvinyl)-6-fluorobenzyl)picolinamide (51). Compound **51** was prepared following the general protocol from *N*-(2-fluorobenzyl)picolinamide (**32**) (34.5 mg, 0.15 mmol, 1.00 equiv), to give **51** as a pale yellow oil; yield: 56 mg (92%). **^{1}H NMR ($CDCl_3$, 300 MHz)** δ: 8.26 (d, *J* = 4.7 Hz, 1H), 7.92 (d, *J* = 7.8 Hz, 1H), 7.72 (s, 1H), 7.58 (td, *J* = 7.7, 1.7 Hz, 1H), 7.20 - 7.12 (m, 1H), 7.12 - 7.04 (m, 1H), 7.03 - 6.83 (m, 12H), 6.52 (s, 1H), 4.38 (d, *J* = 5.6 Hz, 2H). **^{13}C NMR ($CDCl_3$, 75 MHz)** δ: 163.5, 160.2, 149.9, 147.9, 146.8 (d, *J* = 3.5 Hz), 140.7 (d, *J* = 2.6 Hz), 139.6, 137.2, 136.8, 131.7, 129.8, 129.5, 129.0 (d, *J* = 9.4 Hz), 128.6, 128.1, 127.7, 127.2, 126.5 (d, *J* = 3.0 Hz), 126.0, 123.3 (d, *J* = 14.9 Hz), 122.2, 115.0 (d, *J* = 22.8 Hz), 35.0 (d, *J* = 4.9 Hz). **^{19}F NMR ($CDCl_3$, 282 MHz)** δ: -115.6. **EI$^+$** calcd. for $C_{27}H_{21}FN_2O$ $(M)^+$: 408.1638; Found: 408.1635.

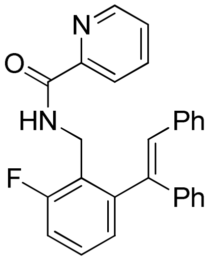

(*E*)-*N*-((3-(1,2-Diphenylvinyl)furan-2-yl)methyl)picolinamide (52). Compound **52** was prepared following the general protocol from *N*-(furan-2-ylmethyl)picolinamide (**33**) (30.3 mg, 0.15 mmol, 1.00 equiv). In this experiment a mixture of Hexane:AcOEt:CH_2Cl_2 (10:1:1) was used in the chromatography column to give to give **52** as an pale yellow oil; yield: 24.1 mg (42%). **^{1}H NMR ($CDCl_3$, 500 MHz)** δ: 8.54 (d, *J* = 4.6 Hz, 1H), 8.17 (d, *J* = 7.7 Hz, 1H), 8.06 (s, 1H), 7.83 (t, *J* = 7.0 Hz, 1H), 7.46 - 7.38 (m, 1H), 7.38 - 7.22 (m, 8H), 7.17 - 6.99 (m, 4H), 6.80 (s, 1H), 6.38 (d, *J* = 1.6 Hz, 1H), 4.32 (d, *J* = 5.7 Hz, 2H). **^{13}C NMR ($CDCl_3$, 75 MHz)** δ: 164.0, 149.9, 148.1, 148.0, 141.7, 140.0, 138.9, 137.4, 137.0, 134.2, 129.8, 129.5, 128.9, 128.7, 128.1, 127.9, 126.9, 126.2, 122.4, 111.5, 35.7. **ESI$^+$** calcd. for $C_{25}H_{21}N_2O_2$ $(M)^+$: 381.1597; Found: 381.1611.

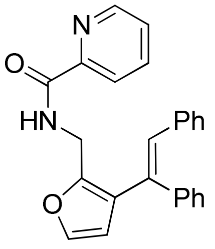

8.2. Scope with regard to the alkyne

8.2.1. Scope with regard to the aryl-aryl alkyne

Synthesis of *N*-(2,6-bis((*E*)-1,2-bis(4-methoxyphenyl)vinyl)benzyl)picolinamide (34). Compound **34** was prepared following the general protocol from 1,2-bis(4-methoxyphenyl)ethyne (**I**) (71.4 mg, 0.30 mmol, 2.00 equiv) to give **34** as an orange solid; yield: 83.9 mg (81%); mp= 158-159 °C. **^{1}H NMR (acetone-d_6, 300 MHz)** δ: 8.54 (d, *J* = 4.8 Hz, 1H), 7.92 (ddd, *J* = 11.1, 9.4, 4.7 Hz, 1H), 7.51 (ddd, *J* = 7.2, 4.7, 1.4 Hz, 1H), 7.37 -

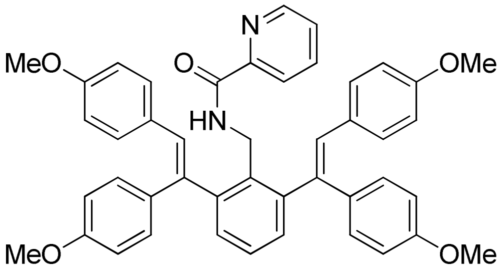

7.30 (m, 3H), 7.14 (d, *J* = 8.8 Hz, 4H), 7.05 (d, *J* = 8.9 Hz, 4H), 6.75 - 6.70 (m, 8H), 6.53 (s, 2H), 4.48 (d, *J* = 5.3 Hz, 2H), 3.73 (s, 6H), 3.67 (s, 6H). **^{13}C NMR (acetone-d$_6$, 75 MHz)** δ: 163.2, 159.8, 159.5, 151.1, 149.0, 147.1, 140.7, 138.1, 134.7, 133.3, 131.7, 131.4, 130.7, 130.7, 130.5, 128.1, 126.8, 122.4, 114.5, 114.2, 55.4, 55.3, 39.7. **ESI$^+$** calcd. for $C_{45}H_{41}N_2O_5$ $(M+H)^+$: 689.3009; Found: 689.3001.

***N*-(2,6-Bis((*E*)-1,2-bis(4-(*tert*-butyl)phenyl)vinyl)benzyl)picolinamide (35).** Compound **35** was prepared following the general protocol from 1,2-bis(4-(*tert*-butyl)phenyl)ethyne (**II**) (87.0 mg, 0.30 mmol, 2.00 equiv). In this experiment a mixture of Hexane:AcOEt (6:1) was used in the chromatography column to give **35** as a pale yellow solid; yield: 84 mg (71%); mp= 115-117 °C. **^{1}H NMR (CDCl$_3$, 300 MHz)** δ: 8.54 - 8.39 (m, 1H), 8.04 (d, *J* = 7.8 Hz, 1H), 7.84 - 7.64 (m, 2H), 7.37 (s, 4H), 7.20 (d, *J* = 1.2 Hz, 7H), 7.14 (d, *J* = 8.5 Hz, 4H), 7.01 (d, *J* = 8.4 Hz, 4H), 6.59 (s, 2H), 4.49 (d, *J* = 5.0 Hz, 1H), 1.28 (s, 18H), 1.20 (s, 18H). **^{13}C NMR (CDCl$_3$, 75 MHz)** δ: 162.9, 150.2, 150.1, 149.7, 147.8, 146.4, 141.0, 137.0, 136.9, 134.3, 133.5, 130.7, 130.1, 129.3, 129.0, 127.4, 125.6, 125.1, 124.8, 122.1, 39.6, 34.5 (d, *J* = 5.1 Hz), 31.7, 31.3 (d, *J* = 4.5 Hz), 22.7, 14.2. **ESI$^+$** calcd. for $C_{57}H_{65}N_2O$ $(M+H)^+$: 793.5091; Found: 793.5108.

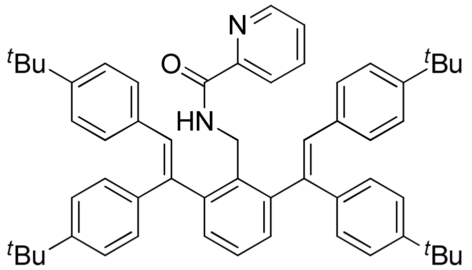

***N*-(2,6-Bis((*E*)-1,2-di-*p*-tolylvinyl)benzyl)picolinamide (36).** Compound **36** was prepared following the general protocol and 1,2-di-*p*-tolylethyne (**III**) (61.8 mg, 0.30 mmol, 2.00 equiv) to give **36** as a pale yellow solid; yield: 72.0 mg (77%); mp= 110-112 °C. **^{1}H NMR (acetone-d$_6$, 300 MHz)** δ: 8.56 (d, *J* = 4.7 Hz, 1H), 8.00 - 7.94 (m, 1H), 7.89 (td, *J* = 7.6, 1.7 Hz, 1H), 7.81 (s, 1H), 7.50 (ddd, *J* = 7.4, 4.8, 1.4 Hz, 1H), 7.40 - 7.30 (m, 3H), 7.11 (d, *J* = 8.1 Hz, 2H), 7.03 - 6.89 (m, 13H), 6.60 (s, 2H), 4.48 (d, *J* = 5.2 Hz, 2H), 2.22 (s, 6H), 2.15 (s, 6H). **^{13}C NMR (acetone-d$_6$, 75 MHz)** δ: 163.1, 151.1, 148.9, 146.9, 142.0, 138.1, 138.1, 137.7, 137.2, 135.3, 134.6, 131.4, 130.8, 130.4, 130.1, 129.8, 129.4, 128.2, 126.7, 122.3, 39.7, 21.1, 21.1. **FB$^+$** calcd. for $C_{45}H_{41}N_2O$ $(M+H)^+$: 625.3219; Found: 625.3234.

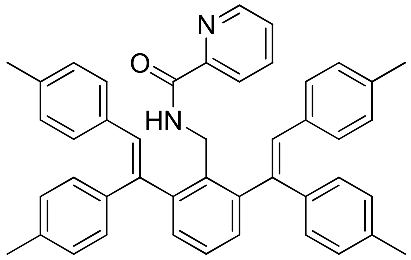

***N*-(2,6-Bis((*E*)-1,2-bis(4-(trifluoromethyl)phenyl)vinyl)benzyl)picolinamide (37).** Compound **37** was prepared following the general protocol 1,2-bis(4-(trifluoromethyl)phenyl)ethyne (**IV**) (94.2 mg, 0.30 mmol, 2.00 equiv), to give **37** as a white solid; yield: 106 mg (84%); mp= 186-187 °C. **^{1}H NMR (acetone -d$_6$, 300 MHz)** δ: 8.54 (d, *J* = 4.7 Hz, 1H), 8.02 - 7.89 (m, 2H), 7.85 (s, 1H), 7.58 - 7.43 (m, 16H), 7.32 (d, *J* = 8.1 Hz, 4H), 6.94 (s, 2H), 4.59 (d, *J* = 5.4 Hz, 2H). **^{13}C NMR (acetone-d$_6$, 75 MHz)** δ: 163.4, 150.6, 149.0, 145.6, 144.4, 143.5, 141.6, 138.4, 134.9, 132.2, 131.6, 131.3, 130.8, 129.9 (q, *J* = 32.2 Hz), 129.4 (q, *J* = 32.2 Hz), 128.9, 127.1, 126.3 (q, *J* = 3.7 Hz), 125.9 (q, *J* = 3.8 Hz), 125.2 (q, *J* = 271.1 Hz), 125.1 (q, *J* = 271.5 Hz), 122.5, 39.6. **ESI$^+$** calcd. for $C_{45}H_{29}F_{12}N_2O$ $(M+H)^+$: 841.2082; Found: 841.2064.

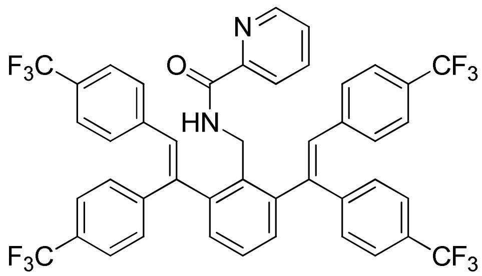

_N_-(2,6-Bis((_E_)-1,2-bis(3-methoxyphenyl)vinyl)benzyl)picolinamide (38). Compound **38** was prepared following the general protocol from 1,2-bis(3-methoxyphenyl)ethyne (**V**) (71.4 mg, 0.30 mmol, 2.00 equiv), to give **38** as a yellow solid; yield: 56.1 mg (54%); mp= 78-79 °C. **^{1}H NMR ($CDCl_3$, 300 MHz)** δ: 8.40 (d, *J* = 4.3 Hz, 1H), 8.00 (d, *J* = 7.8 Hz, 1H), 7.75 (dt, *J* = 7.6, 3.8 Hz, 2H), 7.36 (s, 4H), 7.04 (t, *J* = 7.9 Hz, 4H), 6.83 - 6.75 (m, 4H), 6.70 - 6.54 (m, 10H), 4.48 (d, *J* = 5.1 Hz, 2H), 3.55 (d, *J* = 5.2 Hz, 12H). **^{13}C NMR ($CDCl_3$, 126 MHz)** δ: 162.9, 159.4, 159.2, 150.1, 147.7, 145.7, 142.0, 141.2, 138.4, 137.0, 133.5, 131.3, 130.3, 129.4, 129.0, 127.6, 125.7, 122.4, 122.2, 121.9, 114.9, 114.1, 113.5, 113.5, 55.1, 55.0, 39.7. **ESI$^+$** calcd. for $C_{45}H_{41}N_2O_5$ $(M+H)^+$: 689.3015; Found: 689.3001.

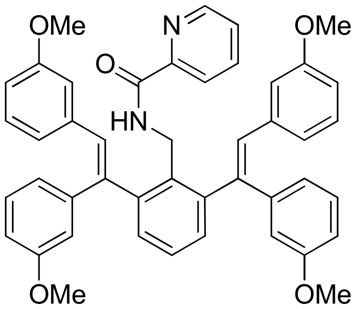

8.2.2. Scope with regard to the alkyl-aryl alkyne (Scheme 3)

_N_-(2,6-Bis((_E_)-1-cyclohexyl-2-(4-methoxyphenyl)vinyl)benzyl)picolinamide (58). Compound **58** was prepared following the general protocol from 1-(cyclohexylethynyl)-4-methoxybenzene (**VII**) (64.2 mg, 0.30 mmol, 2.00 equiv). In this experiment *n*-hexane was used as only eluent in the chromatography column to give **58** as a yellow oil; yield: 78.1 mg (88%). **^{1}H NMR ($CDCl_3$, 300 MHz)** δ: 8.43 (d, *J* = 4.4 Hz, 1H), 8.18 (s, 1H), 8.10 (d, *J* = 7.8 Hz, 1H), 7.77 (t, *J* = 7.6 Hz, 1H), 7.36 - 7.24 (m, 2H), 7.17 (d, *J* = 7.0 Hz, 6H), 6.83 (d, *J* = 8.5 Hz, 4H), 6.33 (s, 2H), 4.70 (s, 2H), 3.82 (s, 6H), 2.98 (t, *J* = 11.1 Hz, 2H), 1.97 - 1.53 (m, 10H), 1.37 - 0.80 (m, 10H). **^{13}C NMR ($CDCl_3$, 126 MHz)** δ: 162.9, 158.2, 150.3, 148.0, 145.6, 144.1, 137.2, 130.2, 130.1, 129.4, 129.3, 128.9, 125.9, 125.7, 125.7, 122.2, 113.6, 55.3, 40.8, 31.0, 26.5, 26.0. **ESI$^+$** calcd. for $C_{43}H_{49}N_2O_3$ $(M+H)^+$: 641.3737; Found: 641.3721.

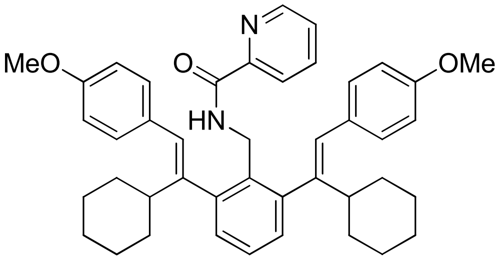

_N_-(2,6-Bis((_E_)-1-cyclohexyl-2-(4-(trifluoromethyl)phenyl)vinyl)benzyl)picolinamide (59). Compound **59** was prepared following the general protocol from 1-(cyclohexylethynyl)-4-(trifluoromethyl)benzene (**VIII**) (75.6 mg, 0.30 mmol, 2.00 equiv). In this experiment an increase of the catalityc species was necessary, thus (7.39 mg, 0.015 mmol, 0.1 equiv) of $[Rh(cod)Cl]_2$ and (10.3 mg, 0.03 mmol, 0.2 equiv) of $AgSbF_6$ was used. Likewise *n*-hexane was used as only eluent in the chromatography column to give **59** as a pale yellow oil; yield: 92.5 mg (86%). **^{1}H NMR ($CDCl_3$, 300 MHz)** δ: 8.38 (d, *J* = 4.9 Hz, 1H), 8.18 (s, 1H), 8.06 (d, *J* = 7.6 Hz, 1H), 7.76 (t, *J* = 7.7 Hz, 1H), 7.53 (d, *J* = 8.2 Hz, 3H), 7.37 - 7.29 (d, *J* = 8.0 Hz, 5H), 7.17 (d, *J* = 7.5 Hz, 2H), 6.39 (s, 2H), 4.70 (s, 2H), 2.97 - 2.84 (s, 2H) 1.84 - 1.62 (m, 10H), 1.26 - 0.91 (m, 12H). **^{13}C NMR ($CDCl_3$, 75 MHz)** δ: 163.0, 150.0, 148.7, 148.2, 148.0, 143.4, 141.2, 137.4, 133.5, 129.8, 129.2, 129.0 (q, *J* = 32.4 Hz), 128.9, 128.4, 127.9, 126.2, 126.1, 125.2 (q, *J* = 3.8 Hz), 124.4 (q, *J* = 271.9 Hz), 122.2, 41.1, 32.9, 26.3, 25.9, 1.1. **ESI$^+$** calcd. for $C_{43}H_{43}F_6N_2O$ $(M+H)^+$: 717.3274; Found: 717.3307.

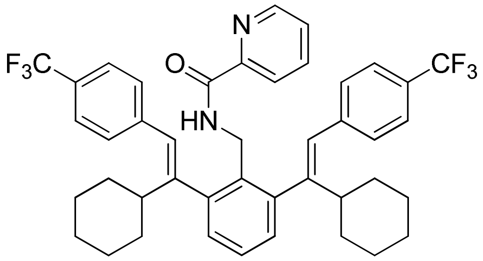

***N*-(2,6-Bis((*E*)-1-cyclohexyl-2-(thiophen-3-yl)vinyl)benzyl)picolinamide (60).** Compound **60** was prepared following the general protocol from 2-(cyclohexylethynyl)thiophene (**IX**) (57.2 mg, 0.30 mmol, 2.00 equiv), to give **60** as a colorless oil; yield: 67.5 mg (76%). **^{1}H NMR ($CDCl_3$, 300 MHz)** δ: 8.49 (s, 1H), 8.10 (s, 1H), 8.01 (d, *J* = 7.8 Hz, 1H), 7.75 (t, *J* = 7.7 Hz, 1H), 7.40 - 7.34 (m, 1H), 7.25 - 7.19 (m, 3H), 7.13 (d, *J* = 7.2 Hz, 2H), 6.90 (s, 3H), 6.74 (s, 2H), 6.48 (s, 2H), 4.59 (s, 2H), 3.28 - 3.12 (s, 2H). 2.08 - 1.62 (m, 10H), 1.52 - 0.81 (m, 10H). **^{13}C NMR ($CDCl_3$, 75 MHz)** δ: 162.9, 150.2, 147.9, 145.0, 144.9, 143.9, 139.7, 137.2, 133.6, 128.6, 127.7, 126.7, 126.1, 125.9, 125.3, 122.5, 122.2, 41.6, 32.5, 30.8, 26.7, 26.0. **ESI$^+$** calcd. for $C_{37}H_{41}N_2OS_2$ $(M+H)^+$: 593.2654; Found: 593.2645.

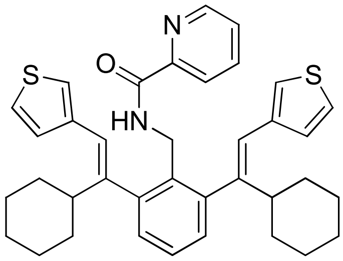

8.3. Scope with regard to the 1,3-enyne (Scheme 4)

Synthesis of (2*E*,2'*E*,4*E*,4'*E*)-dimethyl 5,5'-(2-(picolinamidomethyl)-1,3-phenylene)bis(6-cyclohexylhexa-2,4-dienoate) (55). An oven-dried, nitrogen-flushed 20 mL vessel was charged with *N*-benzylpicolinamide (**1**) (31.8 mg, 0.15 mmol, 1.00 equiv), chloro(1,5-cyclooctadiene), rhodium dimer (3.96 mg, 0.0075 mmol, 0.05 equiv), sodium acetate (49.8 mg, 0.60 mmol, 4.00 equiv), and silver hexafluoroantimonate (5.85 mg, 0.015 mmol, 0.10 equiv). Under oxygen atmosphere the solvent 1,2-dichloroethane (1.00 mL) and the (*E*)-methyl 6-cyclohexylhex-2-en-4-ynoate (**X**) (61.8 mg, 0.30 mmol, 2.00 equiv) were added *via* syringe and the resulting mixture was saturated of oxygen by bubbling at 0 °C for 10 min. Then the reaction was stirred at 120 °C for 12 h. After the reaction was complete, the volatiles were removed *in vacuo* and the residue was purified by column chromatography (cyclohexane-AcOEt-CH_2Cl_2 2:1:1), yielding **55** as a yellow oil; yield: 84.3 mg (90%). **^{1}H NMR ($CDCl_3$, 300 MHz)** δ: 8.42 (d, *J* = 4.6 Hz, 1H), 8.11 (d, *J* = 7.8 Hz, 1H), 7.99 (s, 1H), 7.78 (td, *J* = 7.7, 1.6 Hz, 1H), 7.58 (dd, *J* = 15.2, 11.6 Hz, 2H), 7.40 - 7.32 (m, 1H), 7.30 (t, *J* = 7.7 Hz, 1H), 7.07 (d, *J* = 7.6 Hz, 2H), 6.18 (d, *J* = 11.6 Hz, 2H), 5.71 (d, *J* = 15.1 Hz, 2H), 4.56 (d, *J* = 5.0 Hz, 2H), 3.73 (s, 6H), 2.52 (d, *J* = 6.9 Hz, 4H), 1.72 - 1.55 (m, 10H), 1.32 - 1.23 (m, 2H), 1.18 - 1.04 (m, 6H), 1.02 - 0.87 (m, 4H). **^{13}C NMR ($CDCl_3$, 75 MHz)** δ: 167.6, 163.4, 150.2, 149.6, 148.0, 145.0, 140.1, 137.3, 131.3, 129.5, 128.4, 127.4, 126.2, 122.3, 121.4, 51.6, 40.9, 39.3, 36.6, 33.5, 26.4, 26.3. **ESI$^+$** calcd. for $C_{39}H_{49}N_2O_5$ $(M+H)^+$: 625.3641; Found: 625.3644. Configuration determined by analysis of its ^{1}H NMR and a nOe experiment.

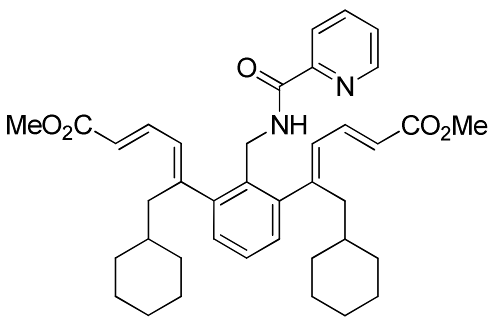

(2*E*,2'*E*,4*E*,4'*E*)-Dimethyl 5,5'-(2-(picolinamidomethyl)-1,3-phenylene)bis(7-phenylhepta-2,4-dienoate) (56). Compound **56** was prepared following the general protocol from (*E*)-methyl 7-phenylhept-2-en-4-ynoate (**XI**) (64.2 mg, 0.30 mmol, 2.00 equiv), to give **56** as a orange oil; yield: 89.3 mg (93%). **^{1}H NMR ($CDCl_3$, 300 MHz)** δ: 8.42 (d, *J* = 4.7 Hz, 1H), 8.11 (d, *J* = 7.8 Hz, 1H), 8.07 (s, 1H), 7.78 (td, *J* = 7.8, 1.6 Hz, 1H),

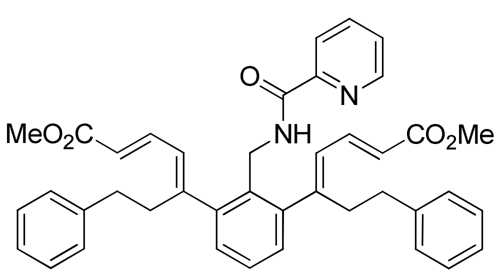

7.55 (dd, J = 15.1, 11.7 Hz, 2H), 7.41 - 7.33 (m, 1H), 7.29 - 7.07 (m, 13H), 6.16 (d, J = 11.7 Hz, 2H), 5.74 (d, J = 15.1 Hz, 2H), 4.57 (d, J = 5.1 Hz, 2H), 3.74 (s, 6H), 3.02 - 2.94 (m, 4H), 2.74 - 2.66 (m, 4H). **^{13}C NMR ($CDCl_3$, 75 MHz)** δ: 167.4, 163.4, 150.1, 149.6, 148.0, 144.4, 140.8, 139.3, 137.4, 131.7, 128.7, 128.6, 128.5, 128.4, 127.6, 126.2, 122.4, 121.9, 51.6, 39.4, 34.8, 34.7. **ESI$^+$** calcd. for $C_{41}H_{41}N_2O_5$ $(M+H)^+$: 641.2937; Found: 641.2943.

(2*E*,2'*E*,4*E*,4'*E*)-Dimethyl 5,5'-(2-(picolinamidomethyl)-1,3-phenylene)bis(9-chloronona-2,4-dienoate) (57). Compound **57** was prepared following the general protocol from (E)-methyl 9-chloronon-2-en-4-ynoate (**XII**) (60.0 mg, 0.30 mmol, 2.00 equiv), to give **57** as a orange oil; yield: 66.2 mg (72%). %). **^{1}H NMR ($CDCl_3$, 300 MHz)** δ: 8.44 (d, J = 4.7 Hz, 1H), 8.11 (d, J = 7.8 Hz, 1H), 8.02 (s, 1H), 7.79 (td, J = 7.7, 1.7 Hz, 1H), 7.58 (dd, J = 15.2, 11.7 Hz, 2H), 7.41 - 7.35 (m, 1H), 7.28 (t, J = 7.4 Hz, 1H), 7.09 (d, J = 7.7 Hz, 2H), 6.14 (d, J = 11.7 Hz, 2H), 5.75 (d, J = 15.1 Hz, 2H), 4.56 (d, J = 5.1 Hz, 2H), 3.74 (s, 6H), 3.46 (t, J = 6.5 Hz, 4H), 2.69 - 2.61 (m, 4H), 1.83 - 1.71 (m, 4H), 1.59 - 1.47 (m, 4H). **^{13}C NMR ($CDCl_3$, 75 MHz)** δ: 167.5, 163.4, 150.6, 149.5, 148.0, 144.3, 139.3, 137.4, 131.5, 128.6, 128.5, 127.6, 126.3, 122.4, 122.0, 53.5, 51.6, 44.6, 39.3, 32.4, 26.0. **ESI$^+$** calcd. for $C_{33}H_{39}Cl_2N_2O_5$ $(M+H)^+$: 613.2158; Found: 613.2160.

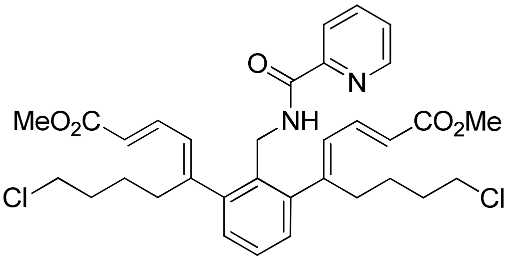

9. Rhodium-controlled divergent aryl/heteroaryl C–H functionalization using an alkynyl propiolate (Scheme 5)

Synthesis of (2*E*,2'*E*)-diethyl 3,3'-(2-(picolinamidomethyl)-1,3-phenylene)bis(pent-2-enoate) (54). An oven-dried, nitrogen-flushed 20 mL vessel was charged with *N*-benzylpicolinamide (**1**) (31.8 mg, 0.15 mmol, 1.00 equiv), chloro(1,5-cyclooctadiene), rhodium dimer (3.96 mg, 0.0075 mmol, 0.05 equiv), sodium acetate (49.8 mg, 0.60 mmol, 4.00 equiv), and silver hexafluoroantimonate (5.85 mg, 0.015 mmol, 0.1 equiv). Under oxygen atmosphere the solvent 1,2-dichloroethane (1.00 mL) and the ethyl 2-pentynoate (39.5 μL, 0.30 mmol, 2.00 equiv) were added *via* syringe and the resulting mixture was saturated of oxygen by bubbling at 0 °C for 10 min. Then the reaction was stirred at 120 °C for 12 h. After the reaction was complete, the volatiles were removed *in vacuo* and the residue was purified by column chromatography (cyclohexane-AcOEt-CH_2Cl_2 2:1:1), yielding **54** as a dark orange oil; yield: 27.9 mg (40%). **^{1}H NMR ($CDCl_3$, 300 MHz)** δ: 8.46 (d, J = 4.6 Hz, 1H), 8.15 (d, J = 7.9 Hz, 1H), 8.04 (s, 1H), 7.81 (td, J = 7.7, 1.5 Hz, 1H), 7.43 - 7.33 (m, 1H), 7.30 (t, J = 7.8 Hz, 1H), 7.08 (d, J = 7.6 Hz, 2H), 5.76 (s, 2H), 4.61 (d, J = 5.1 Hz, 2H), 4.10 (q, J = 7.1 Hz, 4H), 2.95 (q, J = 7.5 Hz, 4H), 1.21 (t, J = 7.1 Hz, 6H), 1.02 (t, J = 7.5 Hz, 6H). **^{13}C NMR ($CDCl_3$, 75 MHz)** δ: 165.8, 162.4, 149.8, 148.0, 143.9, 137.3, 130.5, 128.1, 127.3, 126.0, 122.2, 120.0, 59.9, 39.4, 27.1, 14.3, 12.6. **ESI$^+$** calcd. for $C_{27}H_{33}N_2O_5$ $(M+H)^+$: 465.2311; Found: 465.2313.

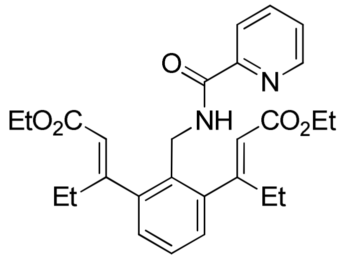

Ethyl 2-(6-benzyl-5-ethyl-7-oxo-6,7-dihydro-5H-pyrrolo[3,4-*b*]pyridin-5-yl)acetate (53). An

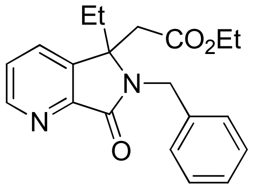

oven-dried, nitrogen-flushed 20 mL vessel was charged with *N*-benzylpicolinamide (**1**) (31.8 mg, 0.15 mmol, pentamethyl-cyclopentadienylrhodium(III) chloride dimer (4.64 mg, 0.0075 mmol, 0.050 equiv), copper(II) acetate (54.5 mg, 0.30 mmol, 2.00 equiv) and silver hexafluoroantimonate(V) (10.7 mg, 0.03 mmol, 0.20 equiv). The reaction vessel was sealed with a Teflon lined cap, then evacuated and flushed with nitrogen three times. Under the atmosphere of nitrogen, 1,2-dichloroethane (1.00 mL) and the ethyl 2-pentynoate (39.5 µL, 0.30 mmol, 2.00 equiv) were added *via* syringe. The resulting mixture was then stirred at 120 °C for 24 h. After the reaction was complete, the volatiles were removed *in vacuo* and the residue was purified by column chromatography (cyclohexane-AcOEt-CH_2Cl_2 8:1:1), yielding **53** as a brown oil; yield: 45.5 mg (90%). **^{1}H NMR ($CDCl_3$, 300 MHz)** δ: 8.78 (d, *J* = 4.7 Hz, 1H), 7.73 (d, *J* = 7.8 Hz, 1H), 7.51 - 7.41 (m, 3H), 7.34 - 7.22 (m, 3H), 4.75 (dd, *J* = 47.7, 15.4 Hz, 2H), 3.80 - 3.60 (m, 2H), 2.81 (d, *J* = 3.0 Hz, 2H), 2.06 - 1.88 (m, 2H), 0.91 (t, *J* = 7.1 Hz, 3H), 0.21 (t, *J* = 7.2 Hz, 3H). **^{13}C NMR ($CDCl_3$, 75 MHz)** δ: 168.3, 151.1, 141.0, 137.4, 129.8, 128.9, 128.6, 127.7, 125.5, 65.8, 60.8, 43.4, 42.2, 29.5, 13.8, 6.9. **EI$^+$** calcd. for $C_{20}H_{22}N_2O_3$ $(M)^+$: 338.1630; Found: 338.1641.

10. Rh(I)-catalyzed *ortho*-olefination of phenethylamine derivatives (Scheme 6)

10.1. Scope with regard to the phenethylamine

Synthesis of *N*-(2,6-bis((*E*)-1,2-diphenylvinyl)phenethyl)picolinamide (73). An oven-dried,

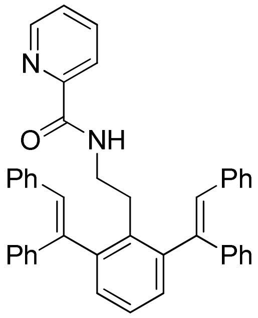

nitrogen-flushed 20 mL vessel was charged with *N*-phenethylpicolinamide (**61**) (33.9 mg, 0.15 mmol, 1.00 equiv), diphenylacetylene (53.3 mg, 0.30 mmol, 2.00 equiv), chloro(1,5-cyclooctadiene)rhodium dimer (3.70 mg, 0.0075 mmol, 0.05 equiv), sodium acetate (49.8 mg, 0.60 mmol, 4.00 equiv), and silver hexafluoroantimonate(V) (2.58 mg, 0.0075 mmol, 0.05 equiv). The reaction vessel was sealed with a Teflon lined cap, then evacuated and flushed with nitrogen three times. Under the atmosphere of nitrogen, 1,2-dichloroethane (1.00 mL) were added *via* syringe. The resulting mixture was then stirred at 120 °C for 24 h. After the reaction was complete, the volatiles were removed *in vacuo* and the residue was purified by column chromatography (*n*-hexane-EtOAc 5:1), yielding **73** as a white solid; yield: 66.6 mg (76%); mp= 145-146 °C. **^{1}H NMR (acetone-d_6, 300 MHz)** δ: 8.55 (d, *J* = 3.9 Hz, 1H), 8.03 (d, *J* = 7.8 Hz, 1H), 7.92 (td, *J* = 7.7, 1.5 Hz, 2H), 7.55 - 7.47 (m, 1H), 7.40 (s, 3H), 7.24 - 7.17 (m, 10H), 7.18 - 7.09 (m, 10H), 6.73 (s, 2H), 3.44 (dd, *J* = 14.3, 6.9 Hz, 2H), 2.77 - 2.69 (m, 2H). **^{13}C NMR ($CDCl_3$, 125 MHz)** δ: 163.7, 150.2, 147.9, 145.4, 143.0, 140.0, 137.3, 137.2, 135.6, 130.9, 130.8, 129.9, 129.5, 128.3, 128.1, 127.4, 126.9, 126.3, 125.8, 122.2, 39.3, 30.7. **ESI$^+$** calcd. for $C_{42}H_{35}N_2O$ $(M)^+$: 583.2743; Found: 583.2754. The structure of this compound was confirmed by X-ray diffraction.

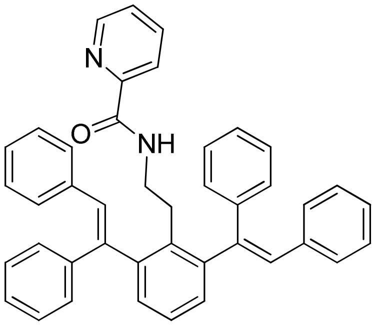

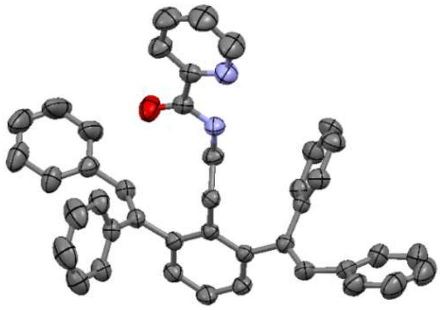

ORTEP view of **67**, hydrogen atoms have been removed for simplicity

***N*-(2,6-Bis((*E*)-1,2-diphenylvinyl)-4-methoxyphenethyl)picolinamide (77).** Compound **77**

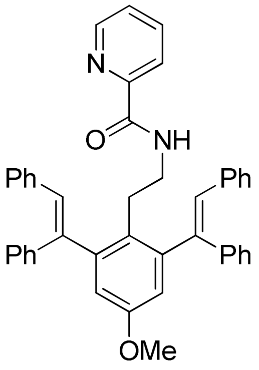

was prepared following the general protocol from *N*-(4-methoxyphenethyl)picolinamide (**62**) (38.4 mg, 0.15 mmol, 1.00 equiv), to give **77** as a white solid; yield: 74 mg (81%); mp= 169-170 °C. **^{1}H NMR ($CDCl_3$, 300 MHz)** δ: 8.46 (d, *J* = 4.1 Hz, 1H), 8.09 (d, *J* = 7.8 Hz, 1H), 7.77 (t, *J* = 7.7 Hz, 1H), 7.58 (s, 1H), 7.42 - 7.30 (m, 1H), 7.21 - 7.06 (m, 20H), 6.95 (s, 2H), 6.71 (s, 2H), 3.88 (s, 3H), 3.26 (dd, *J* = 13.7, 6.6 Hz, 2H), 2.55 (t, *J* = 7.5 Hz, 2H). **^{13}C NMR ($CDCl_3$, 125 MHz)** δ: 163.7, 157.5, 150.3, 148.0, 146.5, 143.0, 139.8, 137.3, 137.2, 130.8, 129.9, 129.5, 128.3, 128.1, 127.8, 127.5, 126.9, 125.9, 122.2, 116.2, 55.5, 39.5, 29.9. **ESI^{+}** calcd. for $C_{43}H_{37}N_2O_2$ $(M+H)^+$: 613.2849; Found: 613.2830.

***N*-(4-Chloro-2,6-bis((*E*)-1,2-diphenylvinyl)phenethyl)picolinamide (78).** Compound **78** was

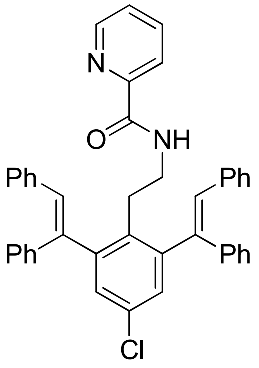

prepared following the general protocol from *N*-(4-chlorophenethyl)picolinamide (**63**) (39.0 mg, 0.15 mmol, 1.00 equiv), to give **78** as a white solid; yield: 77.6 mg (84%); mp= 199-200 °C. **^{1}H NMR ($CDCl_3$, 300 MHz)** δ: 8.47 (d, *J* = 4.2 Hz, 1H), 8.08 (d, *J* = 7.8 Hz, 1H), 7.78 (td, *J* = 7.7, 1.6 Hz, 1H), 7.59 (s, 1H), 7.40 (s, 2H), 7.40 - 7.32 (m, 1H), 7.22 - 7.02 (m, 20H), 6.70 (s, 2H), 3.27 (dd, *J* = 13.8, 6.7 Hz, 2H), 2.59 (t, *J* = 7.5 Hz, 2H). **^{13}C NMR ($CDCl_3$, 75 MHz)** δ: 163.7, 150.1, 148.0, 146.9, 141.8, 139.3, 137.2, 136.9, 134.4, 131.8, 131.6, 130.4, 129.8, 129.5, 128.4, 128.1, 127.7, 127.1, 125.9, 122.2, 39.1, 30.3. **ESI^{+}** calcd. for $C_{42}H_{34}ClN_2O$ $(M+H)^+$: 617.2354; Found: 617.2352.

***N*-(2,6-Bis((*E*)-1,2-diphenylvinyl)-4-fluorophenethyl)picolinamide (79).** Compound **79** was

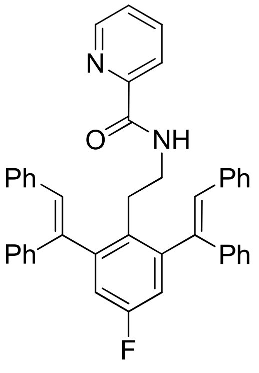

prepared following the general protocol from *N*-(4-fluorophenethyl)picolinamide (**64**) (36.6 mg, 0.15 mmol, 1.00 equiv), to give **79** as a pale yellow solid; yield: 50.2 mg (56%); mp= 204-205 °C. **^{1}H NMR ($CDCl_3$, 300 MHz)** δ: 8.47 (d, *J* = 4.4 Hz, 1H), 8.09 (d, *J* = 7.8 Hz, 1H), 7.82 - 7.73 (m, 1H), 7.59 (s, 1H), 7.37 (dd, *J* = 6.9, 5.2 Hz, 1H), 7.23 - 7.06 (m, 22H), 6.71 (s, 2H), 3.28 (dd, *J* = 13.9, 6.7 Hz, 2H), 2.60 (t, *J* = 7.5 Hz, 2H). **^{13}C NMR ($CDCl_3$, 125 MHz)** δ: 163.7, 160.79 (d, *J* = 246.8 Hz), 150.1, 148.0, 147.1 (d, *J* = 7.5 Hz), 142.0 (d, *J* = 1.3 Hz), 139.4, 137.2, 136.9, 131.5 (d, *J* = 3.2 Hz), 131.4, 129.8, 129.5, 128.4, 128.1, 127.7, 127.1, 125.9, 122.2, 117.36 (d, *J* = 20.5 Hz), 39.2, 30.0. **ESI^{+}** calcd. for $C_{42}H_{34}FN_2O$ $(M+H)^+$: 601.2649; Found: 601.2648.

(*E*)-*N*-(2-(1,2-Diphenylvinyl)-5-methoxyphenethyl)picolinamide (80). Compound **80** was prepared following the general protocol from *N*-(3-methoxyphenethyl)picolinamide (**65**) (38.4 mg, 0.15 mmol, 1.00 equiv), to give **80** as a colorless oil; yield: 40 mg (62%). **^{1}H NMR ($CDCl_3$, 300 MHz)** δ: 8.50 (d, *J* = 4.2 Hz, 1H), 8.16 (d, *J* = 7.8 Hz, 1H), 7.89 (s, 1H), 7.82 (td, *J* = 7.7, 1.6 Hz, 1H), 7.40 (ddd, *J* = 7.5, 4.8, 1.0 Hz, 1H), 7.29 - 7.24 (m, 1H), 7.23 - 7.05 (m, 10H), 6.84 - 6.78 (m, 2H), 6.63 (s, 1H), 3.80 (s, 3H), 3.41 (dd, *J* = 14.0, 6.7 Hz, 2H), 2.75 (t, *J* = 7.3 Hz, 2H). **^{13}C NMR ($CDCl_3$, 75 MHz)** δ: 164.2, 159.3, 150.1, 148.1, 142.4, 140.7, 138.7, 137.4, 137.4, 137.0, 132.1, 130.4, 130.0, 129.5, 128.4, 128.1, 127.4, 126.8, 126.1, 122.3, 115.3, 112.2, 55.4, 40.0, 33.7. **EI$^+$** calcd. for $C_{29}H_{26}N_2O_2$ $(M)^+$: 434.1994; Found: 434.1991.

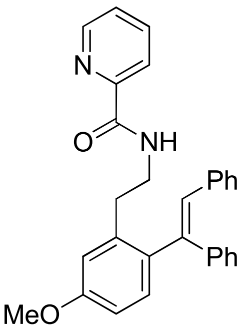

(*E*)-*N*-(2-(1,2-Diphenylvinyl)-5-methylphenethyl)picolinamide (81). Compound **81** was prepared following the general protocol from *N*-(3-methylphenethyl)picolinamide (**66**) (36.0 mg, 0.15 mmol, 1.00 equiv), to give **81** as a pale yellow oil; yield: 20.1 mg (32%). **^{1}H NMR ($CDCl_3$, 300 MHz)** δ: 8.42 (d, *J* = 4.6 Hz, 1H), 8.07 (d, *J* = 7.8 Hz, 1H), 7.80 (s, 1H), 7.73 (t, *J* = 7.7 Hz, 1H), 7.34 - 7.27 (m, 1H), 7.19 - 6.97 (m, 13H), 6.56 (s, 1H), 3.31 (dd, *J* = 14.1, 6.7 Hz, 2H), 2.67 (t, *J* = 7.4 Hz, 2H), 2.26 (s, 3H). **^{13}C NMR ($CDCl_3$, 75 MHz)** δ: 164.1, 150.1, 148.1, 142.7, 141.5, 140.6, 137.6, 137.4, 137.3, 136.9, 131.1, 130.9, 130.4, 130.0, 129.5, 128.4, 128.1, 127.4, 127.3, 126.9, 126.1, 122.2, 40.2, 33.4, 21.2. **EI$^+$** calcd. for $C_{29}H_{26}N_2O$ $(M)^+$: 418.2045; Found: 418.2049.

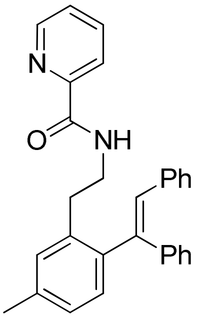

(*E*)-*N*-(2-(1,2-Diphenylvinyl)-6-methoxyphenethyl)picolinamide (82). Compound **82** was prepared following the general protocol from *N*-(2-methoxyphenethyl)picolinamide (**67**) (38.4 mg, 0.15 mmol, 1.00 equiv), to give **82** as a colorless oil; yield: 57 mg (87%). **^{1}H NMR ($CDCl_3$, 300 MHz)** δ: 8.51 (d, *J* = 4.8 Hz, 1H), 8.17 (s, 1H), 8.14 (d, *J* = 7.8 Hz, 1H), 7.80 (td, *J* = 7.7, 1.7 Hz, 1H), 7.38 (ddd, *J* = 7.6, 4.8, 1.2 Hz, 1H), 7.27 - 7.24 (m, 1H), 7.22 - 7.18 (m, 5H), 7.15 (d, *J* = 2.1 Hz, 1H), 7.14 (d, *J* = 1.9 Hz, 2H), 7.10 - 7.06 (m, 2H), 6.98 (dd, *J* = 7.6, 1.1 Hz, 1H), 6.87 (dd, *J* = 8.3, 0.9 Hz, 1H), 6.64 (s, 1H), 3.88 (s, 3H), 3.34 (dd, *J* = 12.6, 6.8 Hz, 2H), 2.92 (t, *J* = 6.9 Hz, 2H). **^{13}C NMR ($CDCl_3$, 75 MHz)** δ: 164.1, 158.1, 150.4, 148.0, 145.9, 142.5, 140.4, 137.3, 130.6, 129.9, 129.5, 128.4, 128.1, 127.5, 127.2, 126.9, 126.0, 125.9, 123.3, 122.2, 109.5, 55.6, 39.5, 27.1. **EI$^+$** calcd. for $C_{29}H_{26}N_2O_2$ $(M)^+$: 434.1994; Found: 434.2005.

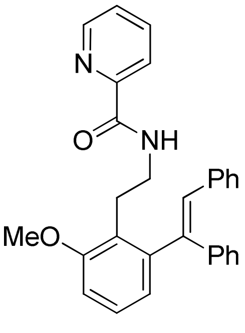

(*E*)-*N*-(2-(1,2-Diphenylvinyl)-6-methylphenethyl)picolinamide (83). Compound **83** was prepared following the general protocol from *N*-(2-methylphenethyl)picolinamide (**68**) (36.0 mg, 0.15 mmol, 1.00 equiv), to give **83** as a yellow oil; yield: 50 mg (79%). **^{1}H NMR ($CDCl_3$, 300 MHz)** δ: 8.52 (d, *J* = 4.8 Hz, 1H), 8.16 (d, *J* = 7.8 Hz, 1H), 7.88 (s, 1H), 7.83 (td, *J* = 7.7, 1.7 Hz, 1H), 7.41 (ddd, *J* = 7.6, 4.8, 1.2 Hz, 1H), 7.25 - 7.08 (m, 13H), 6.66 (s, 1H), 3.16 (dd, *J* = 15.8, 6.3 Hz, 2H), 2.93 - 2.82 (m, 2H), 2.45 (s, 3H). **^{13}C NMR ($CDCl_3$, 75 MHz)** δ: 164.0, 150.0, 147.9, 144.9, 143.5, 140.6, 137.9, 137.6, 137.3, 135.3, 130.4, 130.2, 130.0, 129.5, 128.8, 128.4, 128.1, 127.5, 126.9, 126.4, 126.1, 122.4, 38.8, 31.1, 19.9. **EI$^+$** calcd. for

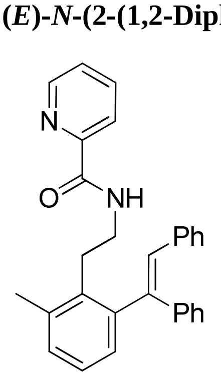

$C_{29}H_{26}N_2O$ (M)$^+$: 418.2045; Found: 418.2041.

(*E*)-*N*-(2-Bromo-6-(1,2-diphenylvinyl)phenethyl)picolinamide (84). Compound **84** was prepared following the general protocol from *N*-(2-bromophenethyl)picolinamide (**69**) (45.6 mg, 0.15 mmol, 1.00 equiv), to give **84** as a yellow oil; yield: 50.0 mg (69%). **^{1}H NMR ($CDCl_3$, 300 MHz)** δ: 8.39 (d, *J* = 4.6 Hz, 1H), 8.02 (d, *J* = 7.8 Hz, 1H), 7.74 (s, 1H), 7.68 (td, *J* = 7.7, 1.6 Hz, 1H), 7.44 (d, *J* = 6.9 Hz, 1H), 7.33 - 7.24 (m, 1H), 7.22 (d, *J* = 7.5 Hz, 1H), 7.13 - 6.99 (m, 9H), 6.97 - 6.90 (m, 2H), 6.52 (s, 1H), 3.25 (dd, *J* = 14.1, 6.9 Hz, 2H), 2.94 - 2.85 (m, 2H). **^{13}C NMR ($CDCl_3$, 75 MHz)** δ: 164.1, 150.1, 148.0, 146.6, 142.3, 139.8, 137.3, 136.9, 136.8, 132.7, 131.2, 130.3, 129.9, 129.5, 128.5, 128.1, 127.9, 127.8, 127.1, 126.3, 126.0, 122.3, 38.4, 33.8. **ESI$^+$** calcd. for $C_{28}H_{24}BrN_2O$ (M+H)$^+$: 483.1066; Found: 483.1064.

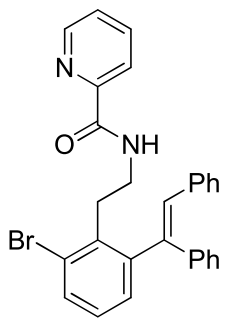

(*E*)-*N*-(2-Chloro-6-(1,2-diphenylvinyl)phenethyl)picolinamide (85). Compound **85** was prepared following the general protocol from *N*-(2-chlorophenethyl)picolinamide (**70**) (39.01 mg, 0.15 mmol, 1.00 equiv), to give **85** as a colorless oil; yield: 60 mg (92%). **^{1}H NMR ($CDCl_3$, 300 MHz)** δ: 8.41 (d, *J* = 4.6 Hz, 1H), 8.04 (d, *J* = 7.8 Hz, 1H), 7.76 (s, 1H), 7.70 (td, *J* = 7.8, 1.5 Hz, 1H), 7.35- 7.23 (s, 1H), 7.22 - 7.00 (m, 10H), 6.98 - 6.92 (m, 2H), 6.55 (s, 1H), 3.27 (dd, *J* = 14.4, 6.6 Hz, 2H), 2.93 - 2.86 (m, 2H). **^{13}C NMR ($CDCl_3$, 75 MHz)** δ: 164.1, 150.2, 148.1, 146.6, 142.1, 139.8, 137.3, 136.9, 135.6, 135.2, 131.2, 129.9, 129.6, 129.5, 129.2, 128.5, 128.1, 127.8, 127.6, 127.1, 126.0, 122.3, 38.4, 31.3. **EI$^+$** calcd. for $C_{28}H_{23}ClN_2O$ (M)$^+$: 438.1499; Found: 438.1494.

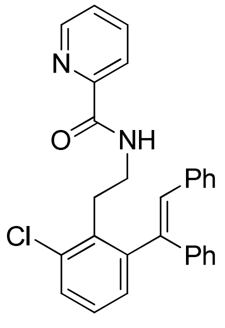

(*E*)-*N*-(2-(3-(1,2-Diphenylvinyl)naphthalen-2-yl)ethyl)picolinamide (86). Compound **86** was prepared following the general protocol from *N*-(2-(naphthalen-2-yl)ethyl)picolinamide (**71**) (41.4 mg, 0.15 mmol, 1.00 equiv), to give **86** as a yellow oil; yield: 28 mg (42%). **^{1}H NMR ($CDCl_3$, 300 MHz)** δ: 8.48 (d, *J* = 4.2 Hz, 1H), 8.17 (d, *J* = 7.8 Hz, 1H), 7.93 - 7.76 (m, 5H), 7.72 (s, 1H), 7.48 (dd, *J* = 6.0, 3.3 Hz, 2H), 7.42 - 7.34 (m, 1H), 7.25 - 7.13 (m, 10H), 6.81 (s, 1H), 3.46 (dd, *J* = 13.8, 6.8 Hz, 2H), 2.85 (t, *J* = 7.2 Hz, 2H). **^{13}C NMR ($CDCl_3$, 75 MHz)** δ: 164.2, 150.1, 148.1, 142.9, 142.7, 140.0, 137.3, 137.3, 135.6, 133.2, 132.3, 130.8, 130.1, 129.8, 129.6, 128.9, 128.5, 128.2, 127.7, 127.7, 127.4, 127.1, 126.2, 126.1, 125.9, 122.3, 40.0, 33.7. **ESI$^+$** calcd. for $C_{32}H_{26}N_2O$ (M+H)$^+$: 455.2123; Found: 455.2117.

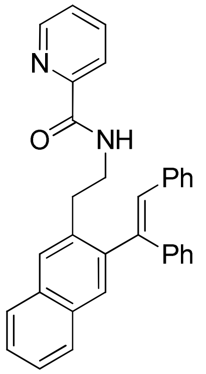

(*E*)-*N*-(2-(3-(1,2-Diphenylvinyl)thiophen-2-yl)ethyl)picolinamide (87). Compound **87** was prepared following the general protocol from *N*-(2-(thiophen-2-yl)ethyl)picolinamide (**72**) (34.8 mg, 0.15 mmol, 1.00 equiv), to give **87** as a yellow oil; yield: 59 mg (97%). **^{1}H NMR ($CDCl_3$, 300 MHz)** δ: 8.53 (d, *J* = 4.3 Hz, 1H), 8.18 (d, *J* = 7.8 Hz, 1H), 8.12 (s, 1H), 7.83 (td, *J* = 7.7, 1.5 Hz, 1H), 7.43 - 7.38 (m, 1H), 7.25 - 7.16 (m, 5H), 7.14 - 7.09 (m, 4H), 7.07 - 7.01 (m, 2H), 6.83 (d, *J* = 5.2 Hz, 1H), 6.69 (s, 1H), 3.66 (q, *J* = 6.7 Hz, 2H), 3.03 (t, *J* = 6.9 Hz, 2H). **^{13}C NMR ($CDCl_3$, 75 MHz)** δ: 164.3, 150.0, 148.1, 142.1, 140.4, 137.6, 137.4, 137.4, 137.1, 130.1, 129.9, 129.9, 129.5, 128.5, 128.1, 127.5, 126.9, 126.2, 122.3, 40.9, 28.7. **EI$^+$** calcd. for $C_{26}H_{22}N_2OS$ (M+H)$^+$: 411.1531; Found: 411.1526.

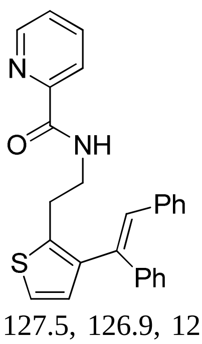

10.2. Scope with regard to the alkyne

***N*-(2,6-Bis((*E*)-1,2-bis(4-methoxyphenyl)vinyl)phenethyl)picolinamide (74).** Compound **74** was prepared following the general protocol from 1,2-bis(4-methoxy-phenyl)ethyne (**I**) (71.4 mg, 0.30 mmol, 2.00 equiv) to give **74** as a white solid; yield: 86.4 mg (82%); mp= 92-93 °C. **^{1}H NMR ($CDCl_3$, 300 MHz)** δ: 8.41 (d, *J* = 4.6 Hz, 1H), 8.03 (d, *J* = 7.8 Hz, 1H), 7.72 (t, *J* = 7.7 Hz, 1H), 7.54 (s, 1H), 7.35 - 7.16 (m, 3H), 7.02 (dd, *J* = 18.9, 8.6 Hz, 8H), 6.65 (t, *J* = 8.2 Hz, 8H), 6.50 (s, 2H), 3.71 (s, 6H), 3.69 (s, 6H), 3.27 (dd, *J* = 12.6, 6.0 Hz, 2H), 2.58 (t, *J* = 7.5 Hz, 2H). **^{13}C NMR ($CDCl_3$, 75 MHz)** δ: 163.7, 158.7, 158.4, 150.3, 147.9, 145.7, 141.0, 137.2, 135.6, 132.7, 131.1, 130.7, 130.6, 130.3, 129.4, 126.2, 125.8, 122.2, 113.7, 113.5, 55.3, 55.2, 39.4, 30.6. **ESI$^+$** calcd. for $C_{46}H_{43}N_2O_5$ $(M)^+$: 703.3166; Found: 703.3172.

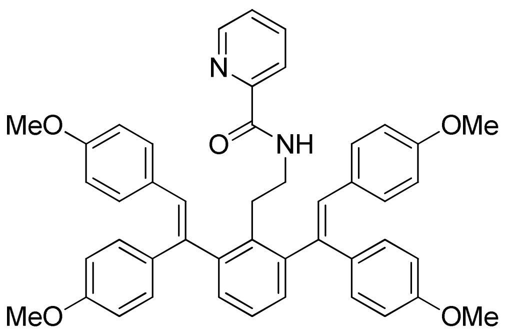

***N*-(2,6-Bis((*E*)-1,2-di-*p*-tolylvinyl)phenethyl)picolinamide (75).** Compound **75** was prepared following the general protocol from 1,2-di-*p*-tolylethyne (**III**) (61.8 mg, 0.30 mmol, 2.00 equiv) to give **75** as a pale yellow solid; yield: 80 mg (84%); mp= 85-86 °C. **^{1}H NMR ($CDCl_3$, 300 MHz)** δ: 8.39 (d, *J* = 4.2 Hz, 1H), 8.02 (d, *J* = 7.8 Hz, 1H), 7.71 (t, *J* = 7.7 Hz, 1H), 7.48 (s, 1H), 7.48 - 7.33 (m, 4H), 7.01 (d, *J* = 7.8 Hz, 4H), 6.95 - 6.84 (m, 12H), 6.54 (s, 2H), 3.25 (dd, *J* = 13.2, 6.2 Hz, 2H), 2.58 (t, *J* = 7.5 Hz, 2H), 2.21 (s, 12H). **^{13}C NMR ($CDCl_3$, 75 MHz)** δ: 163.6, 150.3, 147.9, 145.6, 142.2, 137.2, 137.1, 137.0, 136.5, 135.6, 134.7, 130.6, 130.3, 129.8, 129.4, 129.0, 128.8, 126.1, 125.8, 122.2, 39.4, 30.6, 21.3, 21.3. **ESI$^+$** calcd. for $C_{46}H_{43}N_2O$ $(M)^+$: 639.3369; Found: 639.3367.

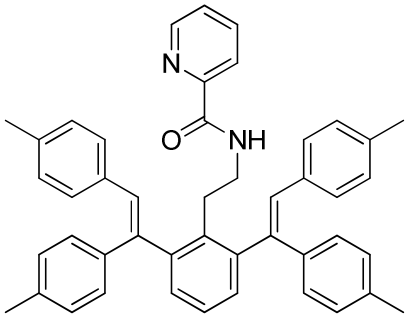

***N*-(2,6-Bis((*E*)-1,2-bis(4-(trifluoromethyl)phenyl)vinyl)phenethyl)picolinamide (76).** Compound **76** was prepared following the general protocol from 1,2-bis(4-(trifluoromethyl)phenyl)ethyne (**IV**) (94.2 mg, 0.30 mmol, 2.00 equiv), to give **76** as a pale yellow solid; yield: 55 mg (43%); mp= 86-88 °C. **^{1}H NMR ($CDCl_3$, 300 MHz)** δ: 8.46 (d, *J* = 4.2 Hz, 1H), 8.07 (d, *J* = 7.8 Hz, 1H), 7.80 (td, *J* = 7.7, 1.7 Hz, 1H), 7.63 (s, 1H), 7.50 - 7.38 (m, 12H), 7.27 (d, *J* = 8.0 Hz, 5H), 7.17 (d, *J* = 8.2 Hz, 3H), 6.81 (s, 2H), 3.36 (dd, *J* = 14.3, 6.8 Hz, 2H), 2.58 (t, *J* = 7.6 Hz, 2H). **^{13}C NMR ($CDCl_3$, 75 MHz)** δ: 163.9, 149.8, 148.0, 144.3, 143.4, 142.9 (d, *J* = 1.3 Hz), 140.1 (d, *J* = 1.3 Hz), 137.4, 135.4, 131.3 (d, *J* = 2.5 Hz), 130.1 (s, *J* = 5.6 Hz), 130.0 (d, *J* = 32.5 Hz), 129.7 (s, *J* = 5.7 Hz), 129.3 (d, *J* = 32.5 Hz), 127.0, 126.2, 125.9, 125.8, 125.6 (q, *J* = 3.7 Hz), 125.39 (q, *J* = 3.7 Hz), 124.17 (d, *J* = 272.1 Hz), 124.03 (d, *J* = 272.2 Hz), 122.3, 39.4, 31.1. **ESI$^+$** calcd. for $C_{46}H_{31}F_{12}N_2O$ $(M)^+$: 855.2239; Found: 855.2254.

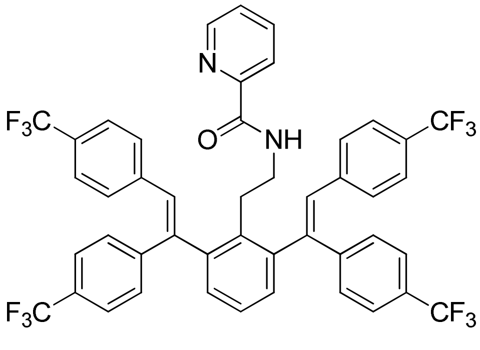

11. Typical procedure for the cleavage of the benzyl group (Scheme 8)

Synthesis of 5,6,7,8-tetraphenylisoquinoline-1-carboxamide (82)

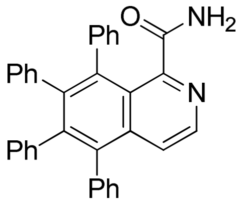

An oven-dried, argon flushed 10 mL microwave vessel was charged with *N*-benzyl-5,6,7,8-tetraphenylisoquinoline-1-carboxamide (**2**) (30.0 mg, 0.05 mmol, 1.00 equiv) and then sealed with a Teflon lined cap, evacuated and flushed with argon three times. Under the atmosphere of argon, toluene (1.00 mL) and triflic acid (106 µL, 1.20 mmol, 4.00 equiv) were added *via* syringe. The resulting solution was then stirred for 5 min at room temperature followed by microwave irradiation at 150 °C for 1 h. Removal of solvent *in vacuo* gave the crude product as a brown solid that was diluted with 15 mL of CH_2Cl_2 and and washed with water (2 × 20 mL). The organic phases were combined and concentrated under reduced pressure. The residue was purified by column chromatography (*n*-hexane-EtOAc 1:1 with 10% of MeOH), yielding **82** as a yellow solid; yield: 19.5 mg (82%). **^{1}H NMR (acetone-d$_6$, 300 MHz)** δ: 8.36 (d, *J* = 5.4 Hz, 1H), 7.36 (d, *J* = 5.7 Hz, 1H), 7.31 - 7.18 (s, 6H), 7.16 - 7.00 (m, 7H), 6.95 - 6.78 (m, 11H), 6.15 (s, 1H). **^{13}C NMR (acetone-d$_6$, 75 MHz)** δ: 144.1, 142.8, 141.6, 140.7, 140.6, 139.7, 139.3, 139.1, 138.4, 137.2, 133.4, 131.9, 131.9, 131.5, 128.6, 128.2, 127.8, 127.4, 127.3, 127.3, 126.6, 126.3, 123.9, 120.8, 119.7, 115.5. **ESI$^+$** calcd. for $C_{34}H_{25}N_2O$ $(M+H)^+$: 477.1961; Found: 477.1969.

12. Typical procedure for the cleavage of the picolinate group (Scheme 8)

Synthesis of (2,6-bis((*E*)-1,2-diphenylvinyl)phenyl)methanamine (90). A 20 mL vessel was charged with *N*-(2,6-bis((*E*)-1,2-diphenylvinyl))picolinamide (**3**) (56.8 mg, 0.10 mmol, 1.00 equiv) and KOH (336 mg, 6.00 mmol, 60.0 equiv). The reaction vessel was sealed with a Teflon lined cap, and ethanol (3.00 mL) was added *via* syringe. The resulting mixture was stirred at 125 °C for 24-48 h. After the reaction was complete, the reaction mixture was cooled down to room temperature, diluted by 50 mL of ethyl acetate and washed with water (2 × 20 mL). The organic layer was dried over $MgSO_4$ and concentrated *in vacuo* to give **90** as a yellow solid; yield: 40 mg (86%); mp= 137-138 °C. **^{1}H NMR (acetone-d$_6$, 300 MHz)** δ: 7.28 - 7.20 (m, 5H), 7.20 - 7.05 (m, 7H), 6.70 (s, 2H), 4.54 (s, 2H). **^{13}C NMR (acetone-d$_6$, 75 MHz)** δ: 146.6, 143.0, 141.9, 138.4, 131.8, 130.7, 130.1, 130.0, 129.0, 128.8, 128.0, 127.5, 126.9, 51.7. **ESI$^+$** calcd. for $C_{35}H_{30}N$ $(M+H)^+$: 464.2372; Found: 464.2381.

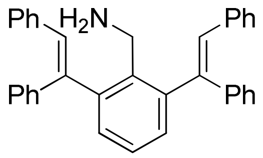

Synthesis of 2-(2,6-bis((*E*)-1,2-diphenylvinyl)phenyl)ethanamine (91). A 20 mL vessel was charged with *N*-(2,6-bis((*E*)-1,2-diphenylvinyl)phenethyl)picolinamide (**73**) (58.2 mg, 0.10 mmol, 1.00 equiv) and NaOH (336 mg, 6.00 mmol, 60.0 equiv). The reaction vessel was sealed with a Teflon lined cap, and ethanol (3.00 mL) was added *via* syringe. The resulting mixture was stirred at 125 °C for 24-48 h. After the reaction was complete, the reaction mixture was cooled down to room temperature, diluted by 50 mL of ethyl acetate and washed with water (2 × 20 mL). The organic layer was dried over $MgSO_4$ and concentrated *in vacuo* to give **91** as a pale yellow oil; yield: 43 mg (89%). **^{1}H NMR (acetone-d$_6$, 300 MHz)** δ: 7.40 - 7.27 (m, 4H), 7.24 - 7.11 (m, 21H), 6.68 (s, 2H), 3.12 (dd, *J* = 9.9, 6.4 Hz, 2H), 2.82 (dd, *J* = 10.0, 6.5 Hz, 2H). **^{13}C NMR (acetone-d$_6$, 75 MHz)** δ: 146.2, 144.0, 141.1, 138.2, 137.5, 131.2, 131.0, 130.5, 130.1, 129.0, 128.8, 128.1, 127.7, 126.6, 52.7, 33.0. **ESI$^+$** calcd. for $C_{36}H_{32}N$ $(M+H)^+$: 478.2529; Found: 478.2539.

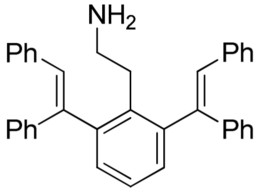

13. Synthesis of the Rh^{III}-complex A[6]

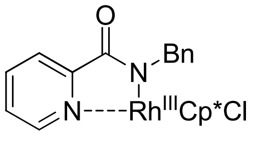

An oven-dried, nitrogen-flushed 20 mL vessel was charged with *N*-benzylpicolinamide (**1**) (21.2 mg, 0.10 mmol, 1.00 equiv), pentamethyl-cyclopentadienylrhodium(III) chloride dimer (30.5 mg, 0.05 mmol, 0.50 equiv), sodium acetate (32.8 mg, 0.40 mmol, 4.00 equiv). The reaction vessel was sealed with a Teflon lined cap, then evacuated and flushed with nitrogen three times. Under the atmosphere of nitrogen, CH_2Cl_2 (10.0 mL) was added *via* syringe and the mixture was left stirring for 16 h at room temperature. After that the resulting mixture was filtered through Celite® and the volatiles were partially removed *in vacuo* until observing the formation by *n*-hexane addition of an orange solid that it was characterized as the Rh^{III}-complex **A**. **^{1}H NMR (CDCl$_3$, 300 MHz)** δ: 8.61 (d, *J* = 5.4 Hz, 1H, py-H^6), 8.09 (d, *J* = 7.8 Hz, 1H, py-H^3), 7.90 (t, *J* = 7.7 Hz, 1H, py-H^4), 7.48 (d, *J* = 7.3 Hz, 3H), 7.30 - 7.21 (m, 2H), 7.14 (t, *J* = 7.3 Hz, 1H), 4.97 (q, *J* = 15.2 Hz, 2H), 1.59 (s, 15H).**^{13}C NMR (acetone-d$_6$, 75 MHz)** δ: 170.3 (C=O), 157.1 (py-C^1), 151.3 (py-C^6), 143.6, 139.4 (py-C^3), 128.7, 128.2, 127.4 (py-C^5), 126.2, 125.4 (py-C^2), 95.3 (d, *J* = 8.0 Hz), 54.8, 9.2. This compound was also characterized by X-ray diffraction.

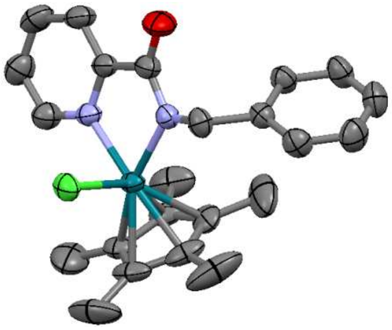

ORTEP view of Rh^{III}-complex **A**, hydrogen atoms have been removed for simplicity

---

[6] A. M. Martínez, N. Rodríguez, R. Gómez Arrayás and J. C. Carretero, *Chem. Commun.*, 2014, **50**, 6105.

14. Synthesis of the Rh^I-complex B[1b]

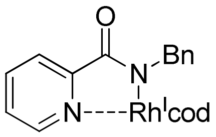

An oven-dried, nitrogen-flushed 20 mL vessel was charged with *N*-benzylpicolinamide (**1**) (106 mg, 0.50 mmol, 1.00 equiv), and chloro(1,5.cyclooctadiene)rhodium(I) dimer (123 mg, 0.25 mmol, 0.50 equiv). The reaction vessel was sealed with a Teflon lined cap, then evacuated and flushed with nitrogen three times. Under the atmosphere of nitrogen, CH_2Cl_2 (5.0 mL) was added *via* syringe and then a solution of KOH (56.1 mg, 1.00 mmol, 2.00 equiv) in EtOH (3.0 mL) was added. After stirring for 10 min at room temperature, the resulting mixture was filtered through Celite® and the volatiles were partially removed *in vacuo* until observing the formation of an orange solid by *n*-hexane addition. This solid was characterized as the Rh^I-complex **B**. **^{1}H NMR (acetone-d$_6$, 300 MHz)** δ: 8.11 (t, *J* = 7.1 Hz, 1H, py-H^4), 7.96 (d, *J* = 7.5 Hz, 1H, py-H^3), 7.86 (d, *J* = 5.3 Hz, 1H, py-H^6), 7.60 (t, *J* = 5.9 Hz, 1H, py-H^5), 7.33 (d, *J* = 7.4 Hz, 2H), 7.24 (t, *J* = 7.5 Hz, 2H), 7.14 (q, *J* = 7.1 Hz, 1H), 4.30 (s, 2H), 4.21 (d, *J* = 2.7 Hz, 2H), 4.02 (d, *J* = 2.7 Hz, 2H), 2.50 - 2.31 (m, 4H), 1.91 (d, *J* = 8.7 Hz, 4H). **^{13}C NMR (acetone-d$_6$, 125 MHz)** δ: 173.4 (d, *J* = 1.4 Hz, C=O), 157.1 (py-C^1), 146.7 (py-C^6), 143.9, 140.9 (py-C^3), 128.6, 127.5 (py-C^5), 127.4, 126.4 (py-C^2), 125.3, 83.9 (d, *J* = 12.9 Hz), 78.5 (d, *J* = 12.0 Hz), 47.7 (d, *J* = 1.5 Hz), 31.6, 30.8. For the X-ray diffraction studies, the orange solid was dissolved in toluene. Pentane was added to form the upper layer. Then the vessel was kept under refrigeration for 12 h. The obtained crystals were suitable for the characterization by X-ray diffraction.

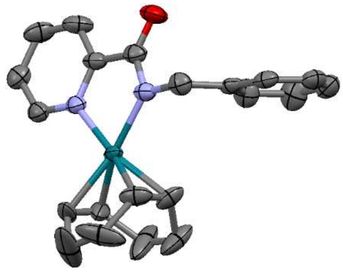

ORTEP view of Rh^I-complex **B**, hydrogen atoms have been removed for simplicity

15. Synthesis of the Rh^I-complex M

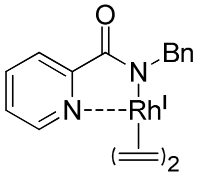

An oven-dried, nitrogen-flushed 20 mL vessel was charged with *N*-benzylpicolinamide (**1**) (106 mg, 0.50 mmol, 1.00 equiv), and acetylacetonatobis(ethylene)rhodium(I) (129 mg, 0.25 mmol, 0.50 equiv). The reaction vessel was sealed with a Teflon lined cap, then evacuated and flushed with nitrogen three times. Under the atmosphere of nitrogen, CH_2Cl_2 (5.0 mL) and a solution of KOH (56.1 mg, 1.00 mmol, 2.00 equiv) in EtOH (3.0 mL) were added *via* syringe. After stirring for 10 min at room temperature, the volatiles were partially removed *in vacuo* until observing the formation of an orange solid by dropwise *n*-hexane addition. Then the remaining solvent was evacuate under inert atmosphere and the resulting solid was totally dried *in vacuo*. This Rh^I-complex **M** was quickly characterized due to its moderate stability. The NMR tube was prepared under nitrogen atmosphere. **^{1}H NMR ($CDCl_3$, 300 MHz)** δ: 8.09 (d, *J* = 7.8 Hz, 1H), 7.95 (td, *J* = 7.7, 1.4 Hz, 1H), 7.63 (d, *J* = 5.4 Hz, 1H), 7.50 - 7.44 (m, 1H), 7.34 (d, *J* = 7.5 Hz, 2H), 7.29 - 7.22 (m, 2H), 7.15 (t, *J* = 7.1 Hz, 1H), 4.33 (s, 2H), 3.21 (s, 6H), 2.46 (s, 2H). **^{13}C NMR ($CDCl_3$, 75 MHz)** δ: 174.3 (d, *J* = 1.4 Hz), 174.3, 156.5, 142.4, 142.1, 140.1, 128.3, 126.7, 126.5, 126.0, 125.3, 61.5 (d, *J* = 11.8 Hz), 45.8 (d, *J* = 1.5 Hz). **ESI$^+$** calcd. for $C_{17}H_{20}N_2ORh$ $(M+H)^+$: 371.0625; Found: 371.0621.

16. Mechanistic studies

16.1 Stoichiometric studies with the isolated Rh^{III} and Rh^{I} picolinamide complexes

16.1.1. Using the Rh^{III}-complex A

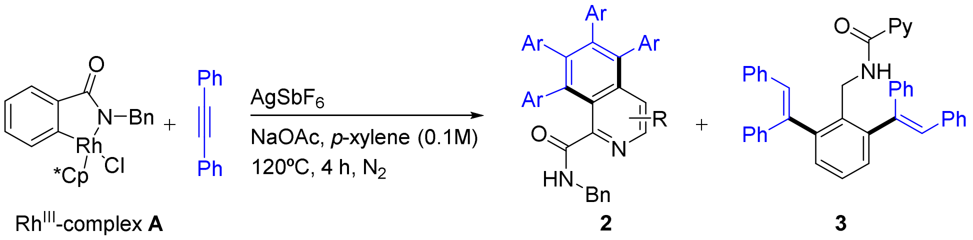

General procedure. An oven-dried, nitrogen-flushed 20 mL vessel was charged with Rh^{III}-complex **A** (24.3 mg, 0.05 mmol, 1.00 equiv), diphenylacetylene (17.8 mg, 0.10 mmol, 2.00 equiv), sodium acetate (16.4 mg, 0.20 mmol, 4.00 equiv), and silver hexafluoroantimonate(V) (17.2 mg, 0.05 mmol, 1.00 equiv). The reaction vessel was sealed with a Teflon lined cap, then evacuated and flushed with nitrogen three times. Under the atmosphere of nitrogen, *p*-xylene (1.00 mL) was added *via* syringe. The resulting mixture was then stirred at 120 °C for 4 h. After the reaction was complete, the volatiles were removed *in vacuo* and the residue was analysed by ^{1}HNMR, yielding **2** and **3** in a 70% and 30% respectively.

16.1.2. Using the Rh^{I}-complex B

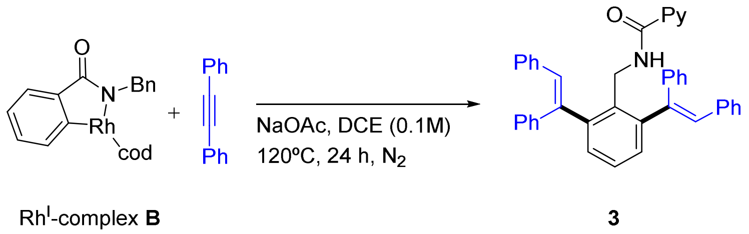

General procedure. An oven-dried, nitrogen-flushed 20 mL vessel was charged with Rh^{I}-complex **B** (21.1 mg, 0.05 mmol, 1.00 equiv), diphenylacetylene (17.8 mg, 0.10 mmol, 2.00 equiv) and sodium acetate (16.4 mg, 0.20 mmol, 4.00 equiv). The reaction vessel was sealed with a Teflon lined cap, then evacuated and flushed with nitrogen three times. Under the atmosphere of nitrogen, 1,2-dichloroethane (1.00 mL) was added *via* syringe. The resulting mixture was then stirred at 120 °C for 24 h. After the reaction was complete, the volatiles were removed *in vacuo* and the residue was analysed by ^{1}HNMR, yielding **3** in a 79% (**2** was not observed).

16.2. H/D exchange experiments using D_2O as deuterium donor

16.2.1. Rh^{III}-catalyzed C–H functionalization process

16.2.1.1. Standard reaction

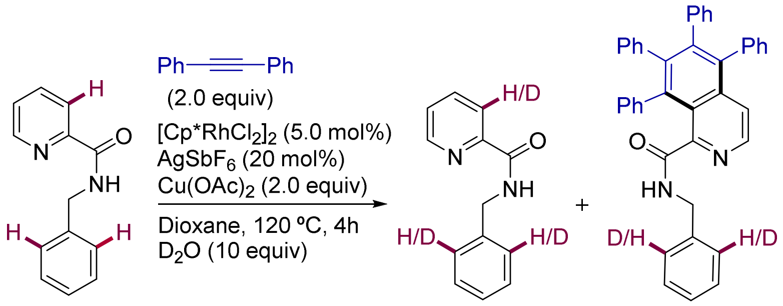

1 1-D[1], 55% recovery 2-D, 39%

50% H/D scrambling in C_3-Py

46% H/D scrambling in the Bn

General procedure. An oven-dried, nitrogen-flushed 20 mL vessel was charged with *N*-benzylpicolinamide (**1**) (31.8 mg, 0.15 mmol, 1.00 equiv), diphenylacetylene (53.3 mg, 0.30 mmol, 2.00 equiv), pentamethyl-cyclopentadienylrhodium(III) chloride dimer (4.64 mg, 0.0075 mmol, 0.05 equiv), copper (II) acetate (54.5 mg, 0.30 mmol, 2.00 equiv) and silver hexafluoroantimonate(V) (10.7 mg, 0.03 mmol, 0.20 equiv). The reaction vessel was sealed with a Teflon lined cap, then evacuated and flushed with nitrogen three times. Under the atmosphere of nitrogen, 1,4-dioxane (1.00 mL) and deuterium oxide (27.1 µL, 10.0 equiv) were added *via* syringe. The resulting mixture was then stirred at 120 °C for 4 h. After the reaction was complete, the volatiles were removed *in vacuo* and the residue was purified by column chromatography (*n*-hexane-EtOAc 2:1), obtaining **1-D[1]** in 55% yield (19 mg) and **2-D** in 39% yield (33 mg).

In the spectrum of **1-D[1]**, the integration of the doublet at 8.17-8.14 ppm (py-H^3) was 0.48 instead of 0.96 (50% H/D scrambling) and the integration of the doublet at 7.42-7.39 ppm (corresponding to the *o*-benzyl positions) was 1.09 instead of 2.00 (46% H/D scrambling).

The deuteration percentage of **2-D** could not be determined by ^{1}H NMR, being analyzed by mass spectrometry instead.

Spectra of 1 and 1-D[1]

^{1}H NMR (acetone-d_6, 300 MHz)

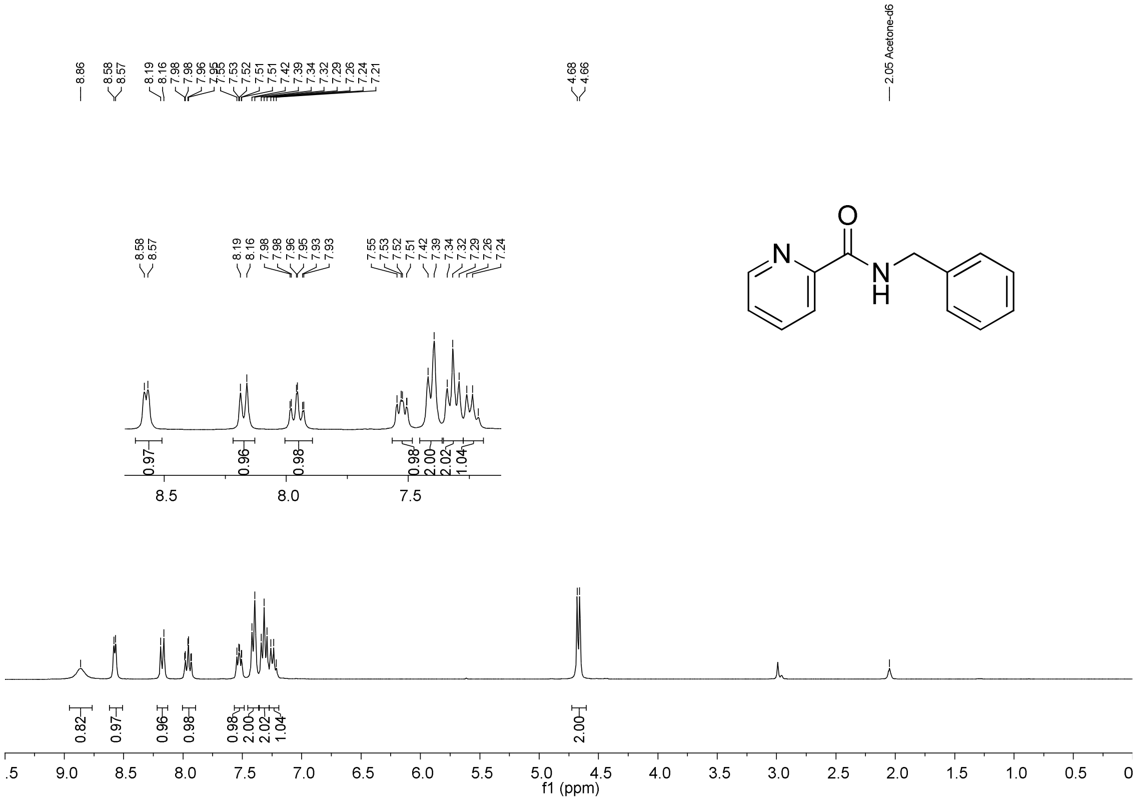

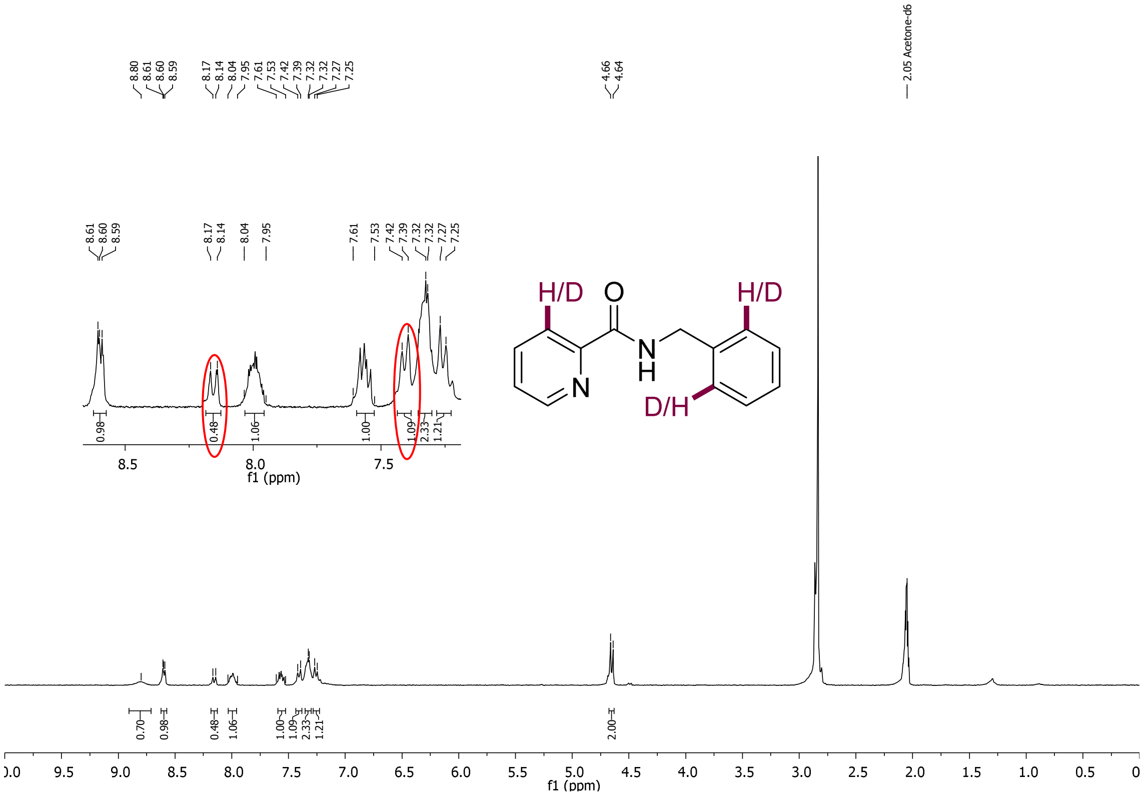

16.2.1.2. Standard reaction from Rh^{III}-complex A

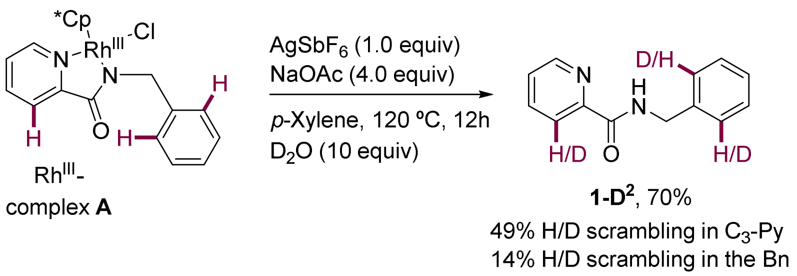

An oven-dried, nitrogen-flushed 20 mL vessel was charged with Rh^{III}-complex **A** (12.1 mg, 0.025 mmol, 1.00 equiv), silver hexafluoroantimonate(V) (8.59 mg, 0.025 mmol, 1.00 equiv) and sodium acetate (8.20 mg, 0.10 mmol, 4.00 equiv). The reaction vessel was sealed with a Teflon lined cap, then evacuated and flushed with nitrogen three times. Under the atmosphere of nitrogen, *p*-xylene (1.00 mL) and deuterium oxide (4.52 µL, 10.0 equiv) were added *via* syringe. The resulting mixture was then stirred at 120 °C for 12 h. After the reaction was complete, 2.00 mL of D_2O and 2.00 mL of CH_2Cl_2 were added and the organic layer was filtered through Celite®. Then the volatiles were removed *in vacuo* and the residue was purified by column chromatography (*n*-hexane-EtOAc 5:1), obtaining $\mathbf{1\text{-}D^2}$ in 70% yield (3.90 mg).

In the spectrum of $\mathbf{1\text{-}D^2}$, the integration of the doublet at 8.17-8.14 ppm (py-H^3) was 0.51 instead of 0.96 (49% H/D scrambling) and the integration of the doublet at 7.42-7.39 ppm (corresponding to the *o*-benzyl positions) was 1.72 instead of 2.00 (14% H/D scrambling).

Spectra of 1 and 1-D[2]

^{1}H NMR (acetone-d$_6$, 300 MHz)

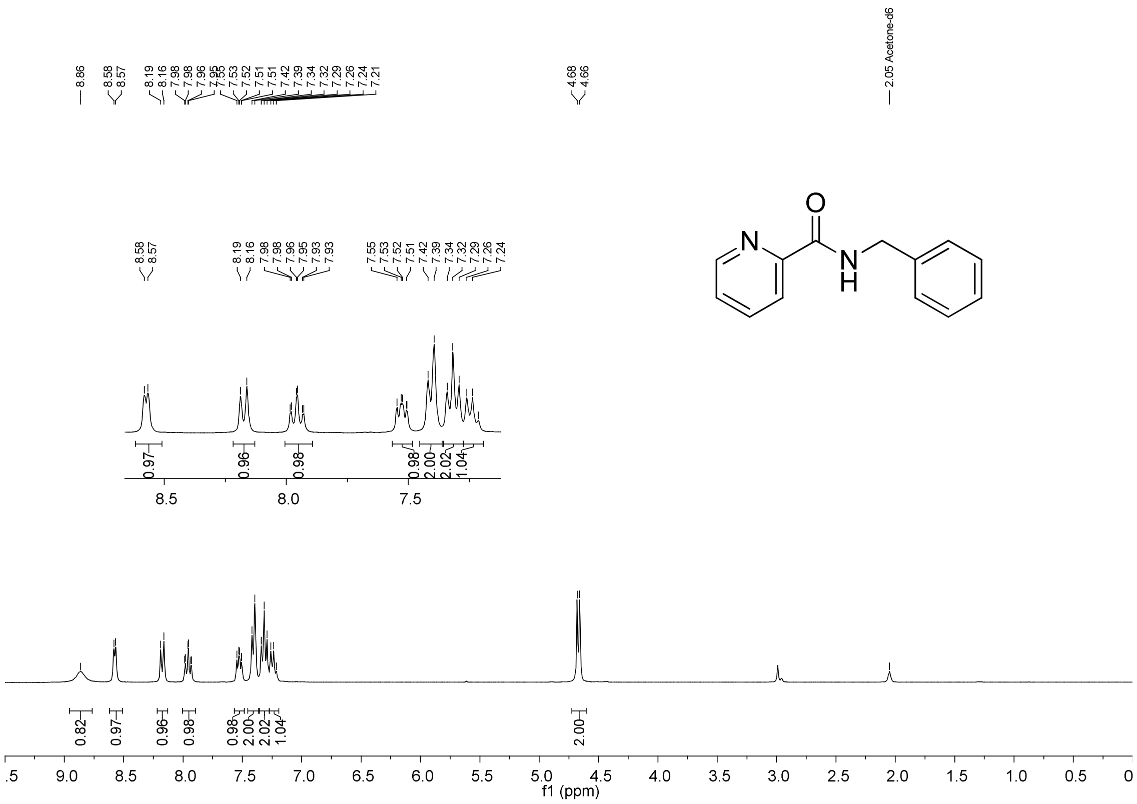

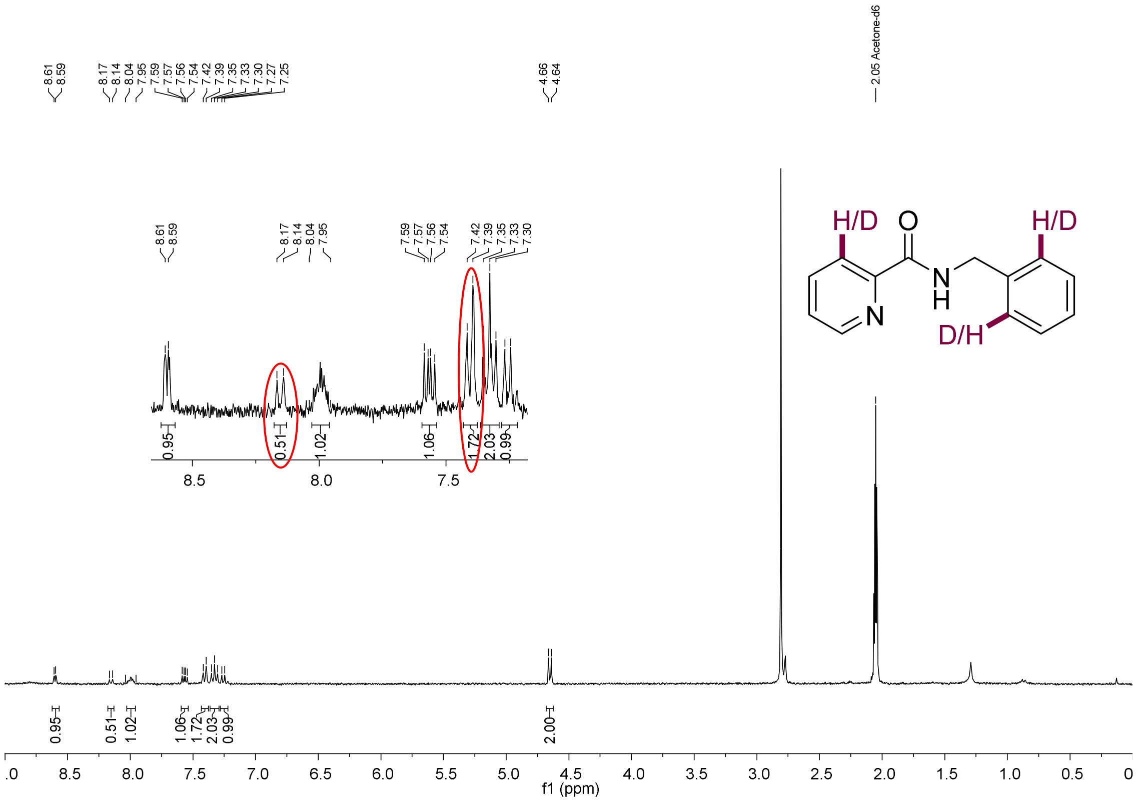

16.2.2. Rh^I^-catalyzed C–H functionalization process

16.2.2.1. Standard reaction

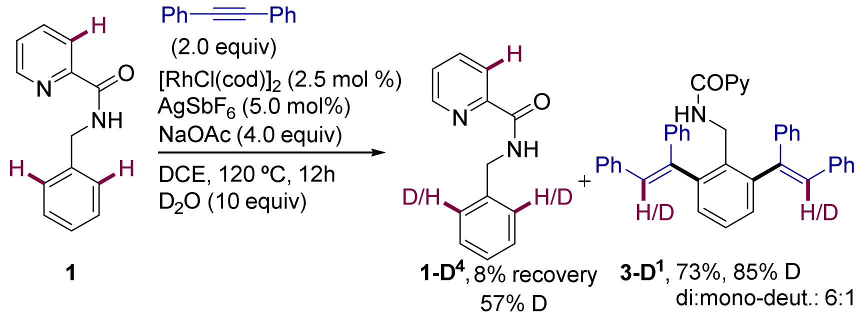

General procedure. An oven-dried, nitrogen-flushed 20 mL vessel was charged with *N*-benzylpicolinamide (**1**) (31.8 mg, 0.15 mmol, 1.00 equiv), diphenylacetylene (53.3 mg, 0.30 mmol, 2.00 equiv), chloro(1,5-cyclooctadiene)rhodium dimer (1.85 mg, 0.00375 mmol, 0.025 equiv), sodium acetate (49.8 mg, 0.60 mmol, 4.00 equiv), and silver hexafluoroantimonate(V) (2.58 mg, 0.0075 mmol, 0.05 equiv). The reaction vessel was sealed with a Teflon lined cap, then evacuated and flushed with nitrogen three times. Under the atmosphere of nitrogen, 1,2-dichloroethane (1.00 mL) and deuterium oxide (27.1 µL, 10.0 equiv) were added *via* syringe. The resulting mixture was then stirred at 120 °C for 12 h. After the reaction was complete, the volatiles were removed *in vacuo* and the residue was purified by column chromatography (*n*-hexane-EtOAc 5:1), yielding **3-D^{1}** in 73% yield (62.2 mg) and recovering 8% (3.00 mg) of **1-D^{4}**. The obtained products were analysed by ^{1}H NMR and the deuteration percentage was deduced from the comparison of the ^{1}H NMR spectra of **1** and **3** respectively.

In the spectrum of **3-D^{1}**, the integration of the peak at the singlet at 6.71 ppm (corresponding to the olefin) was 0.30 instead of 2.03 (85% H/D scrambling). Likewise, the product was characterized by ESI^{+}: Calcd. for $C_{41}H_{31}D_2N_2O$ $(M+H)^+$: 371.0625; Found: 371.0621.

In the spectrum of **1-D^{4}**, the integration of the peak at the doublet at 7.42-7.39 ppm (corresponding to the *o*-benzyl positions) was 0.86 instead of 2.00 (57% H/D scrambling).

This experiment was also performed running the reaction only for 2h. Herein the volatiles were removed *in vacuo* and the residue was purified by column chromatography (*n*-hexane-EtOAc 5:1), yielding **3-D^{1}** in 59% yield (50.0 mg) and recovering 38% (12.1 mg) of **1-D^{4}**.

In the spectrum of **3-D^{1}**, the integration of the peak at the singlet at 6.71 ppm (corresponding to the olefin) was 0.51 instead of 2.03 (75% H/D scrambling).

In the spectrum of **1-D^{4}**, the integration of the peak at the doublet at 7.42-7.39 ppm (corresponding to the *o*-benzyl positions) was 1.07 instead of 2.00 (47% H/D scrambling).

Reaction performed for 12h. Spectra of 3 and 3-D[1]

^1H NMR ($CDCl_3$, 500 MHz)

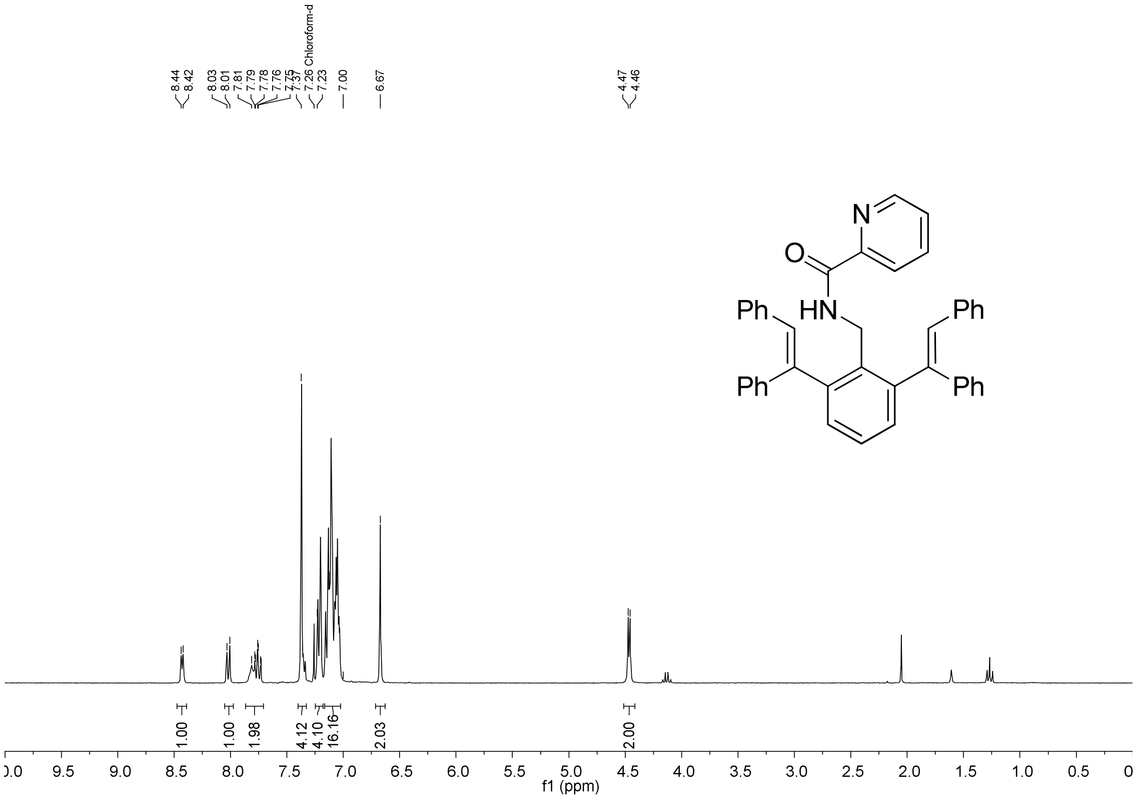

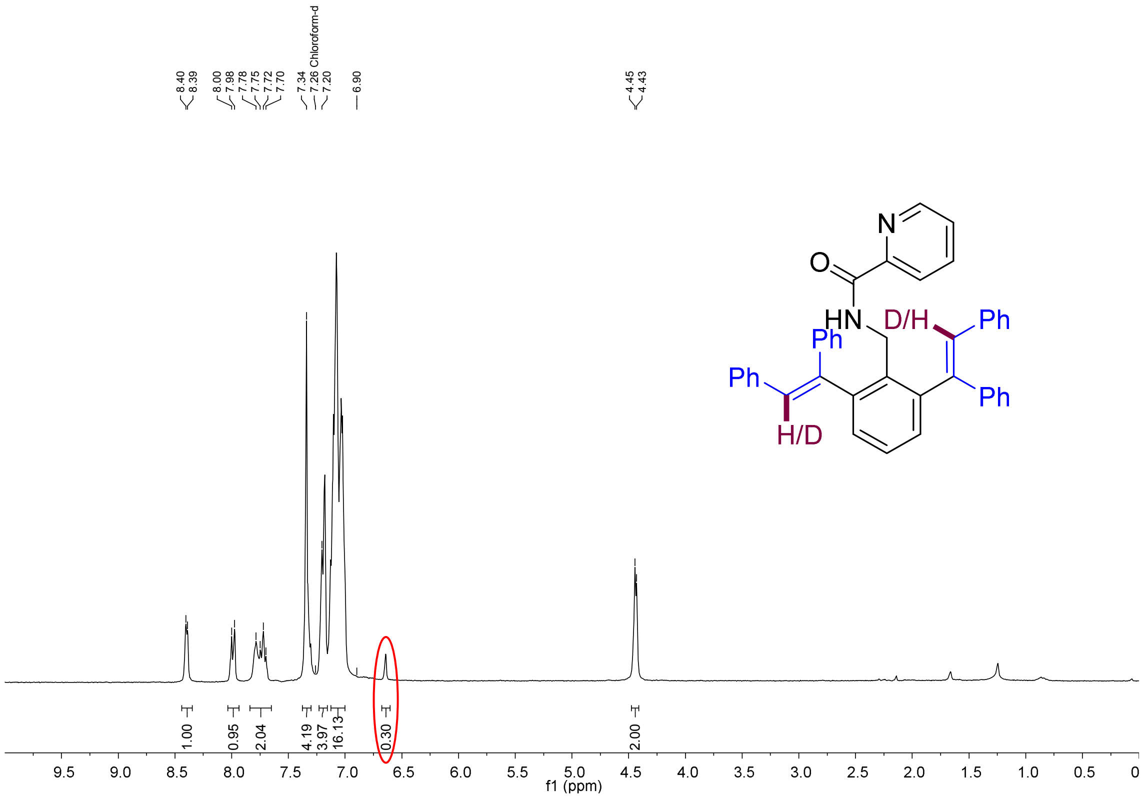

Spectra of 1 and 1-D[4]

^{1}H NMR (acetone-d$_6$, 300 MHz)

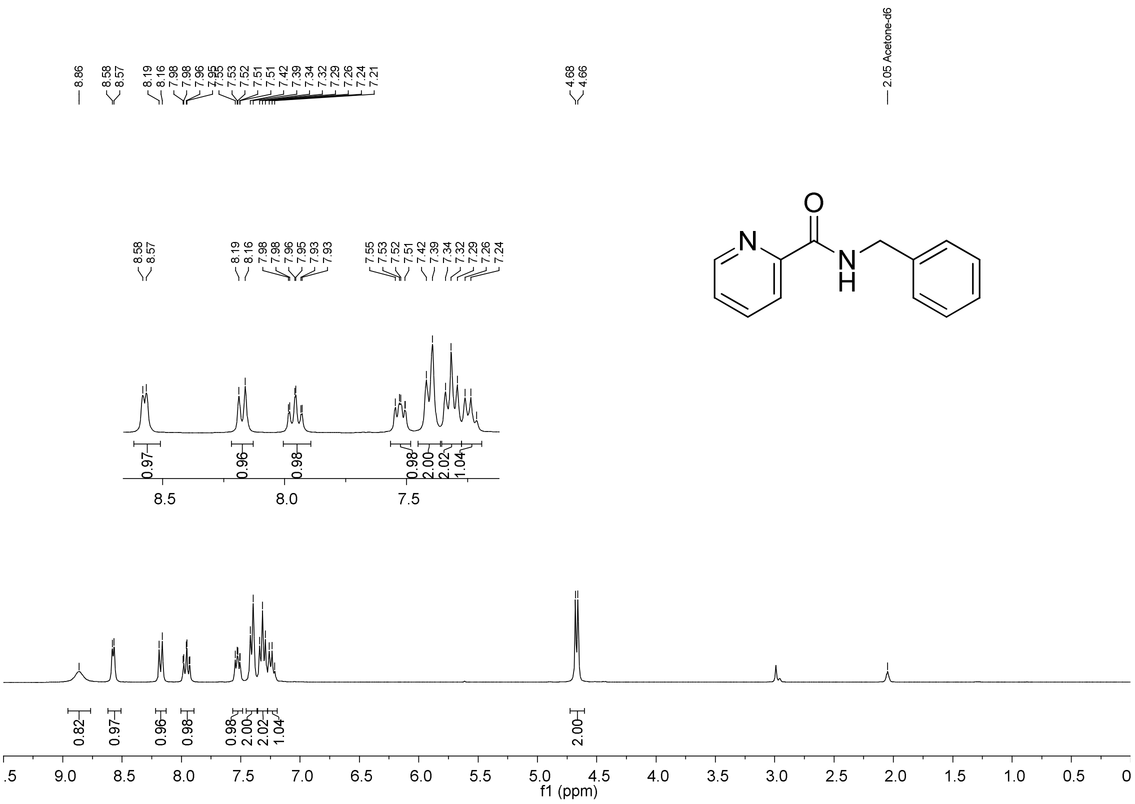

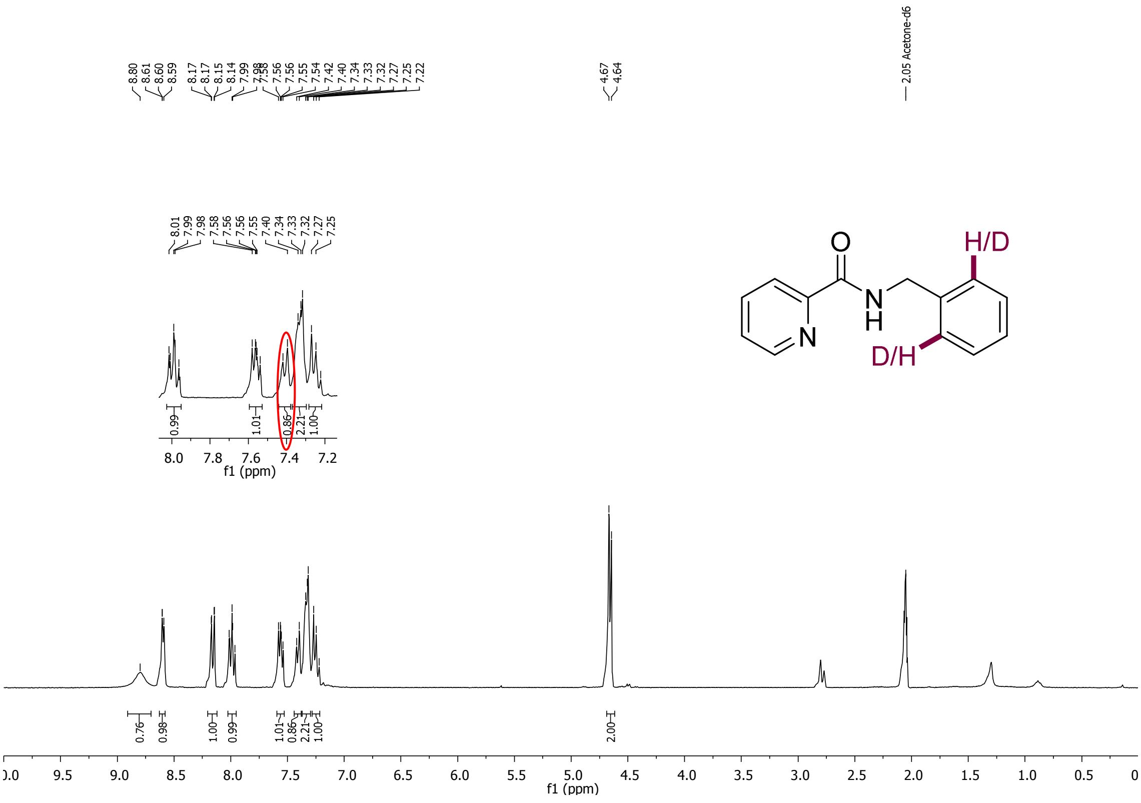

Reaction performed for 2h. Spectra of 3 and 3-D[1]

^1H NMR ($CDCl_3$, 500 MHz)

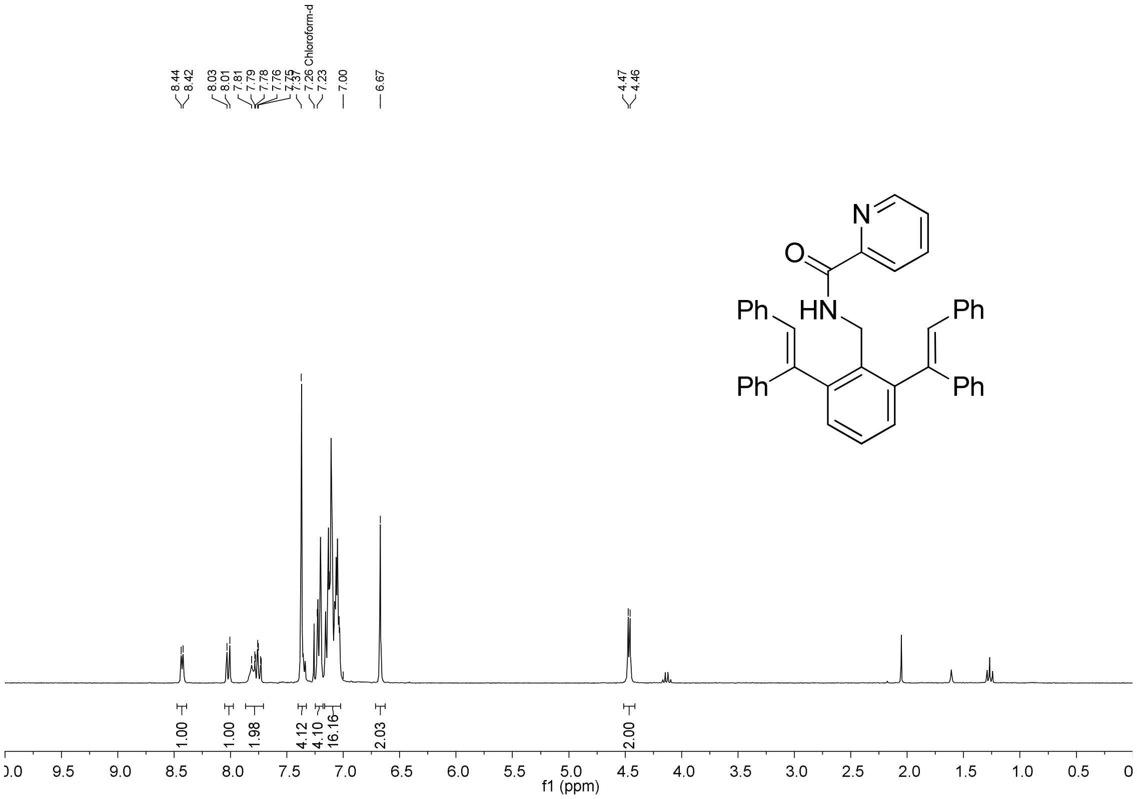

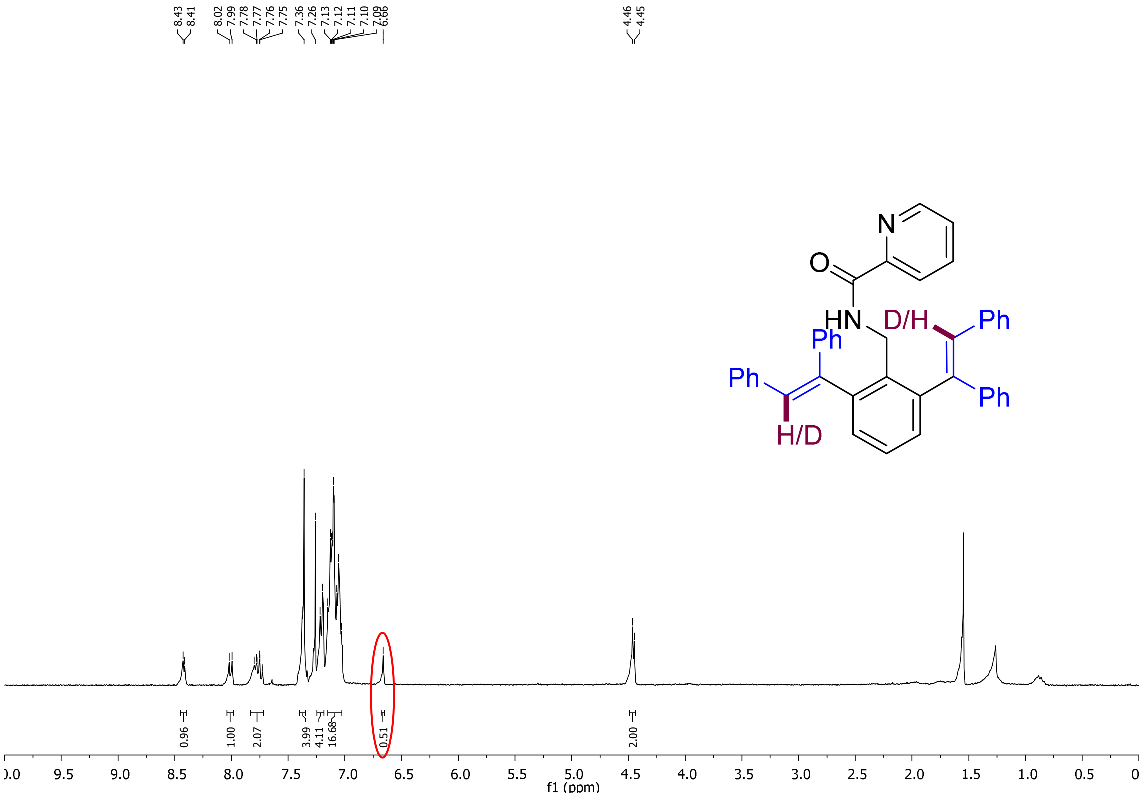

Spectra of 1 and 1-D[4]

^{1}H NMR (acetone-d$_6$, 300 MHz)

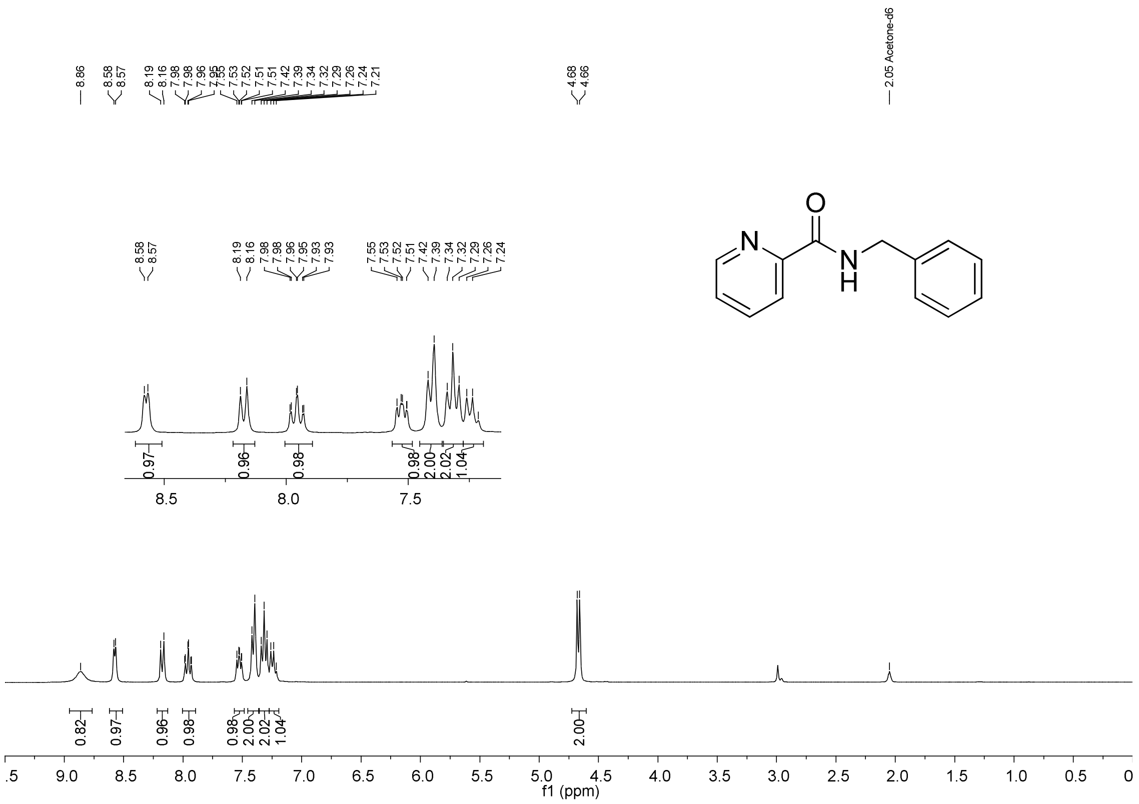

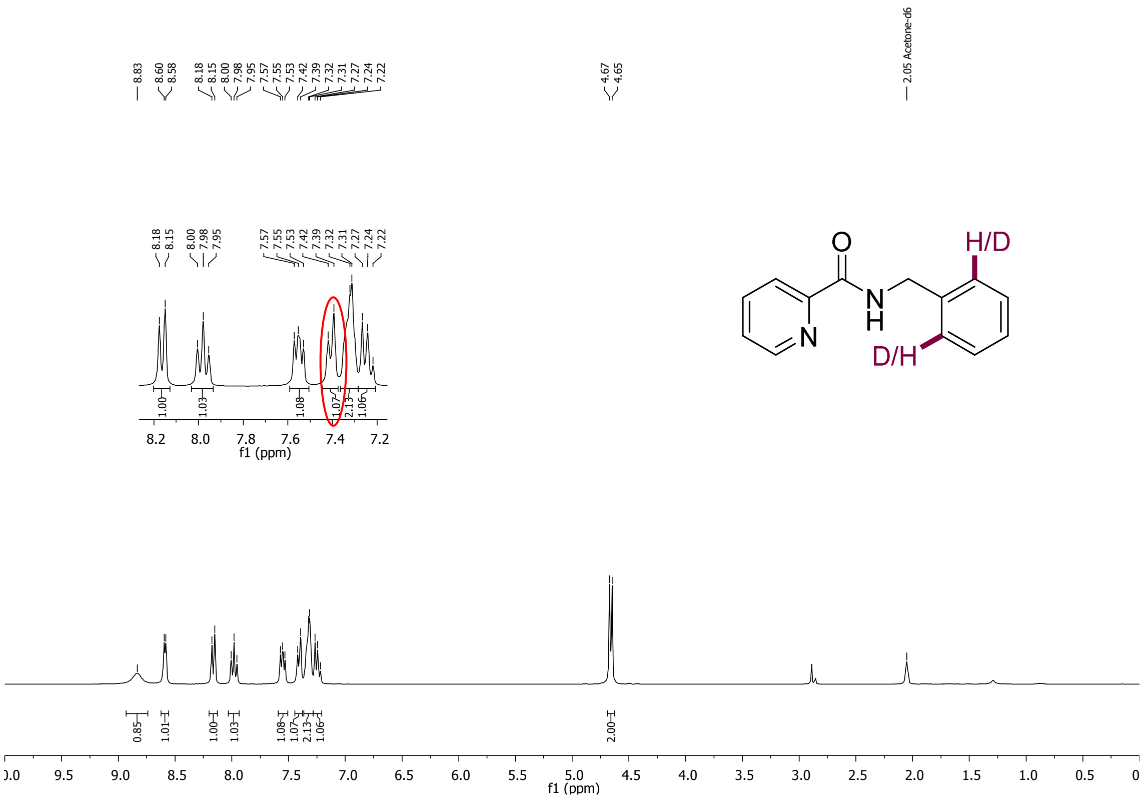

16.2.2.2. Evaluation of the potential of the Rh^{I}-complex B for the cleavage and formation of C–H bonds

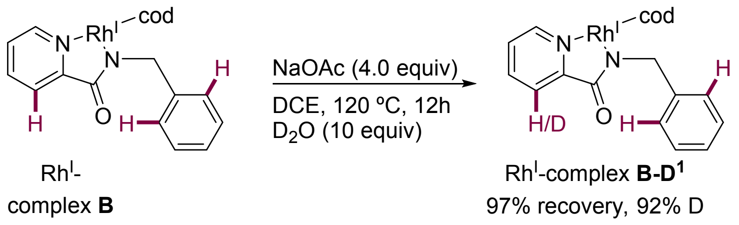

An oven-dried, nitrogen-flushed 20 mL vessel was charged with Rh-complex **B** (10.6 mg, 0.025 mmol, 1.00 equiv) and sodium acetate (8.20 mg, 0.10 mmol, 4.00 equiv). The reaction vessel was sealed with a Teflon lined cap, then evacuated and flushed with nitrogen three times. Under the atmosphere of nitrogen, 1,2-dichloroethane (1.00 mL) and deuterium oxide (4.52 µL, 10.0 equiv) were added *via* syringe. The resulting mixture was then stirred at 120 °C for 12 h. After the reaction was complete, the volatiles were removed *in vacuo* and the residue was analysed by ^{1}HNMR and the deuteration percentage was deduced from the comparison with the standard ^{1}H NMR spectrum of Rh^{I}-complex **B**. HSQC/HMBC experiments were used to assign the resonances.

In the spectrum of Rh^{I}-complex **B-D^1,** the integration of the peak at the doublet at 7.98-7.95 ppm (py-H^3) was 0.08 instead of 1.02 (92% H/D scrambling). Likewise a ^{2}H NMR experiment was performed to corroborate the deuterium presence. In addition we observed that the carbon signal corresponding to the deuterated position disappeared in the ^{13}C NMR spectrum.

Spectra of Rh[I]-complex B and Rh[I]-complex B-D[1]

^{1}H NMR (acetone-d$_6$, 500 MHz)

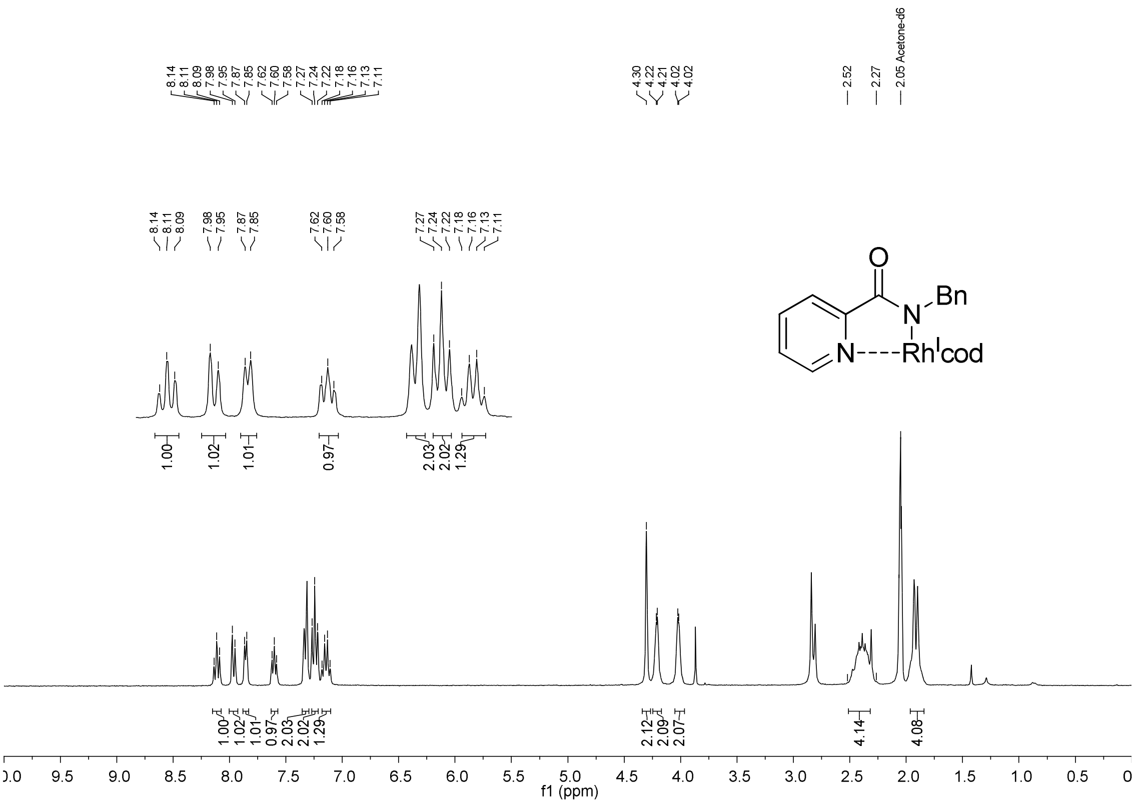

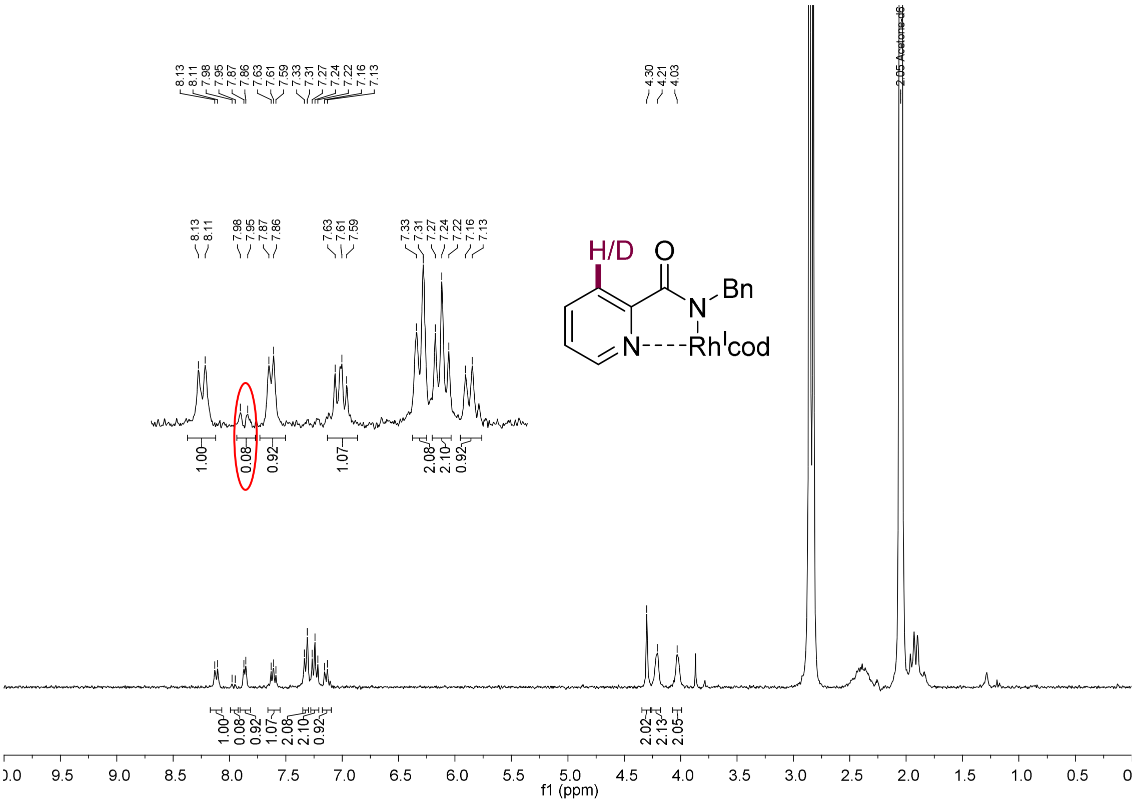

Deuterium spectrum of Rh^{I}-complex B-D^{1}

^{2}H NMR (acetone, 76 MHz)

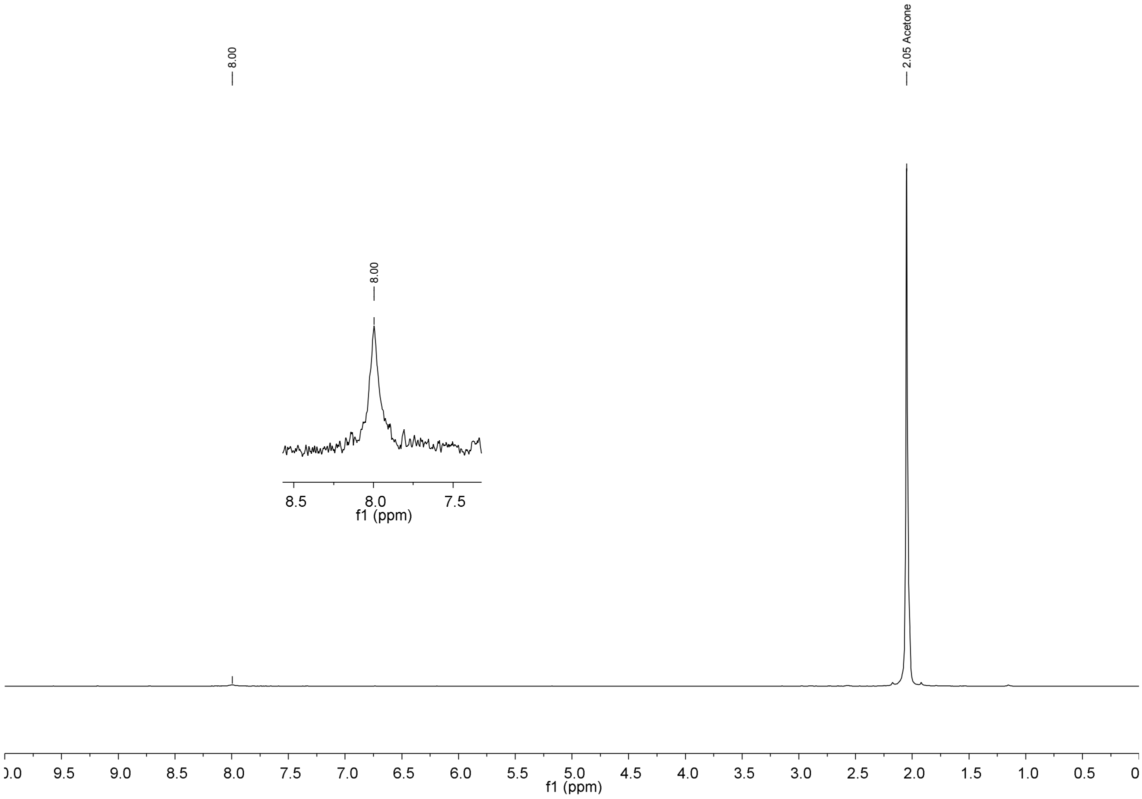

Spectra of Rh^I-complex B and Rh^I-complex B-D[1]

^{13}C NMR (acetone-d$_6$, 126 MHz)

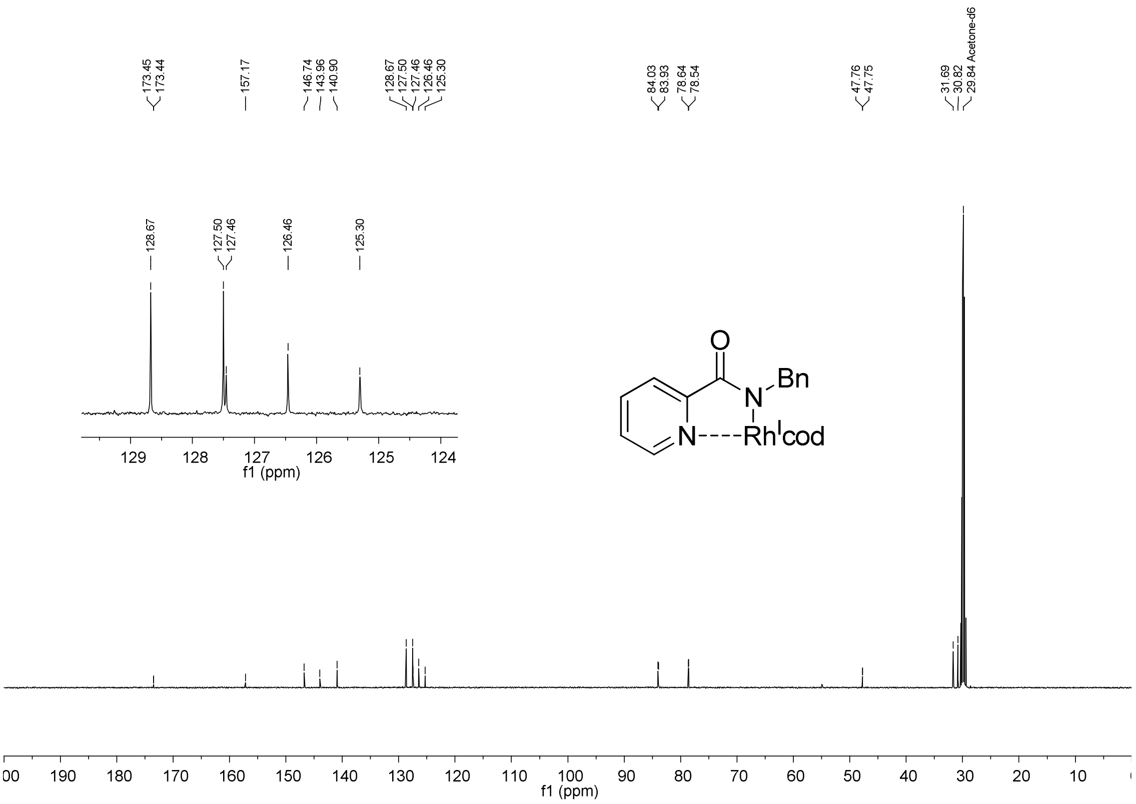

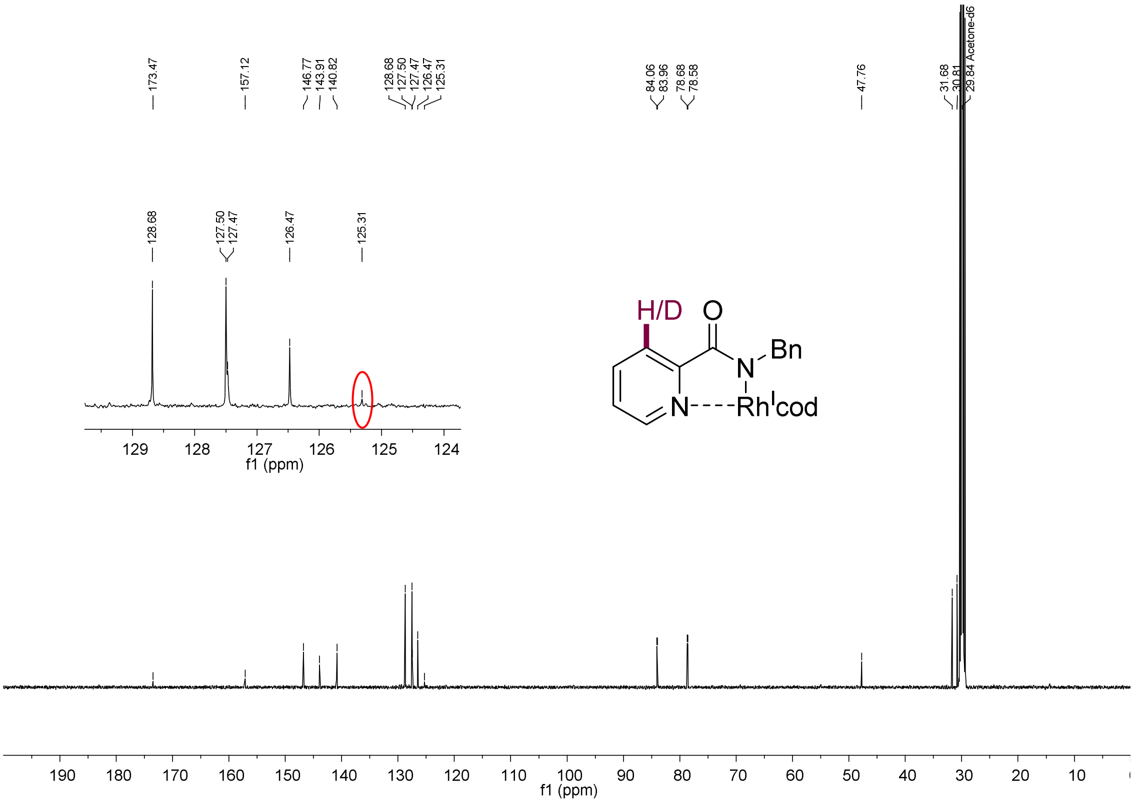

16.2.2.3. Standard reaction from Rh^{I}-complex B in presence of D_2O

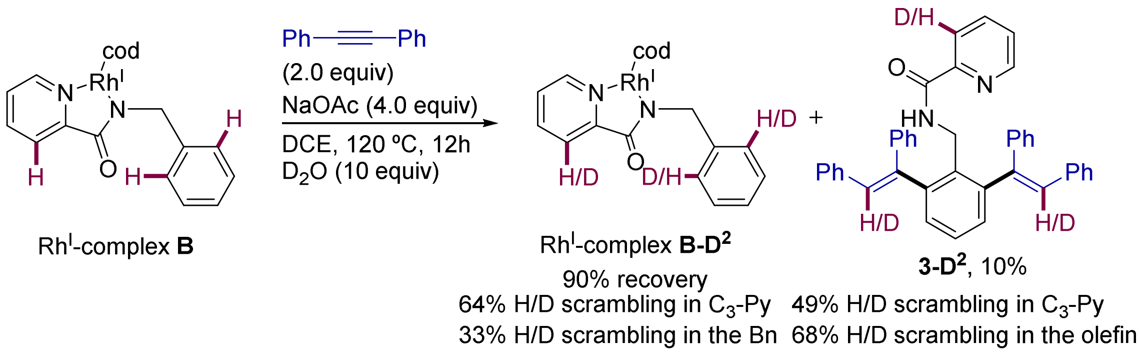

Rh^I-complex **B**

Rh^I-complex **B-D^2**
90% recovery
64% H/D scrambling in C3-Py
33% H/D scrambling in the Bn

3-D^2, 10%
49% H/D scrambling in C3-Py
68% H/D scrambling in the olefin

An oven-dried, nitrogen-flushed 20 mL vessel was charged with Rh^{I}-complex **B** (21.1 mg, 0.05 mmol, 1.00 equiv), diphenylacetylene (17.8 mg, 0.10 mmol, 2.00 equiv) and sodium acetate (16.4 mg, 0.10 mmol, 4.00 equiv). The reaction vessel was sealed with a Teflon lined cap, then evacuated and flushed with nitrogen three times. Under the atmosphere of nitrogen, 1,2-dichloroethane (1.00 mL) and deuterium oxide (9.04 µL, 10.0 equiv) were added *via* syringe. The resulting mixture was then stirred at 120 °C for 12 h. After the reaction was complete, the volatiles were removed *in vacuo* and the residue was purified by column chromatography (*n*-hexane-EtOAc 4:1), yielding Rh^{I}-complex **B-D**^{2} in 90% yield (18.3 mg) and recovering 10% (2.86 mg) of **3-D**^{2}.Then the reaction was analysed by ^{1}HNMR and the deuteration percentage was deduced from the comparison with the standard ^{1}H NMR spectrum of Rh^{I}-complex **B**.

In the spectrum of the remaining Rh^{I}-complex **B-D**^{2}, the integration of the doublet at 8.17-8.14 ppm (py-H^{3}) was 0.37 instead of 1.02 (64% H/D scrambling) and the integration of the doublet at 7.42-7.39 ppm (corresponding to the *o*-benzyl positions) was 1.35 instead of 2.03 (33% H/D scrambling).

In the spectrum of **3-D**^{2}, the integration of the doublet at 8.17-8.14 ppm (py-H^{3}) was 0.53 instead of 1.04 (49% H/D scrambling) and the integration of the doublet at 7.42-7.39 ppm (corresponding to the olefin) was 0.67 instead of 2.08 (68% H/D scrambling).

Spectra of Rh^{I}-complex B and Rh^{I}-complex B-D^{2}

^{1}H NMR (acetone-d$_6$, 500 MHz)

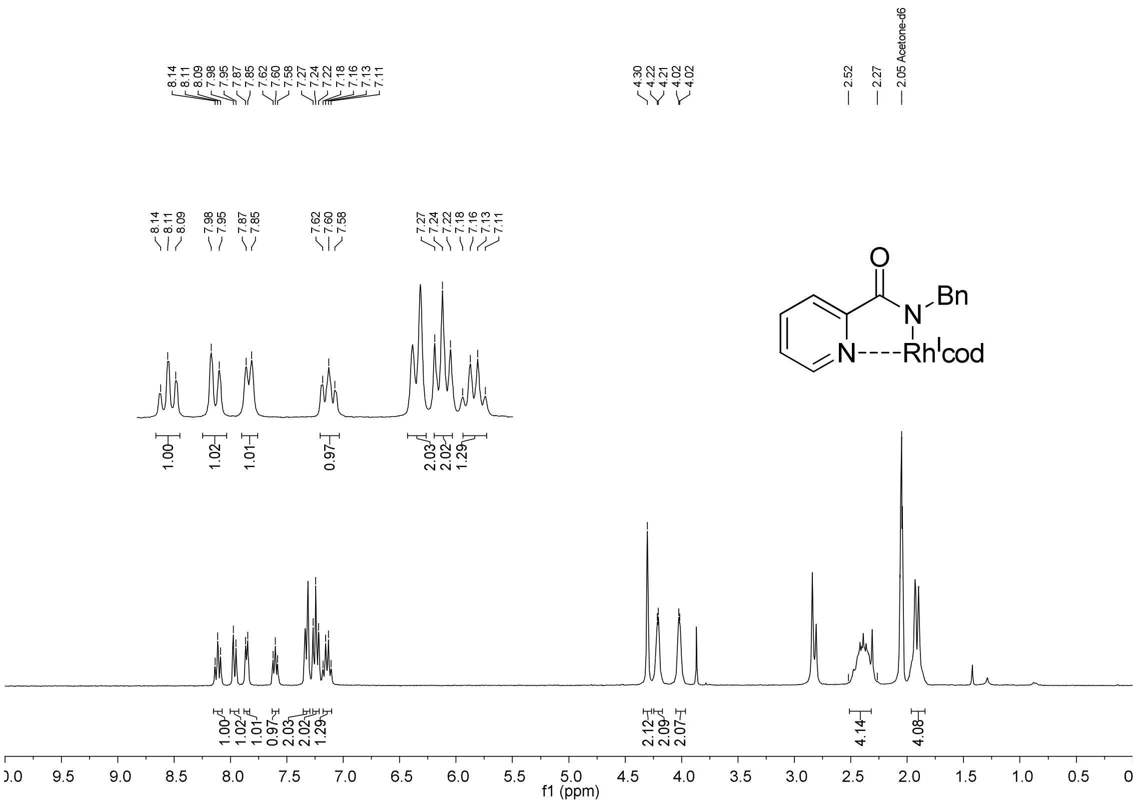

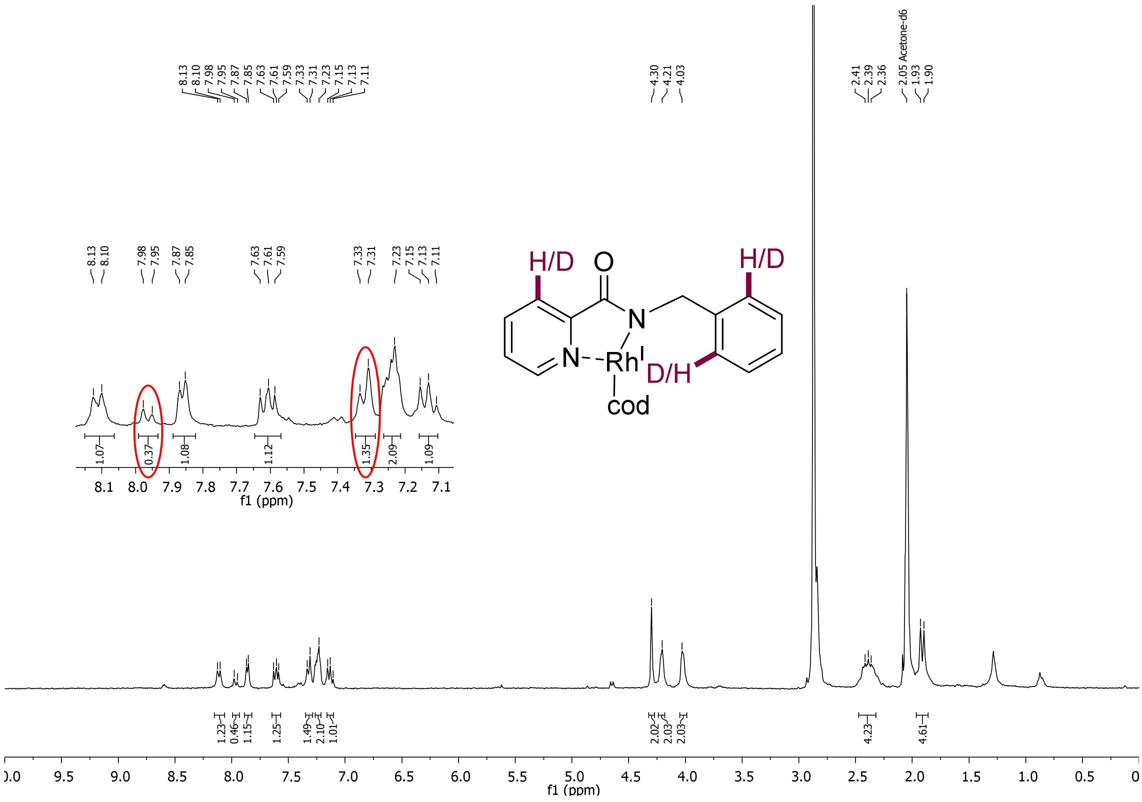

Spectra of 3 and 3-D[2]

^{1}H NMR ($CDCl_3$, 500 MHz)

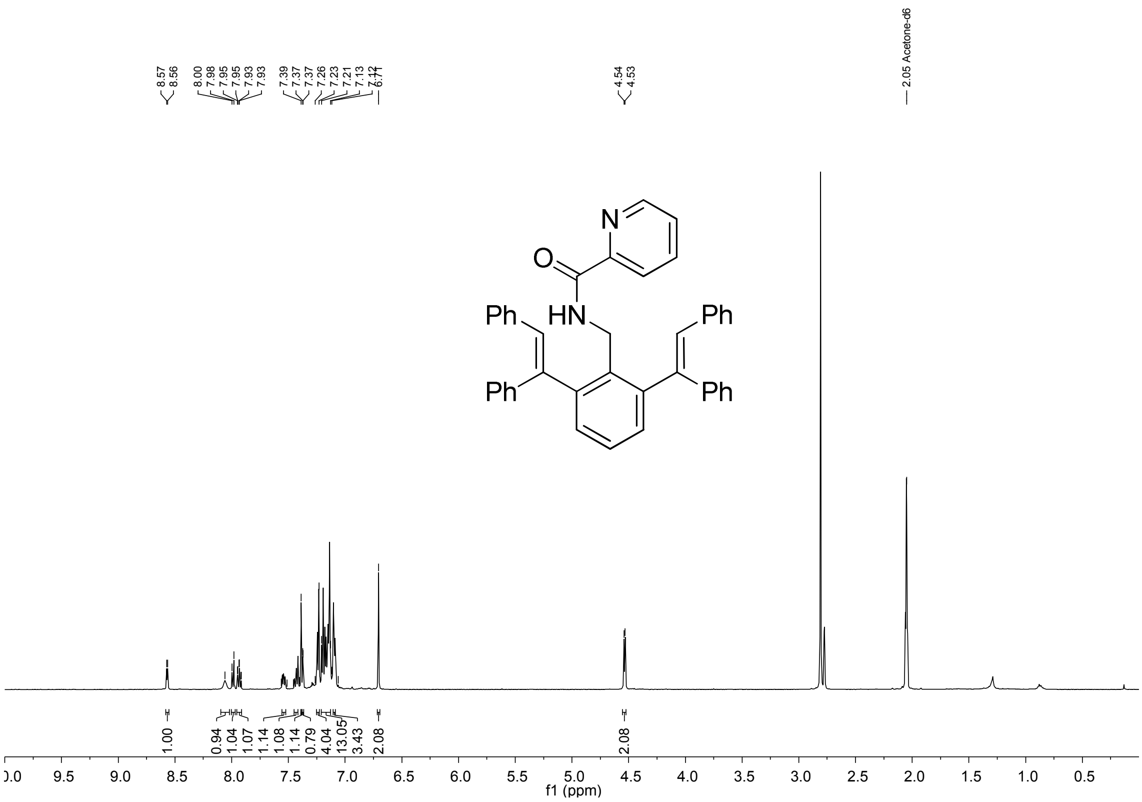

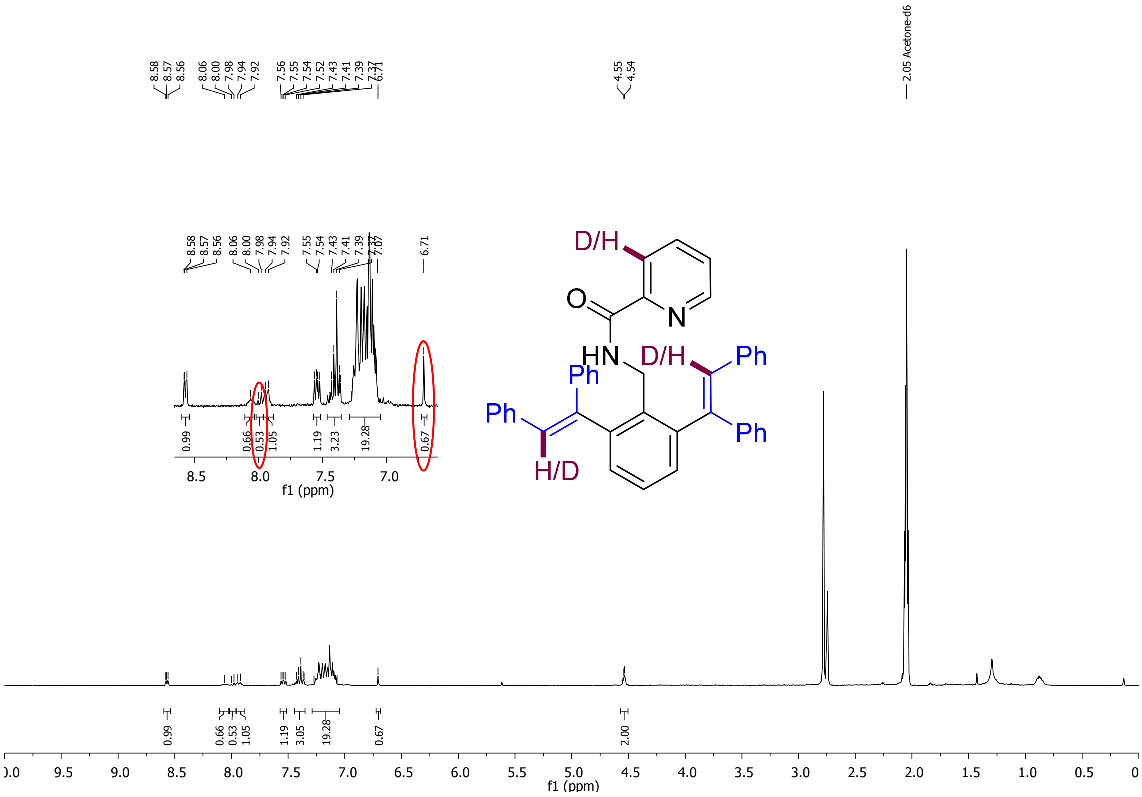

16.3. Kinetic studies of the Rh^{I}-catalyzed *ortho*-olefination of *N*-benzylamide derivatives

These studies were performed running several identical reactions in parallel, each of them stopped at the given time.

16.3.1. Evaluation of the substitution of the aryl alkyne

General procedure. An oven-dried, nitrogen-flushed 20 mL vessel was charged with *N*-benzylpicolinamide (**1**) (31.8 mg, 0.15 mmol, 1.00 equiv), alkyne (0.30 mmol, 2.00 equiv), (chloro(1,5-cyclooctadiene)rhodium dimer (1.85 mg, 0.00375 mmol, 0.025 equiv), sodium acetate (49.8 mg, 0.60 mmol, 4.00 equiv), and silver hexafluoroantimonate(V) (2.58 mg, 0.0075 mmol, 0.05 equiv). The vessel was sealed with a Teflon lined cap, then evacuated and flushed with N_2 three times. Under the N_2 atmosphere, 1,2-dichloroethane (1.00 mL) was added *via* syringe and the resulting mixture was then stirred at 120 °C for a given time. Percentage of the final product was determined by ^{1}HNMR of the crude mixture.

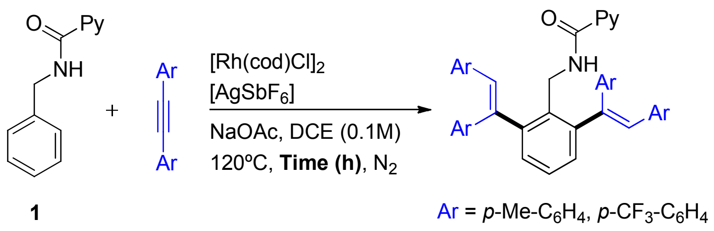

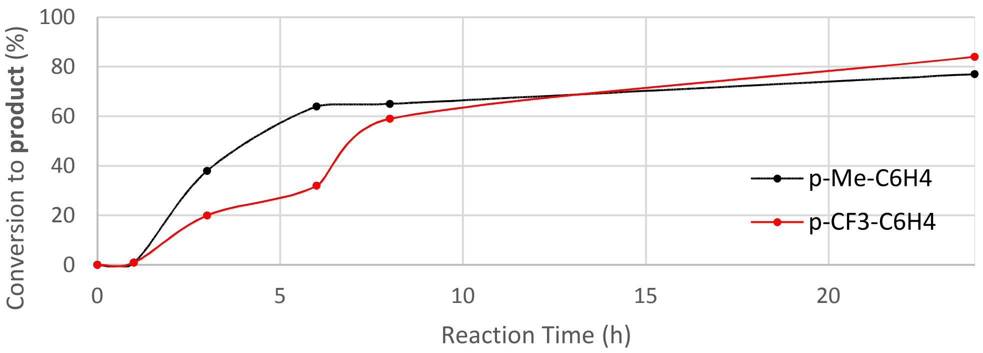

16.3.2. Evaluation of the substitution of the *N*-benzylamide

General procedure. An oven-dried, nitrogen-flushed 20 mL vessel was charged with picolinamide derivative (0.15 mmol, 1.00 equiv), chloro(1,5-cyclooctadiene)rhodium dimer (1.85 mg, 0.00375 mmol, 0.025 equiv), sodium acetate (49.8 mg, 0.60 mmol, 4.00 equiv), silver hexafluoroantimonate(V) (2.58 mg, 0.0075 mmol, 0.05 equiv) and diphenylacetylene (35.6 mg, 0.20 mmol, 2.00 equiv). The vessel was sealed with a Teflon lined cap, then evacuated and flushed with N_2 three times. Under the atmosphere of N_2, 1,2-dichloroethane (1.00 mL) was added *via* syringe and the resulting mixture was then stirred at 120 °C for a given time. Percentage of the final product was determined by ^{1}HNMR of the crude mixture.

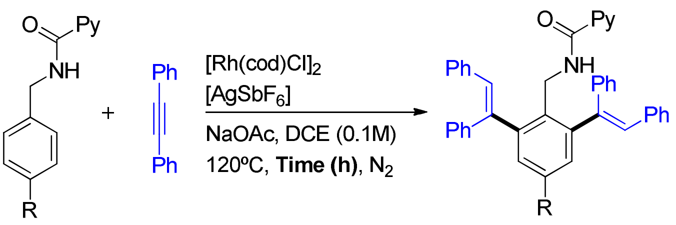

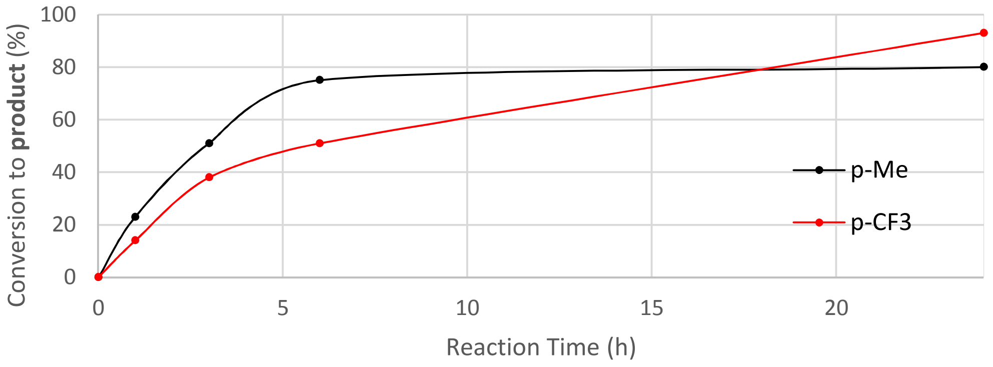

16.3.1. Standard reaction from Rh^I-complex B in presence of D

General procedure. An oven-dried, nitrogen-flushed 20 mL vessel was charged with *N*-benzylpicolinamide (**1**) (21.2 mg, 0.10 mmol, 1.00 equiv), diphenylacetylene (35.6 mg, 0.20 mmol, 2.00 equiv) and sodium acetate (32.8 mg, 0.40 mmol, 4.00 equiv). The reaction vessel was sealed with a Teflon lined cap, then evacuated and flushed with nitrogen three times. Under the atmosphere of N_2, a solution of the Rh^I-based catalyst (5 mol% of Rh^I) in 1,2-dichloroethane (1.00 mL) was added *via* syringe and the resulting mixture was then stirred at 120 °C for a given time. Percentage of **3** was determined by ^{1}HNMR of the crude mixture. When using $[Rh(cod)Cl]_2$, silver hexafluoroantimonate(V) (1.72 mg, 0.005 mmol, 0.05 equiv) was added.

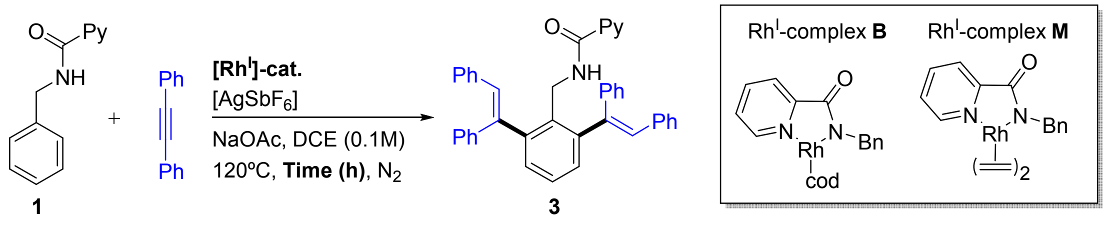

$[Rh^I]$-cat. = $[Rh(cod)Cl]_2$, $[Rh(acac)(C_2H_4)_2]$, Rh^I-complex **B**, Rh^I-complex **M**

$[Rh(cod)Cl]_2$ *vs* $[Rh(acac)(C_2H_4)_2]$

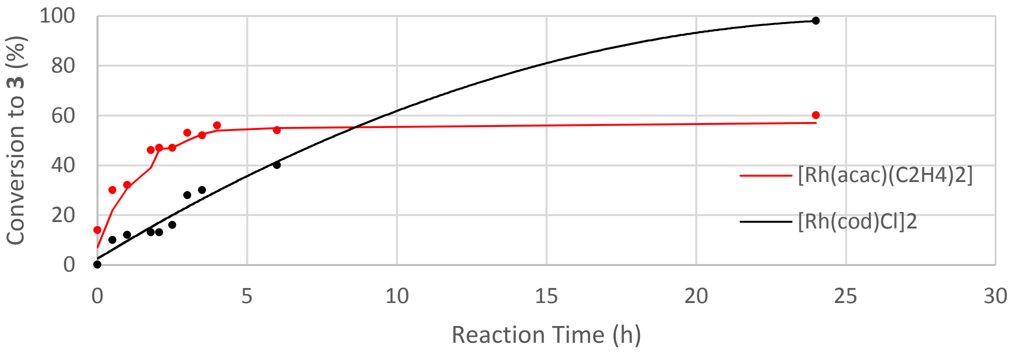

Rh^I-complex B *vs* Rh^I-complex M

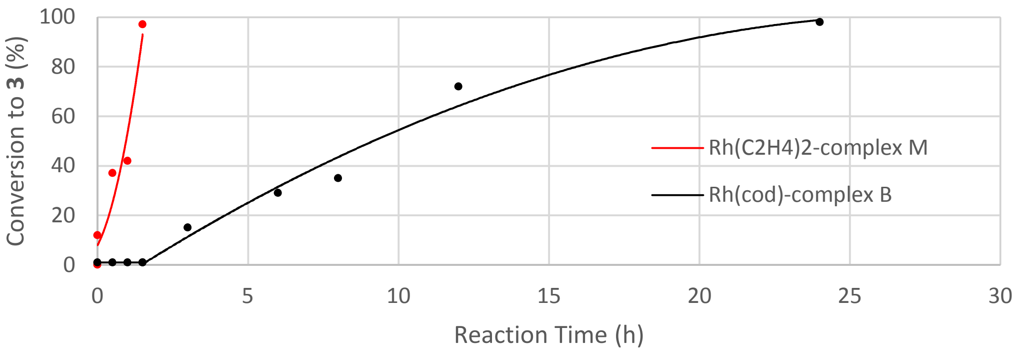

16.4. Role of the base in the Rh^{I}-catalyzed *ortho*-olefination of *N*-benzylamine (1)

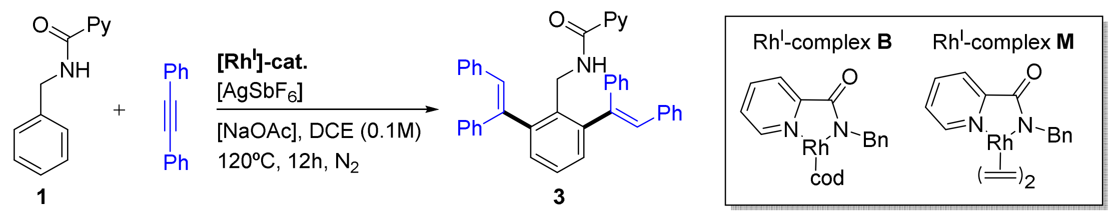

[Rh^{I}]-cat. = [Rh(cod)Cl]$_2$, [Rh(acac)(C$_2$H$_4$)$_2$], Rh^{I}-complex **B**, Rh^{I}-complex **M**

**Entry**	**[Rh]-cat.**	**NaOAc**[a]	**3 (%)**[b]
**1**[c]	[Rh(cod)Cl]$_2$	✓	55
		X	<1
**2**	[Rh(acac)(C$_2$H$_4$)$_2$]	✓	54
		X	49
**3**	Rh^{I}-complex **B**	✓	72
		X	37
**4**	Rh^{I}-complex **M**	✓	89
		X	51

Reaction conditions: **1** (0.15 mmol, 1.00 equiv), diphenylacetylene (0.30 mmol, 2.00 equiv), 5 mol% of [Rh^{I}], DCE (0.1M), 120 °C, 12 h. [a] NaOAc (4.00 equiv). [b] Conversion yield determined by ^{1}H NMR from the crude mixture. [c] AgSbF$_6$ (1.72 mg, 0.005 mmol, 0.05 equiv) was added.

17. NMR Spectra

The chemical shifts of the solvents (used in this SI) signals observed for ^{1}H NMR and ^{13}C NMR spectra are listed in the following chart. The multiplicity is shown as 1 for singlet, 2 for doublet, etc.

Solvent	^{1}H NMR Chemical Shift (ppm)	^{13}C NMR Chemical Shift (ppm)
**Acetone**	11.65 (1), 2.04 (5)	206.7 (13), 29.9 (7)
**Chloroform**	7.26 (1)	77.2 (3)
**Methanol**	4.87 (1), 3.31(5)	49.1 (7)

In the following table are the chemical shifts of the water signal in the solvents listed before. (H_2O in aprotic solvents or HOD in protic solvents)

Solvent	^{1}H NMR Chemical Shift (ppm)
**Acetone**	2.84
**Chloroform**	1.56
**Methanol**	4.87

N-Benzylpicolinamide (1)

^{1}H NMR ($CDCl_3$, 300 MHz)

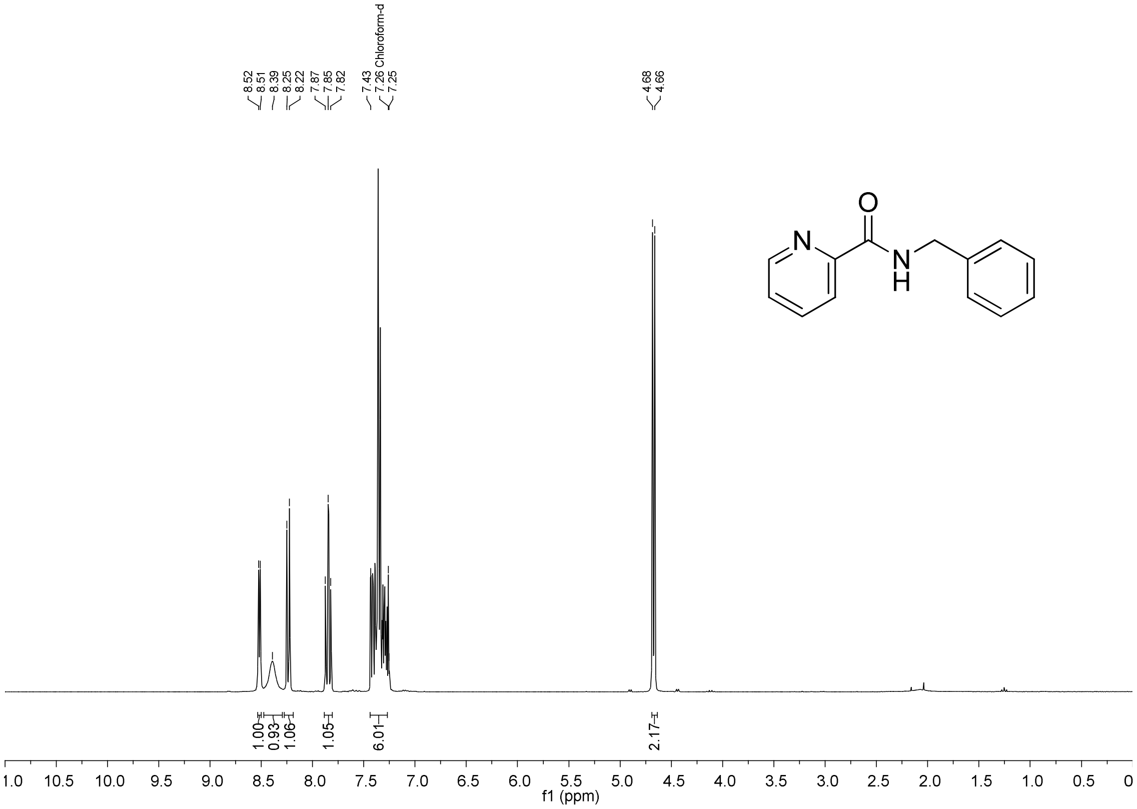

^{13}C NMR ($CDCl_3$, 75 MHz)

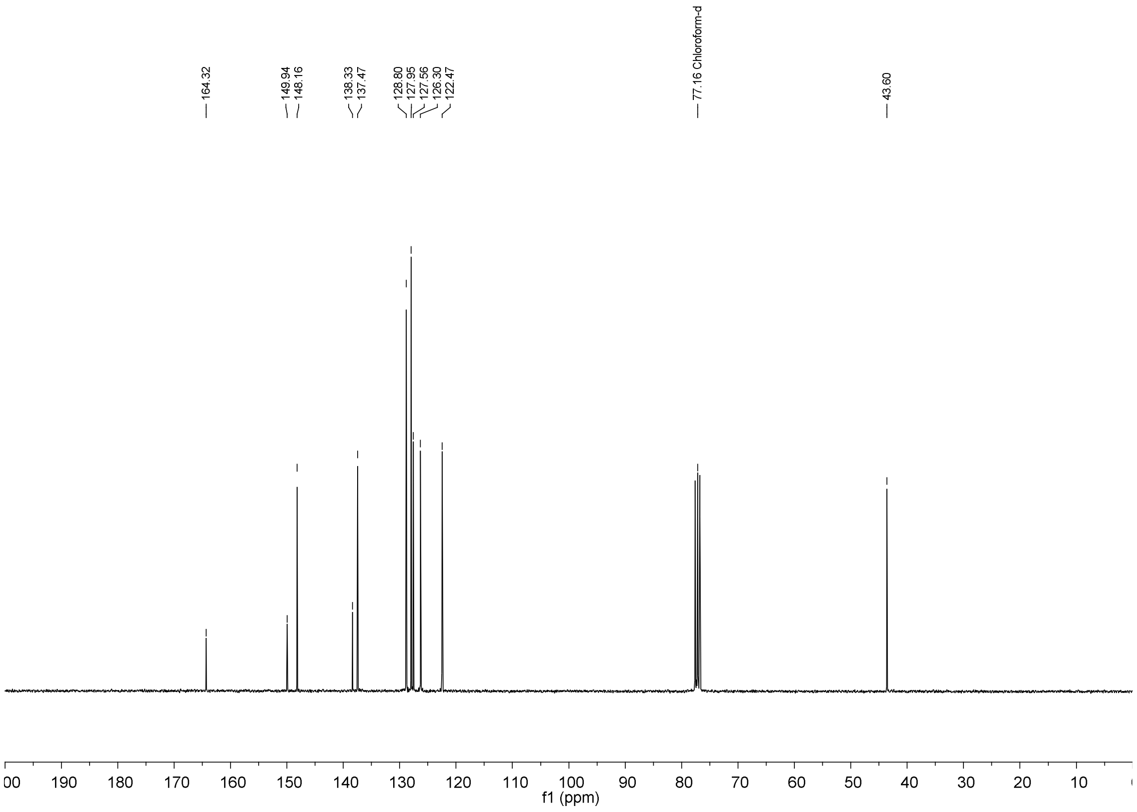

HSQC ($CDCl_3$, 500 MHz)

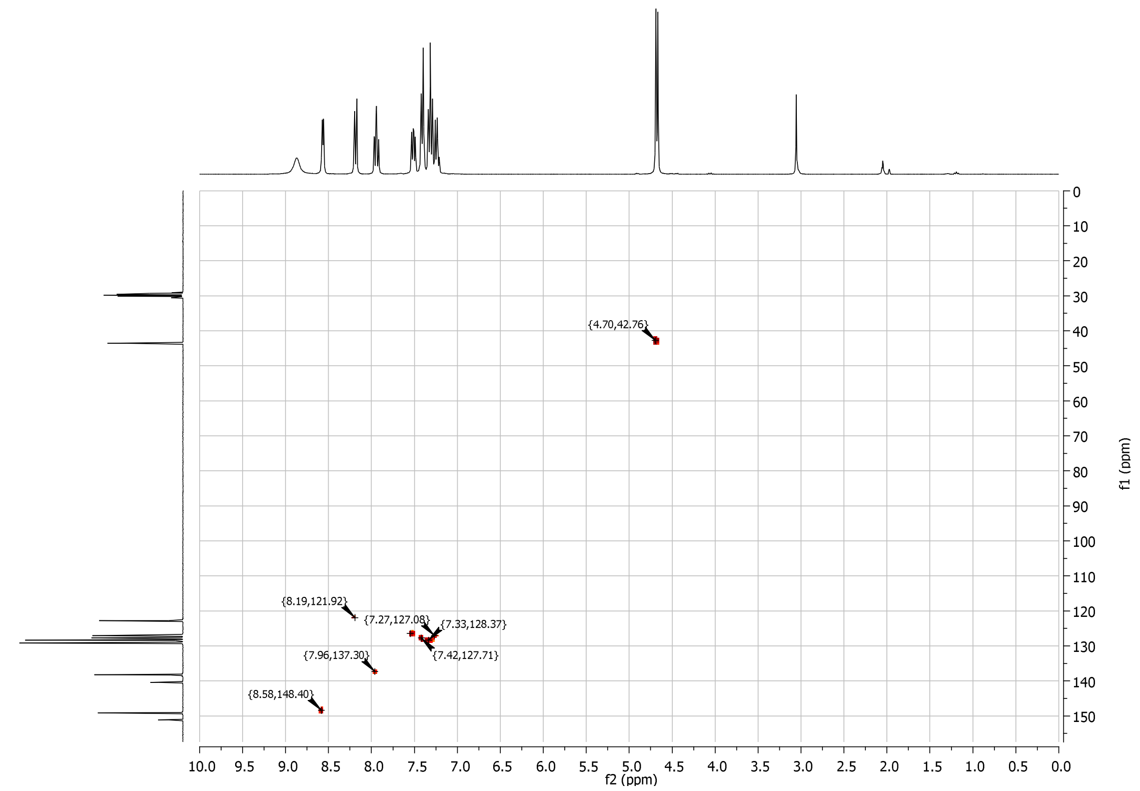

HMBC ($CDCl_3$, 500 MHz)

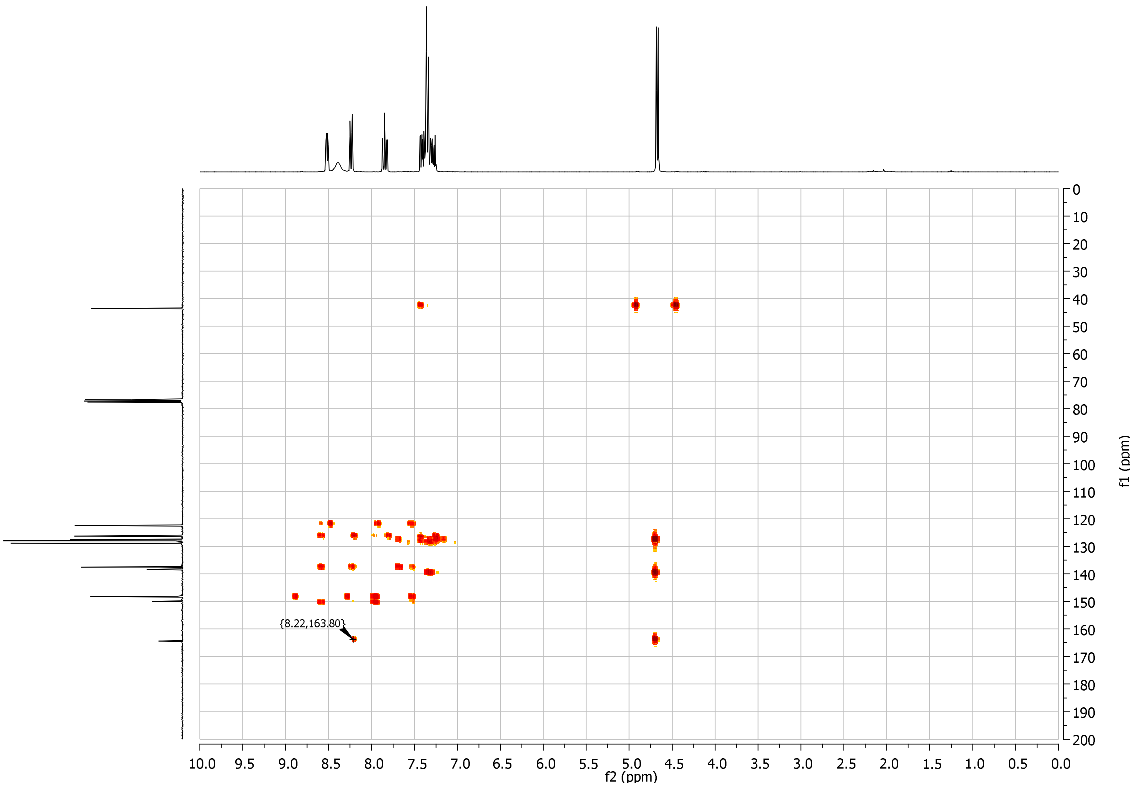

***N*-Benzyl-6-methylpicolinamide (5)**

^{1}H NMR ($CDCl_3$, 300 MHz)

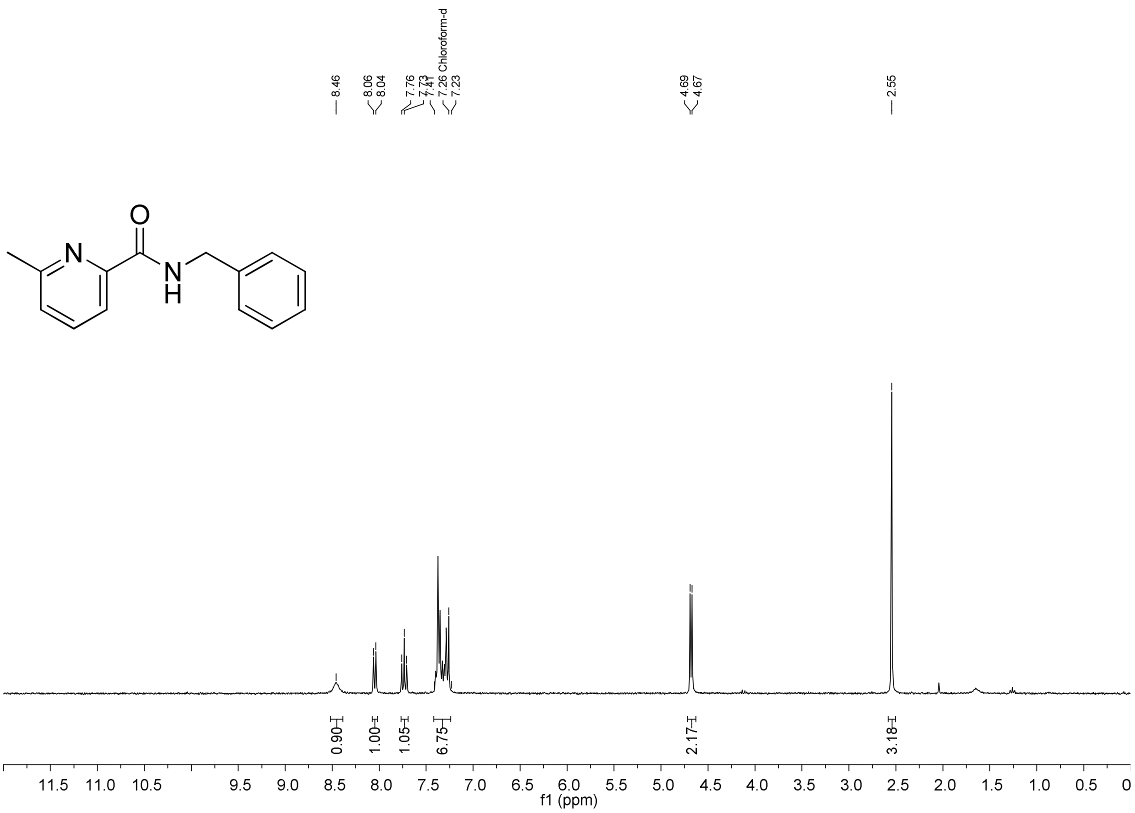

^{13}C NMR ($CDCl_3$, 75 MHz)

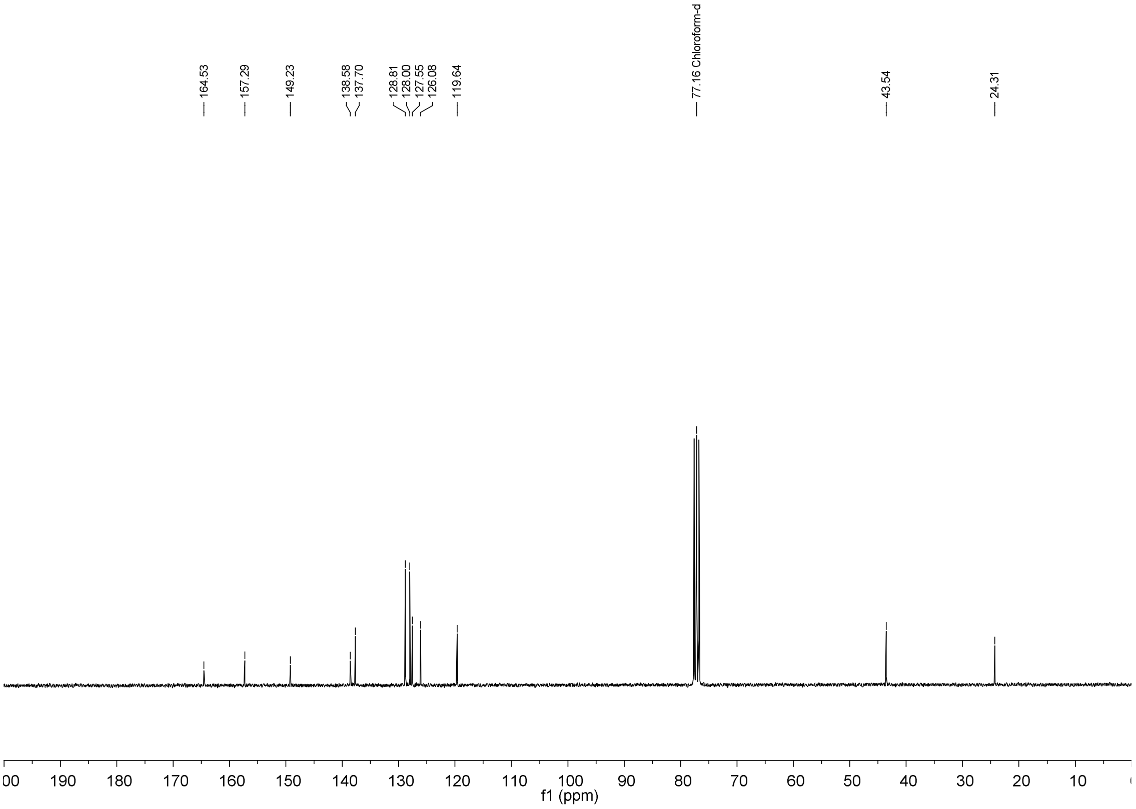

***N*-Benzyl-6-chloropicolinamide (6)**

^{1}H NMR ($CDCl_3$, 300 MHz)

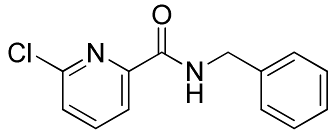

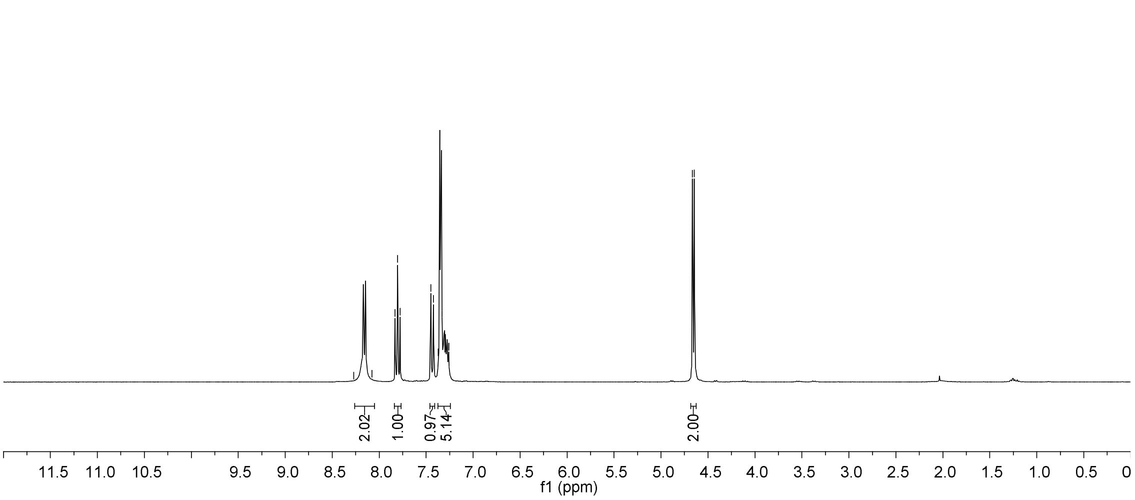

^{13}C NMR ($CDCl_3$, 75 MHz)

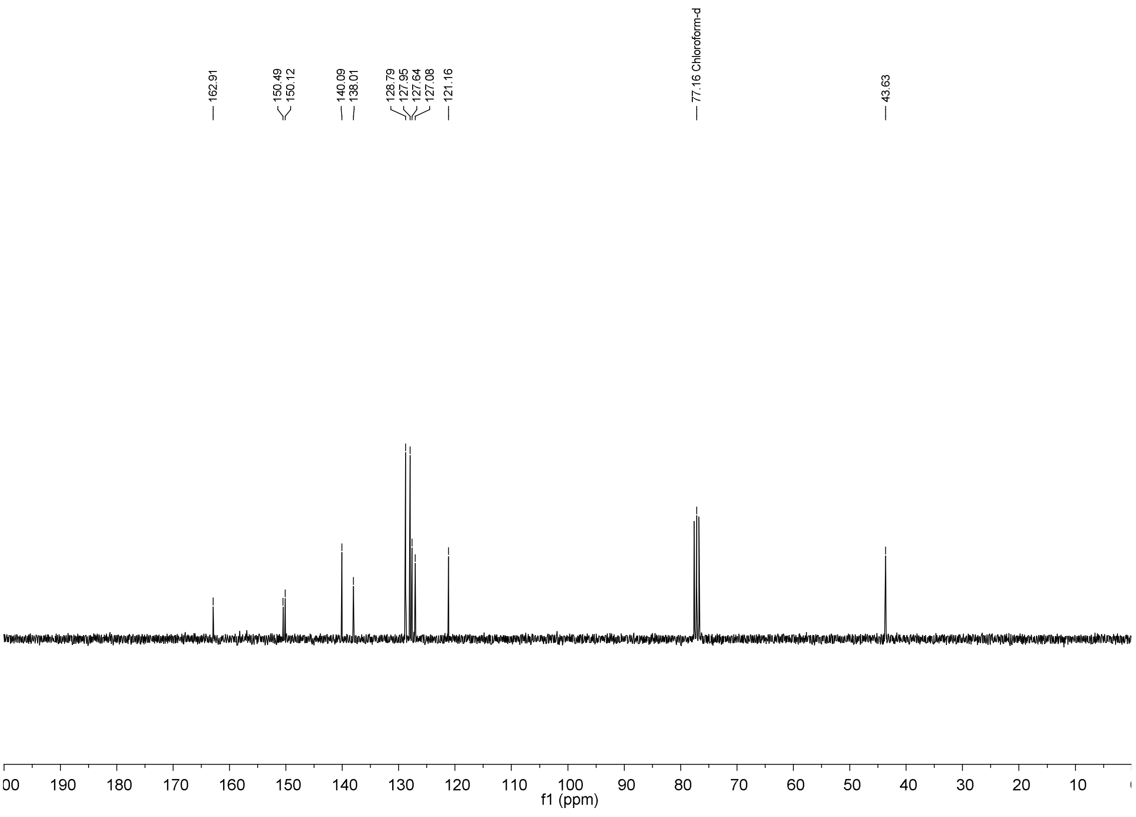

N-Benzyl-5-(trifluoromethyl)picolinamide (7)

^{1}H NMR ($CDCl_3$, 300 MHz)

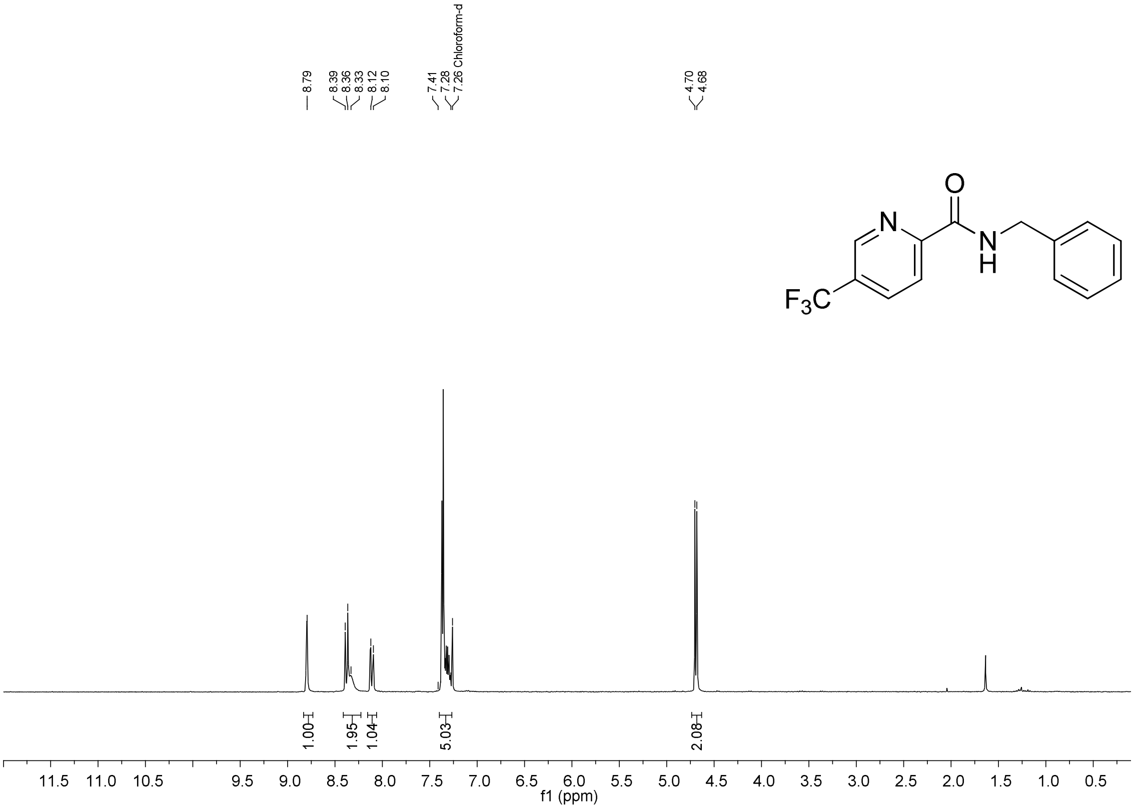

^{13}C NMR ($CDCl_3$, 75 MHz)

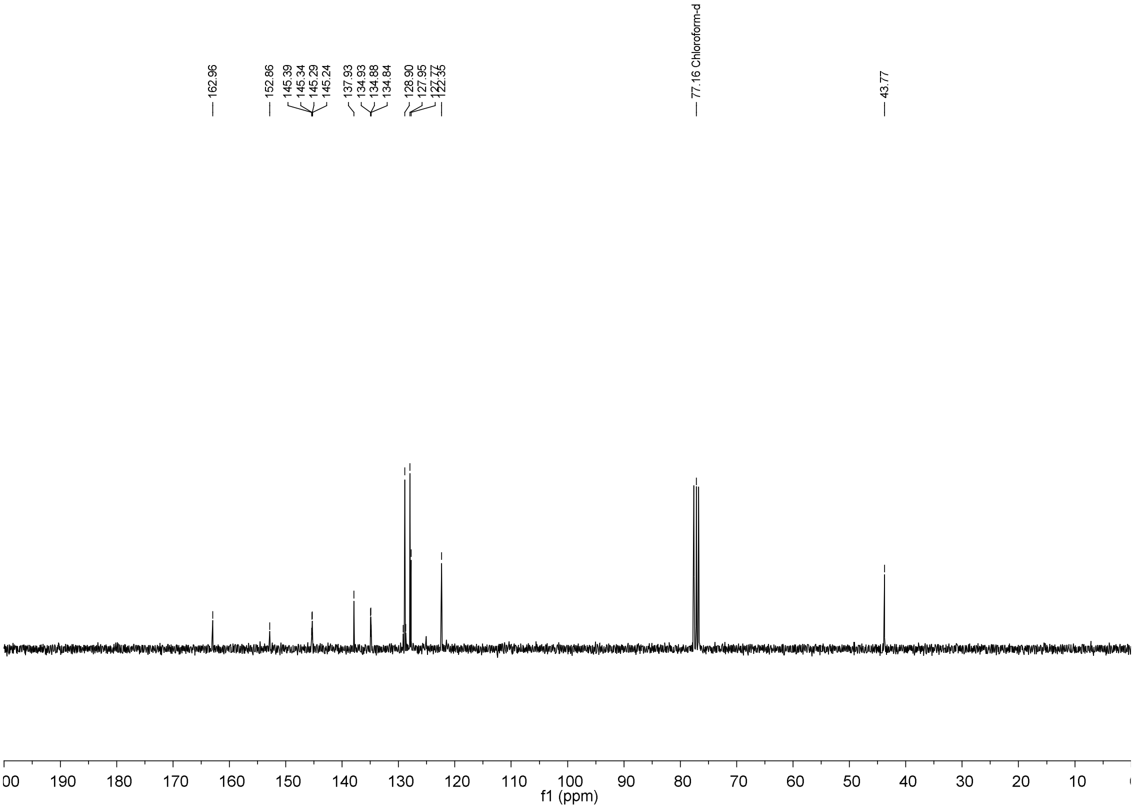

N-(4-Methoxybenzyl)-3-methylpicolinamide

^{1}H NMR ($CDCl_3$, 300 MHz)

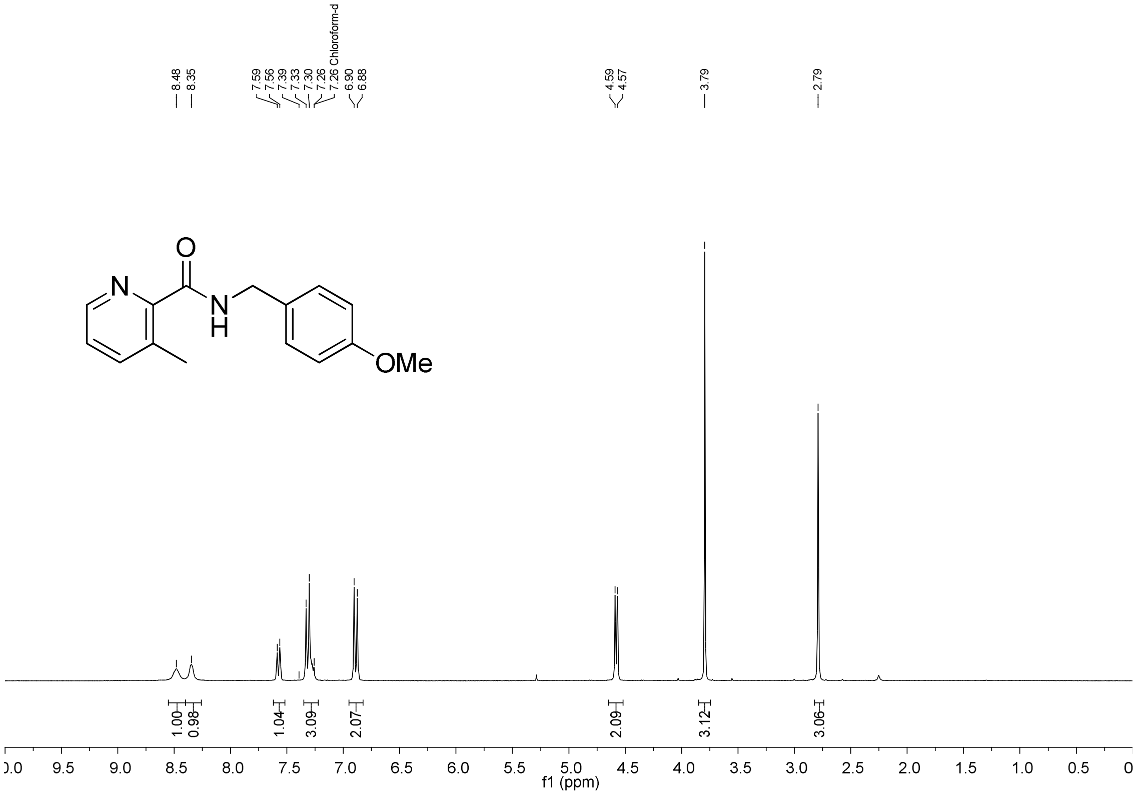

^{13}C NMR ($CDCl_3$, 75 MHz)

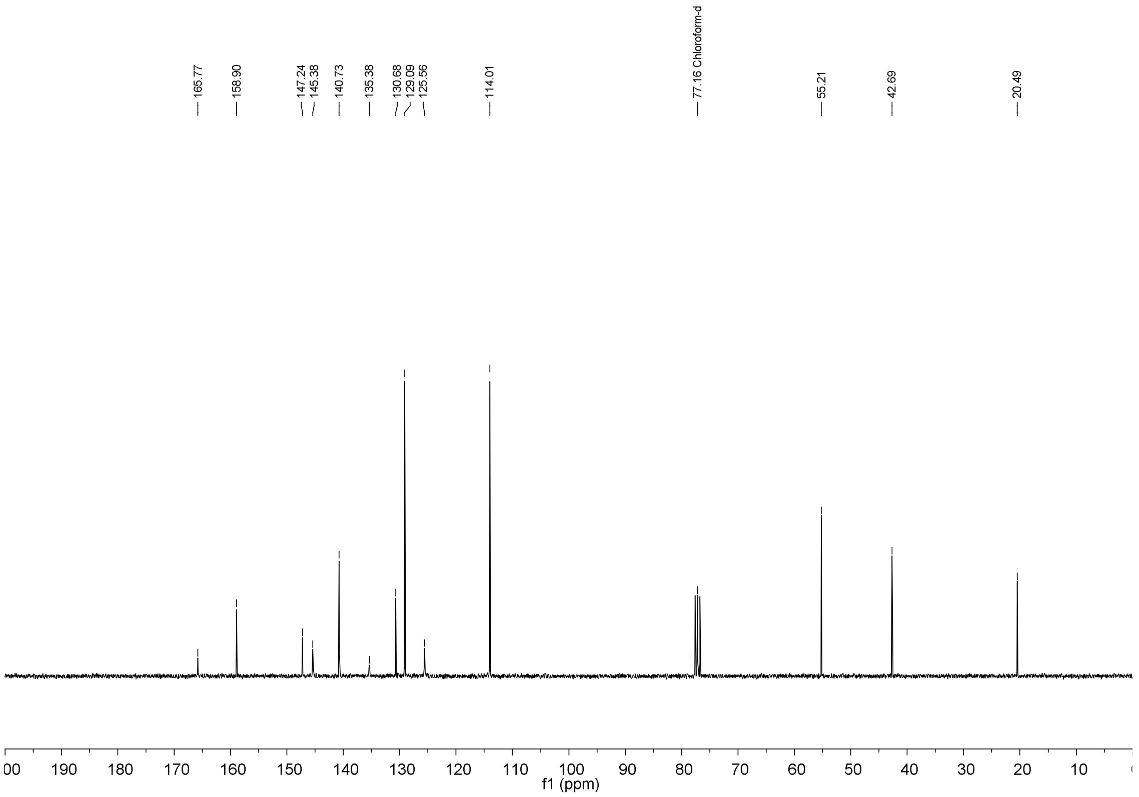

N-(4-(Methylthio)benzyl)picolinamide (20)

^{1}H NMR ($CDCl_3$, 300 MHz)

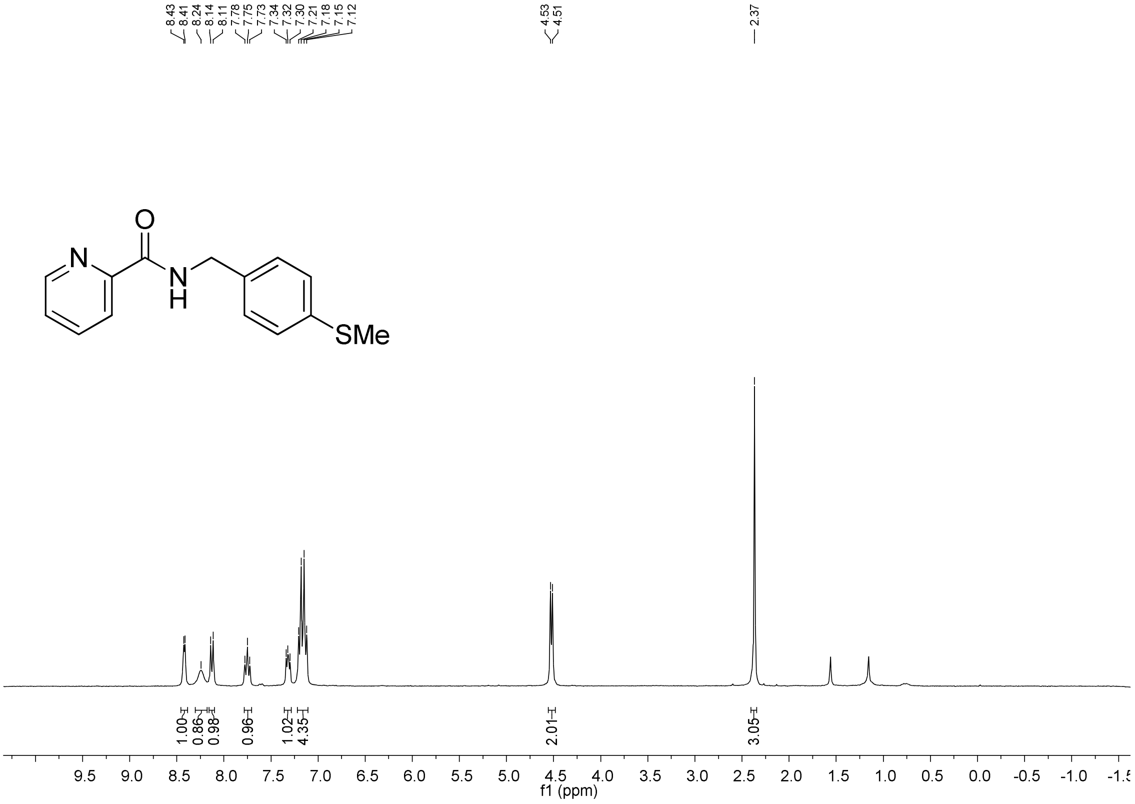

^{13}C NMR ($CDCl_3$, 75 MHz)

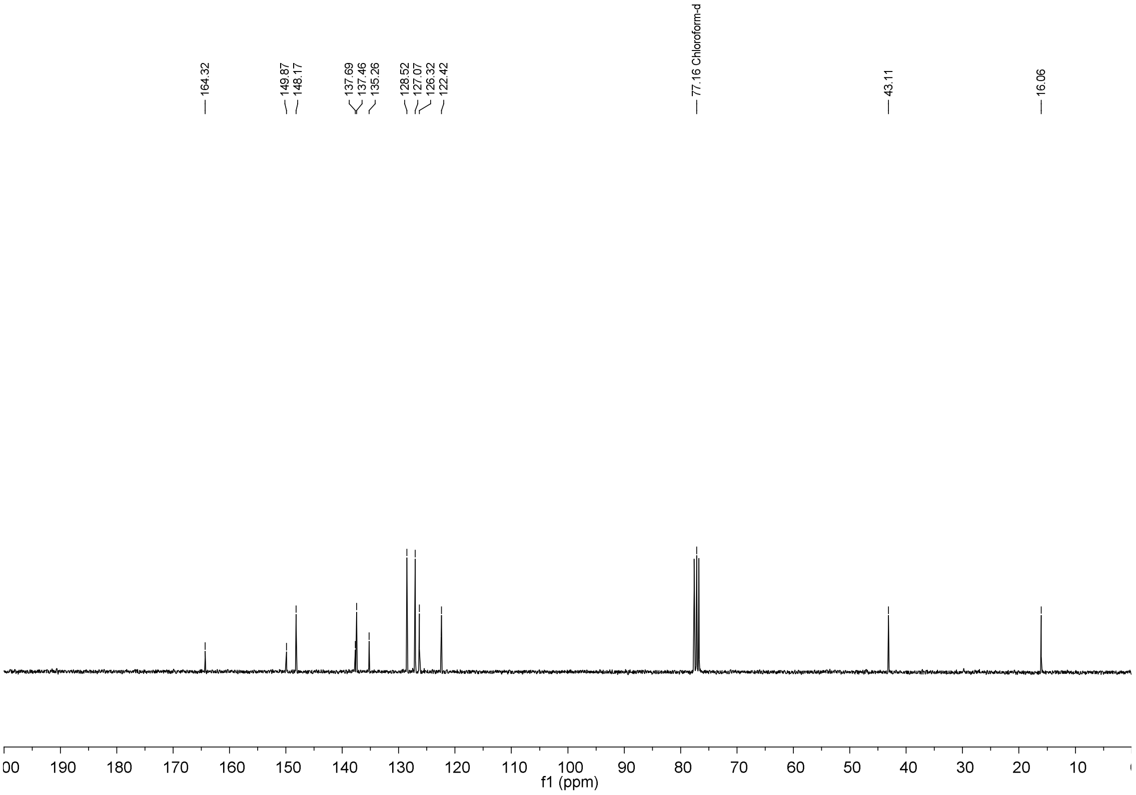

N-(4-Methoxybenzyl)picolinamide (21)

^{1}H NMR ($CDCl_3$, 300 MHz)

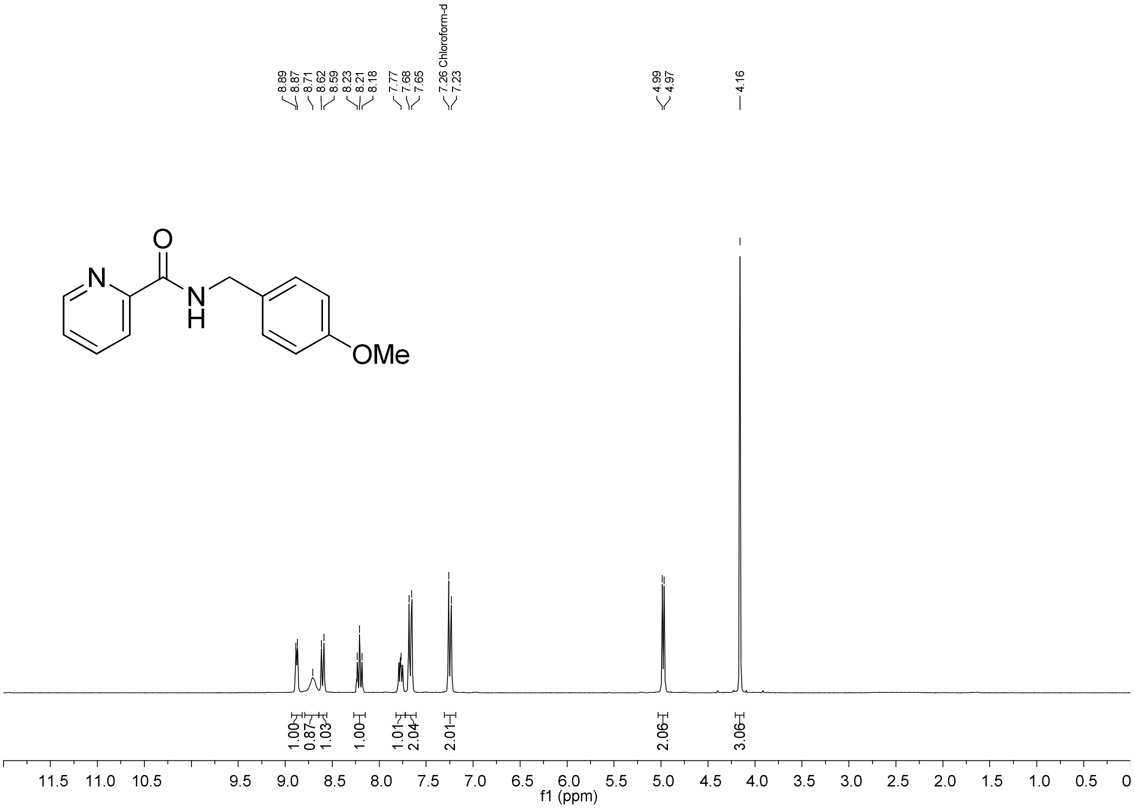

^{13}C NMR ($CDCl_3$, 75 MHz)

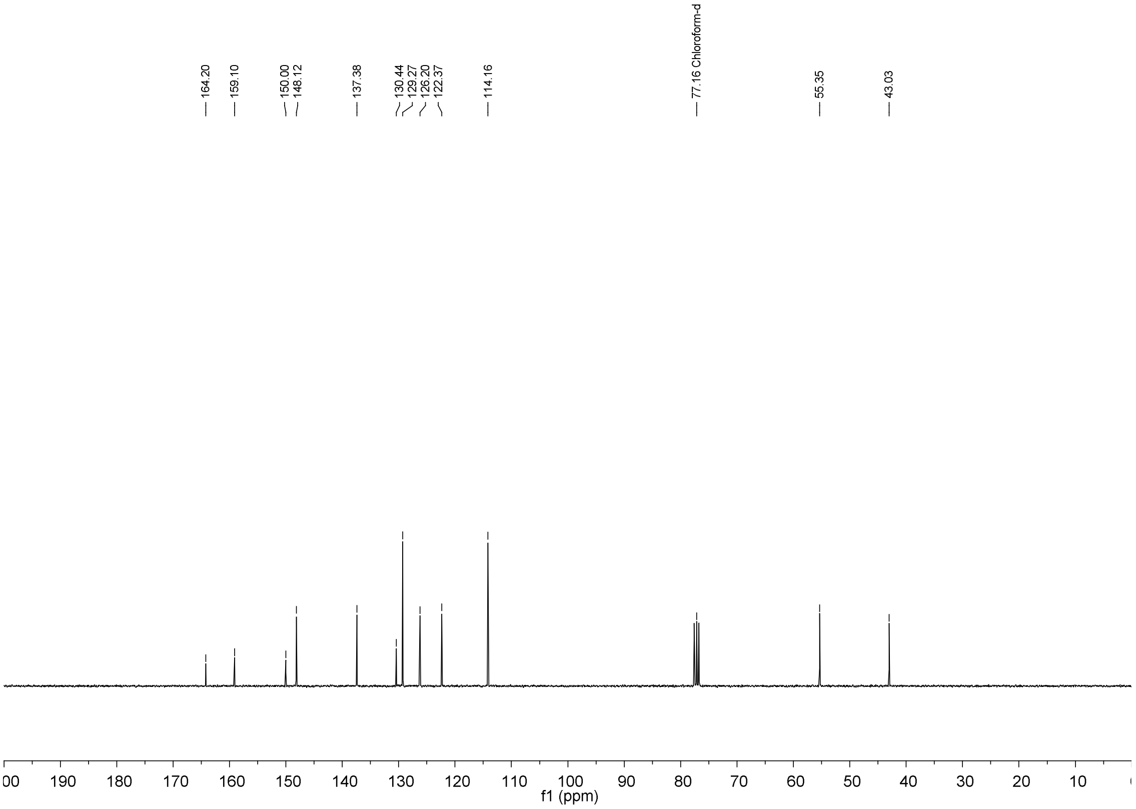

***N*-(4-Methylbenzyl)picolinamide (22)**

^{1}H NMR (acetone-d$_6$, 300 MHz)

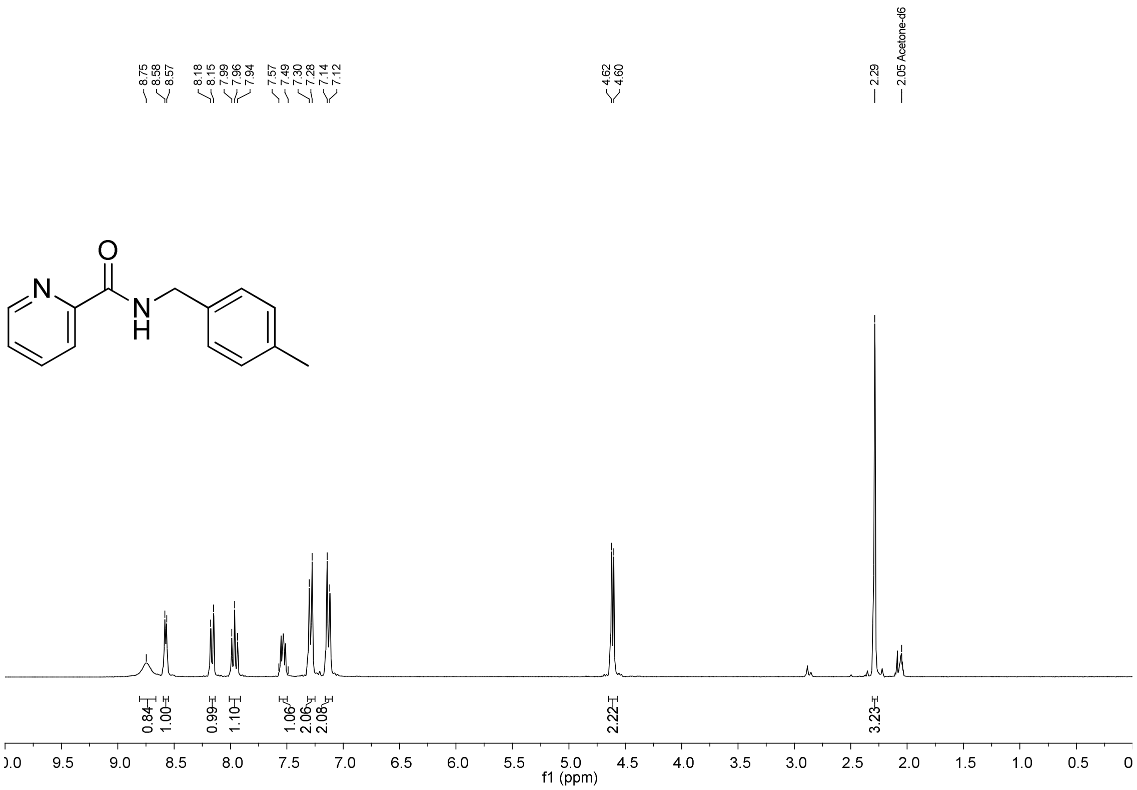

^{13}C NMR (acetone-d$_6$, 75 MHz)

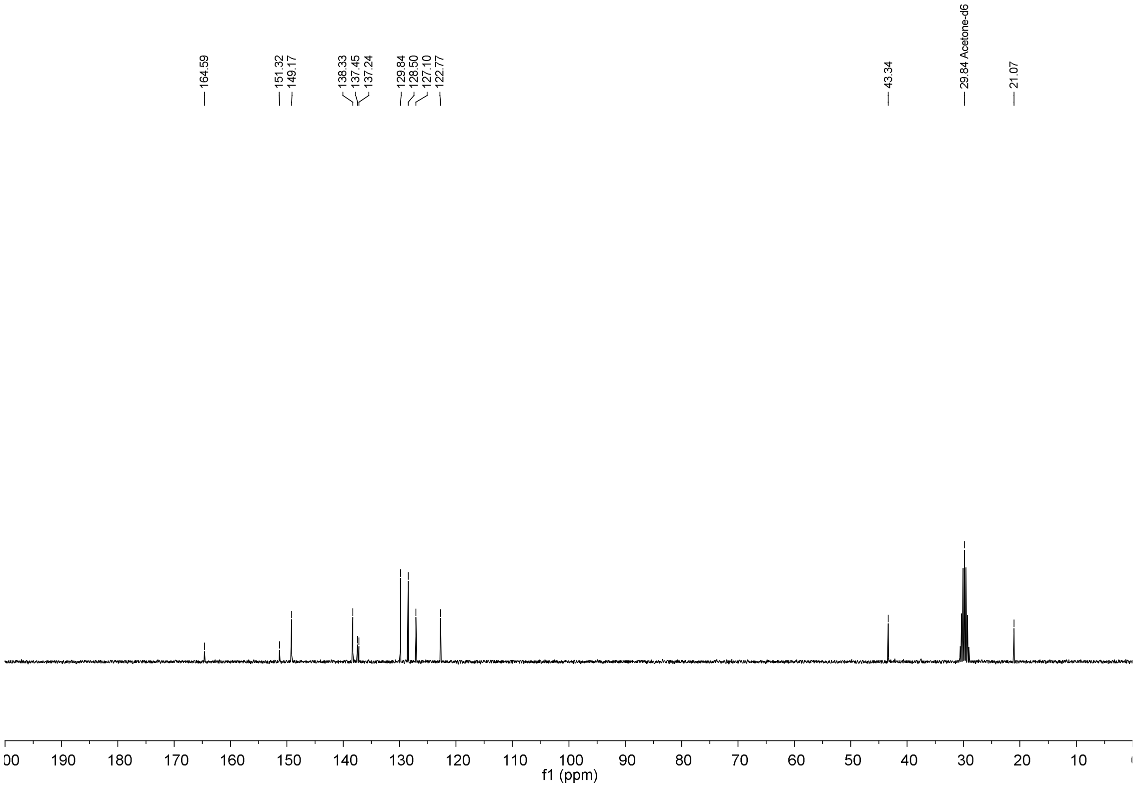

_N_-(4-Chlorobenzyl)picolinamide (23)

^{1}H NMR (acetone-d_6, 300 MHz)

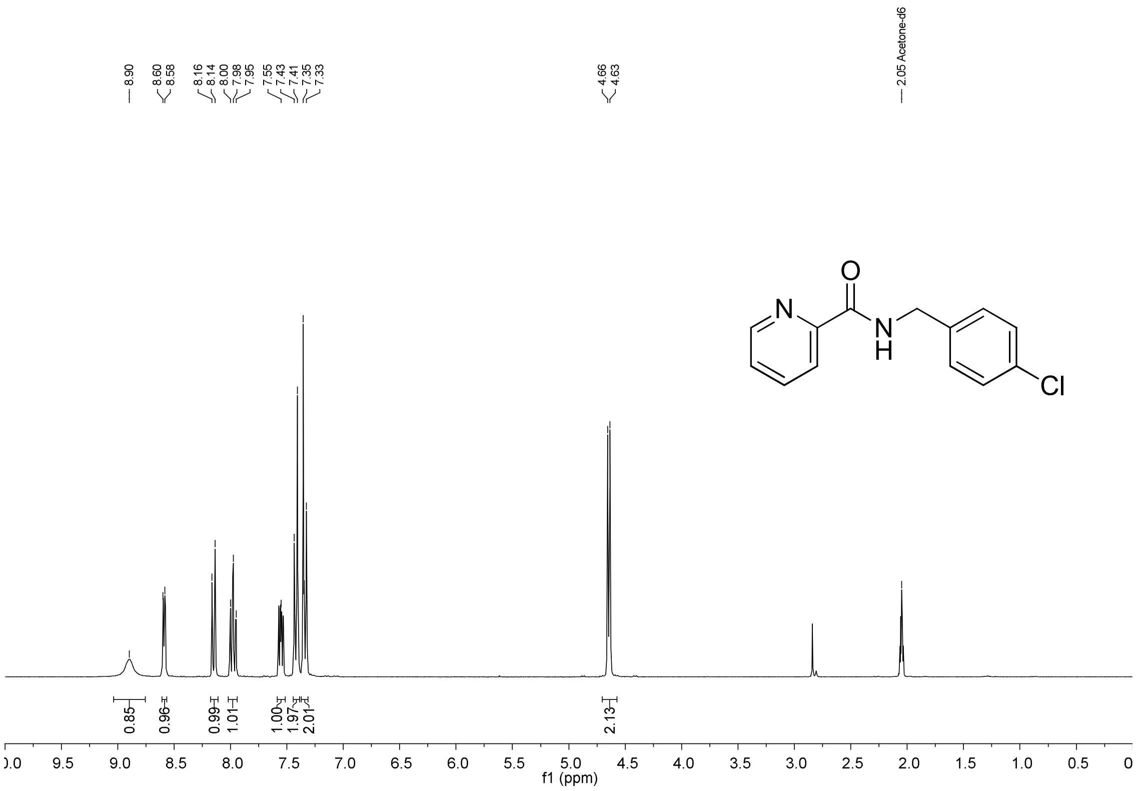

^{13}C NMR (acetone-d_6, 75 MHz)

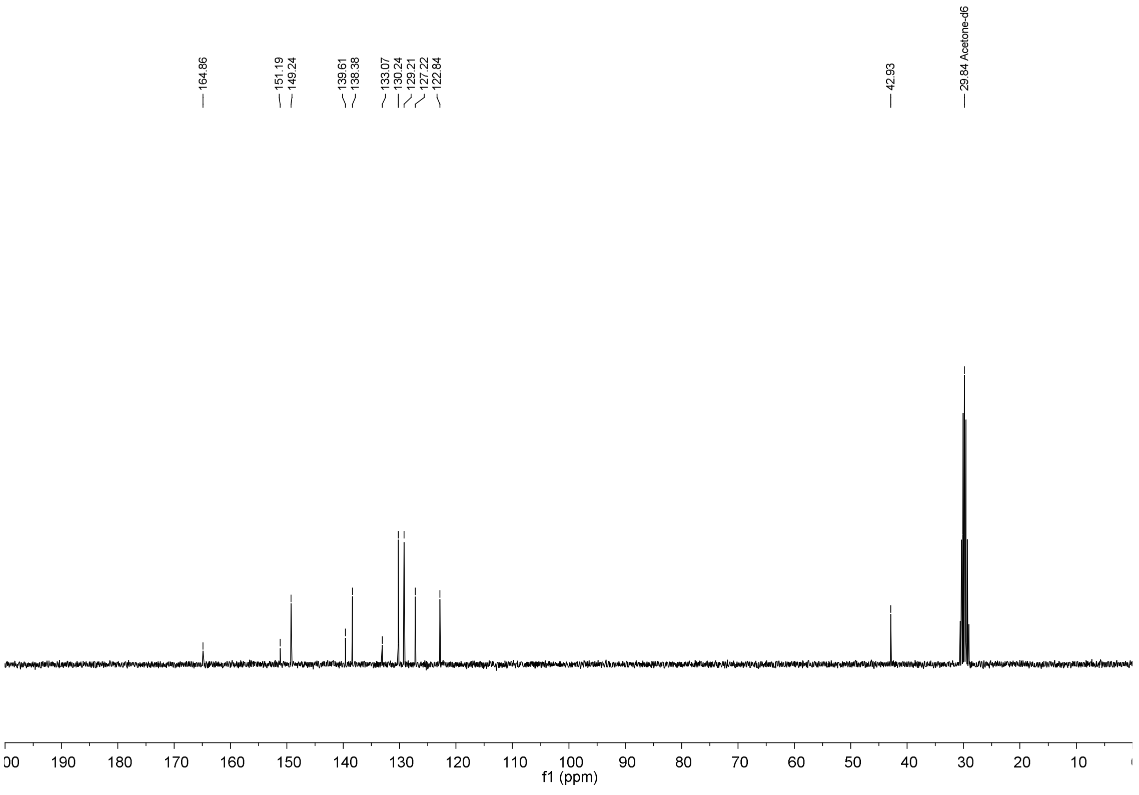

N-(4-Fluorobenzyl)picolinamide (24)

^{1}H NMR (acetone-d_6, 300 MHz)

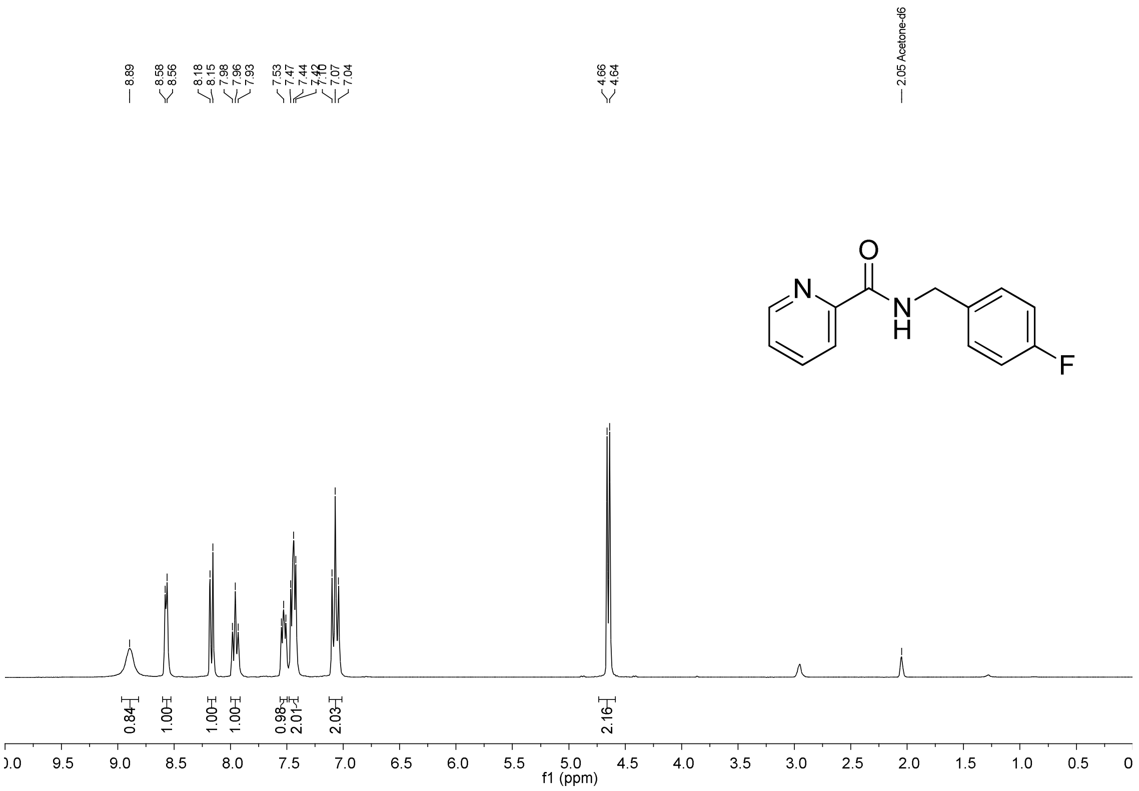

^{13}C NMR (acetone-d_6, 75 MHz)

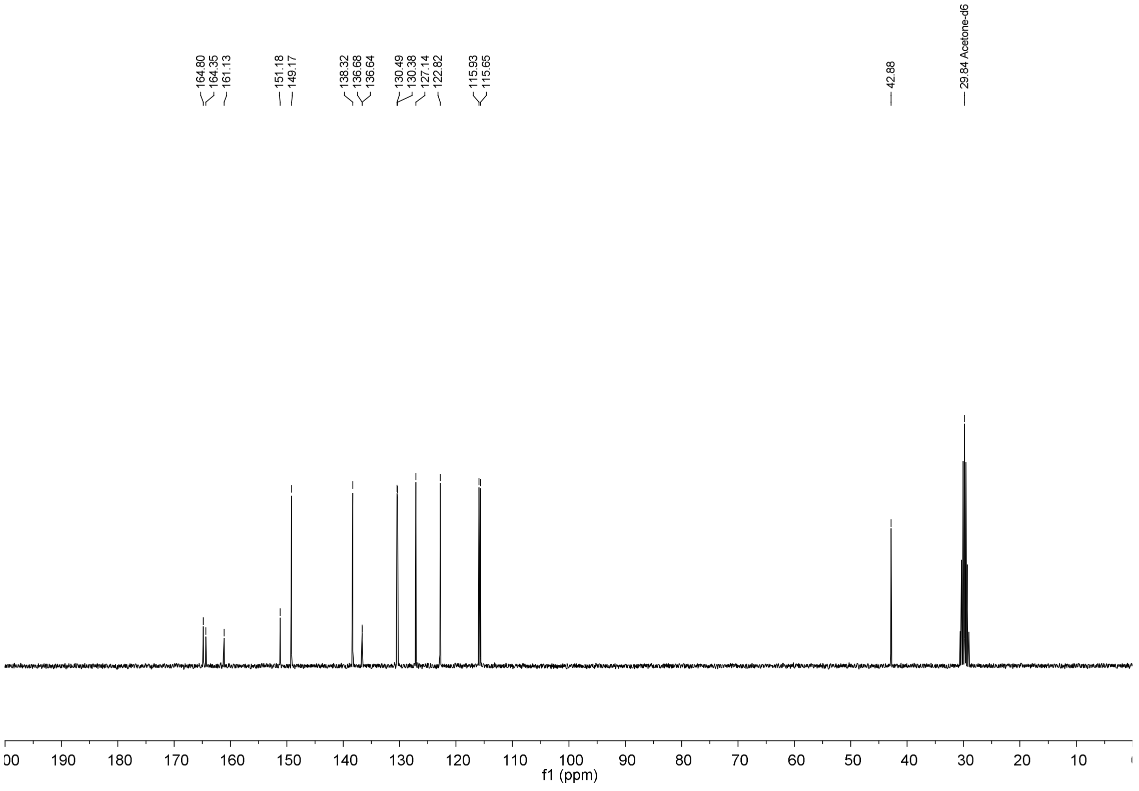

N-(4-(Trifluoromethyl)benzyl)picolinamide (25)

^{1}H NMR ($CDCl_3$, 300 MHz)

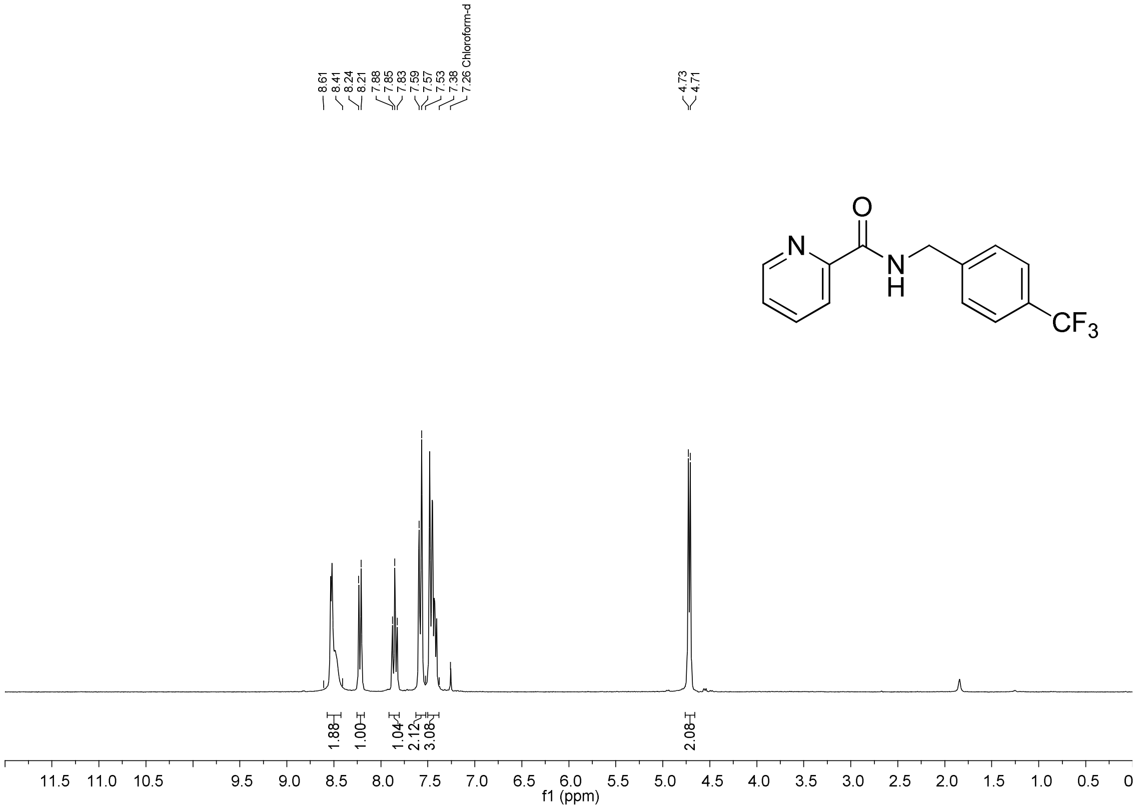

^{13}C NMR ($CDCl_3$, 75 MHz)

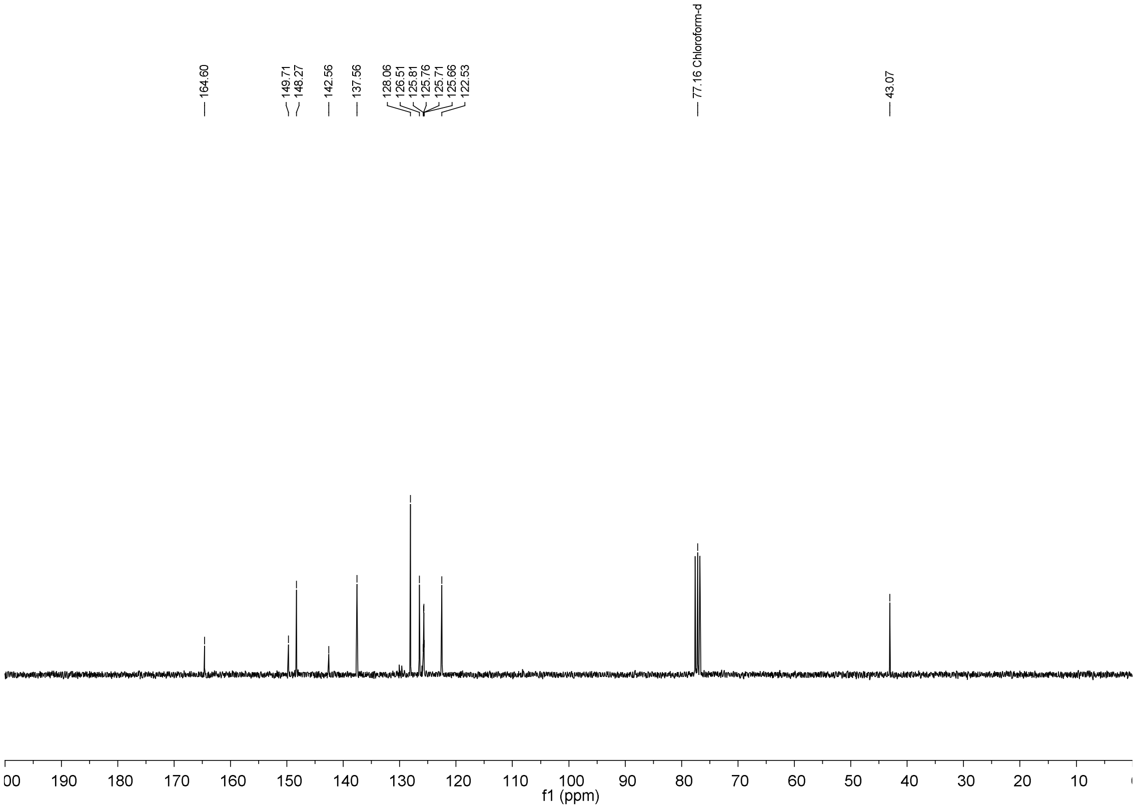

_N_-(4-Cyanobenzyl)picolinamide (26)

^{1}H NMR (acetone-d_6, 300 MHz)

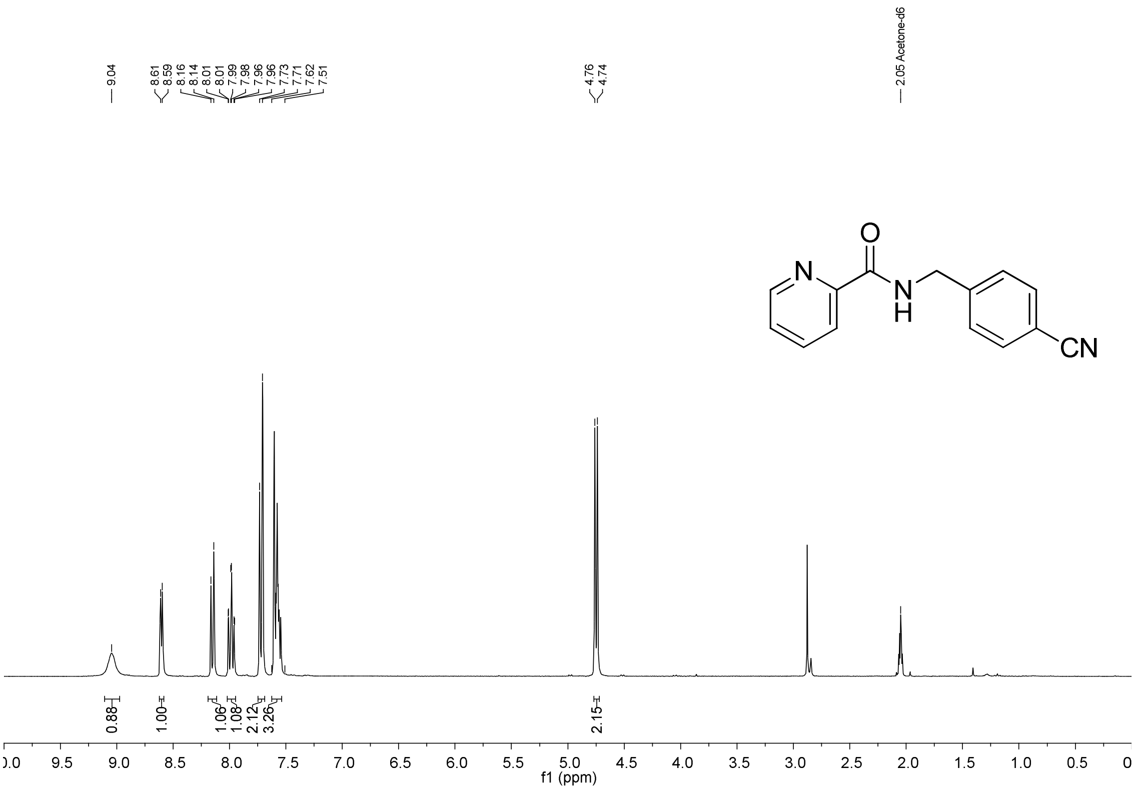

^{13}C NMR (acetone-d_6, 75 MHz)

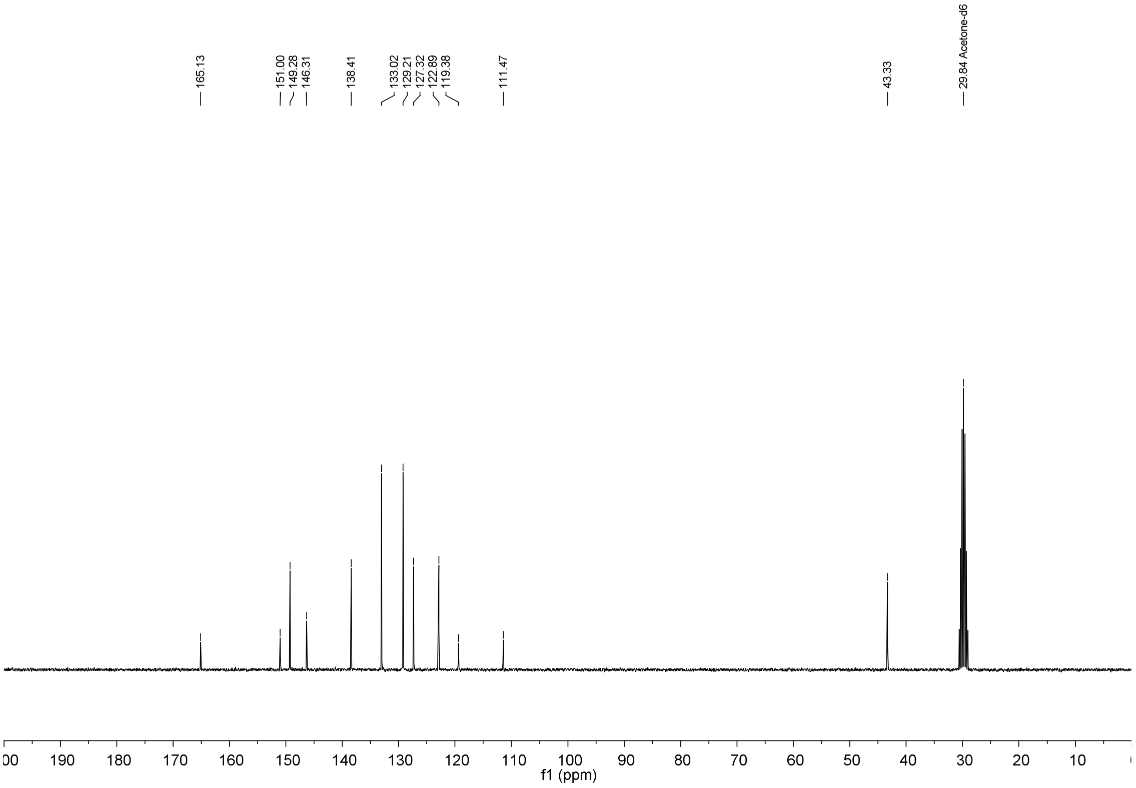

Methyl 4-(picolinamidomethyl)benzoate (27)

^{1}H NMR ($CDCl_3$, 300 MHz)

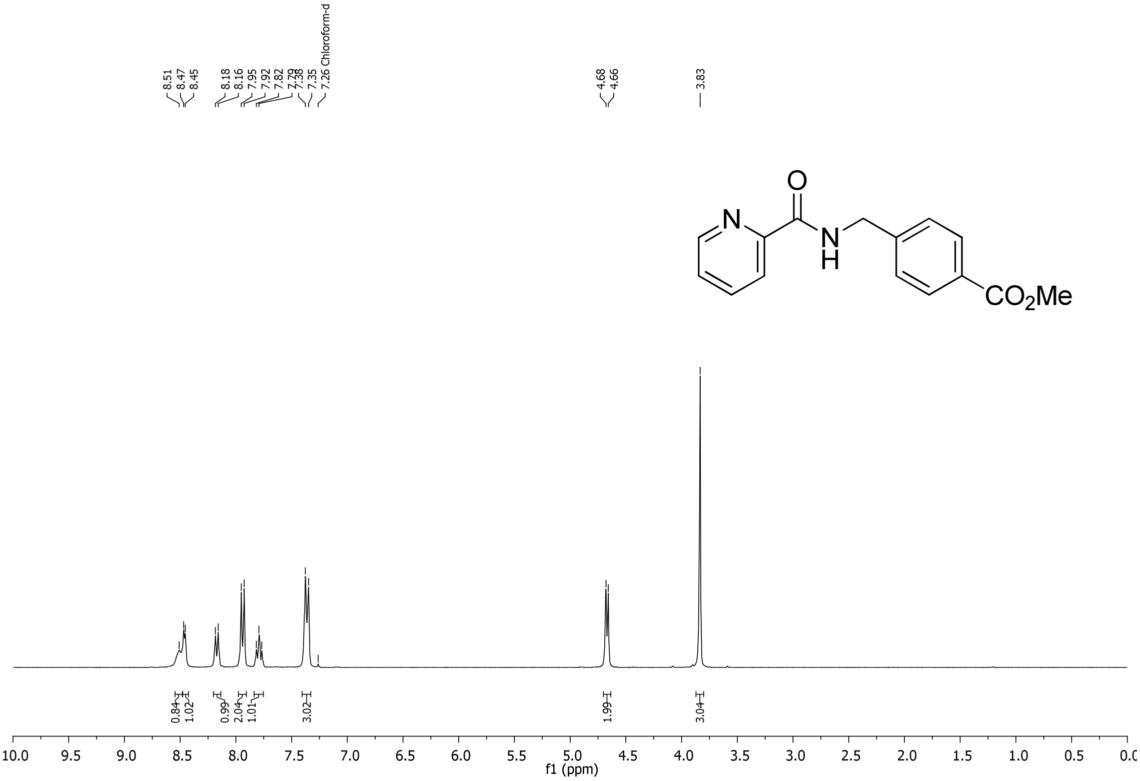

^{13}C NMR ($CDCl_3$, 75 MHz)

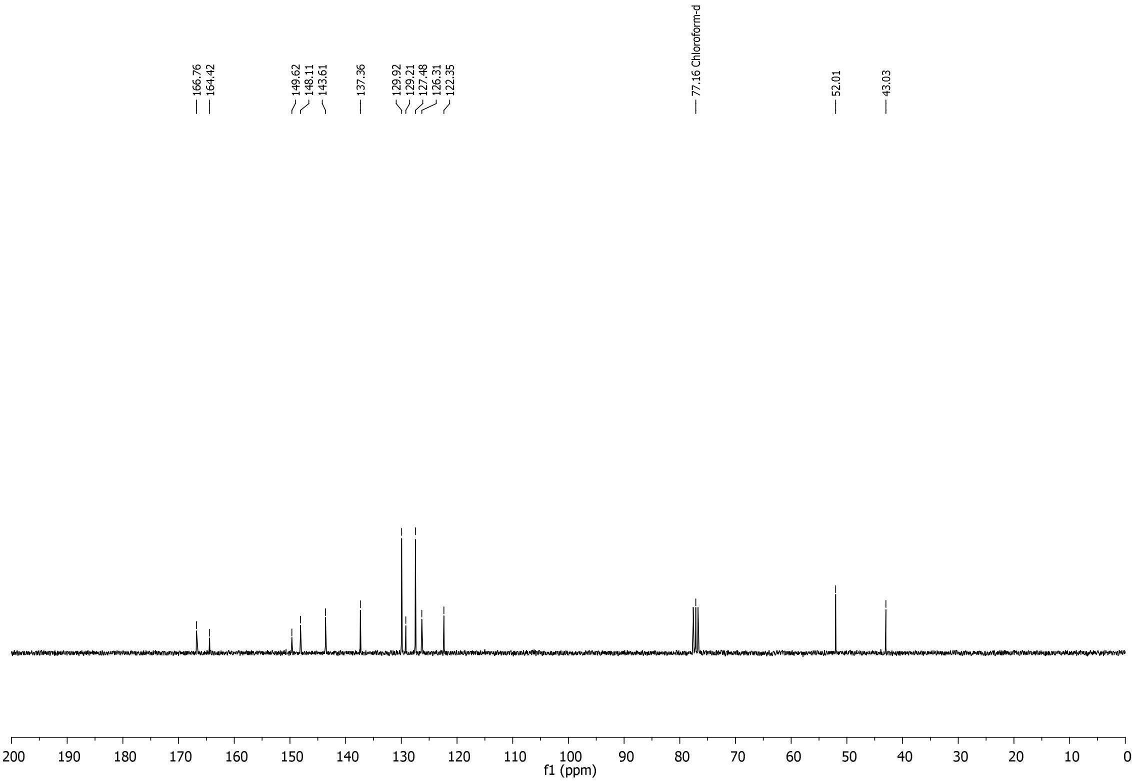

N-(3-Methylbenzyl)picolinamide (28)

^{1}H NMR (acetone-d$_6$, 300 MHz)

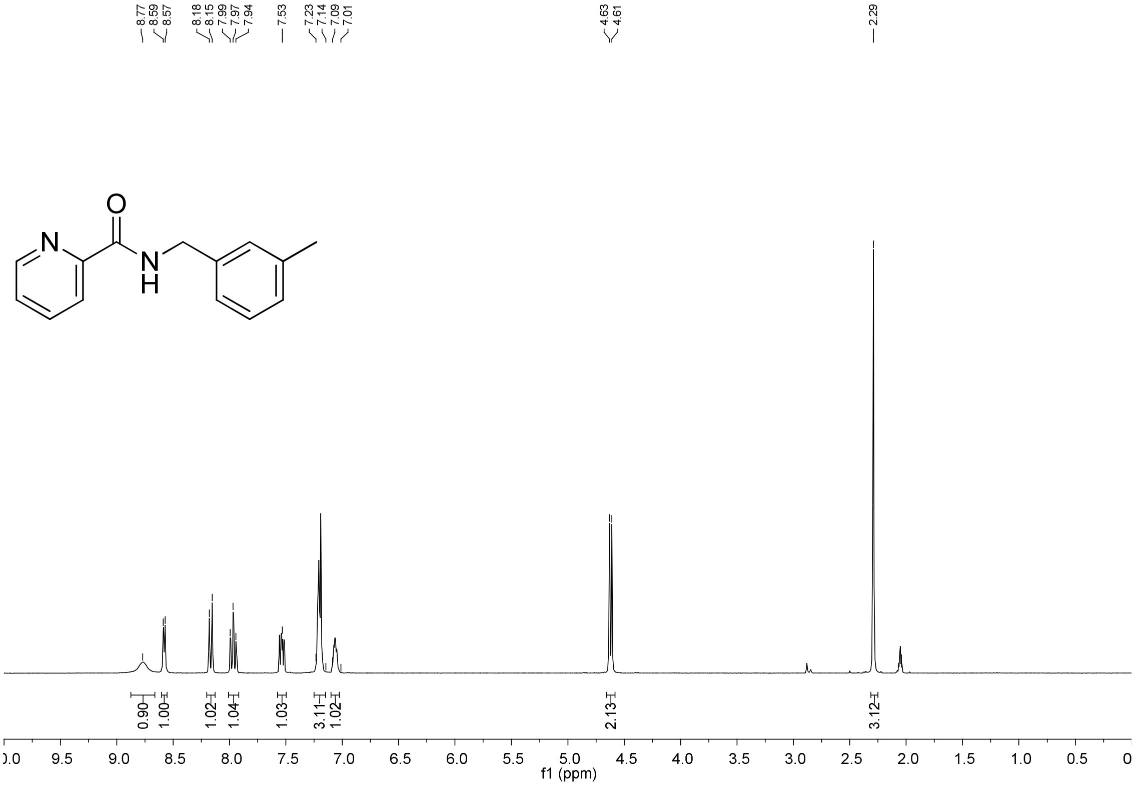

^{13}C NMR (acetone-d$_6$, 75 MHz)

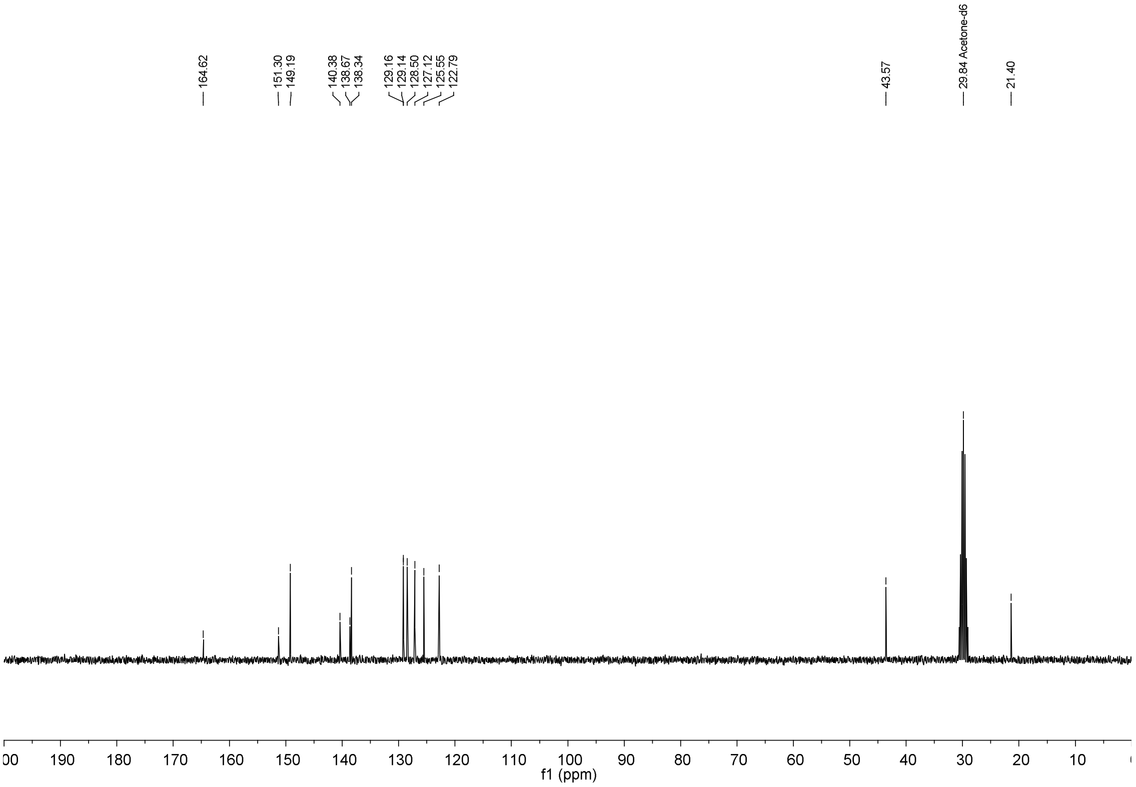

***N*-(3-(Trifluoromethyl)benzyl)picolinamide (29)**.

^{1}H NMR (acetone-d$_6$, 300 MHz)

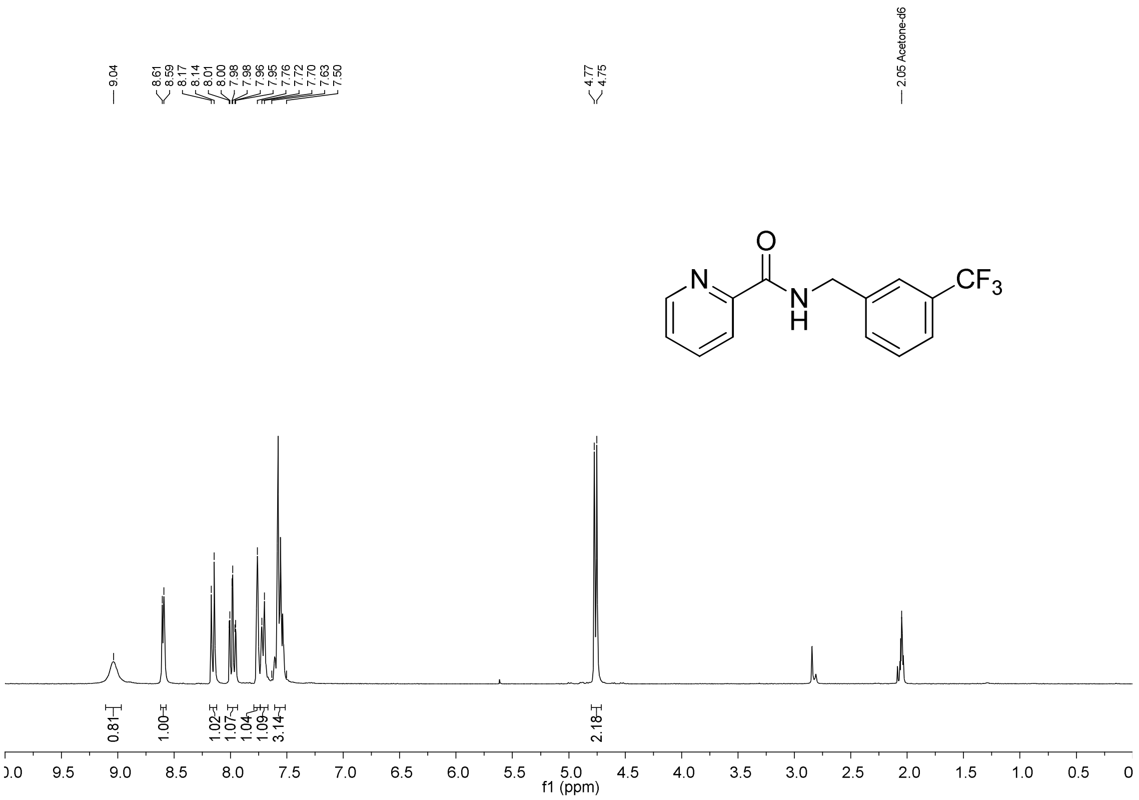

^{13}C NMR (acetone-d$_6$, 75 MHz)

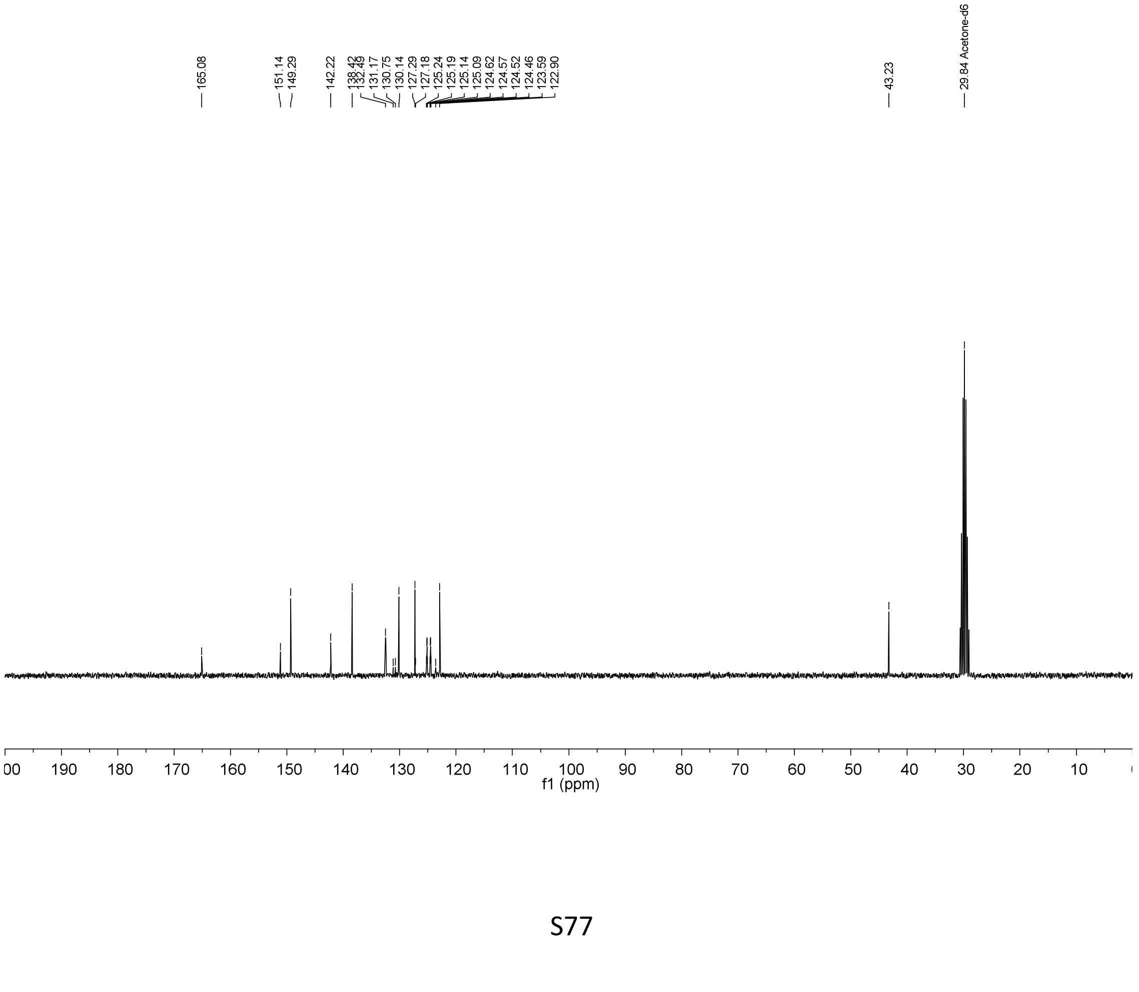

***N*-(2-Methylbenzyl)picolinamide (30)**

^{1}H NMR (acetone-d$_6$, 300 MHz)

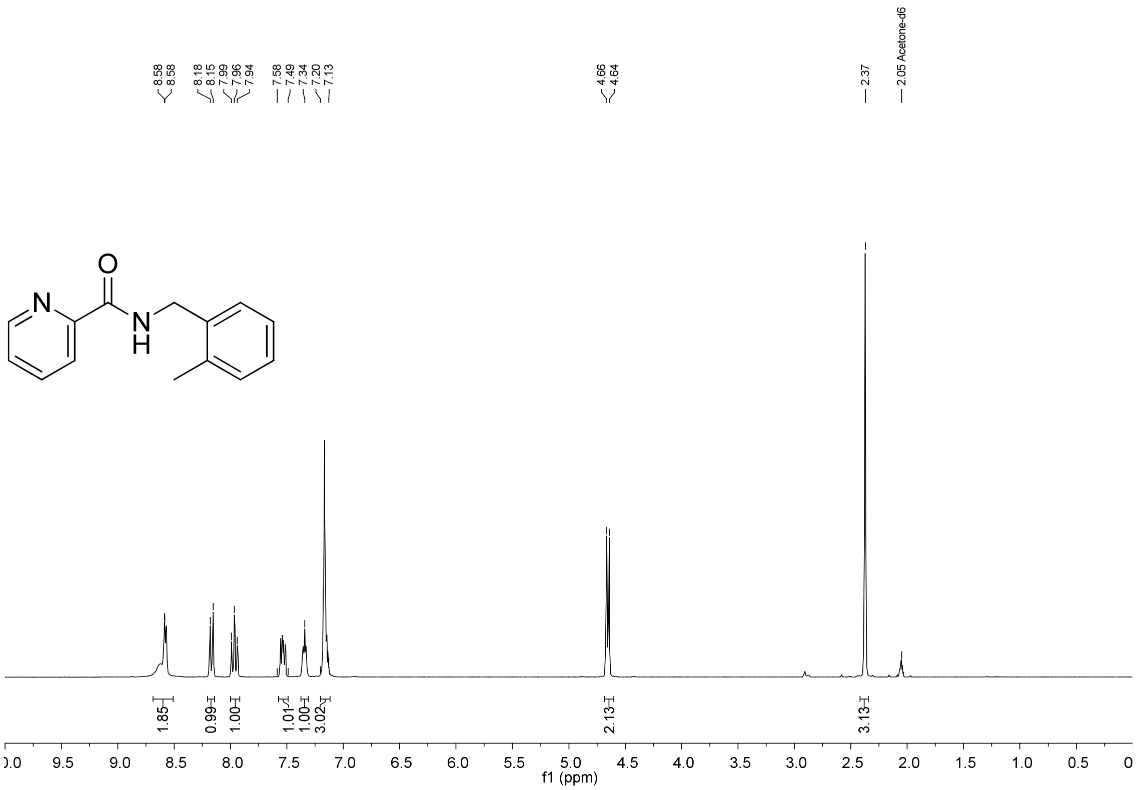

^{13}C NMR (acetone-d$_6$, 75 MHz)

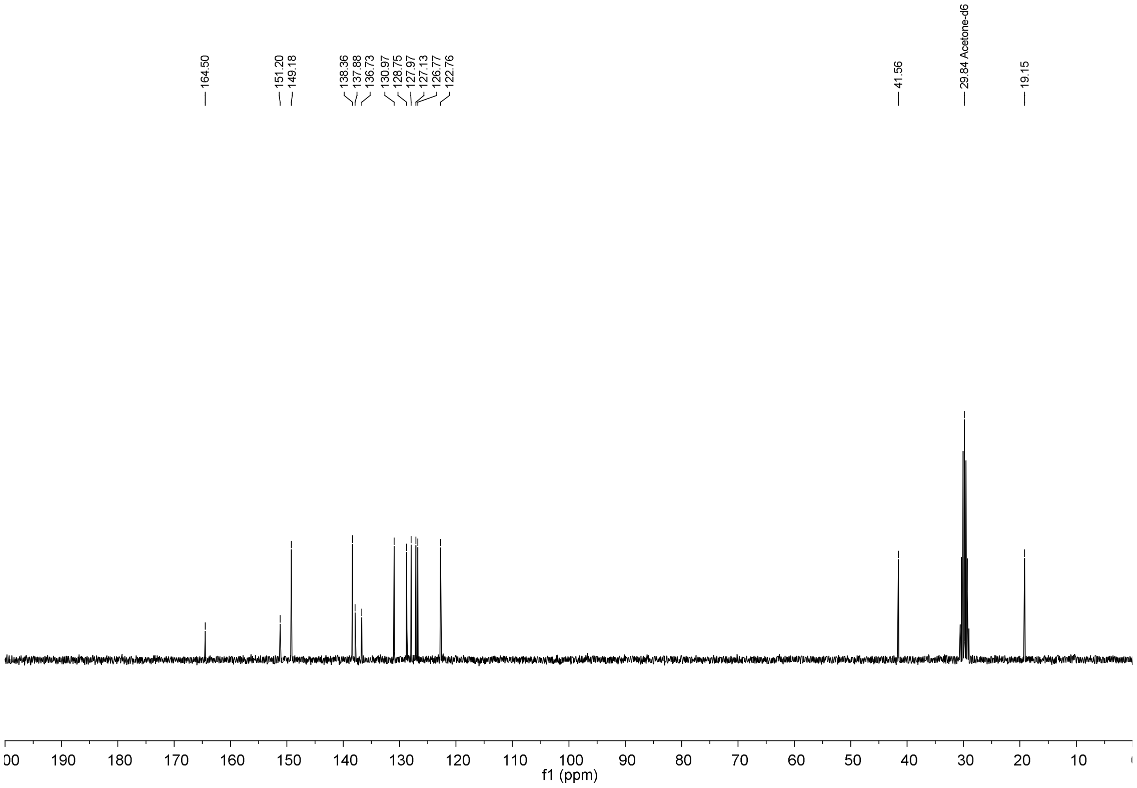

_N_-(2-Bromobenzyl)picolinamide (31)

^{1}H NMR (acetone-d$_6$, 300 MHz)

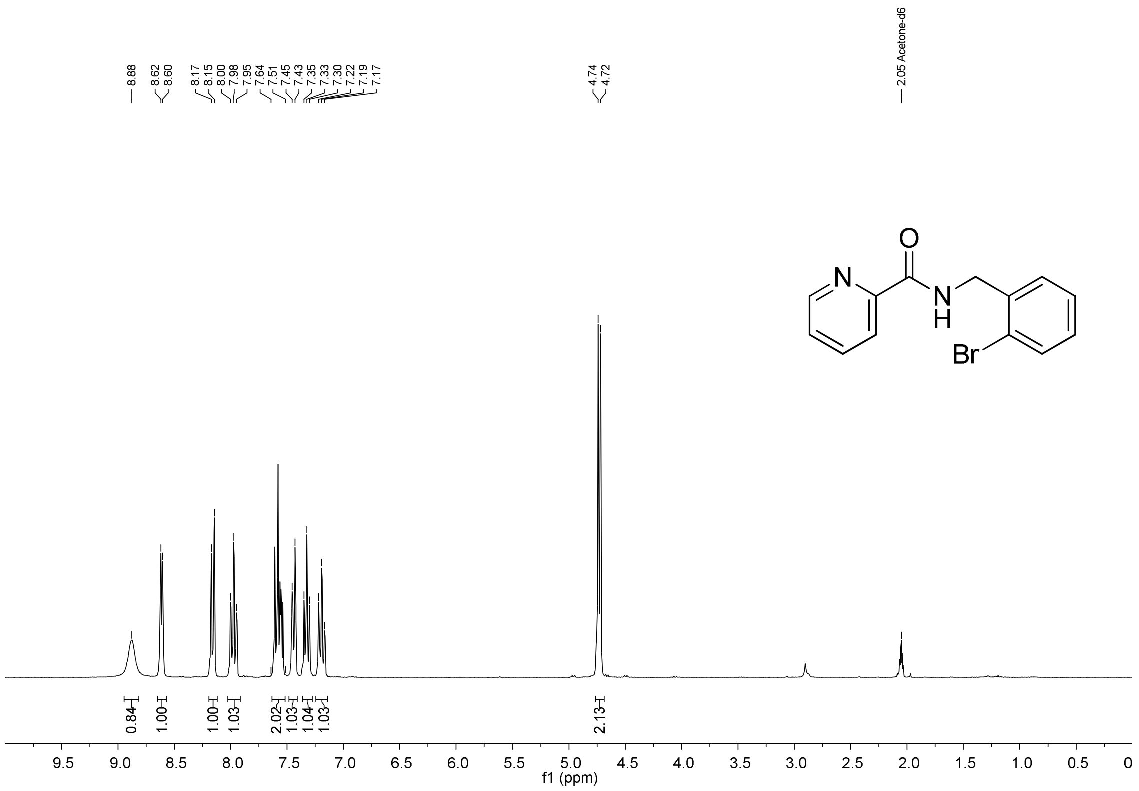

^{13}C NMR (acetone-d$_6$, 75 MHz)

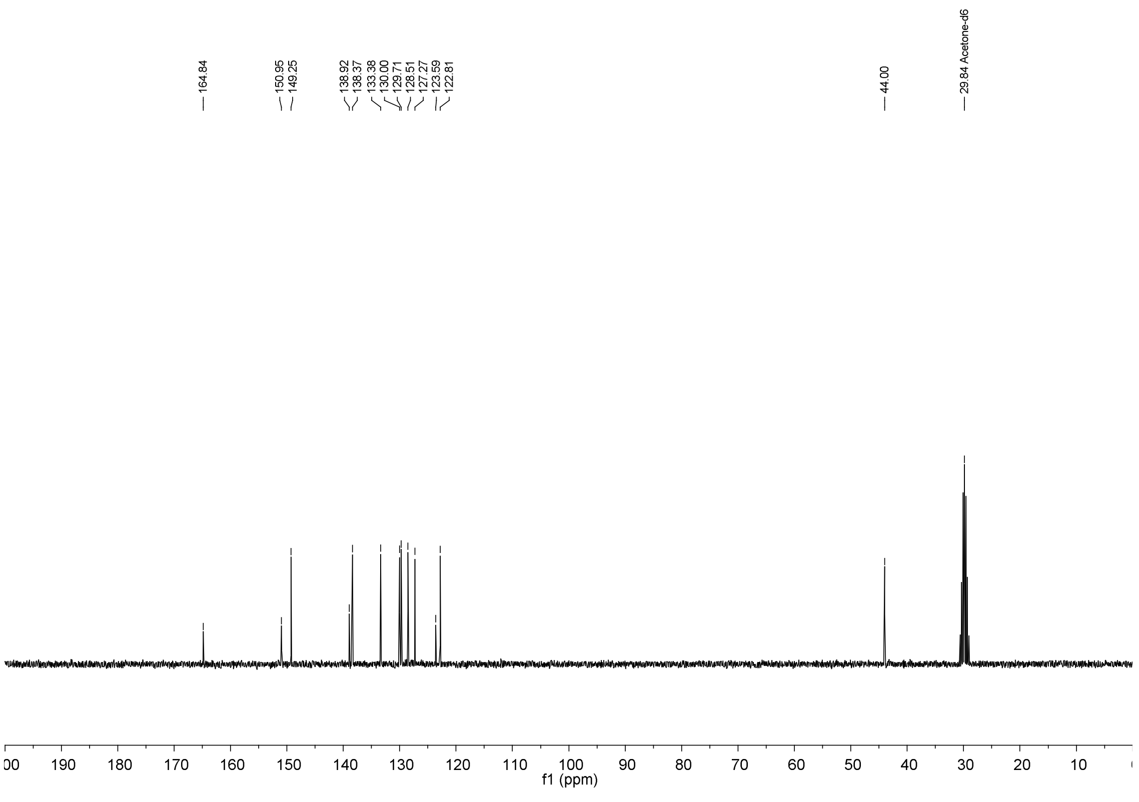

N-(2-Fluorobenzyl)picolinamide (32)

^{1}H NMR (acetone-d$_6$, 300 MHz)

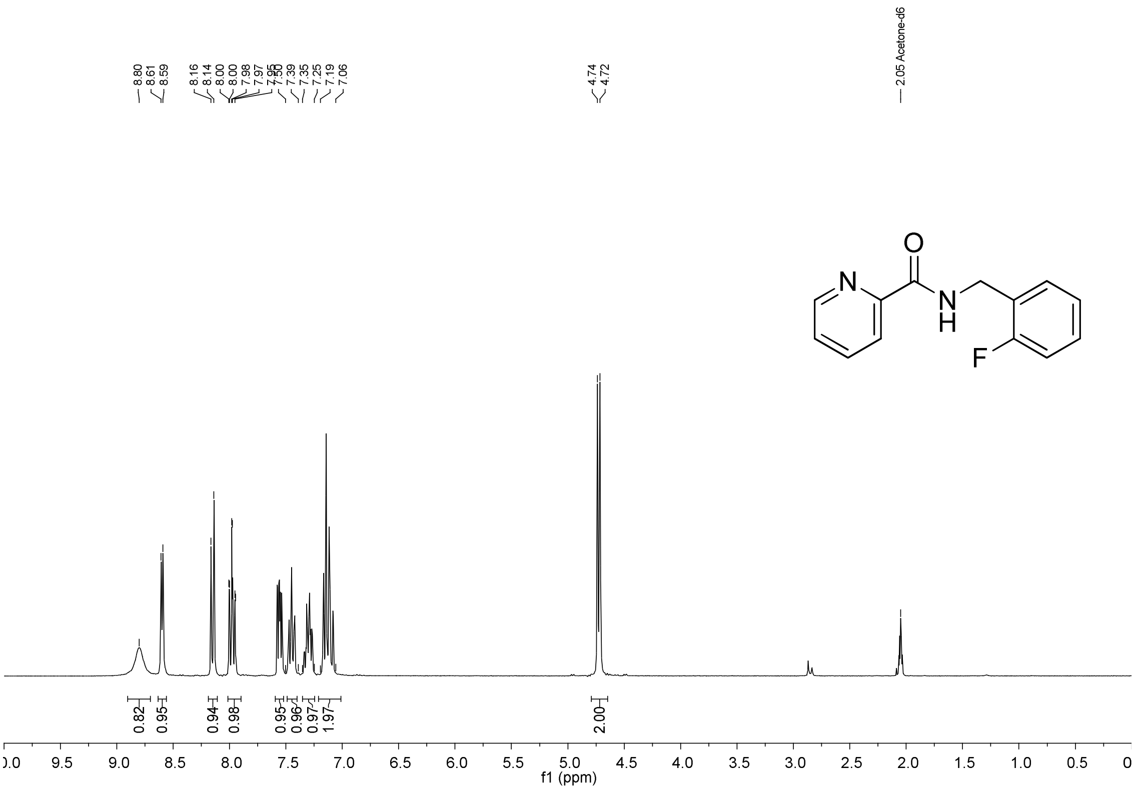

^{13}C NMR (acetone-d$_6$, 75 MHz)

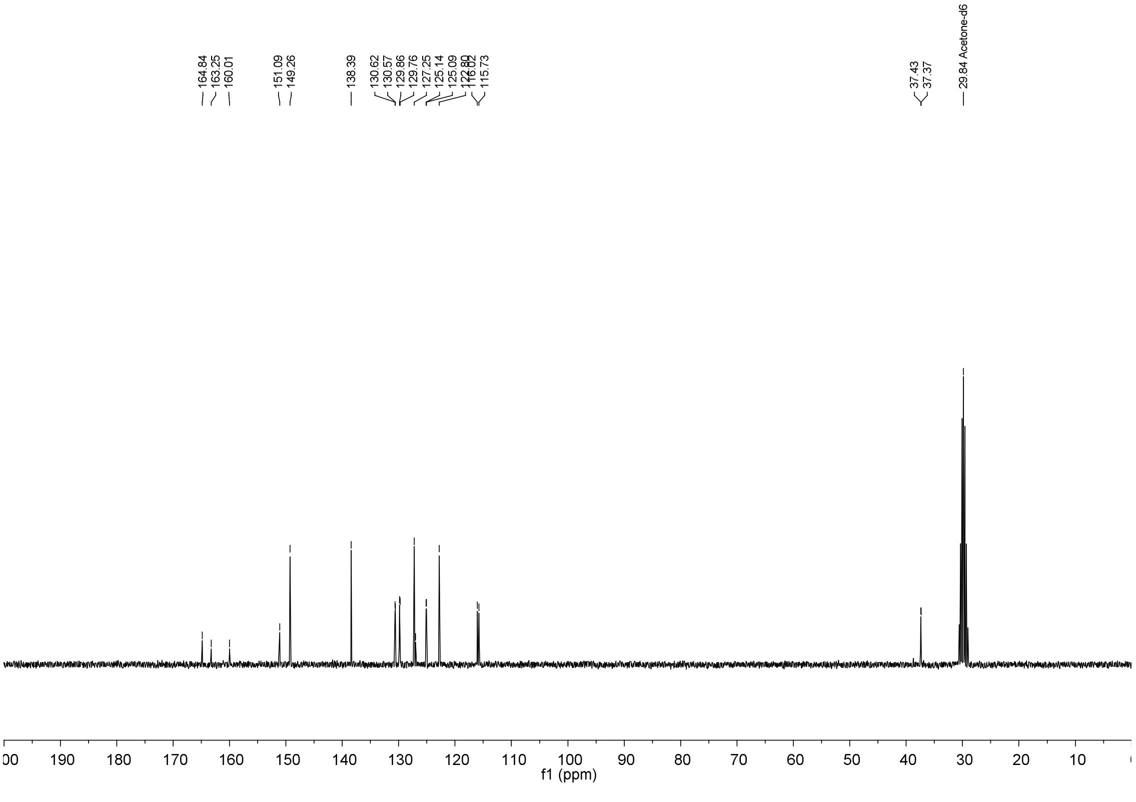

_N_-(Furan-2-ylmethyl)picolinamide (33)

^{1}H NMR ($CDCl_3$, 300 MHz)

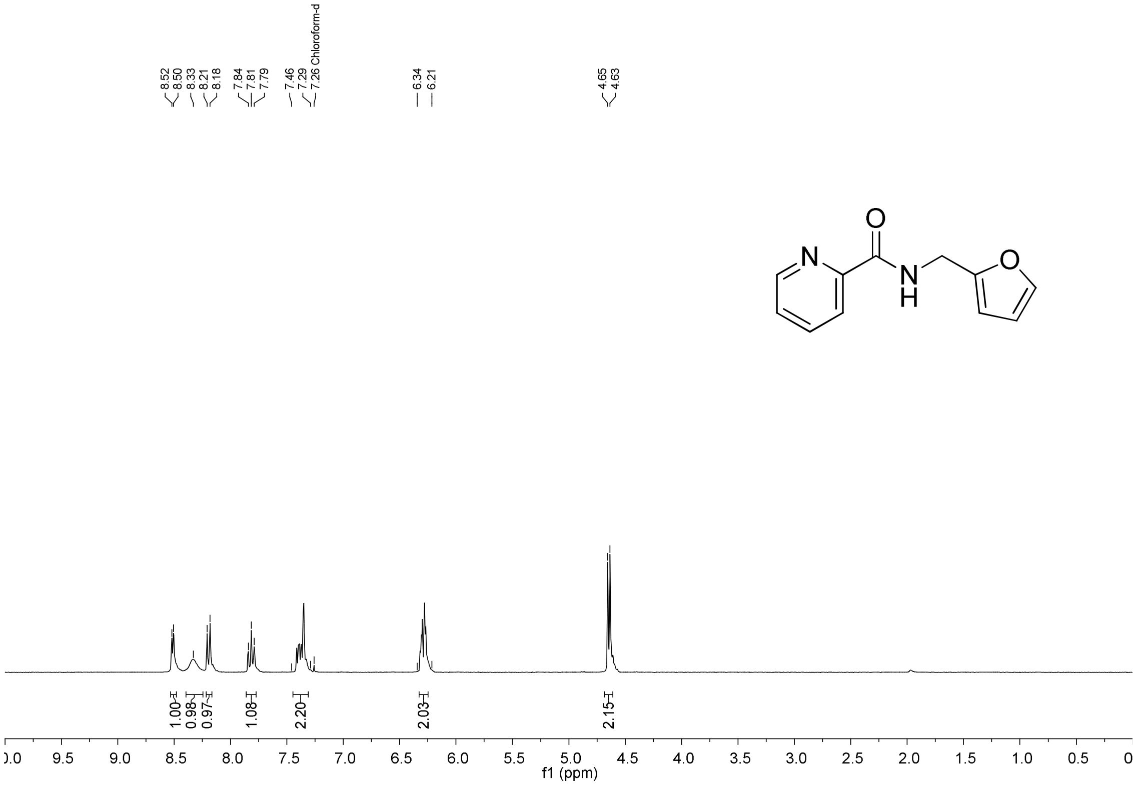

^{13}C NMR ($CDCl_3$, 75 MHz)

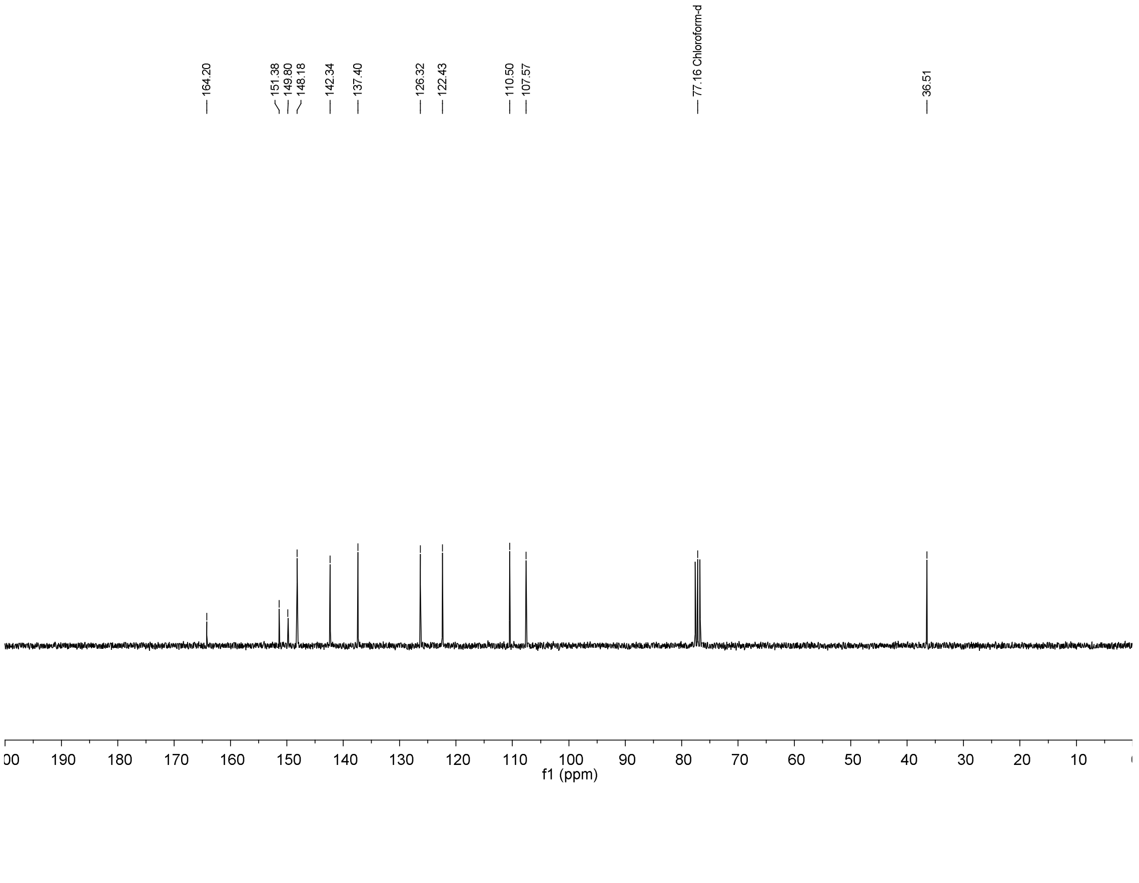

***N*-Benzylquinoline-2-carboxamide (8)**

^{1}H NMR ($CDCl_3$, 300 MHz)

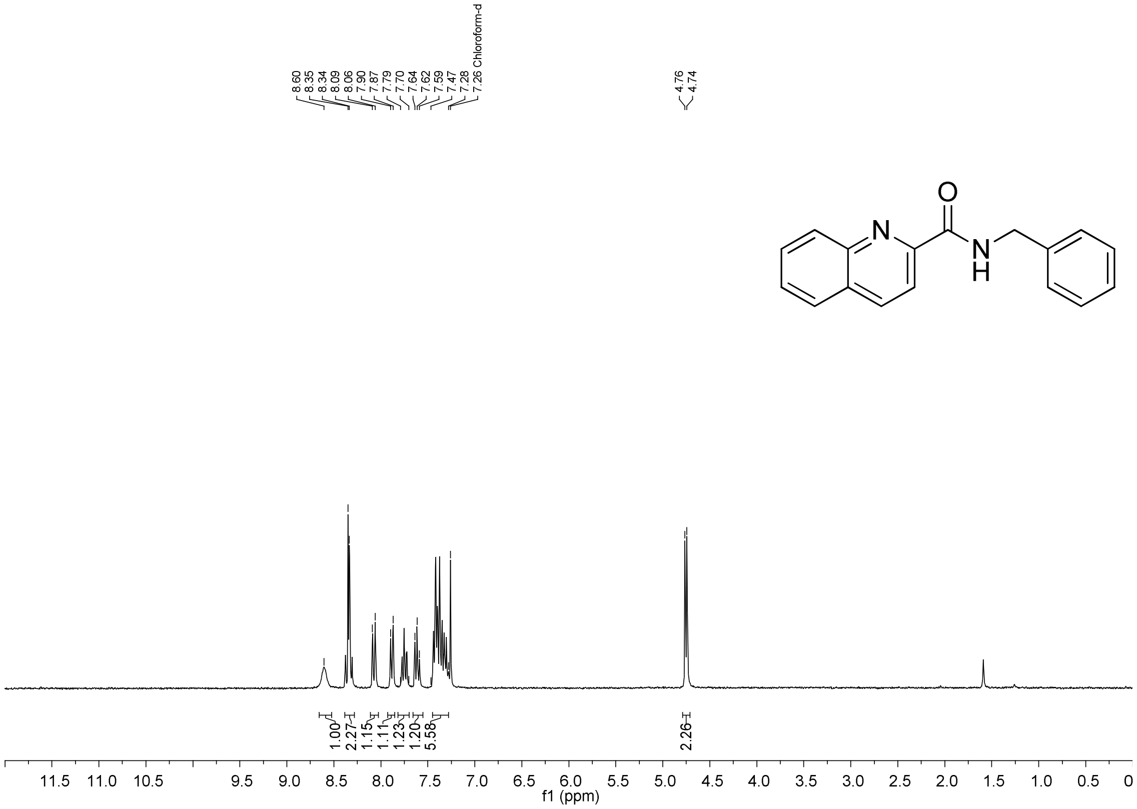

^{13}C NMR ($CDCl_3$, 75 MHz)

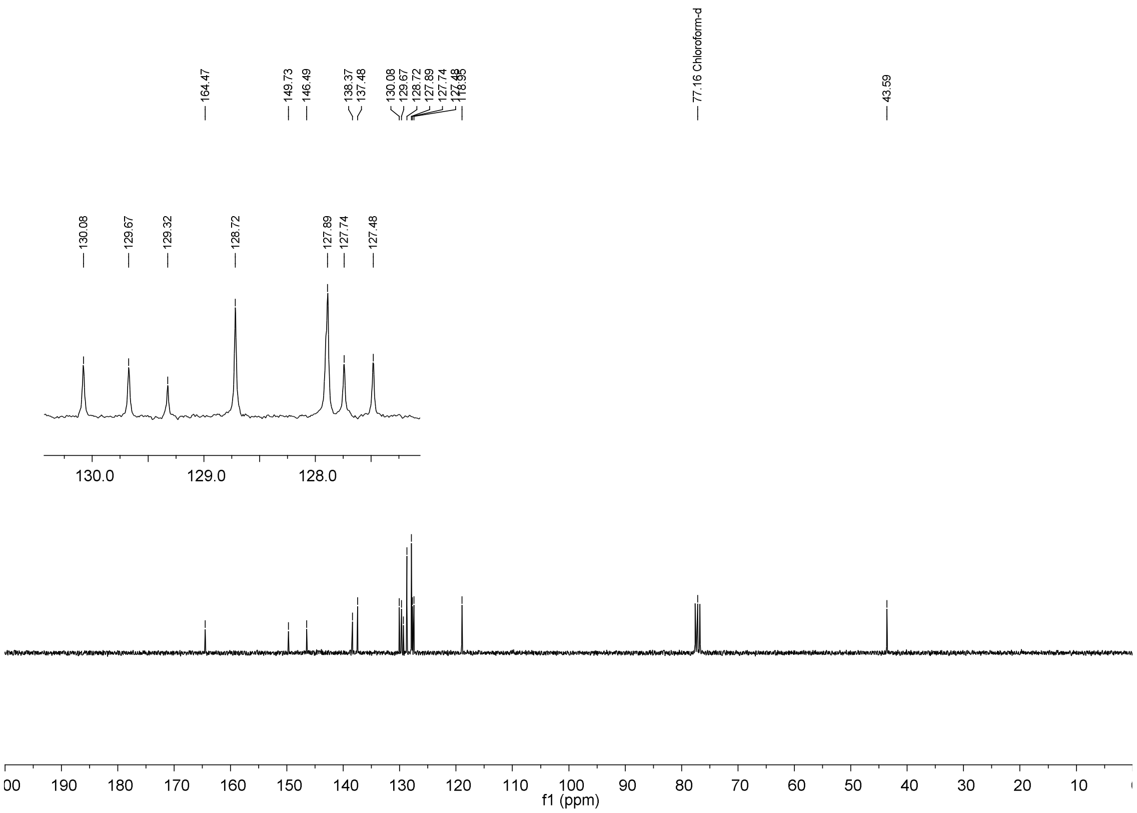

***N*-Benzyl-5-methylthiophene-2-carboxamide (9)**

^{1}H NMR (acetone-d$_6$, 300 MHz)

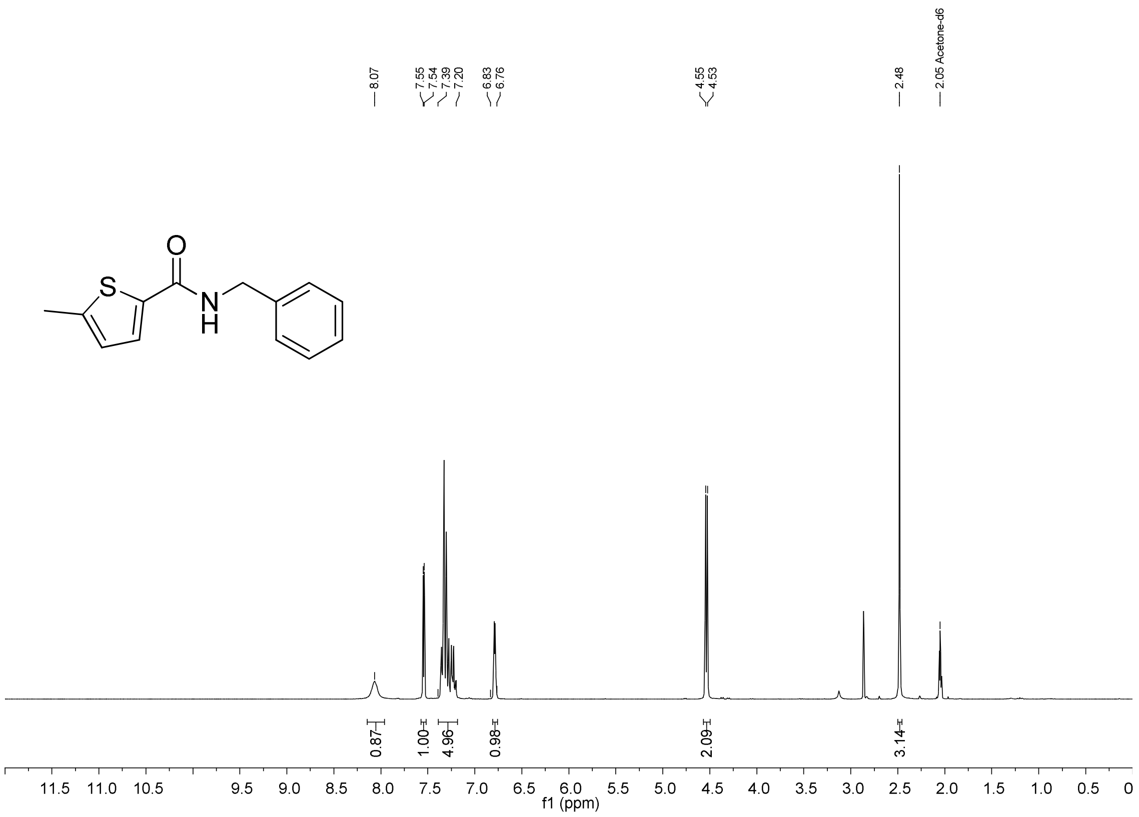

^{13}C NMR (CDCl$_3$, 75 MHz)

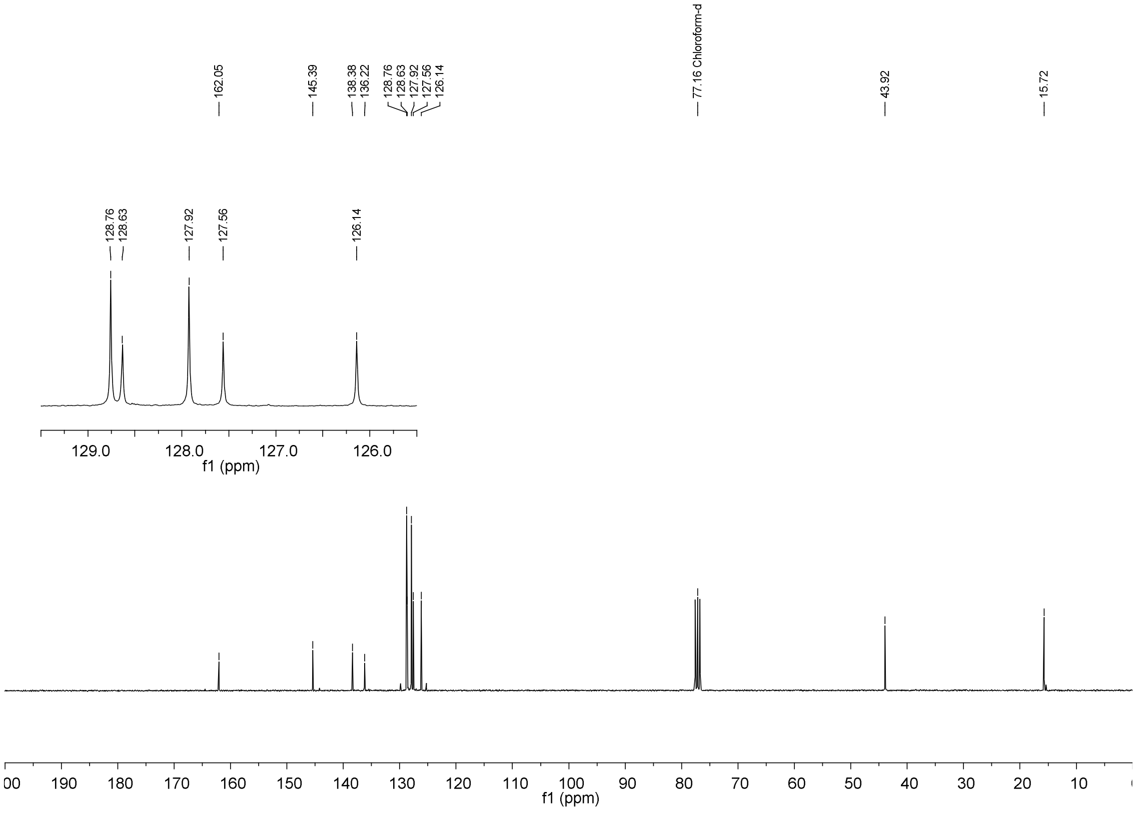

***N*-Benzylbenzo[*b*]thiophene-2-carboxamide (10)**

^{1}H NMR ($CDCl_3$, 300 MHz)

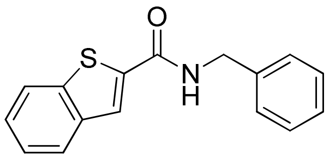

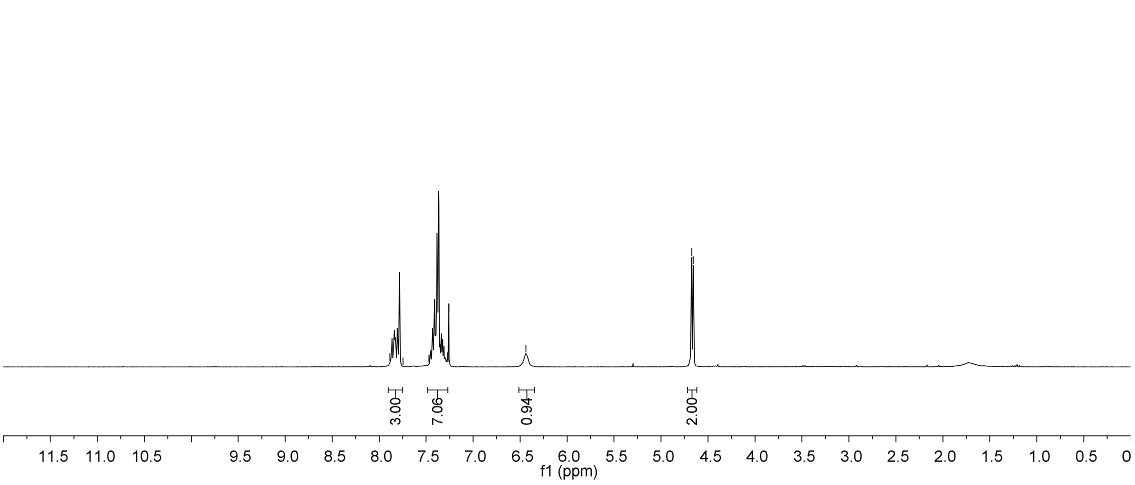

^{13}C NMR ($CDCl_3$, 75 MHz)

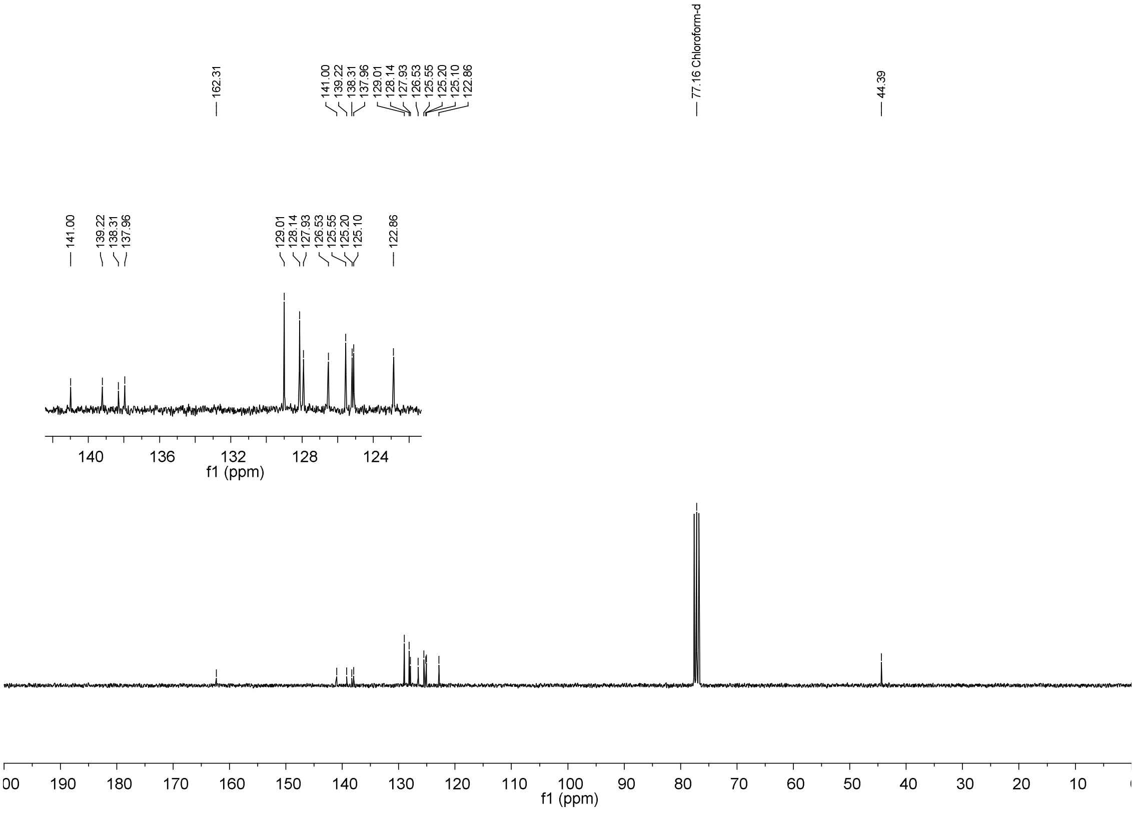

***N*-Phenethylpicolinamide (61)**

^{1}H NMR ($CDCl_3$, 300 MHz)

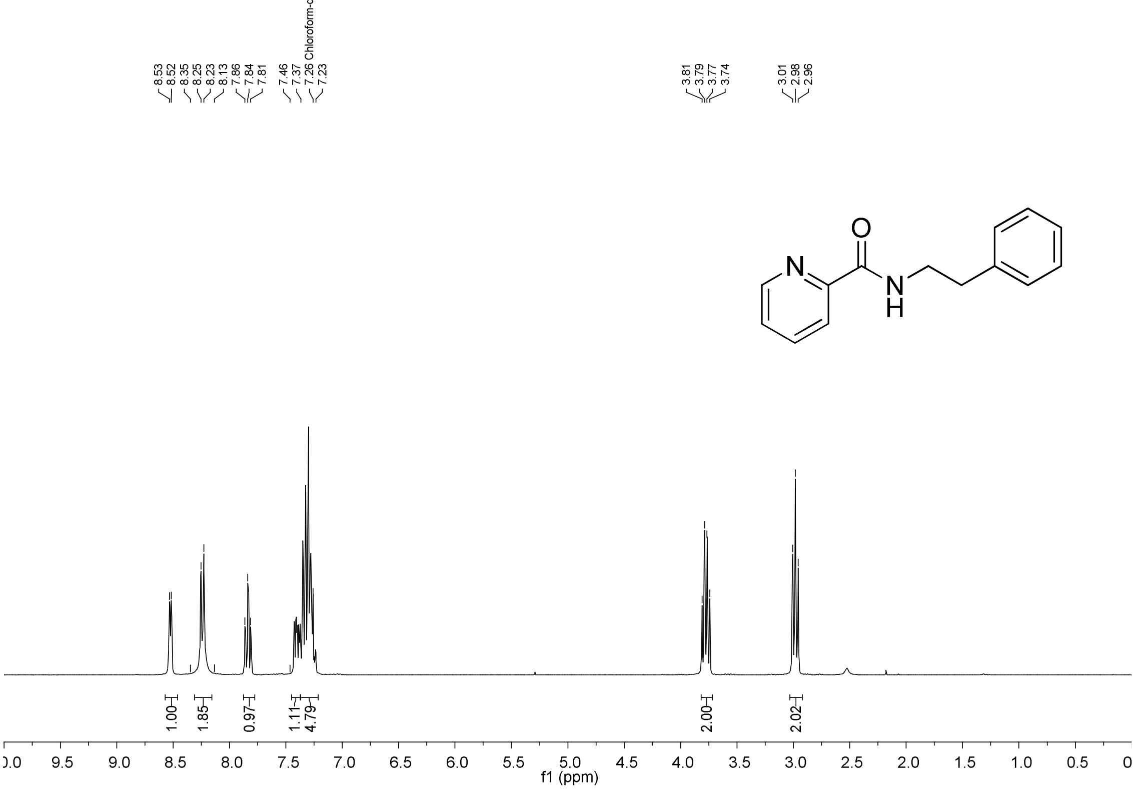

^{13}C NMR ($CDCl_3$, 75 MHz)

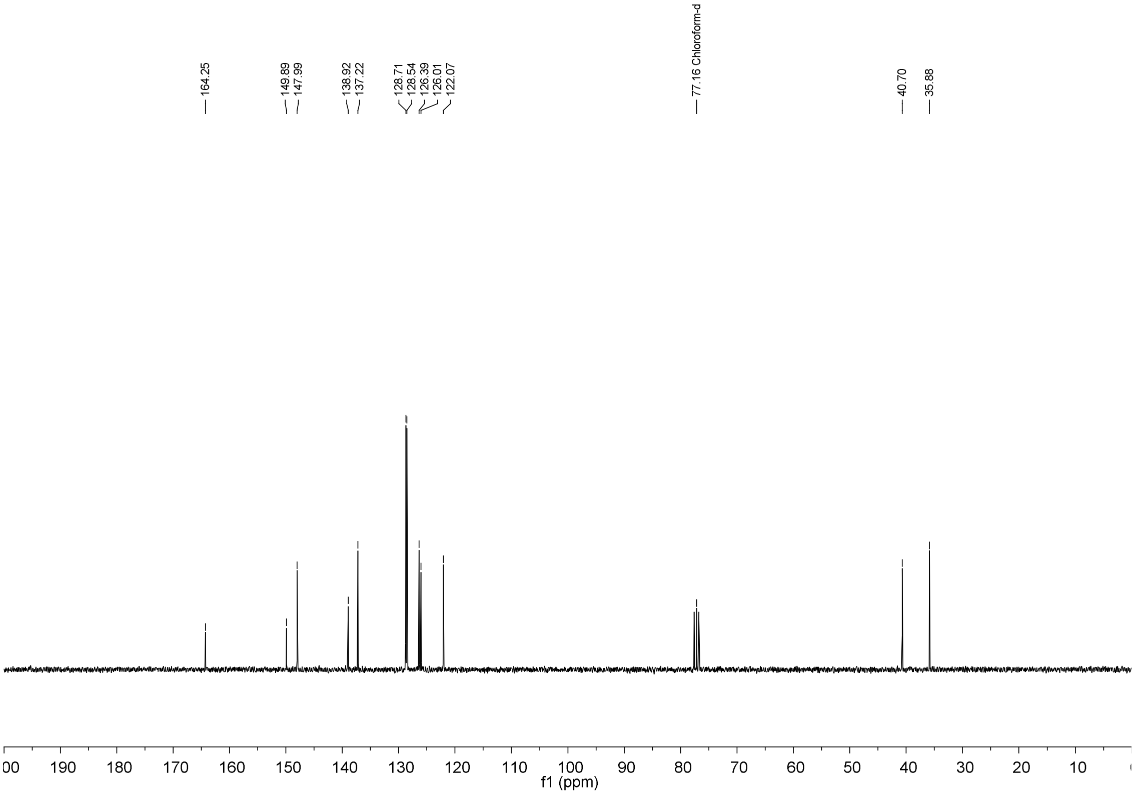

***N*-(4-Methoxyphenethyl)picolinamide (62)**

^{1}H NMR (acetone-d_6, 300 MHz)

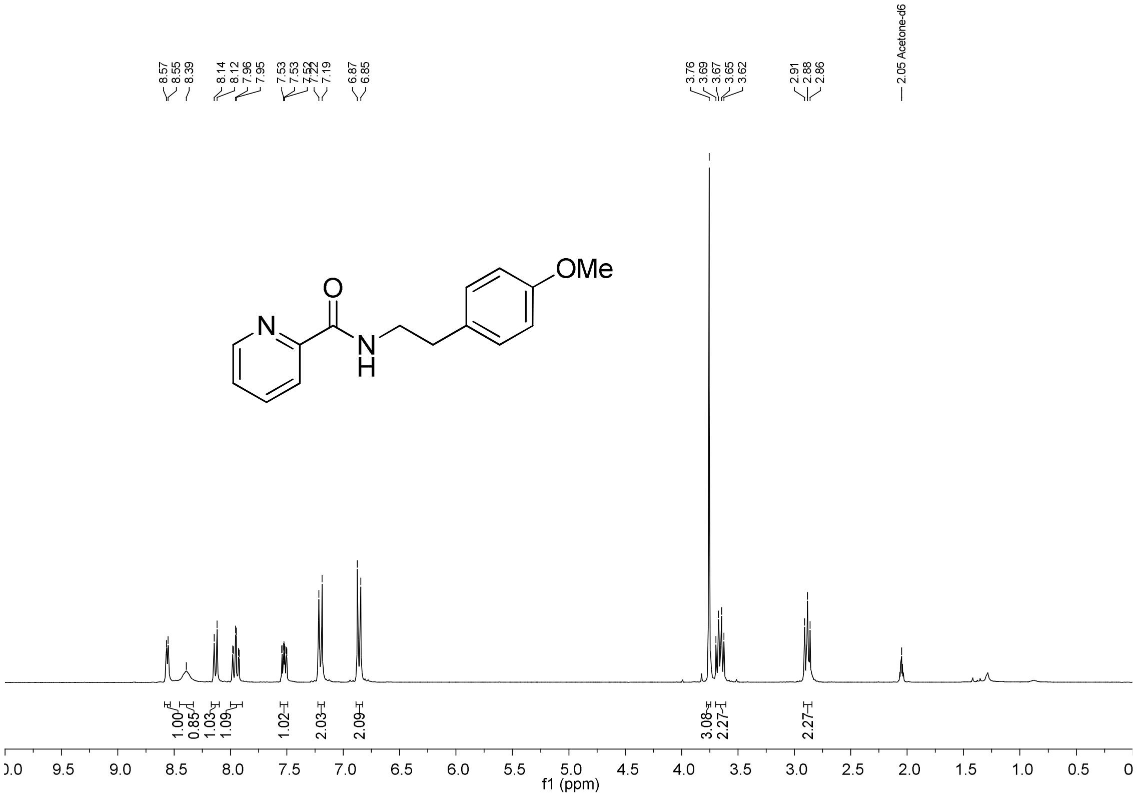

^{13}C NMR (acetone-d_6, 75 MHz)

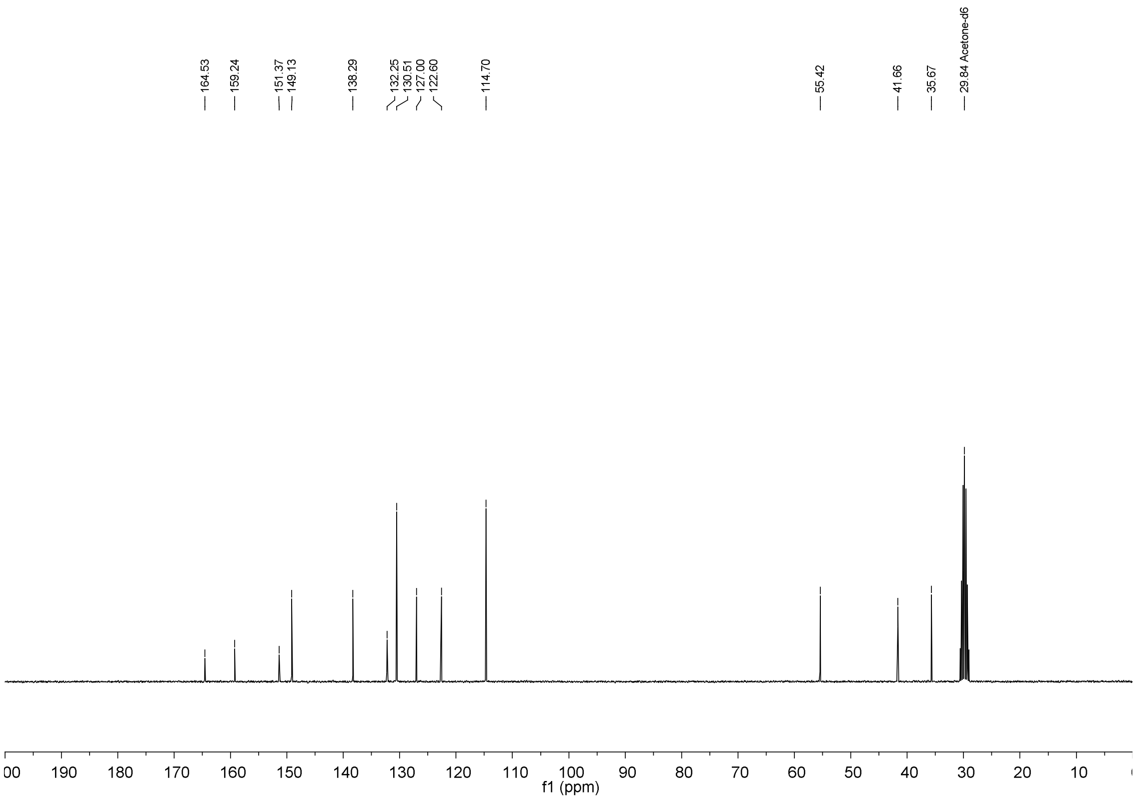

_N_-(4-Chlorophenethyl)picolinamide (63)

^{1}H NMR (acetone-d$_6$, 300 MHz)

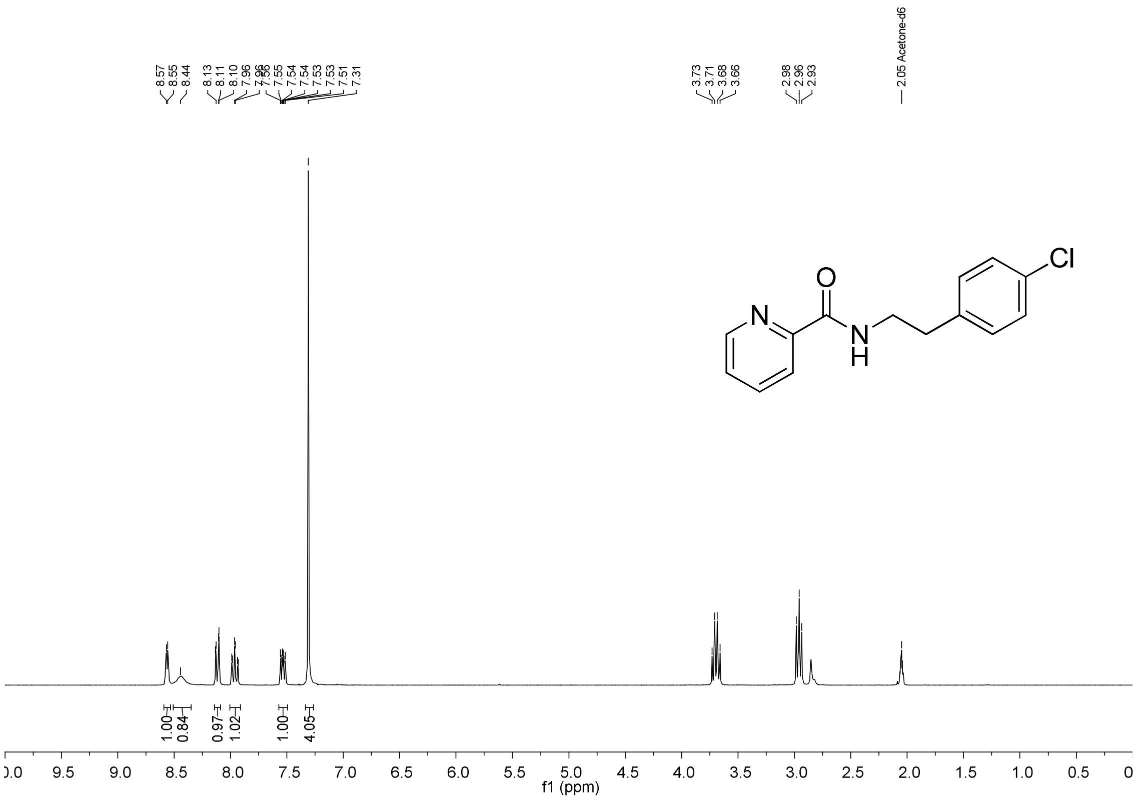

^{13}C NMR (acetone-d$_6$, 75 MHz)

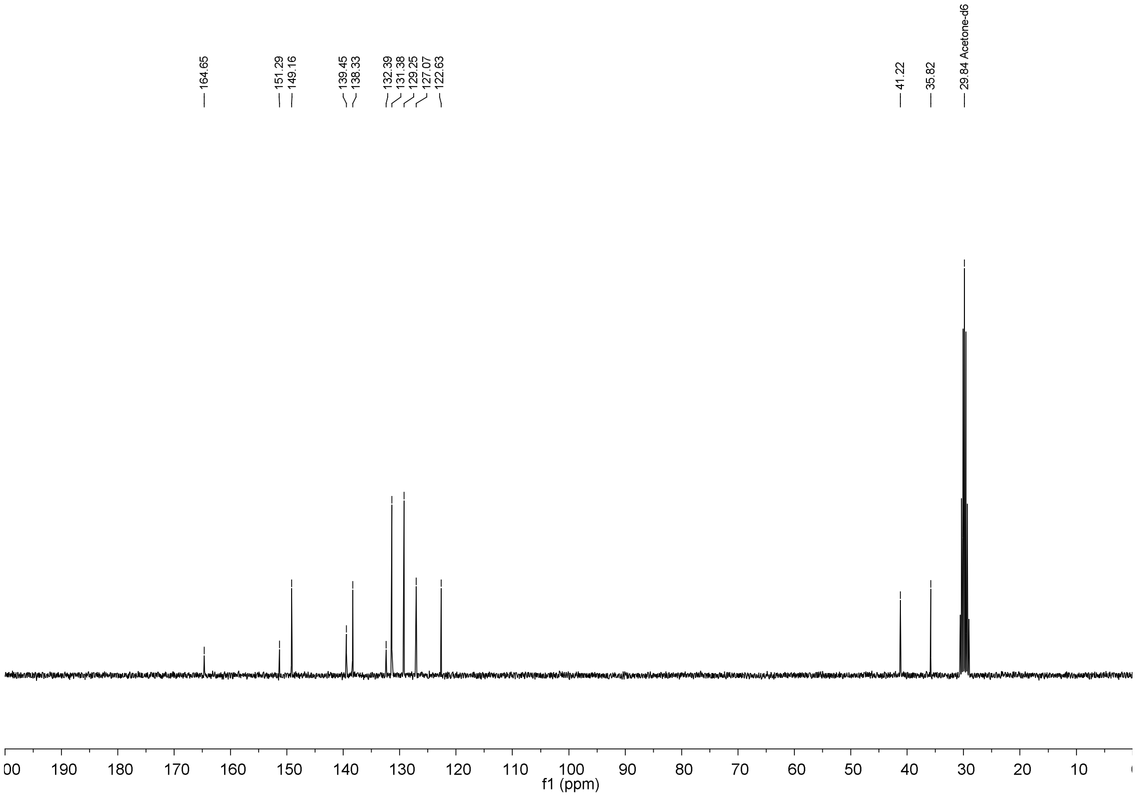

N-(4-Fluorophenethyl)picolinamide (64)

^{1}H NMR ($CDCl_3$, 300 MHz)

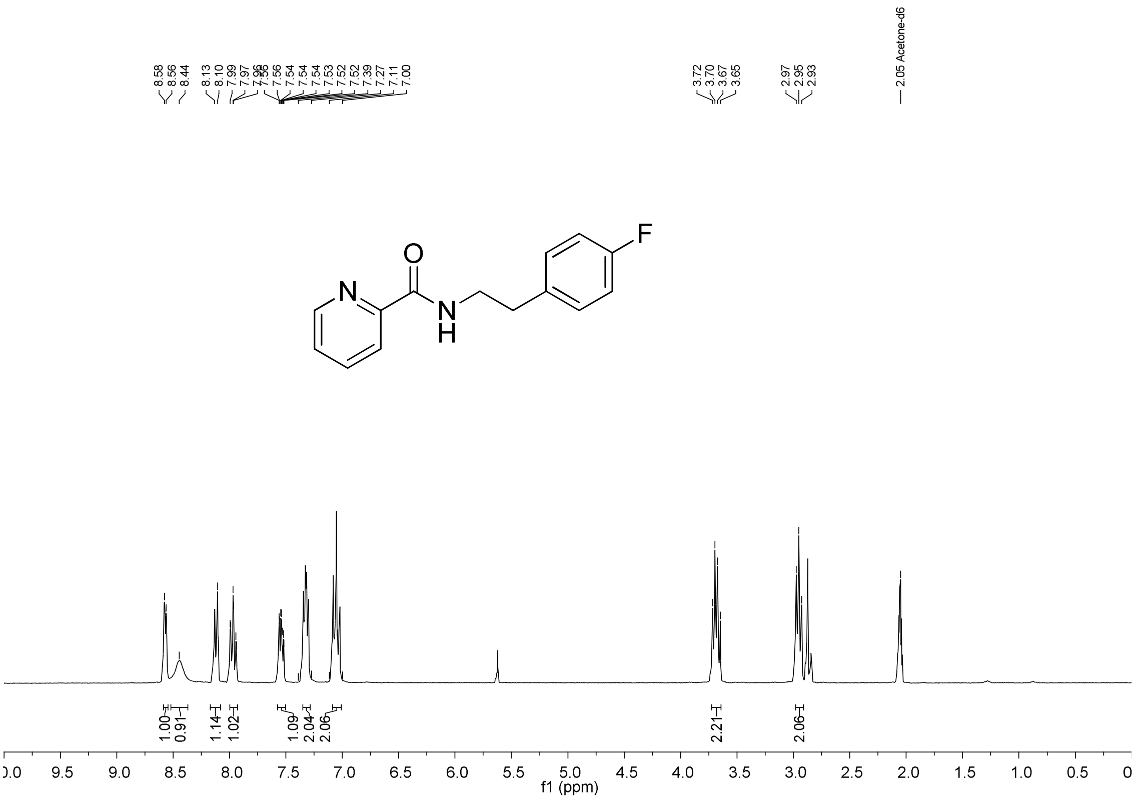

^{13}C NMR (acetone-d_6, 125 MHz)

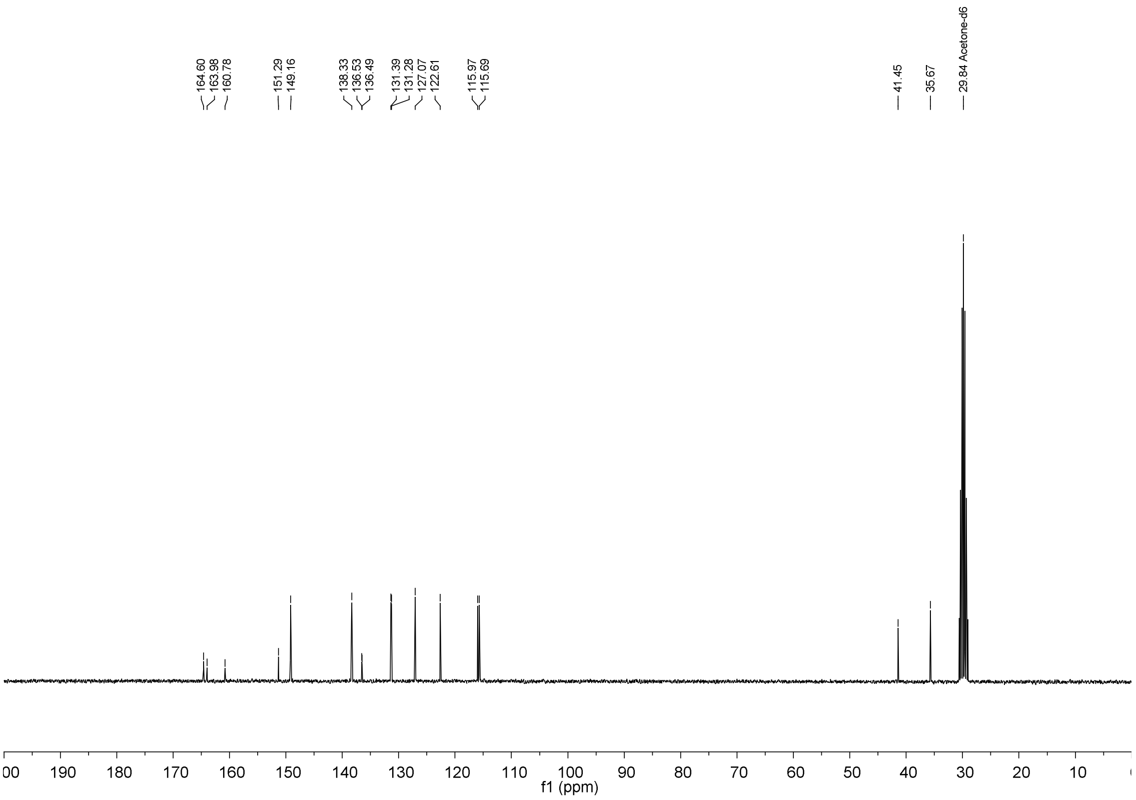

N-(3-Methoxyphenethyl)picolinamide (65)

^{1}H NMR (acetone-d$_6$, 300 MHz)

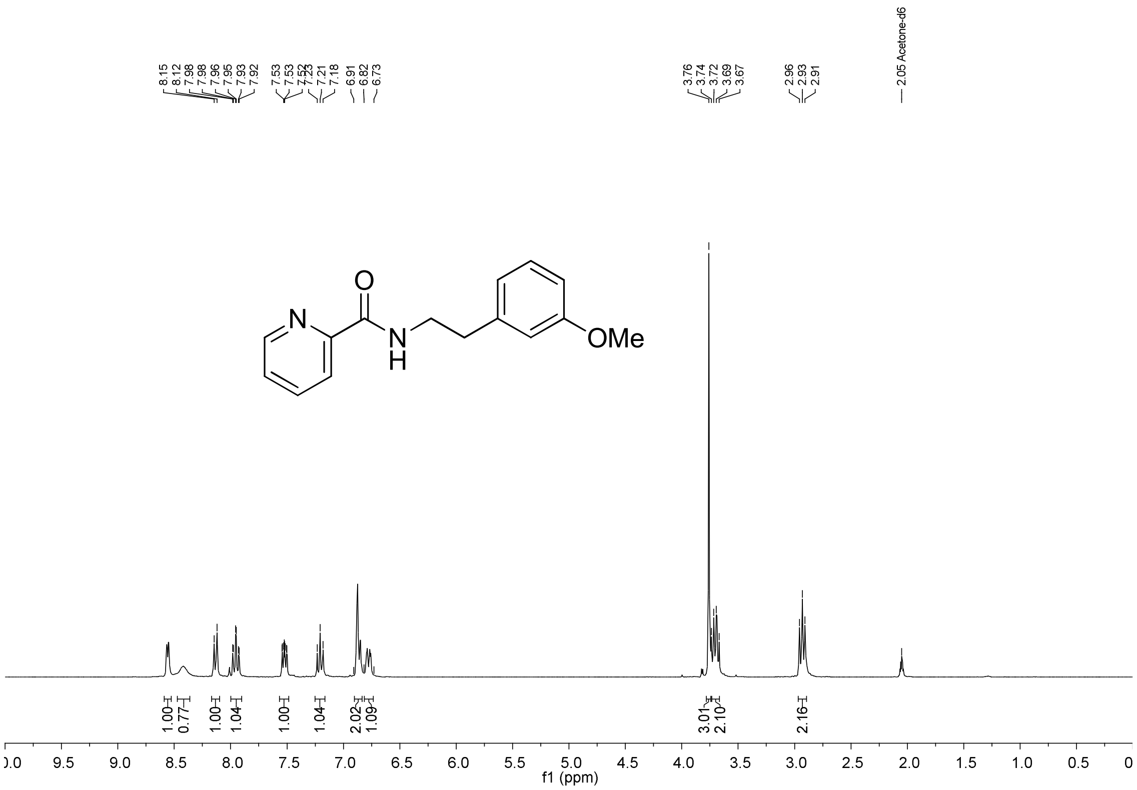

^{13}C NMR (acetone-d$_6$, 75 MHz)

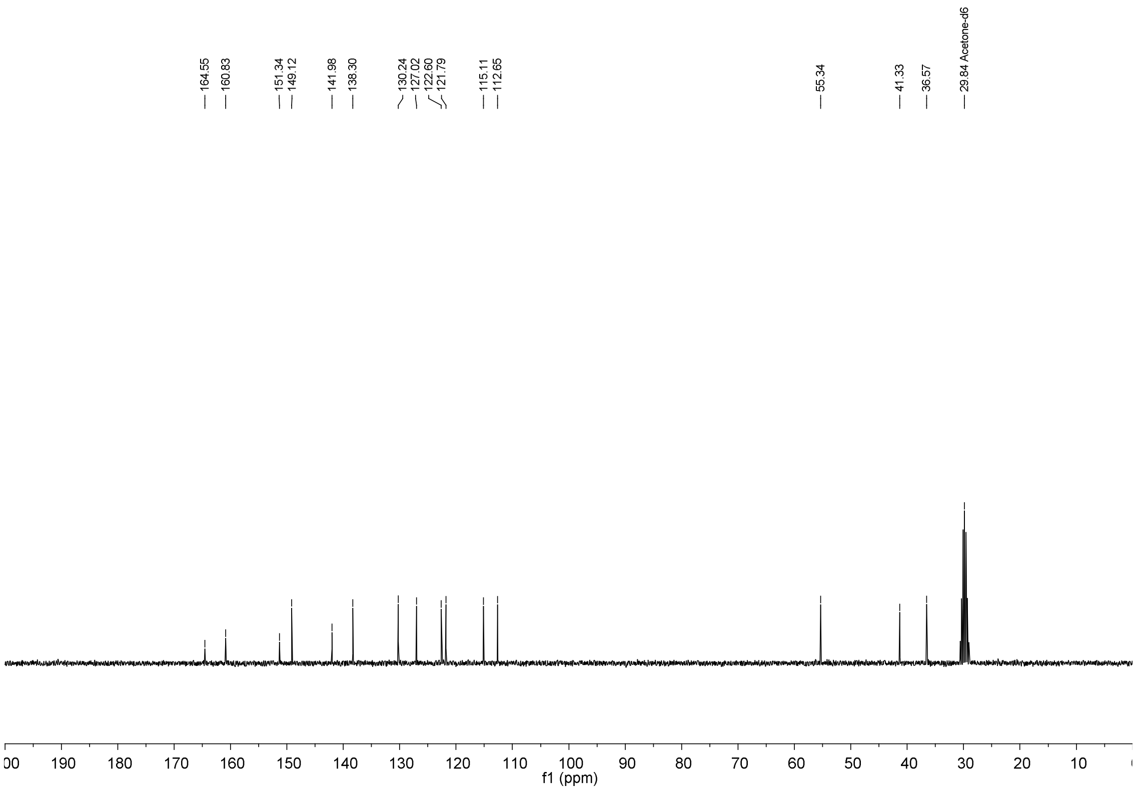

N-(3-Methylphenethyl)picolinamide (66)

^{1}H NMR (acetone-d$_6$, 300 MHz)

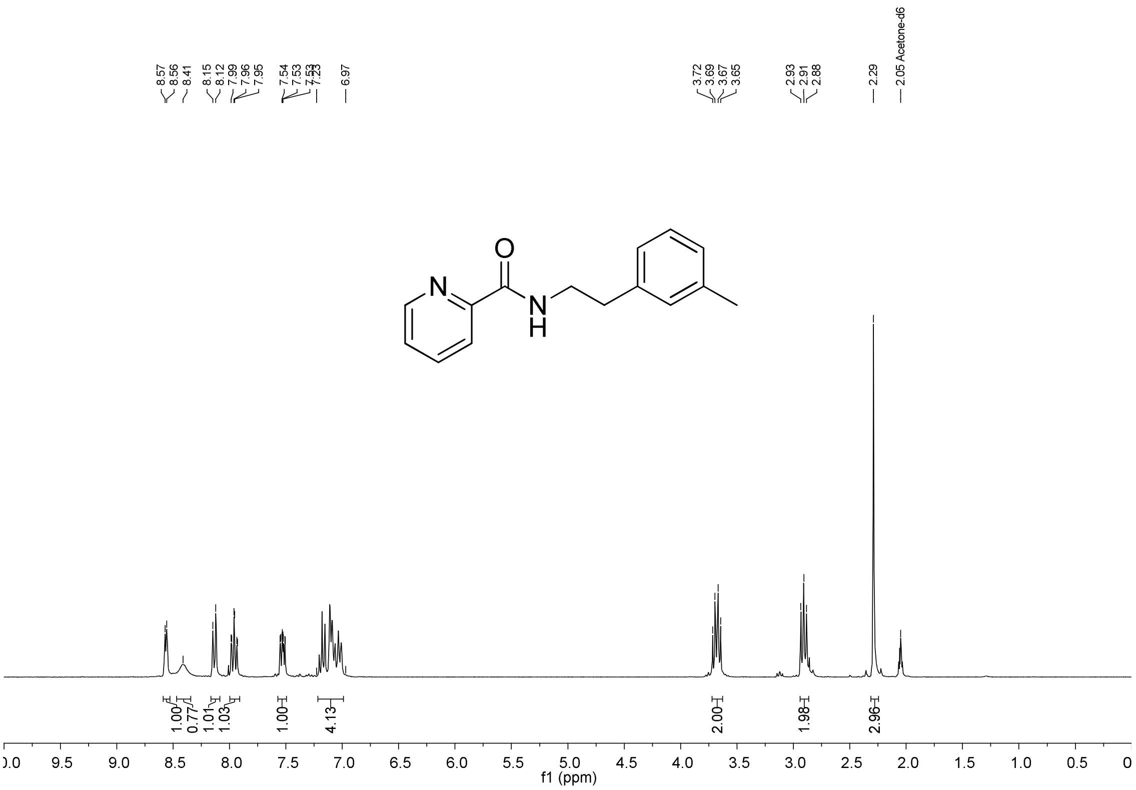

^{13}C NMR (acetone-d$_6$, 75 MHz)

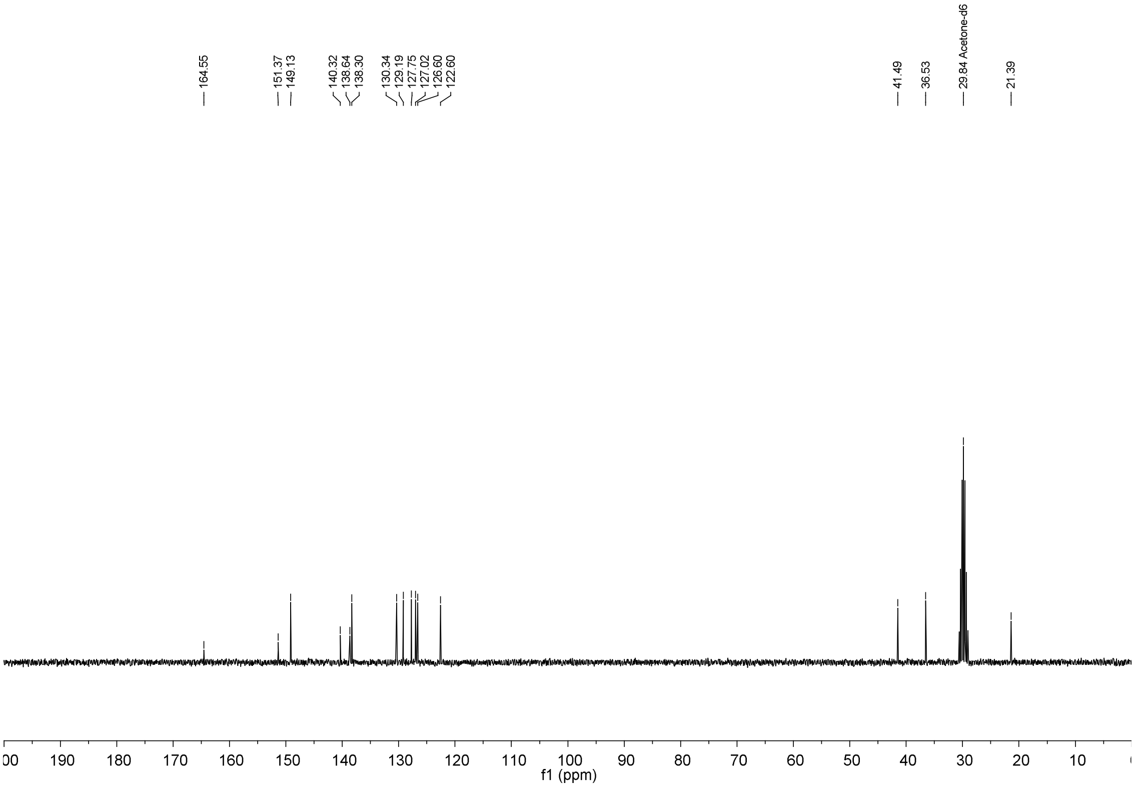

_N_-(2-Methoxyphenethyl)picolinamide (67)

^{1}H NMR (acetone-d$_6$, 300 MHz)

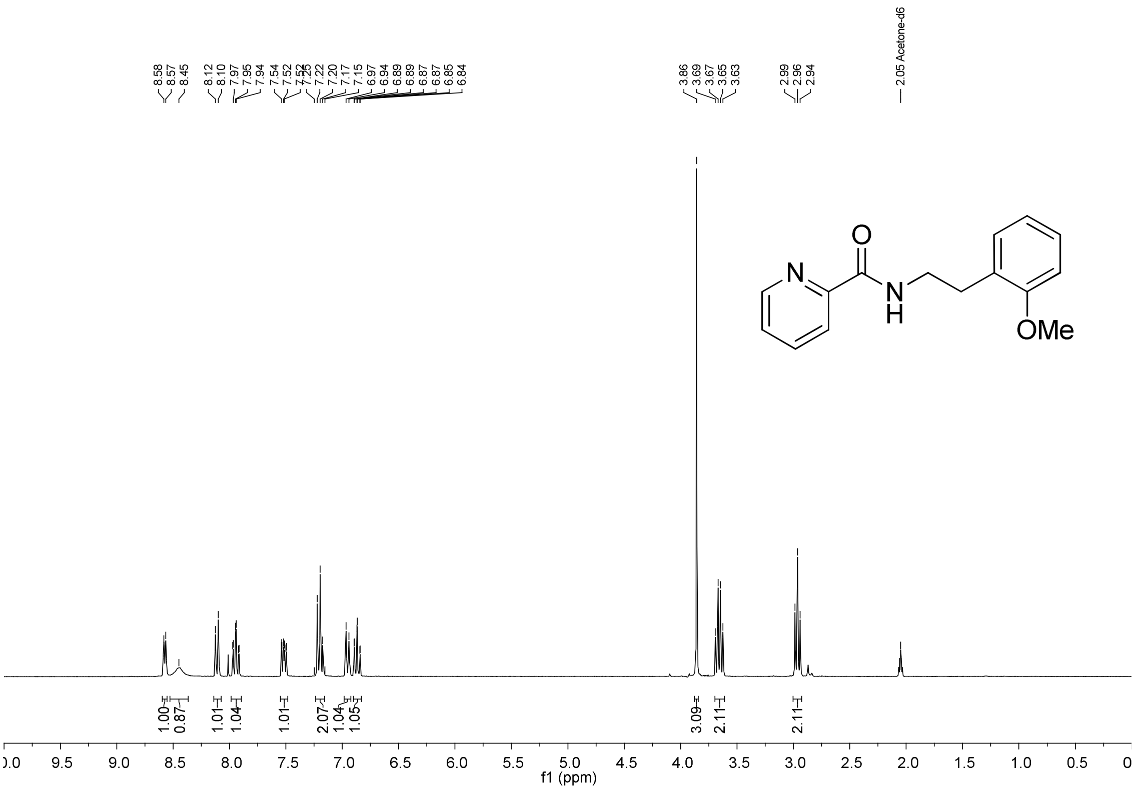

^{13}C NMR (acetone-d$_6$, 75 MHz)

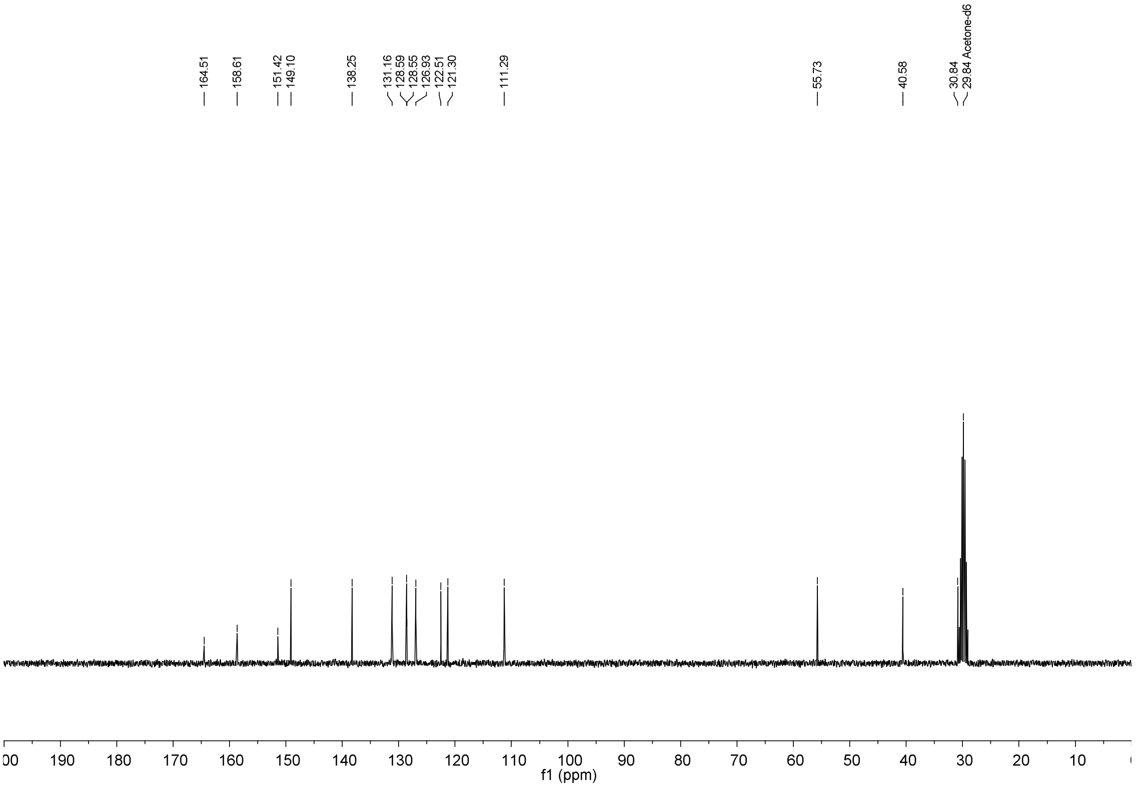

N-(2-Methylphenethyl)picolinamide (68)

^{1}H NMR (acetone-d$_6$, 300 MHz)

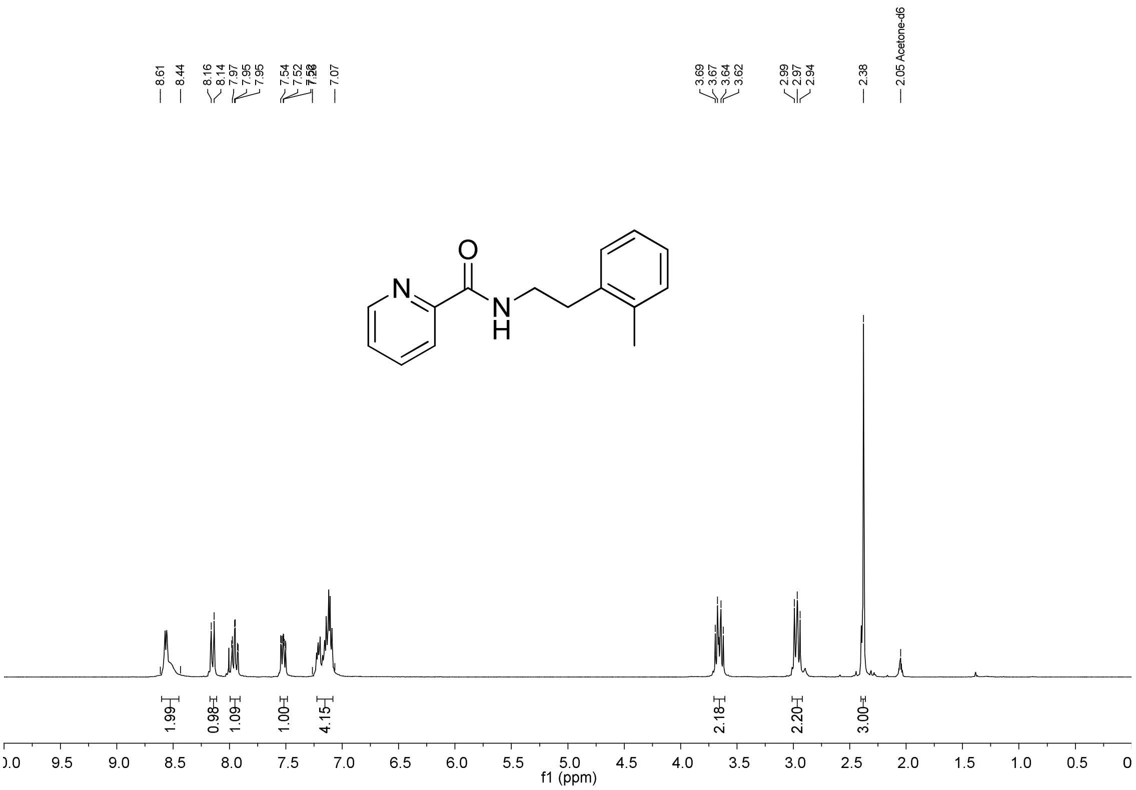

^{13}C NMR (acetone-d$_6$, 75 MHz)

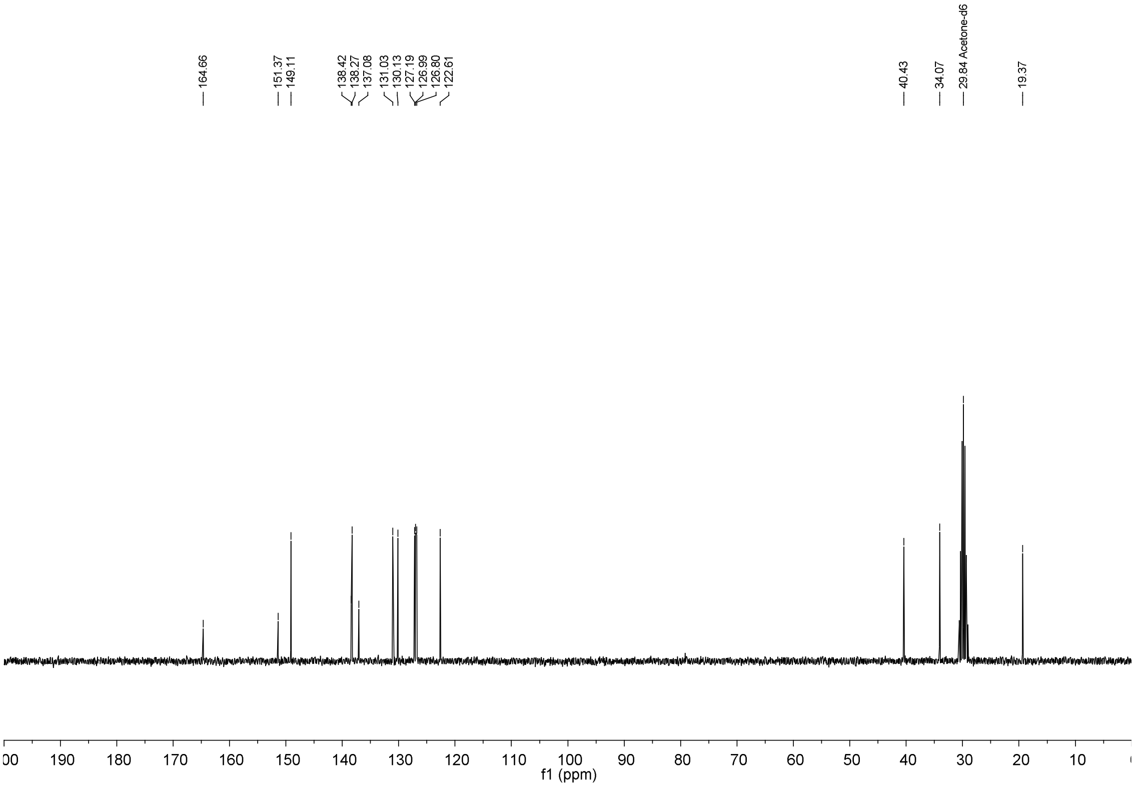

***N*-(2-Bromophenethyl)picolinamide (69)**

^{1}H NMR (acetone-d$_6$, 300 MHz)

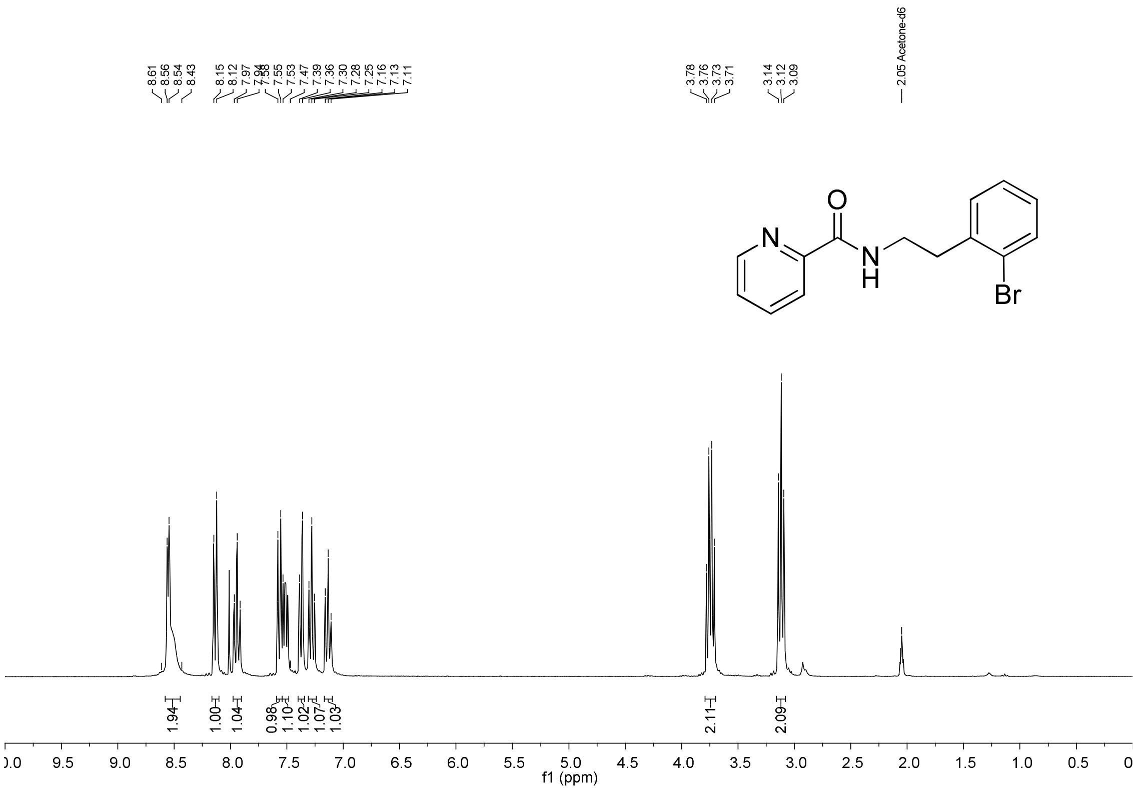

^{13}C NMR (acetone-d$_6$, 75 MHz)

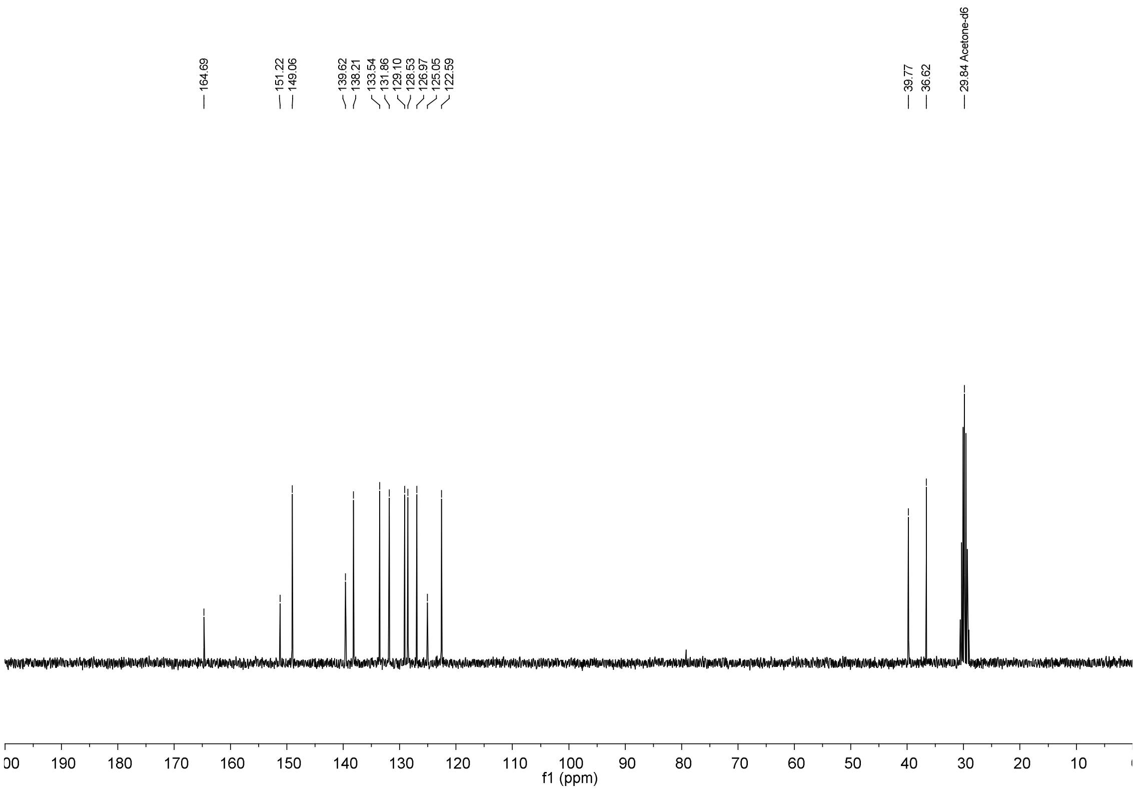

***N*-(2-Chlorophenethyl)picolinamide (70)**

^{1}H NMR (acetone-d$_6$, 300 MHz)

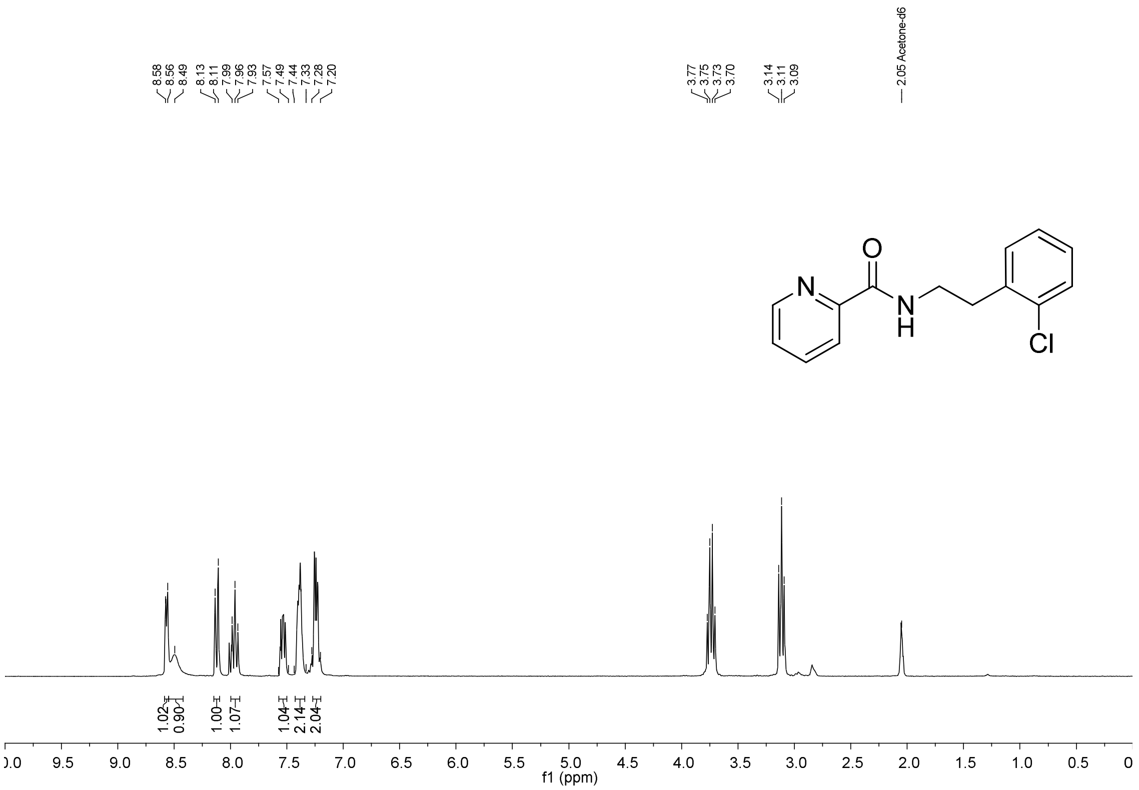

^{13}C NMR (acetone-d$_6$, 75 MHz)

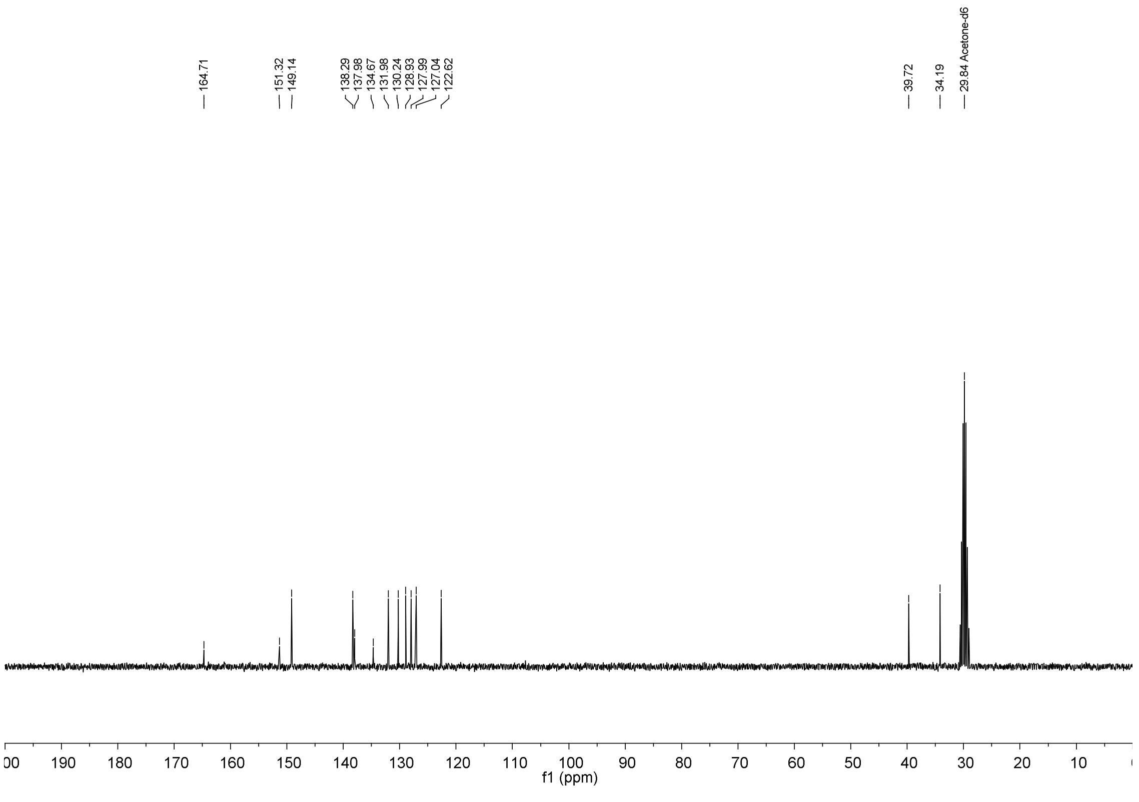

N-(2-(Naphthalen-2-yl)ethyl)picolinamide (71)

^{1}H NMR (CDCl$_3$, 300 MHz)

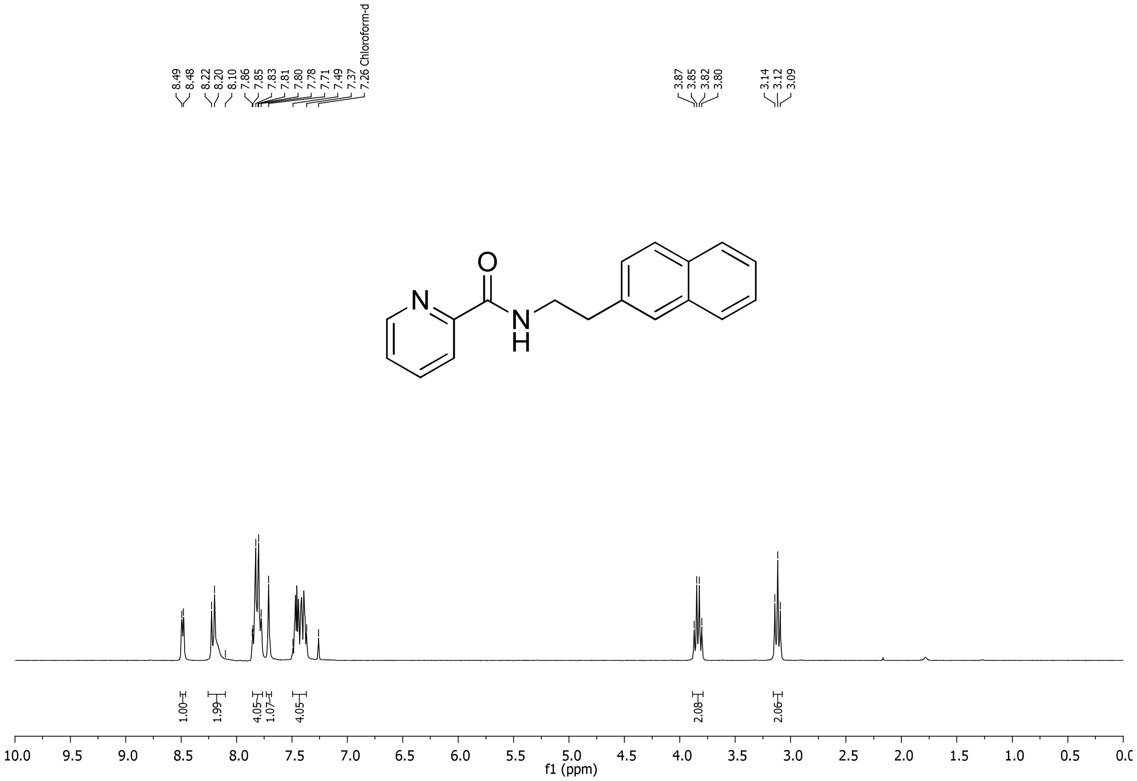

^{13}C NMR (CDCl$_3$, 75 MHz)

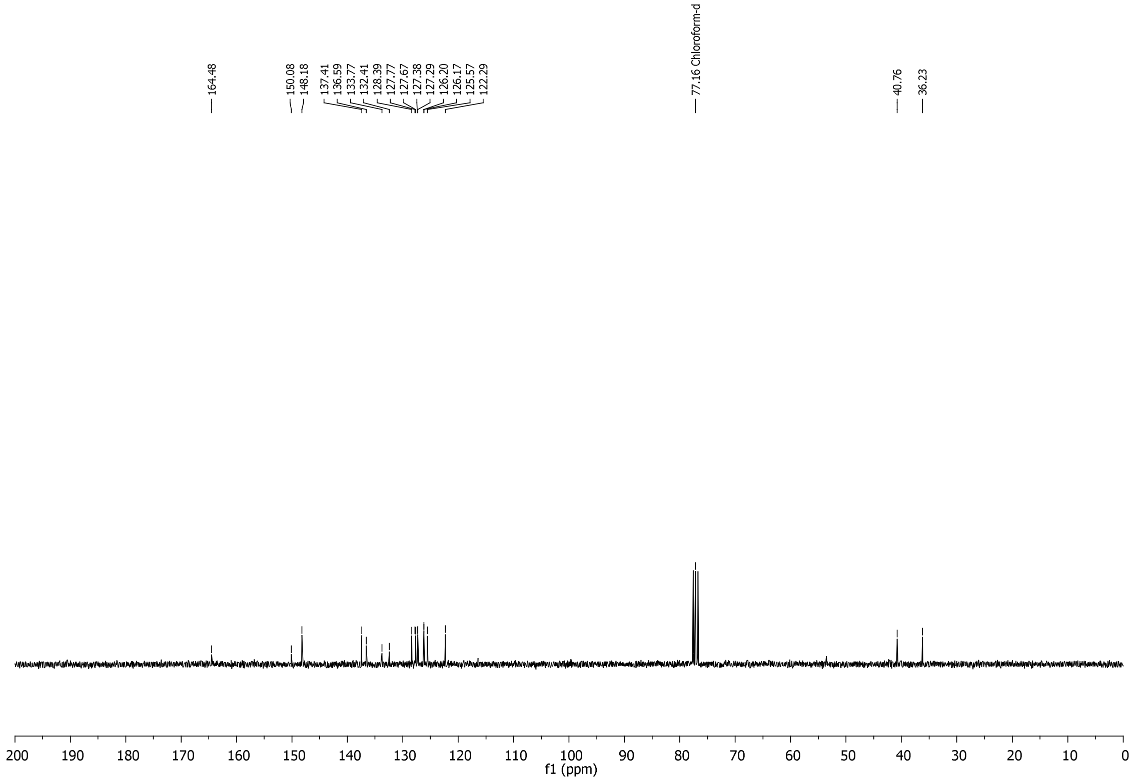

_N_-(2-(Thiophen-2-yl)ethyl)picolinamide (72)

^{1}H NMR ($CDCl_3$, 300 MHz)

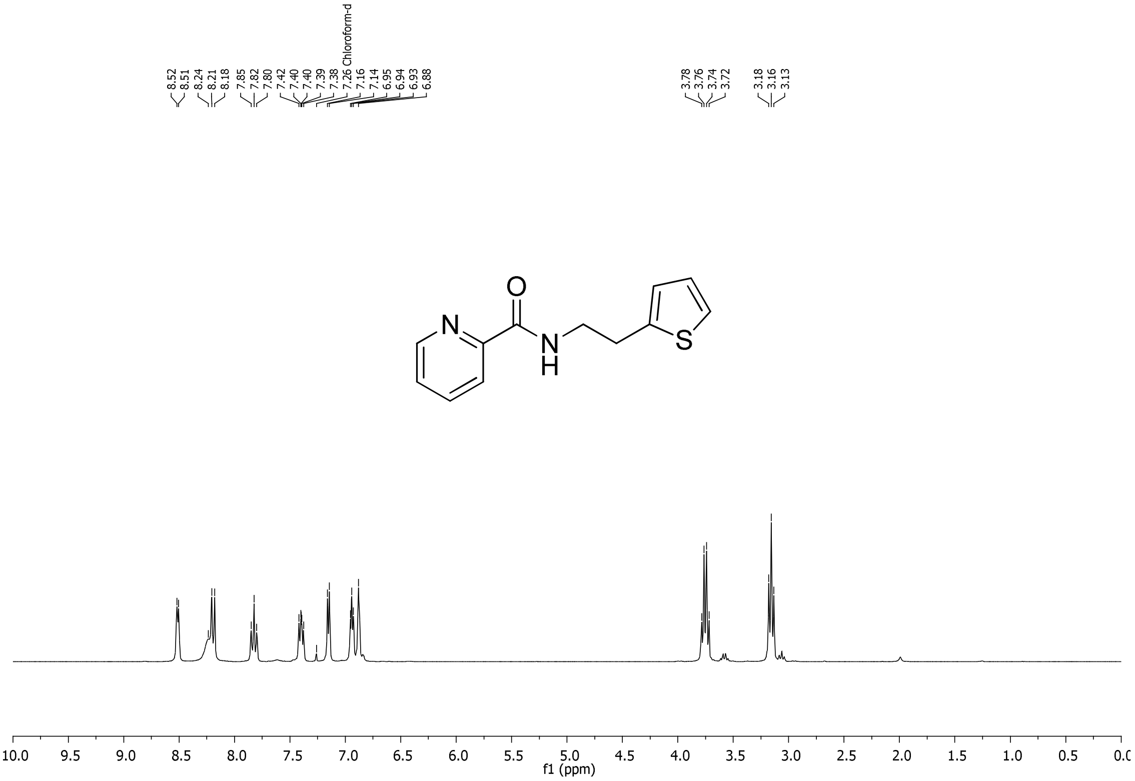

^{13}C NMR ($CDCl_3$, 75 MHz)

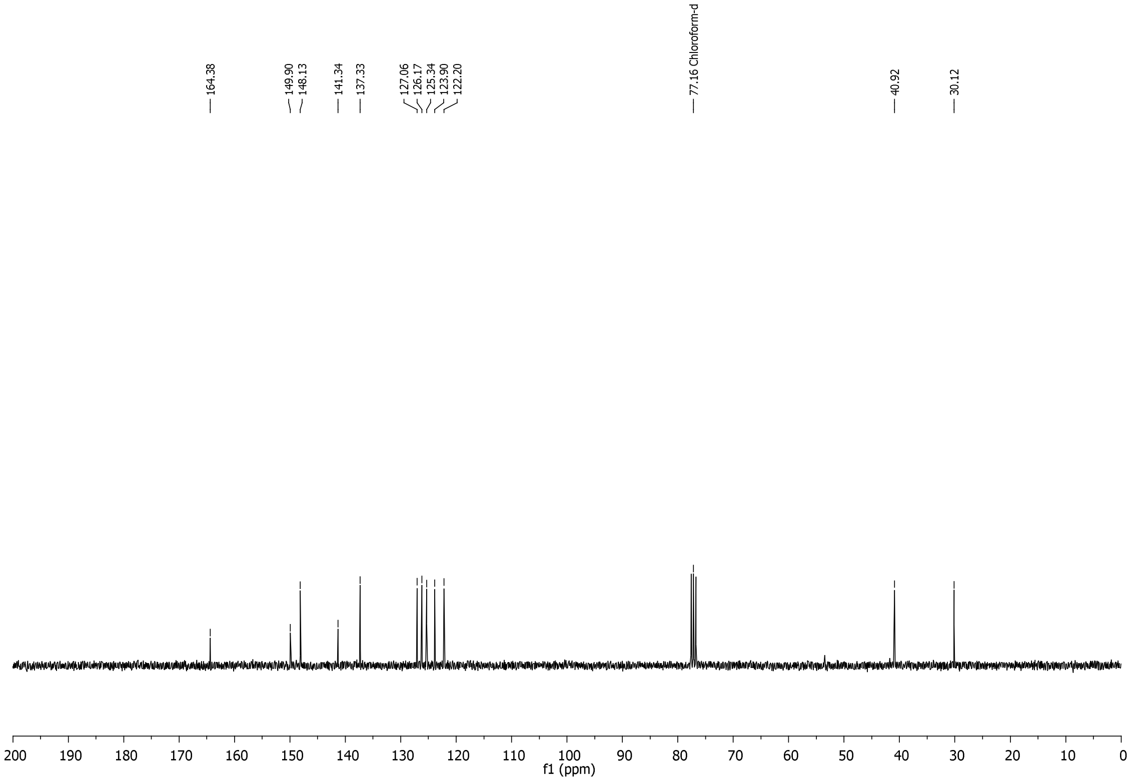

***N*-(3-Phenylpropyl)picolinamide**

^{1}H NMR (acetone-d$_6$, 300 MHz)

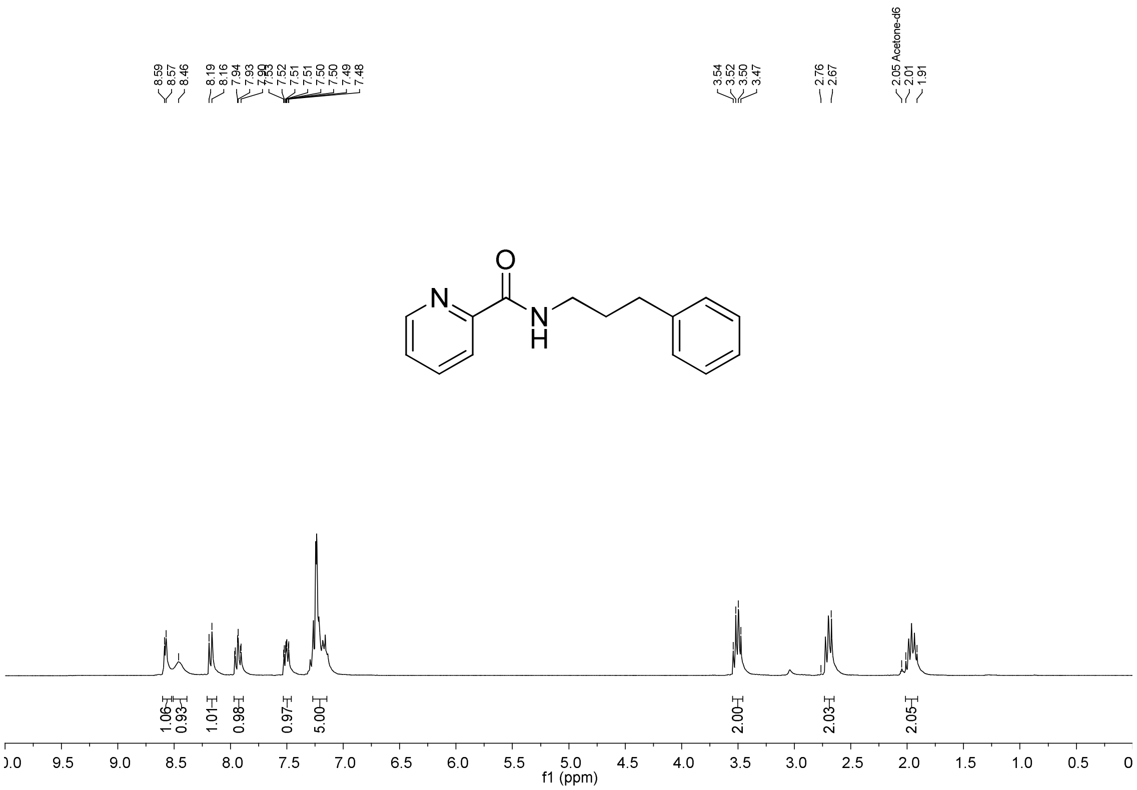

^{13}C NMR (acetone-d$_6$, 75 MHz)

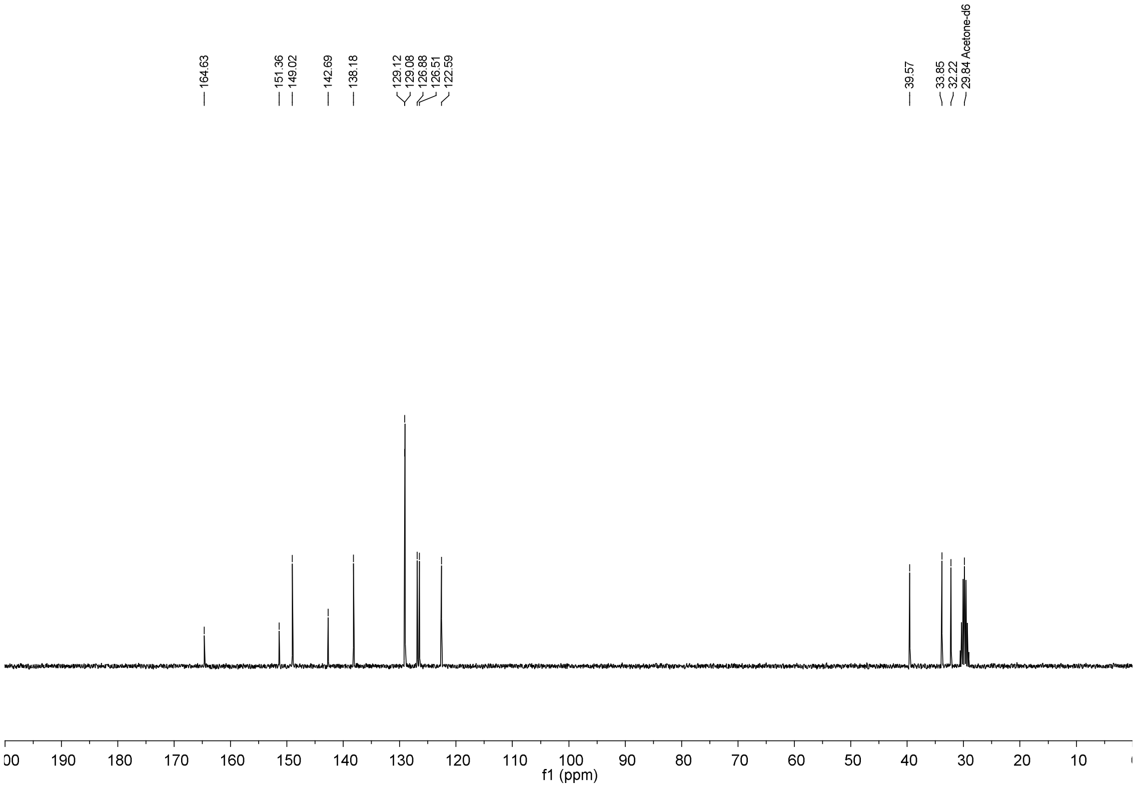

***N*-Ethylpicolinamide (4)**

^{1}H NMR ($CDCl_3$, 300 MHz)

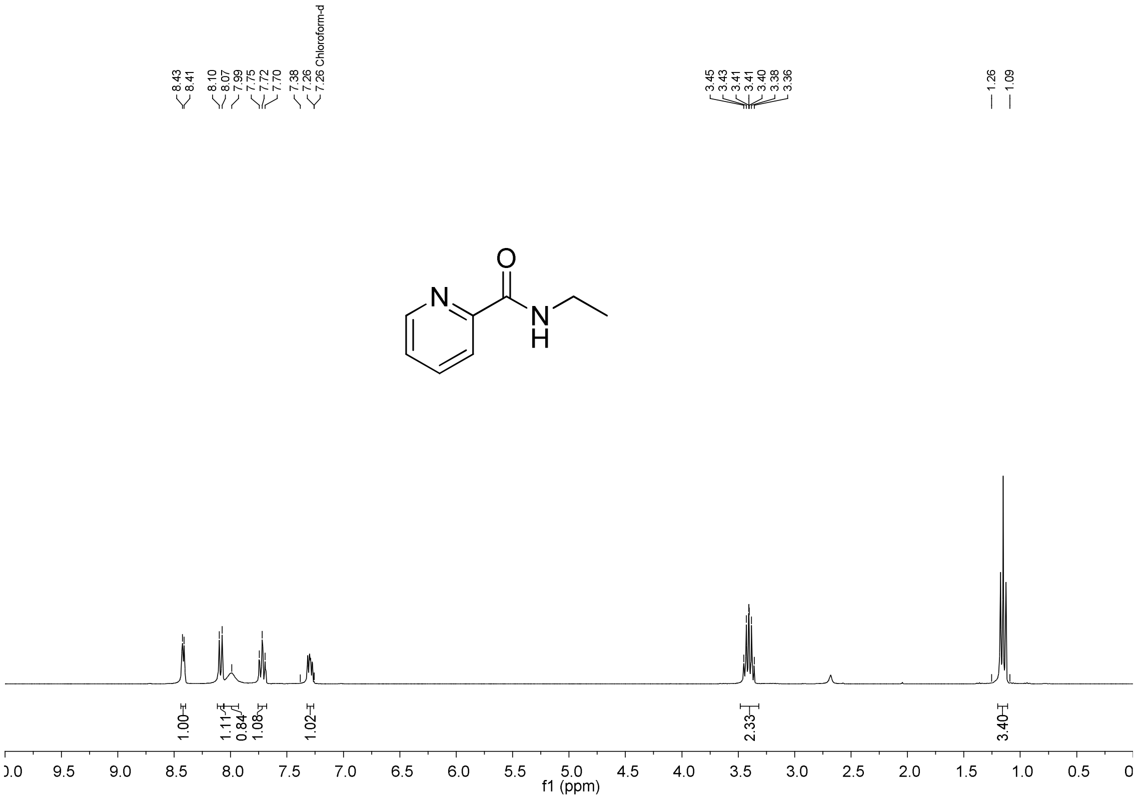

^{13}C NMR ($CDCl_3$, 75 MHz)

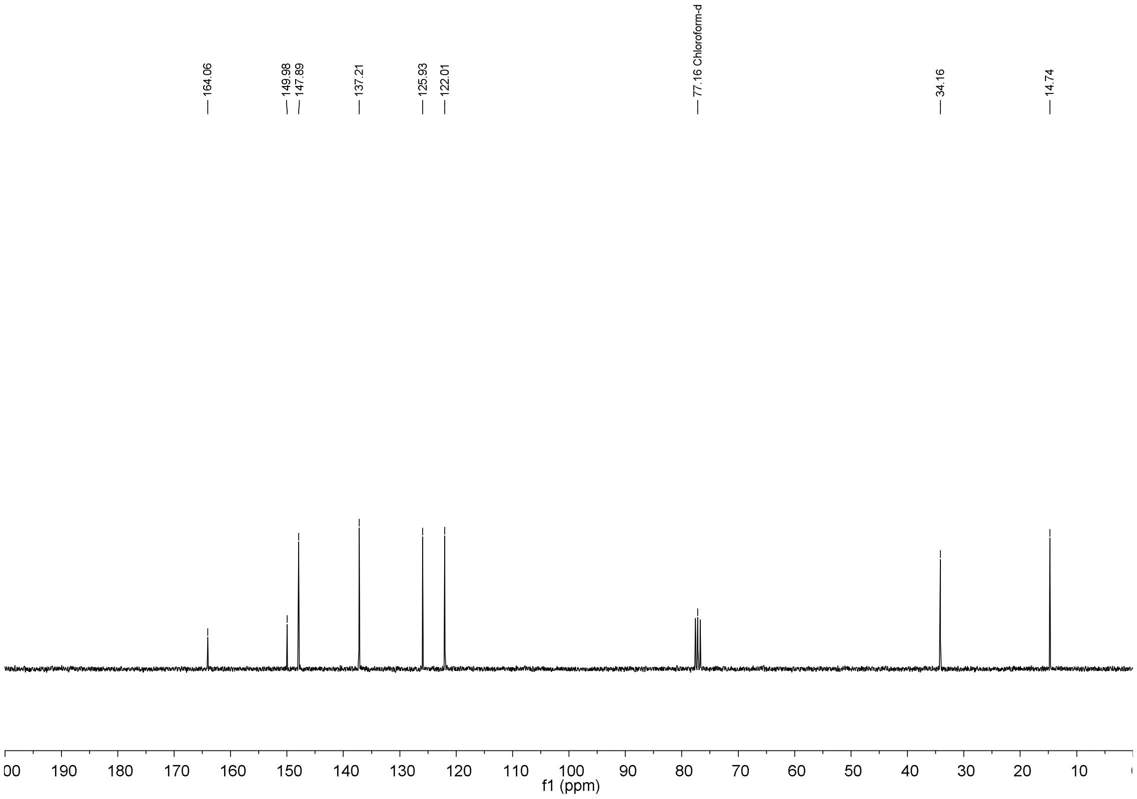

1,2-Bis(4-methoxyphenyl)ethyne (I)

^{1}H NMR (acetone-d$_6$, 300 MHz)

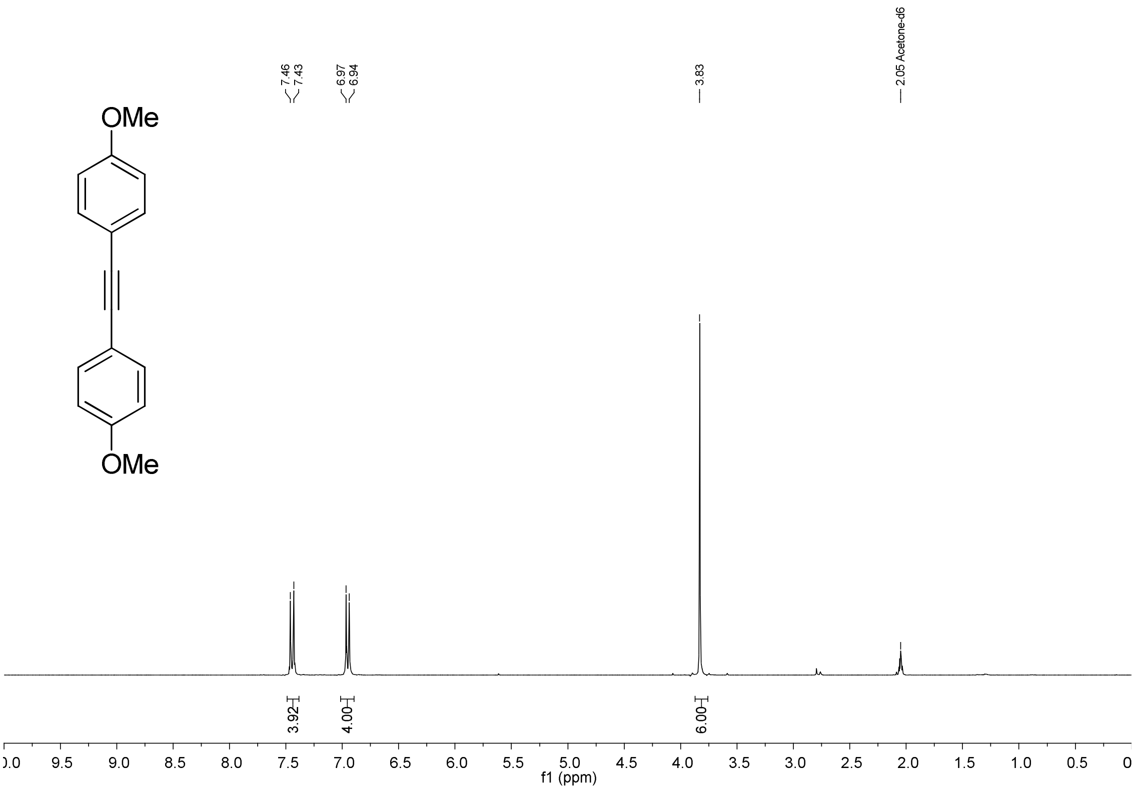

^{13}C NMR (acetone-d$_6$, 75 MHz)

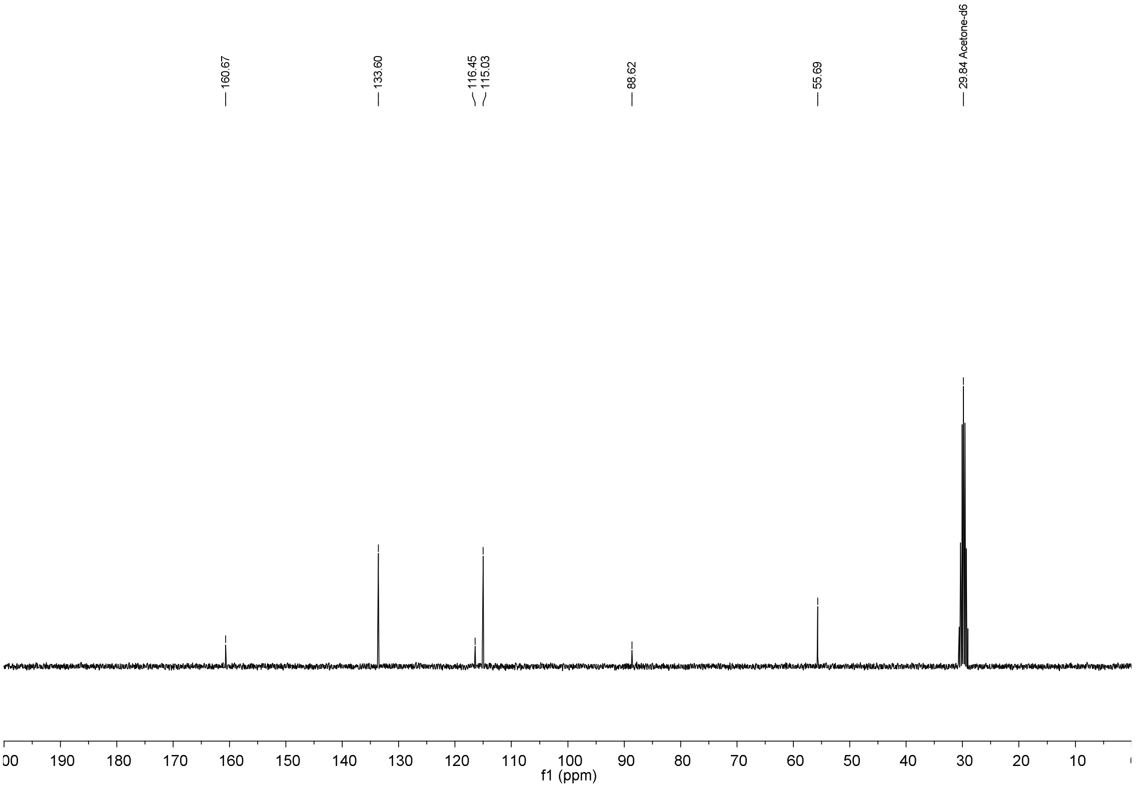

1,2-Bis(4-(*tert*-butyl)phenyl)ethyne (II)

^{1}H NMR (acetone-d$_6$, 300 MHz)

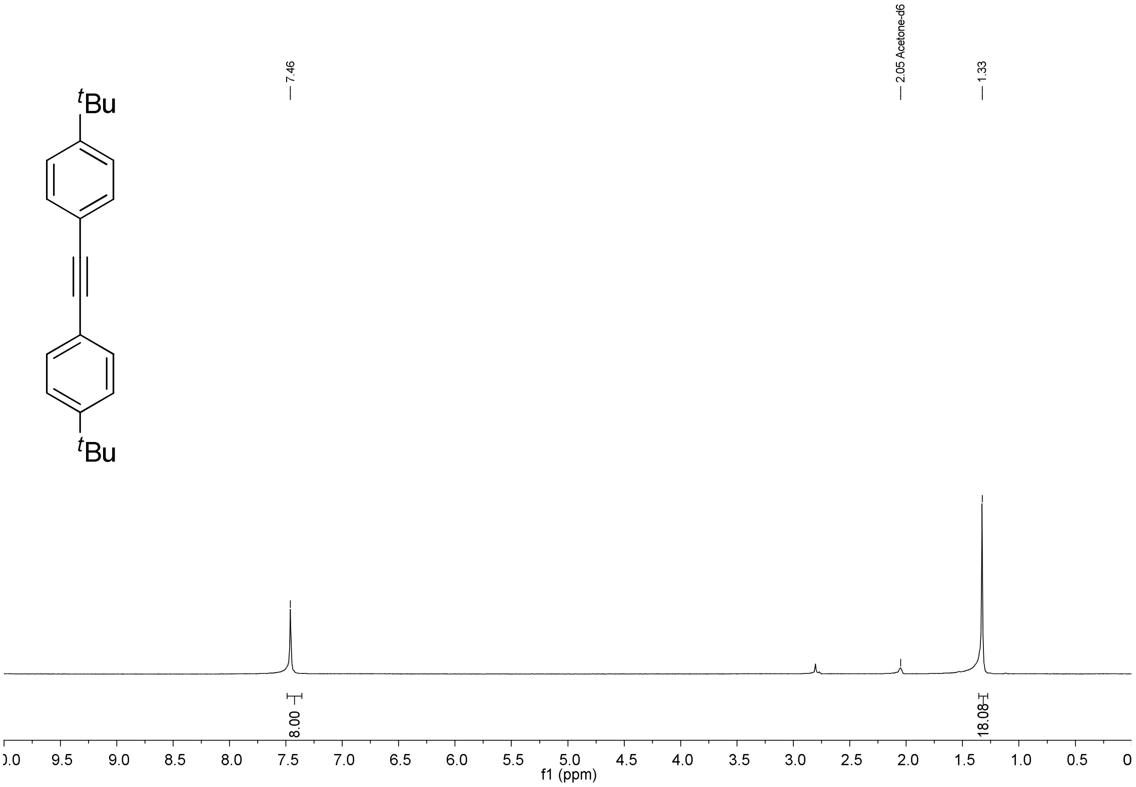

^{13}C NMR (acetone-d$_6$, 75 MHz)

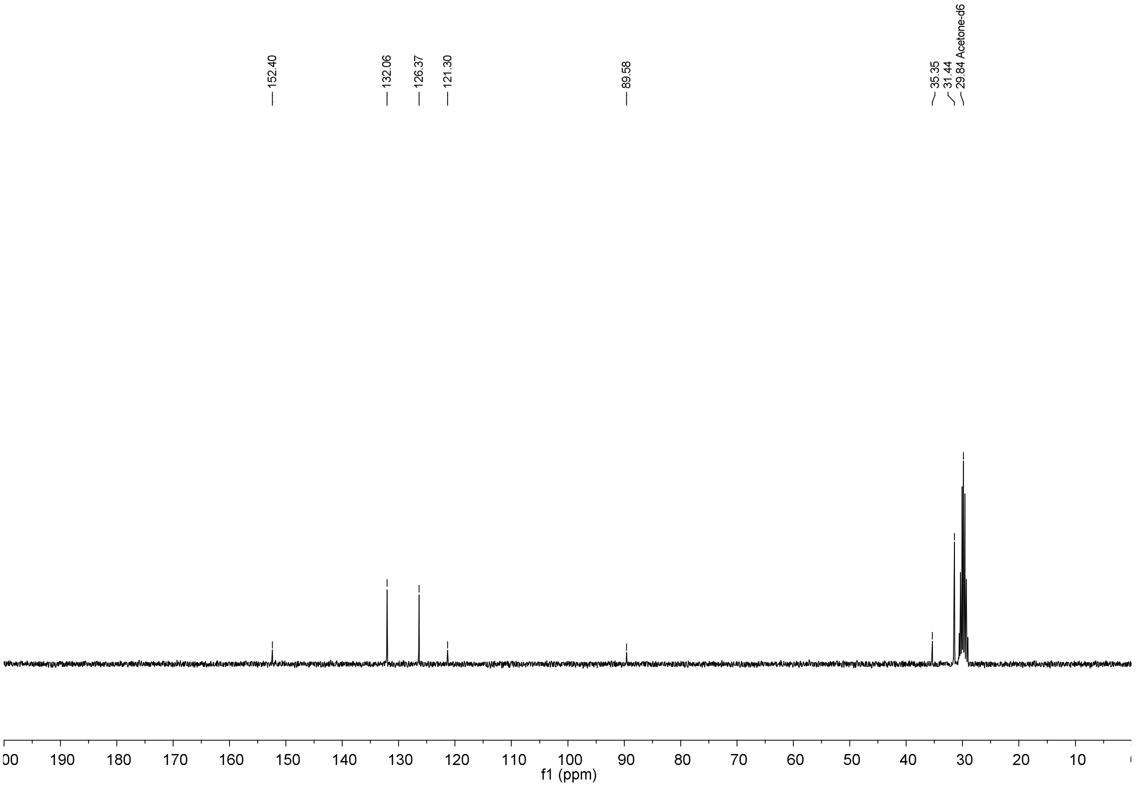

1,2-Di-*p*-tolylethyne (III)

^{1}H NMR (acetone-d_6, 300 MHz)

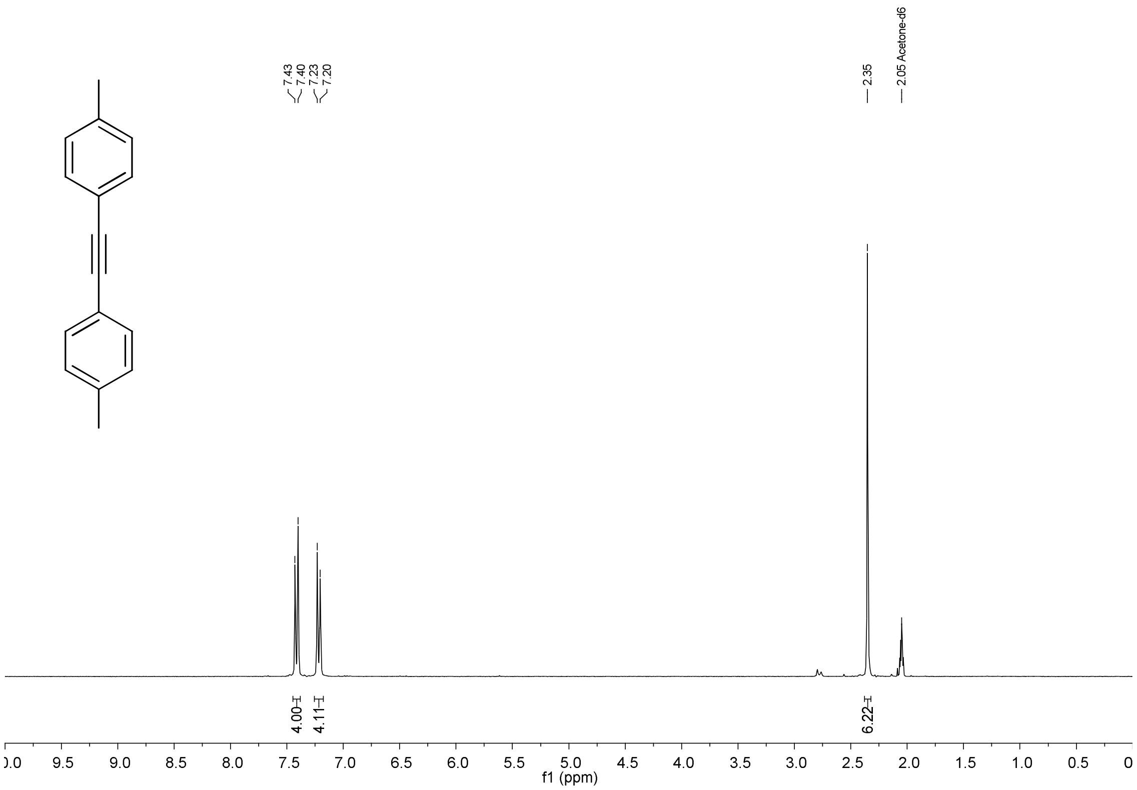

^{13}C NMR (acetone-d_6, 75 MHz)

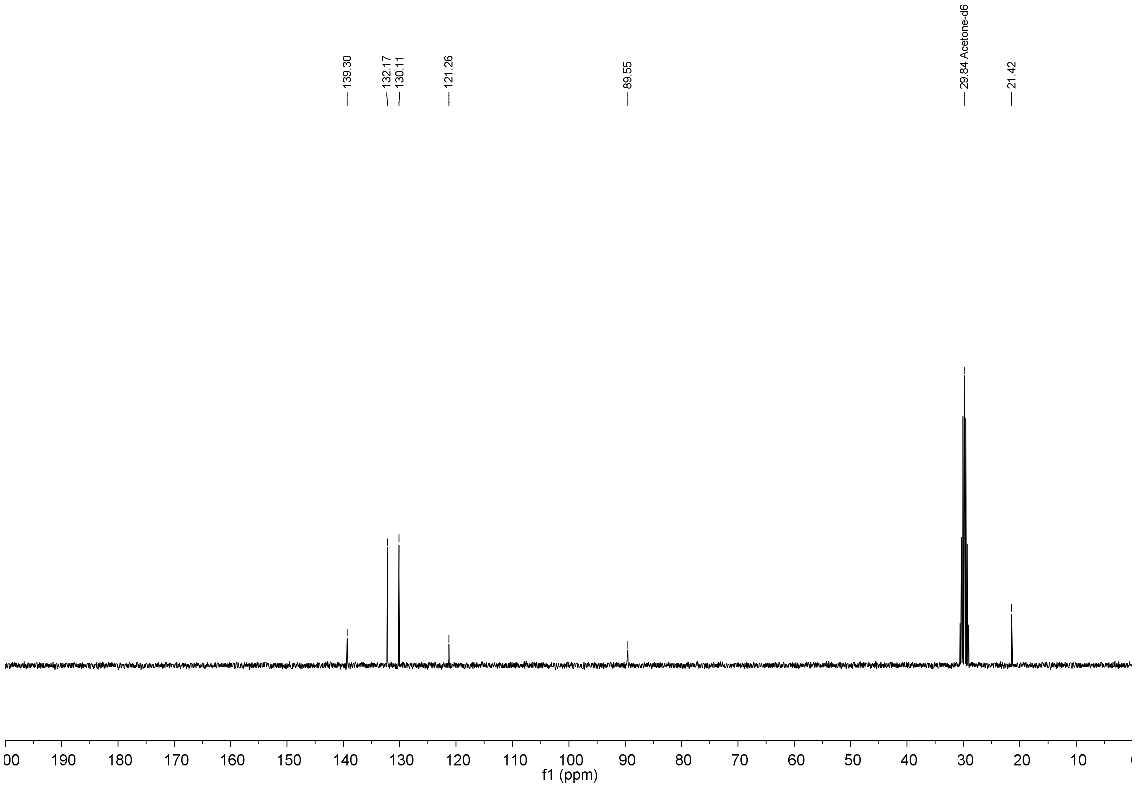

1,2-Bis(4-(trifluoromethyl)phenyl)ethyne (IV)

^{1}H NMR ($CDCl_3$, 300 MHz)

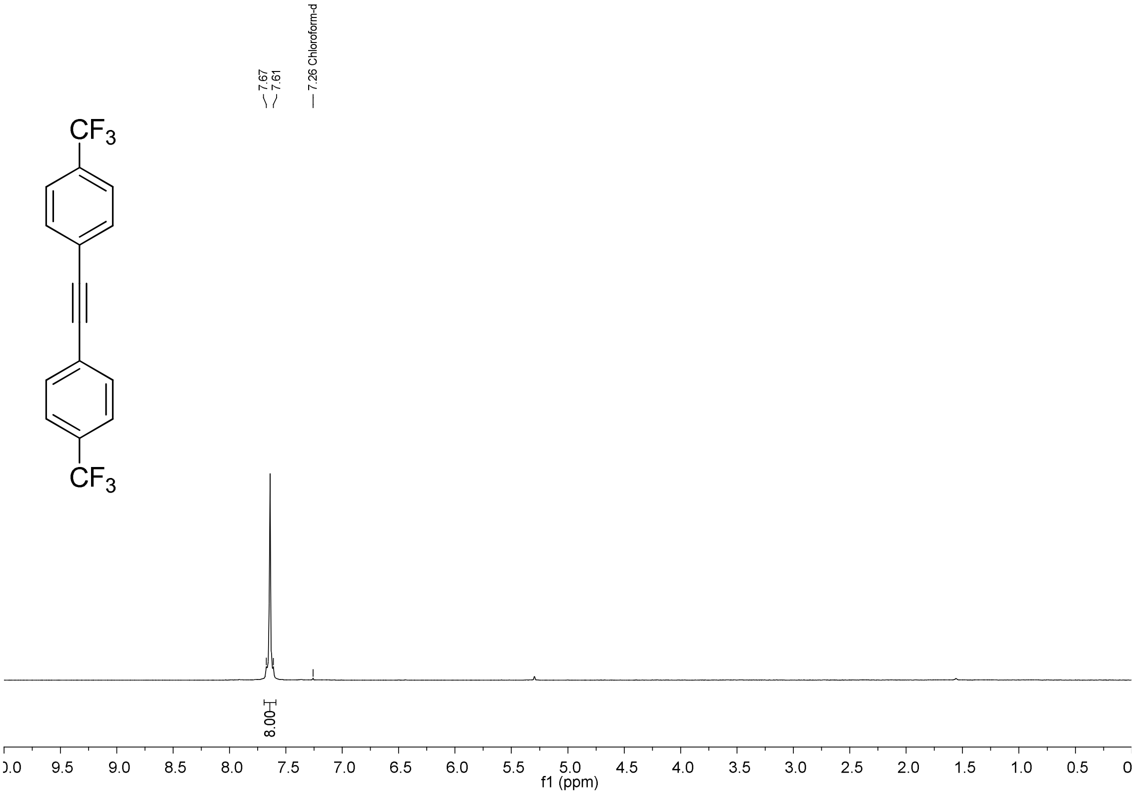

^{13}C NMR ($CDCl_3$, 125 MHz)

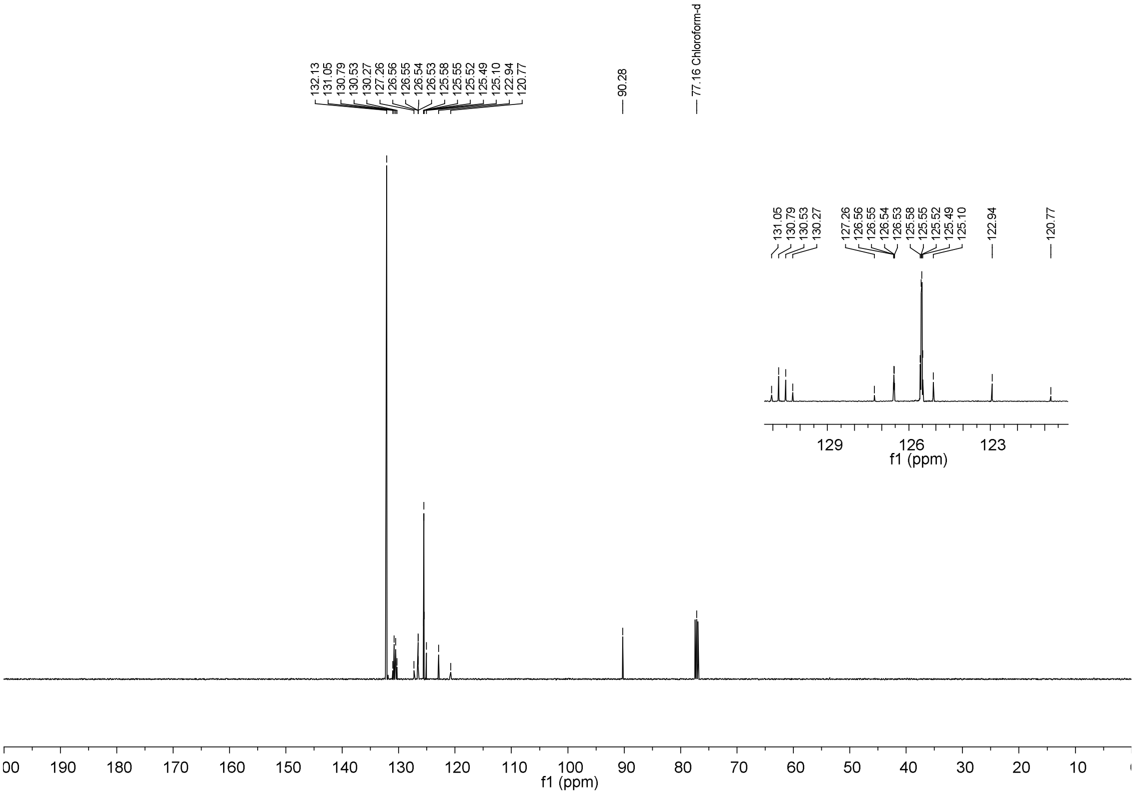

1,2-Bis(3-methoxyphenyl)ethyne (V)

^{1}H NMR (acetone-d$_6$, 300 MHz)

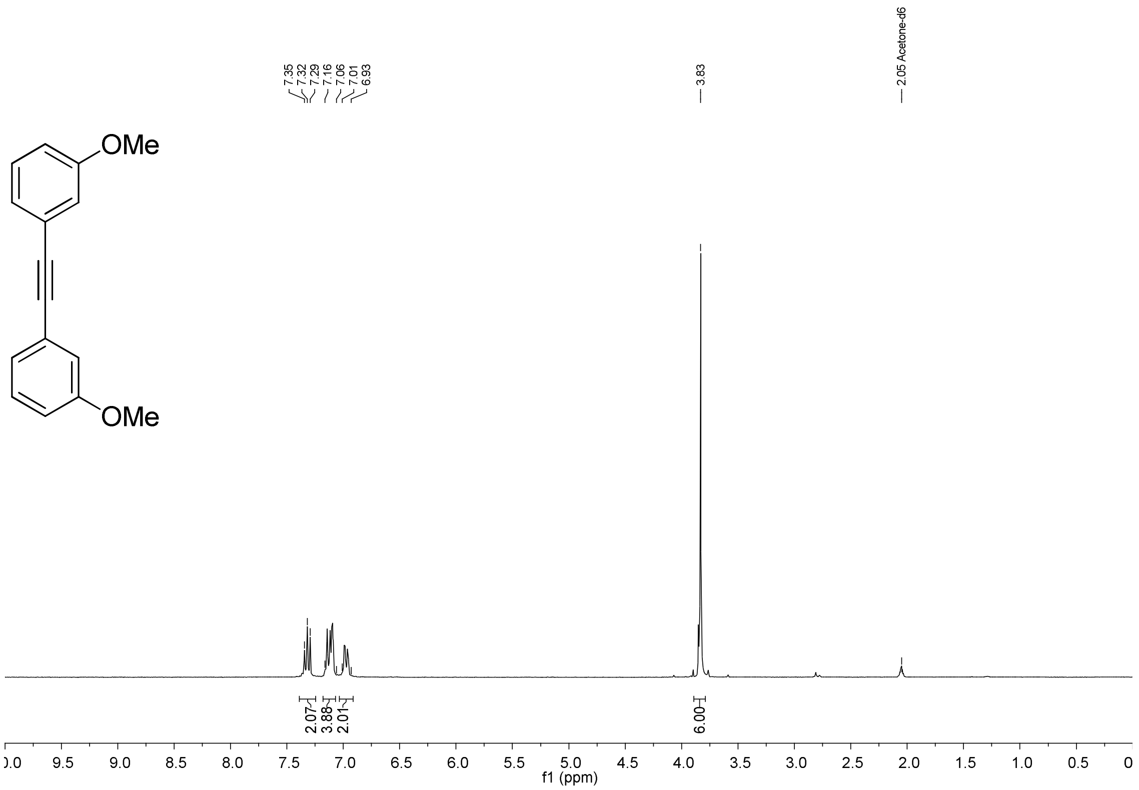

^{13}C NMR (acetone-d$_6$, 75 MHz)

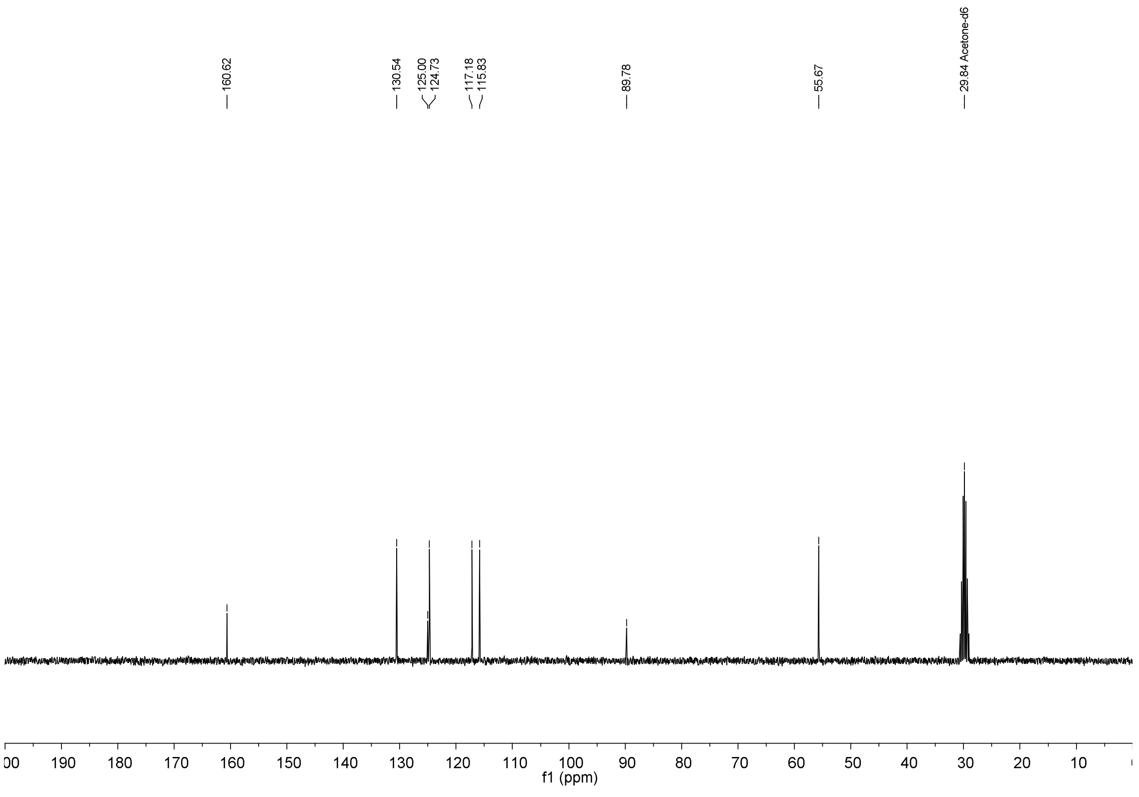

1,2-Di-*o*-tolylethyne (VI)

^{1}H NMR (acetone-d_6, 300 MHz)

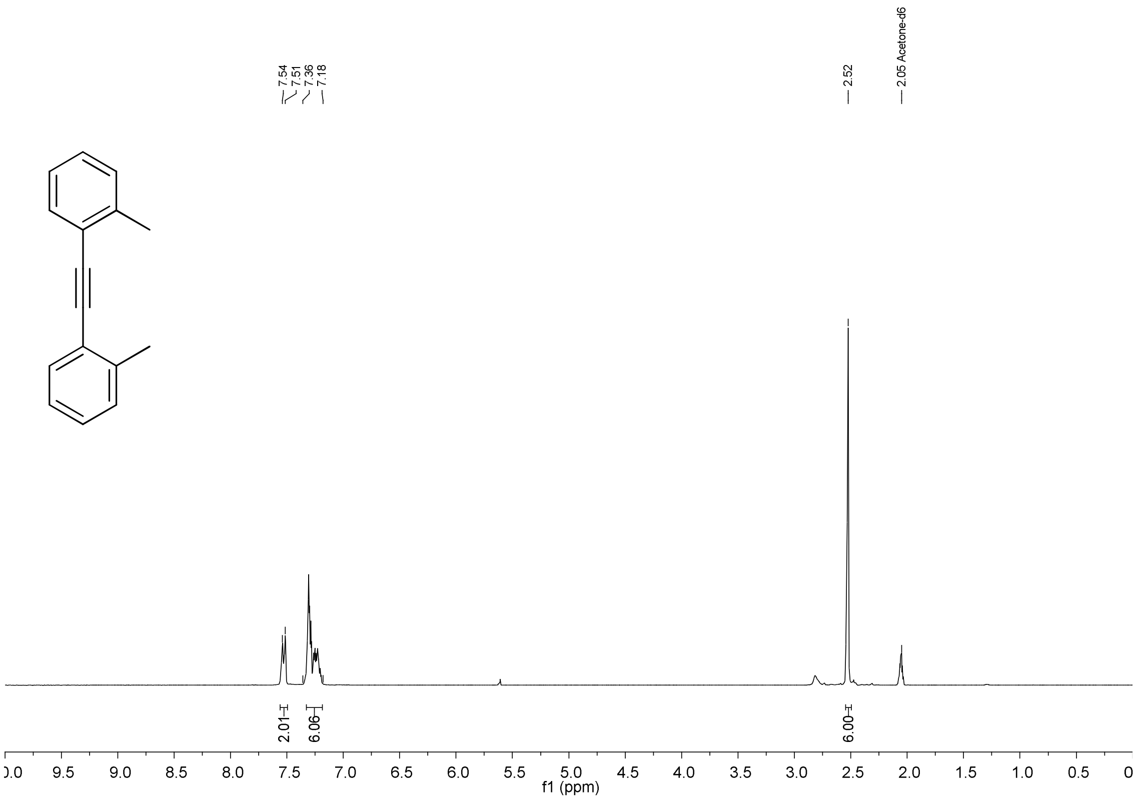

^{13}C NMR (acetone-d_6, 75 MHz)

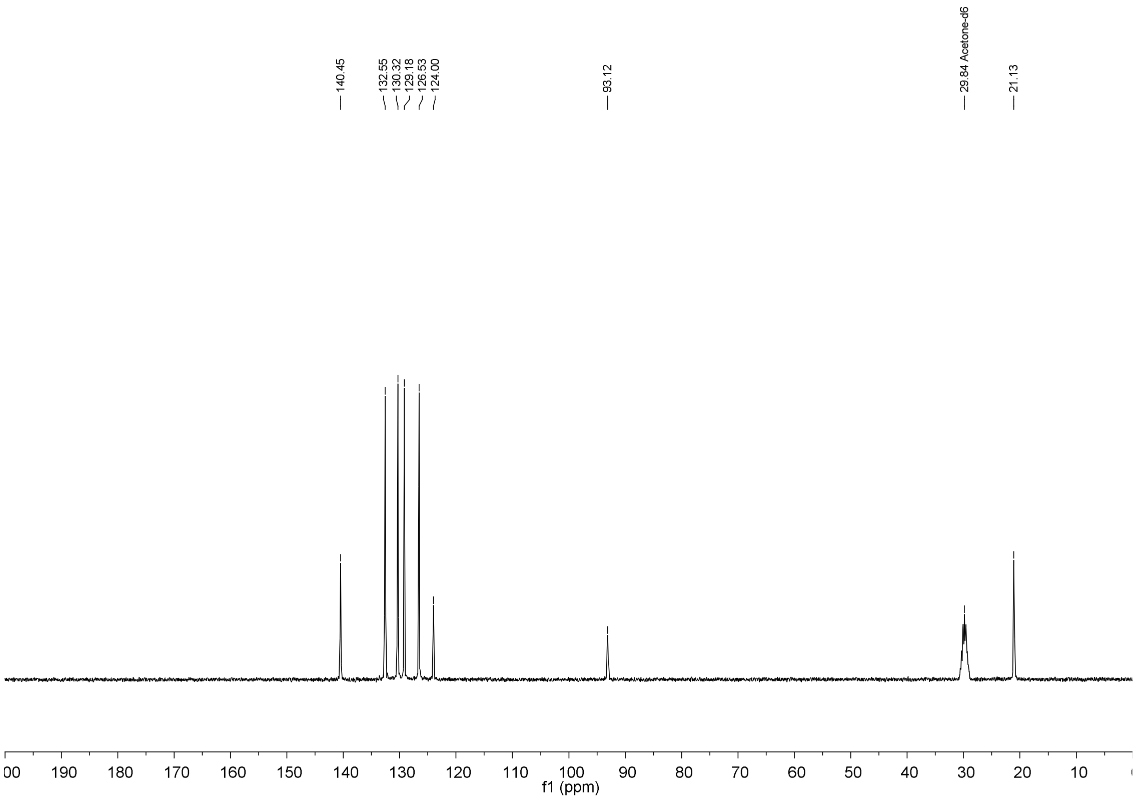

1-(Cyclohexylethynyl)-4-methoxybenzene (VII)

^{1}H NMR ($CDCl_3$, 300 MHz)

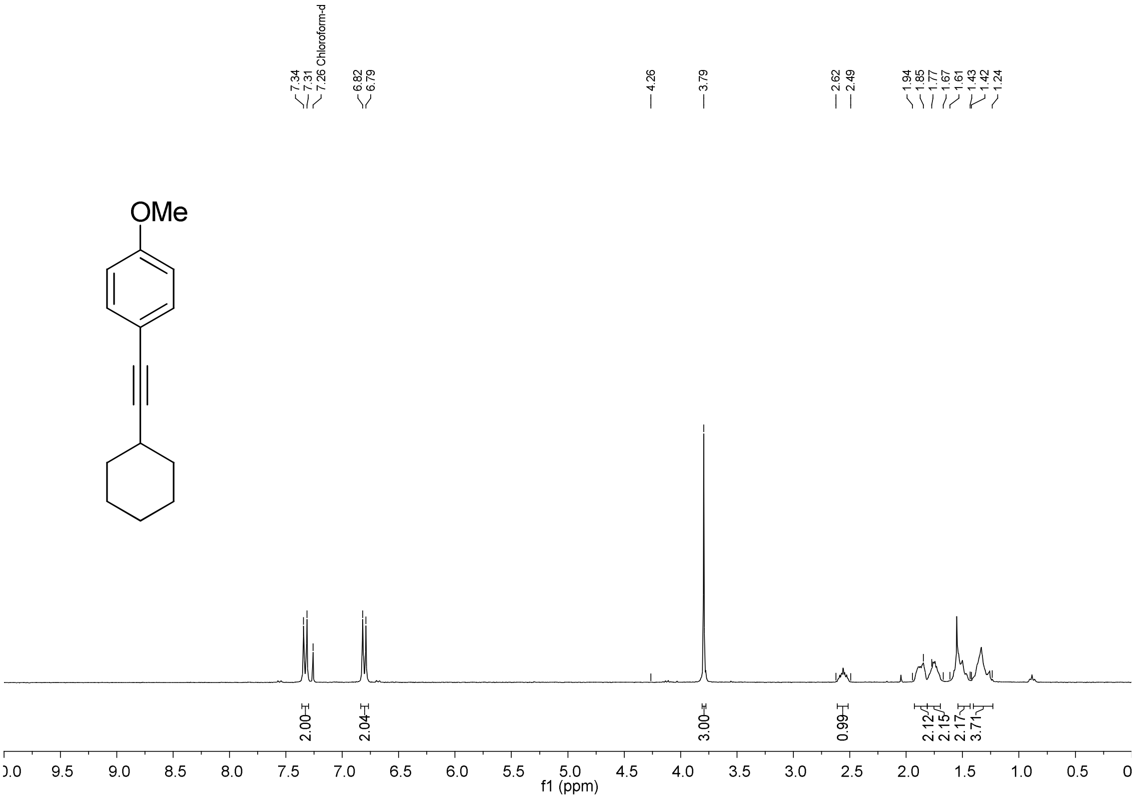

^{13}C NMR ($CDCl_3$, 75 MHz)

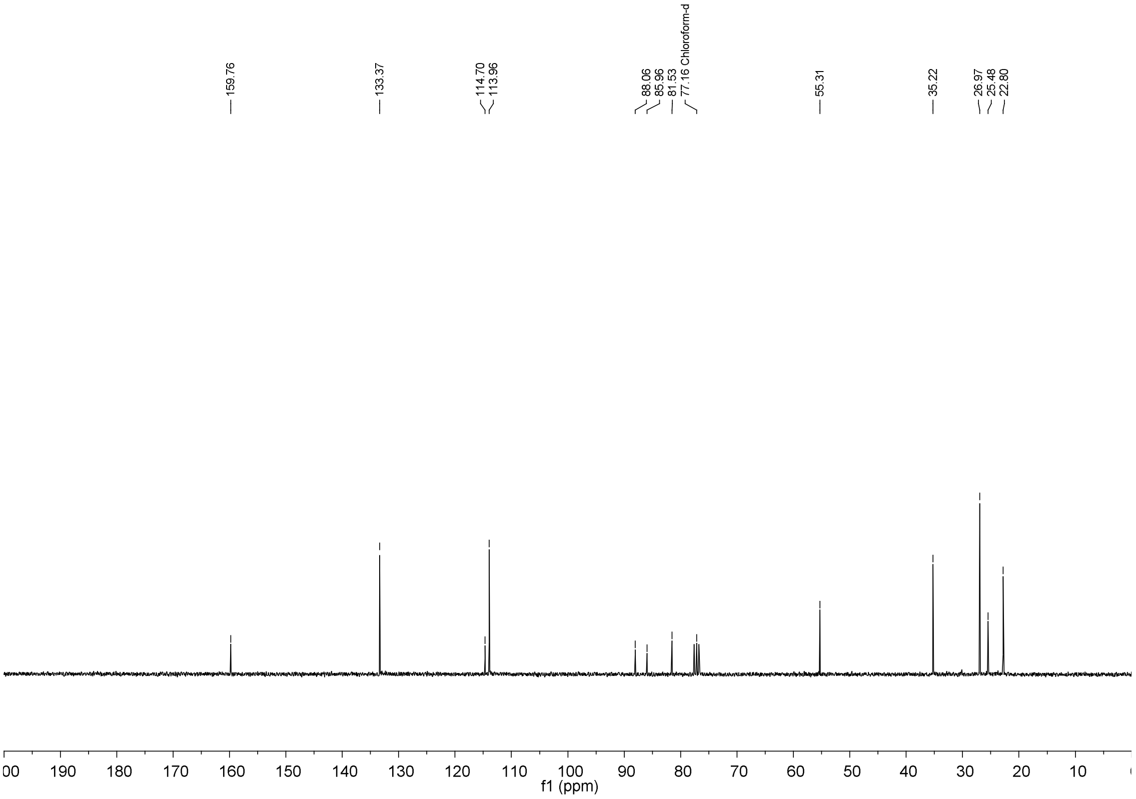

1-(Cyclohexylethynyl)-4-(trifluoromethyl)benzene (VIII)

^{1}H NMR ($CDCl_3$, 300 MHz)

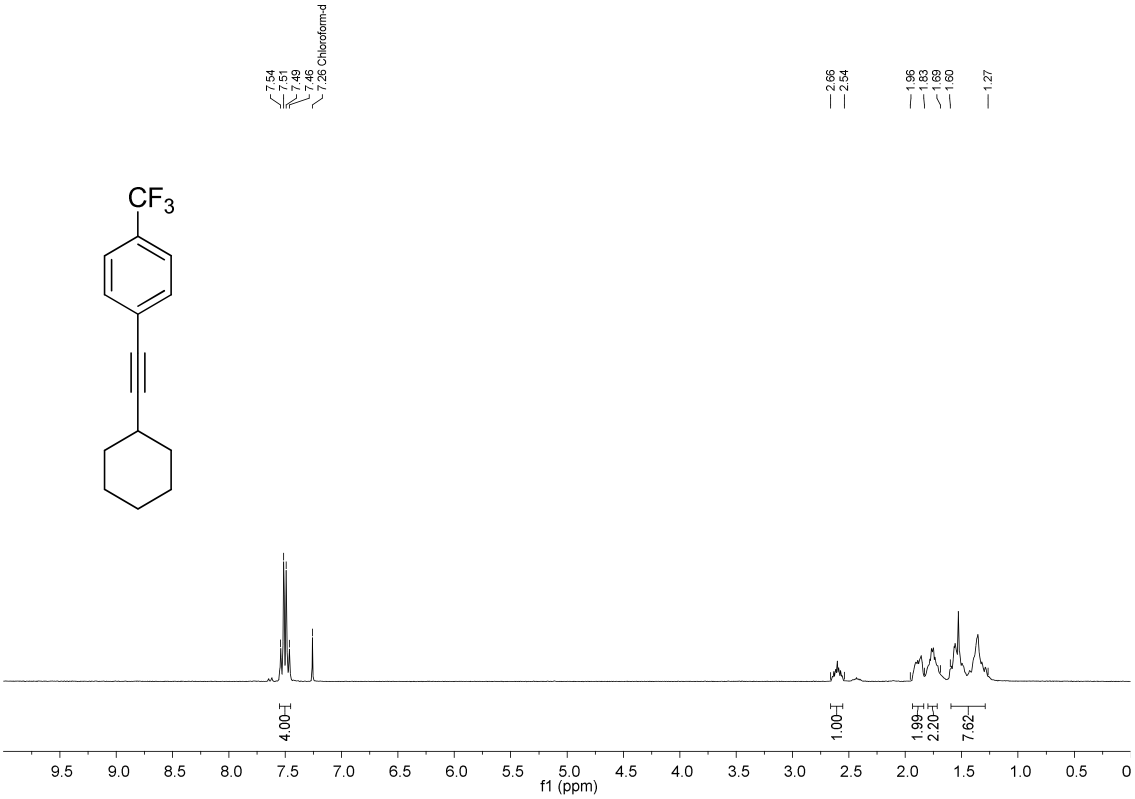

^{13}C NMR ($CDCl_3$, 126 MHz)

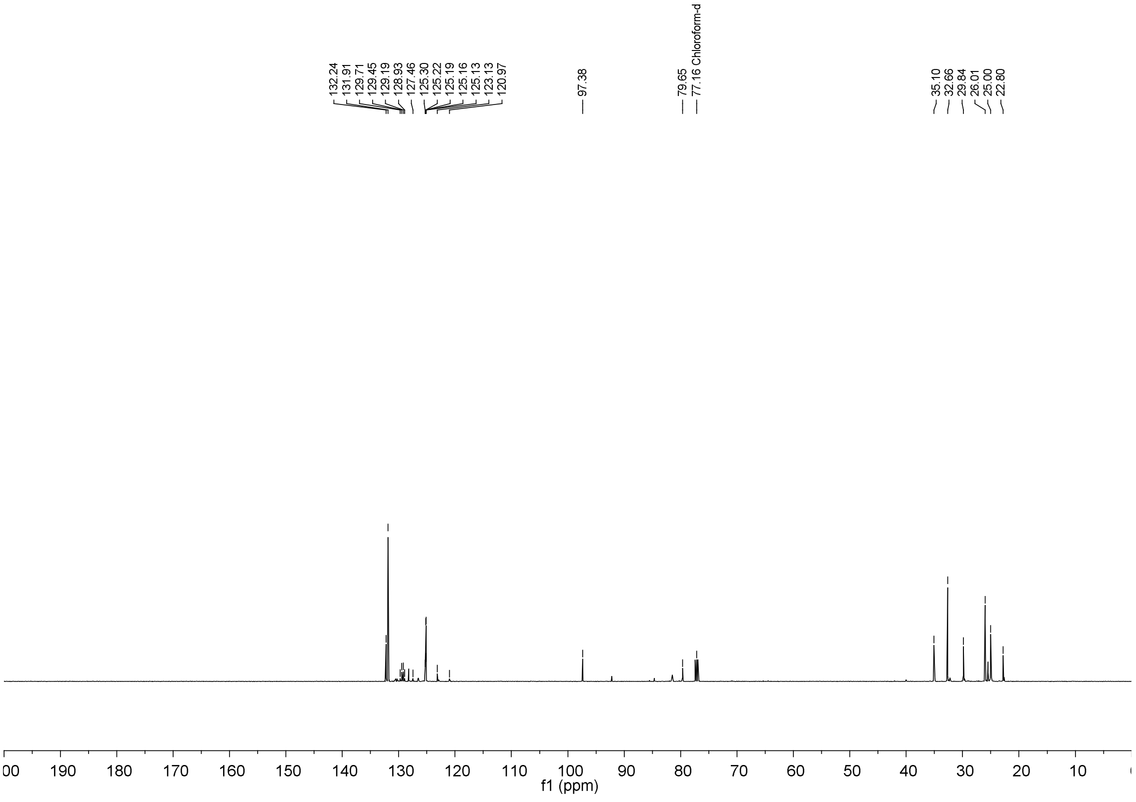

2-(Cyclohexylethynyl)thiophene (IX)

^{1}H NMR ($CDCl_3$, 300 MHz)

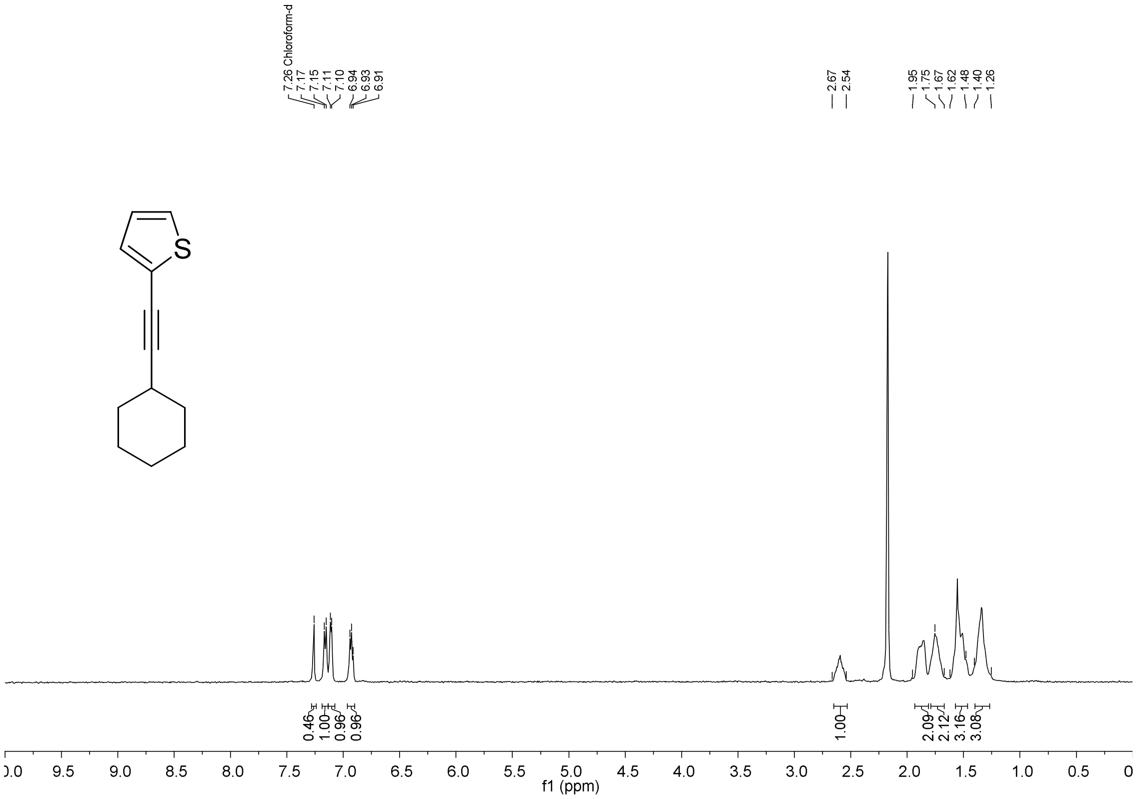

^{13}C NMR (acetone-d_6, 75 MHz)

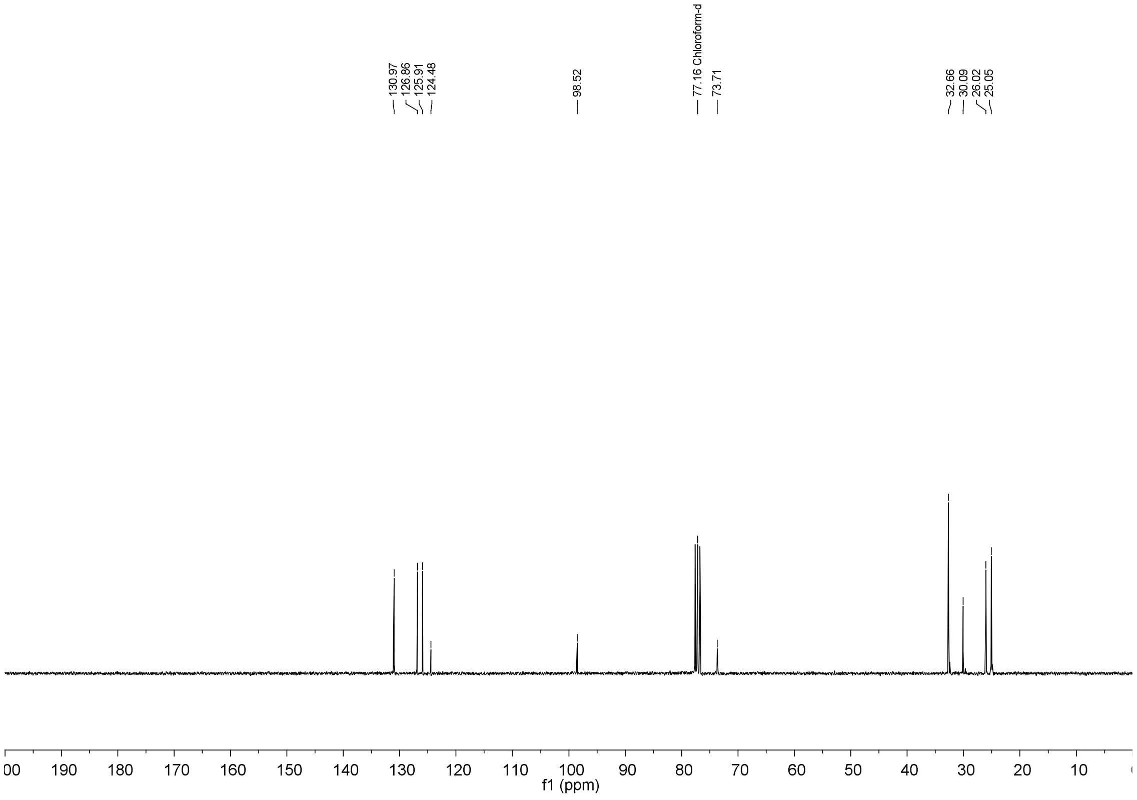

(*E*)-Methyl 6-cyclohexylhex-2-en-4-ynoate (X)

^{1}H NMR ($CDCl_3$, 300 MHz)

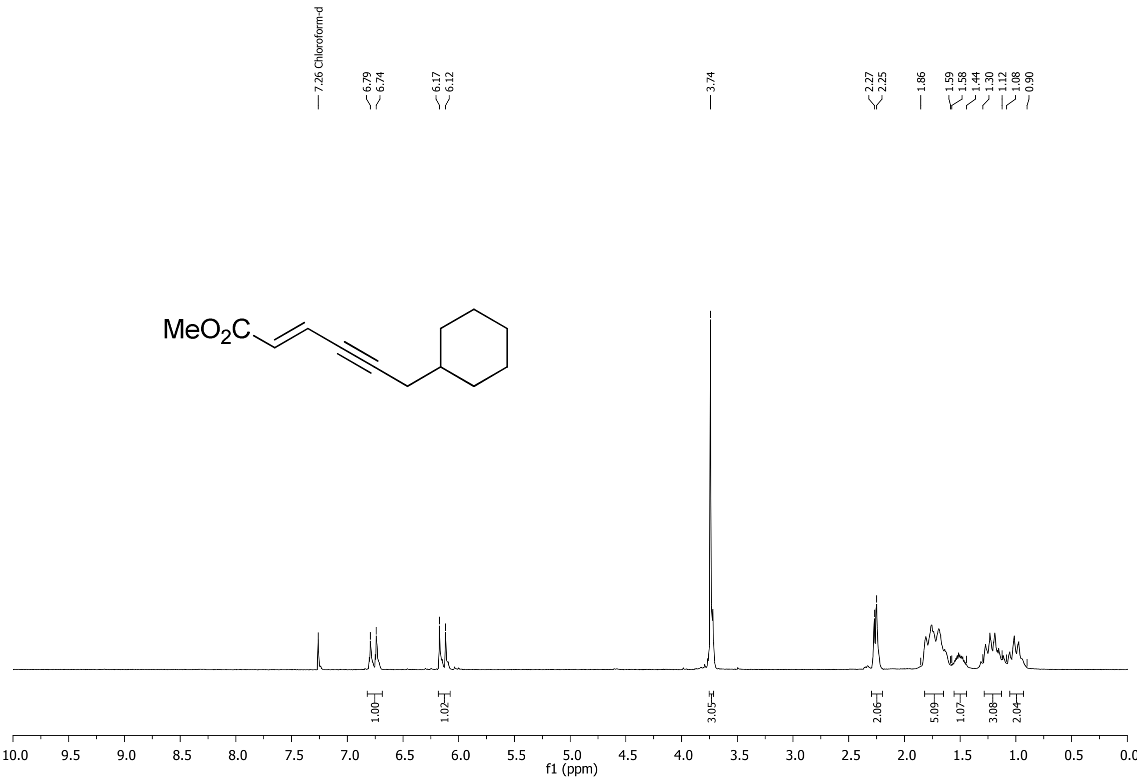

^{13}C NMR ($CDCl_3$, 75 MHz)

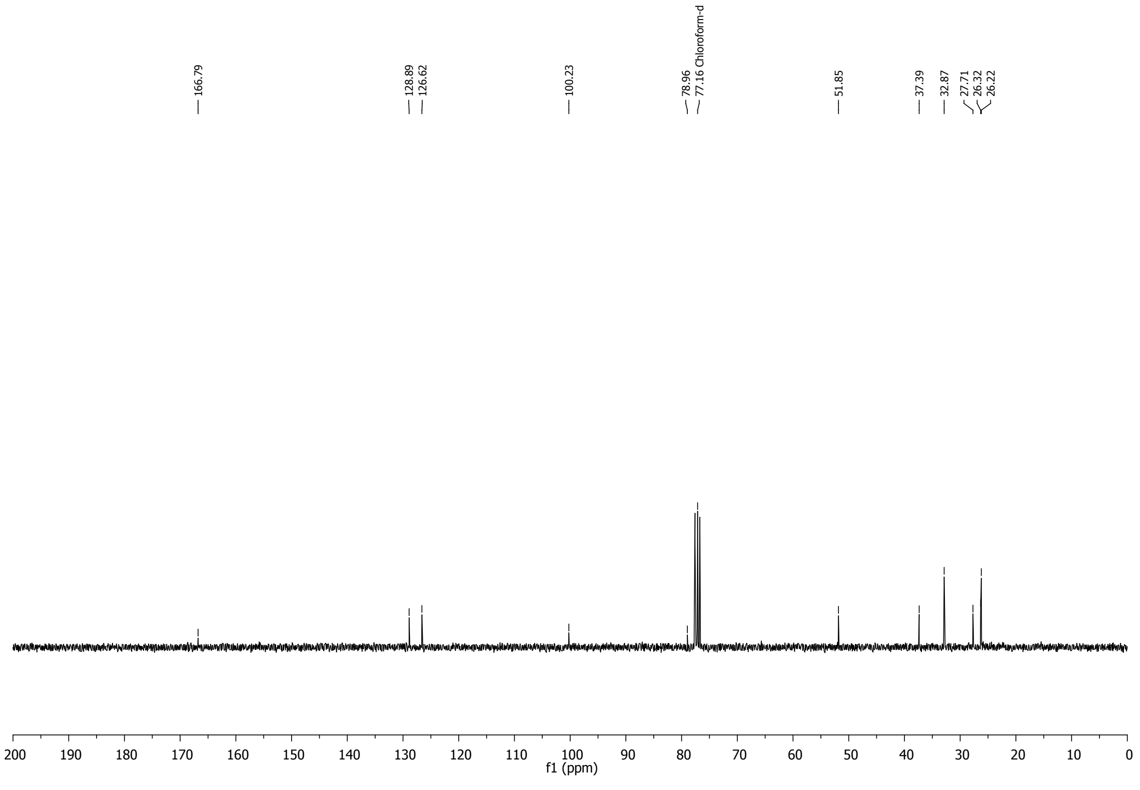

(*E*)-Methyl 7-phenylhept-2-en-4-ynoate (XI)

^{1}H NMR ($CDCl_3$, 300 MHz)

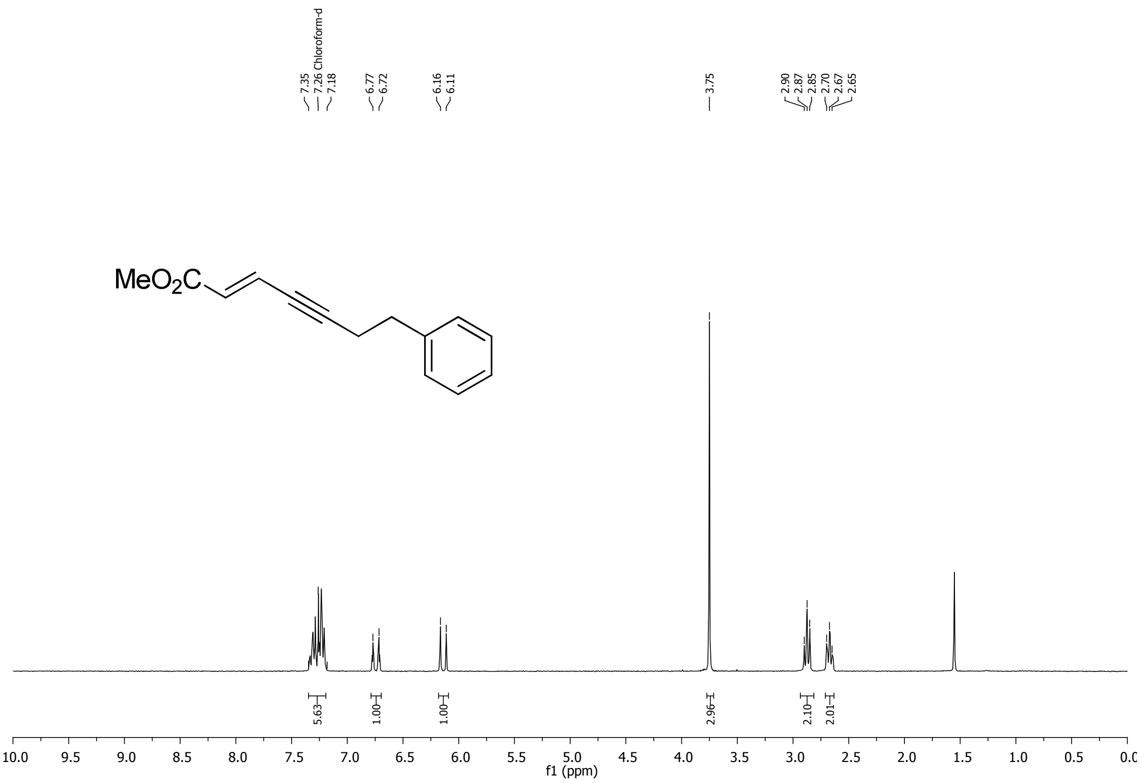

^{13}C NMR ($CDCl_3$, 75 MHz)

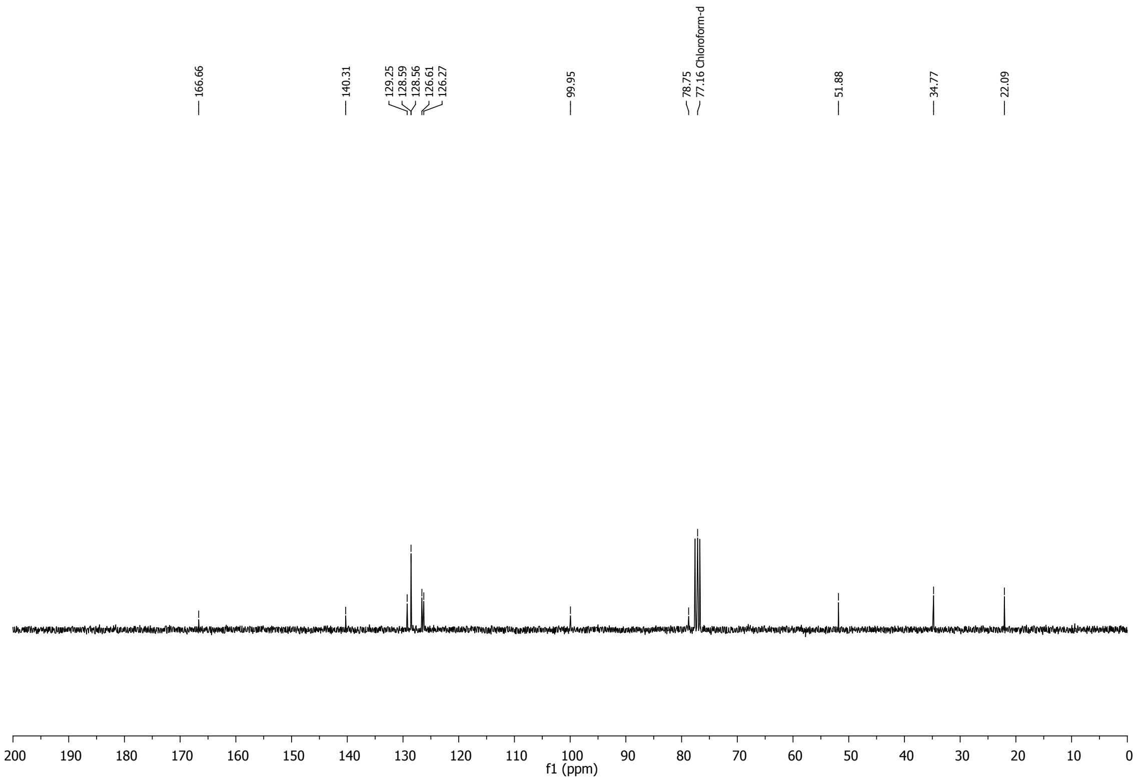

(*E*)-Methyl 9-chloronon-2-en-4-ynoate (XII)

^{1}H NMR ($CDCl_3$, 300 MHz)

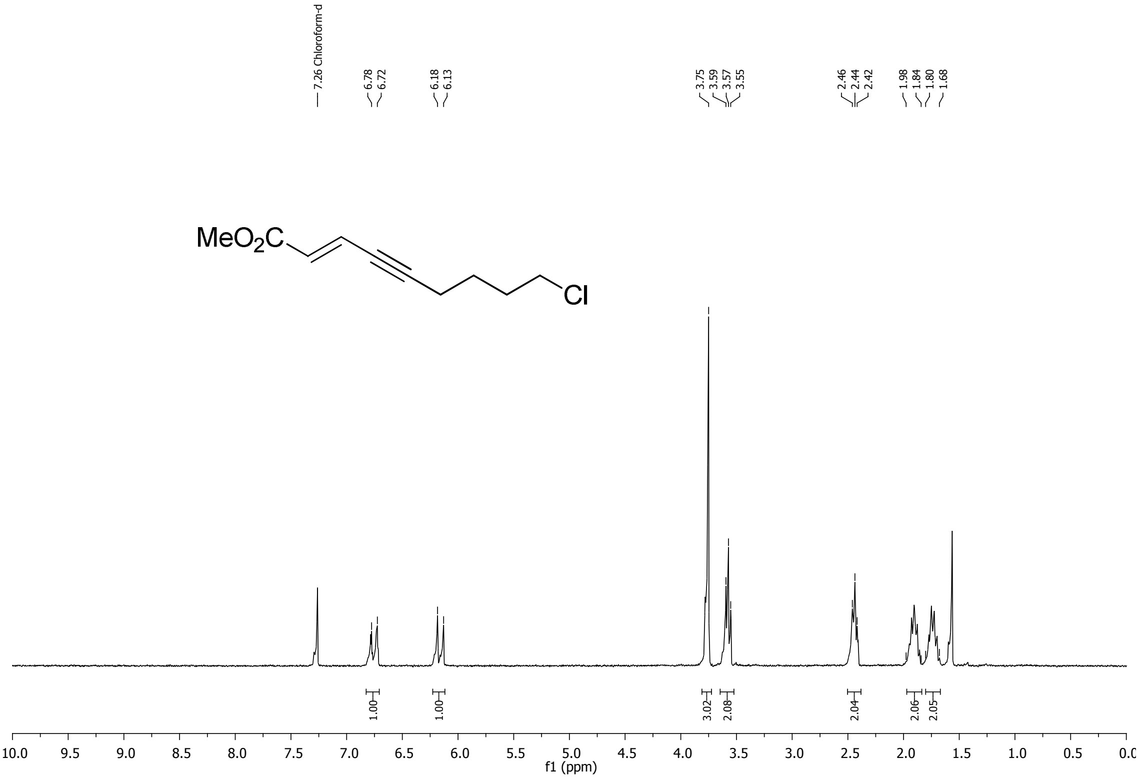

^{13}C NMR ($CDCl_3$, 75 MHz)

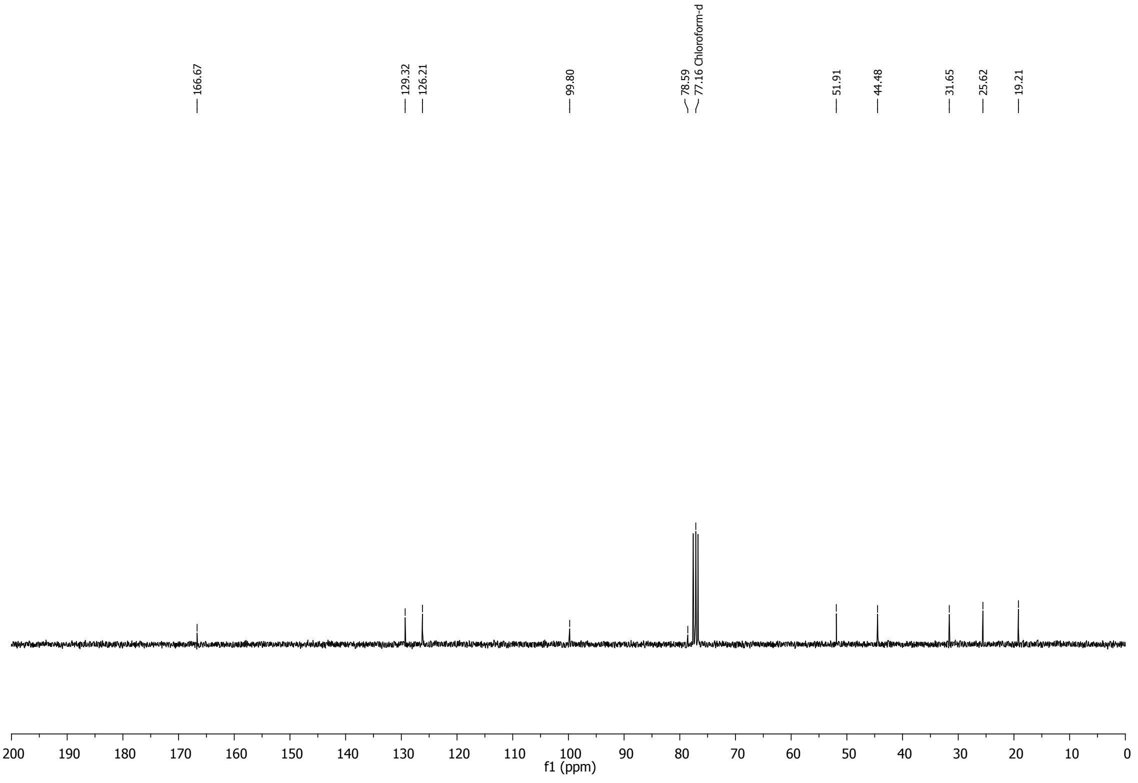

***N*-Benzyl-5,6,7,8-tetraphenylisoquinoline-1-carboxamide (2)**

^{1}H NMR (methanol-d_4, 500 MHz)

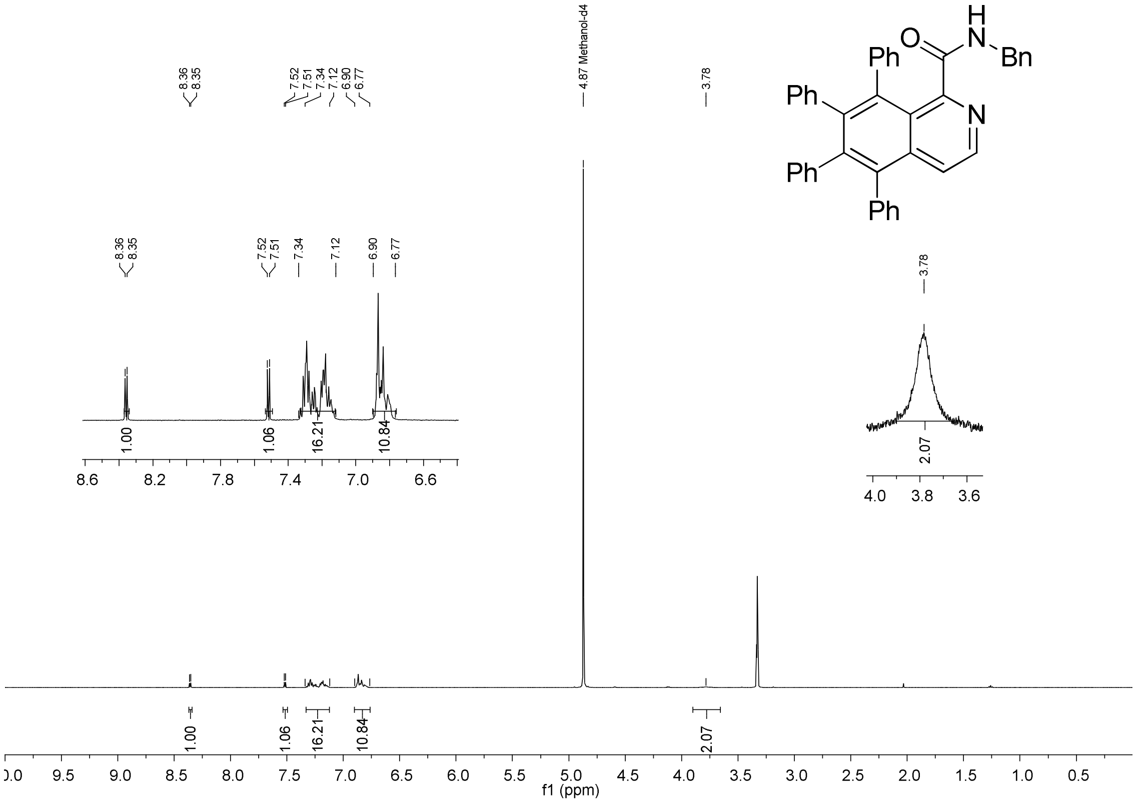

^{13}C NMR ($CDCl_3$, 125 MHz)

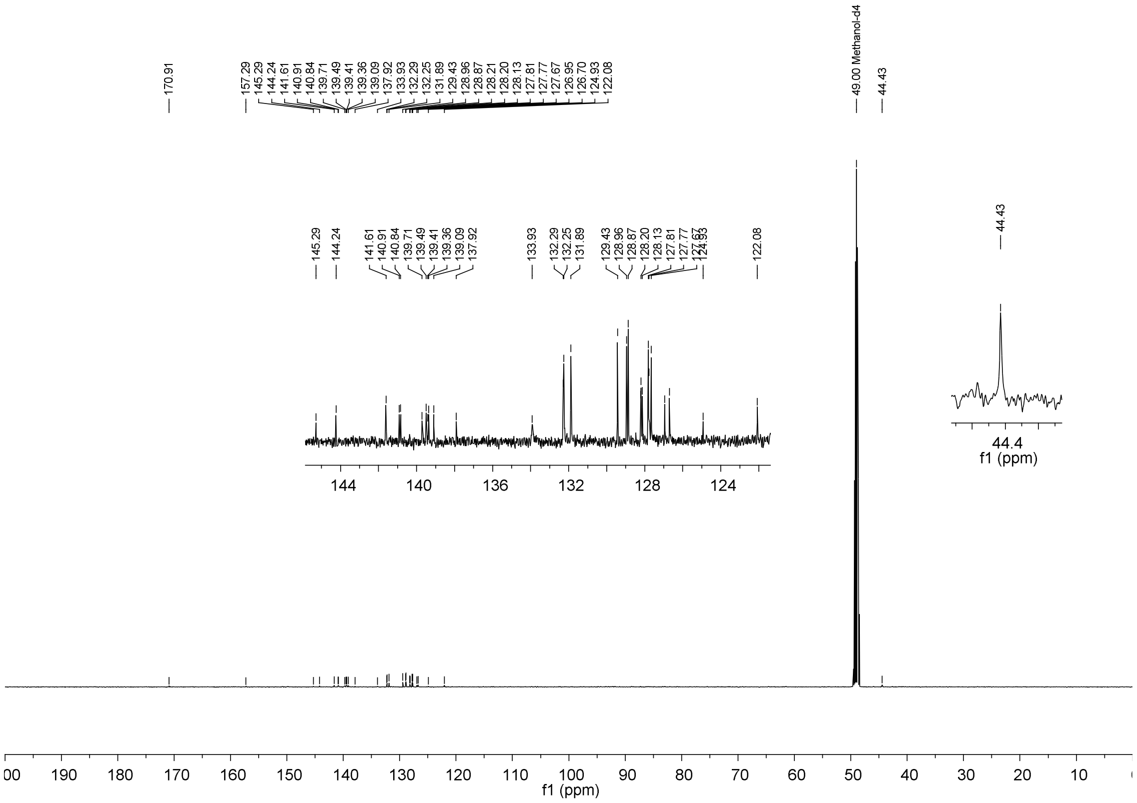

_N_-Benzyl-3-methyl-5,6,7,8-tetraphenylisoquinoline-1-carboxamide (14)

^{1}H NMR ($CDCl_3$, 300 MHz)

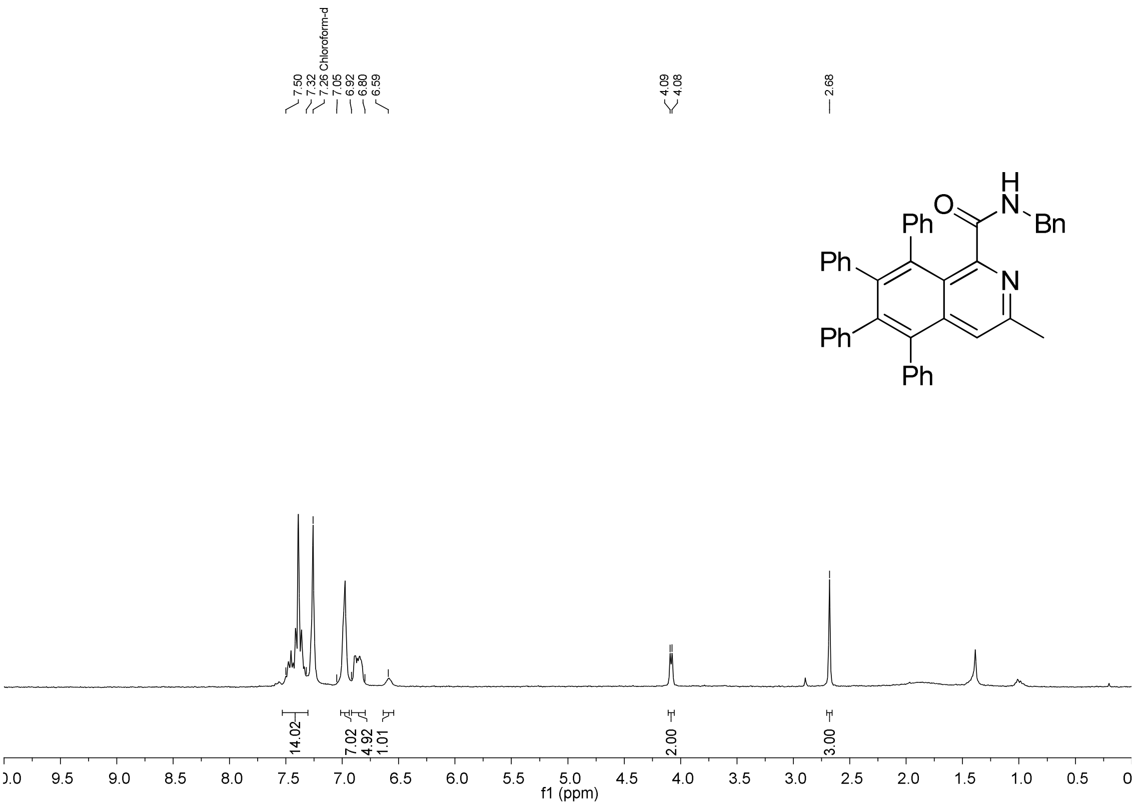

^{13}C NMR ($CDCl_3$, 75 MHz)

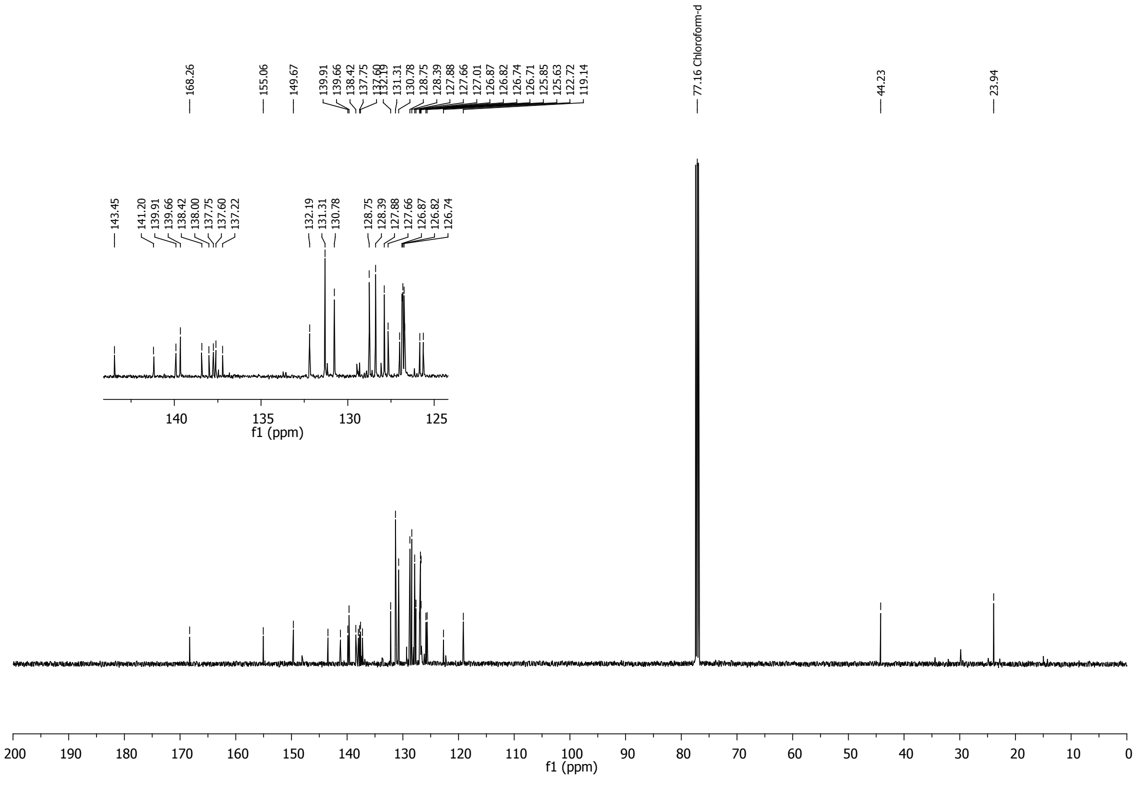

_N_-Benzyl-3-chloro-5,6,7,8-tetraphenylisoquinoline-1-carboxamide (15)

^{1}H NMR ($CDCl_3$, 300 MHz)

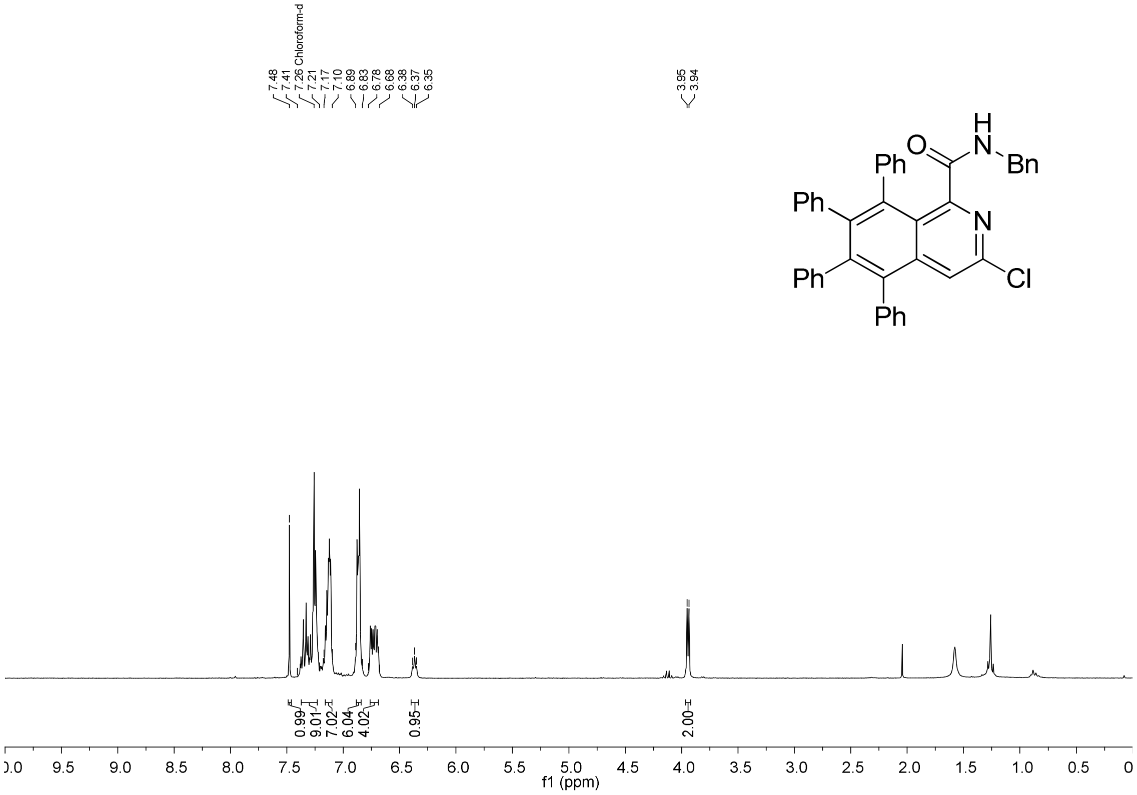

^{13}C NMR ($CDCl_3$, 75 MHz)

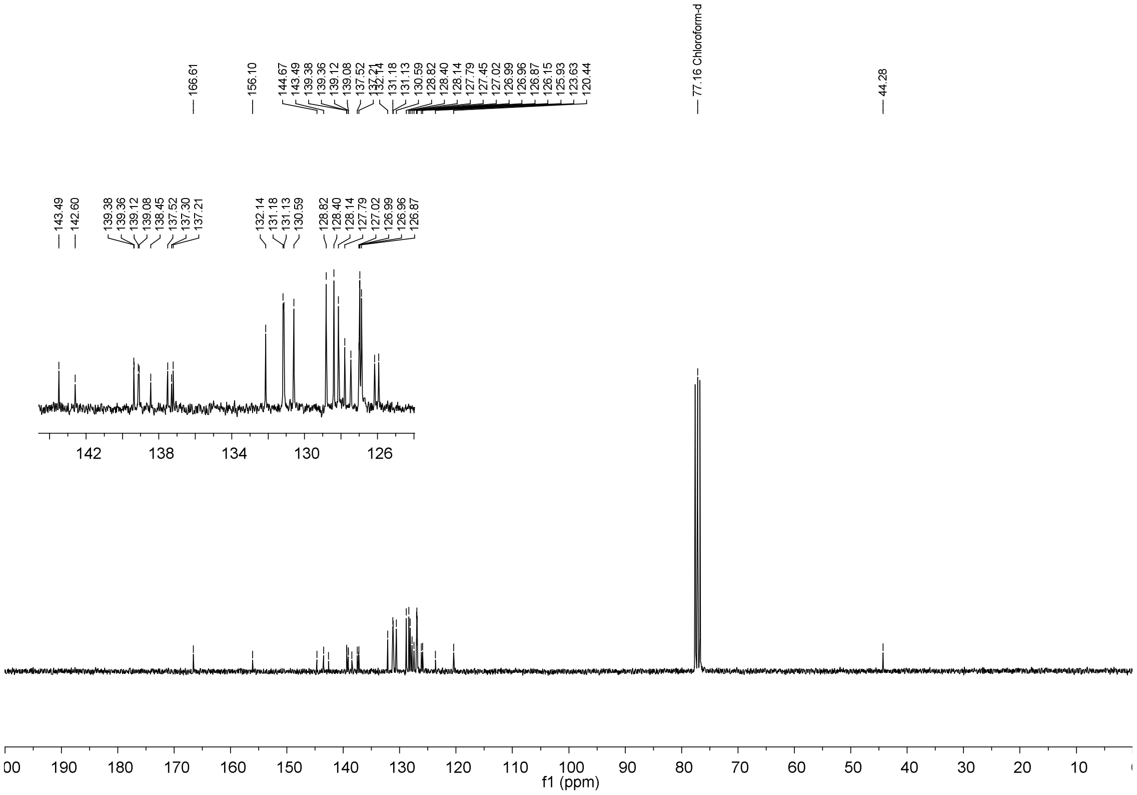

7-Benzyl-5,6-diphenyl-3-(trifluoromethyl)-1,7-naphthyridin-8(7H)-one (16)

^{1}H NMR ($CDCl_3$, 300 MHz)

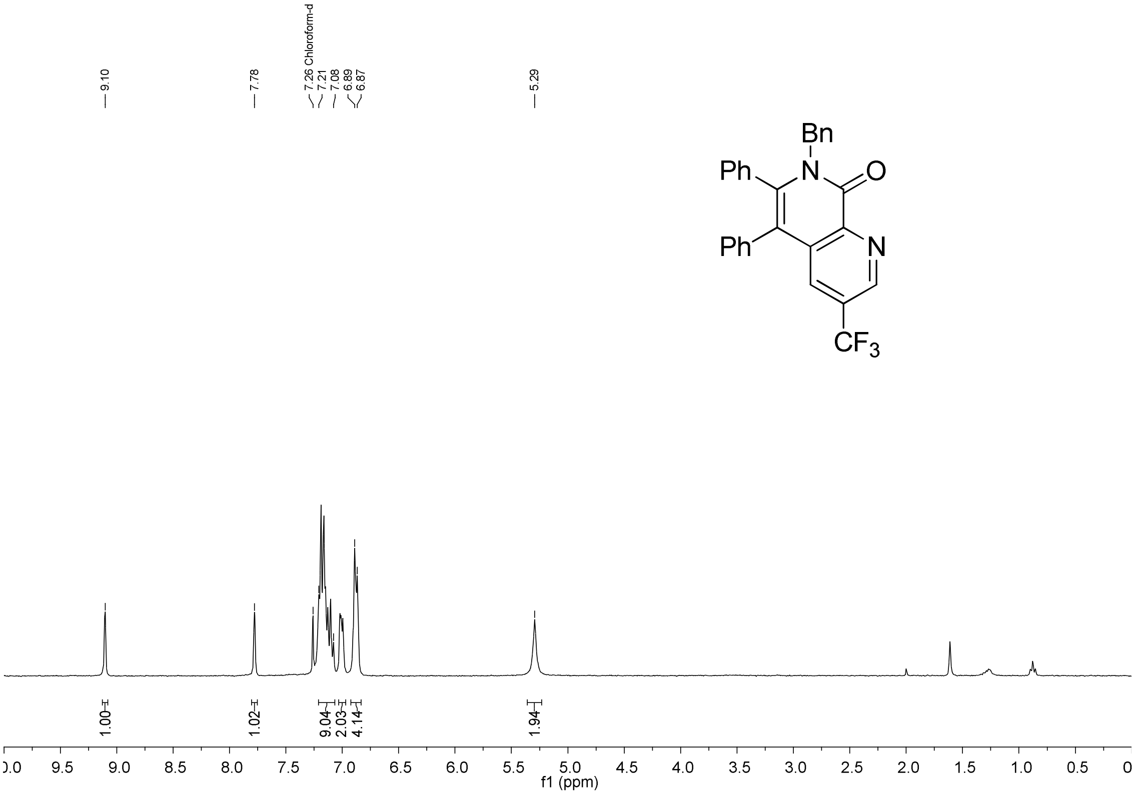

^{13}C NMR ($CDCl_3$, 125 MHz)

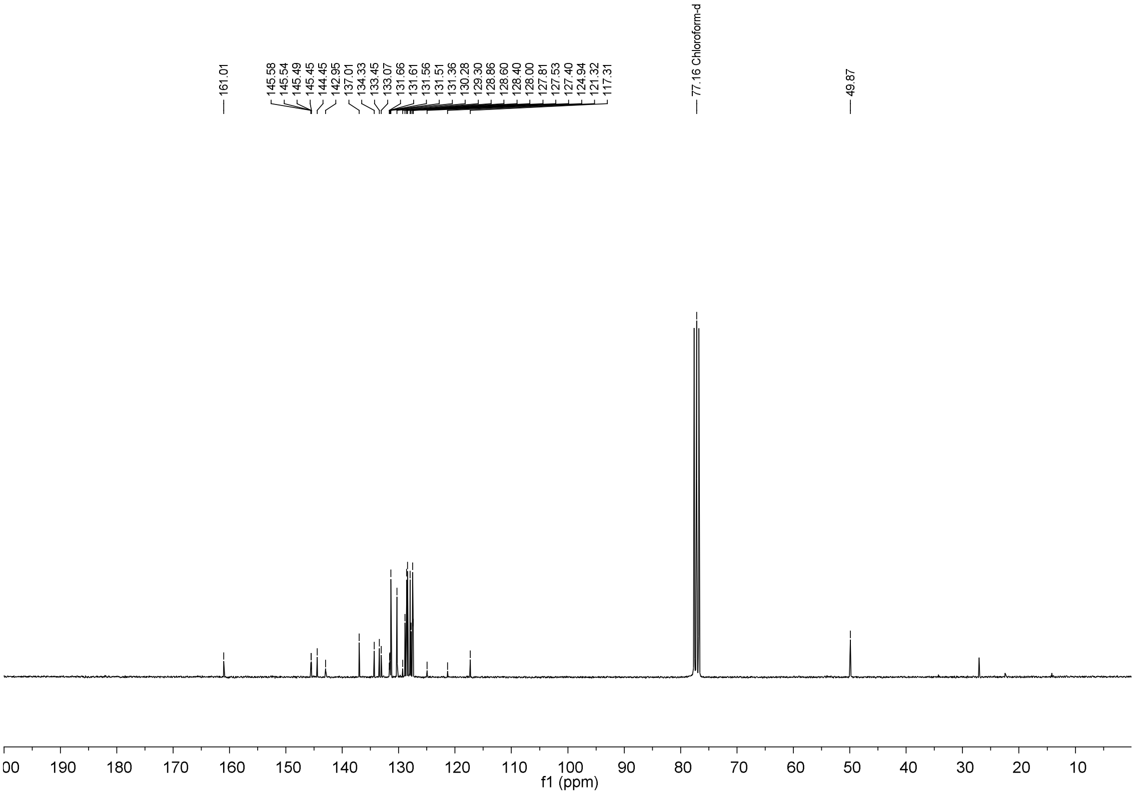

^{19}F NMR ($CDCl_3$, 282 MHz)

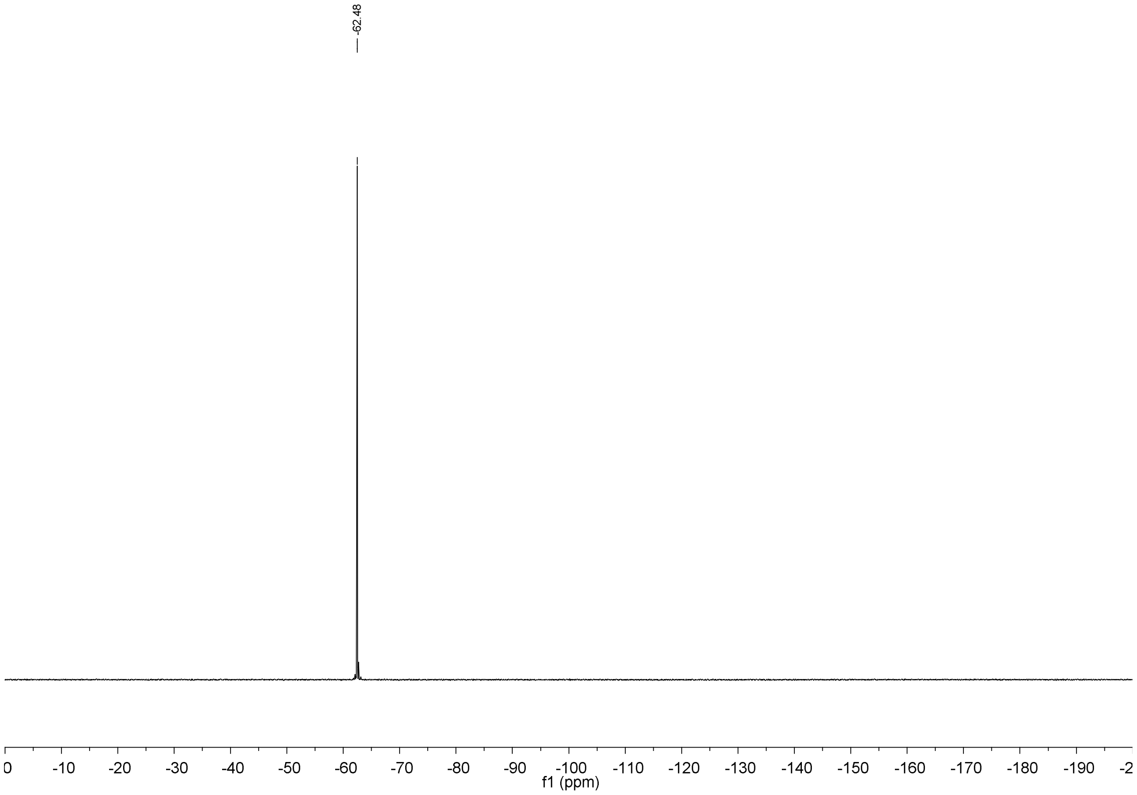

***N*-Benzyl-7,8,9,10-tetraphenylphenanthridine-6-carboxamide (17)**

^{1}H NMR ($CDCl_3$, 300 MHz)

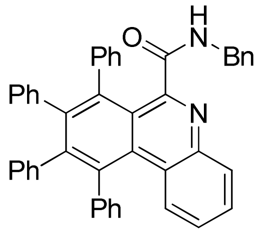

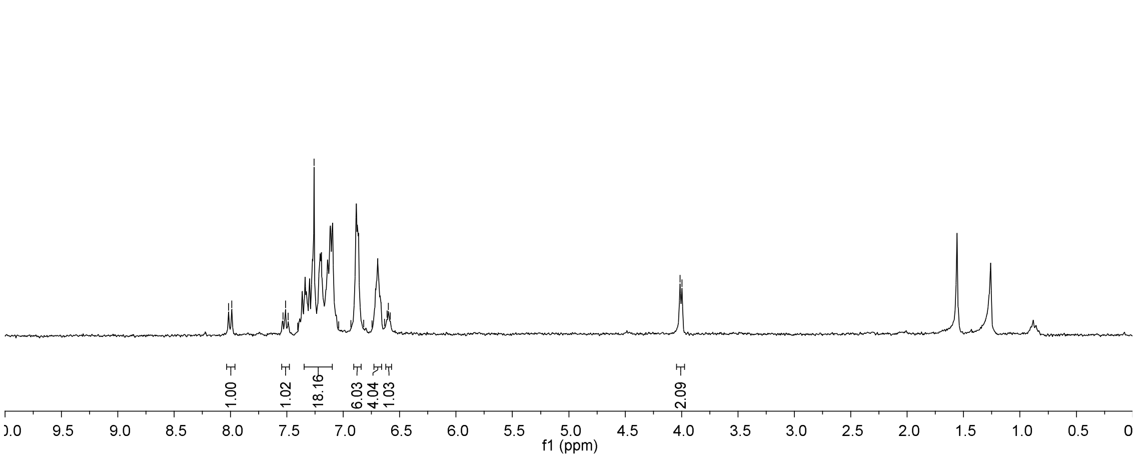

^{13}C NMR ($CDCl_3$, 75 MHz)

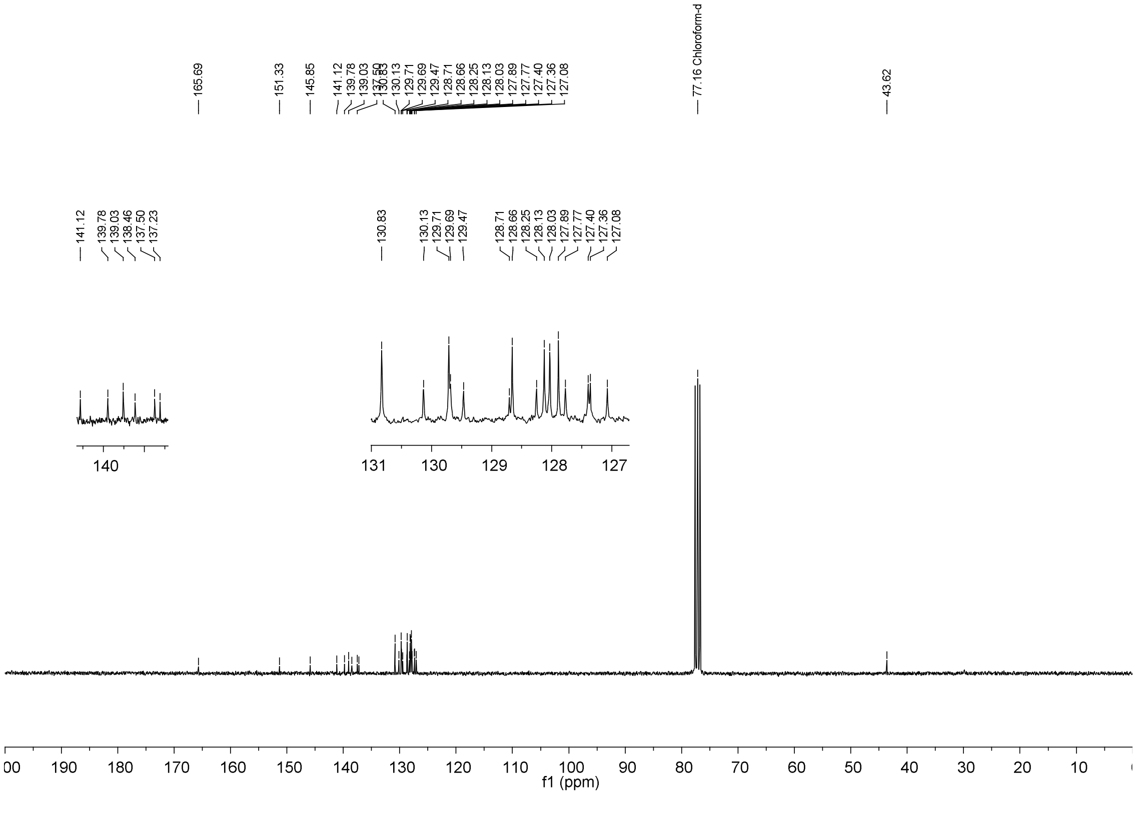

6-Benzyl-2-methyl-4,5-diphenylthieno[2,3-*c*]pyridin-7(6H)-one (18)

^{1}H NMR ($CDCl_3$, 300 MHz)

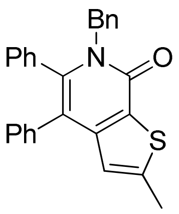

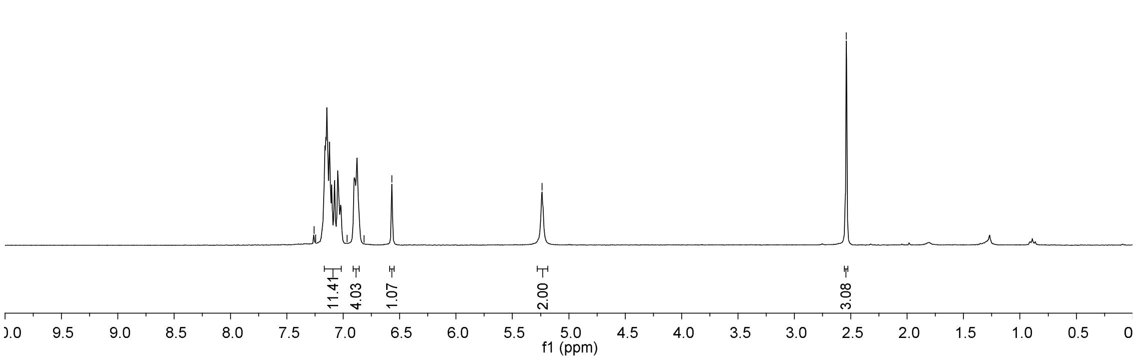

^{13}C NMR ($CDCl_3$, 125 MHz)

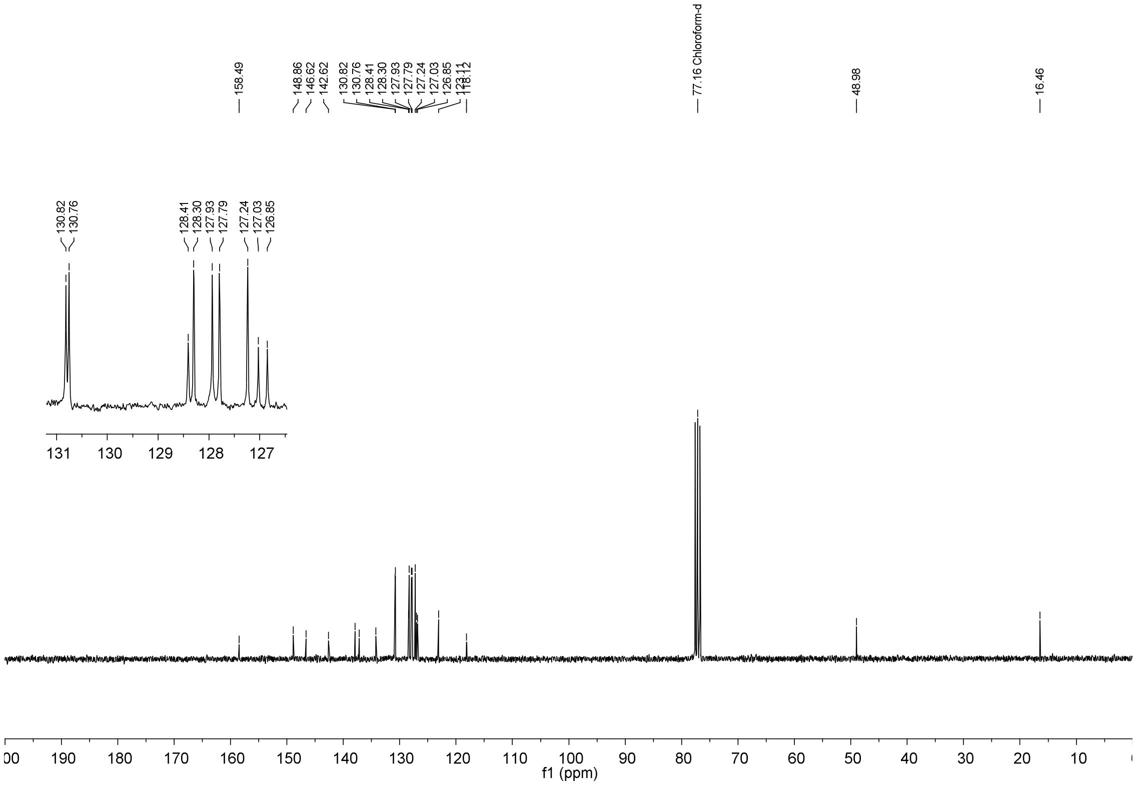

2-Benzyl-3,4-diphenylbenzo[4,5]thieno[2,3-*c*]pyridin-1(2H)-one (19)

^{1}H NMR (CDCl$_3$, 300 MHz)

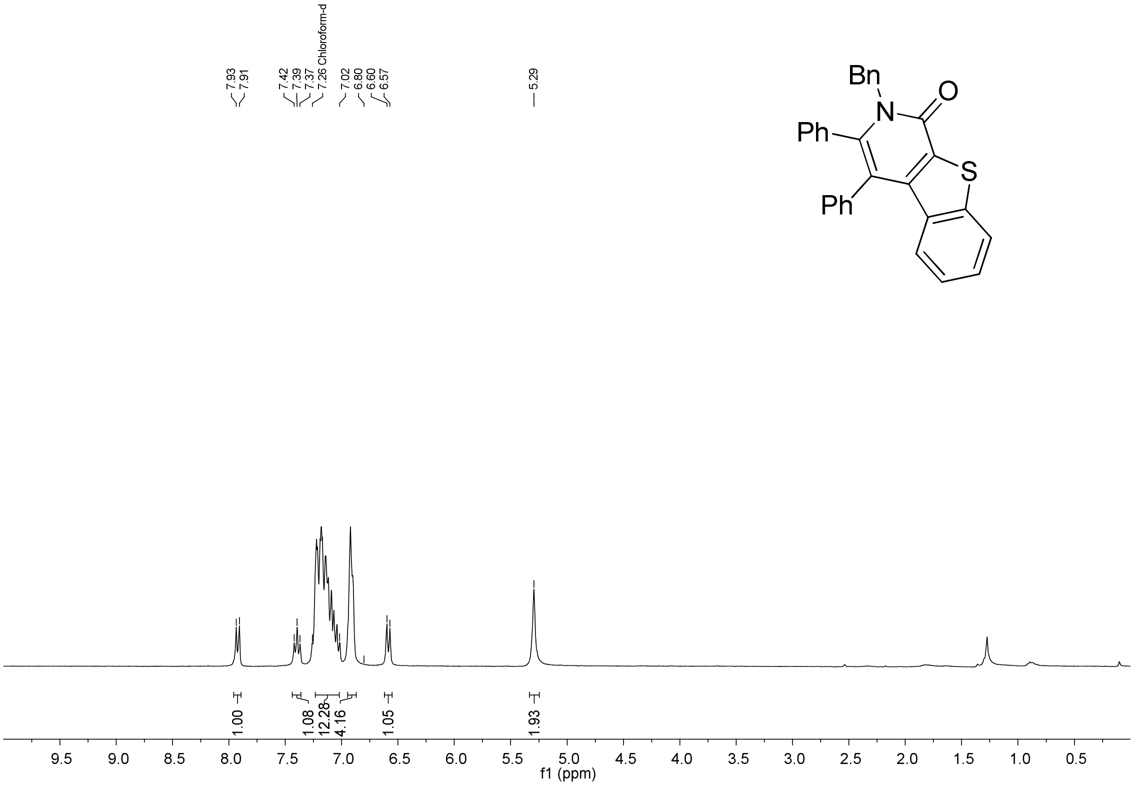

^{13}C NMR (CDCl$_3$, 75 MHz)

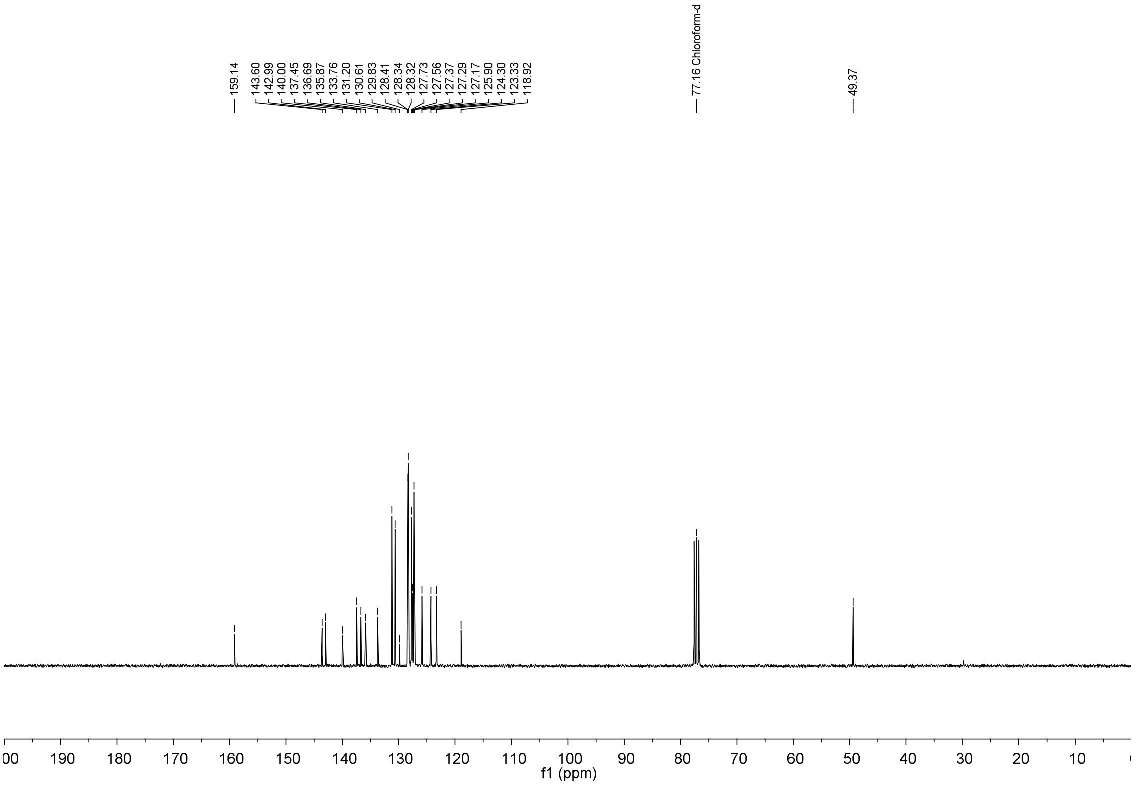

7-Phenethyl-5,6-diphenyl-1,7-naphthyridin-8(7H)-one (88)

^{1}H NMR ($CDCl_3$, 300 MHz)

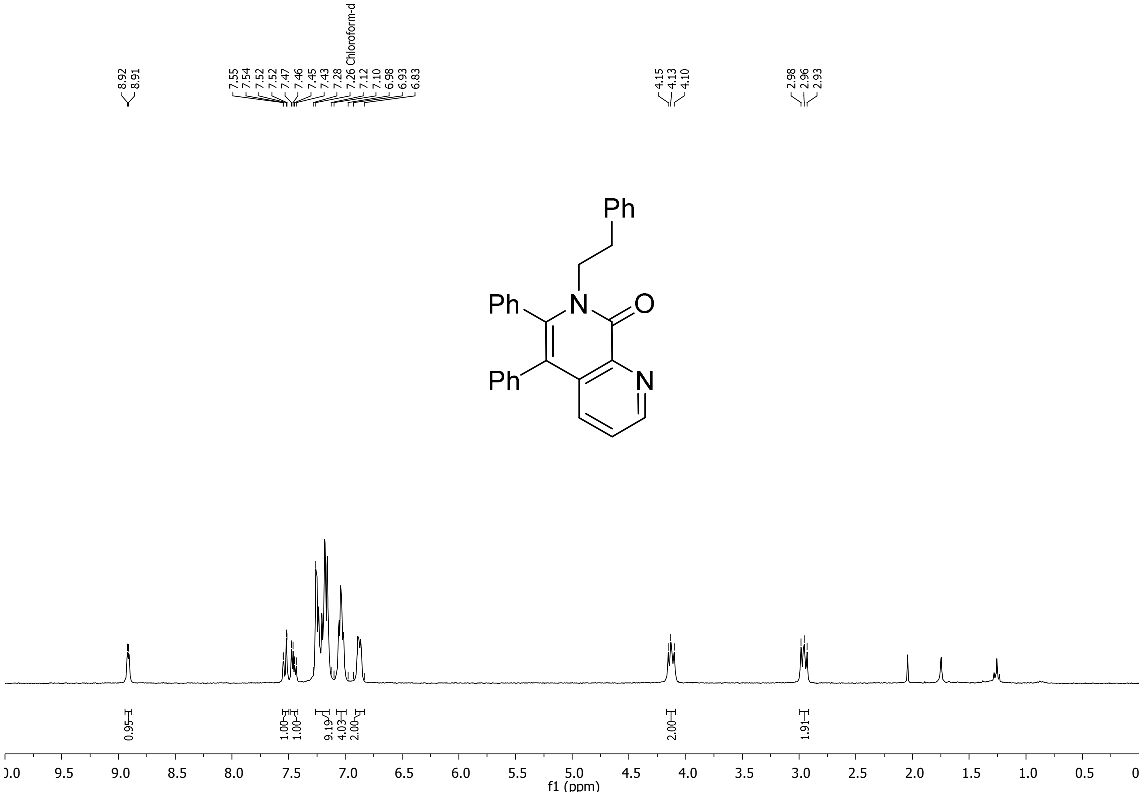

^{13}C NMR ($CDCl_3$, 75 MHz)

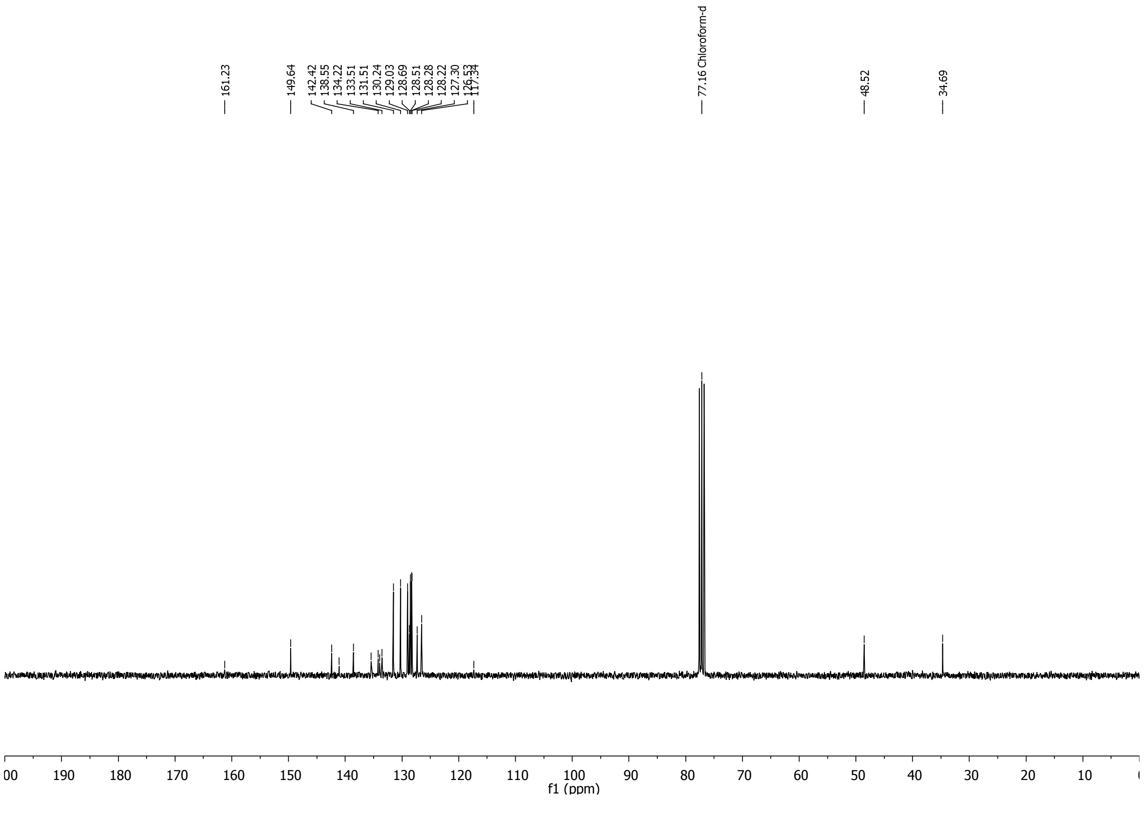

N-Ethyl-5,6,7,8-tetraphenylisoquinoline-1-carboxamide (13)

^{1}H NMR ($CDCl_3$, 300 MHz)

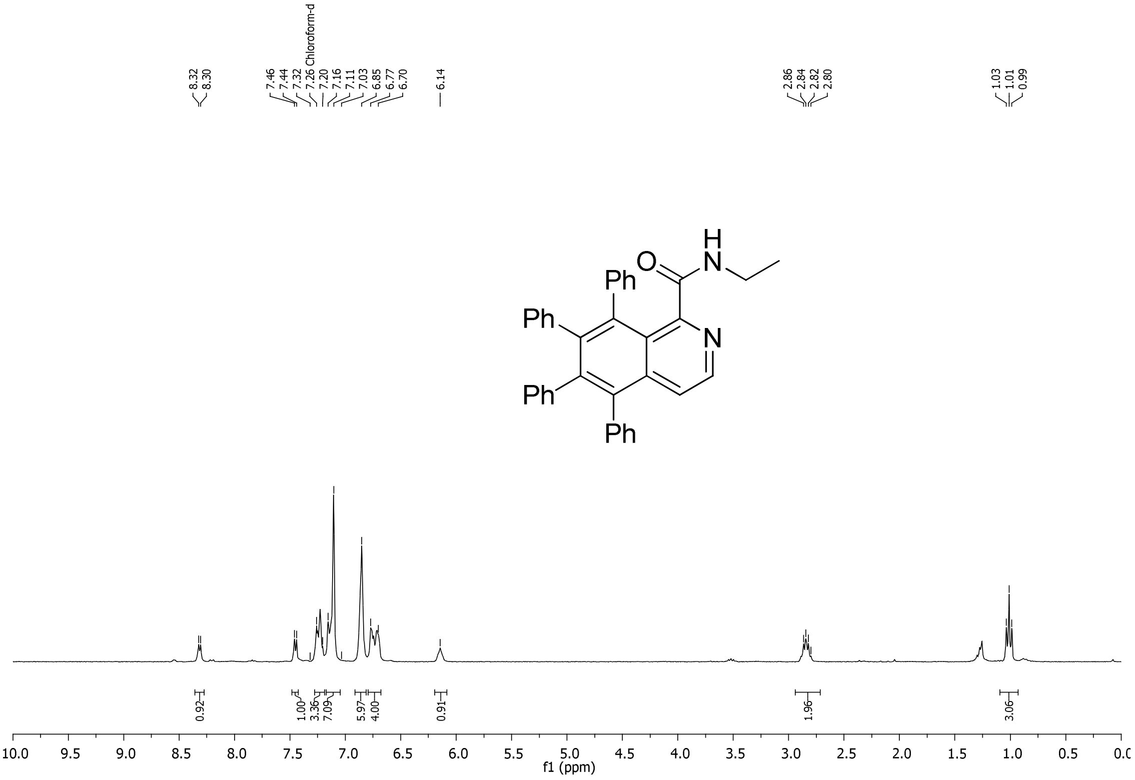

^{13}C NMR ($CDCl_3$, 75 MHz)

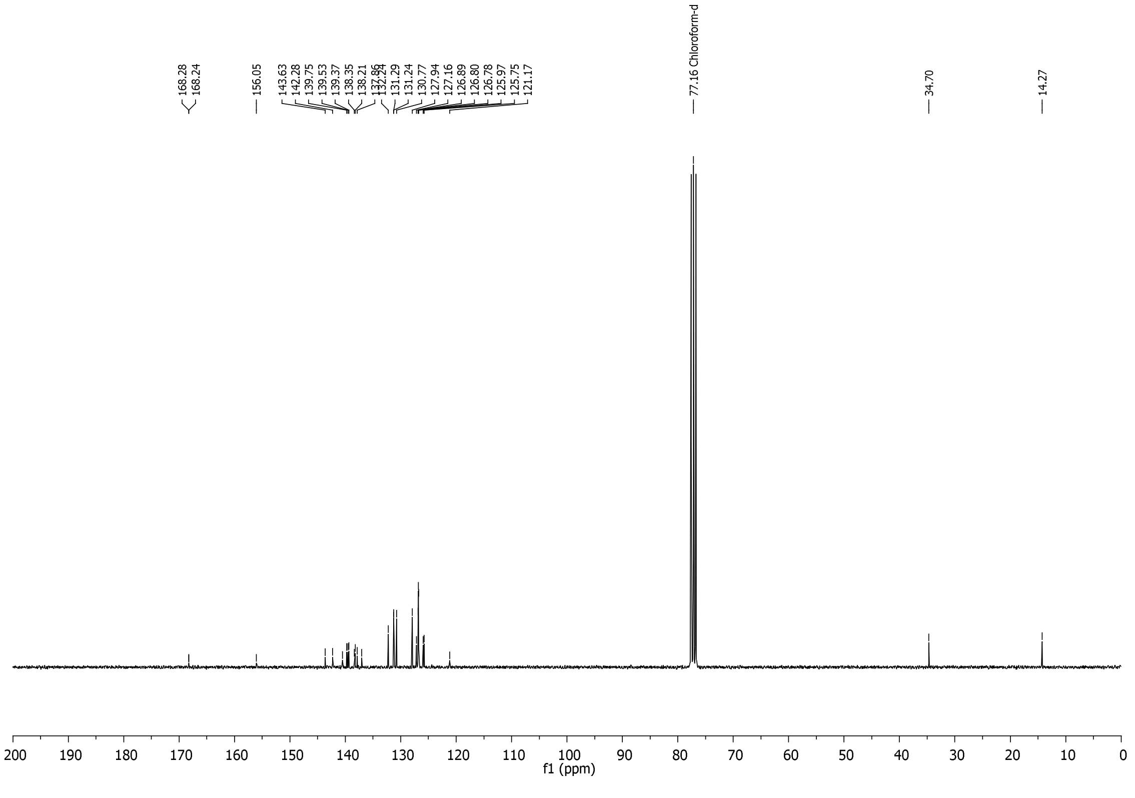

N-Benzyl-5,6,7,8-tetra-*p*-tolylisoquinoline-1-carboxamide (11)

^{1}H NMR ($CDCl_3$, 300 MHz)

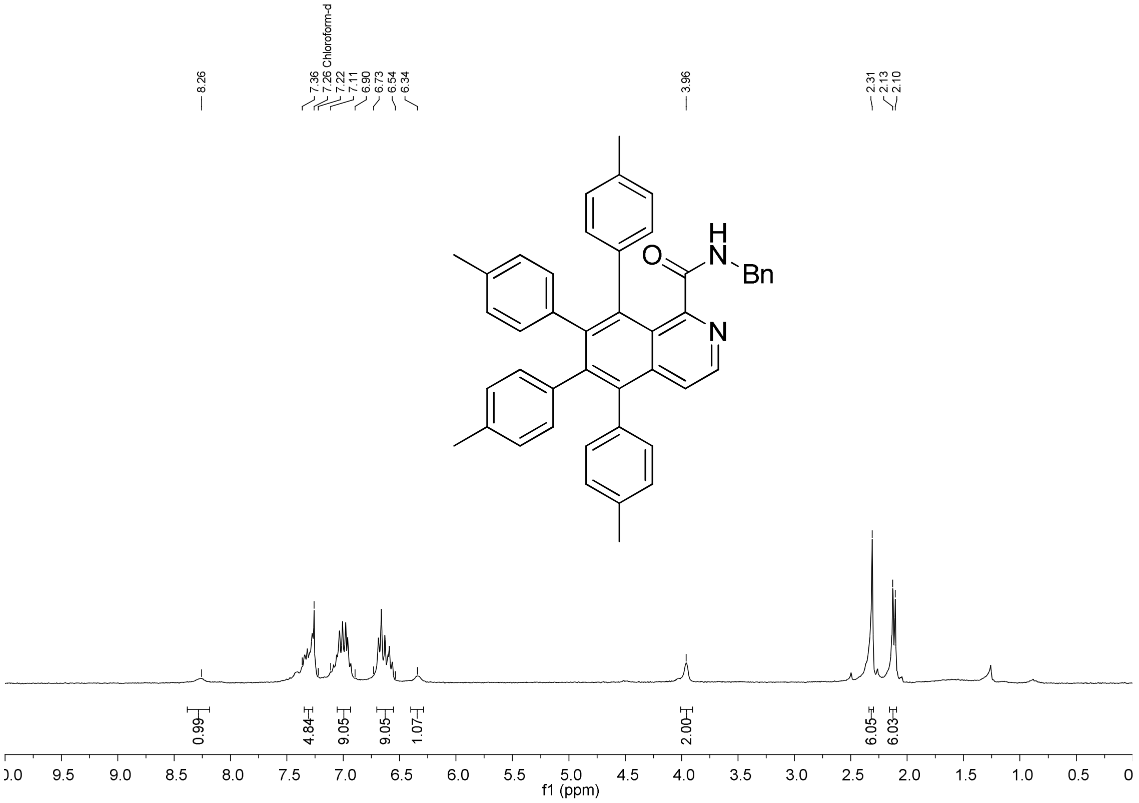

^{13}C NMR ($CDCl_3$, 75 MHz)

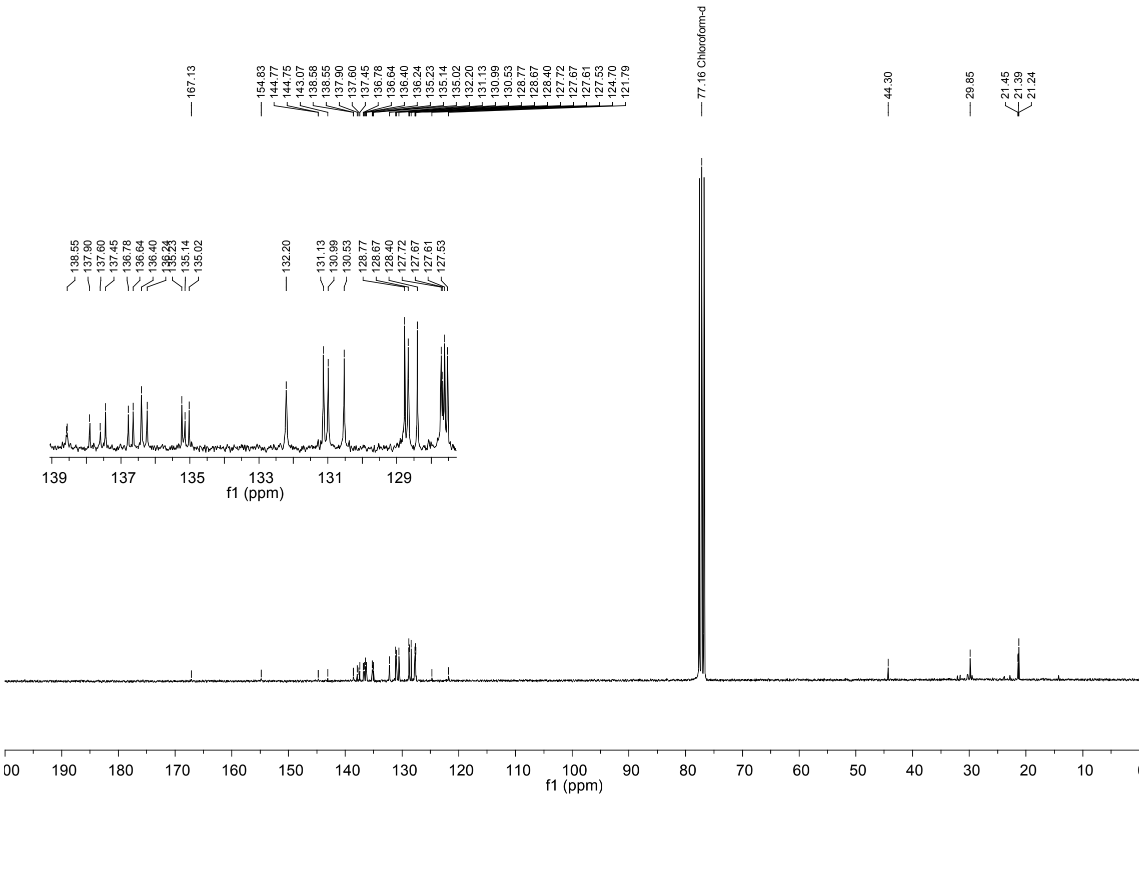

N-Benzyl-5,6,7,8-tetrakis(4-(trifluoromethyl)phenyl)isoquinoline-1-carboxamide (12)

^{1}H NMR ($CDCl_3$, 300 MHz)

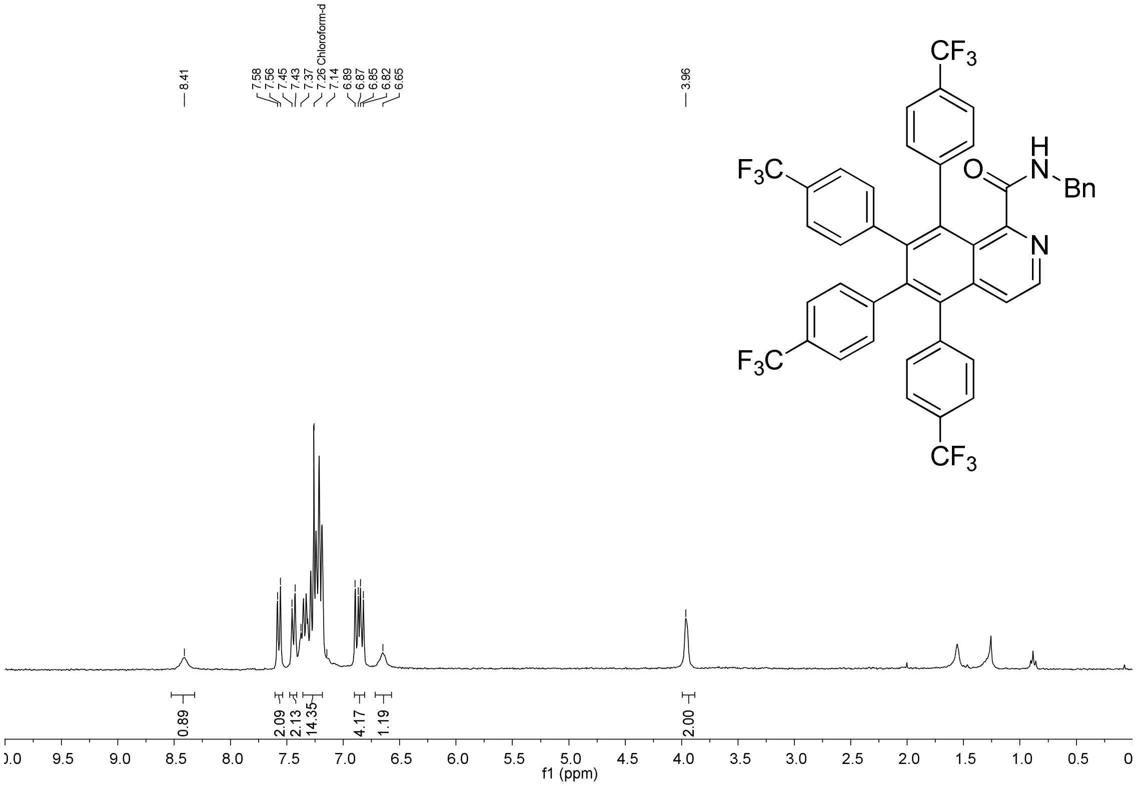

^{13}C NMR ($CDCl_3$, 125 MHz)

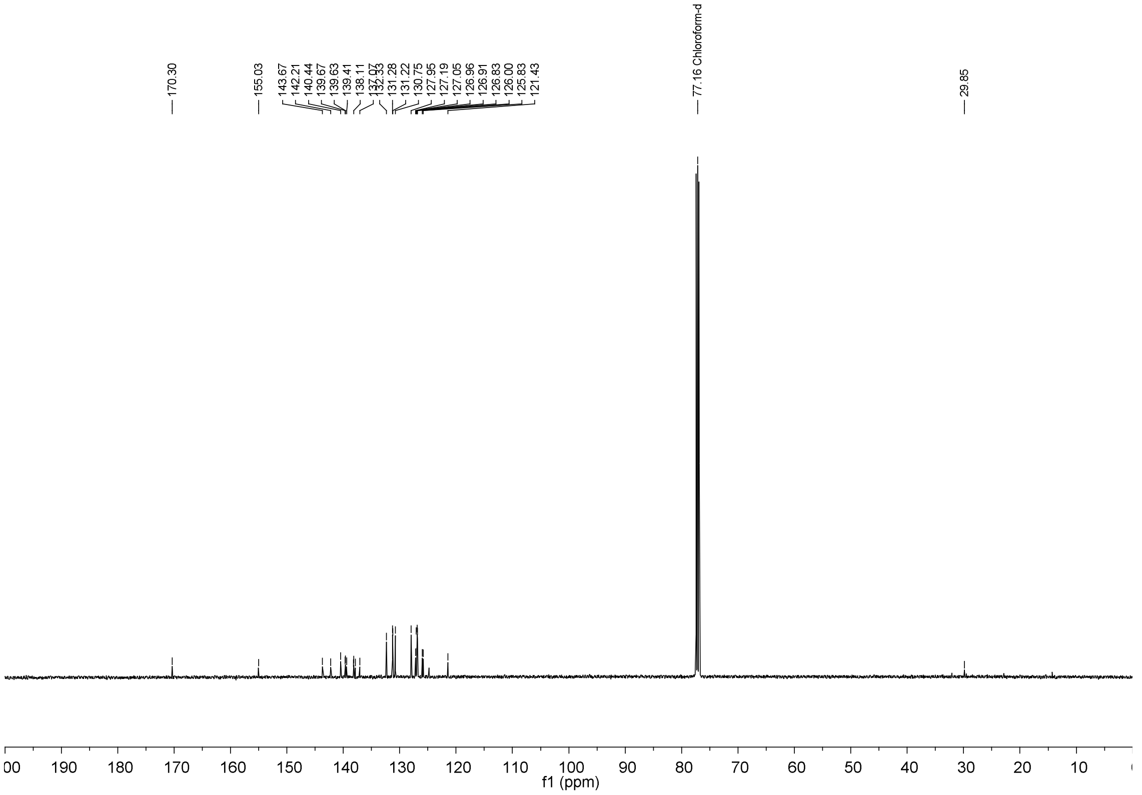

N-(2,6-Bis((*E*)-1,2-diphenylvinyl)benzyl)picolinamide (3)

^{1}H NMR (acetone-d$_6$, 500 MHz)

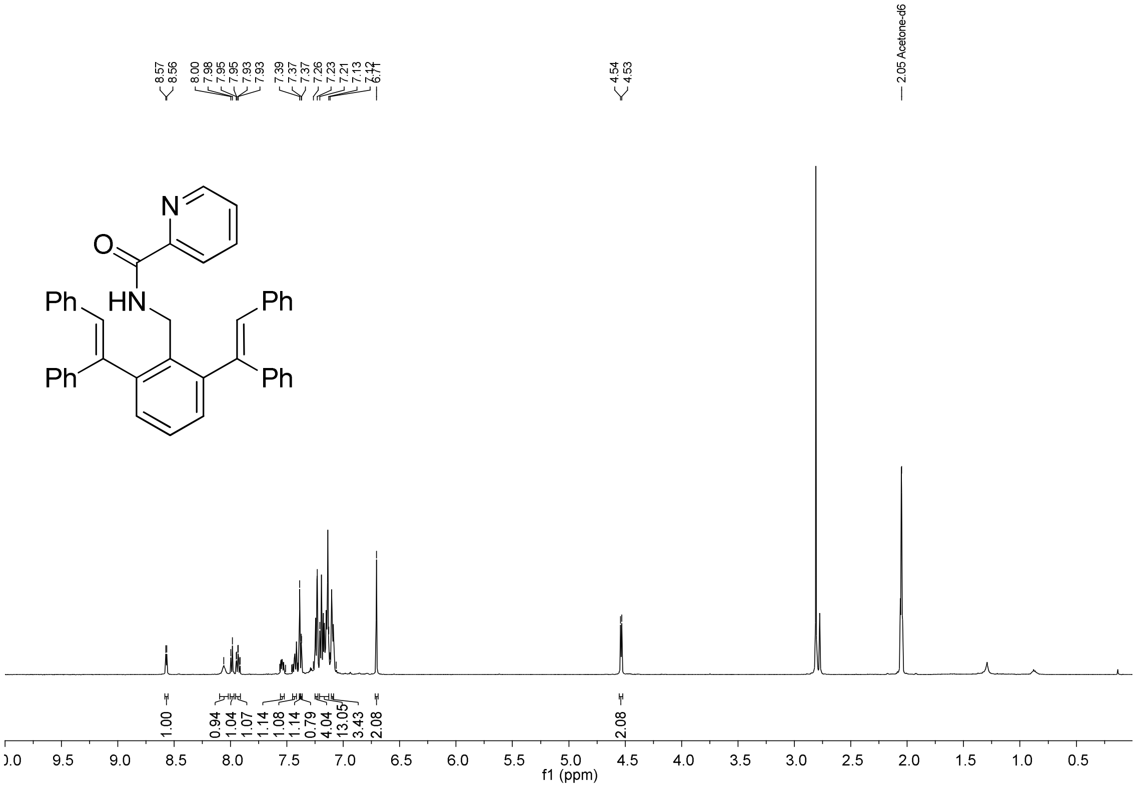

^{13}C NMR (acetone-d$_6$, 125 MHz)

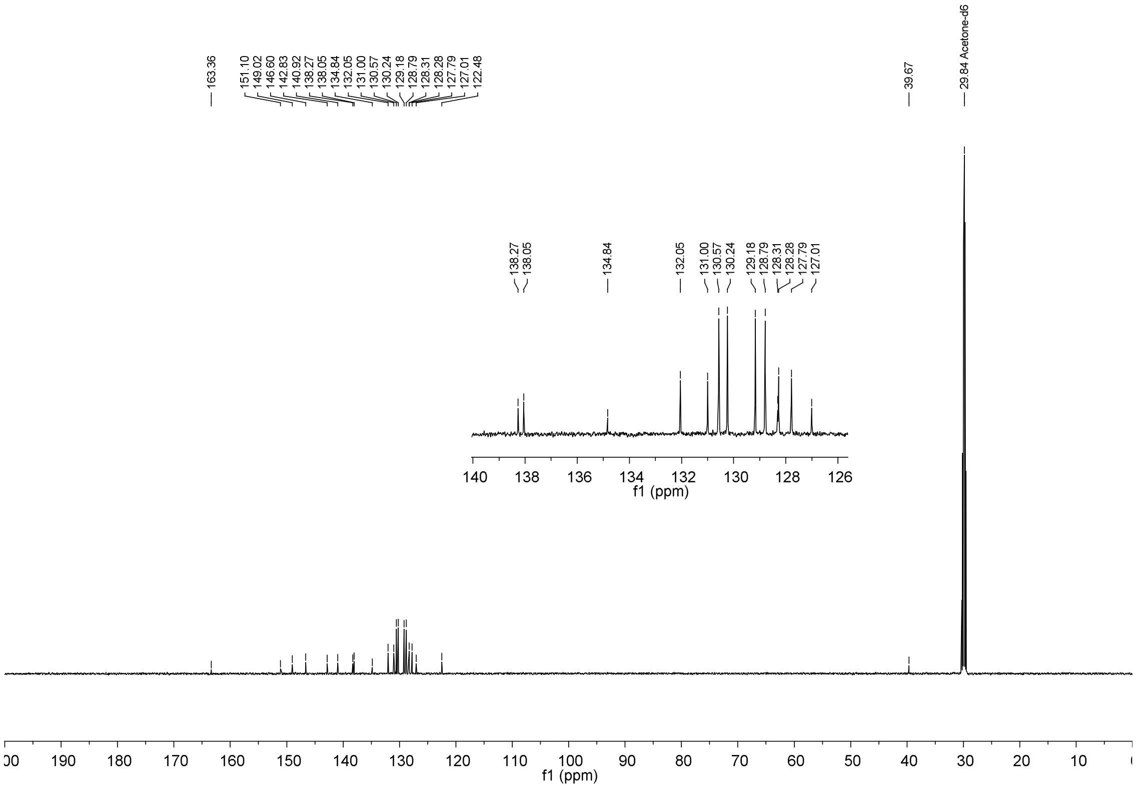

N-(2,6-Bis((E)-1,2-diphenylvinyl)-4-(methylthio)benzyl)picolinamide (39)

^{1}H NMR ($CDCl_3$, 300 MHz)

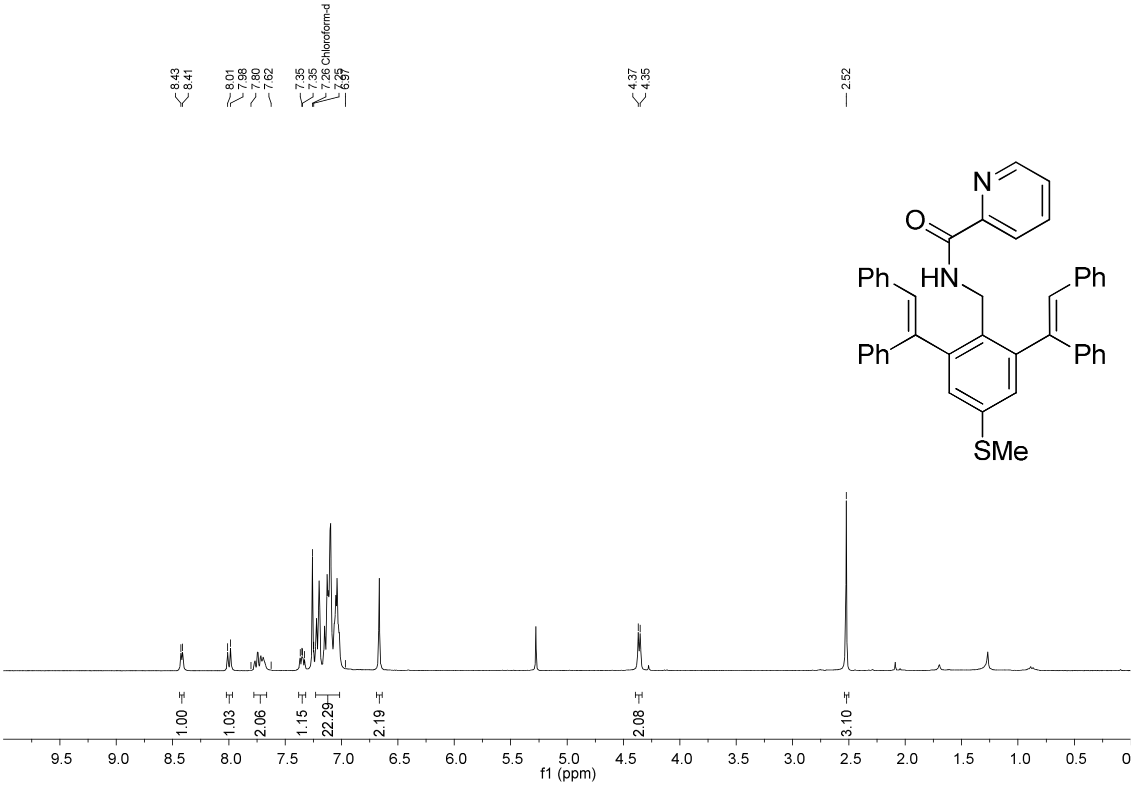

^{13}C NMR ($CDCl_3$, 75 MHz)

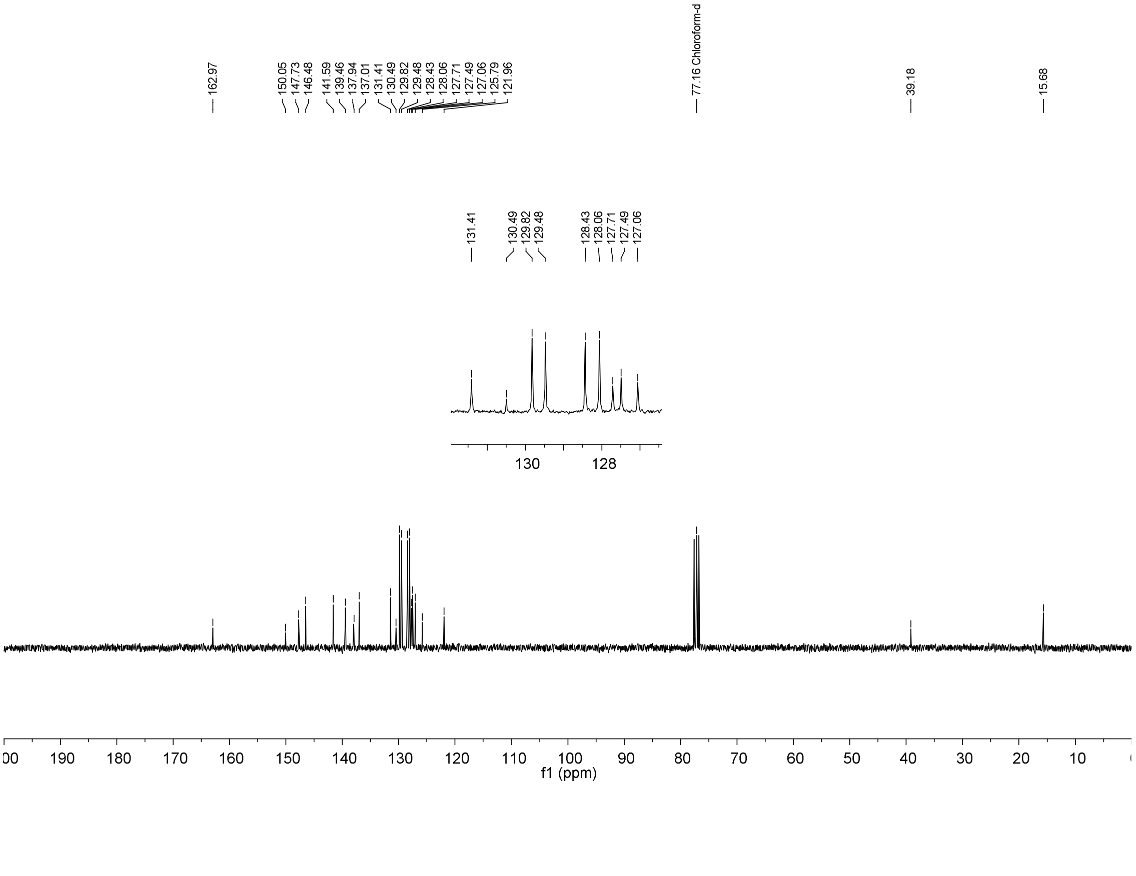

N-(2,6-Bis((E)-1,2-diphenylvinyl)-4-methoxybenzyl)picolinamide (40)

^{1}H NMR (acetone-d_6, 300 MHz)

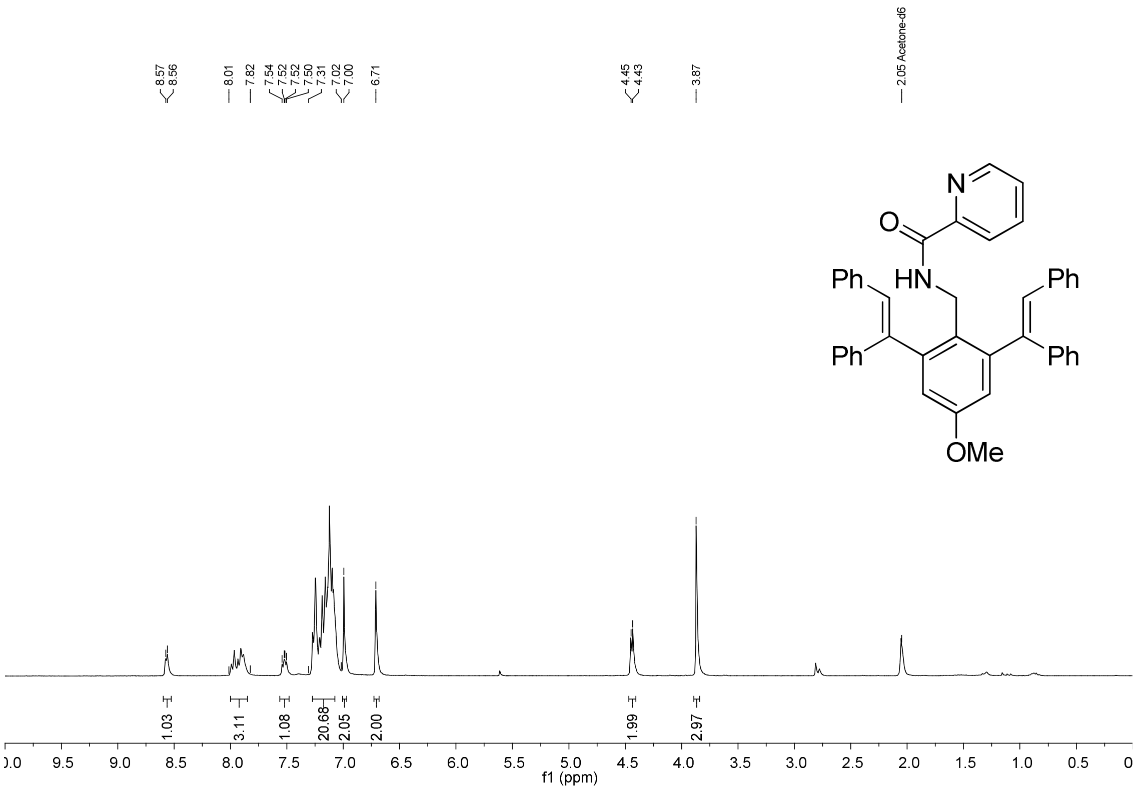

^{13}C NMR ($CDCl_3$, 75 MHz)

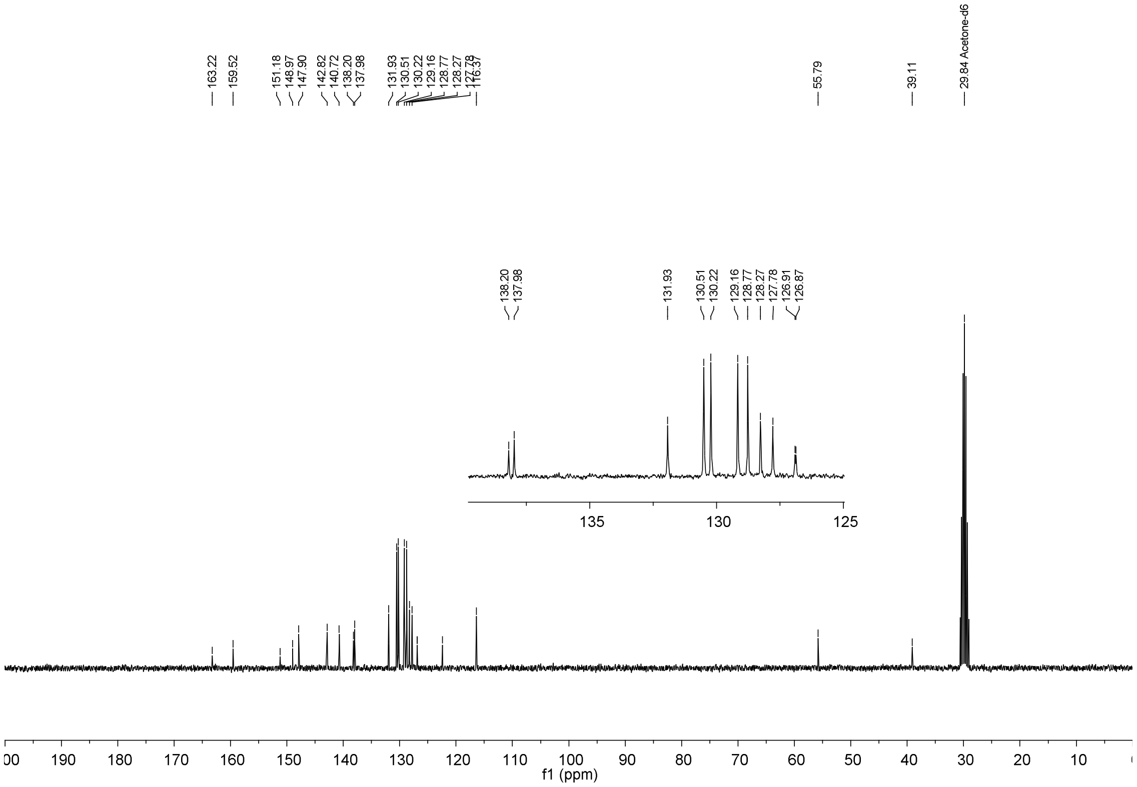

***N*-(2,6-Bis((*E*)-1,2-diphenylvinyl)-4-methylbenzyl)picolinamide (41)**

^{1}H NMR (acetone-d_6, 300 MHz)

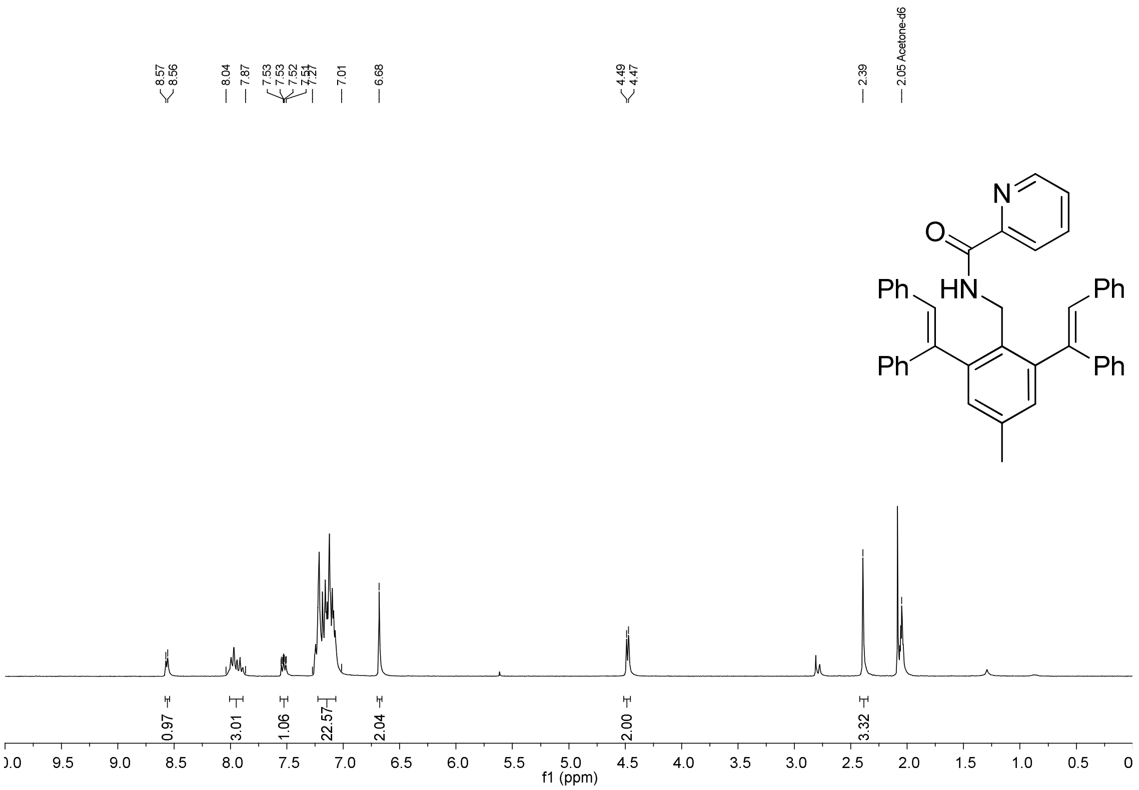

^{13}C NMR ($CDCl_3$, 75 MHz)

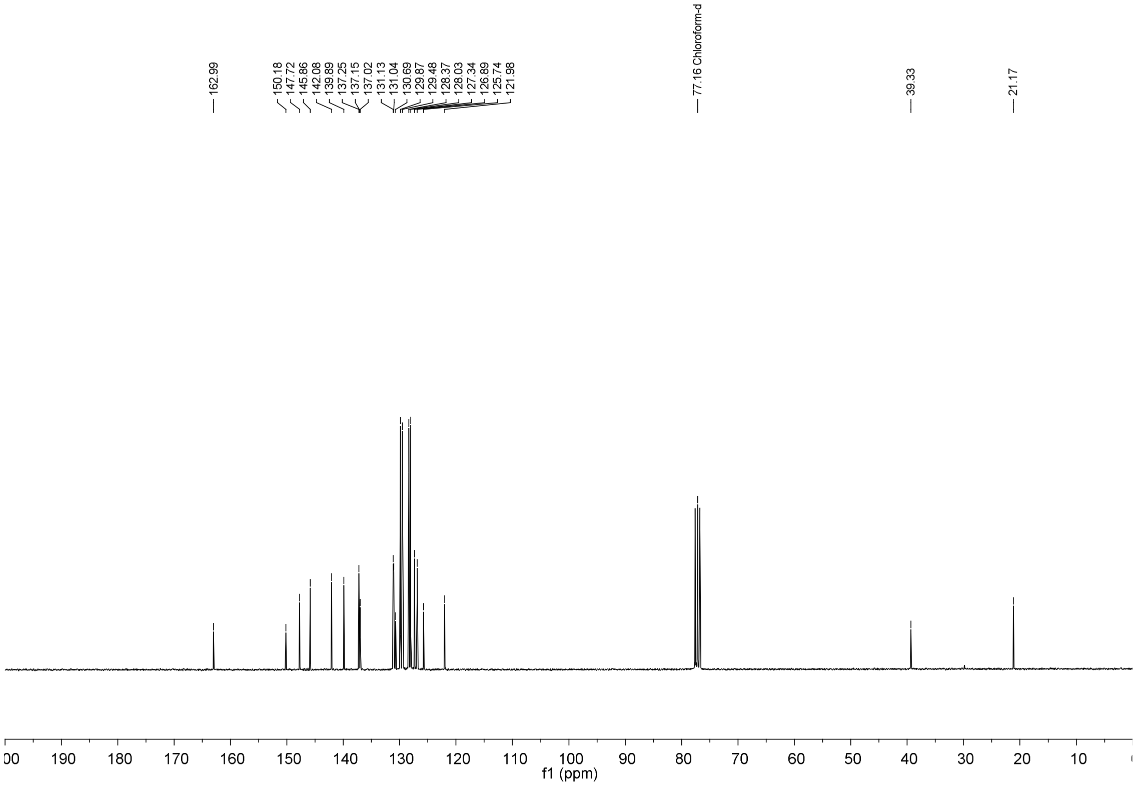

_N_-(2,6-Bis((E)-1,2-diphenylvinyl)benzyl)picolinamide (42)

^{1}H NMR (acetone-d$_6$, 300 MHz)

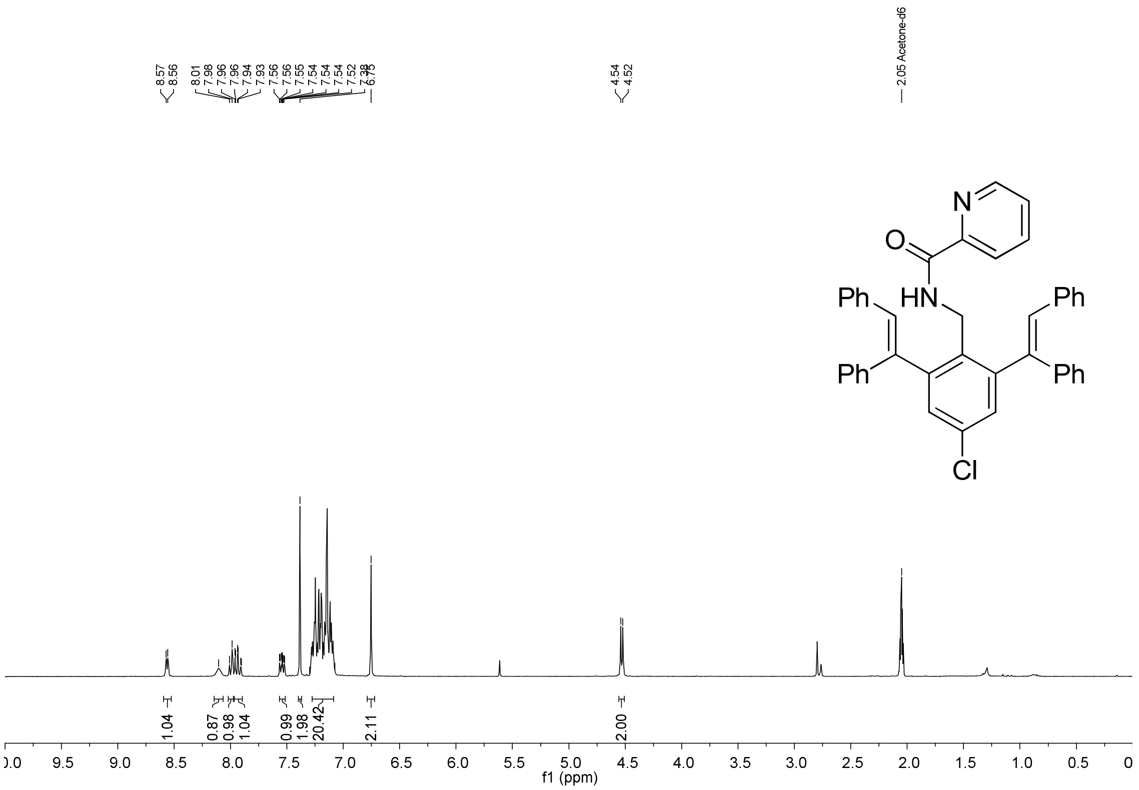

^{13}C NMR (acetone-d$_6$, 75 MHz)

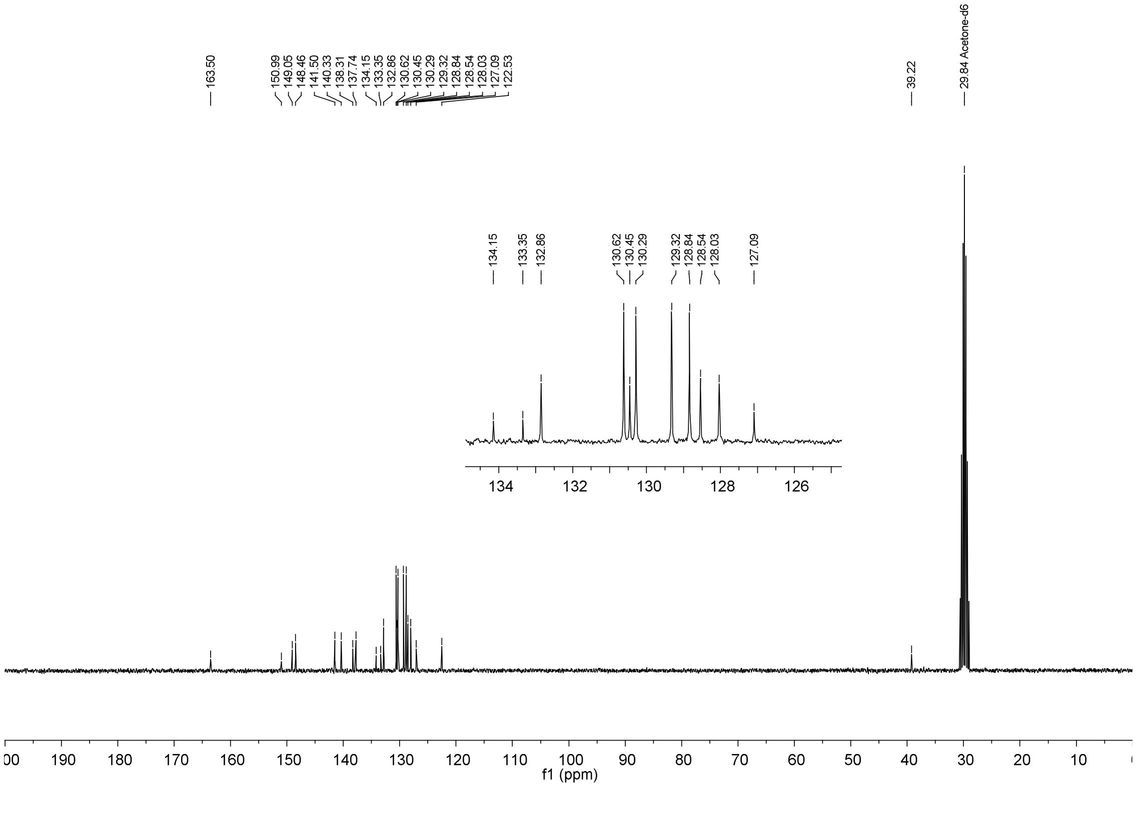

N-(2,6-Bis((*E*)-1,2-diphenylvinyl)-4-fluorobenzyl)picolinamide (43)

^{1}H NMR (CDCl$_3$, 300 MHz)

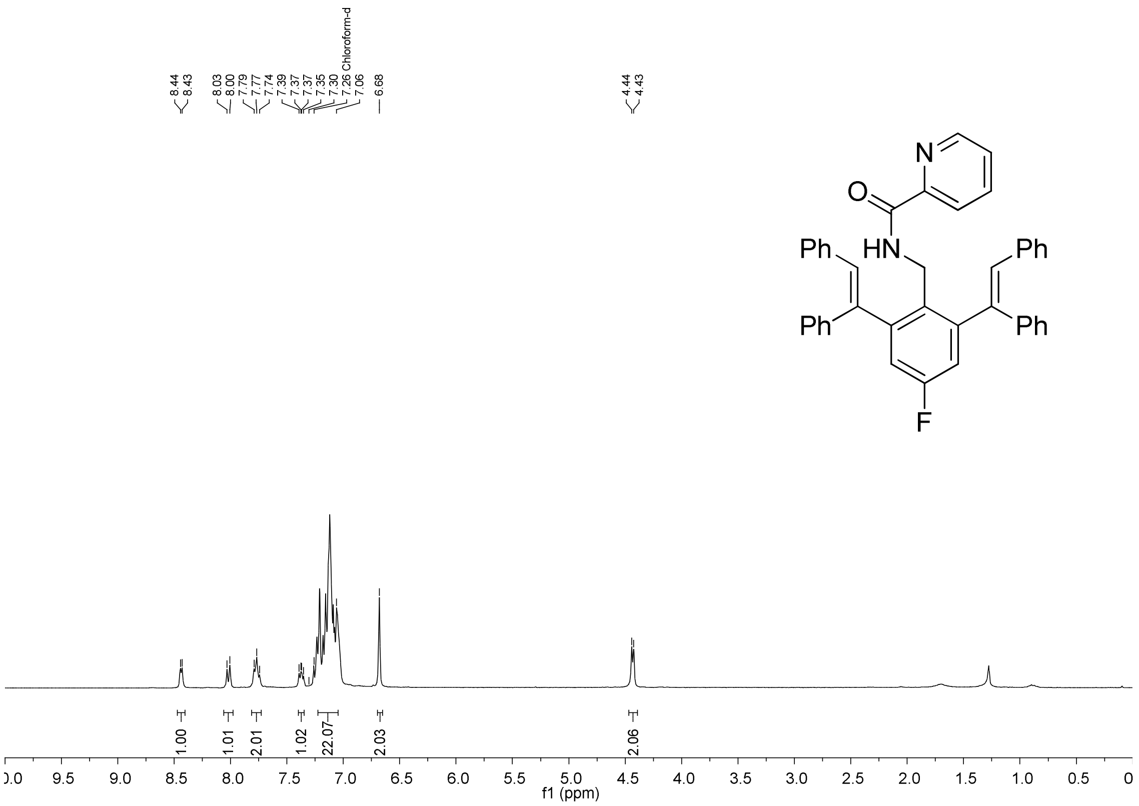

^{13}C NMR (CDCl$_3$, 75 MHz)

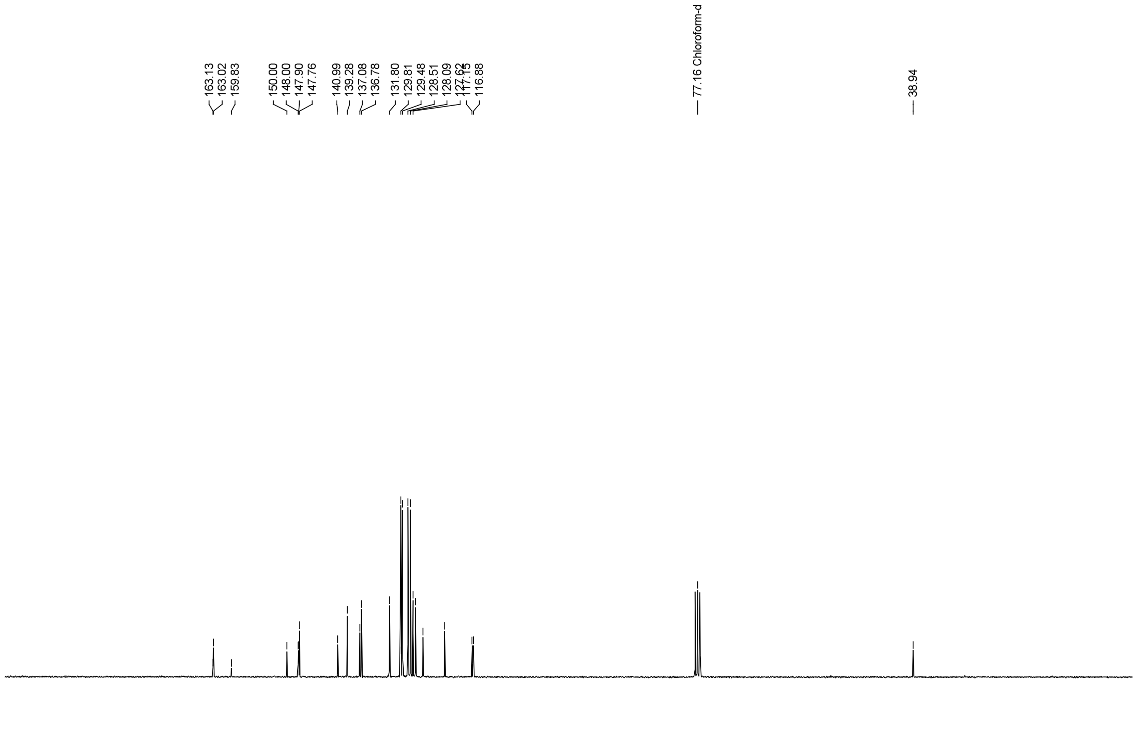

***N*-(2,6-Bis((*E*)-1,2-diphenylvinyl)-4-(trifluoromethyl)benzyl)picolinamide (44)**

^{1}H NMR (acetone-d$_6$, 300 MHz)

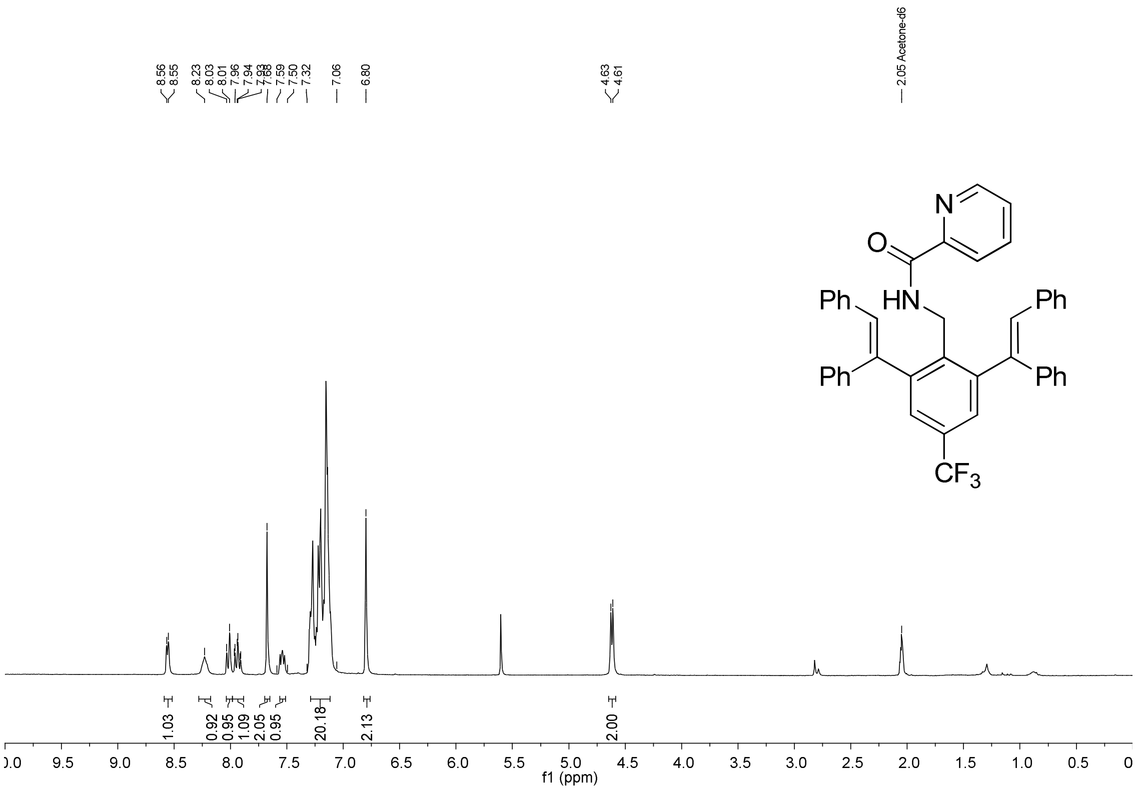

^{13}C NMR (acetone-d$_6$, 75 MHz)

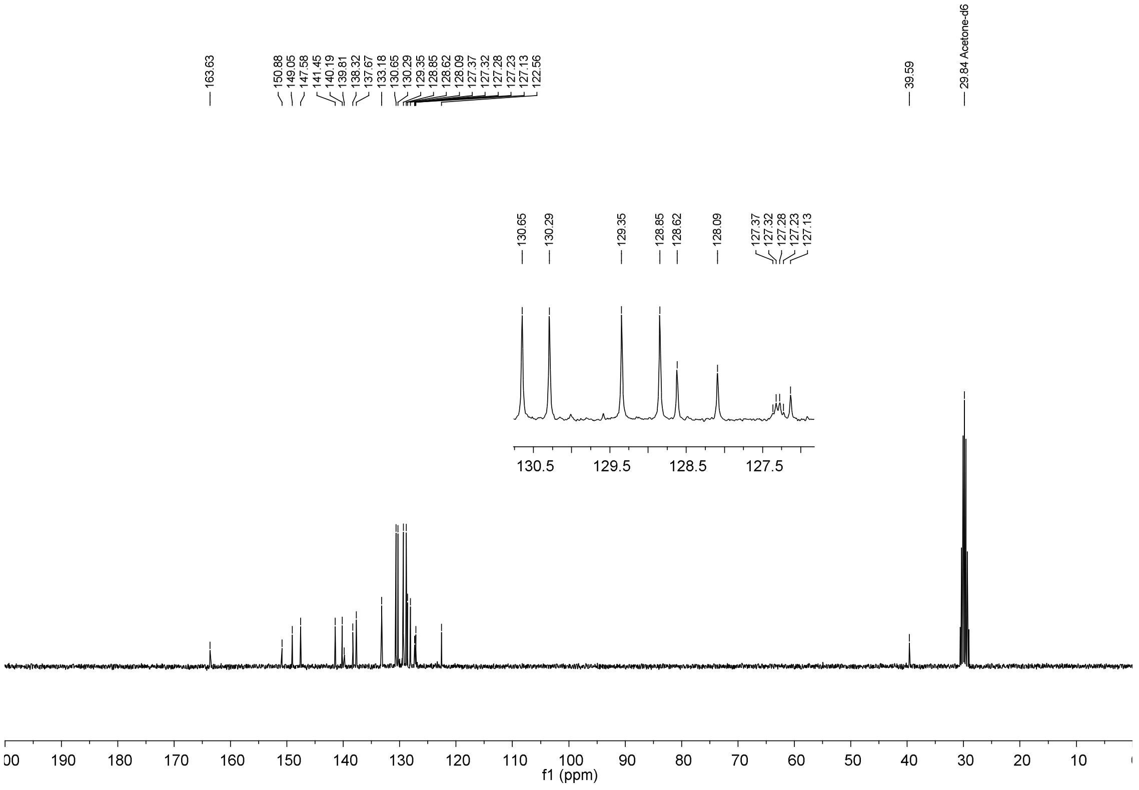

N-(4-Cyano-2,6-bis((E)-1,2-diphenylvinyl)benzyl)picolinamide (45)

^{1}H NMR ($CDCl_3$, 300 MHz)

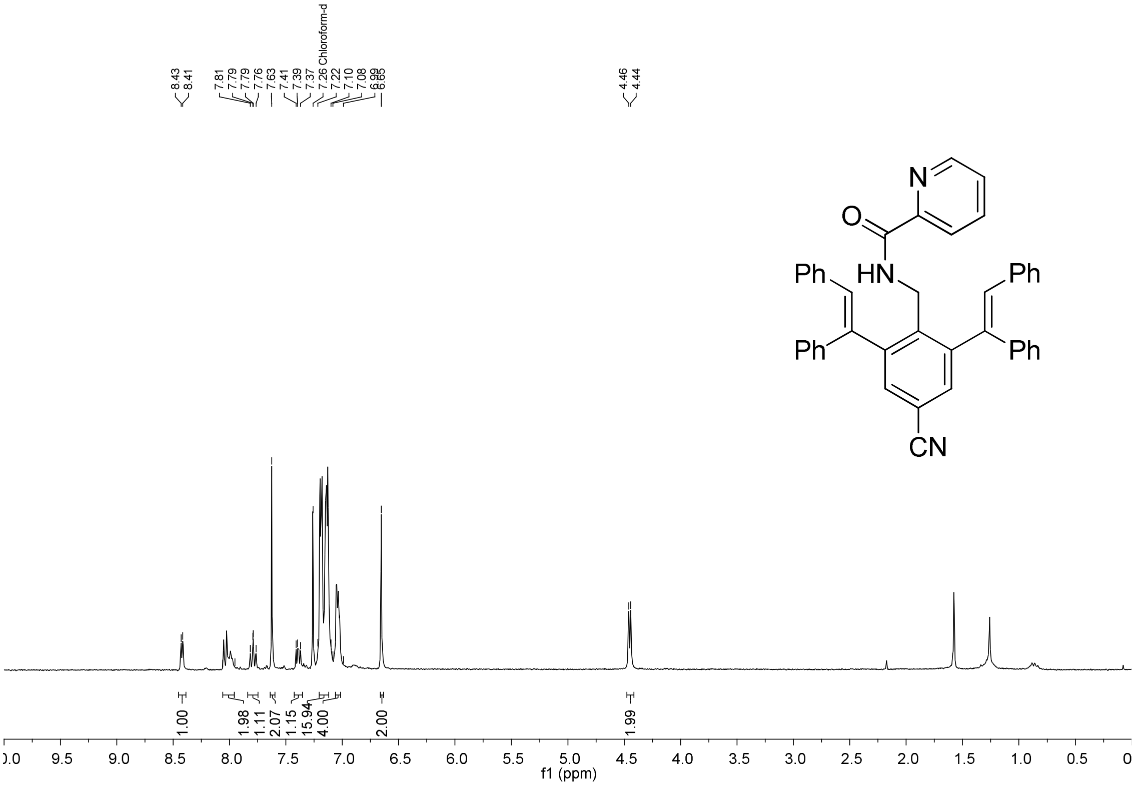

^{13}C NMR ($CDCl_3$, 75 MHz)

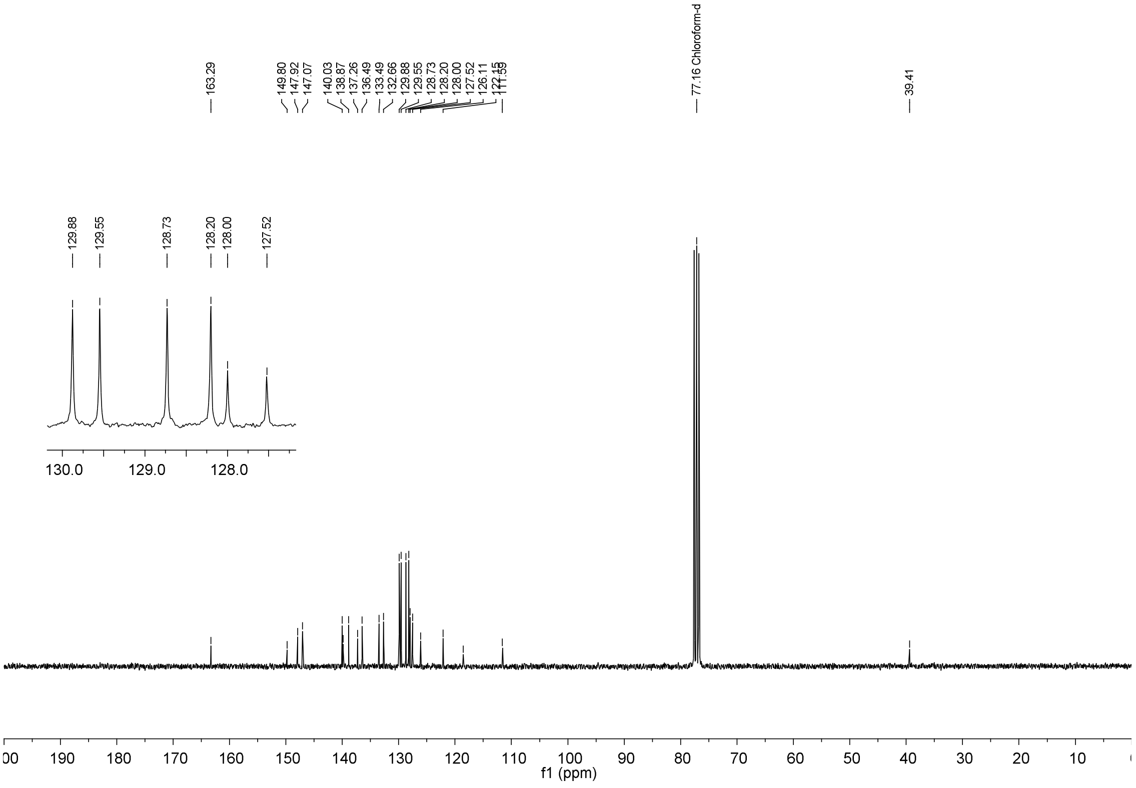

Methyl 3,5-bis((*E*)-1,2-diphenylvinyl)-4-(picolinamidomethyl)benzoate (46)

^{1}H NMR ($CDCl_3$, 300 MHz)

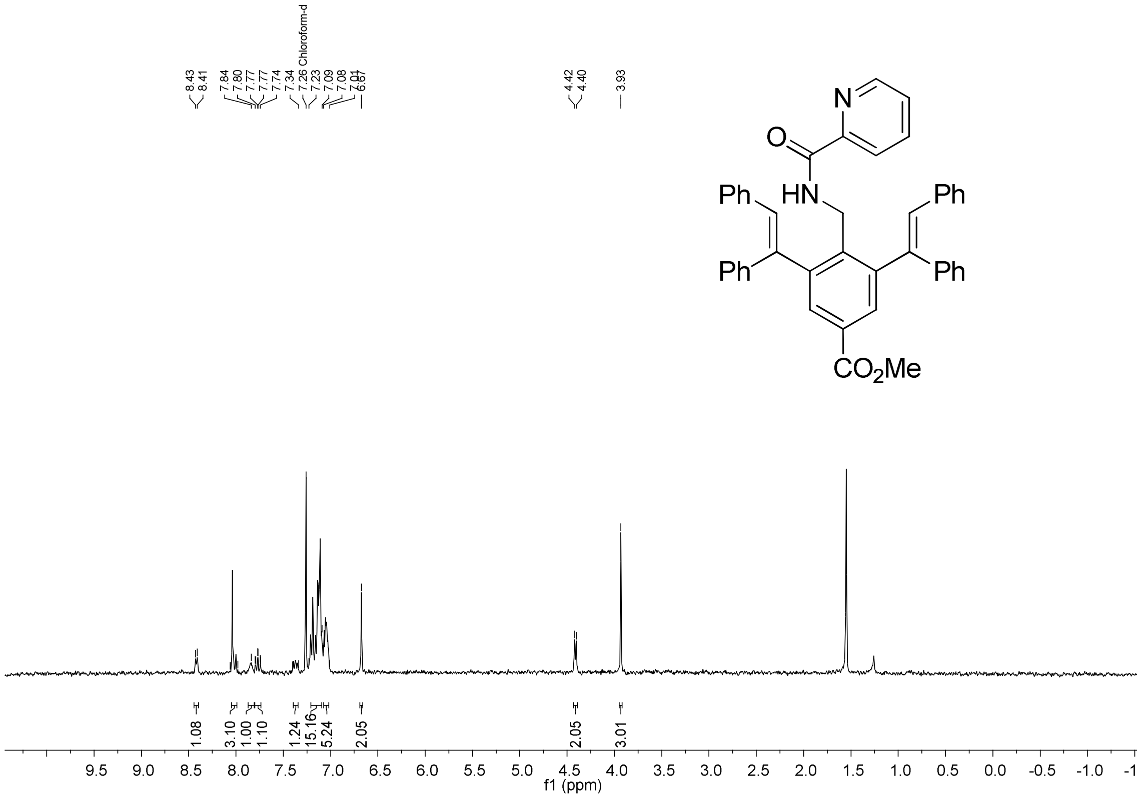

^{13}C NMR ($CDCl_3$, 75 MHz)

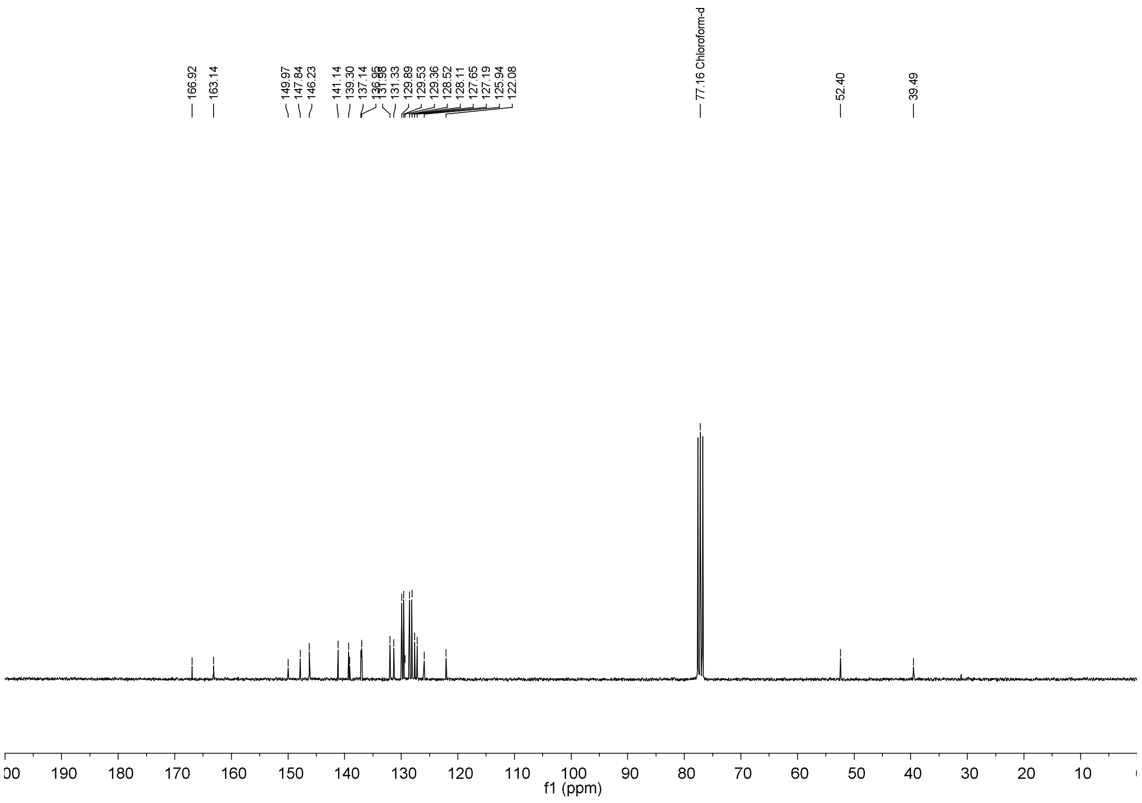

(*E*)-*N*-(2-(1,2-Diphenylvinyl)-5-methylbenzyl)picolinamide (47a)

^{1}H NMR ($CDCl_3$, 300 MHz)

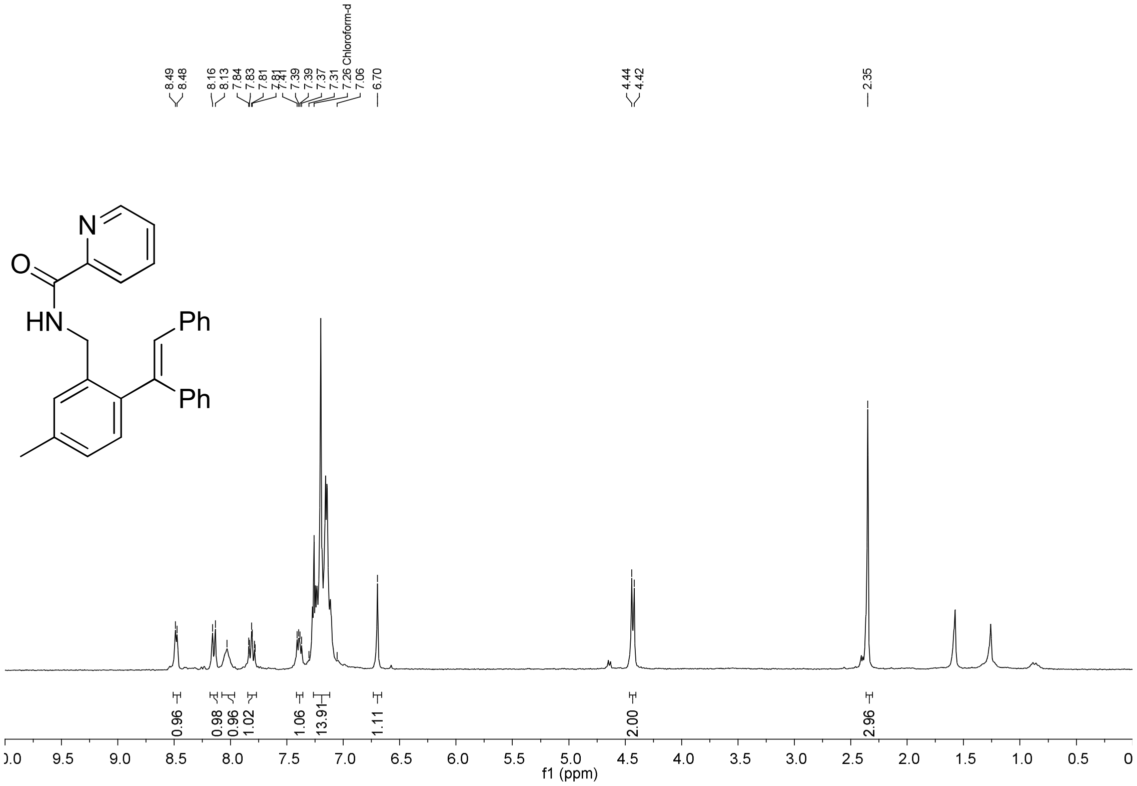

_N_-(2,6-Bis((_E_)-1,2-diphenylvinyl)-3-methylbenzyl)picolinamide (47b)

^{1}H NMR ($CDCl_3$, 300 MHz)

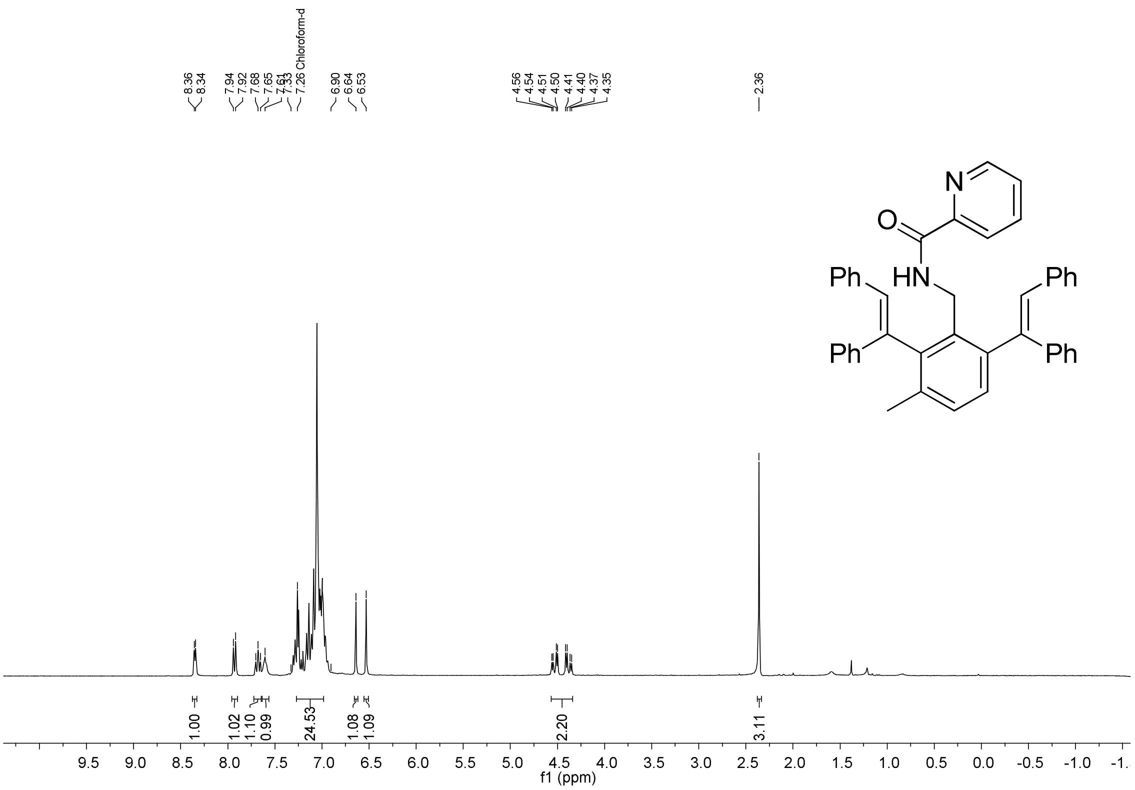

^{13}C NMR ($CDCl_3$, 75 MHz)

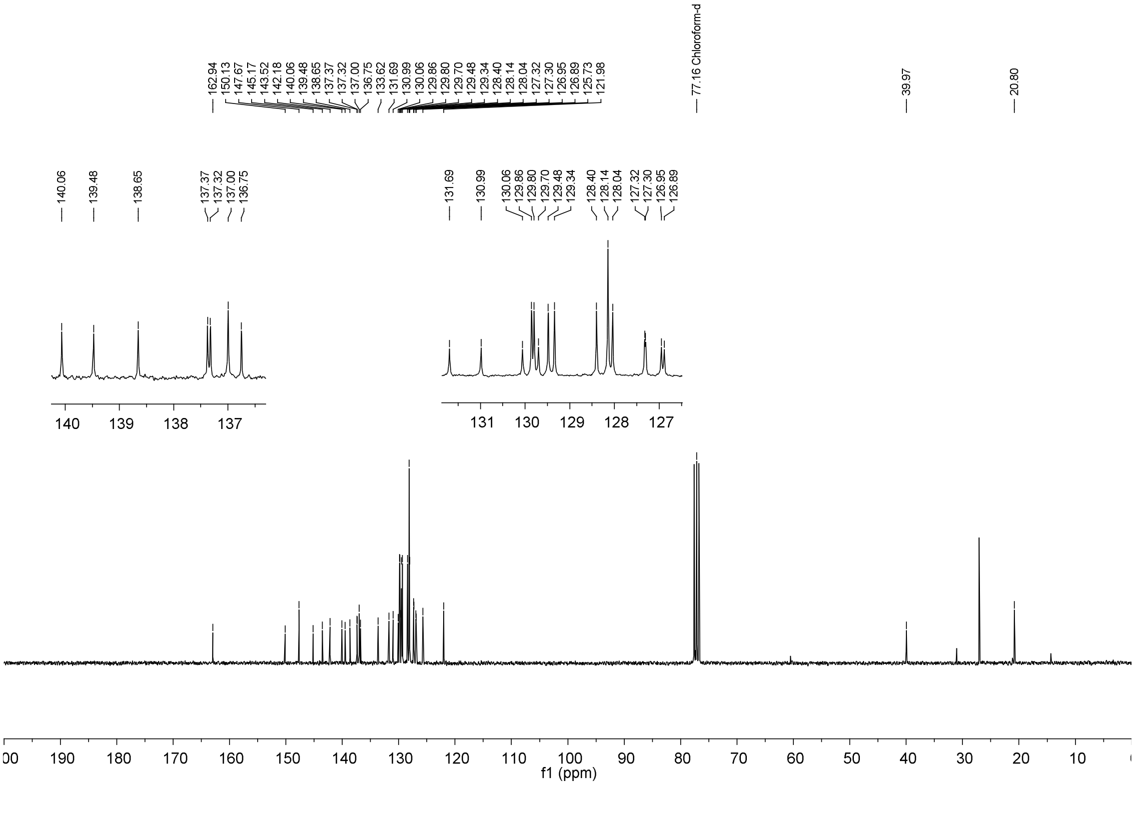

(*E*)-*N*-(2-(1,2-Diphenylvinyl)-5-(trifluoromethyl)benzyl)picolinamide (48)

^{1}H NMR ($CDCl_3$, 300 MHz)

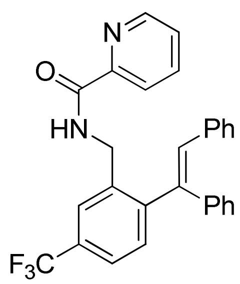

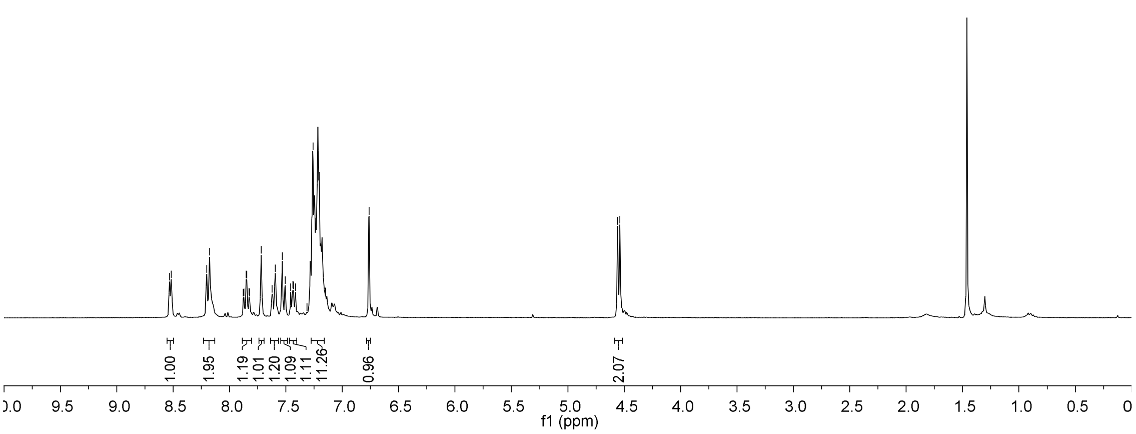

^{13}C NMR ($CDCl_3$, 75 MHz)

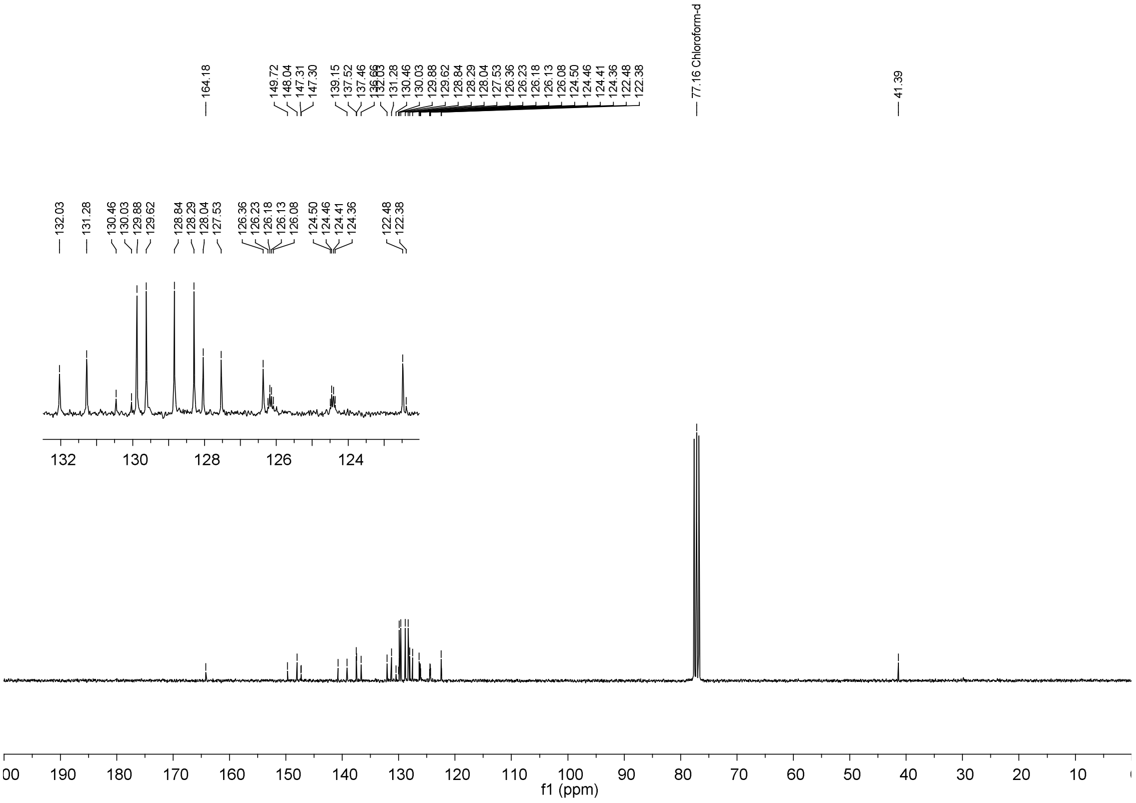

(*E*)-*N*-(2-(1,2-Diphenylvinyl)-6-methylbenzyl)picolinamide (49)

^{1}H NMR (acetone-d$_6$, 300 MHz)

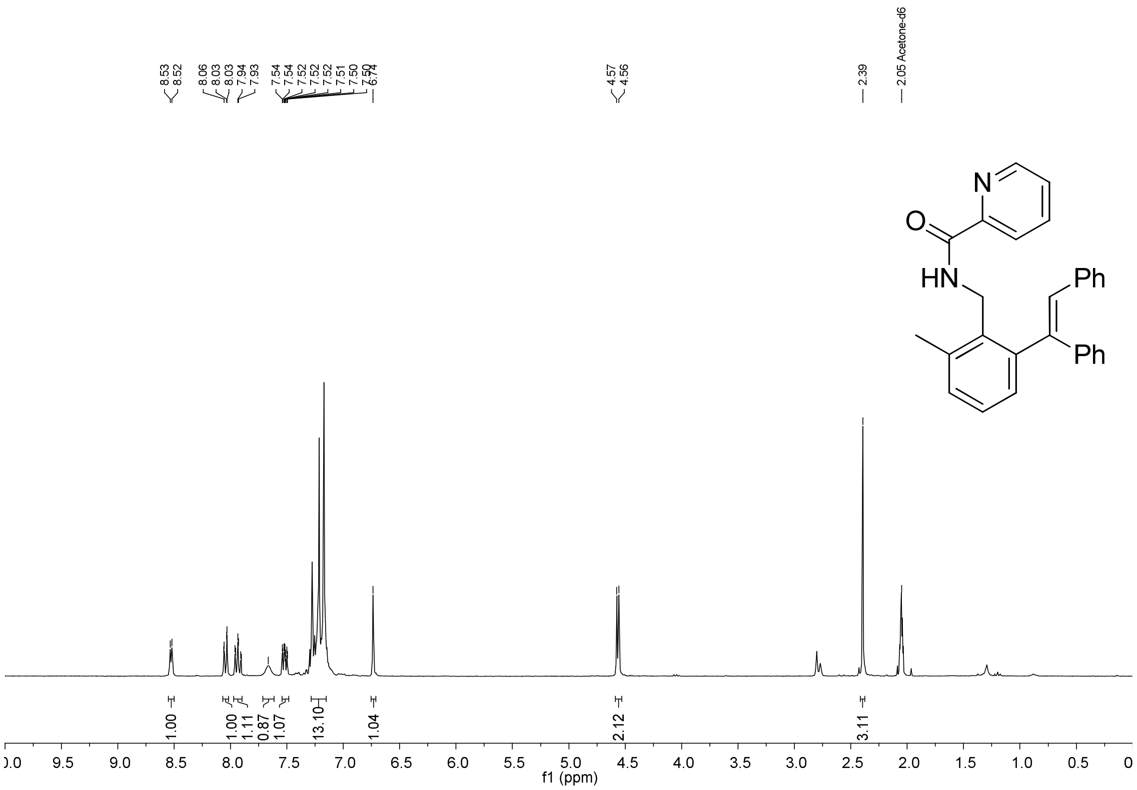

^{13}C NMR (acetone-d$_6$, 75 MHz)

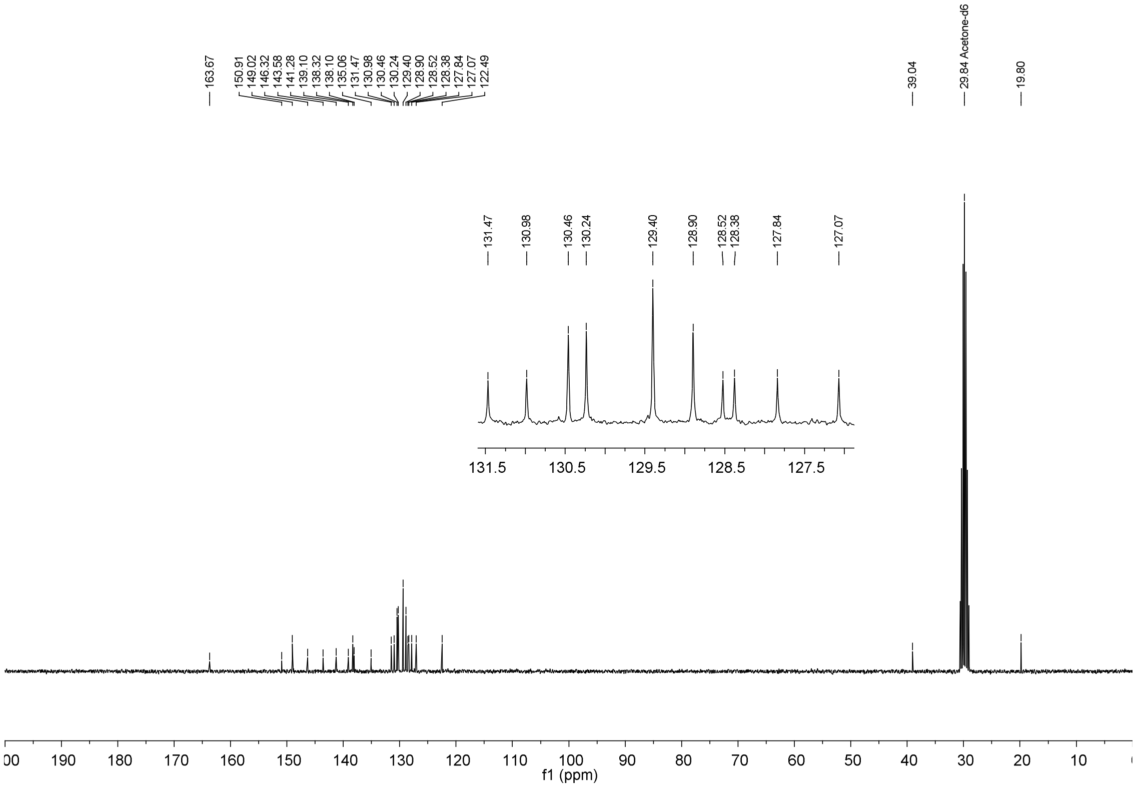

(*E*)-*N*-(2-Bromo-6-(1,2-diphenylvinyl)benzyl)picolinamide (50)

^{1}H NMR (acetone-d$_6$, 300 MHz)

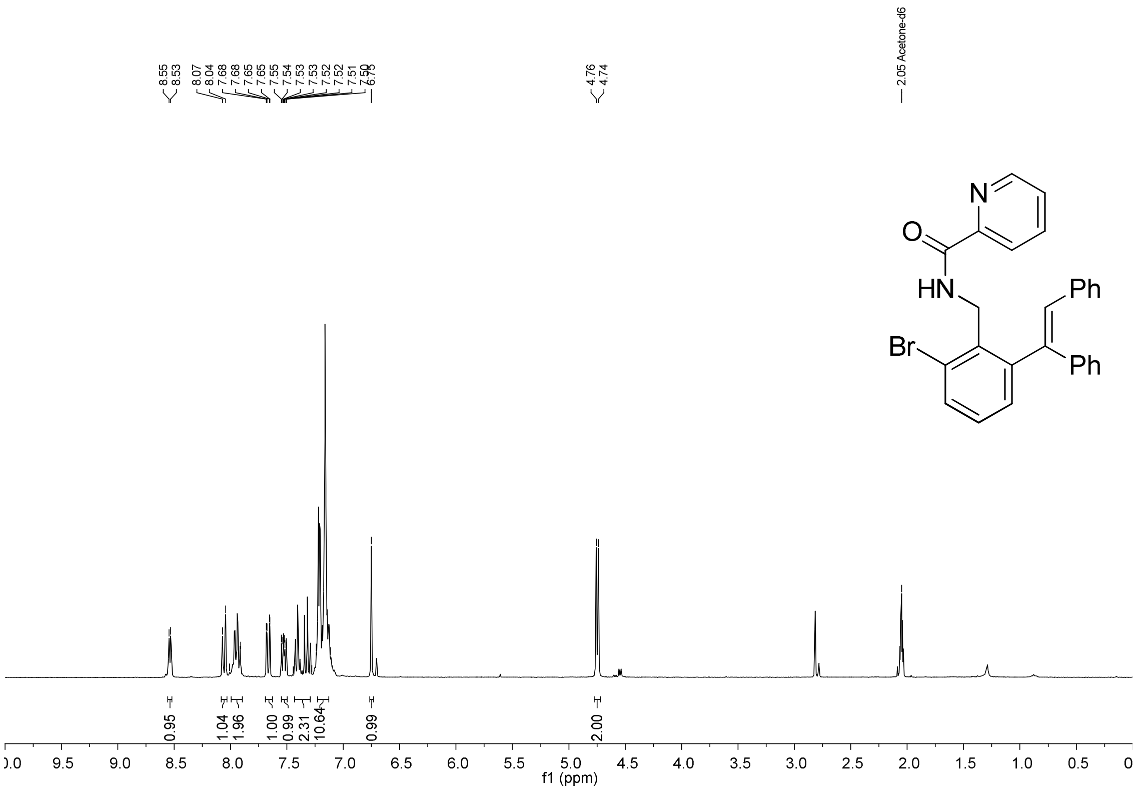

^{13}C NMR (acetone-d$_6$, 75 MHz)

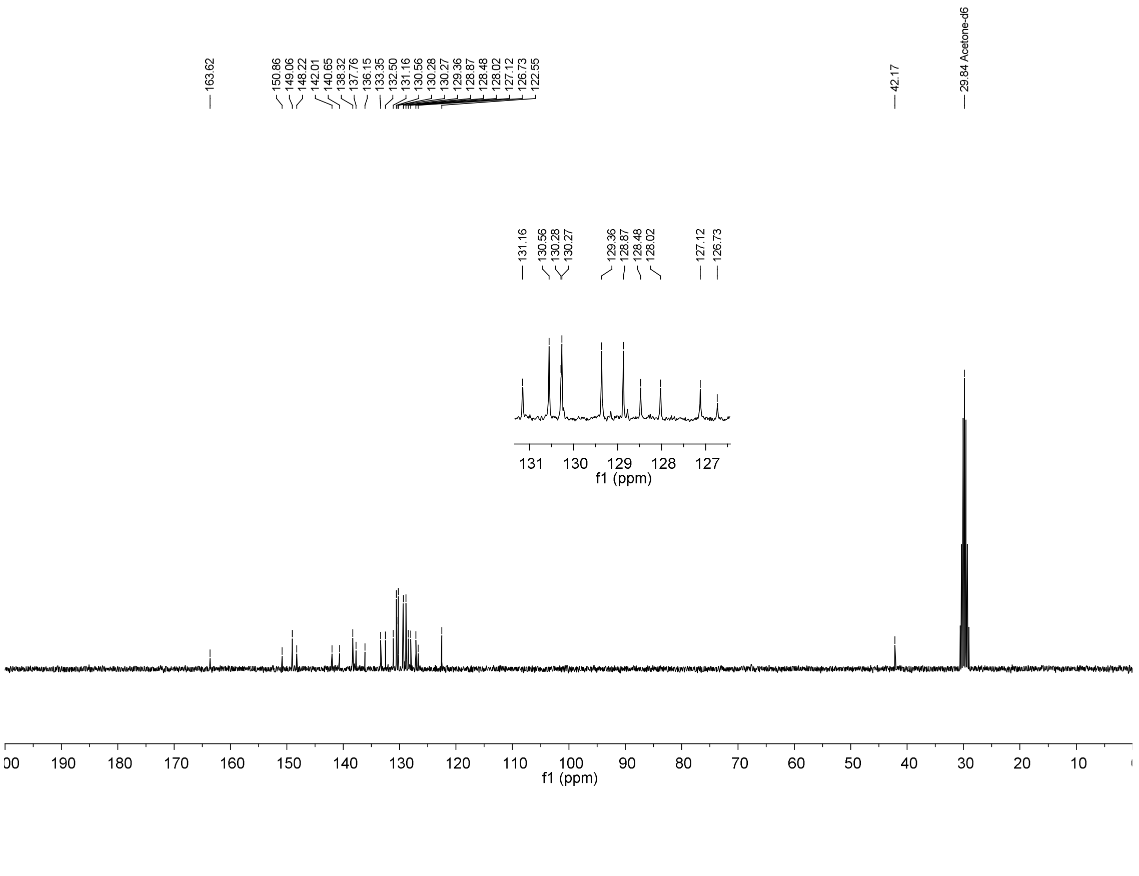

(*E*)-*N*-(2-(1,2-Diphenylvinyl)-6-fluorobenzyl)picolinamide (51)

^{1}H NMR ($CDCl_3$, 300 MHz)

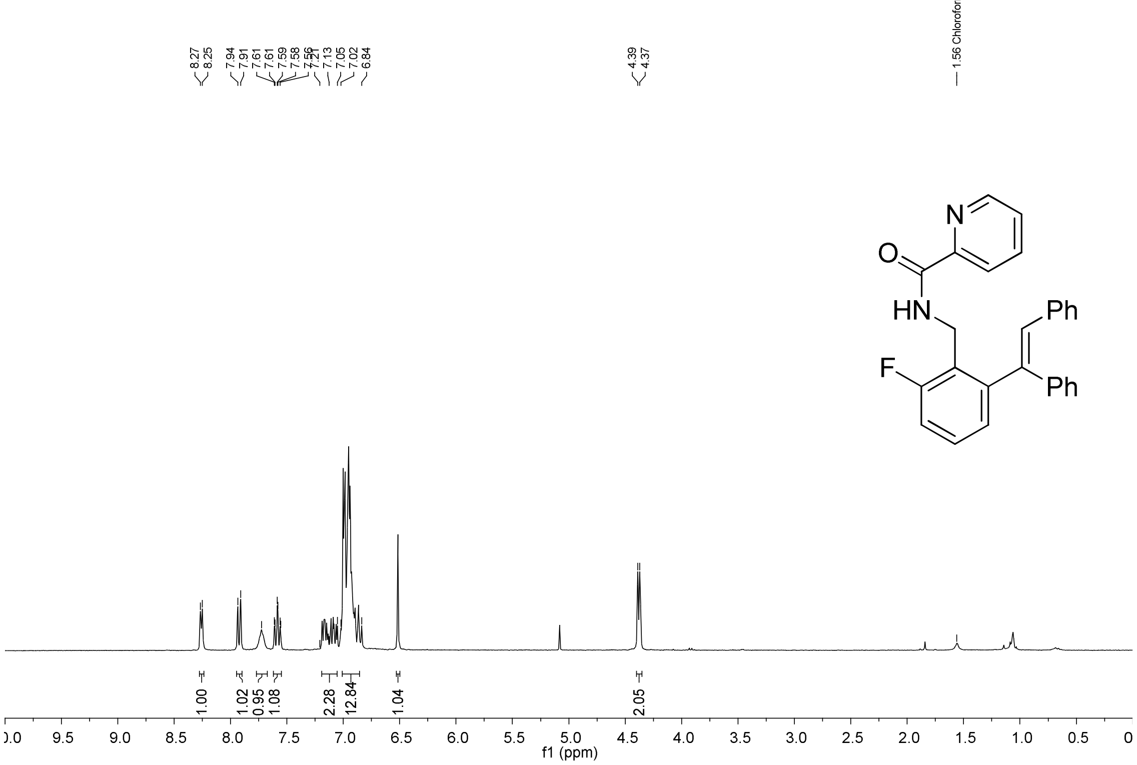

^{13}C NMR ($CDCl_3$, 75 MHz)

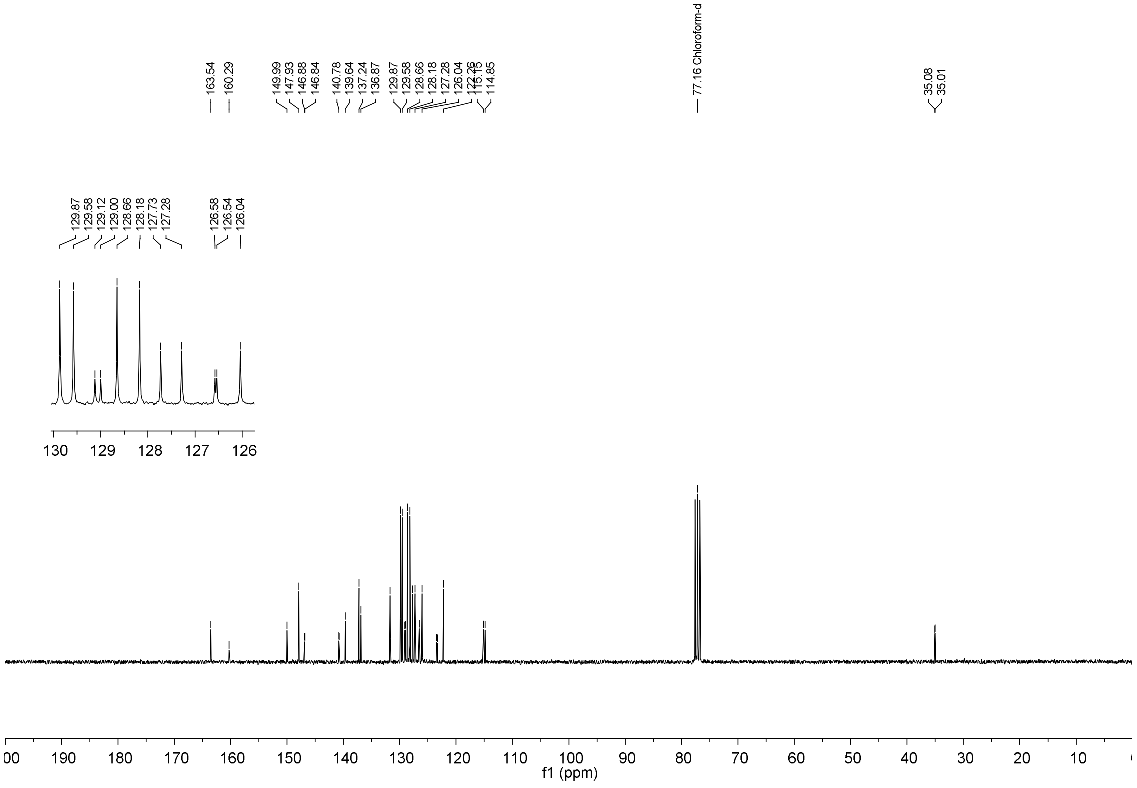

^{19}F NMR ($CDCl_3$, 282 MHz)

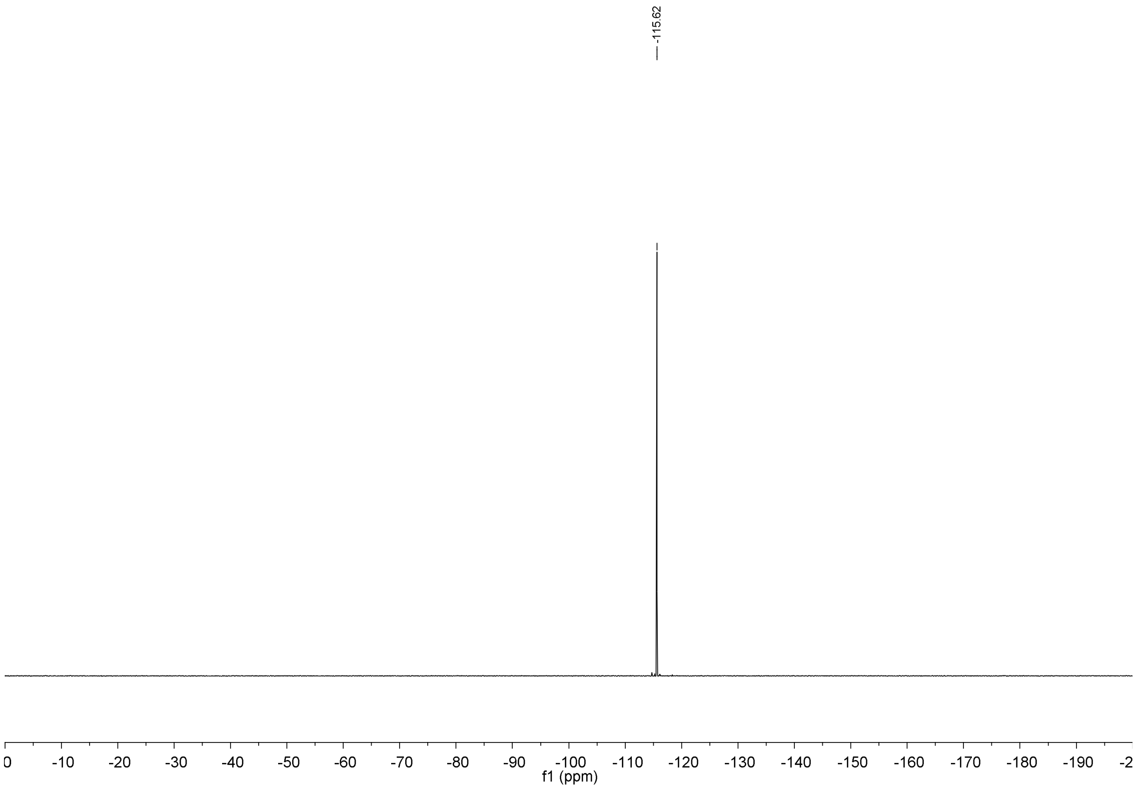

(*E*)-*N*-((3-(1,2-Diphenylvinyl)furan-2-yl)methyl)picolinamide (52)

^{1}H NMR ($CDCl_3$, 300 MHz)

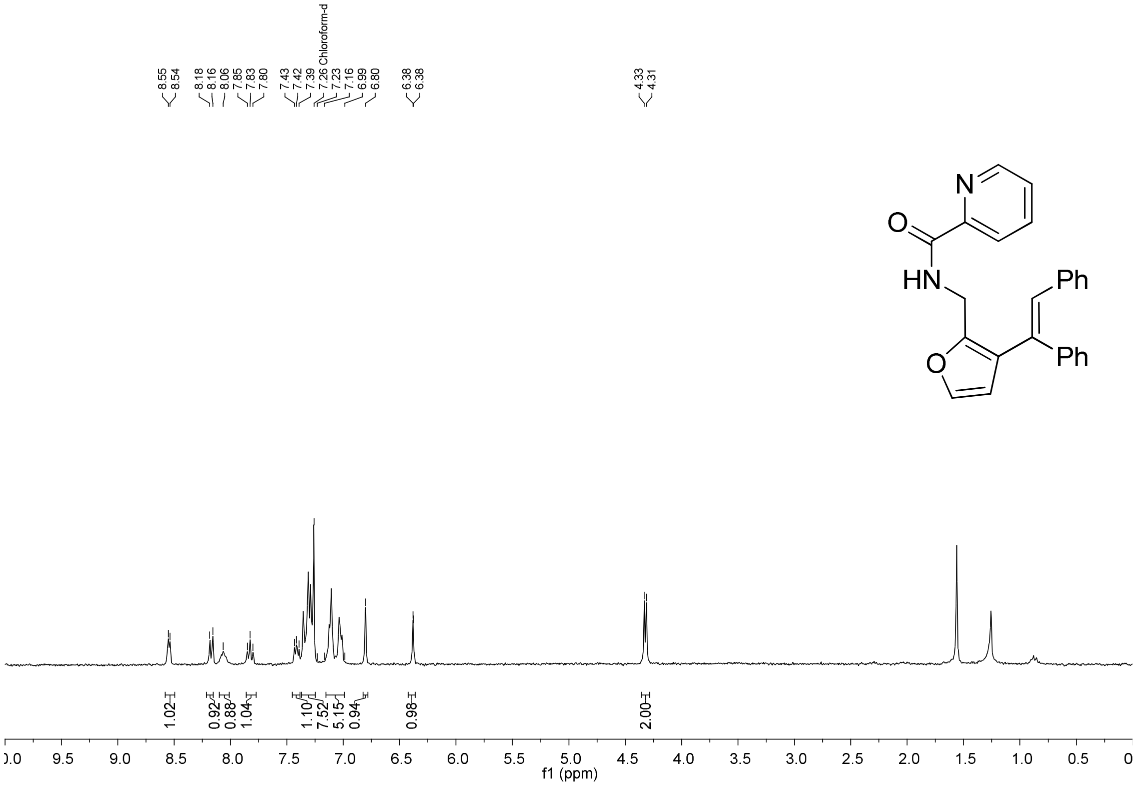

^{13}C NMR ($CDCl_3$, 75 MHz)

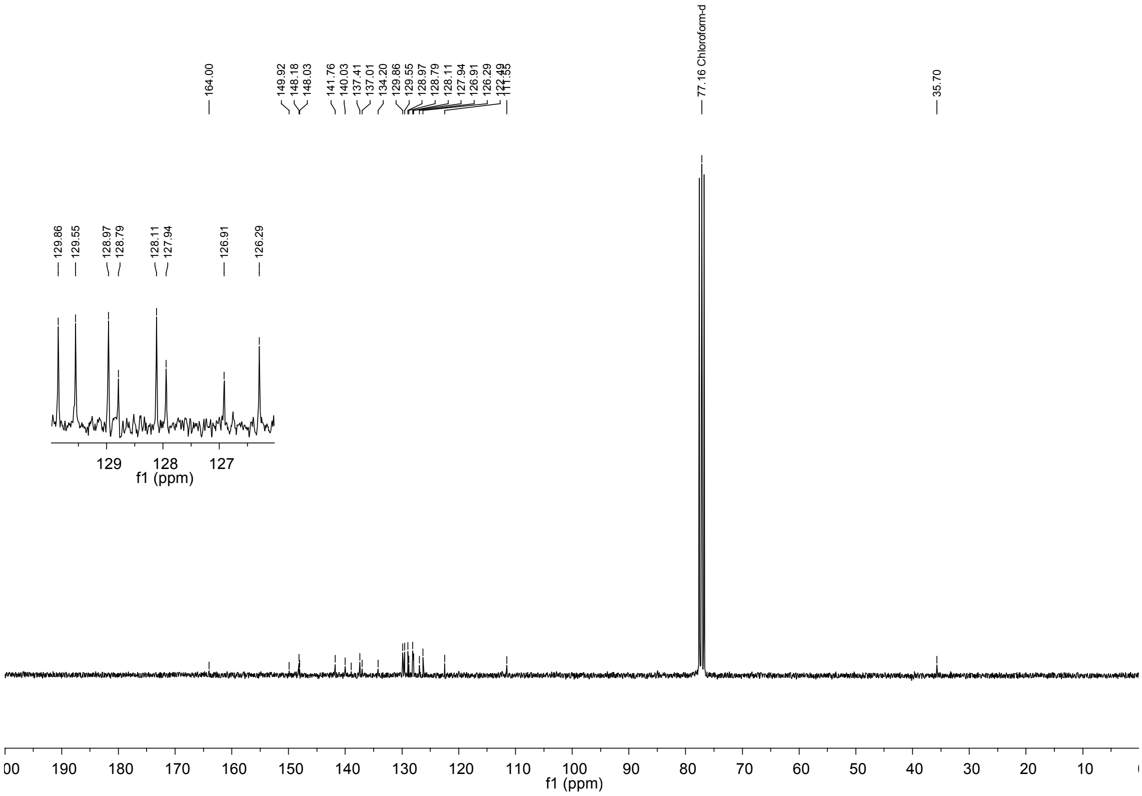

***N*-(2,6-Bis((*E*)-1,2-bis(4-methoxyphenyl)vinyl)benzyl)picolinamide (34)**

^{1}H NMR (acetone-d$_6$, 300 MHz)

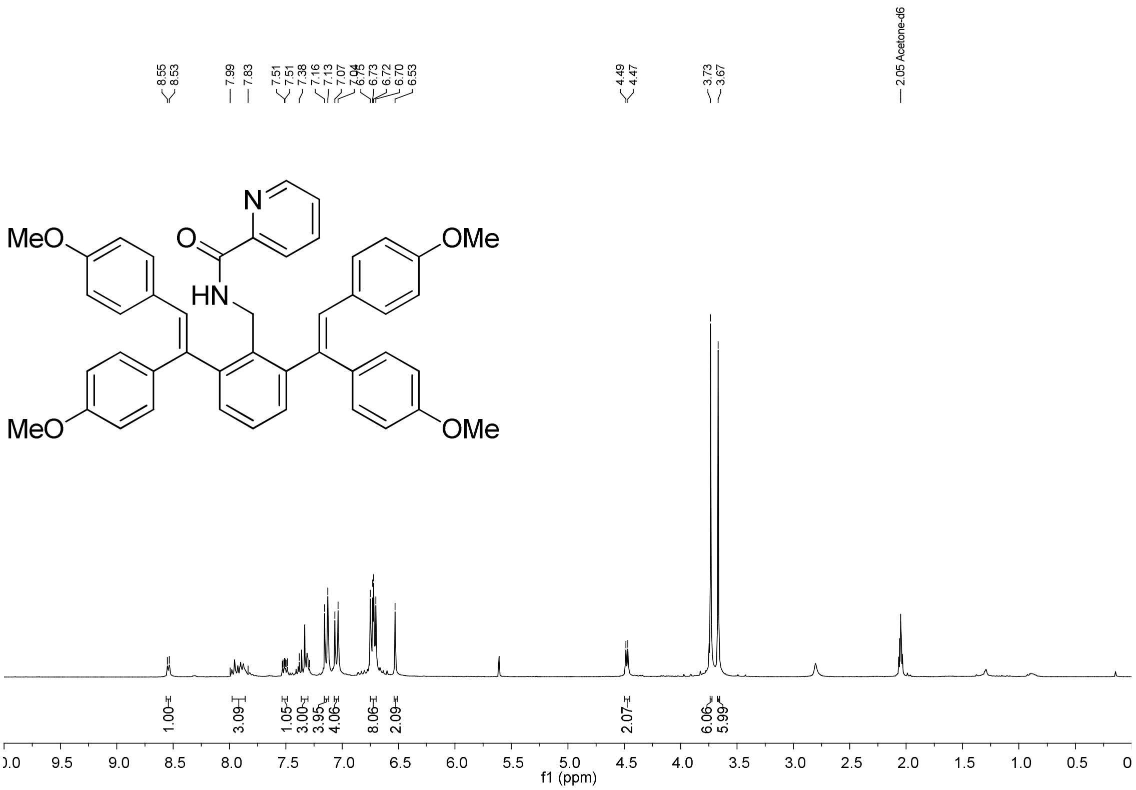

^{13}C NMR (acetone-d$_6$, 75 MHz)

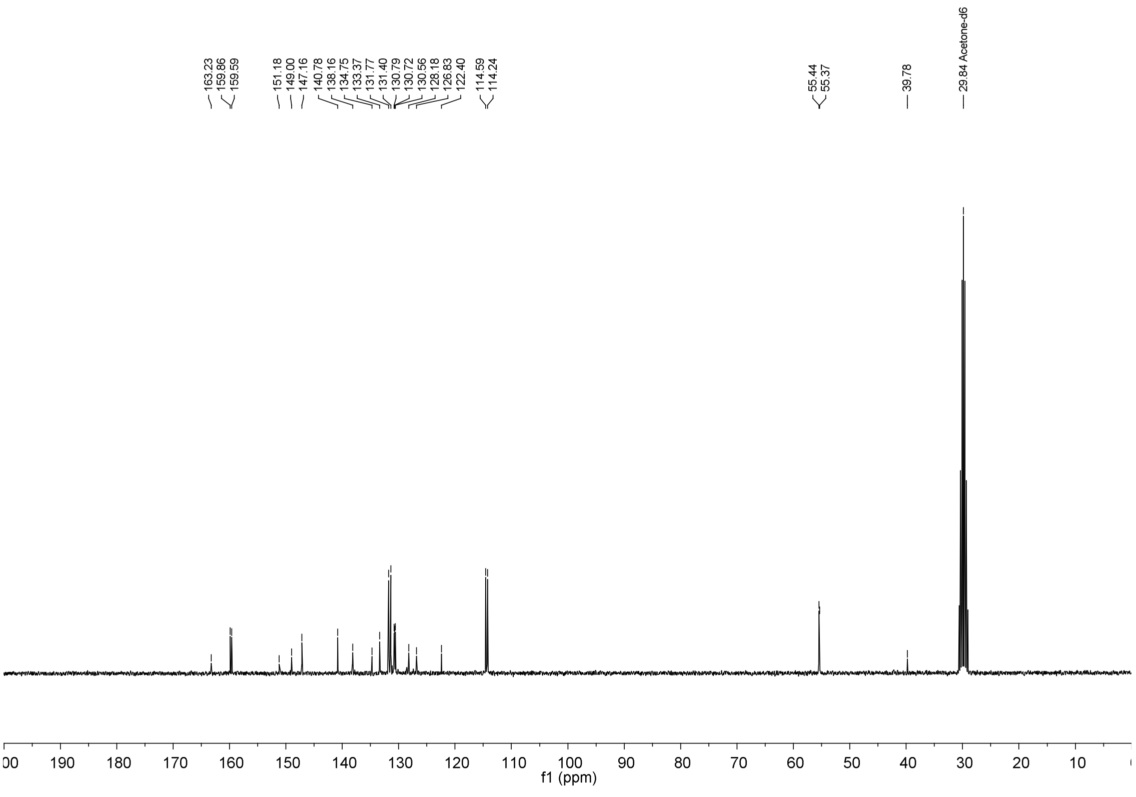

***N*-(2,6-Bis((*E*)-1,2-bis(4-(*tert*-butyl)phenyl)vinyl)benzyl)picolinamide (35)**

^{1}H NMR ($CDCl_3$, 300 MHz)

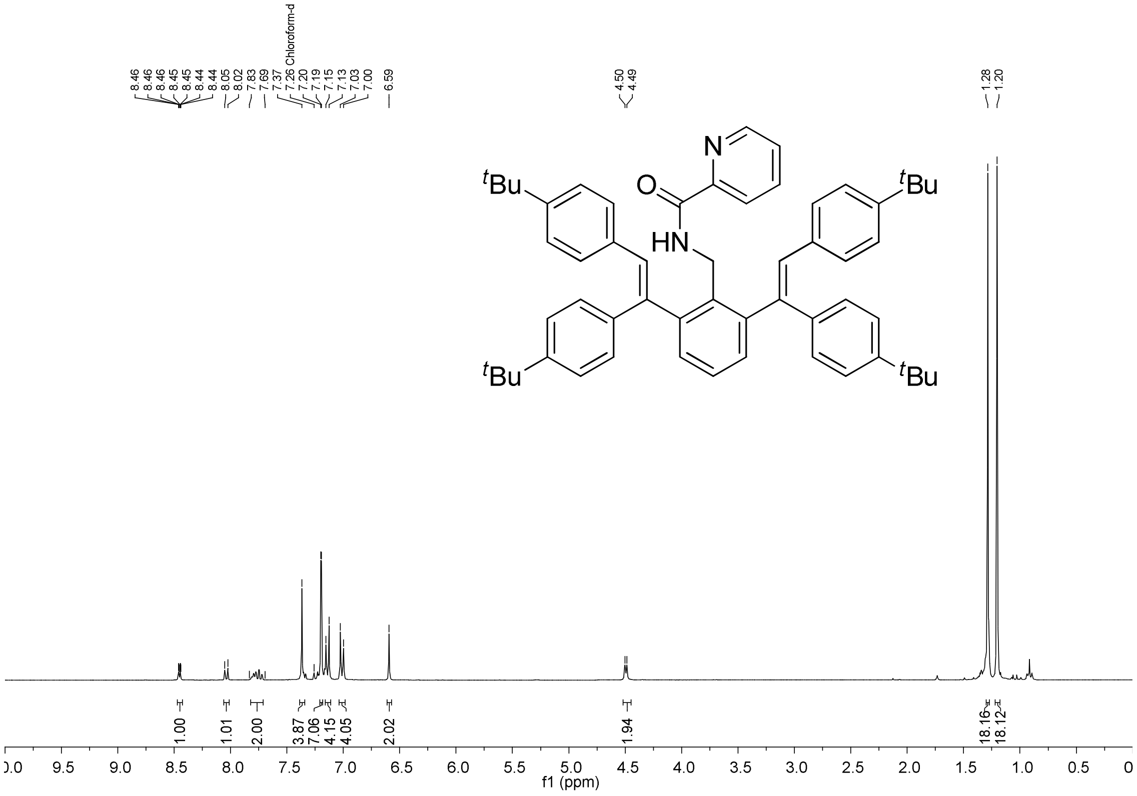

^{13}C NMR ($CDCl_3$, 75 MHz)

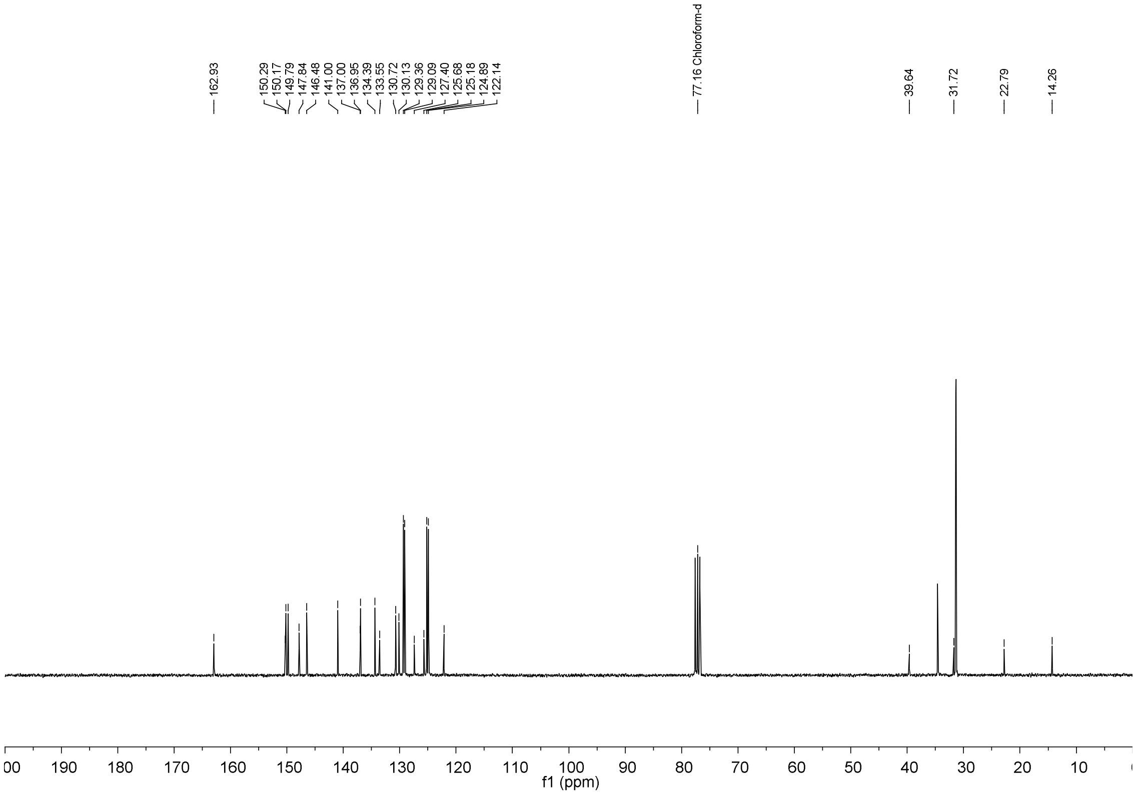

***N*-(2,6-Bis((*E*)-1,2-di-*p*-tolylvinyl)benzyl)picolinamide (36)**

^{1}H NMR (acetone-d$_6$, 300 MHz)

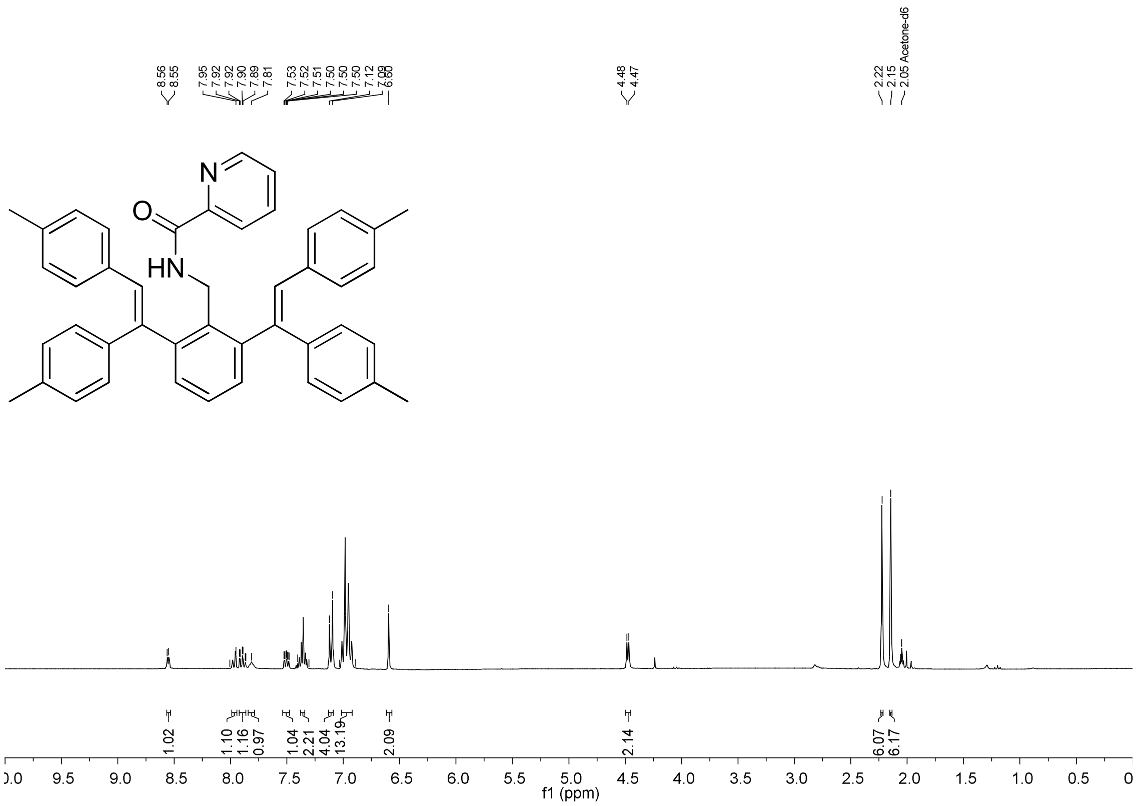

^{13}C NMR (acetone-d$_6$, 75 MHz)

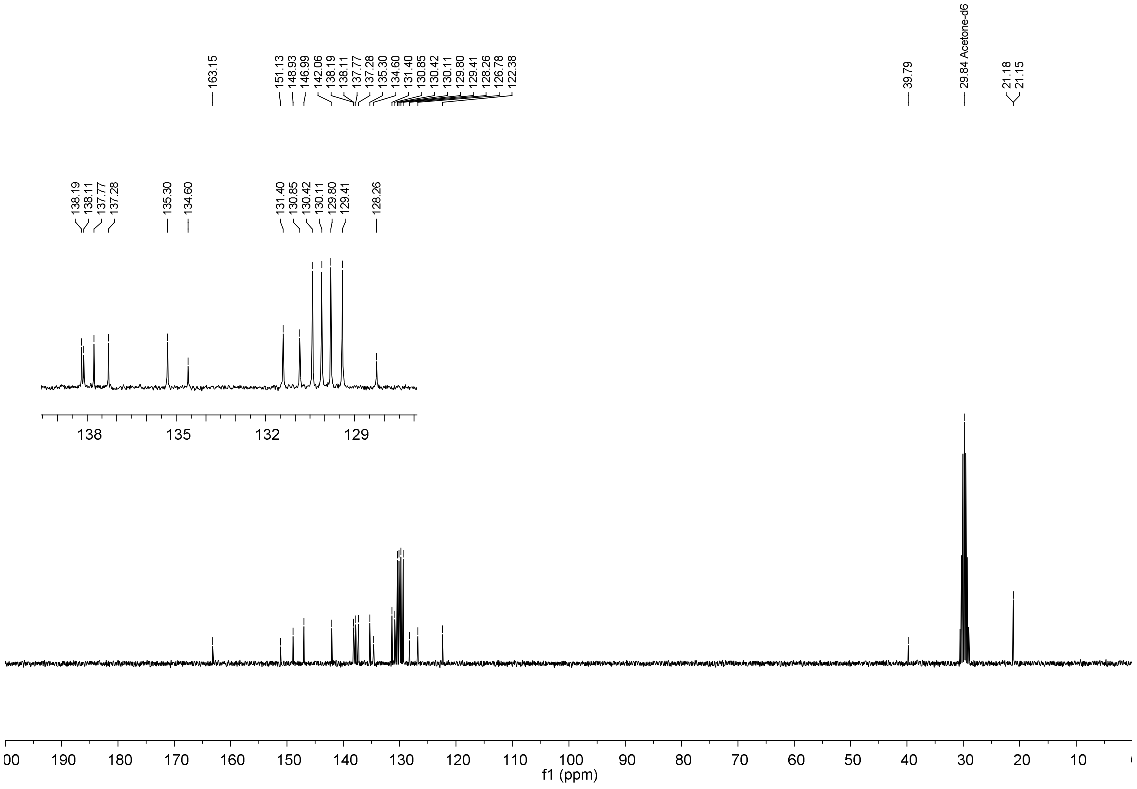

_N_-(2,6-Bis((_E_)-1,2-bis(4-(trifluoromethyl)phenyl)vinyl)benzyl)picolinamide (37)

^{1}H NMR (acetone-d$_6$, 300 MHz)

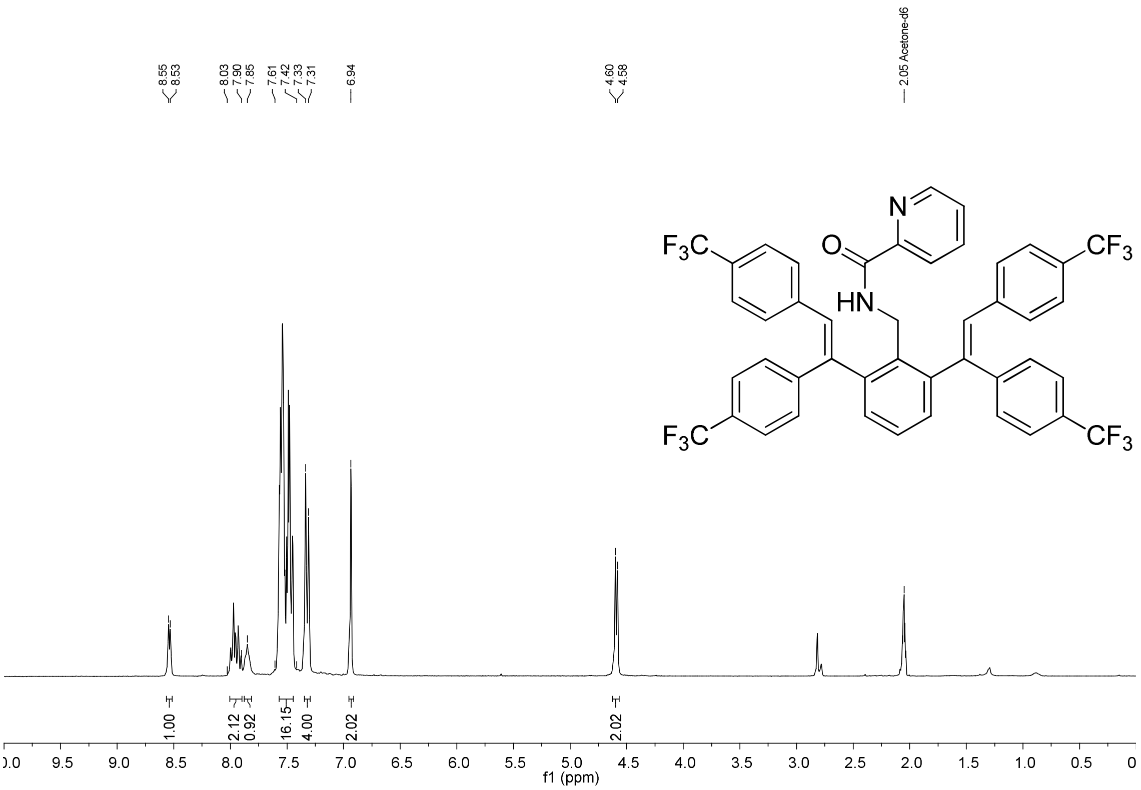

^{13}C NMR (acetone-d$_6$, 75 MHz)

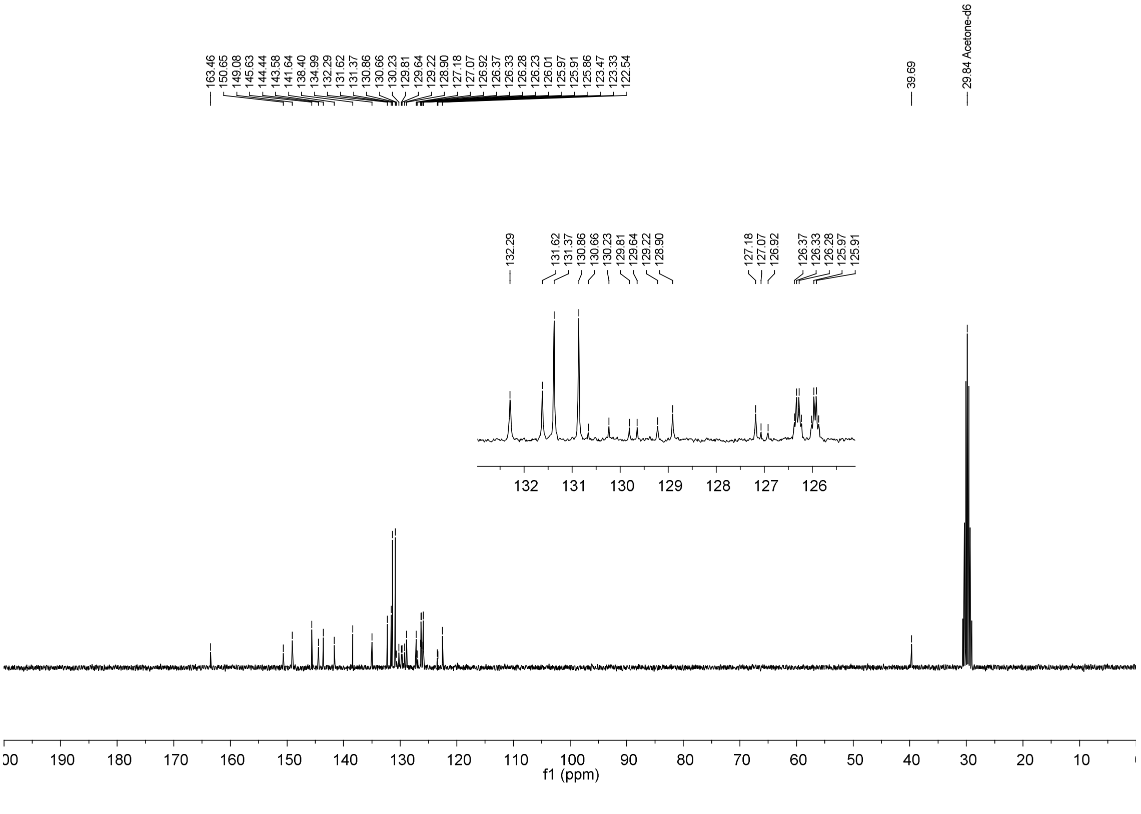

_N_-(2,6-Bis((_E_)-1,2-bis(3-methoxyphenyl)vinyl)benzyl)picolinamide (38)

^{1}H NMR ($CDCl_3$, 300 MHz)

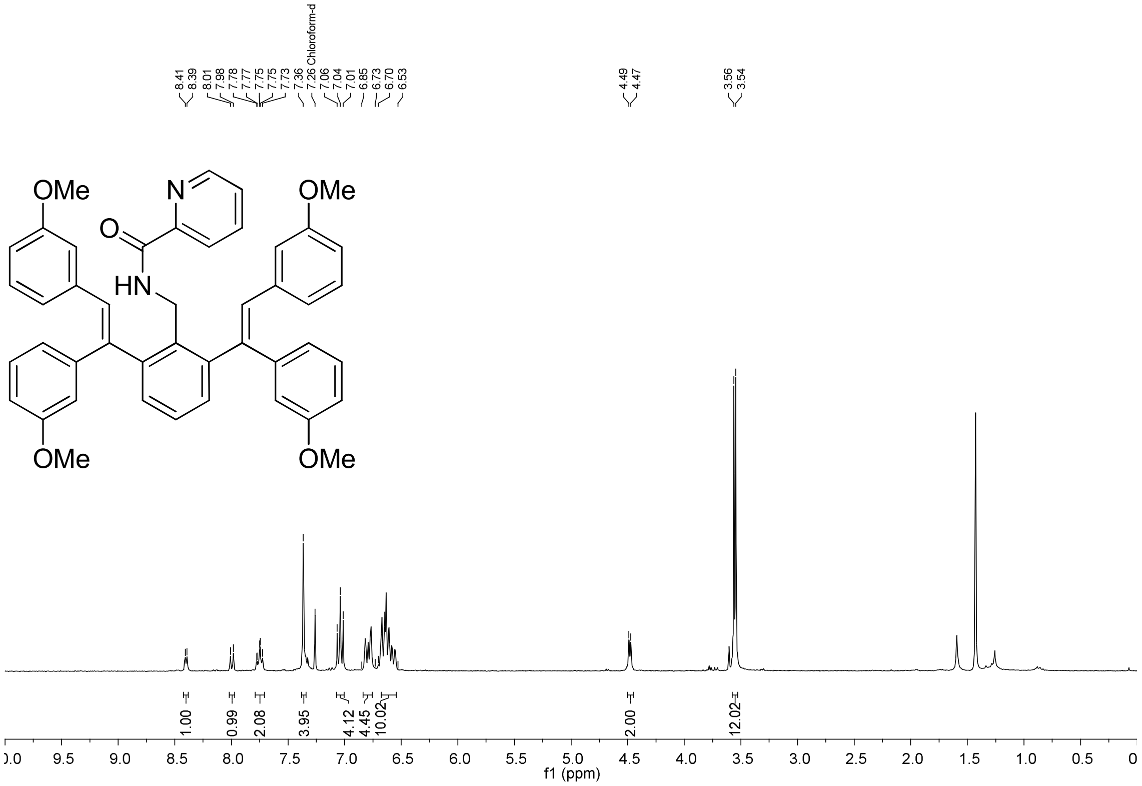

^{13}C NMR ($CDCl_3$, 125 MHz)

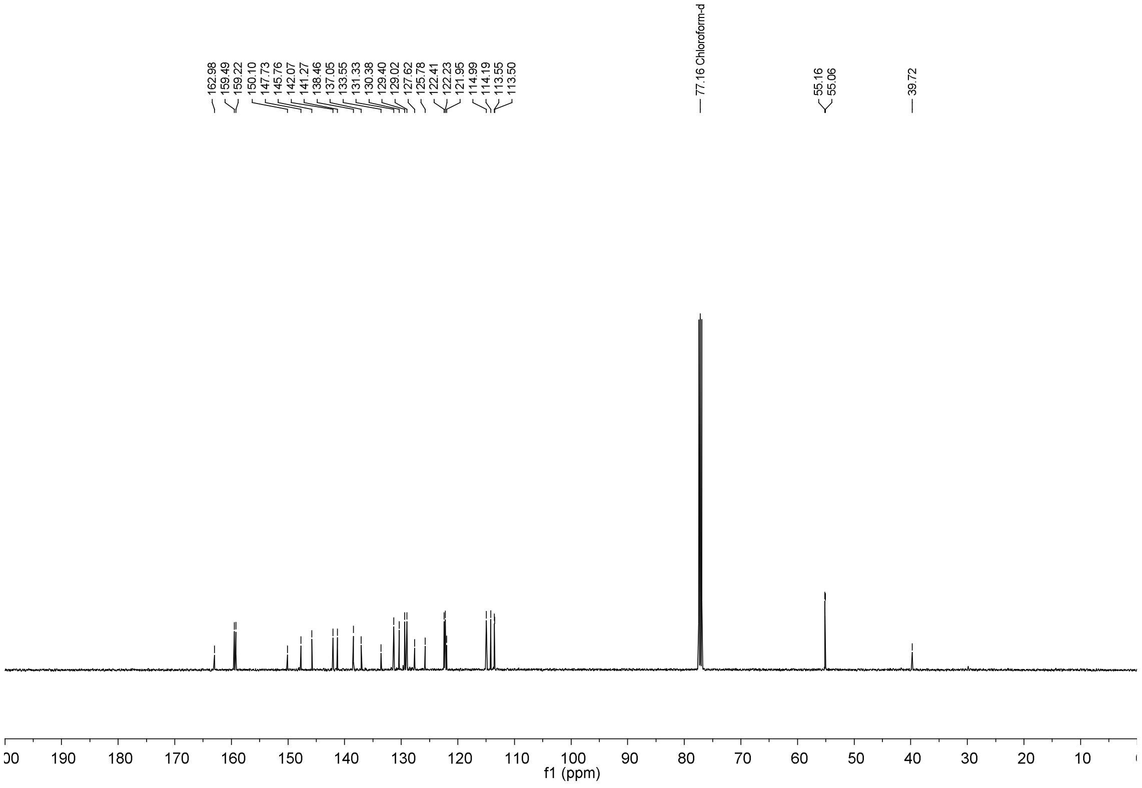

_N_-(2,6-Bis((_E_)-1-cyclohexyl-2-(4-methoxyphenyl)vinyl)benzyl)picolinamide (58)

^{1}H NMR ($CDCl_3$, 300 MHz)

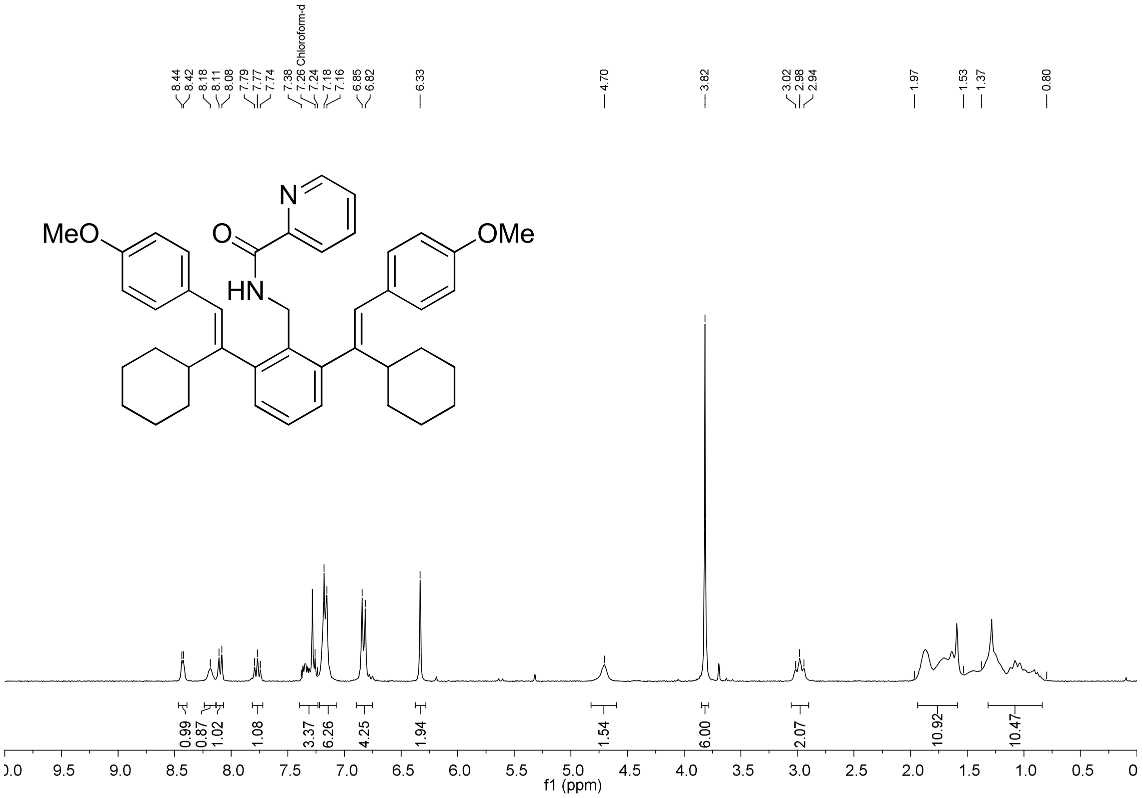

^{13}C NMR ($CDCl_3$, 75 MHz)

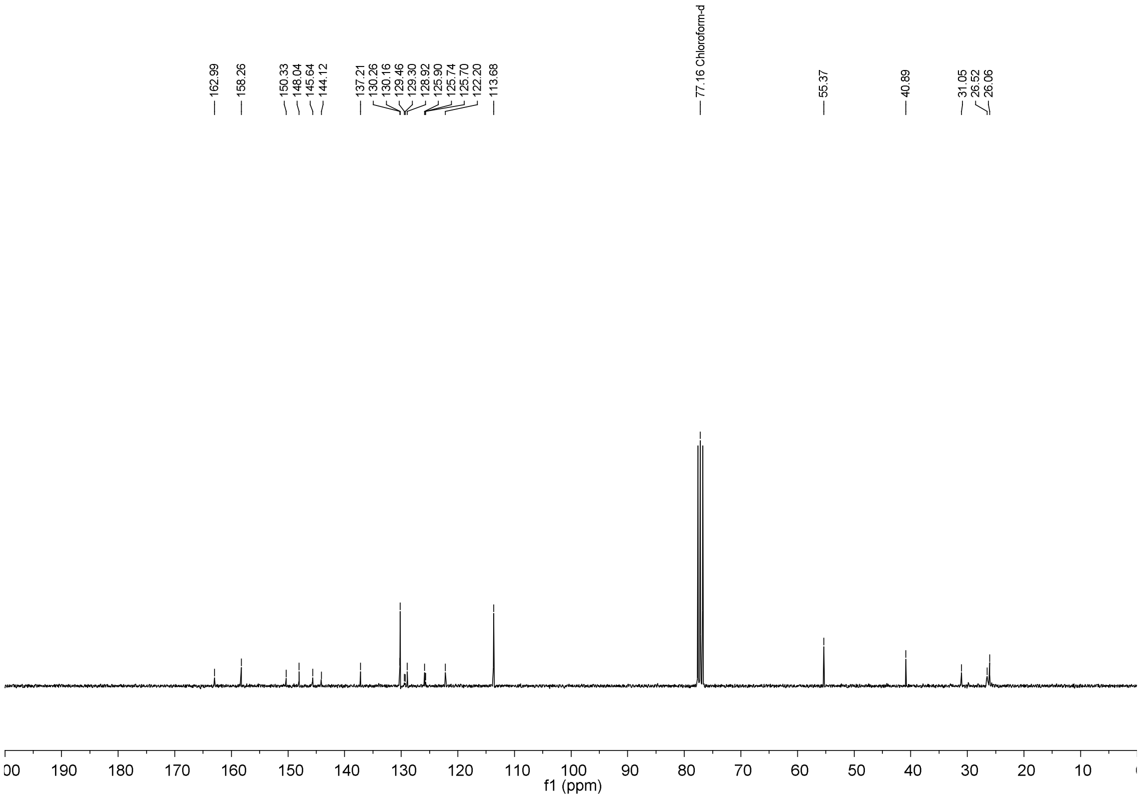

***N*-(2,6-Bis((*E*)-1-cyclohexyl-2-(4-(trifluoromethyl)phenyl)vinyl)benzyl)picolinamide (59)**

^{1}H NMR ($CDCl_3$, 300 MHz)

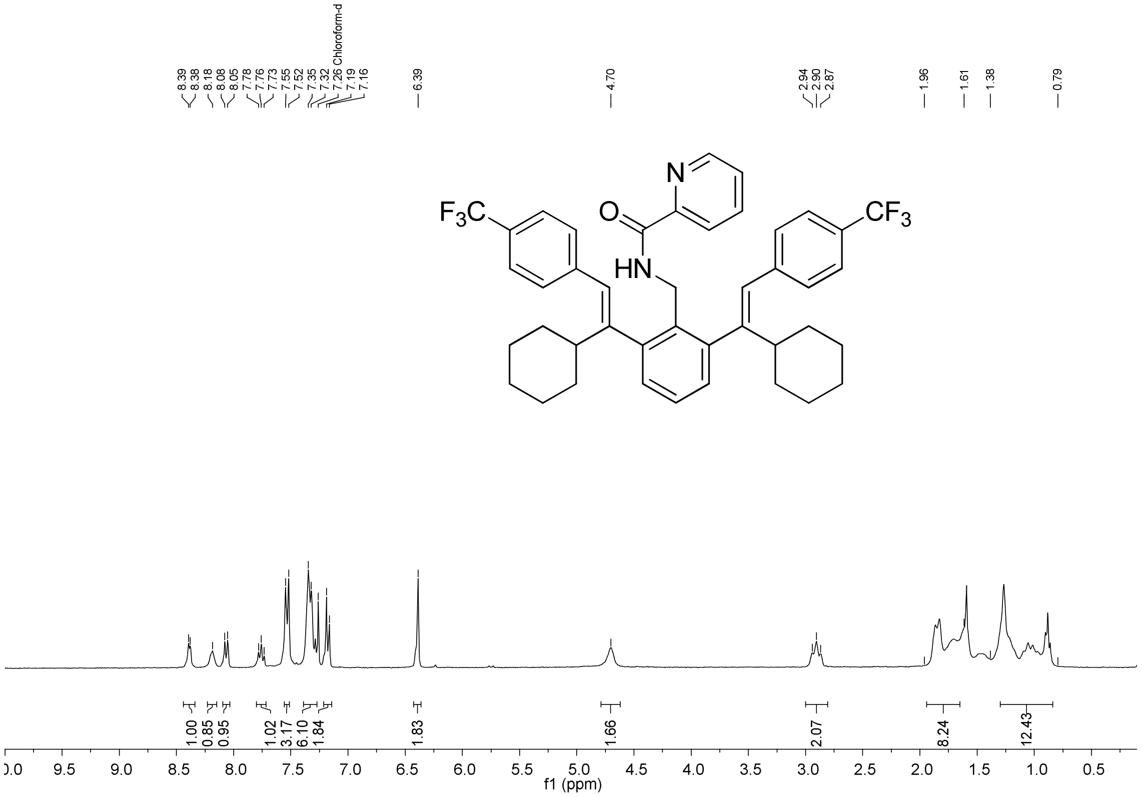

^{13}C NMR ($CDCl_3$, 75 MHz)

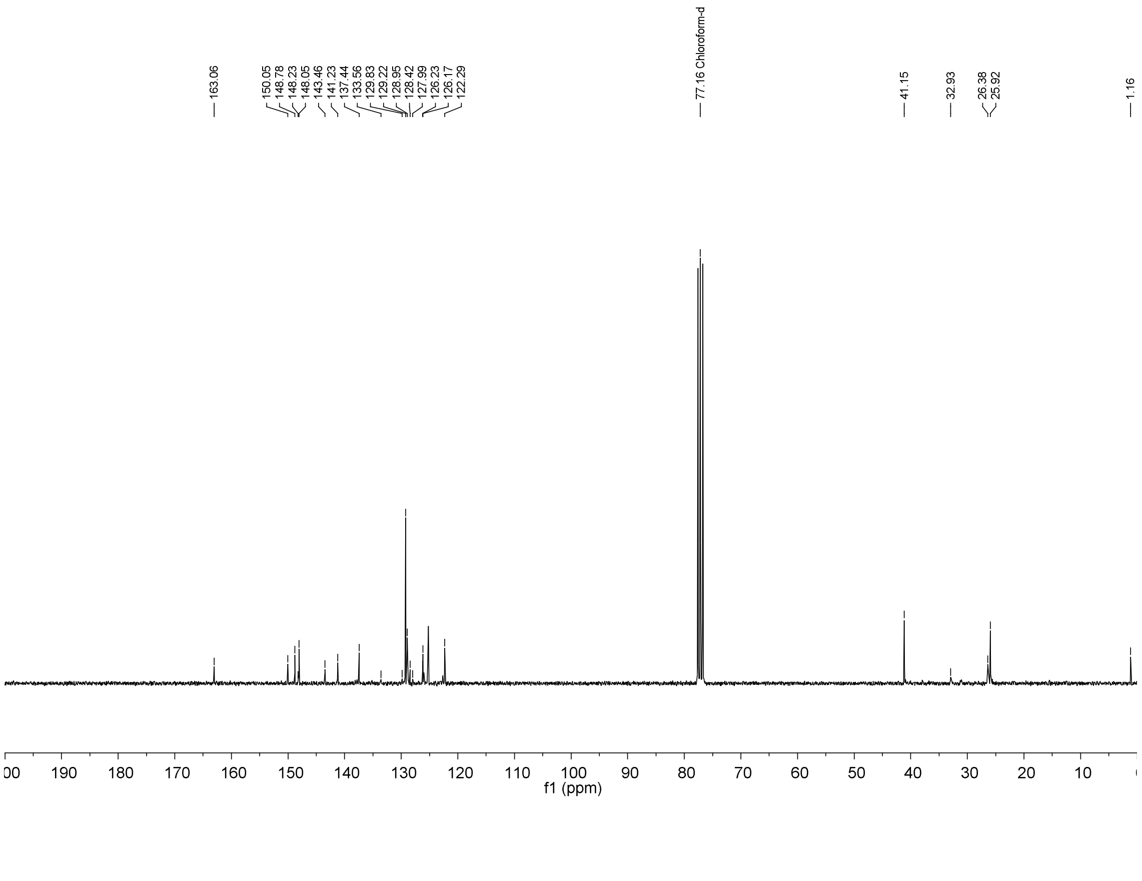

N-(2,6-Bis((E)-1-cyclohexyl-2-(thiophen-3-yl)vinyl)benzyl)picolinamide (60)

^{1}H NMR ($CDCl_3$, 300 MHz)

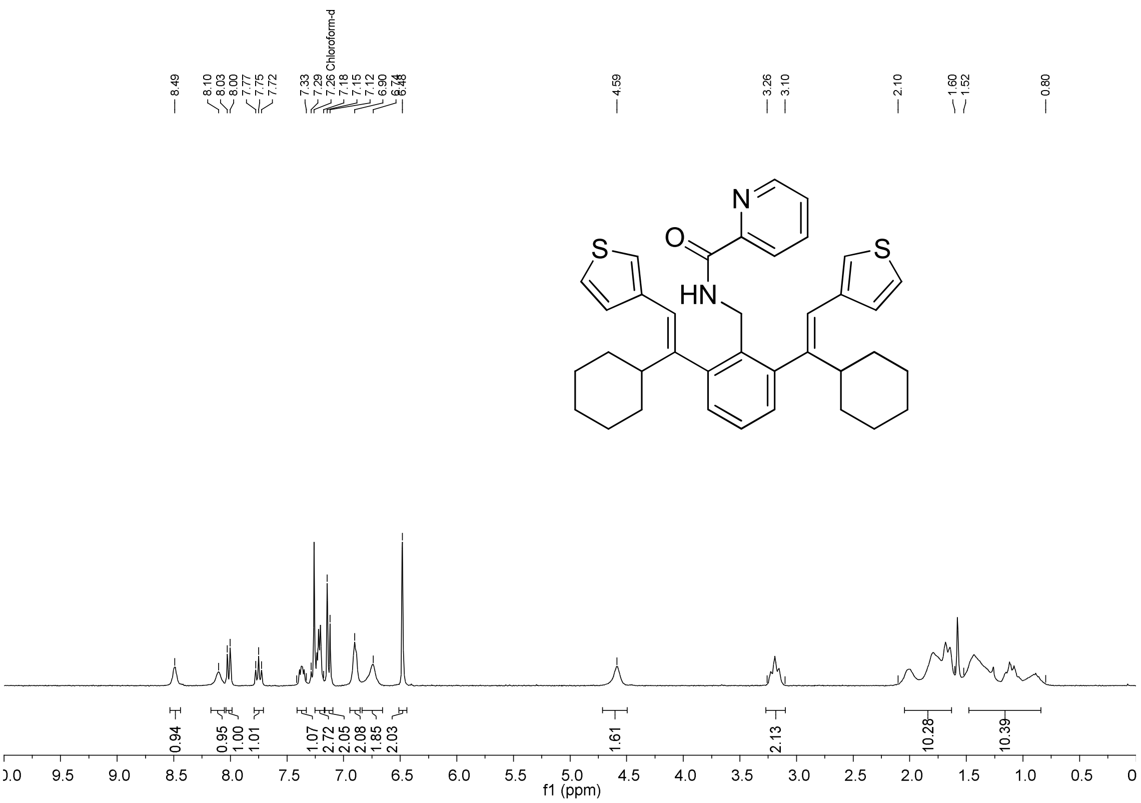

^{13}C NMR ($CDCl_3$, 75 MHz)

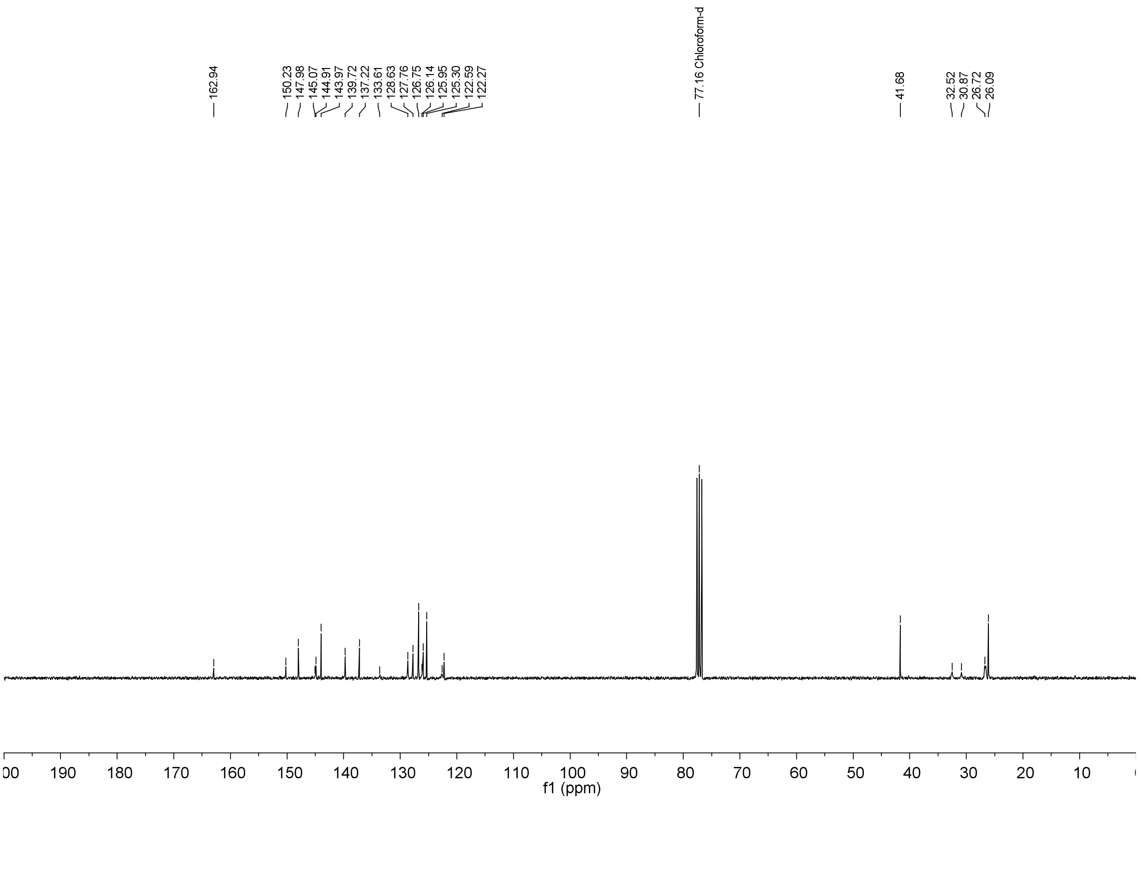

(2*E*,2'*E*,4*E*,4'*E*)-Dimethyl 5,5'-(2-(picolinamidomethyl)-1,3-phenylene)bis(6-cyclohexylhexa-2,4-dienoate) (55)

^{1}H NMR ($CDCl_3$, 300 MHz)

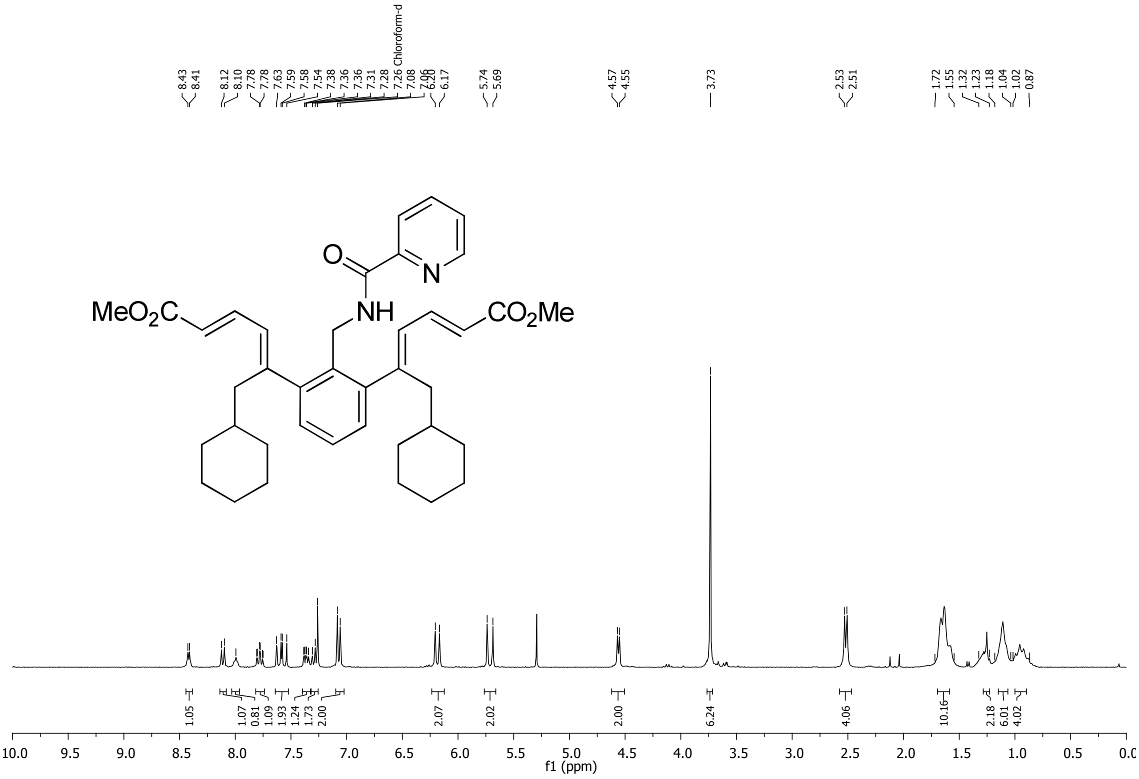

^{13}C NMR ($CDCl_3$, 75 MHz)

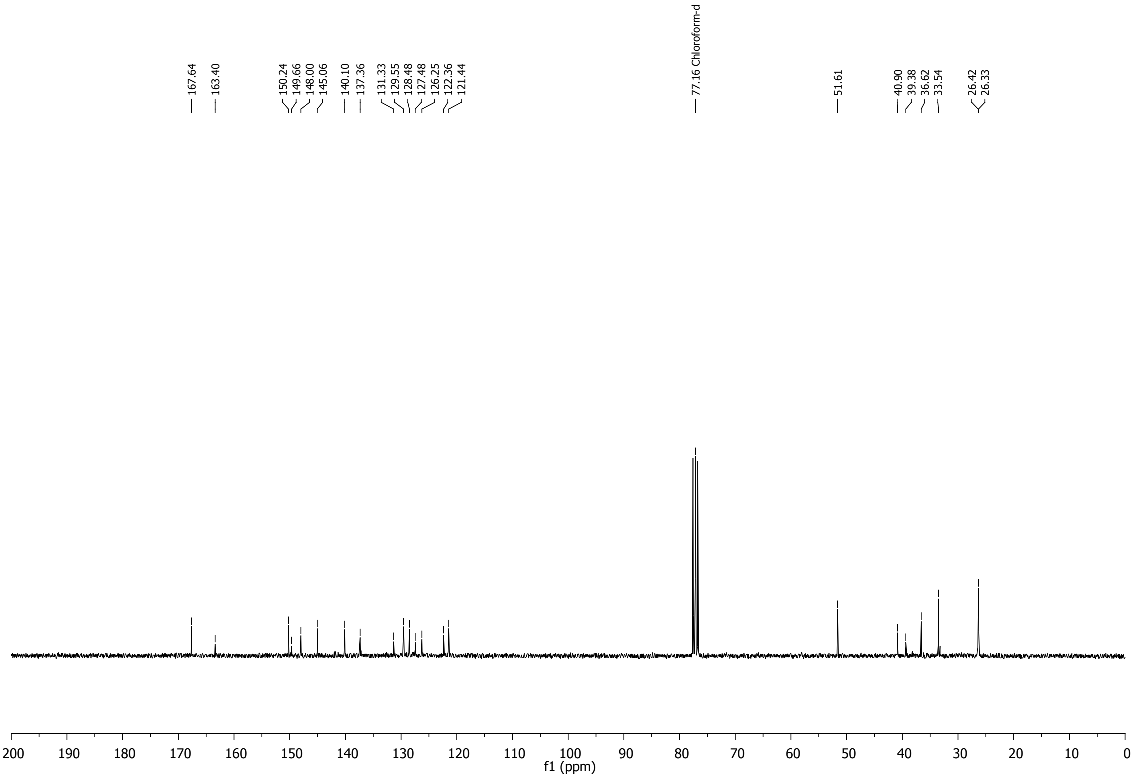

(2*E*,2'*E*,4*E*,4'*E*)-Dimethyl 5,5'-(2-(picolinamidomethyl)-1,3-phenylene)bis(7-phenylhepta-2,4-dienoate) (56)

^{1}H NMR ($CDCl_3$, 300 MHz)

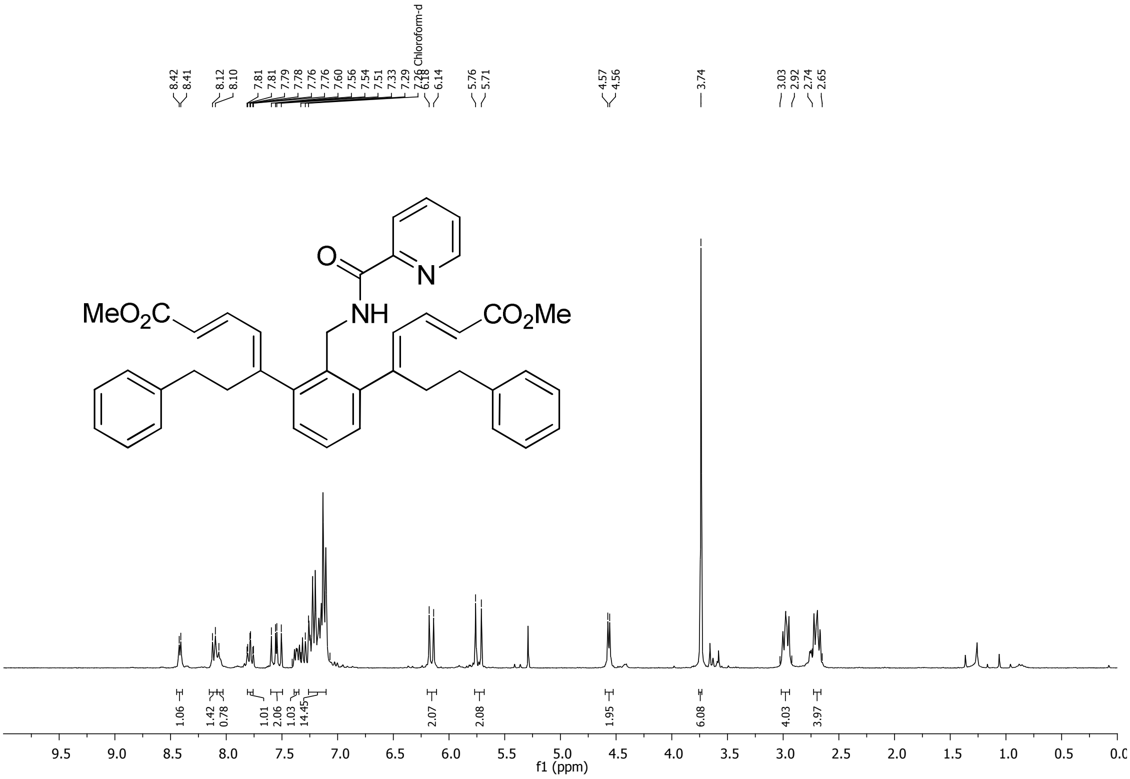

^{13}C NMR ($CDCl_3$, 75 MHz)

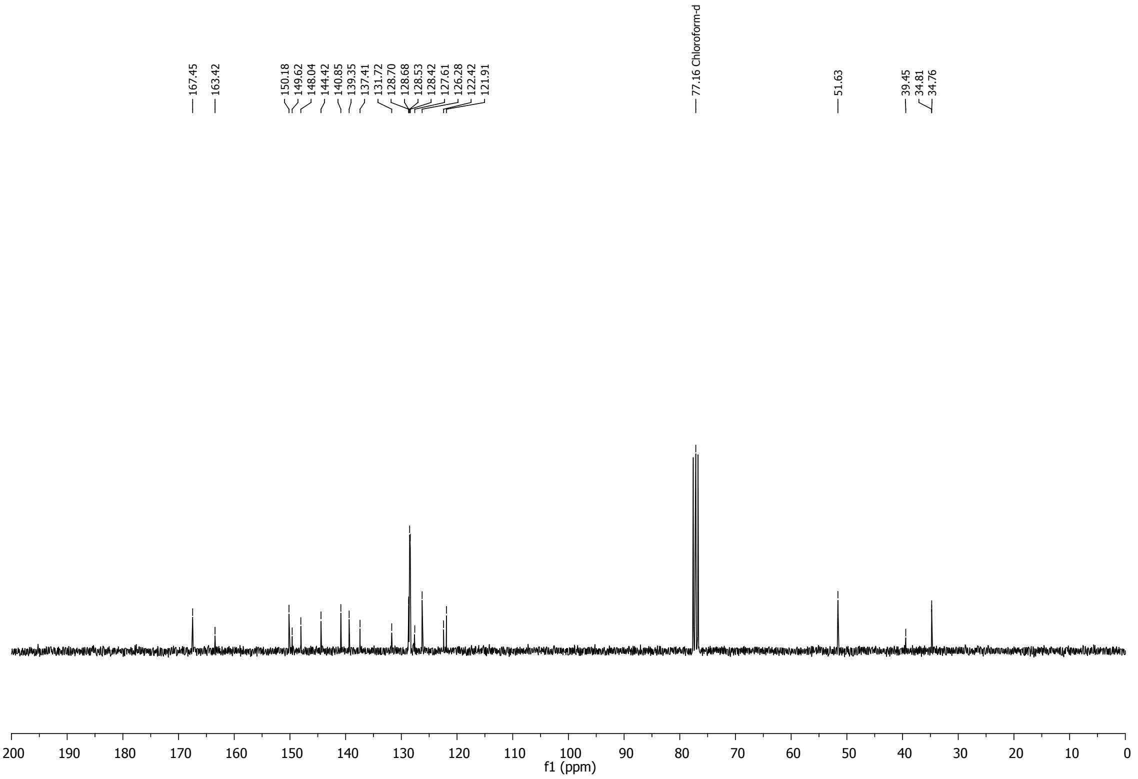

(2*E*,2'*E*,4*E*,4'*E*)-Dimethyl 5,5'-(2-(picolinamidomethyl)-1,3-phenylene)bis(9-chloronona-2,4-dienoate) (57)

^{1}H NMR ($CDCl_3$, 300 MHz)

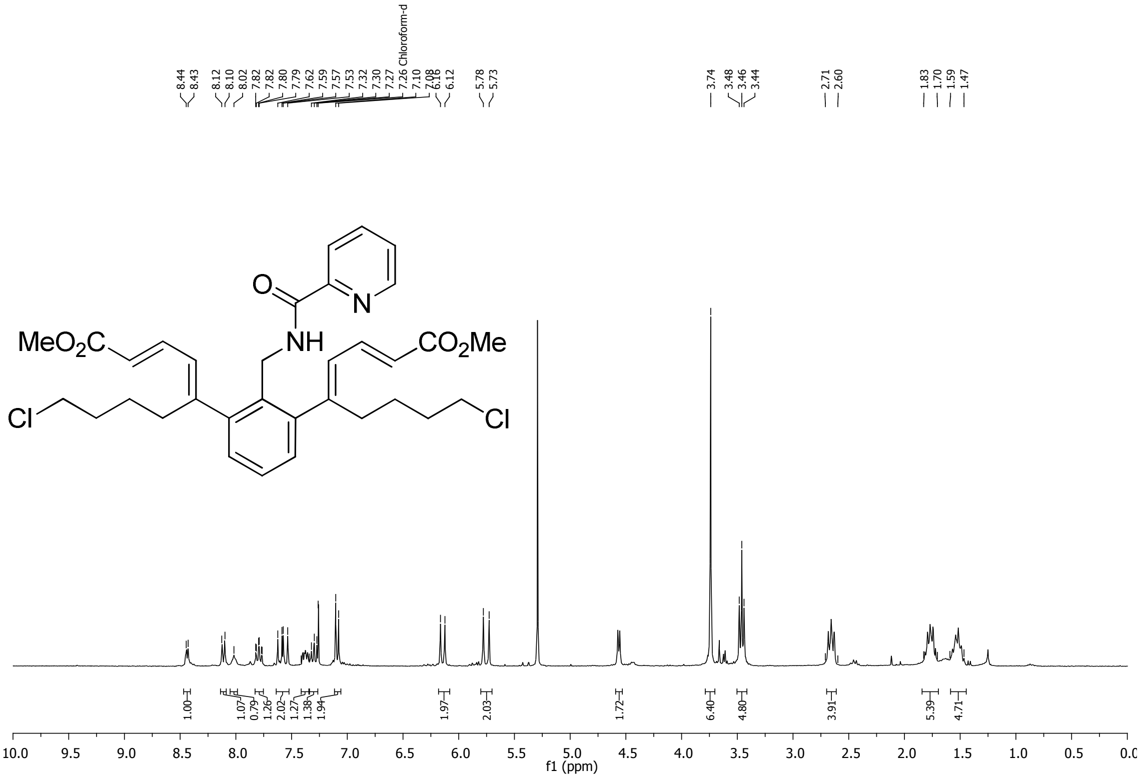

^{13}C NMR ($CDCl_3$, 75 MHz)

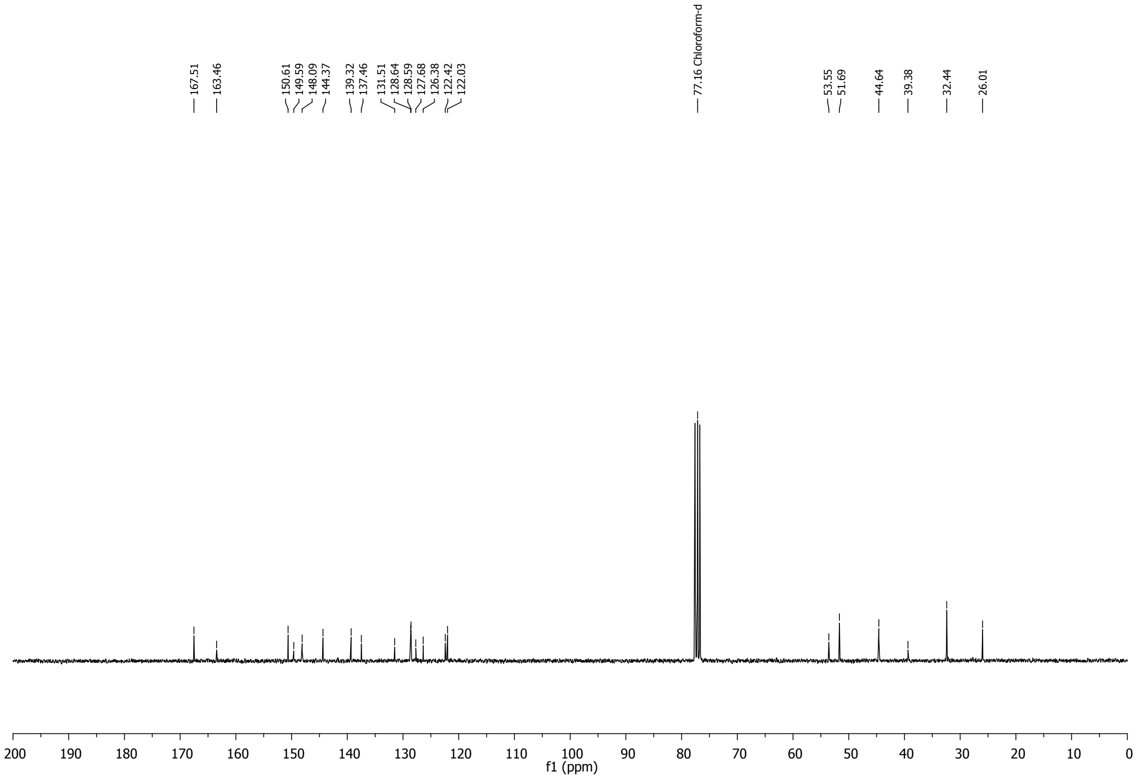

Ethyl 2-(6-benzyl-5-ethyl-7-oxo-6,7-dihydro-5H-pyrrolo[3,4-*b*]pyridin-5-yl)acetate (53)

^{1}H NMR ($CDCl_3$, 300 MHz)

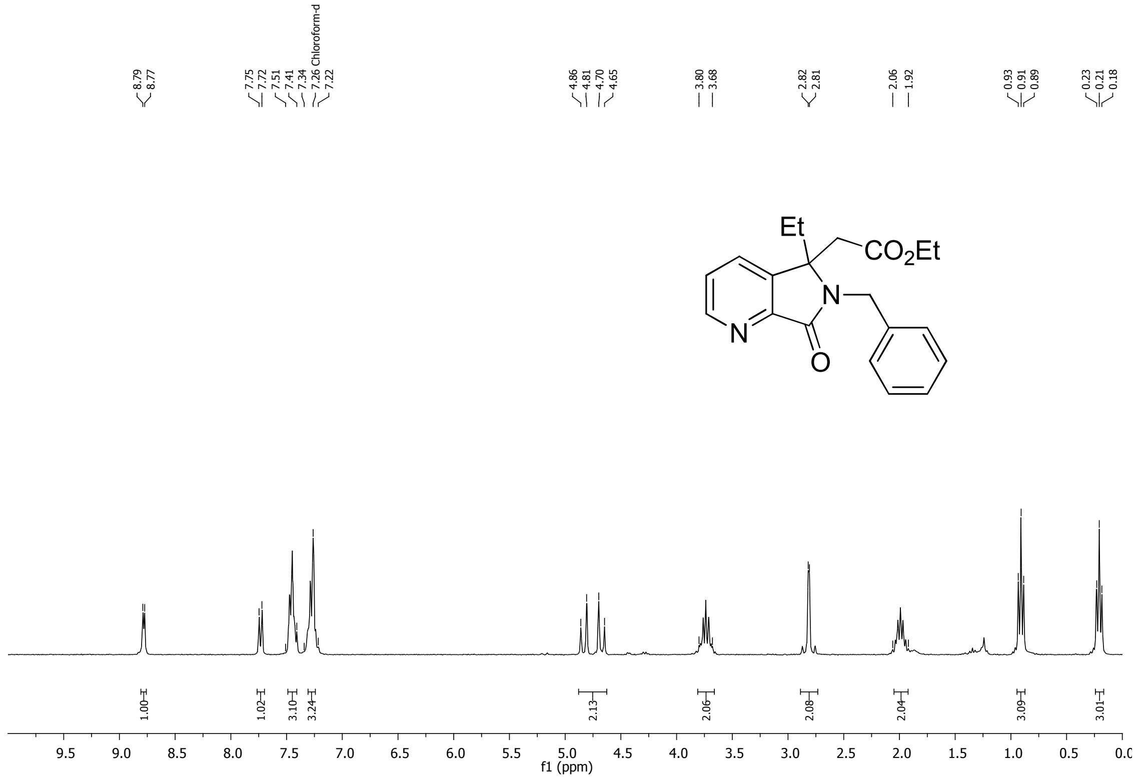

^{13}C NMR ($CDCl_3$, 75 MHz)

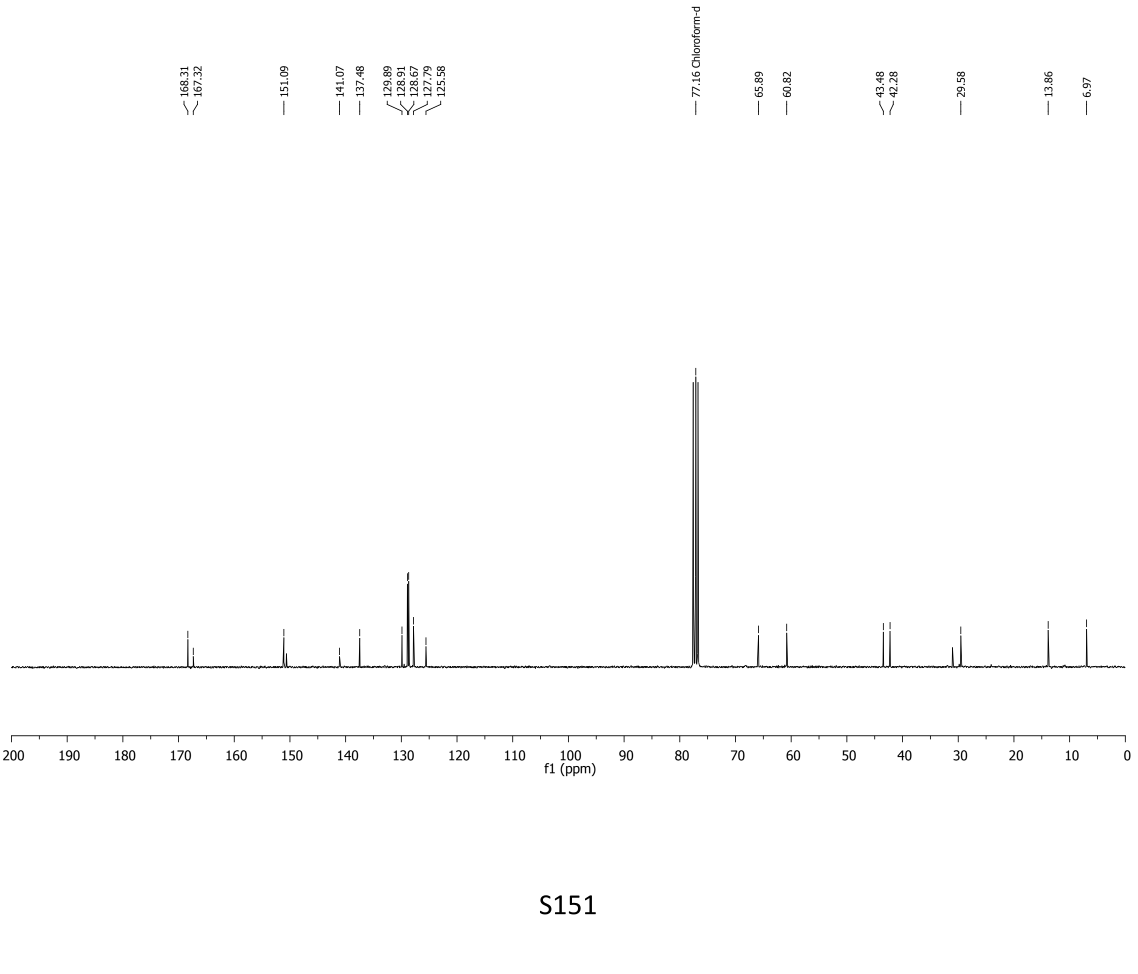

(2*E*,2'*E*)-Diethyl 3,3'-(2-(picolinamidomethyl)-1,3-phenylene)bis(pent-2-enoate) (54)

^{1}H NMR ($CDCl_3$, 300 MHz)

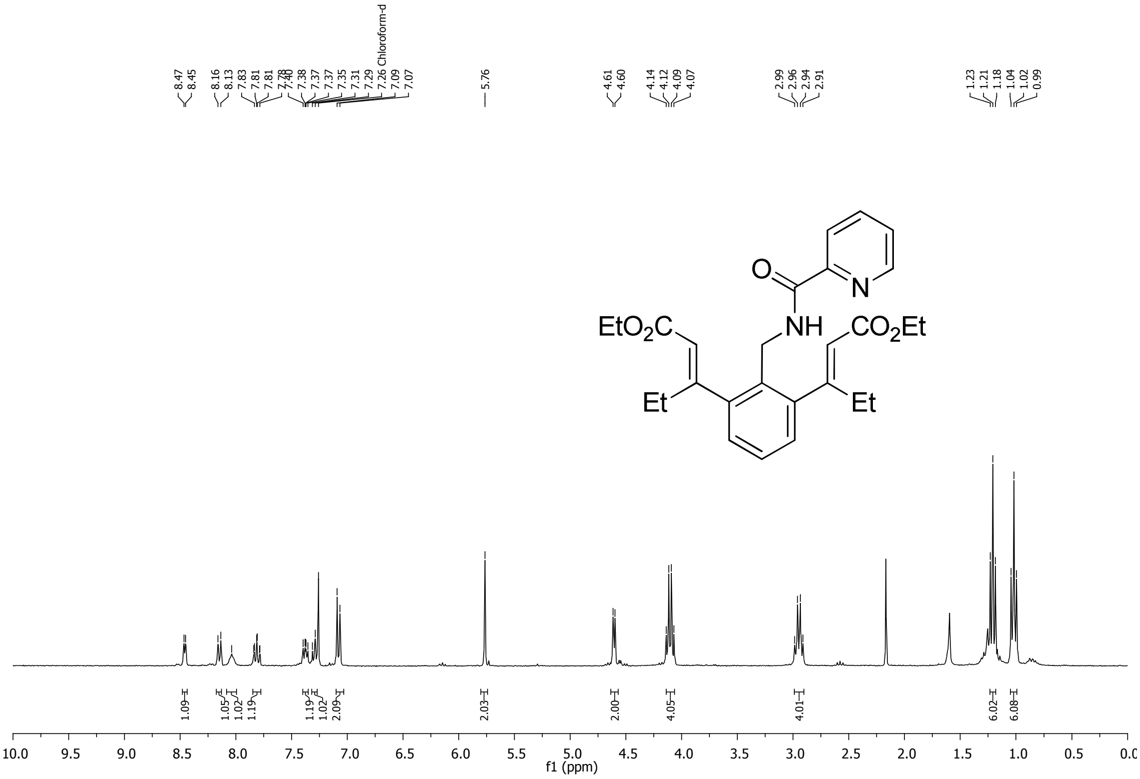

^{13}C NMR ($CDCl_3$, 75 MHz)

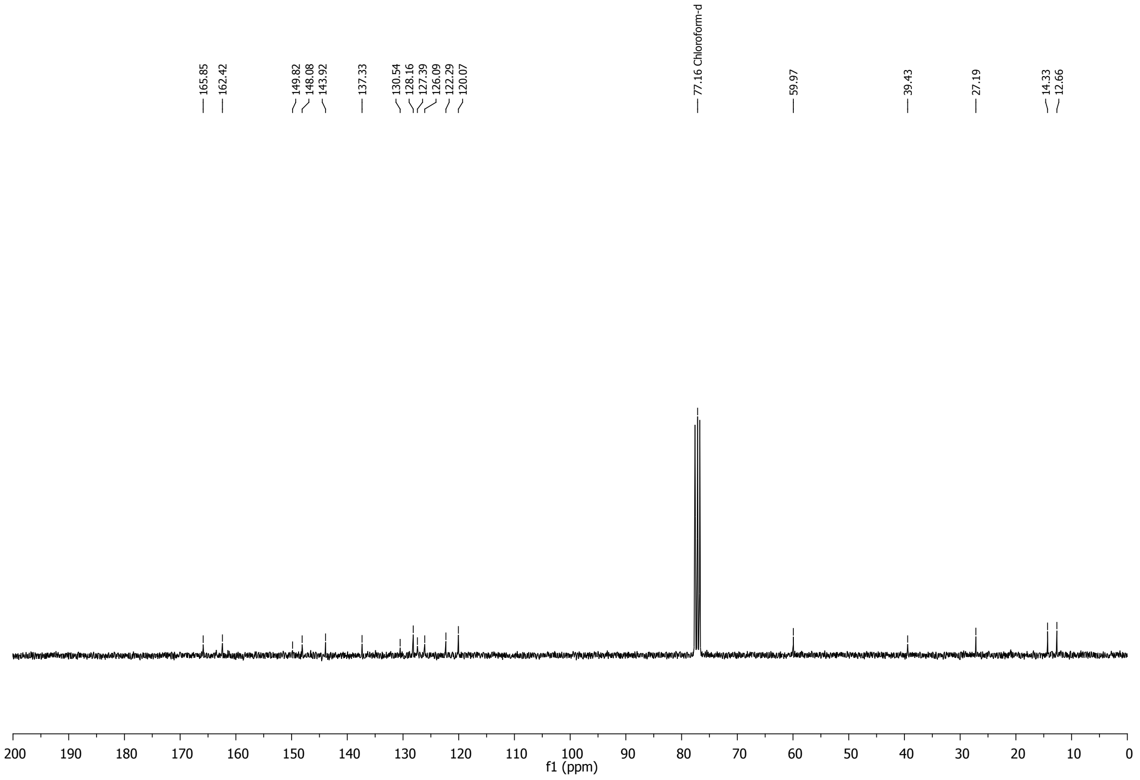

N-(2,6-Bis((E)-1,2-diphenylvinyl)phenethyl)picolinamide (73)

^{1}H NMR (acetone-d$_6$, 300 MHz)

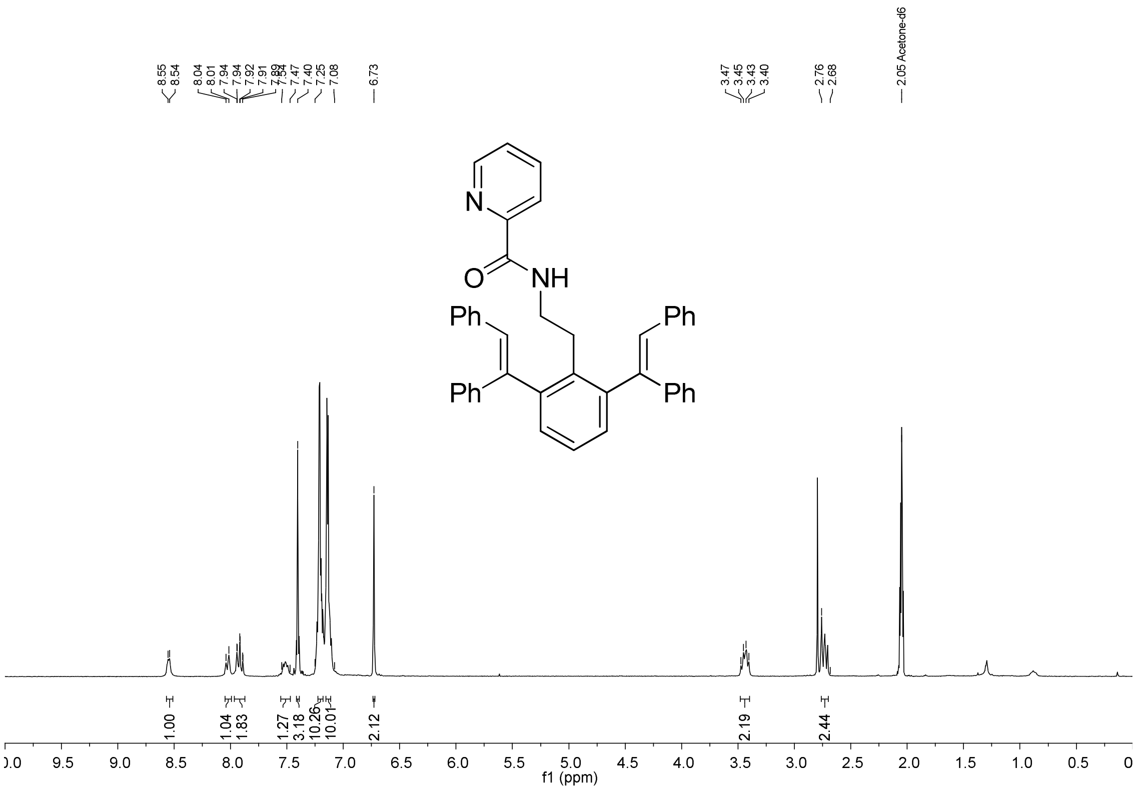

^{13}C NMR ($CDCl_3$, 125 MHz)

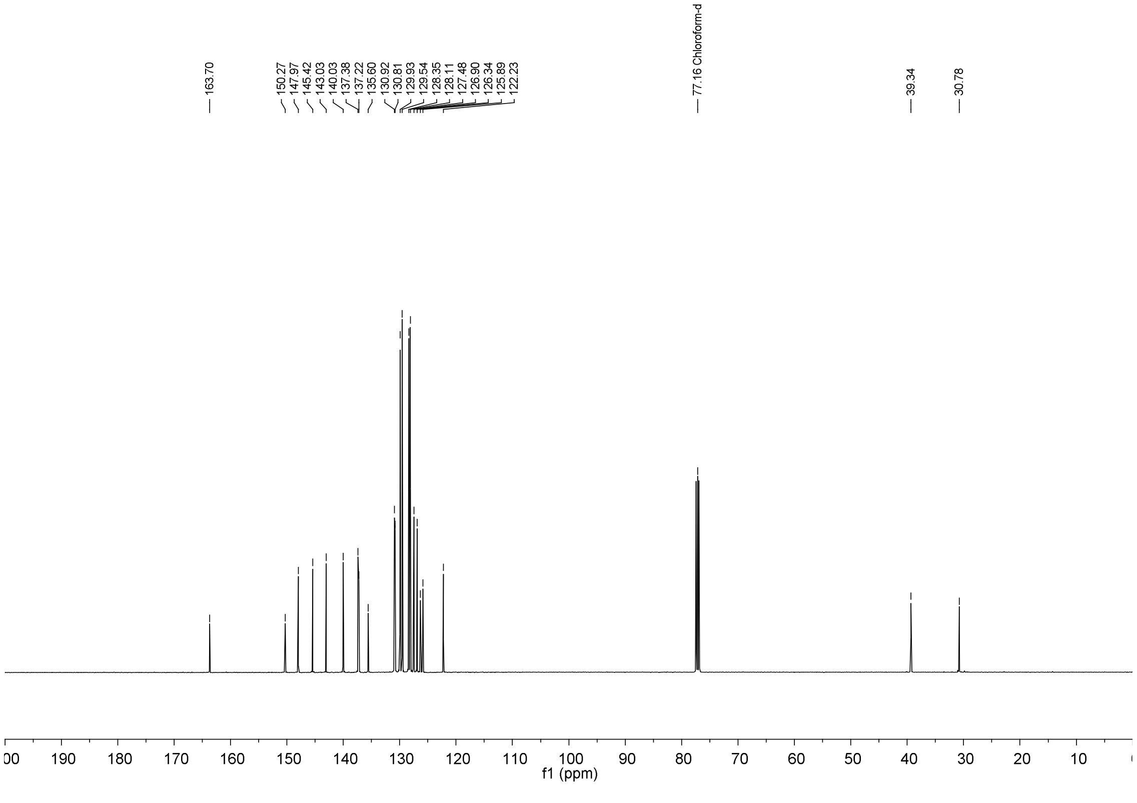

N-(2,6-Bis((E)-1,2-diphenylvinyl)-4-methoxyphenethyl)picolinamide (77)

^{1}H NMR (CDCl$_3$, 300 MHz)

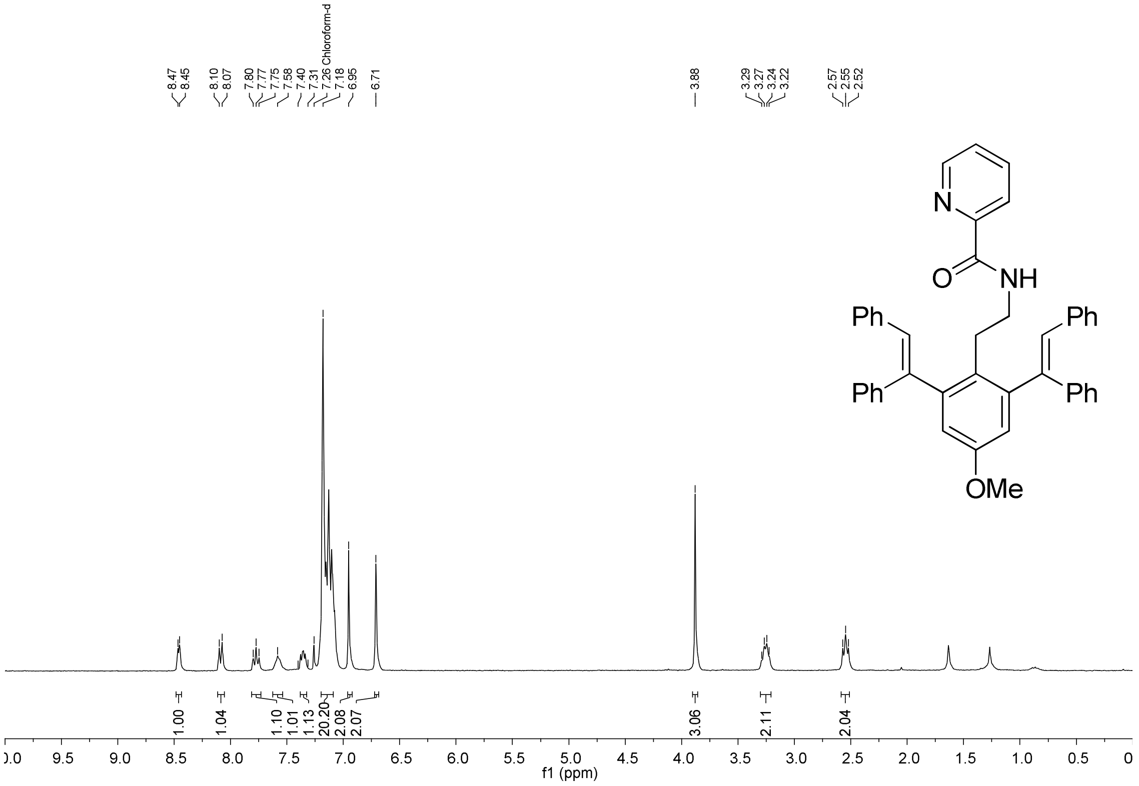

^{13}C NMR (CDCl$_3$, 125 MHz)

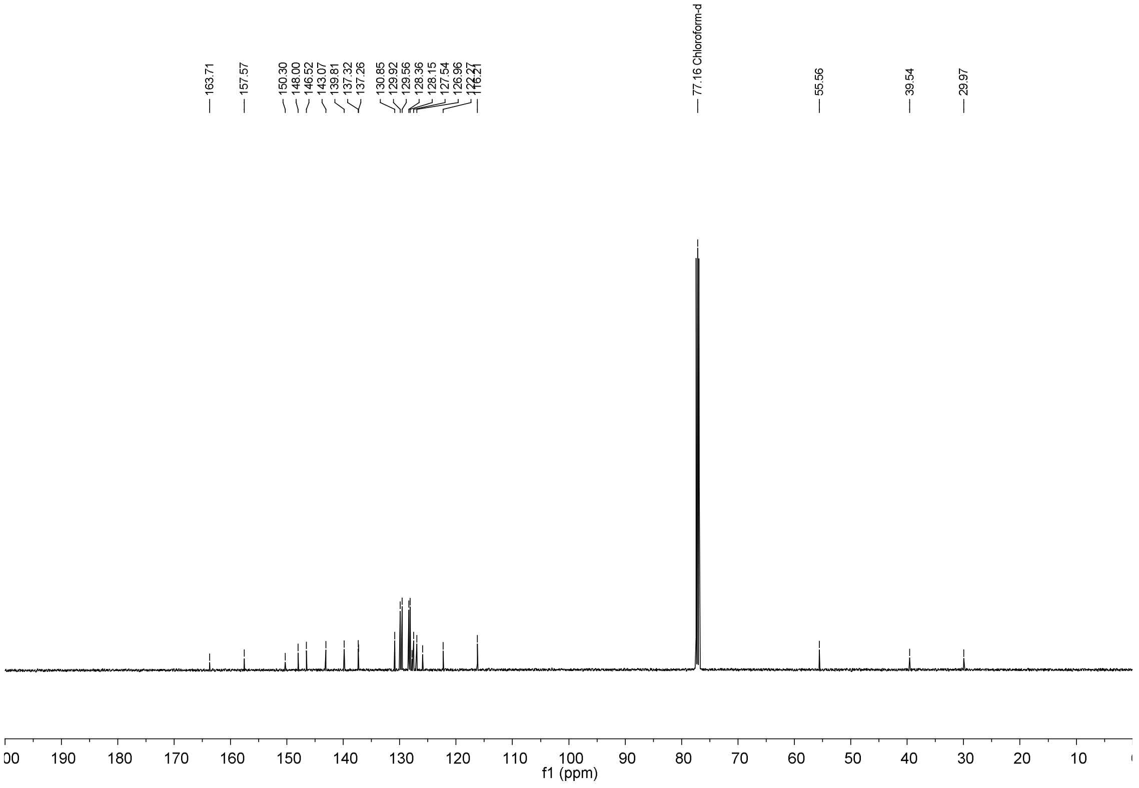

_N_-(4-Chloro-2,6-bis((_E_)-1,2-diphenylvinyl)phenethyl)picolinamide (78)

^{1}H NMR ($CDCl_3$, 300 MHz)

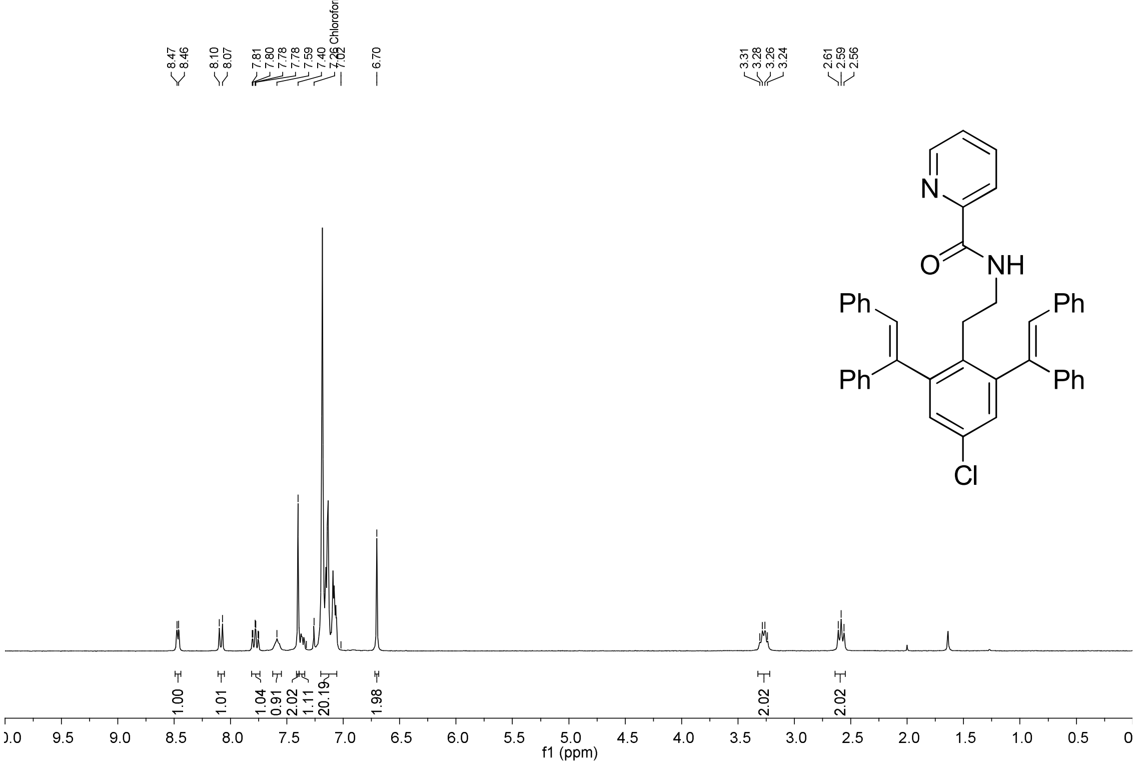

^{13}C NMR ($CDCl_3$, 75 MHz)

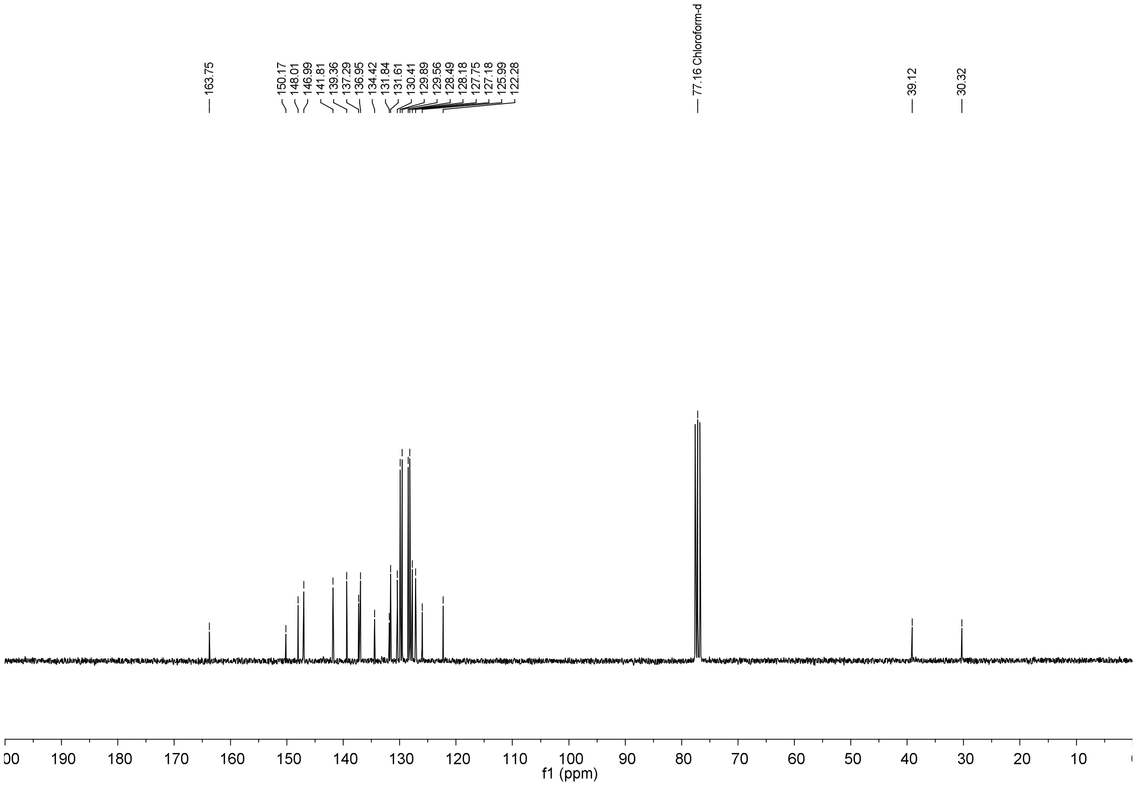

***N*-(2,6-Bis((*E*)-1,2-diphenylvinyl)-4-fluorophenethyl)picolinamide (79)**

^{1}H NMR ($CDCl_3$, 300 MHz)

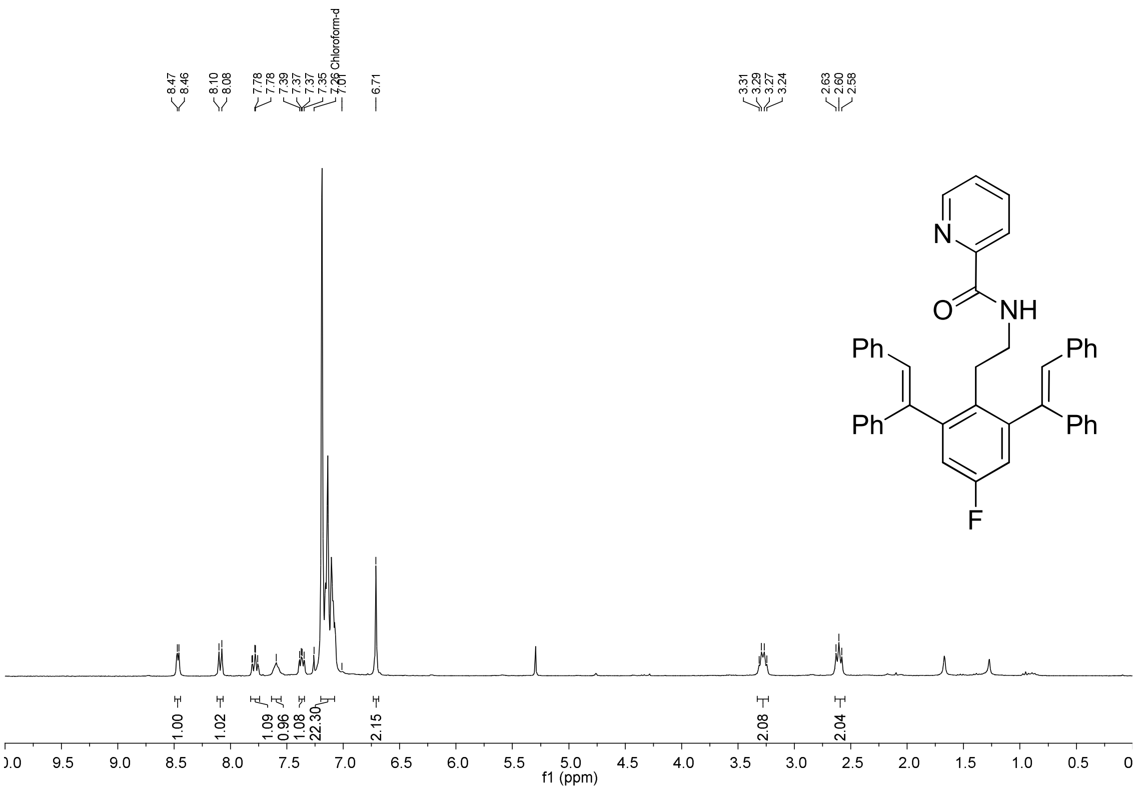

^{13}C NMR ($CDCl_3$, 125 MHz)

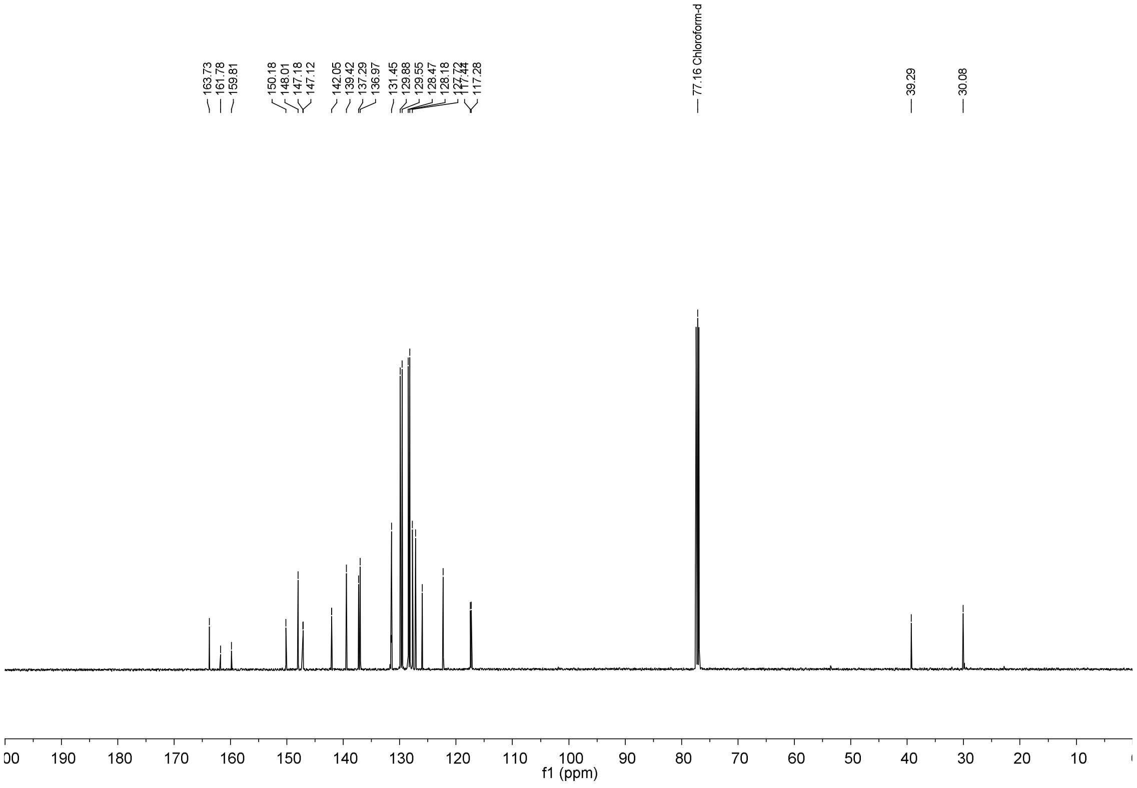

(*E*)-*N*-(2-(1,2-Diphenylvinyl)-5-methoxyphenethyl)picolinamide (80)

^{1}H NMR ($CDCl_3$, 300 MHz)

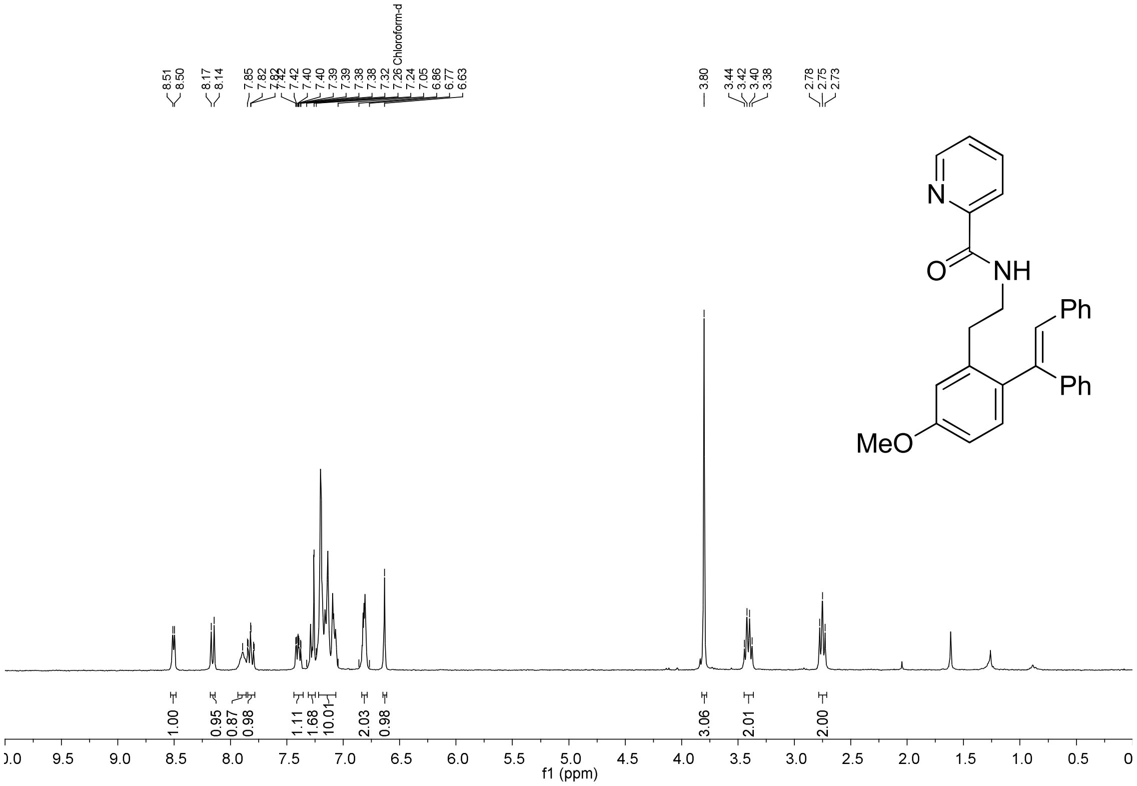

^{13}C NMR ($CDCl_3$, 75 MHz)

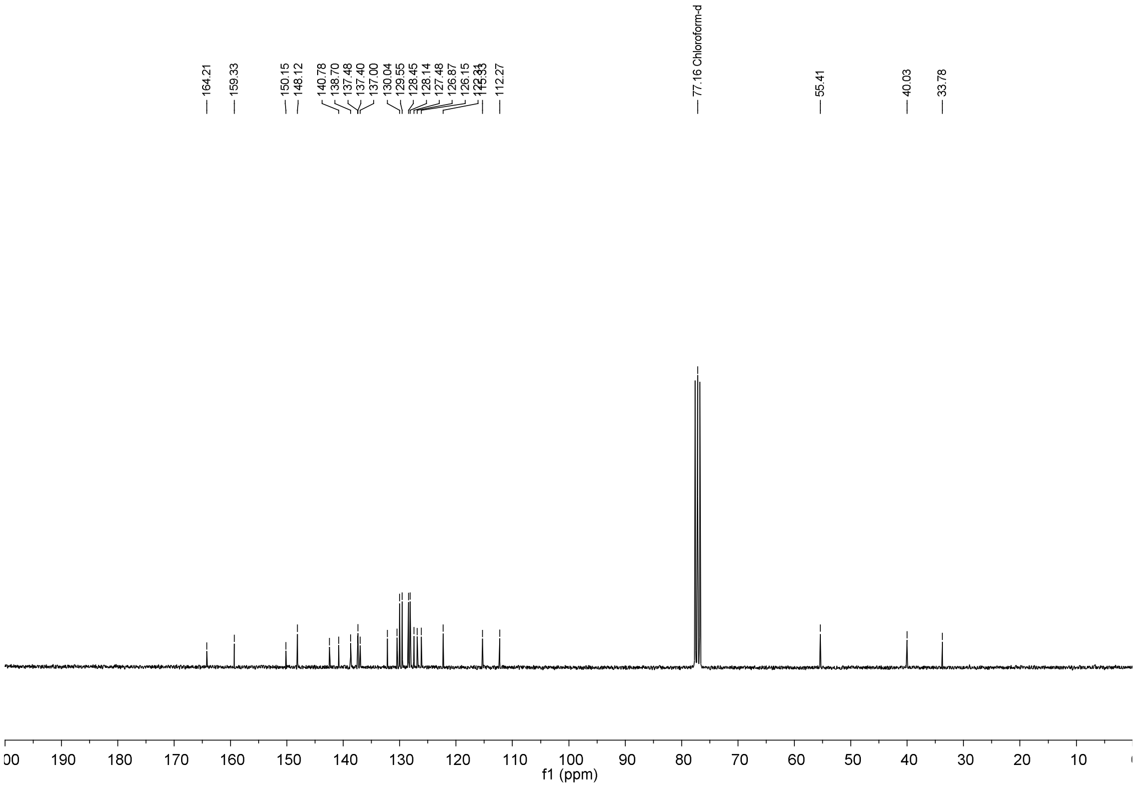

(*E*)-*N*-(2-(1,2-Diphenylvinyl)-5-methylphenethyl)picolinamide (81)

^{1}H NMR ($CDCl_3$, 300 MHz)

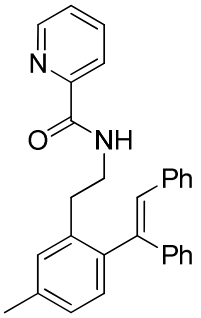

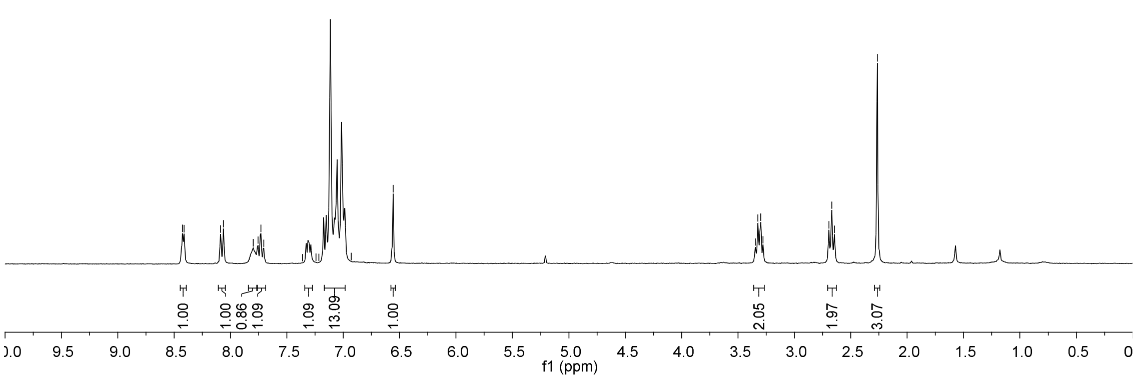

^{13}C NMR ($CDCl_3$, 75 MHz)

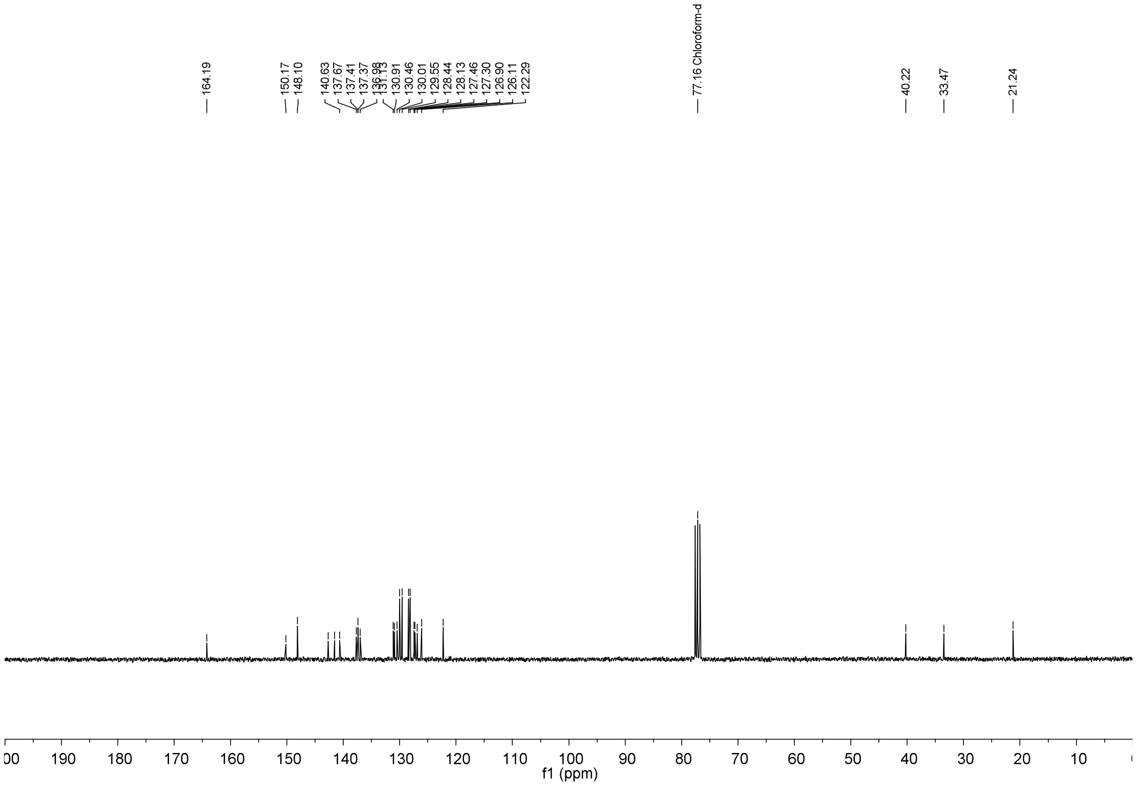

(*E*)-*N*-(2-(1,2-Diphenylvinyl)-6-methoxyphenethyl)picolinamide (82)

^{1}H NMR ($CDCl_3$, 300 MHz)

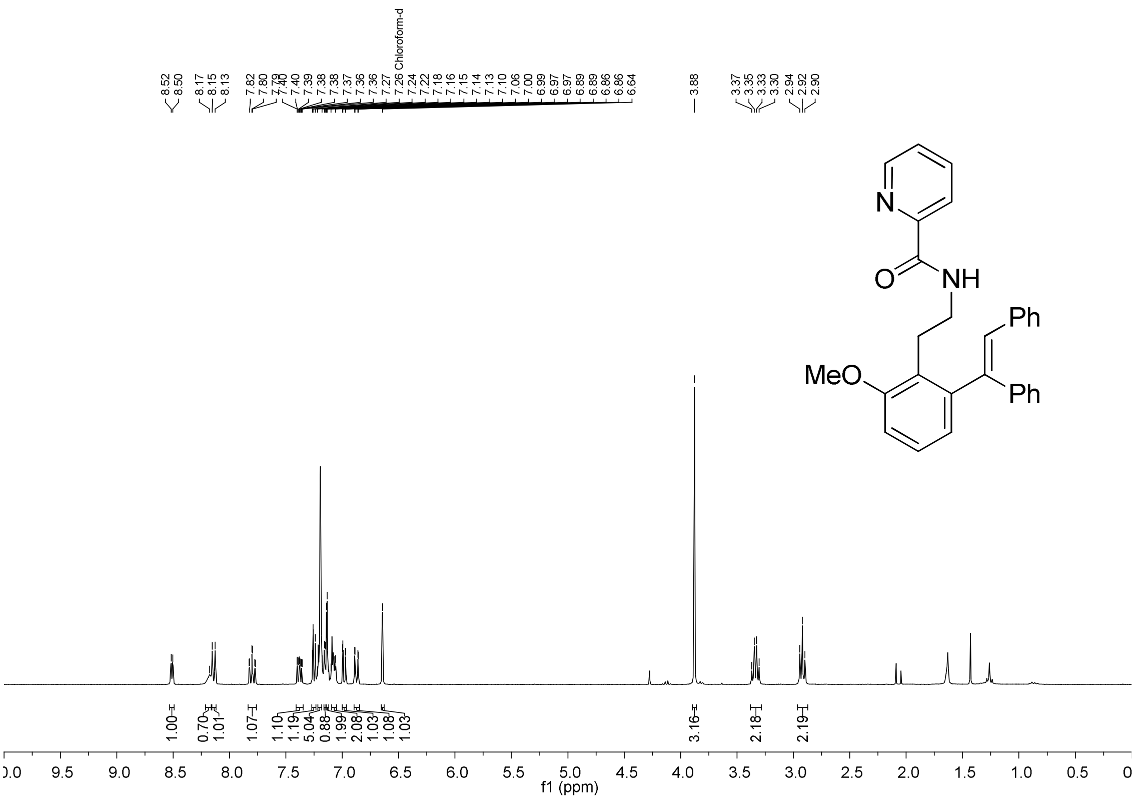

^{13}C NMR ($CDCl_3$, 75 MHz)

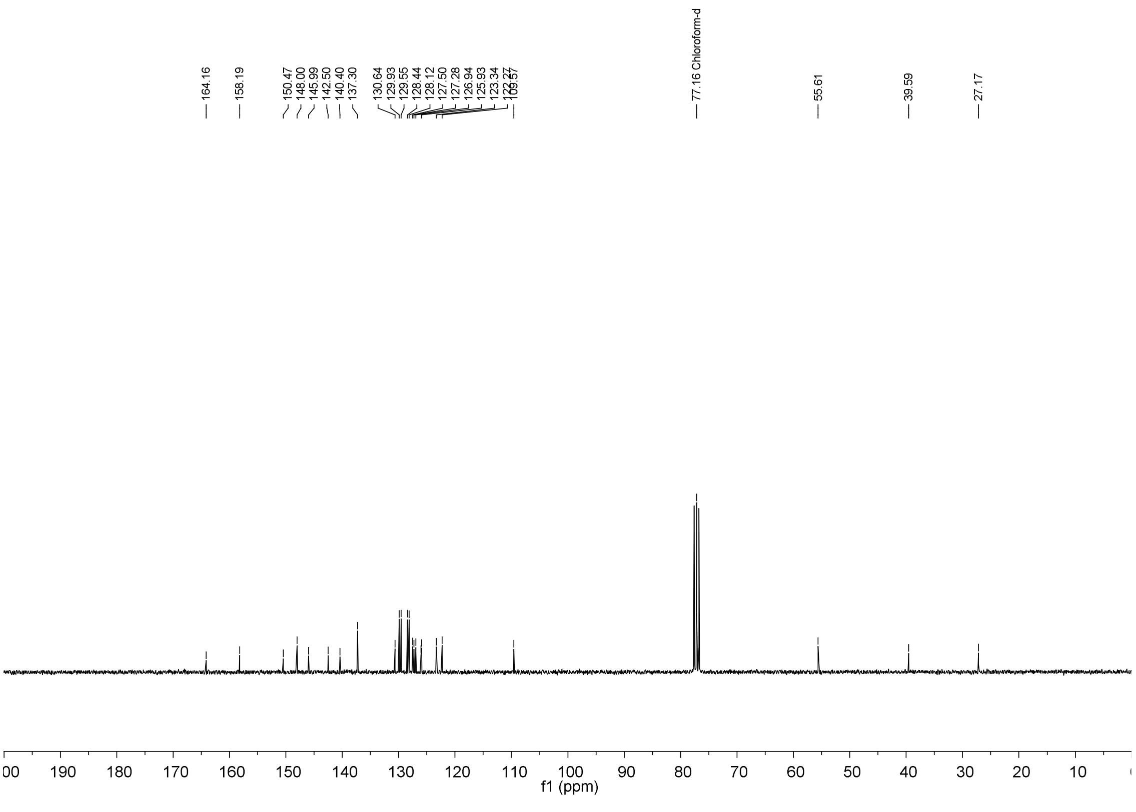

(*E*)-*N*-(2-(1,2-Diphenylvinyl)-6-methylphenethyl)picolinamide (83)

^{1}H NMR ($CDCl_3$, 300 MHz)

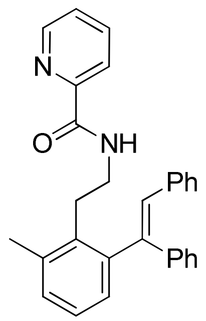

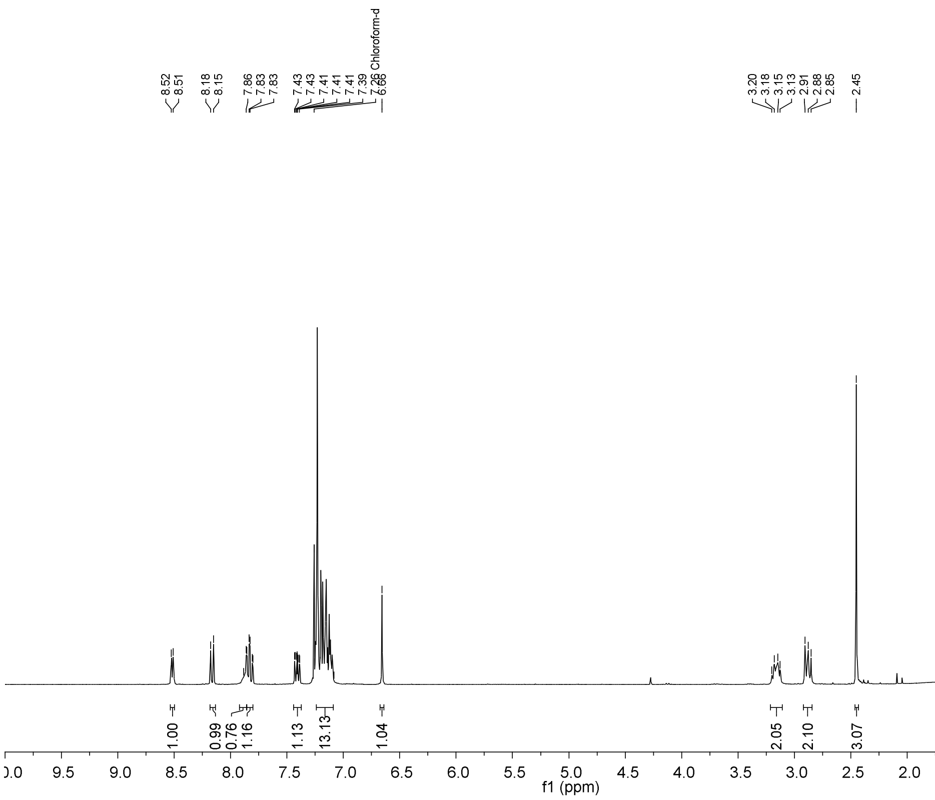

^{13}C NMR ($CDCl_3$, 75 MHz)

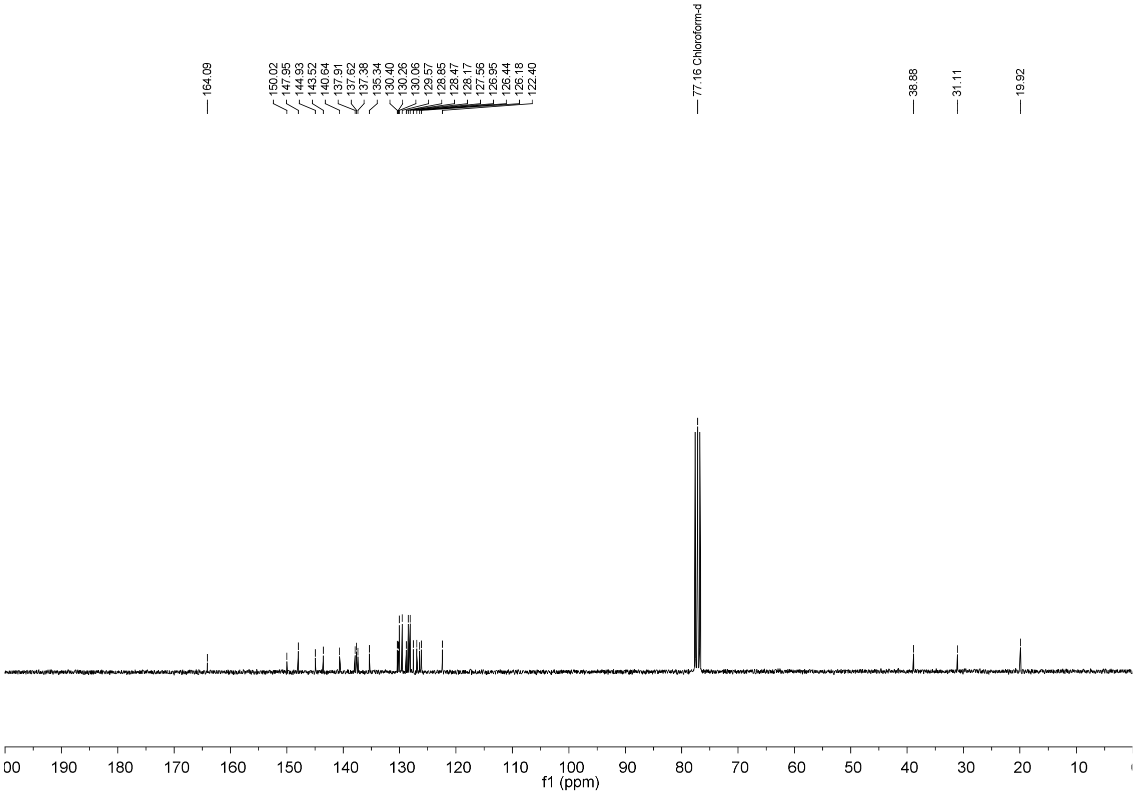

(*E*)-*N*-(2-Bromo-6-(1,2-diphenylvinyl)phenethyl)picolinamide (84)

^{1}H NMR ($CDCl_3$, 300 MHz)

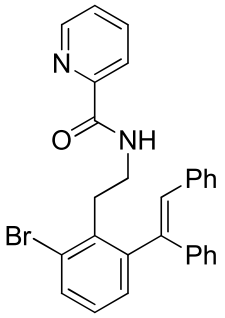

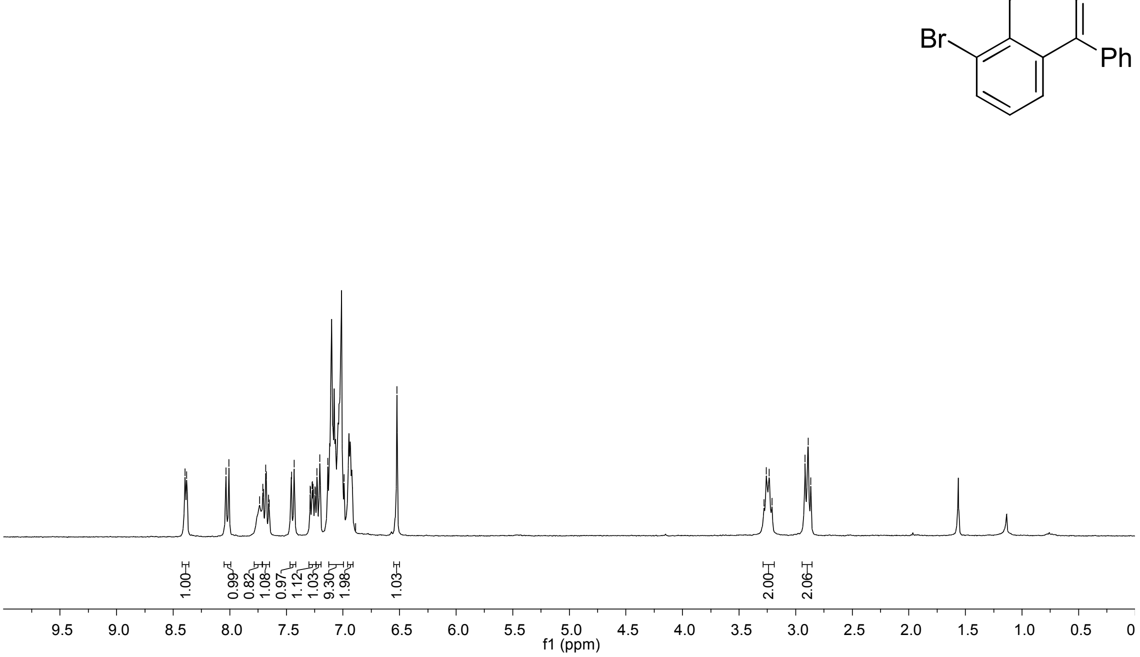

^{13}C NMR ($CDCl_3$, 75 MHz)

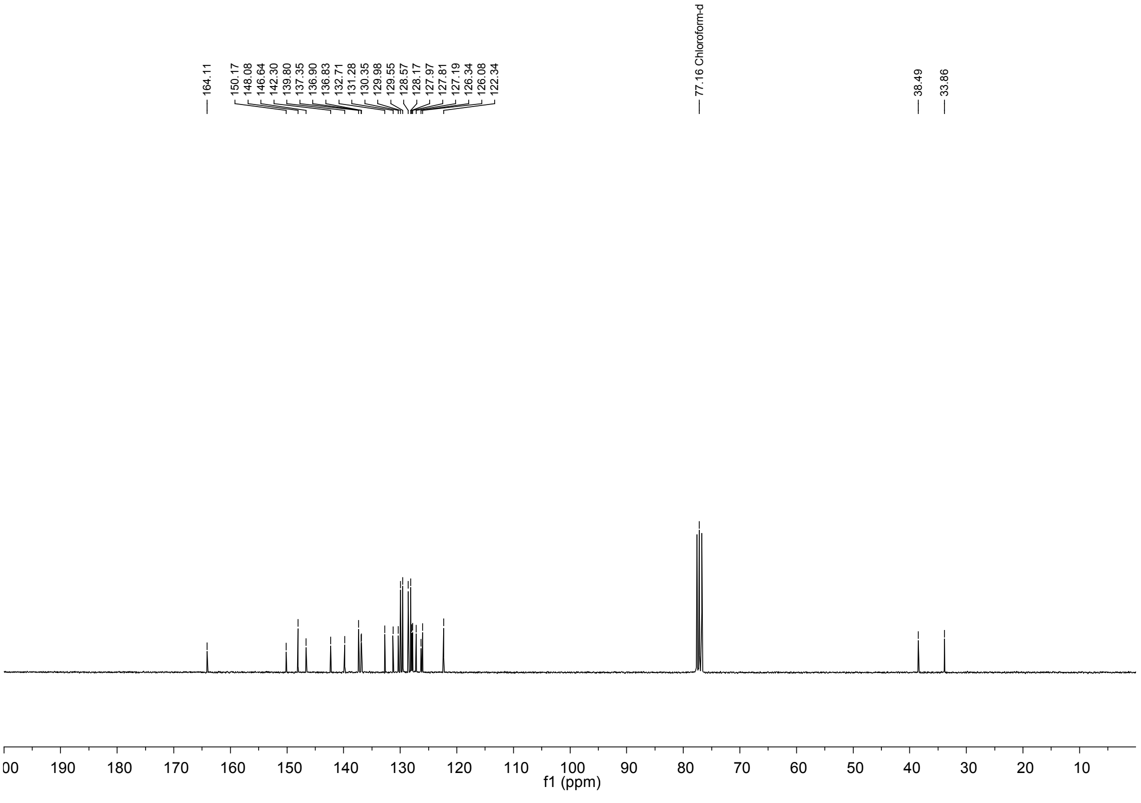

(*E*)-*N*-(2-Chloro-6-(1,2-diphenylvinyl)phenethyl)picolinamide (85)

^{1}H NMR ($CDCl_3$, 300 MHz)

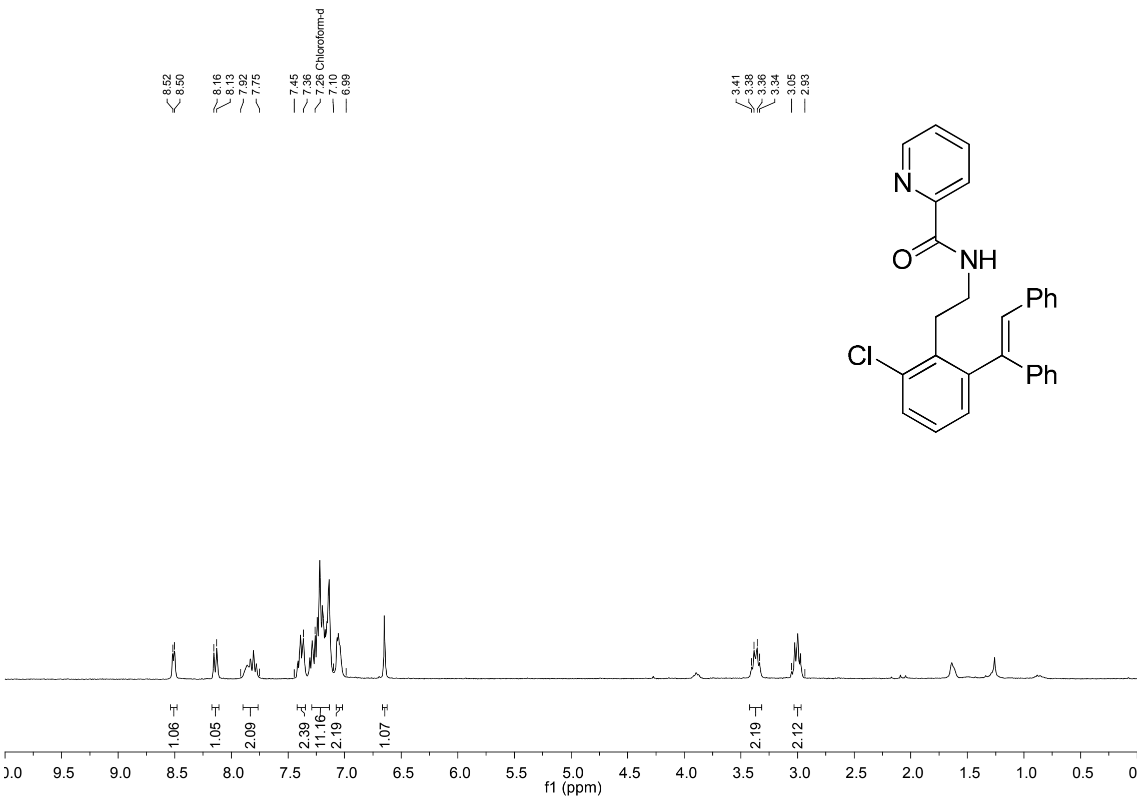

^{13}C NMR ($CDCl_3$, 75 MHz)

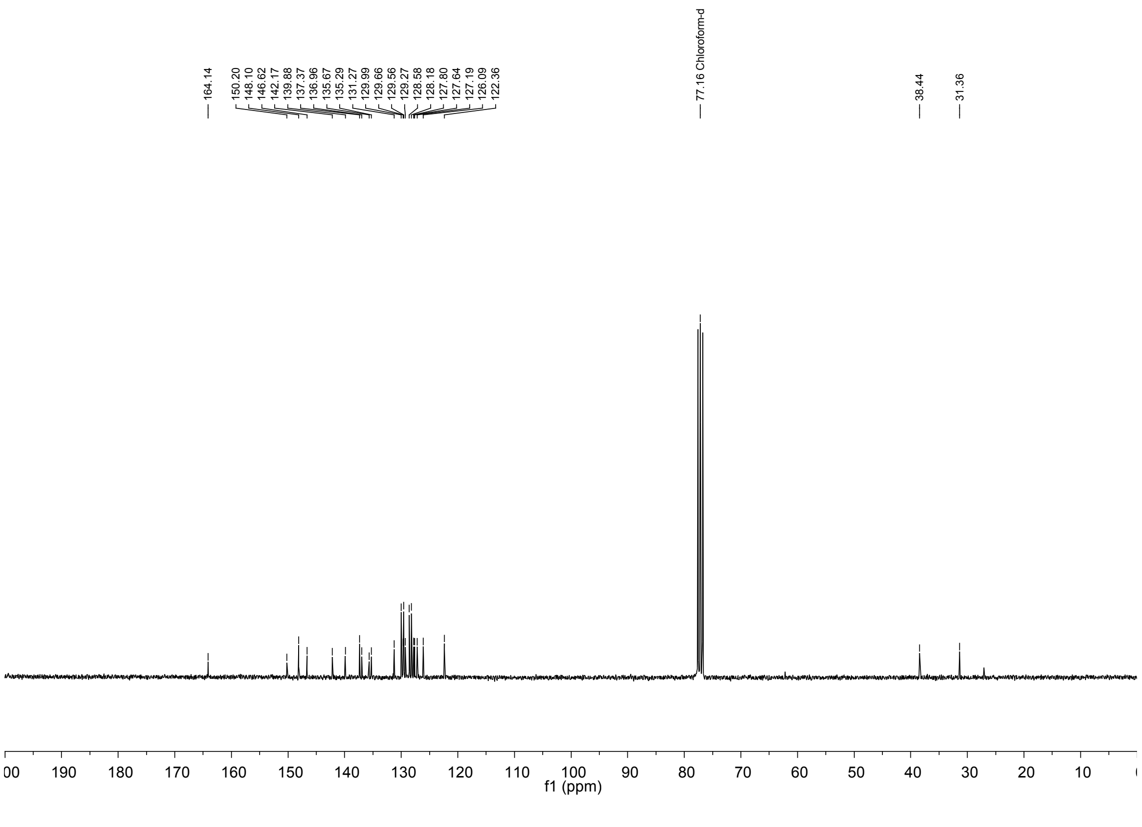

(*E*)-*N*-(2-(3-(1,2-Diphenylvinyl)naphthalen-2-yl)ethyl)picolinamide (86)

^{1}H NMR ($CDCl_3$, 300 MHz)

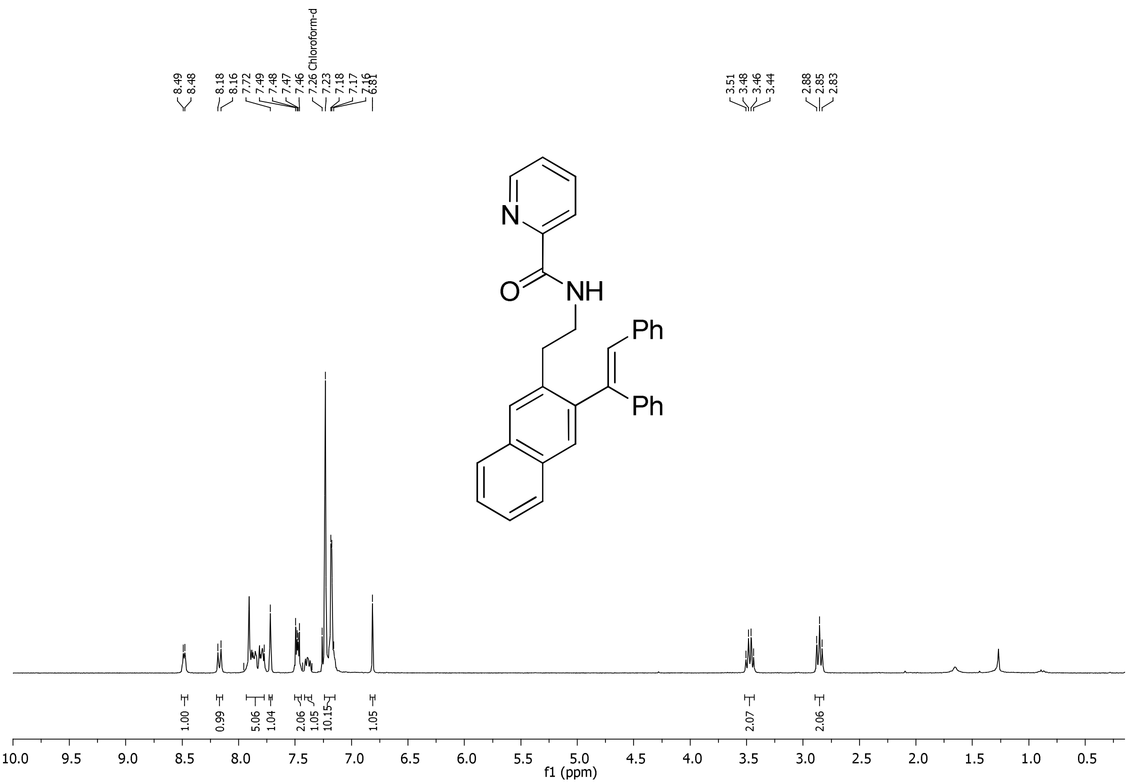

^{13}C NMR ($CDCl_3$, 75 MHz)

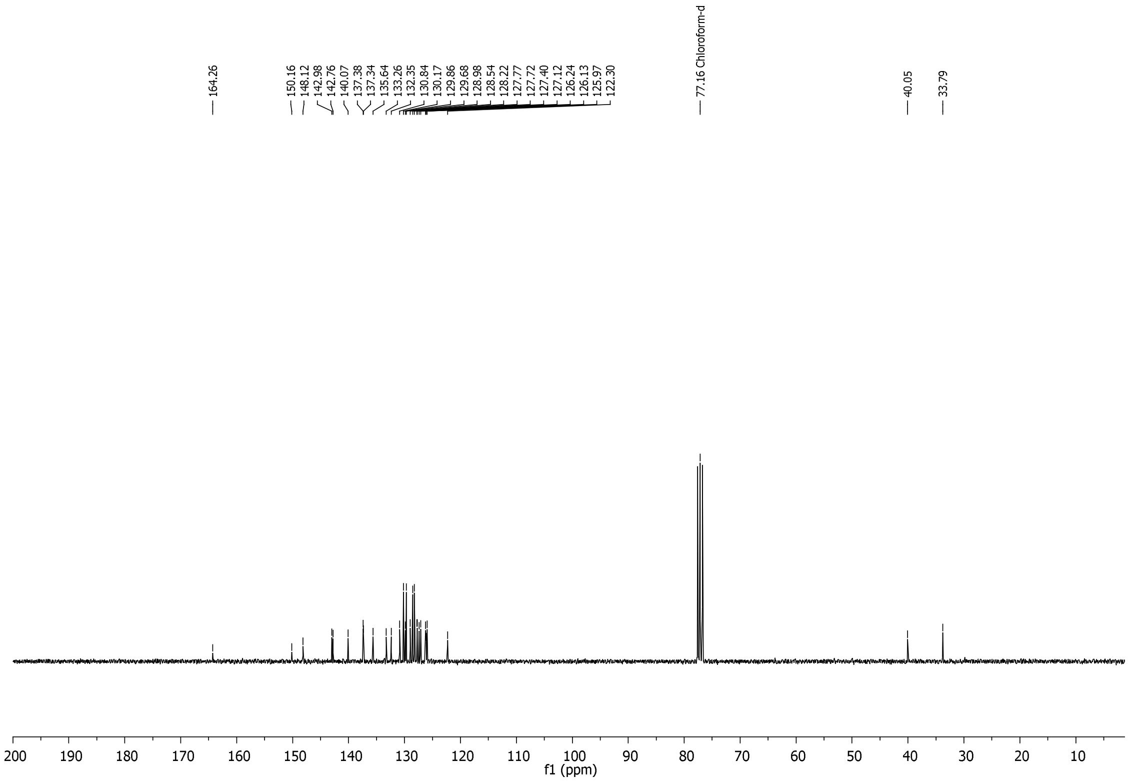

(*E*)-*N*-(2-(3-(1,2-Diphenylvinyl)thiophen-2-yl)ethyl)picolinamide (87)

^{1}H NMR ($CDCl_3$, 300 MHz)

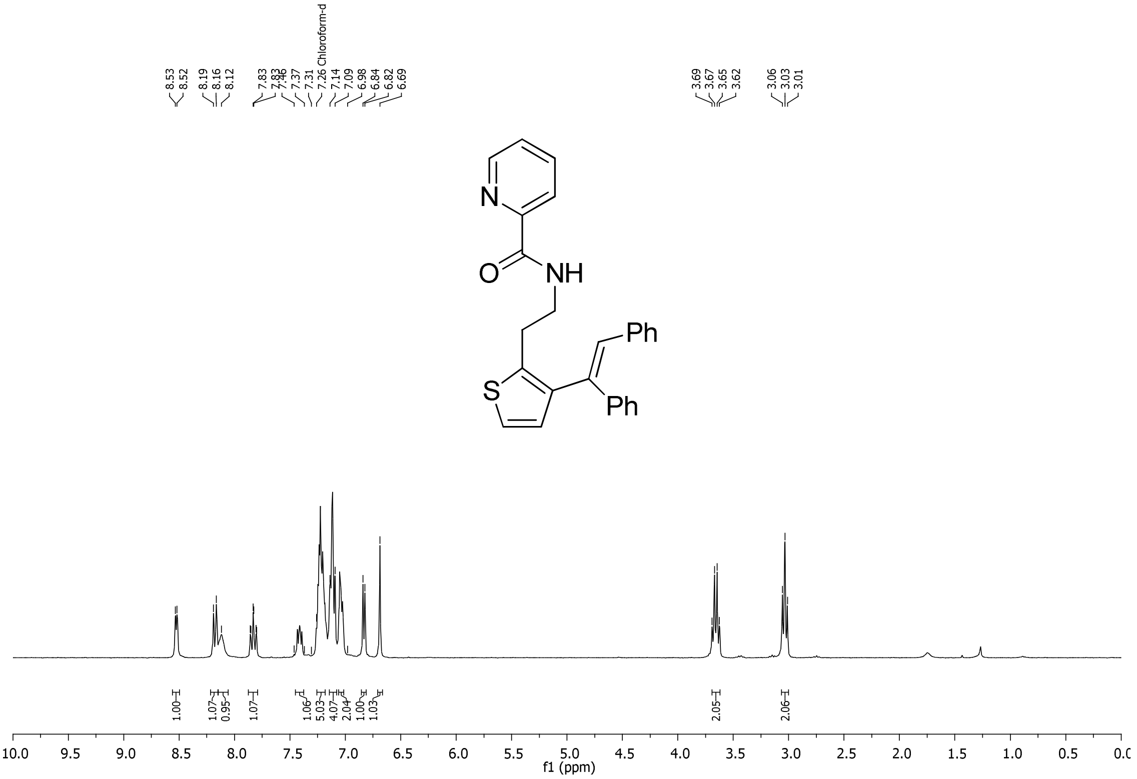

^{13}C NMR ($CDCl_3$, 75 MHz)

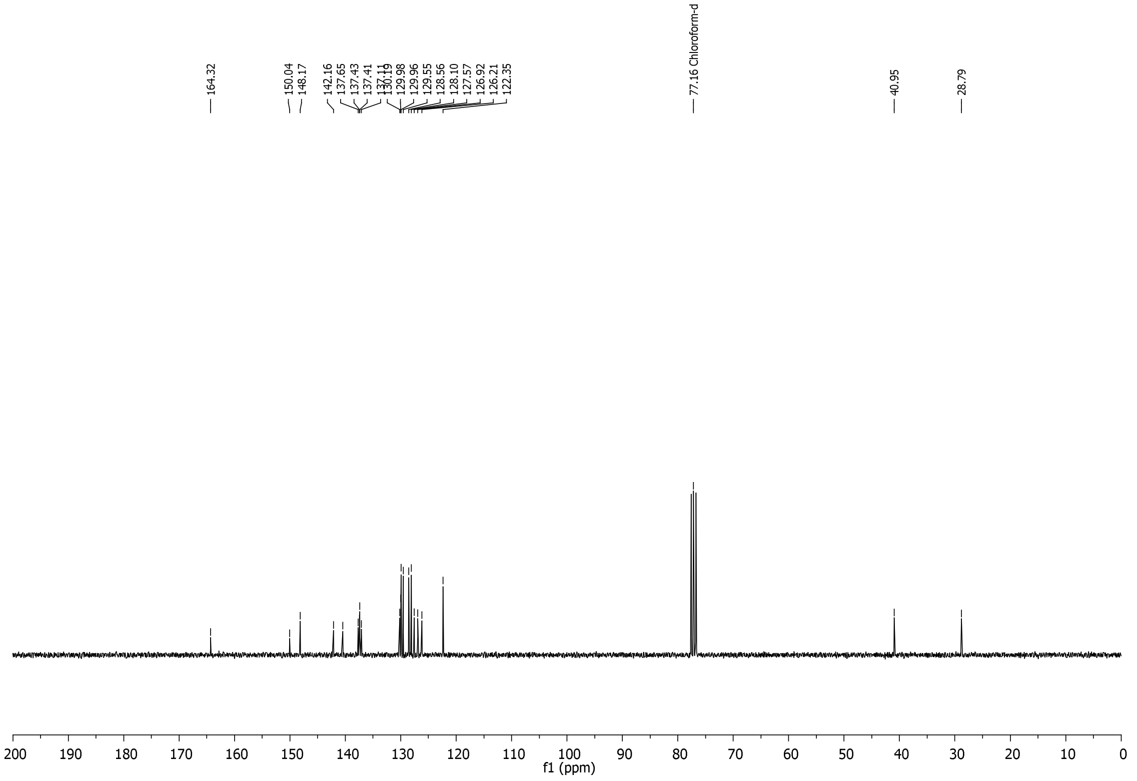

_N_-(2,6-Bis((_E_)-1,2-bis(4-methoxyphenyl)vinyl)phenethyl)picolinamide (74)

^{1}H NMR ($CDCl_3$, 300 MHz)

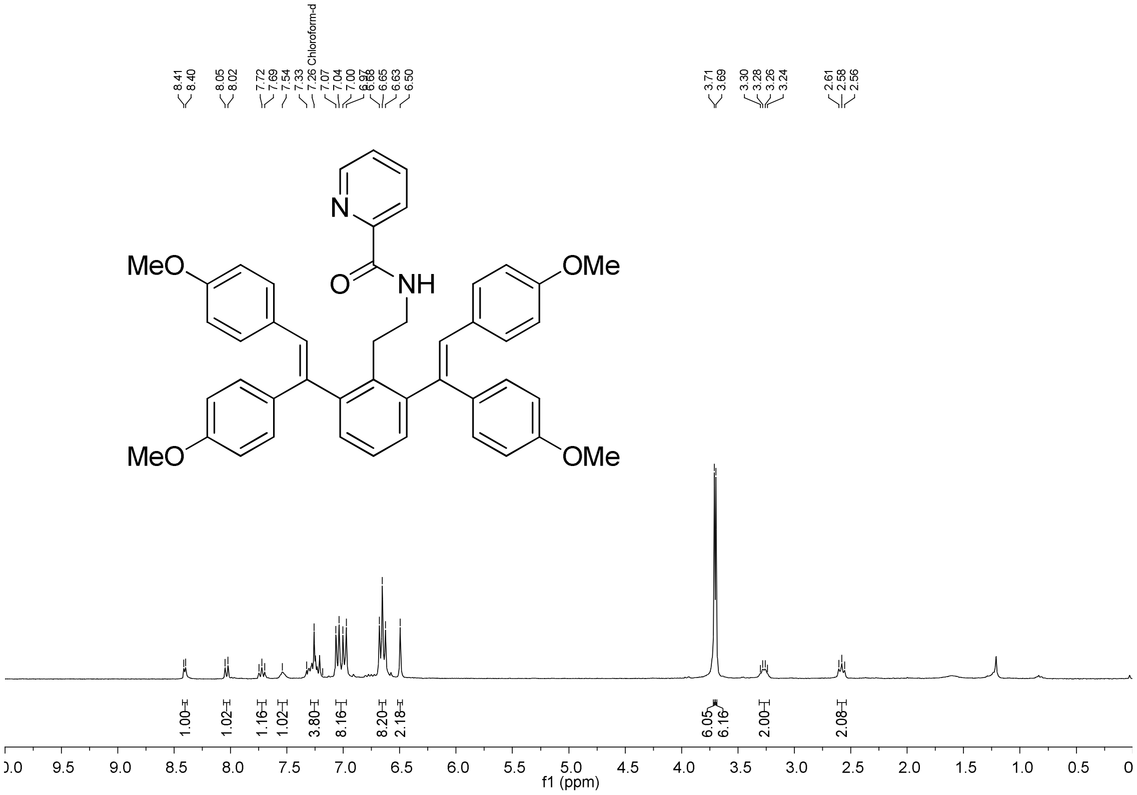

^{13}C NMR ($CDCl_3$, 75 MHz)

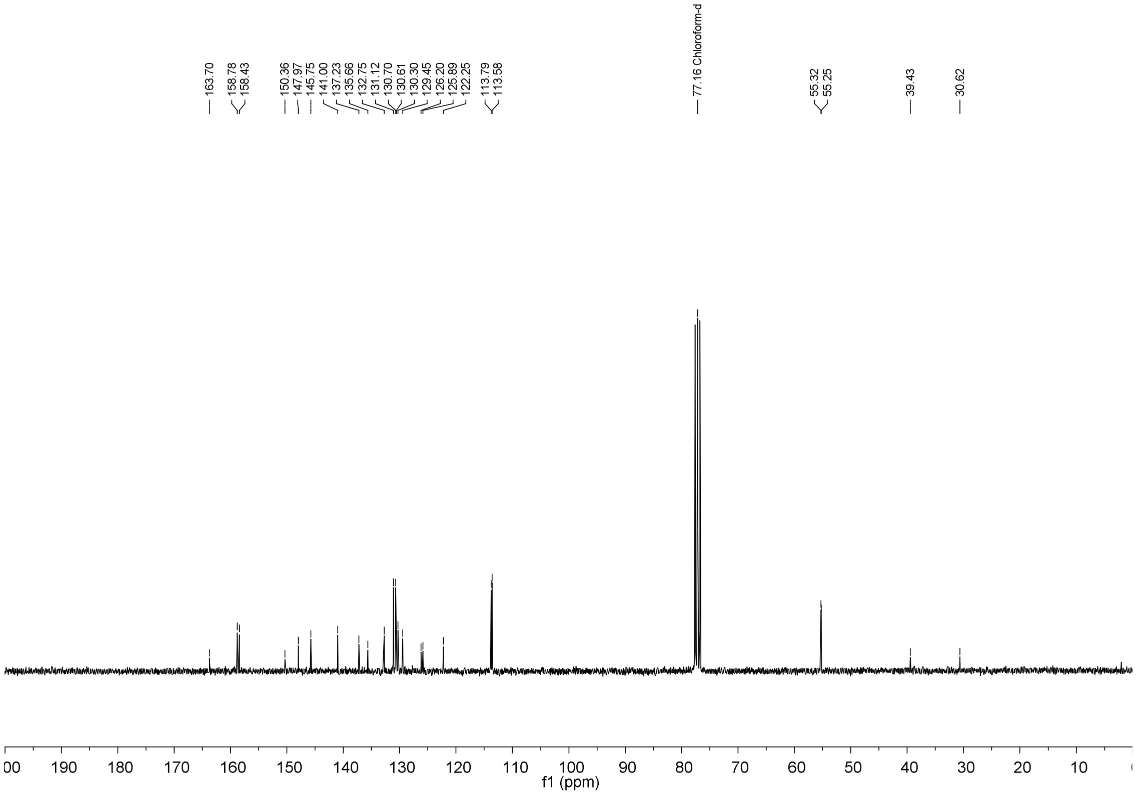

_N_-(2,6-Bis((_E_)-1,2-di-_p_-tolylvinyl)phenethyl)picolinamide (75)

^{1}H NMR ($CDCl_3$, 300 MHz)

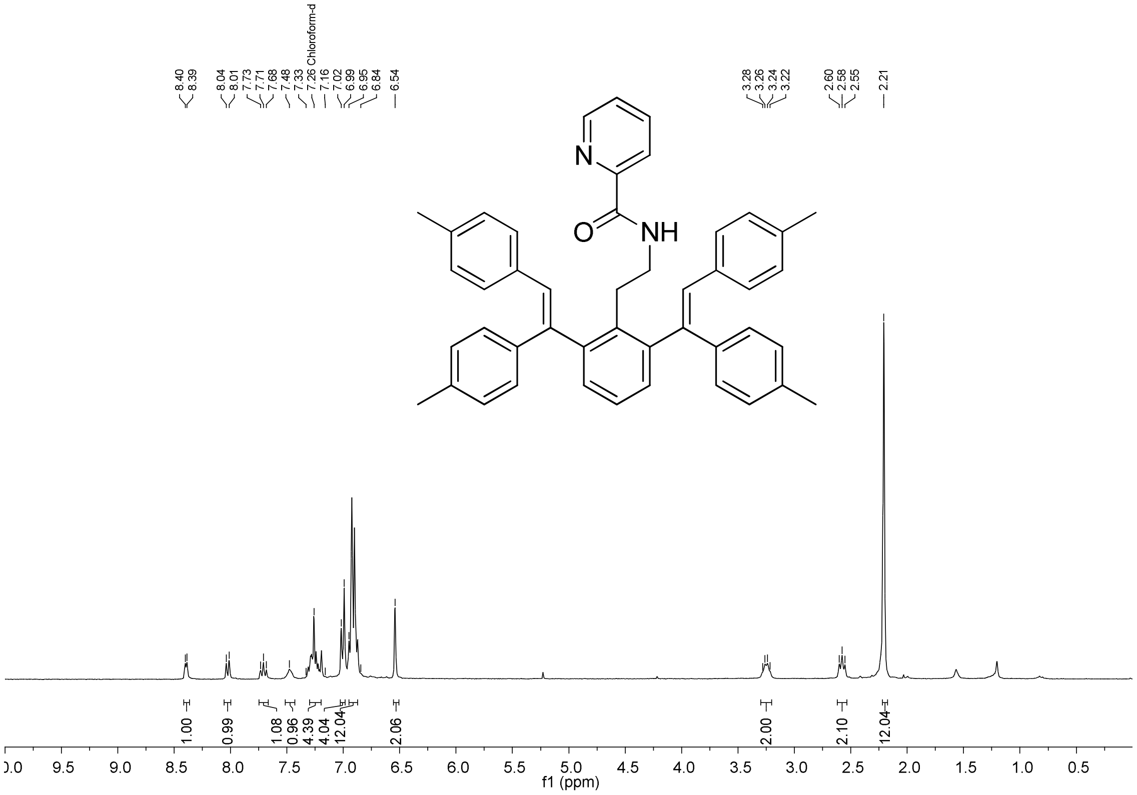

^{13}C NMR ($CDCl_3$, 75 MHz)

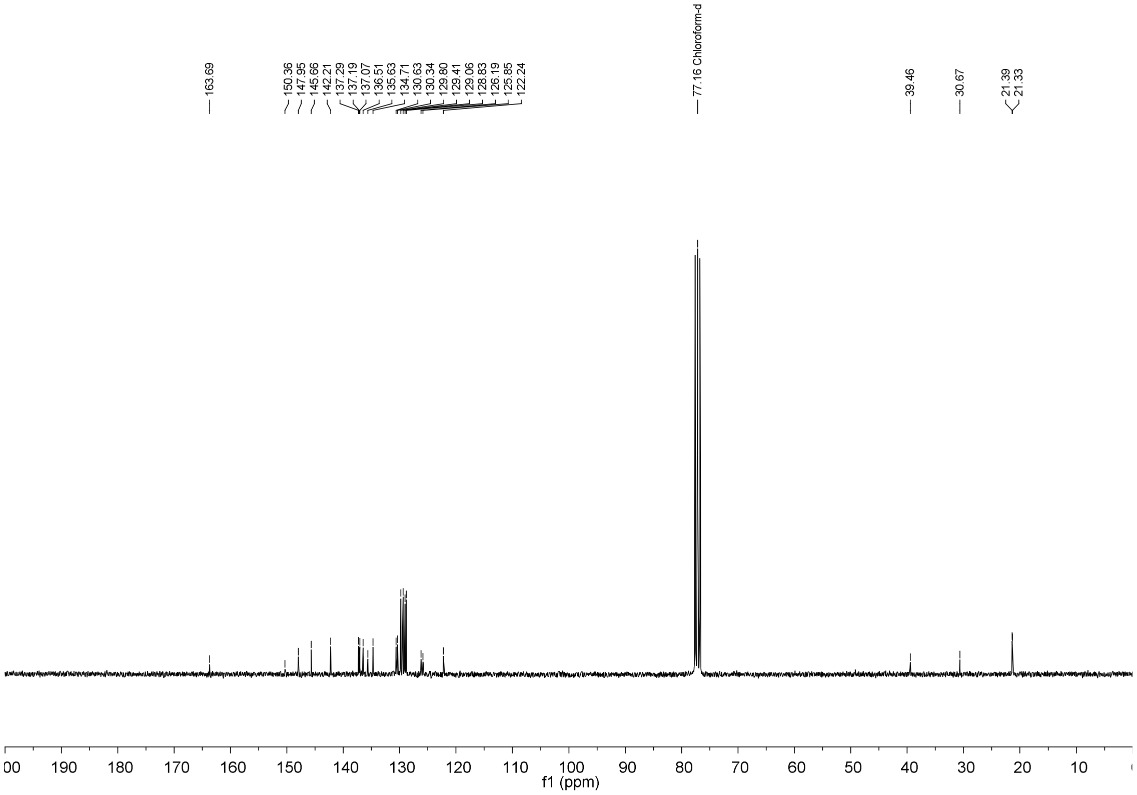

_N_-(2,6-Bis((_E_)-1,2-bis(4-(trifluoromethyl)phenyl)vinyl)phenethyl)picolinamide (76)

^{1}H NMR ($CDCl_3$, 300 MHz)

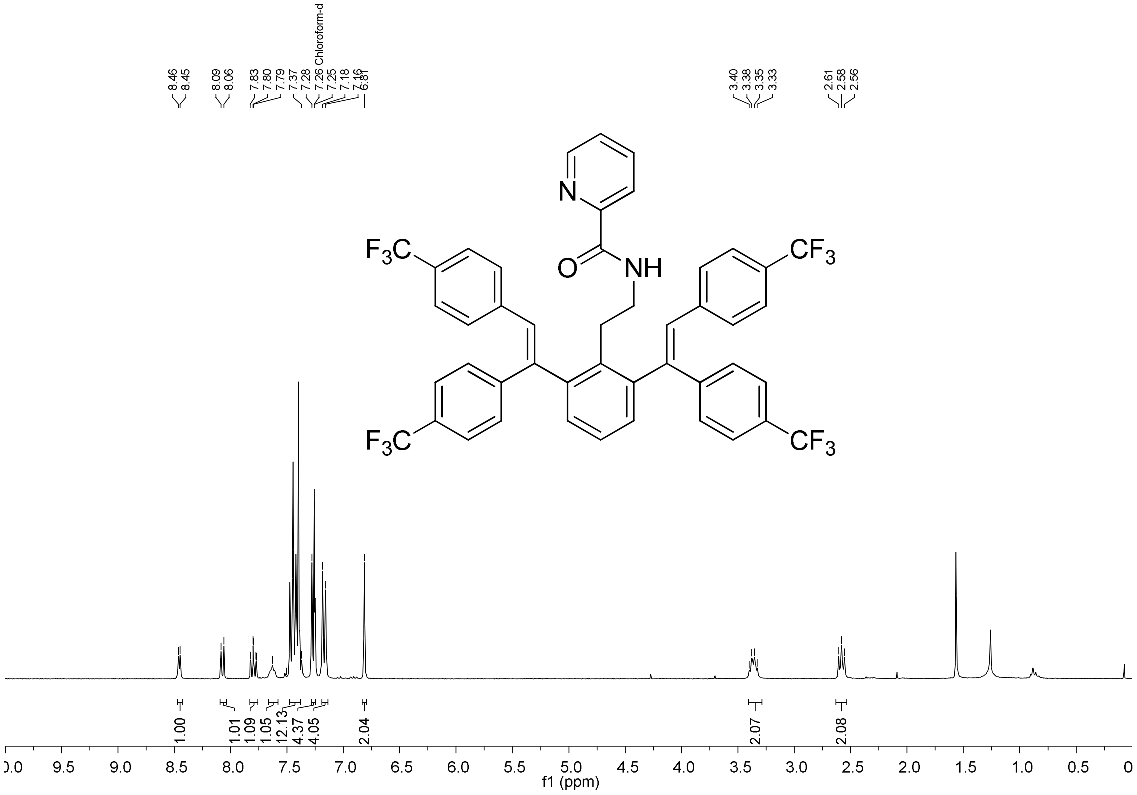

^{13}C NMR ($CDCl_3$, 75 MHz)

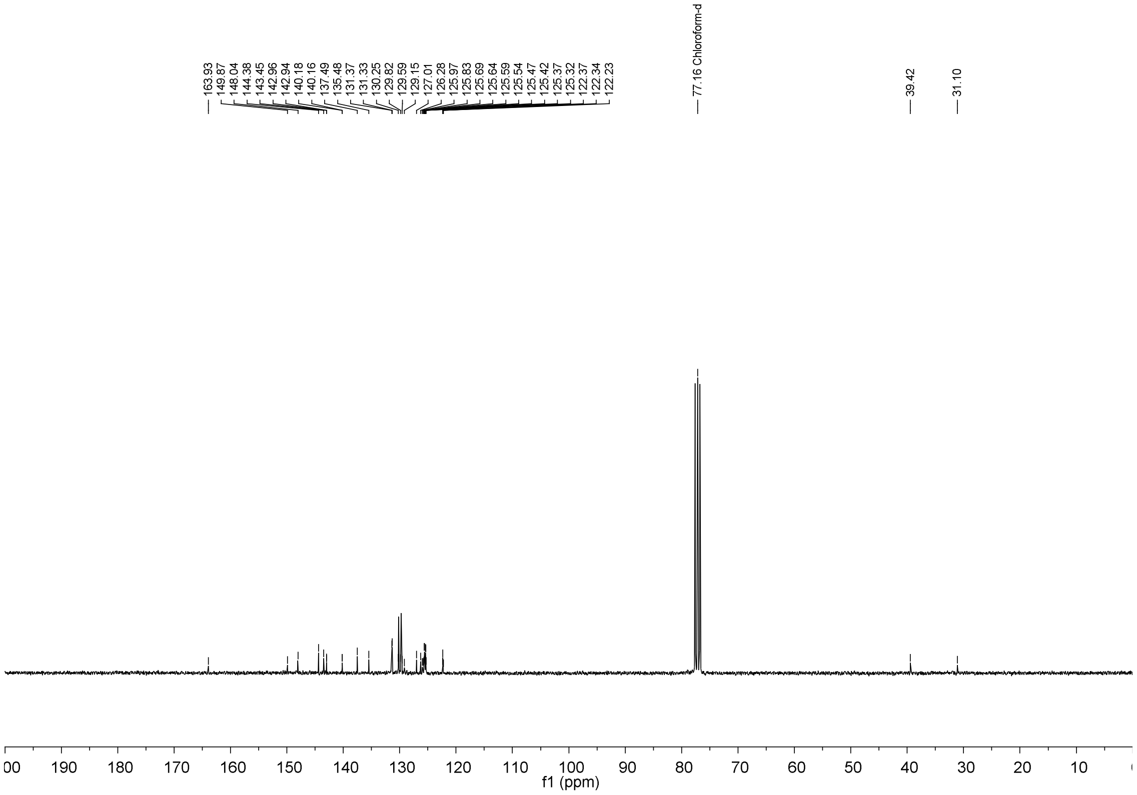

5,6,7,8-Tetraphenylisoquinoline-1-carboxamide (89)

^{1}H NMR (acetone-d_6, 300 MHz)

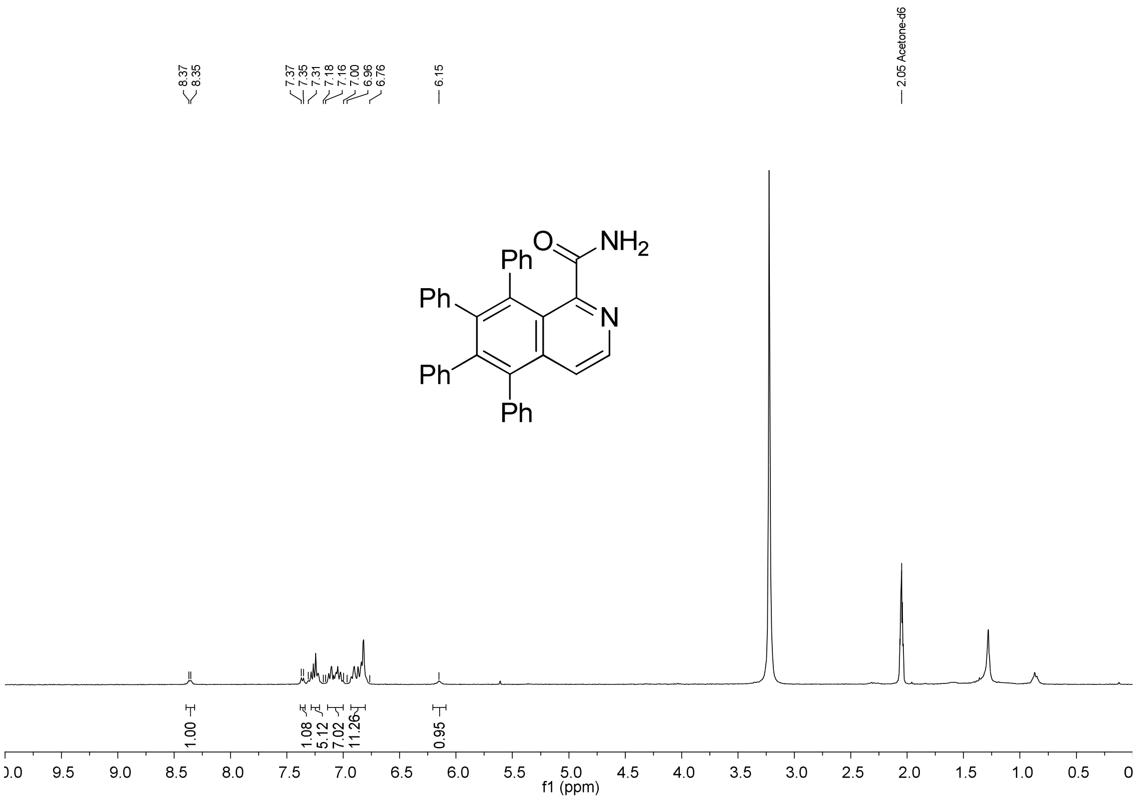

^{13}C NMR (acetone-d_6, 75 MHz)

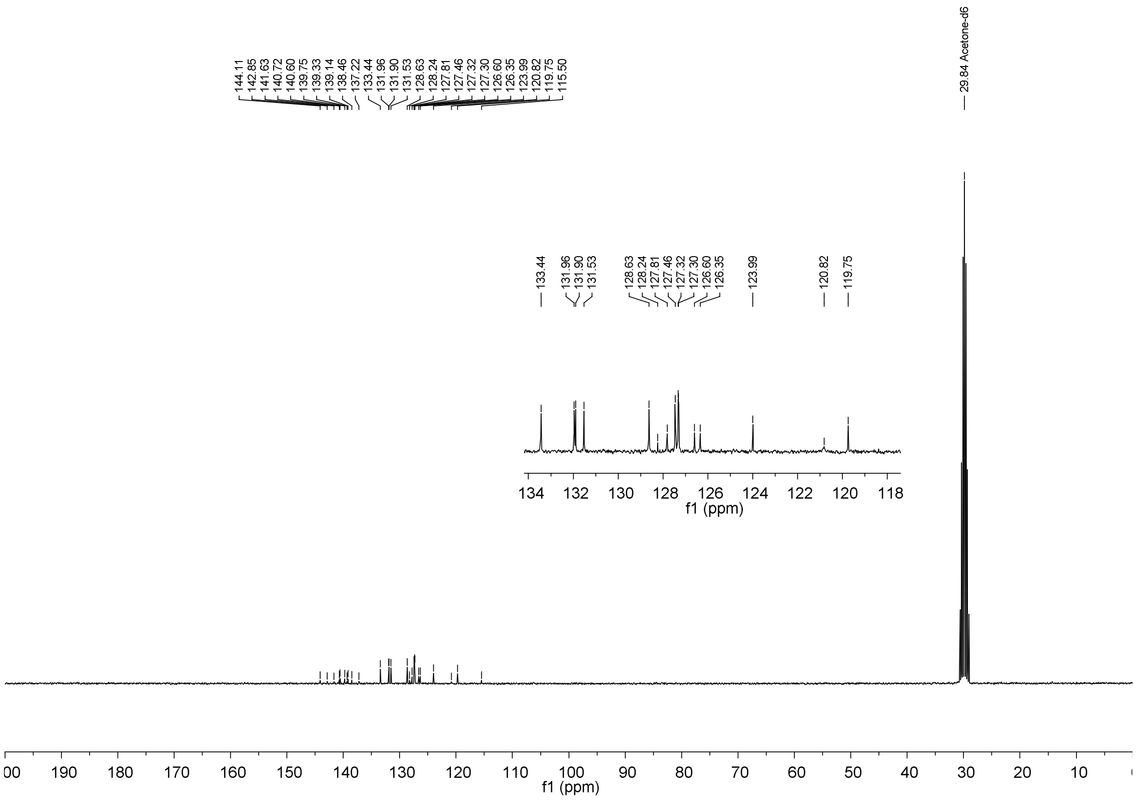

(2,6-Bis((*E*)-1,2-diphenylvinyl)phenyl)methanamine (90)

^{1}H NMR (acetone-d_6, 300 MHz)

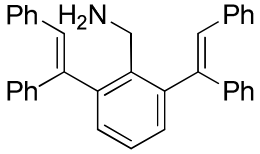

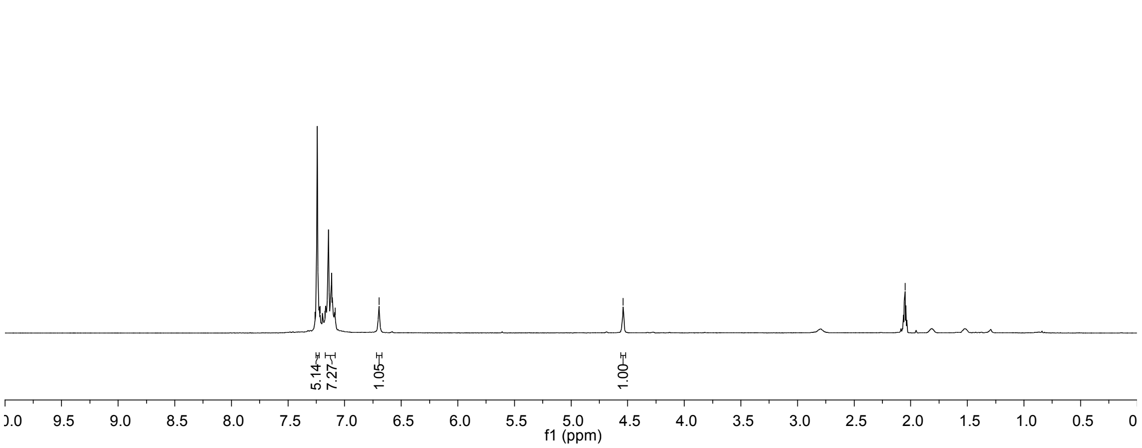

^{13}C NMR (acetone-d_6, 75 MHz)

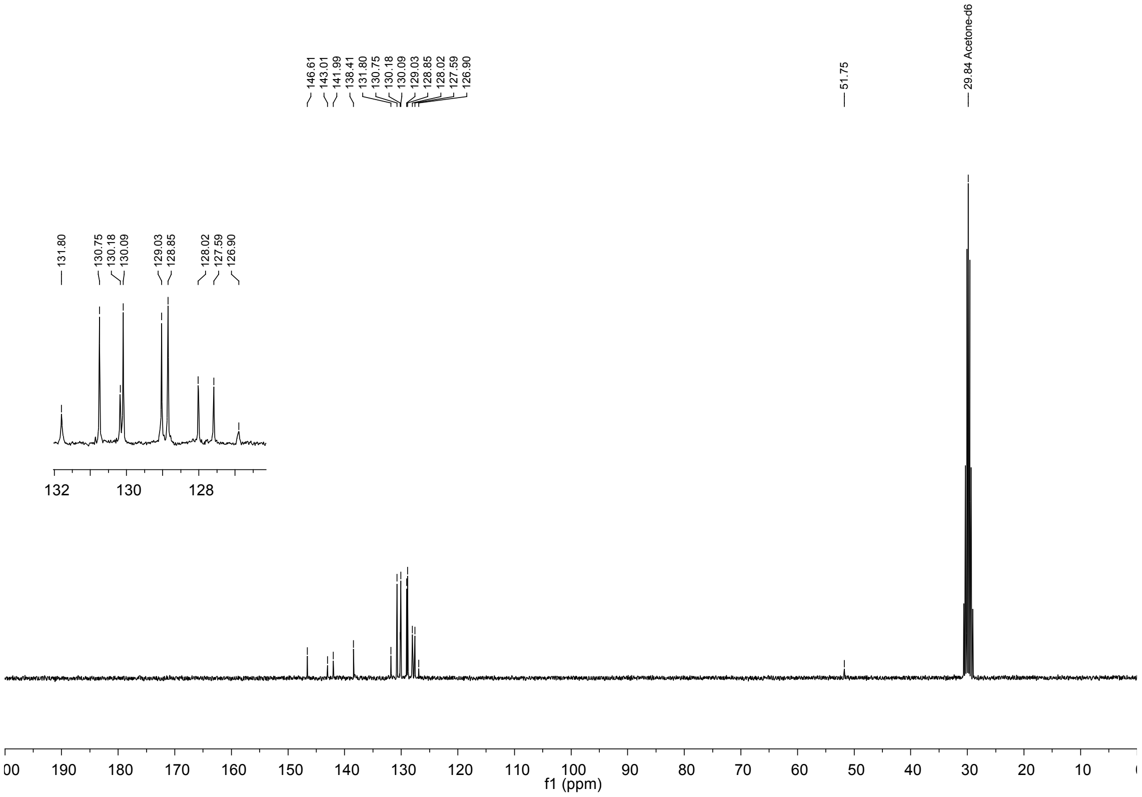

2-(2,6-Bis((*E*)-1,2-diphenylvinyl)phenyl)ethanamine (91)

^{1}H NMR (acetone-d_6, 300 MHz)

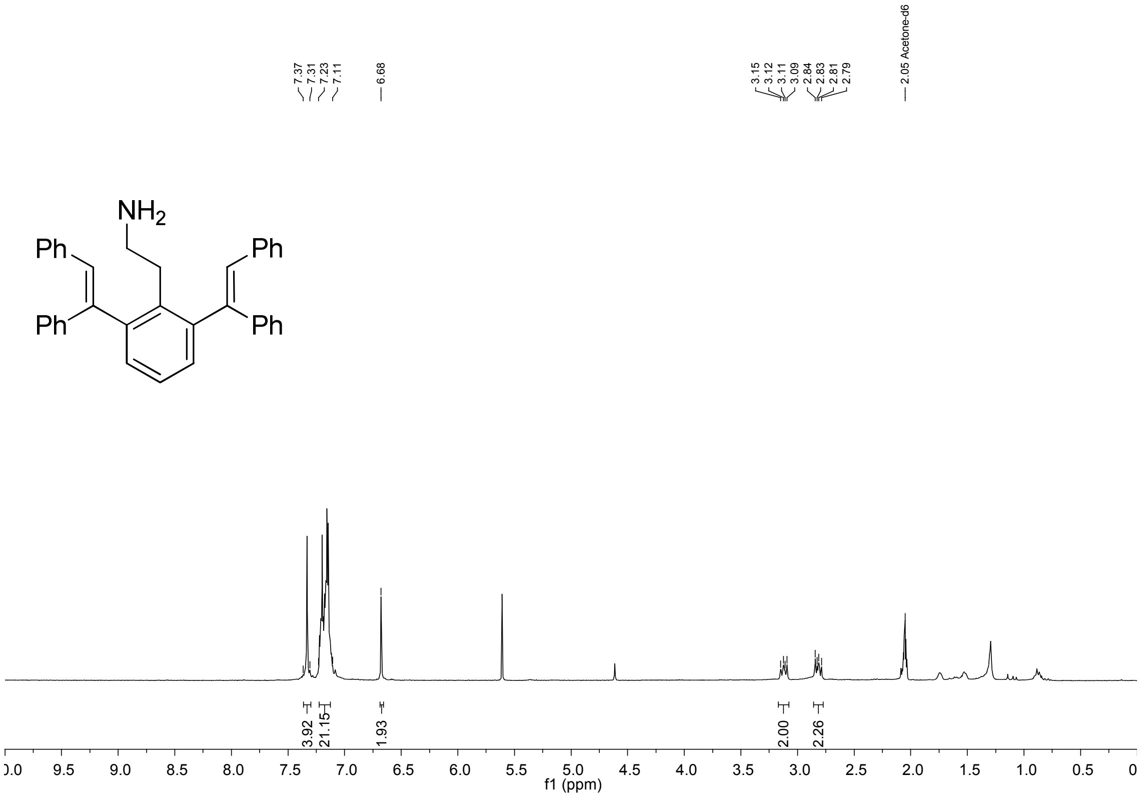

^{13}C NMR (acetone-d_6, 75 MHz)

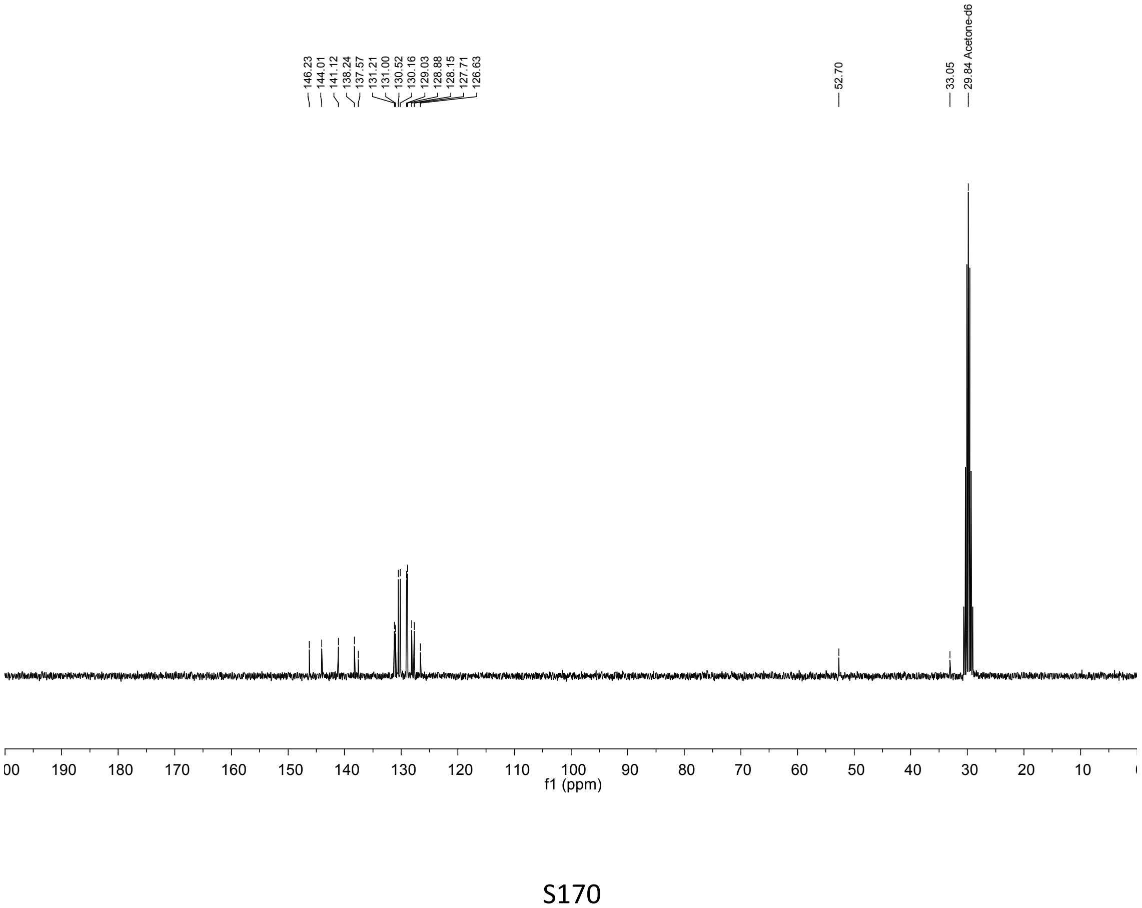

Rh^{III}-complex A

^{1}H NMR (CDCl$_3$, 300 MHz)

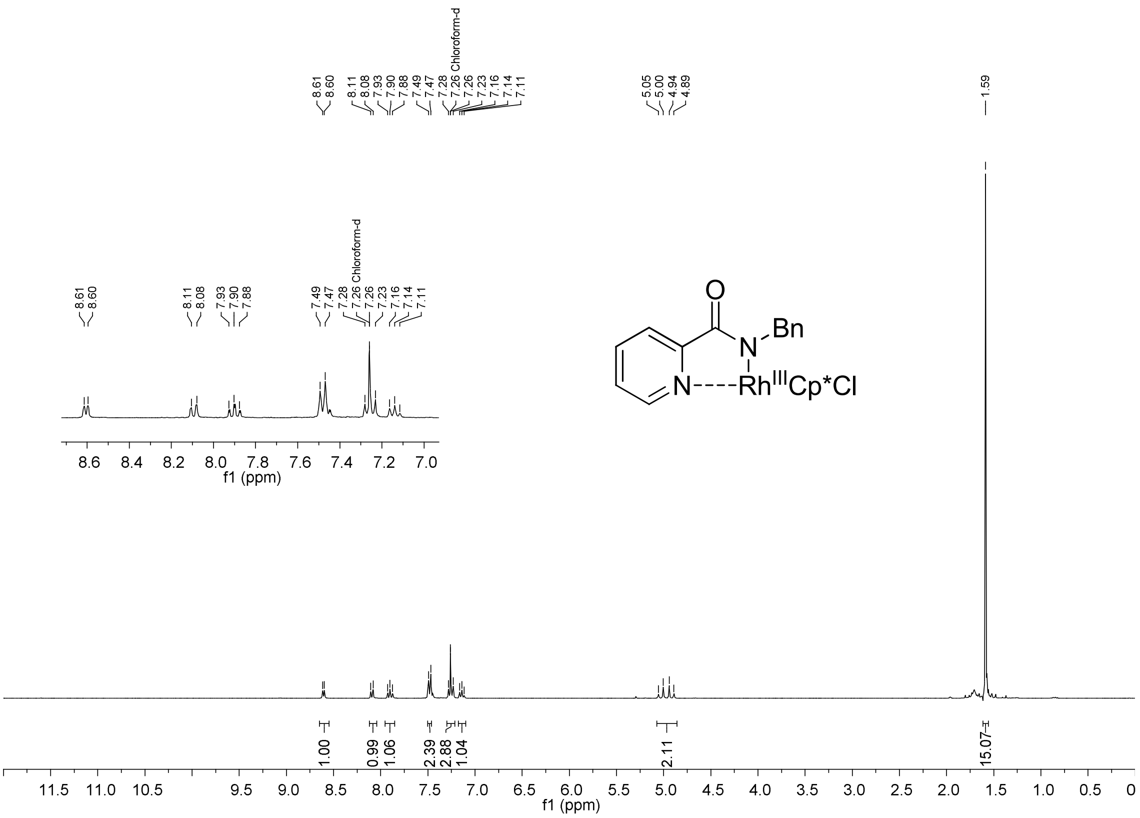

^{13}C NMR (acetone-d$_6$, 126 MHz)

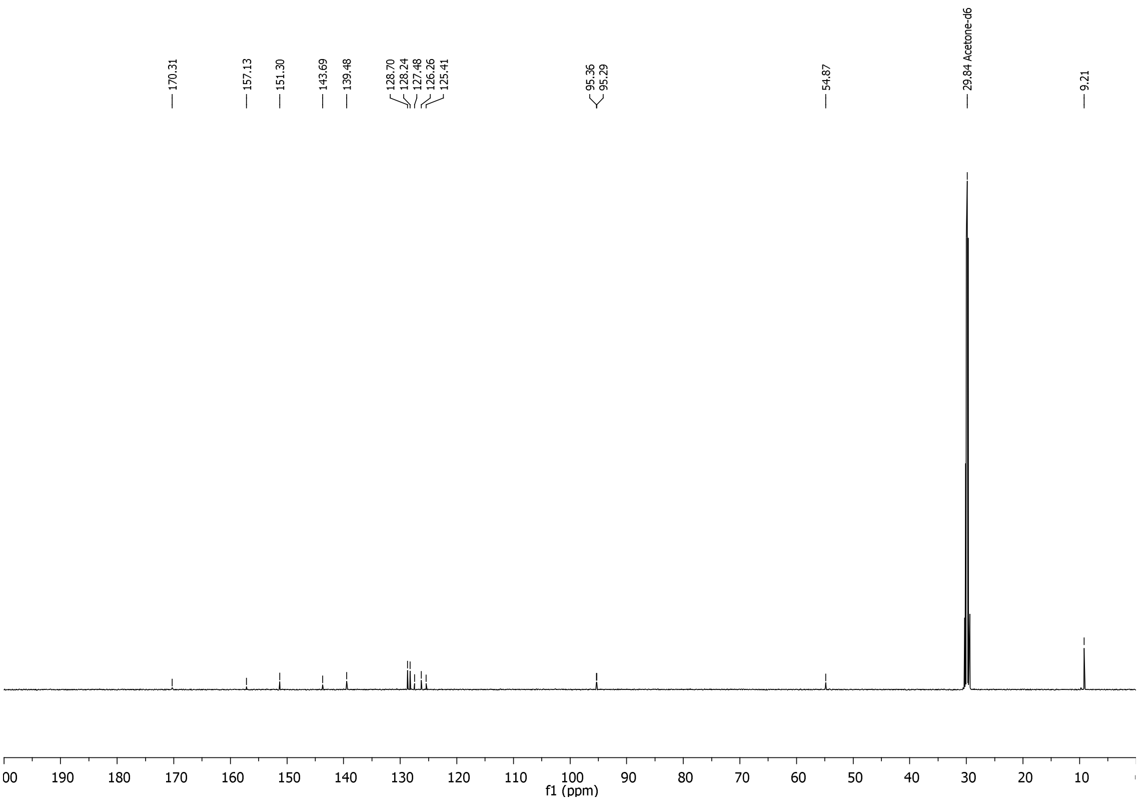

HSQC ($CDCl_3$, 500 MHz)

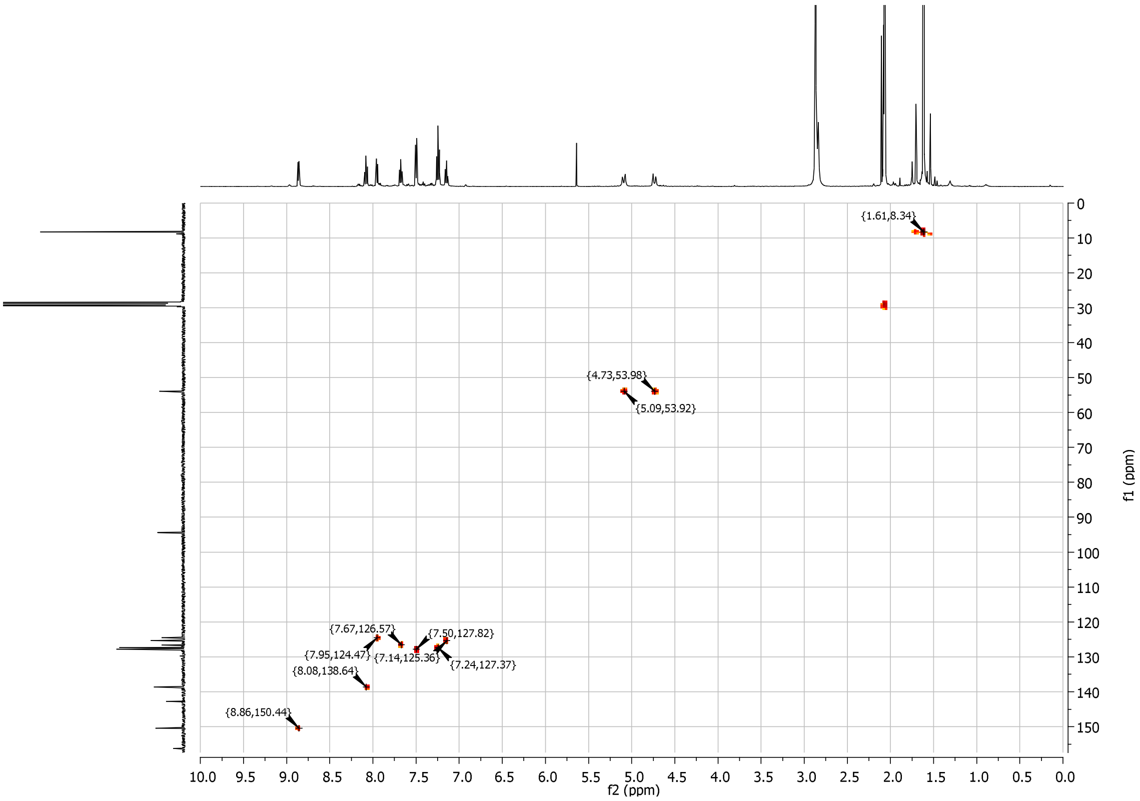

HMBC (acetone-d_6, 75 MHz)

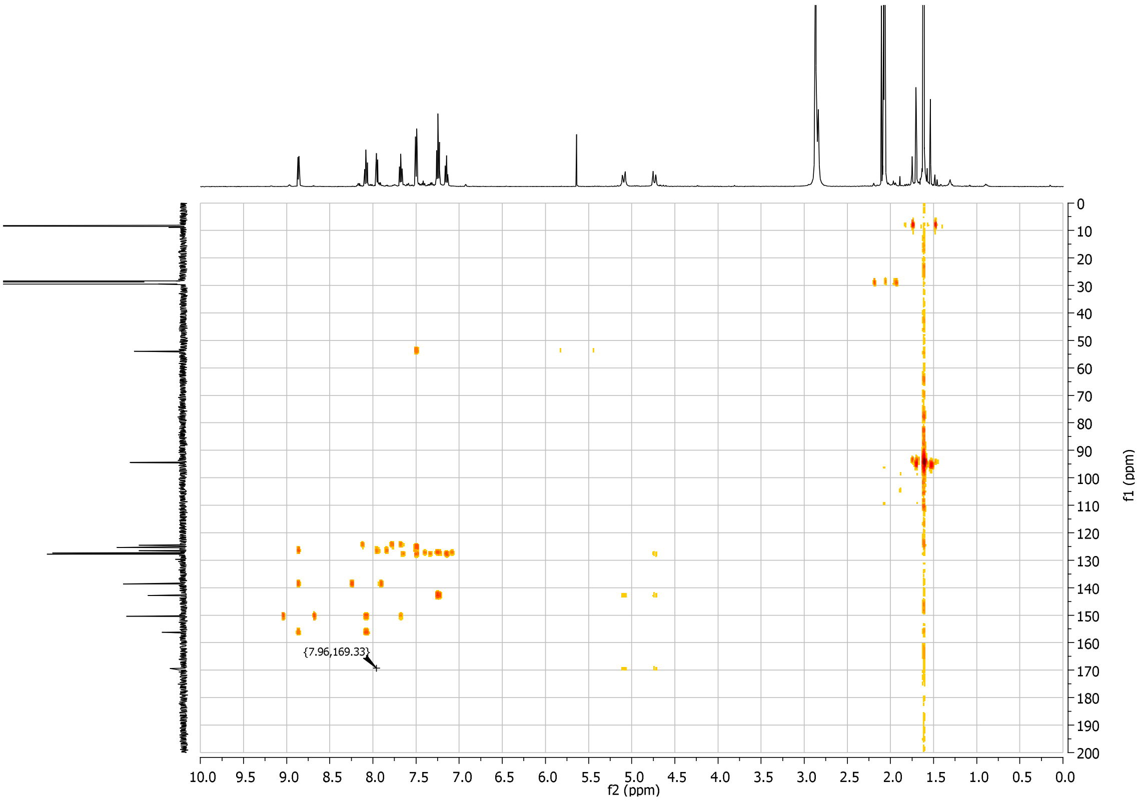

Rh^{I}-complex B

^{1}H NMR (acetone-d$_6$, 300 MHz)

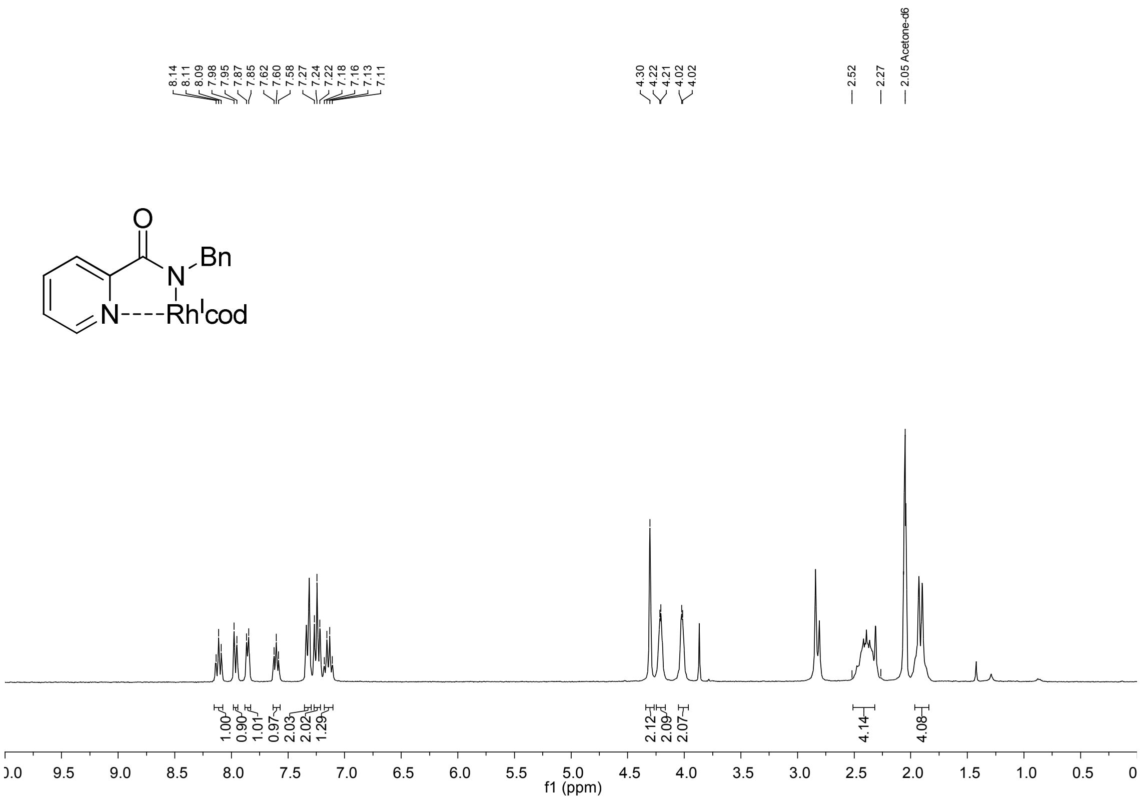

^{13}C NMR (acetone-d$_6$, 125 MHz)

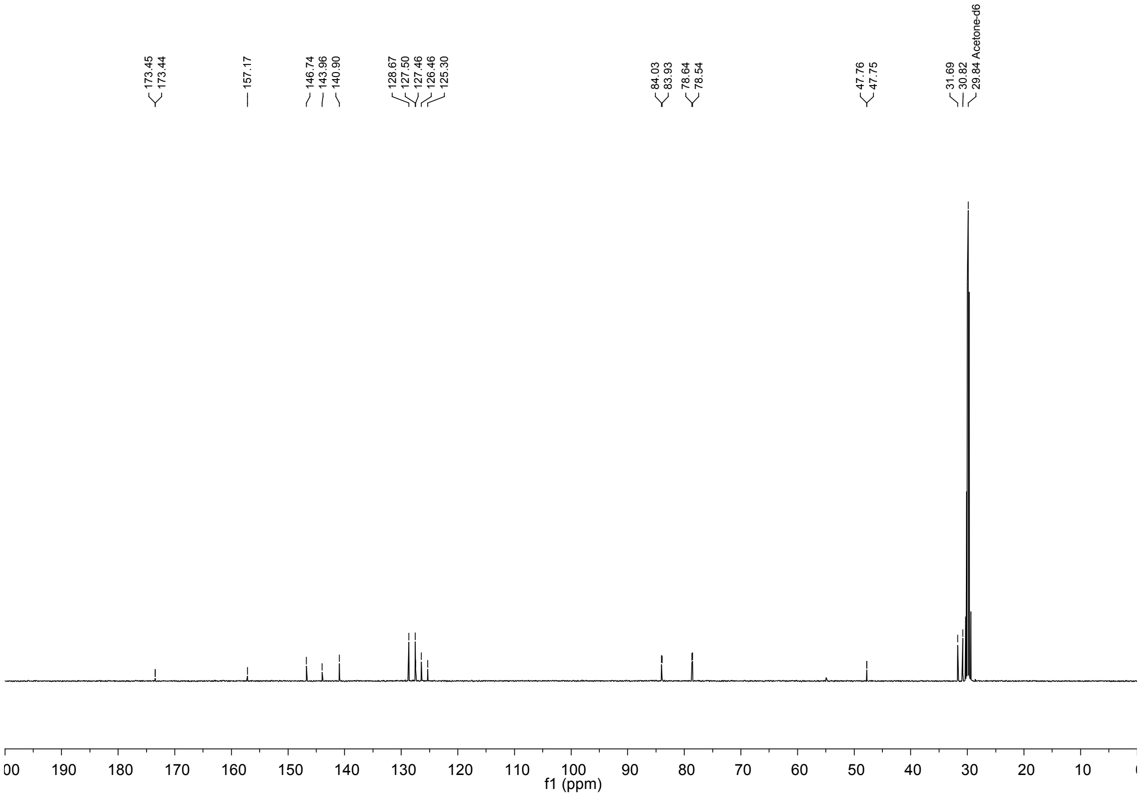

HSQC (acetone-d_6, 500 MHz)

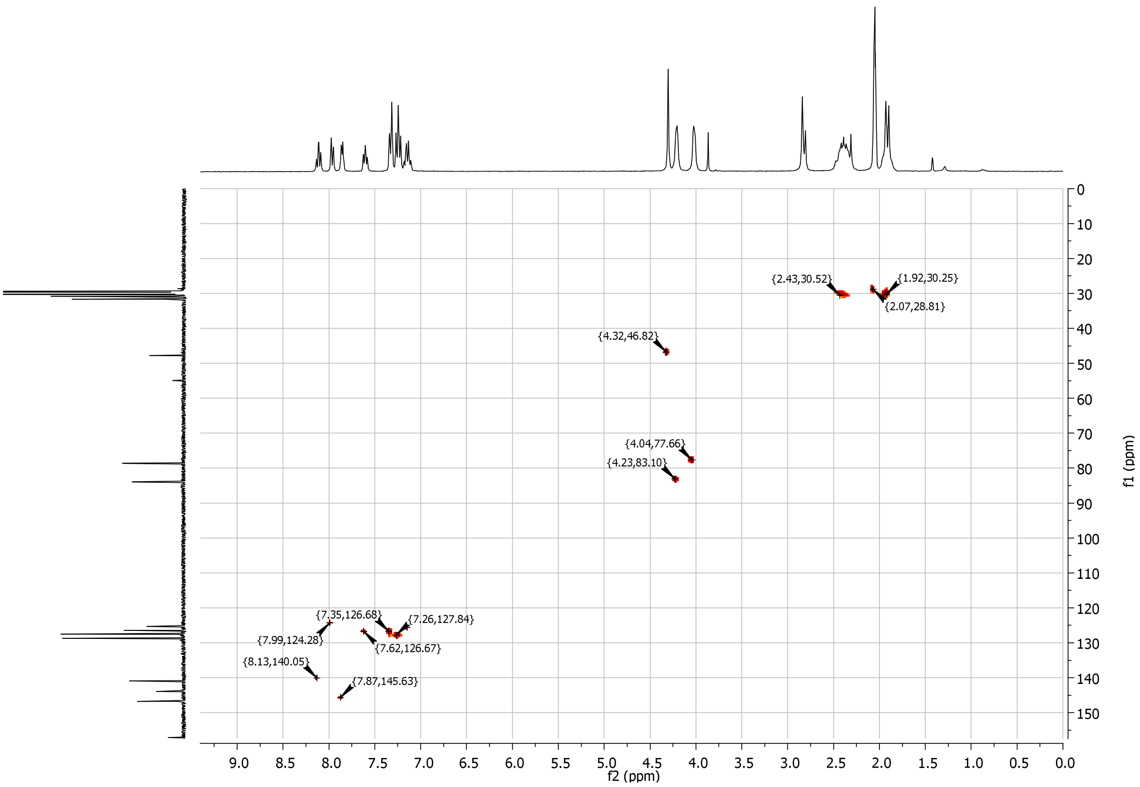

HMBC (acetone-d_6, 500 MHz)

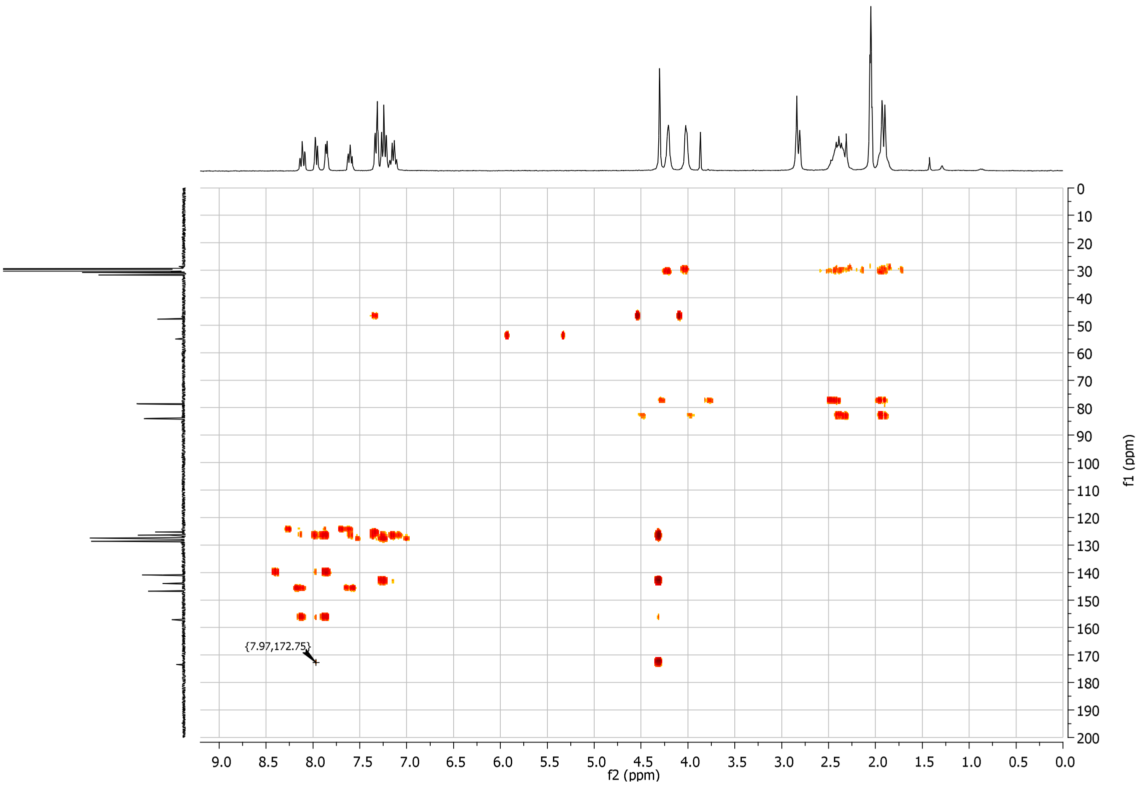

Rh^{I}-complex M

^{1}H NMR ($CDCl_3$, 300 MHz)

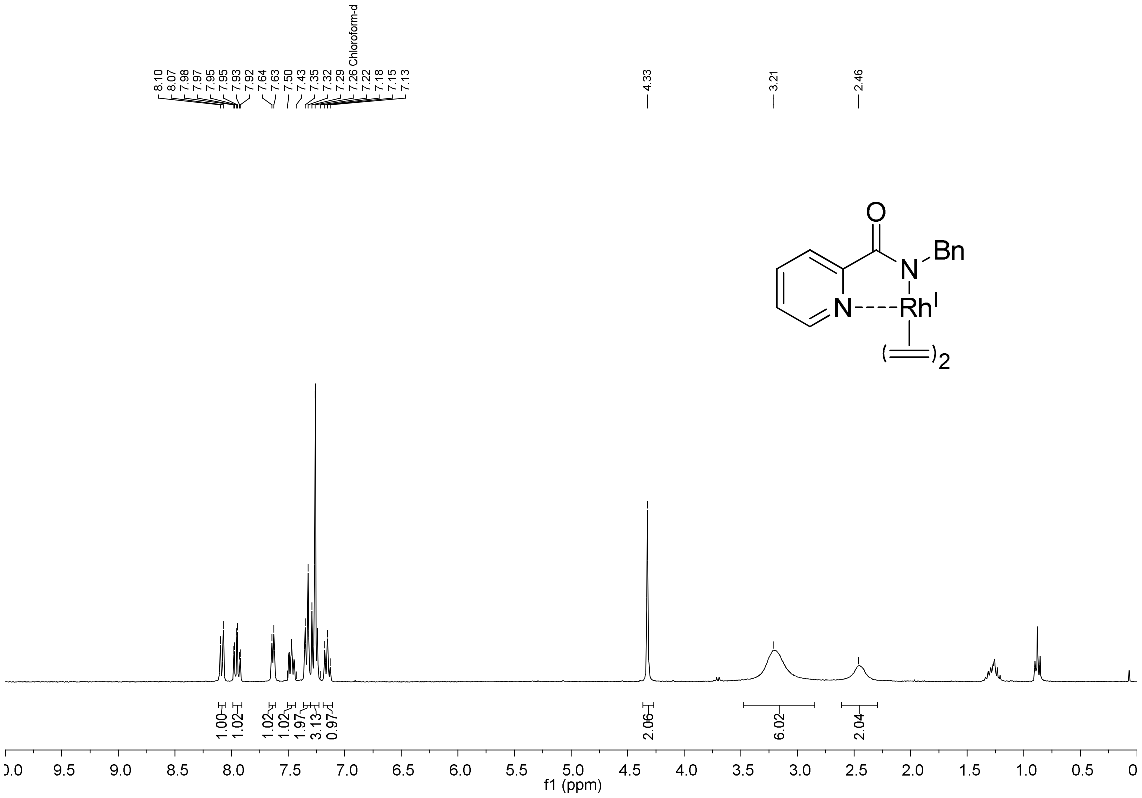

^{13}C NMR (acetone-d_6, 75 MHz)

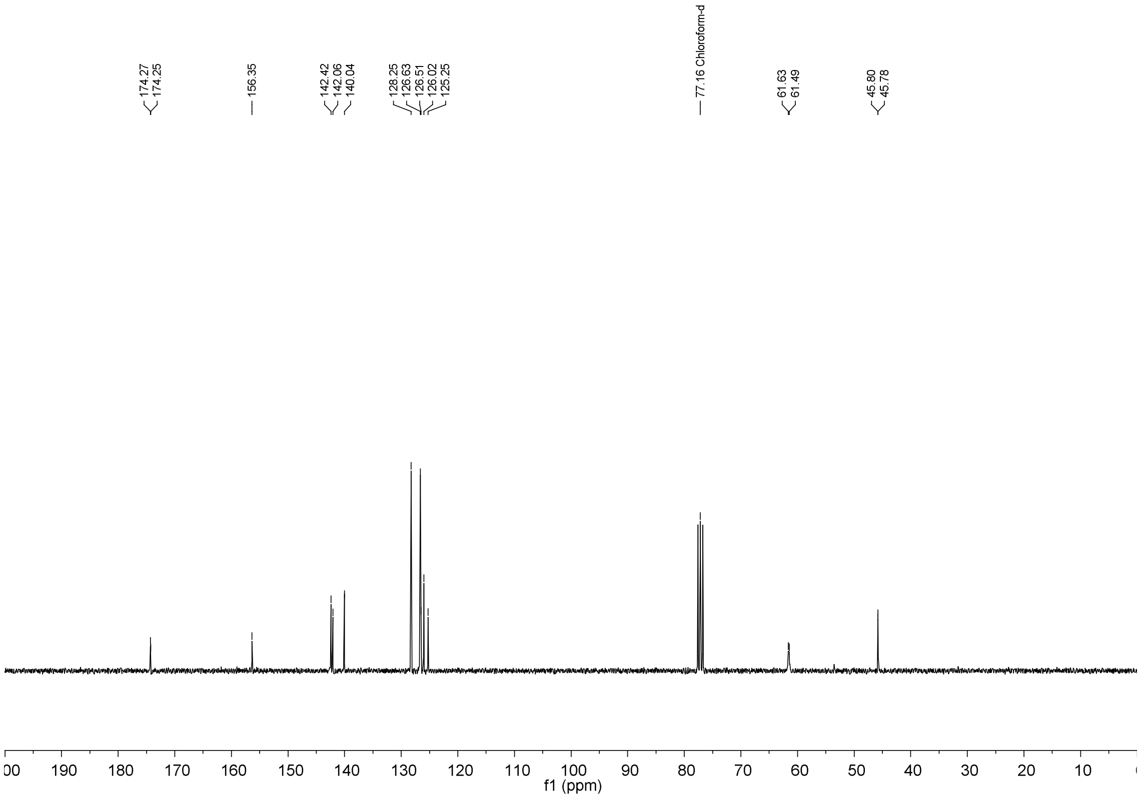

18. Theoretical calculations

18.1. Computational details

All calculations were performed with Gaussian 09[7] at DFT level. The geometries of all complexes here reported were fully optimized using the M06 hybrid functional[8] in the gas phase. The standard 6-31G(d)[9] basis set was used for C, H, N and O atoms. The LANL2DZ basis set, which includes the relativistic effective core potential (ECP) of Hay and Wadt and employs a split-valence (double-ζ) basis set, was used for Rh[10] (B1). Harmonic frequencies were calculated at the same level to characterize the stationary points and to determine the zero-point energies (ZPE). Final energies were obtained using the more extended 6-311+G(d,p)[11] basis set for all atoms expect Rh for which SDD[12] was used (B2). Relative free energies (in kcal·mol^{-1}) were evaluated at the M06/6-311+G(d,p)-SDD with ZPE and entropy corrections evaluated at 298 K by using the frequencies previously calculated at the M06/6-31G(d)-LANL2DZ level.

[7] Gaussian 09, Revision C.01, M. J.Frisch, G. W. Trucks, H. B. Schlegel, G. E. Scuseria, M. A. Robb, J. R. Cheeseman, G. Scalmani, V. Barone, B. Mennucci, G. A. Petersson, H. Nakatsuji, M. Caricato, X. Li, H. P. Hratchian, A. F. Izmaylov, J. Bloino, G. Zheng, J. L. Sonnenberg, M. Hada, M. Ehara, K. Toyota, R. Fukuda, J. Hasegawa, M. Ishida, T. Nakajima, Y. Honda, O. Kitao, H. Nakai, T. Vreven, J. A. JR. Montgomery, J. E. Peralta, F. Ogliaro, M. Bearpark, J. J. Heyd, E. Brothers, K. N. Kudin, V. N. Staroverov, T. Keith, R.; Kobayashi, J. Normand, K. Raghavachari, A. Rendell, J. C. Burant, S. S. Iyengar, J. Tomasi, M. Cossi, N. Rega, J. M. Millam, M.; Klene, J. E. M. Knox, J. B. Cross, V. Bakken, C. Adamo, J R. Jaramillo, R. Gomperts, R. E. Stratmann, O. Yazyev, A. Austin, J. R. Cammi, C. Pomelli, J. W. Ochterski, R. L. Martin, K. Morokuma, V. G. Zakrzewski, G. A. Voth, P. Salvador, J. J. Dannenberg, S. Dapprich, A. D. Daniels, O. Farkas, J. B. Foresman, J. V. Ortiz, J. Cioslowski and D. J. Fox, Gaussian, Inc., Wallingford CT, 2010.

[8] Y. Zhao and D. G. Truhlar, *Theor Chem Account*, 2008, **120**, 215.

[9] (*a*) R. Ditchfield, W. J. Hehre and J. A. Pople, *J. Chem. Phys.*, 1971, **54**, 724; (*b*) M. M. Francl, W. J. Petro, W. J. Hehre, J. S. Binkley, M. S. Gordon, D. J. DeFrees and J. A. Pople, *J. Chem. Phys.*, 1982, **77**, 3654.

[10] (*a*) P.J. Hay and W. R. Wadt, *J. Chem. Phys.*, 1985, **82**, 270; (*b*) P.J. Hay and W. R. Wadt, *J. Chem. Phys.*, 1985, **82**, 299.

[11] K. Raghavachari, J. S. Binkley, R. Seeger and J. A. Pople, *J. Chem. Phys.*, 1980, **72**, 650.

[12] D. Andrae, U. Haeussermann, M. Dolg, H. Stoll and H. Preuss, *Theor. Chem. Acc.*, 1990, **77**, 123.

16.2. Cartesian coordinates (Å) and energies (hartrees) of all the optimized structures

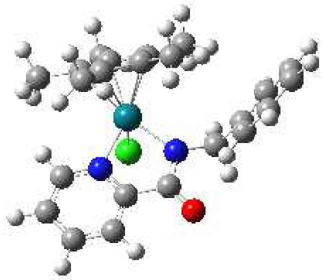

complex A

E(M06 / B1) = -1645.78747278
H(correction)= 0.469311
G(correction)= 0.382838
E(M06 / B2) = -1647.16635412
Imaginary frequencies: 0

6	0	3.20102	1.26484	0.43665
1	0	3.68429	0.29264	0.52609
6	0	3.90464	2.44793	0.60264
7	0	1.89741	1.25338	0.14291
1	0	4.9643	2.4169	0.84345
6	0	3.23089	3.65608	0.43917
6	0	1.24843	2.41073	-0.04168
45	0	0.70996	-0.48381	-0.19798
1	0	3.7573	4.60127	0.55828
6	0	1.88608	3.63635	0.10395
6	0	-0.17435	2.3327	-0.50285
6	0	0.82455	-1.44	1.7703
6	0	-0.50674	-1.60767	1.27605
6	0	-0.42385	-2.33705	0.03477
6	0	0.9594	-2.68827	-0.17539
6	0	1.73475	-2.12849	0.87705
17	0	1.46934	-0.3518	-2.49032
7	0	-0.61309	1.07265	-0.64238
1	0	1.29724	4.53299	-0.07347
8	0	-0.79562	3.37245	-0.74524
6	0	1.2007	-0.72728	3.0219
6	0	-1.74173	-1.13451	1.95767
6	0	-1.55339	-2.80123	-0.81585
6	0	1.4784	-3.50936	-1.29842
6	0	3.20687	-2.28861	1.04296
6	0	-1.82826	0.90007	-1.42026
1	0	0.46059	0.04045	3.27682
1	0	2.17381	-0.22859	2.92743
1	0	1.26651	-1.4247	3.86972
1	0	-2.61169	-1.14897	1.29209
1	0	-1.63589	-0.10483	2.32044
1	0	-1.96355	-1.77483	2.8242
1	0	-1.72108	-3.88131	-0.69138
1	0	-1.34779	-2.61674	-1.87899
1	0	-2.48514	-2.28419	-0.56041
1	0	2.49787	-3.21887	-1.57473
1	0	0.85766	-3.40463	-2.19429
1	0	1.4879	-4.57094	-1.00774
1	0	3.46186	-3.30843	1.36594
1	0	3.60872	-1.60326	1.79949
1	0	3.73629	-2.10227	0.09813
1	0	-1.70249	0.0181	-2.06623
1	0	-1.92528	1.77334	-2.08585
6	0	-3.09052	0.76922	-0.6066
6	0	-3.35078	1.64654	0.45008
6	0	-4.0378	-0.20899	-0.91209
1	0	-2.63537	2.44084	0.66019
6	0	-4.51309	1.52092	1.20158
6	0	-5.2048	-0.33713	-0.16106
1	0	-3.85758	-0.87797	-1.7561
1	0	-4.70278	2.21284	2.0215
6	0	-5.44026	0.52262	0.90625
1	0	-5.93002	-1.11055	-0.41143
1	0	-6.34876	0.42539	1.49884

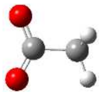

OAc

E(M06 / B1) = -228.377962939
H(correction)= 0.053862
G(correction)= 0.021010
E(M06 / B2) = -228.468403841
Imaginary frequencies: 0

6	0	0.21947	0.00197	-0.00377
8	0	0.80754	-1.10073	0.00091
8	0	0.68498	1.16353	0.00078
6	0	-1.34311	-0.05966	-0.00131
1	0	-1.7472	0.55319	-0.8211
1	0	-1.72147	-1.08763	-0.0939
1	0	-1.72961	0.37819	0.932

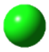

Cl

E(M06 / B1) = -460.218180759
H(correction)= 0.002361
G(correction)= -0.015023
E(M06 / B2) = -460.261704437
Imaginary frequencies: 0

17	0	0.	0.	0.

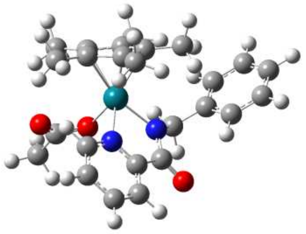

modA

E(M06 / B1) = -1413.96314662
H(correction)= 0.523705
G(correction)= 0.430332
E(M06 / B2) = -1415.37982519
Imaginary frequencies: 0

6	0	-2.85292	1.49445	-0.8591
1	0	-3.39014	0.55926	-1.00089
6	0	-3.43962	2.72933	-1.09666
7	0	-1.60234	1.39095	-0.39751
1	0	-4.45779	2.77709	-1.47455
6	0	-2.71182	3.88458	-0.82328
6	0	-0.90405	2.49756	-0.10635
45	0	-0.53962	-0.43074	-0.04889
1	0	-3.15111	4.86577	-0.99483
6	0	-1.42975	3.76704	-0.30769
6	0	0.43783	2.3121	0.53684
6	0	-0.44612	-1.23598	-2.08305
6	0	0.79404	-1.52025	-1.43218
6	0	0.50327	-2.3259	-0.27124
6	0	-0.91539	-2.59402	-0.2692
6	0	-1.50587	-1.93026	-1.37698
7	0	0.79319	1.02448	0.64887
8	0	-1.25729	-0.33505	1.93851
8	0	-3.36347	-0.70479	1.21749
1	0	-0.80979	4.61749	-0.03379
8	0	1.06733	3.29965	0.93127
6	0	-0.61723	-0.4227	-3.31803
6	0	2.13211	-1.06937	-1.90233
6	0	1.47845	-2.91727	0.68521
6	0	-1.63205	-3.40139	0.75281
6	0	-2.94789	-1.97954	-1.74319
6	0	1.88795	0.72862	1.5551
6	0	-2.52952	-0.4896	2.1016
1	0	0.19819	0.30036	-3.4365
1	0	-1.55836	0.14163	-3.30023
1	0	-0.63158	-1.06201	-4.21274
1	0	2.90072	-1.17222	-1.12806
1	0	2.1191	-0.01284	-2.19665
1	0	2.44324	-1.65997	-2.77658
1	0	1.58755	-3.99907	0.51776
1	0	1.14919	-2.77626	1.72409
1	0	2.46847	-2.45891	0.58138
1	0	-2.67277	-3.07365	0.85169
1	0	-1.15604	-3.29664	1.73591
1	0	-1.61631	-4.46739	0.48207
1	0	-3.23123	-2.98493	-2.08611
1	0	-3.18338	-1.28194	-2.55655
1	0	-3.5639	-1.72667	-0.87003
1	0	1.63927	-0.19309	2.10339
1	0	1.94352	1.54139	2.29776
6	0	3.2352	0.58392	0.89403
6	0	-2.94514	-0.40447	3.55796
6	0	3.66394	1.51828	-0.05315
6	0	4.08719	-0.4697	1.22773
1	0	-2.58988	0.53416	3.99821
1	0	-4.03253	-0.47503	3.65185
1	0	-2.47499	-1.21946	4.12255
1	0	3.02022	2.36739	-0.28105
6	0	4.8976	1.37449	-0.67658
6	0	5.3254	-0.6161	0.6049
1	0	3.77399	-1.18508	1.991
1	0	5.21906	2.1092	-1.41407
6	0	5.72871	0.30115	-0.35928
1	0	5.97272	-1.45031	0.87309
1	0	6.6928	0.18843	-0.85323

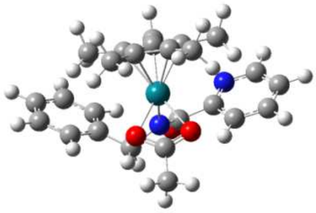

IAa

E(M06 / B1) = -1413.93859303
H(correction)= 0.523395
G(correction)= 0.430161
E(M06 / B2) = -1415.35142771
Imaginary frequencies: 0

6	0	3.9188	-2.62074	-0.71537
1	0	4.2888	-3.27725	-1.50639
6	0	4.80571	-1.82275	0.00322
7	0	2.60575	-2.65169	-0.49784
1	0	5.87362	-1.86226	-0.20209
6	0	4.28245	-0.98282	0.9787
6	0	2.10937	-1.85178	0.45669
1	0	4.93418	-0.33561	1.56451
6	0	2.91042	-0.98418	1.20356
6	0	0.65881	-2.06475	0.82927
1	0	2.45243	-0.32743	1.93956
7	0	-0.08896	-0.96345	1.07077
8	0	0.29413	-3.229	1.00989
6	0	-1.33955	-1.20413	1.78924
45	0	0.08472	0.84378	0.02928
1	0	-1.50983	-0.35906	2.46853
1	0	-1.19388	-2.10859	2.39847
6	0	-2.56815	-1.39262	0.93749
6	0	-0.57662	2.24939	-1.50964
8	0	-0.95671	1.78576	1.72842
6	0	-2.66669	-2.4785	0.05869
6	0	-3.64939	-0.51809	1.05028
6	0	-1.04459	0.90273	-1.80421
6	0	0.83692	2.23082	-1.50218
6	0	-1.47017	3.39492	-1.19595
6	0	0.13318	1.93559	2.3625
1	0	-1.82889	-3.17544	0.00237
6	0	-3.822	-2.67437	-0.69119
6	0	-4.81168	-0.71967	0.30767
1	0	-3.57393	0.32955	1.73404

6	0	0.10331	0.0682	-2.02679
6	0	-2.46525	0.52545	-2.00413
6	0	1.26631	0.87104	-1.79854
6	0	1.76654	3.35277	-1.20459
1	0	-0.92954	4.20189	-0.68901
1	0	-2.28683	3.08093	-0.53395
1	0	-1.9182	3.80327	-2.11319
8	0	1.22899	1.6333	1.81214
6	0	0.09687	2.41979	3.77972
1	0	-3.89115	-3.52887	-1.36389
6	0	-4.89929	-1.79628	-0.56843
1	0	-5.65047	-0.03222	0.41481
6	0	0.06738	-1.36413	-2.42625
1	0	-2.77954	0.78425	-3.027
1	0	-3.12569	1.05219	-1.30559
1	0	-2.62888	-0.5479	-1.85545
6	0	2.68655	0.47106	-1.96094
1	0	2.34264	3.62938	-2.09954
1	0	2.48151	3.07215	-0.42054
1	0	1.22982	4.24402	-0.86149
1	0	-0.14264	1.56979	4.43214
1	0	1.06756	2.82411	4.08065
1	0	-0.68932	3.16996	3.91128
1	0	-5.80728	-1.95927	-1.14775
1	0	-0.83602	-1.85769	-2.04787
1	0	0.92967	-1.93032	-2.04993
1	0	0.05845	-1.44491	-3.52312
1	0	2.80051	-0.61601	-2.02114
1	0	3.29978	0.82326	-1.12097
1	0	3.09664	0.91182	-2.88213

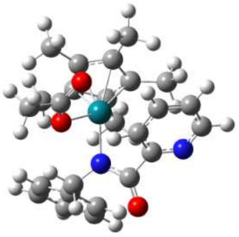

IIAa

E(M06 / B1) = -1413.92923243
H(correction)= 0.523615
G(correction)= 0.430969
E(M06 / B2) = -1415.34305775
Imaginary frequencies: 0

6	0	2.09169	3.69101	0.27266
1	0	2.18514	4.5948	0.87989
6	0	3.2336	2.93827	-0.02843
7	0	0.86024	3.38644	-0.12419
1	0	4.20785	3.25069	0.34253
6	0	3.08688	1.81735	-0.8275
6	0	0.71918	2.29402	-0.88662
1	0	3.93233	1.19465	-1.11438
6	0	1.80534	1.48961	-1.28404
6	0	-0.65752	1.90192	-1.36716
1	0	1.66323	0.71401	-2.03434
7	0	-0.88484	0.60481	-1.1134
8	0	-1.38986	2.69993	-1.94918
6	0	-2.00253	-0.06256	-1.75315
45	0	0.5979	-0.30809	0.04136
1	0	-1.69954	-1.097	-1.97408
1	0	-2.17787	0.44022	-2.71892
6	0	-3.2835	-0.05662	-0.95841
6	0	1.18087	-1.90276	1.44333
8	0	0.8904	-1.52556	-1.66417
6	0	-3.83334	1.15123	-0.51421
6	0	-3.95381	-1.24512	-0.66935
6	0	-0.25281	-1.64407	1.45178
6	0	1.84154	-0.72403	1.84878
6	0	1.81468	-3.18389	1.03617
6	0	2.11745	-1.8643	-1.89895
1	0	-3.32406	2.08153	-0.76943
6	0	-5.00997	1.15798	0.22534
6	0	-5.13392	-1.241	0.07332
1	0	-3.54403	-2.1888	-1.03598
6	0	-0.46006	-0.30557	1.96296
6	0	-1.29076	-2.65439	1.1203
6	0	0.82351	0.27414	2.16403
6	0	3.3148	-0.54235	1.93785
1	0	2.77612	-3.00702	0.5406
1	0	1.17325	-3.7348	0.33746
1	0	1.98152	-3.82444	1.91426
8	0	3.10973	-1.43529	-1.30476
6	0	2.25869	-2.9159	-2.97971
1	0	-5.42746	2.10504	0.56543
6	0	-5.66059	-0.03747	0.5291
1	0	-5.6425	-2.17961	0.29132
6	0	-1.76301	0.36345	2.21815
1	0	-1.27553	-3.4783	1.84902
1	0	-1.10647	-3.08407	0.12561
1	0	-2.29538	-2.21711	1.11328
6	0	1.08221	1.62594	2.7227
1	0	3.70405	-0.95991	2.87796
1	0	3.59224	0.5179	1.90715
1	0	3.81347	-1.04205	1.09844
1	0	1.48575	-2.81115	-3.74738
1	0	3.25553	-2.86638	-3.42797
1	0	2.14	-3.90722	-2.52043
1	0	-6.58108	-0.02814	1.11122
1	0	-2.58643	-0.12699	1.6863
1	0	-1.74414	1.40832	1.88257
1	0	-1.99151	0.35779	3.29391
1	0	0.3111	2.34475	2.42189
1	0	2.05001	2.02466	2.39507
1	0	1.0972	1.58198	3.82187

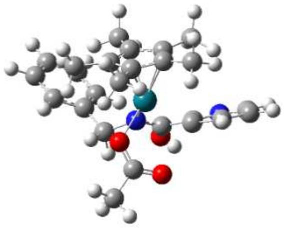

TS(II-III)Aa

E(M06 / B1) = -1413.90469376
H(correction)= 0.517537
G(correction)= 0.425993
E(M06 / B2) = -1415.31935178

Imaginary frequencies: 1 (-1578.5796 cm^{-1})

6	0	-2.90341	3.46858	-0.69462
1	0	-3.23195	4.40539	-1.15082
6	0	-3.80983	2.41698	-0.54004
7	0	-1.62939	3.4235	-0.30826
1	0	-4.8481	2.53839	-0.84436
6	0	-3.34904	1.23417	0.02217
6	0	-1.20898	2.28121	0.23717
1	0	-4.03171	0.39752	0.18443
6	0	-2.00832	1.13472	0.41541
6	0	0.20869	2.19597	0.74131
7	0	0.63004	0.91326	0.80231
8	0	0.84418	3.18507	1.09943
6	0	1.83544	0.61185	1.55404
45	0	-0.61179	-0.54919	0.00465
1	0	1.69985	-0.36947	2.03381
1	0	1.92251	1.36772	2.35239
6	0	3.10864	0.60297	0.74767
6	0	0.45943	-2.09463	-1.13827
8	0	-0.50731	-1.46276	1.96001
6	0	3.51207	1.74551	0.04743
6	0	3.91264	-0.53521	0.69752
6	0	-0.93622	-2.48685	-1.02607
6	0	0.51026	-0.87988	-1.88841
6	0	1.59932	-2.9069	-0.63188
6	0	-1.14597	-0.93914	2.91677
1	0	2.89095	2.64029	0.11211
6	0	4.67996	1.73141	-0.70563
6	0	5.0841	-0.55215	-0.05849
1	0	3.61555	-1.42126	1.26267
6	0	-1.73183	-1.51369	-1.6743
6	0	-1.40136	-3.71585	-0.3297
6	0	-0.84141	-0.47066	-2.15704
6	0	1.72073	-0.13351	-2.31956
1	0	1.64326	-3.88012	-1.14321
1	0	1.49384	-3.10344	0.44442
1	0	2.55821	-2.4	-0.78721
8	0	-1.89804	0.0682	2.81548
6	0	-0.9684	-1.54363	4.28253
1	0	4.98377	2.62674	-1.24689
6	0	5.46633	0.58123	-0.76792
1	0	5.70018	-1.45057	-0.08733
6	0	-3.20501	-1.57026	-1.88277
1	0	-2.49037	-3.72923	-0.21017
1	0	-0.95291	-3.79135	0.66922
1	0	-1.1142	-4.61453	-0.89482
6	0	-1.23778	0.71373	-2.96715
1	0	2.61701	-0.44159	-1.76987
1	0	1.5999	0.94484	-2.16066
1	0	1.89729	-0.29724	-3.393
1	0	-0.68723	-2.59798	4.21063
1	0	-0.15997	-1.00622	4.79463
1	0	-1.87906	-1.42321	4.87614
1	0	6.38039	0.57399	-1.36027
1	0	-3.44424	-2.16403	-2.77723
1	0	-3.63281	-0.57189	-2.02807
1	0	-3.72008	-2.0332	-1.03173
1	0	-0.52639	1.53924	-2.84368
1	0	-2.22804	1.08906	-2.6813
1	0	-1.2705	0.45936	-4.03707
1	0	-1.87027	0.48916	1.55926

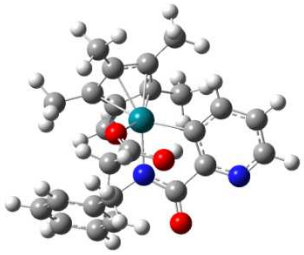

IIIAa

E(M06 / B1) = -1413.92225508
H(correction)= 0.522527
G(correction)= 0.426992
E(M06 / B2) = -1415.33799069
Imaginary frequencies: 0

6	0	-3.29845	0.90351	-0.70169
1	0	-3.85938	-0.0237	-0.569
6	0	-3.9471	2.03816	-1.18437
6	0	-1.94016	0.97378	-0.38658
1	0	-5.00627	2.01619	-1.43771
6	0	-3.21041	3.21102	-1.33812
6	0	-1.32622	2.23138	-0.52721
45	0	-0.61913	-0.5609	0.03312
1	0	-3.68511	4.1092	-1.73944
7	0	-1.9282	3.32426	-0.99978
6	0	0.07259	2.34408	0.01213
7	0	0.52503	1.12713	0.40487
8	0	0.67113	3.40842	0.14718
6	0	1.69373	1.0726	1.26447
1	0	1.56363	0.22959	1.96278
1	0	1.71254	2.00172	1.86097
6	0	3.02356	0.92246	0.57099
6	0	3.39695	1.80576	-0.44682
6	0	3.91445	-0.08176	0.94964
1	0	2.71357	2.61286	-0.71101
6	0	4.62198	1.66123	-1.08699
6	0	5.14601	-0.2254	0.31264
1	0	3.63853	-0.76004	1.76047
1	0	4.9014	2.3549	-1.8792
6	0	5.49816	0.64174	-0.71587
1	0	5.82918	-1.01637	0.62083
1	0	6.45769	0.53251	-1.21955
1	0	-1.98905	1.18666	1.61325
8	0	-1.99886	1.19872	2.60498
6	0	-1.32482	0.16116	3.04423
8	0	-0.81404	-0.68472	2.31441
6	0	-1.22407	0.11252	4.53322
1	0	-0.81328	-0.84625	4.85598
1	0	-0.56528	0.92276	4.86928
1	0	-2.20571	0.28436	4.98697
6	0	-0.48082	-1.31583	-1.95742
6	0	0.81205	-1.64516	-1.36934
6	0	-1.4869	-2.12239	-1.29973
6	0	-0.65235	-0.48814	-3.18358
6	0	0.58895	-2.49816	-0.27337
6	0	2.09947	-1.1336	-1.90574

6	0	-0.84242	-2.76247	-0.21012
6	0	-2.90363	-2.29375	-1.72373
1	0	-0.00696	0.39914	-3.14378
1	0	-1.68333	-0.13249	-3.29242
1	0	-0.3893	-1.0568	-4.08828
6	0	1.59258	-3.0383	0.6867
1	0	2.95207	-1.3789	-1.26403
1	0	2.08202	-0.04225	-2.01441
1	0	2.27979	-1.56503	-2.90199
6	0	-1.47649	-3.63261	0.81708
1	0	-3.00973	-3.17533	-2.37299
1	0	-3.26493	-1.42741	-2.28841
1	0	-3.57689	-2.43422	-0.86831
1	0	1.68352	-4.13201	0.60996
1	0	1.31342	-2.80372	1.72431
1	0	2.58355	-2.60315	0.51035
1	0	-2.56892	-3.54707	0.80439
1	0	-1.13116	-3.36624	1.82427
1	0	-1.21829	-4.68816	0.64588

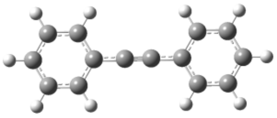

Diphenylacetylene

E(M06 / B1) = -539.031917418
H(correction)= 0.202996
G(correction)= 0.152671
E(M06 / B2) = -539.1569763
Imaginary frequencies: 0

6	0	0.60708	0.00003	0.
6	0	-0.60708	0.00004	0.
6	0	-2.03027	0.00002	0.
6	0	-2.74208	1.20886	0.00016
6	0	-2.74204	-1.20885	-0.00016
6	0	-4.12921	1.20473	0.00016
1	0	-2.18874	2.14625	0.00028
6	0	-4.12917	-1.20476	-0.00016
1	0	-2.18867	-2.14622	-0.00028
6	0	-4.82717	-0.00003	0.
1	0	-4.67123	2.14885	0.00028
1	0	-4.67116	-2.1489	-0.00028
1	0	-5.91558	-0.00004	0.
6	0	2.03027	0.00002	0.
6	0	2.74208	1.20886	-0.00016
6	0	2.74204	-1.20885	0.00016
6	0	4.12921	1.20473	-0.00016
1	0	2.18874	2.14625	-0.00028
6	0	4.12918	-1.20476	0.00016
1	0	2.18867	-2.14622	0.00028
6	0	4.82717	-0.00003	0.
1	0	4.67123	2.14885	-0.00028
1	0	4.67116	-2.1489	0.00028
1	0	5.91558	-0.00004	0.

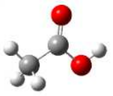

HOAc

E(M06 / B1) = -228.959405766
H(correction)= 0.067747
G(correction)= 0.035499
E(M06 / B2) = -229.030738416
Imaginary frequencies: 0

1	0	1.70653	-0.81366	-0.00005
6	0	0.09255	0.12518	0.00011
8	0	0.65375	1.19206	-0.00009
8	0	0.76171	-1.0475	0.00001
6	0	-1.38731	-0.09887	0.
1	0	-1.67833	-0.68112	0.88159
1	0	-1.9052	0.86217	-0.0004
1	0	-1.67815	-0.6818	-0.8812.

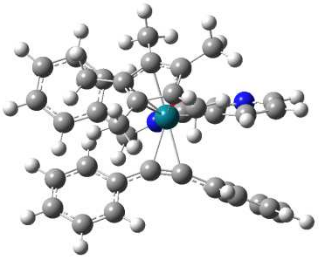

IVAa

E(M06 / B1) = -1723.98134015
H(correction)= 0.658293
G(correction)= 0.550344
E(M06 / B2) = -1725.4678699
Imaginary frequencies: 0

6	0	-3.1727	-0.92293	0.75892
1	0	-3.37486	-0.08064	1.42459
6	0	-4.18824	-1.81817	0.43797
6	0	-1.91593	-1.11119	0.1962
1	0	-5.18977	-1.70311	0.85071
6	0	-3.89852	-2.86226	-0.44024
6	0	-1.74922	-2.16013	-0.70432
45	0	-0.20817	-0.04803	0.6134
1	0	-4.67162	-3.58863	-0.70112
7	0	-2.71259	-3.03338	-1.01632
6	0	-0.45504	-2.19569	-1.45447
6	0	-1.27782	1.52006	-0.7098
6	0	-0.44927	-0.86377	2.72406
7	0	0.34219	-1.15033	-1.10371
8	0	-0.19889	-3.01849	-2.33045
6	0	-0.0704	1.81089	-0.57142
6	0	-2.63541	1.45212	-1.15931
6	0	-0.47476	0.55912	2.78305
6	0	0.82057	-1.2564	2.17818
6	0	-1.46738	-1.82088	3.23563
6	0	1.42834	-0.8566	-2.0197
6	0	1.06052	2.69565	-0.70942
6	0	-2.9739	0.62738	-2.24228
6	0	-3.63772	2.17957	-0.50526
6	0	0.83718	1.03365	2.39588

6	0	-1.56698	1.43494	3.29597
6	0	1.64444	-0.07788	2.05057
6	0	1.27894	-2.66122	2.00042
1	0	-1.56833	-2.69812	2.58493
1	0	-2.46019	-1.36952	3.3372
1	0	-1.15975	-2.17934	4.22962
1	0	1.50606	0.23196	-2.15869
1	0	1.1512	-1.285	-2.99715
6	0	2.77766	-1.41357	-1.64006
6	0	2.38036	2.23607	-0.67391
6	0	0.82534	4.07117	-0.85382
6	0	-4.29464	0.53076	-2.65339
1	0	-2.18903	0.05884	-2.73886
6	0	-4.95852	2.07744	-0.92407
1	0	-3.3695	2.81781	0.33671
6	0	1.24272	2.46247	2.50652
1	0	-1.32207	1.85559	4.28307
1	0	-2.51224	0.88847	3.39808
1	0	-1.7465	2.28056	2.61686
6	0	3.10528	-0.09477	1.75358
1	0	2.13839	-2.71616	1.32245
1	0	0.48689	-3.29594	1.58113
1	0	1.57612	-3.0976	2.96659
6	0	2.88974	-2.71981	-1.15433
6	0	3.9431	-0.66381	-1.81271
6	0	3.44189	3.12447	-0.7821
1	0	2.56764	1.17327	-0.53983
6	0	1.88943	4.95801	-0.95287
1	0	-0.20249	4.43148	-0.87786
6	0	-5.2895	1.24999	-1.99329
1	0	-4.55061	-0.11882	-3.48817
1	0	-5.73399	2.64442	-0.41152
1	0	1.41246	2.71778	3.56352
1	0	0.45674	3.13367	2.13513
1	0	2.16025	2.69033	1.95303
1	0	3.66171	-0.51664	2.60409
1	0	3.50056	0.91212	1.57304
1	0	3.33836	-0.70908	0.87433
1	0	1.98723	-3.32695	-1.08051
6	0	4.13159	-3.24081	-0.80685
6	0	5.18915	-1.18467	-1.46936
1	0	3.87284	0.34265	-2.23071
6	0	3.20091	4.4895	-0.91635
1	0	4.46241	2.74226	-0.75261
1	0	1.69364	6.02382	-1.05795
1	0	-6.32595	1.16667	-2.31578
1	0	4.2022	-4.26086	-0.43008
6	0	5.28547	-2.4714	-0.95098
1	0	6.08594	-0.58125	-1.60654
1	0	4.03205	5.18835	-0.99174
1	0	6.25594	-2.88115	-0.67484

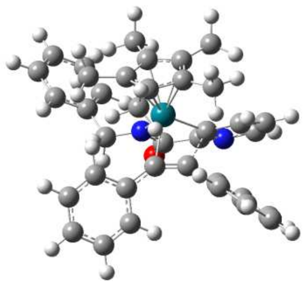

TS(IV-V)Aa

E(M06 / B1) = -1723.9649261
H(correction)= 0.656305
G(correction)= 0.548299
E(M06 / B2) = -1725.44931843
Imaginary frequencies: 1 (-262.4215 cm^{-1})

6	0	-2.05235	-2.4616	-0.25227
1	0	-2.63112	-2.30752	0.66058
6	0	-2.30365	-3.56188	-1.05829
6	0	-1.07773	-1.53744	-0.64555
1	0	-3.04718	-4.30539	-0.77561
6	0	-1.60288	-3.67852	-2.25956
6	0	-0.39537	-1.78798	-1.84556
6	0	-1.80751	0.34806	-0.441
1	0	-1.80274	-4.51446	-2.93316
7	0	-0.6707	-2.81552	-2.65314
6	0	0.72958	-0.87269	-2.20436
6	0	-0.93656	1.2846	-0.28175
6	0	-3.25987	0.22077	-0.48699
7	0	1.14533	-0.16465	-1.12581
8	0	1.21605	-0.81261	-3.33217
6	0	-0.6909	2.68091	-0.51379
45	0	0.01674	-0.30209	0.65727
6	0	-3.90907	-0.44293	-1.53558
6	0	-4.02953	0.84926	0.50054
6	0	2.16743	0.83893	-1.37527
6	0	0.2473	3.41232	0.2262
6	0	-1.43661	3.34287	-1.50432
6	0	1.58378	-1.18801	2.01919
6	0	1.39803	0.20178	2.39725
6	0	0.05829	0.34722	2.85243
6	0	-0.59515	-0.92753	2.73118
6	0	0.37517	-1.88623	2.26963
6	0	-5.29593	-0.4805	-1.58663
1	0	-3.31408	-0.91901	-2.31387
6	0	-5.41801	0.79733	0.4537
1	0	-3.52048	1.3909	1.29871
1	0	2.00442	1.69238	-0.70178
1	0	2.03871	1.21021	-2.40613
6	0	3.5848	0.34674	-1.20826
6	0	0.42589	4.76972	-0.00515
1	0	0.83415	2.89482	0.98459
6	0	-1.25089	4.69748	-1.73468
1	0	-2.15743	2.77204	-2.08876
6	0	2.85275	-1.81031	1.54892
6	0	2.49429	1.20616	2.48369
6	0	-0.58954	1.57543	3.39461
6	0	-1.99196	-1.20216	3.17808
6	0	0.18438	-3.35054	2.07523
6	0	-6.05306	0.12974	-0.58894

1	0	-5.79067	-0.9898	-2.41183
1	0	-6.00423	1.2857	1.23036
6	0	3.99027	-0.87891	-1.7454
6	0	4.52813	1.12117	-0.53034
6	0	-0.32075	5.4165	-0.98613
1	0	1.15837	5.32561	0.57793
1	0	-1.83195	5.19691	-2.5081
1	0	3.57338	-1.06456	1.19321
1	0	2.66943	-2.50429	0.71792
1	0	3.32602	-2.3805	2.36263
1	0	3.13367	1.00286	3.35603
1	0	2.11196	2.22833	2.59493
1	0	3.14224	1.17864	1.5989
1	0	-0.72006	1.50311	4.48465
1	0	-1.58579	1.73293	2.95888
1	0	-0.00051	2.47675	3.18966
1	0	-2.09273	-1.05705	4.26392
1	0	-2.29376	-2.23325	2.9593
1	0	-2.71698	-0.53433	2.69078
1	0	0.67481	-3.69695	1.15557
1	0	-0.87408	-3.62512	2.00002
1	0	0.61661	-3.91632	2.91365
1	0	-7.14026	0.09012	-0.62919
1	0	3.27361	-1.46535	-2.31779
6	0	5.29413	-1.32795	-1.57237
6	0	5.83601	0.67313	-0.35439
1	0	4.231	2.09607	-0.13641
1	0	-0.17326	6.47872	-1.17295
1	0	5.59395	-2.28564	-1.99645
6	0	6.22011	-0.56026	-0.86782
1	0	6.55351	1.2905	0.18511
1	0	7.2392	-0.91911	-0.73026 -

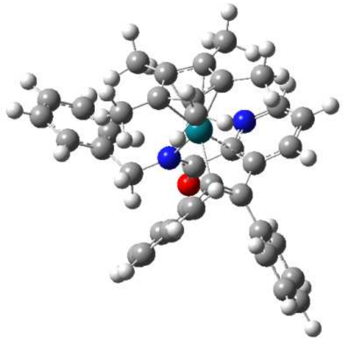

VAa

E(M06 / B1) = -1724.0124951
H(correction)= 0.660039
G(correction)= 0.552458
E(M06 / B2) = -1725.49367446
Imaginary frequencies: 0

6	0	1.96362	2.83784	-0.05474
1	0	2.88116	2.78457	0.53324
6	0	1.34036	4.0421	-0.30112
6	0	1.4324	1.64764	-0.58998
1	0	1.73695	4.97812	0.08785
6	0	0.18483	4.03644	-1.0987
6	0	0.26548	1.76573	-1.38881
6	0	2.12829	0.34085	-0.36488
1	0	-0.32641	4.97603	-1.32245
7	0	-0.34027	2.94678	-1.63134
6	0	-0.33025	0.55335	-2.07968
6	0	1.39761	-0.4962	0.38606
6	0	3.4548	0.13386	-0.97753
7	0	-0.84918	-0.2677	-1.16096
8	0	-0.31119	0.43809	-3.30321
6	0	1.67894	-1.88838	0.72325
45	0	-0.37816	0.51199	0.72831
6	0	3.72246	0.6924	-2.23512
6	0	4.46289	-0.60364	-0.34511
6	0	-1.38726	-1.54956	-1.56781
6	0	1.45991	-2.89135	-0.23291
6	0	2.17707	-2.26626	1.97806
6	0	-1.77727	-0.30067	2.13164
6	0	4.95798	0.51151	-2.84359
1	0	2.93767	1.24768	-2.7511
6	0	5.69915	-0.77998	-0.95409
1	0	4.27572	-1.03336	0.6381
1	0	-1.15507	-2.29777	-0.79314
1	0	-0.86801	-1.85888	-2.49217
6	0	-2.87348	-1.53819	-1.81955
6	0	1.69429	-4.22669	0.07168
1	0	1.12462	-2.60034	-1.22782
6	0	2.41321	-3.60304	2.28037
1	0	2.41206	-1.48924	2.70511
6	0	-0.7063	0.26686	2.92541
6	0	-2.51725	0.80154	1.52189
6	0	-2.19673	-1.72731	2.15792
6	0	5.95304	-0.22215	-2.2041
1	0	5.14181	0.94271	-3.82659
1	0	6.47347	-1.35157	-0.44411
6	0	-3.44038	-0.5662	-2.6495
6	0	-3.70493	-2.49804	-1.24238
6	0	2.15998	-4.59067	1.33247
1	0	1.52332	-4.98888	-0.68728
1	0	2.80936	-3.87355	3.25845
6	0	-0.6628	1.65114	2.63888
6	0	0.12179	-0.4759	3.90997
6	0	-1.81823	1.9801	1.80407
6	0	-3.755	0.69777	0.69994
1	0	-2.81258	-1.9421	3.04493
1	0	-1.32566	-2.39586	2.18655
1	0	-2.78842	-1.98352	1.27006
1	0	6.92366	-0.35957	-2.67833
1	0	-2.787	0.16394	-3.12793
6	0	-4.81221	-0.54444	-2.86901
6	0	-5.0815	-2.47799	-1.46139
1	0	-3.26539	-3.27671	-0.61485
1	0	2.34333	-5.6375	1.56879
6	0	0.29609	2.648	3.19017
1	0	1.08837	0.01384	4.08295
1	0	0.31846	-1.50514	3.58537
1	0	-0.39941	-0.52905	4.87773
6	0	-2.16137	3.34722	1.33359
1	0	-3.9722	-0.33571	0.40597
1	0	-3.67038	1.27996	-0.22729
1	0	-4.62214	1.07876	1.25894
1	0	-5.24174	0.21706	-3.5189
6	0	-5.63912	-1.49418	-2.26987

1	0	-5.71687	-3.23212	-0.99843
1	0	-0.09323	3.11674	4.10633
1	0	0.49106	3.45092	2.46682
1	0	1.26011	2.18269	3.43177
1	0	-2.8194	3.31752	0.45712
1	0	-1.2629	3.91173	1.04768
1	0	-2.66815	3.92113	2.1239
1	0	-6.71418	-1.47118	-2.44255

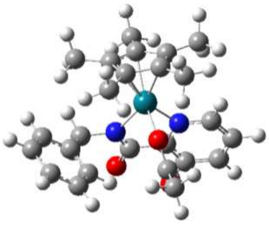

IAb

E(M06 / B1) = -1413.95112369
H(correction)= 0.523261
G(correction)= 0.430800
E(M06 / B2) = -1415.36958443
Imaginary frequencies: 0

6	0	3.17854	-0.63173	0.6072
1	0	3.2186	0.17802	1.33559
6	0	4.17497	-1.59108	0.52728
7	0	2.10887	-0.65791	-0.19234
1	0	5.03074	-1.54001	1.19564
6	0	4.04492	-2.6111	-0.41219
6	0	1.95171	-1.66416	-1.05987
1	0	4.81209	-3.37808	-0.50112
6	0	2.91641	-2.64915	-1.21465
6	0	0.65886	-1.7276	-1.81119
1	0	2.72335	-3.43097	-1.94487
7	0	-0.2145	-0.77842	-1.42139
8	0	0.49164	-2.60083	-2.66394
6	0	-1.51676	-0.79662	-2.05402
45	0	0.42526	0.60994	0.00558
1	0	-1.51965	-1.65052	-2.75008
1	0	-1.66285	0.10296	-2.67502
6	0	-2.66872	-0.92572	-1.0922
6	0	0.77011	2.54655	-0.94149
8	0	-0.15599	-0.55197	1.7126
6	0	-3.85747	-0.22438	-1.3031
6	0	-2.58647	-1.79196	-0.00105
6	0	-0.64397	2.43081	-0.71175
6	0	1.41875	2.56983	0.35353
6	0	1.42888	2.76866	-2.25789
6	0	0.01937	-1.81132	1.96304
1	0	-3.94673	0.42428	-2.17755
6	0	-4.9326	-0.35615	-0.42668
6	0	-3.65733	-1.92588	0.87526
1	0	-1.66824	-2.35924	0.14947
6	0	-0.85366	2.2316	0.68525
6	0	-1.69189	2.58411	-1.75159
6	0	0.43411	2.36463	1.35311
6	0	2.87703	2.78788	0.55163
1	0	0.92006	2.20625	-3.05093
1	0	2.47264	2.43148	-2.24099
1	0	1.42578	3.83178	-2.54256
8	0	0.5647	-2.64613	1.25045
6	0	-0.56723	-2.20001	3.31332
1	0	-5.85163	0.20068	-0.60683
6	0	-4.83025	-1.20084	0.67356
1	0	-3.57928	-2.61135	1.71885
6	0	-2.13748	1.94608	1.3803
1	0	-1.91907	3.65346	-1.87685
1	0	-2.62154	2.07674	-1.47462
1	0	-1.36414	2.20519	-2.72668
6	0	0.62719	2.22838	2.8201
1	0	3.47645	2.1711	-0.13076
1	0	3.19257	2.5676	1.57812
1	0	3.13323	3.83765	0.34792
1	0	-0.43283	-3.27062	3.49362
1	0	-0.08554	-1.62836	4.117
1	0	-1.63542	-1.94794	3.33686
1	0	-5.66539	-1.30497	1.36482
1	0	-1.97978	1.16245	2.13365
1	0	-2.89866	1.56946	0.68429
1	0	-2.53125	2.84121	1.88368
1	0	0.08386	3.01204	3.36636
1	0	1.68509	2.29284	3.10198
1	0	0.24514	1.25029	3.14754

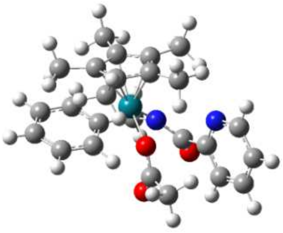

IIAb

E(M06 / B1) = -1413.92120237
H(correction)= 0.522741
G(correction)= 0.431715
E(M06 / B2) = -1415.33481771
Imaginary frequencies: 0

6	0	3.92764	-1.10876	0.71576
1	0	3.98196	-2.1829	0.91216
6	0	4.99823	-0.46425	0.11352
7	0	2.79879	-0.49812	1.09079
1	0	5.88701	-1.02303	-0.17275
6	0	4.89807	0.90873	-0.10186
6	0	2.71629	0.81983	0.88914
1	0	5.70986	1.45834	-0.57613
6	0	3.74932	1.56237	0.30671
6	0	1.52913	1.60413	1.3826
1	0	3.61359	2.63319	0.18543
7	0	0.28964	1.09379	1.23149
8	0	1.77275	2.68532	1.9289
6	0	-0.78989	1.97391	1.64549
45	0	-0.52853	-0.35383	-0.07219
6	0	-1.85858	1.99603	0.58638
1	0	-0.39037	2.98447	1.82841
1	0	-1.23358	1.6354	2.60095
6	0	-0.09711	-2.43941	0.12962
8	0	0.94437	-0.21956	-1.58982

6	0	-1.47922	1.90951	-0.76571
6	0	-3.2179	2.10995	0.89951
6	0	-1.1418	-2.32524	-0.86433
6	0	-0.61159	-1.95472	1.39249
6	0	1.23111	-3.04431	-0.12817
6	0	1.59076	0.79795	-2.06455
6	0	-2.45707	1.89245	-1.77202
1	0	-0.42126	2.00951	-1.0438
1	0	-3.51932	2.19351	1.94527
6	0	-4.17553	2.1244	-0.10686
6	0	-2.26836	-1.74512	-0.2278
6	0	-0.97969	-2.70328	-2.29276
6	0	-1.94714	-1.49984	1.16888
6	0	0.15044	-1.91765	2.66644
1	0	1.11023	-4.08389	-0.4668
1	0	1.85485	-3.03719	0.76989
1	0	1.75207	-2.48463	-0.9179
8	0	1.37099	1.98451	-1.83895
6	0	2.72682	0.37349	-2.97848
6	0	-3.80037	2.00089	-1.45021
1	0	-2.13837	1.83105	-2.8121
1	0	-5.22884	2.22958	0.15253
6	0	-3.5866	-1.45271	-0.84155
1	0	-0.09581	-2.20173	-2.71087
1	0	-1.84978	-2.41016	-2.89076
1	0	-0.84396	-3.78846	-2.40506
6	0	-2.91853	-1.0424	2.19804
1	0	-0.29772	-1.21843	3.381
1	0	1.18037	-1.58343	2.47719
1	0	0.17457	-2.91555	3.12833
1	0	2.3352	-0.1743	-3.84467
1	0	3.28759	1.24896	-3.31947
1	0	3.39466	-0.30705	-2.43366
1	0	-4.55963	2.00773	-2.23081
1	0	-4.31631	-2.21704	-0.53499
1	0	-3.5387	-1.4475	-1.93605
1	0	-3.97522	-0.47857	-0.51671
1	0	-3.6266	-0.31286	1.78684
1	0	-2.41585	-0.57622	3.05345
1	0	-3.50147	-1.8937	2.58072

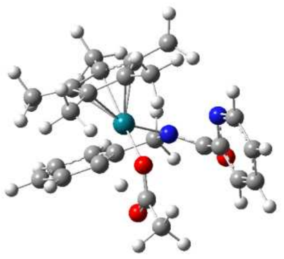

TS(II-III)Ab

E(M06 / B1) = -1413.90227803
H(correction)= 0.516797
G(correction)= 0.425669
E(M06 / B2) = -1415.3176471
Imaginary frequencies: 1 (-1569.4821 cm^{-1})

6	0	3.99422	0.97146	-0.57848
1	0	4.14098	2.04118	-0.74923
6	0	4.95792	0.24605	0.10809
7	0	2.86543	0.44887	-1.06506
1	0	5.85069	0.73755	0.48908
6	0	4.75176	-1.12103	0.27213
6	0	2.67405	-0.86239	-0.89837
1	0	5.48341	-1.73579	0.79513
6	0	3.60471	-1.68699	-0.25735
6	0	1.47251	-1.5492	-1.49219
1	0	3.40383	-2.75465	-0.20012
7	0	0.24833	-1.04595	-1.25311
8	0	1.69613	-2.57267	-2.15072
6	0	-0.8492	-1.8698	-1.73727
45	0	-0.42754	0.42092	0.10495
6	0	-1.95614	-1.90086	-0.72951
1	0	-0.48014	-2.88582	-1.95237
1	0	-1.23618	-1.48807	-2.70214
6	0	0.13902	2.54846	-0.09554
6	0	-0.40579	2.09882	-1.33091
6	0	-1.75878	1.65339	-1.07963
6	0	-2.06236	1.92925	0.30888
6	0	-0.88451	2.42235	0.92694
8	0	1.11587	-0.0076	1.5639
6	0	-1.71109	-1.33135	0.53578
6	0	-3.20864	-2.44161	-1.01974
6	0	1.49496	3.10475	0.14633
6	0	0.31664	2.0423	-2.62966
6	0	-2.74967	1.20045	-2.0952
6	0	-3.40098	1.76603	0.93613
6	0	-0.688	2.79011	2.3554
6	0	1.21514	-1.15687	2.07454
6	0	-2.73645	-1.34576	1.49591
1	0	-3.40068	-2.86462	-2.00742
6	0	-4.21139	-2.43575	-0.05518
1	0	1.42369	4.13366	0.52923
1	0	2.08669	3.11742	-0.7742
1	0	2.03889	2.50354	0.88735
1	0	-0.19424	1.38532	-3.3426
1	0	1.33072	1.64636	-2.48232
1	0	0.38373	3.04381	-3.07901
1	0	-3.40259	0.41221	-1.69745
1	0	-2.25517	0.79946	-2.98765
1	0	-3.38982	2.03559	-2.41688
1	0	-3.98505	2.68888	0.80223
1	0	-3.33289	1.56946	2.01236
1	0	-3.96733	0.94396	0.48317
1	0	0.26244	2.38492	2.72654
1	0	-1.48957	2.39476	2.99062
1	0	-0.66187	3.88192	2.48723
8	0	0.43164	-2.11763	1.83496
6	0	2.33412	-1.40443	3.0509
6	0	-3.97782	-1.89729	1.21306
1	0	-2.53657	-0.92632	2.48526
1	0	-5.18538	-2.86508	-0.2884
1	0	1.9144	-1.54955	4.05376
1	0	2.85527	-2.32917	2.77867
1	0	3.03823	-0.56777	3.06189
1	0	-4.76321	-1.91634	1.96763
1	0	-0.56343	-1.63621	1.09854

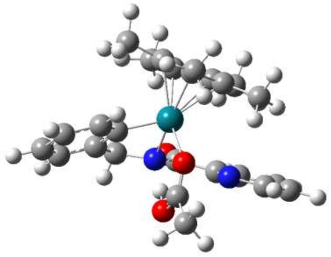

IIIAb

E(M06 / B1) = -1413.94223094
H(correction)= 0.521491
G(correction)= 0.428710
E(M06 / B2) = -1415.35480497
Imaginary frequencies: 0

6	0	3.53004	-0.82997	1.30125
1	0	3.35109	-0.76362	2.37759
6	0	4.82804	-0.78539	0.80005
7	0	2.44815	-0.95154	0.53585
1	0	5.67593	-0.68072	1.47375
6	0	5.00266	-0.89233	-0.5757
6	0	2.62005	-1.03222	-0.7863
45	0	-0.43987	0.4327	0.1528
1	0	6.00019	-0.8745	-1.01213
6	0	3.88196	-1.0191	-1.384
6	0	1.41325	-1.13857	-1.69156
7	0	0.19631	-1.24826	-1.08779
8	0	-0.37075	-0.74956	2.04883
6	0	-2.18385	-0.54214	-0.18576
6	0	0.73371	2.30835	0.89791
1	0	3.94406	-1.09954	-2.46619
8	0	1.59821	-1.1562	-2.91036
6	0	-0.89251	-1.53769	-2.02883
6	0	-0.2032	-1.97439	2.10147
6	0	-2.18105	-1.41561	-1.28058
6	0	-3.32238	-0.42632	0.60659
6	0	-0.71967	2.39971	1.06236
6	0	1.01516	2.17473	-0.4604
6	0	1.68844	2.31072	2.03937
1	0	-0.76169	-2.54097	-2.46728
1	0	-0.85212	-0.83892	-2.8828
8	0	0.01709	-2.74217	1.08073
6	0	-0.24523	-2.70126	3.41082
6	0	-3.33505	-2.13107	-1.59567
6	0	-4.47477	-1.14501	0.28495
1	0	-3.31293	0.21457	1.4917
6	0	-1.31412	2.39903	-0.23394
6	0	-1.39528	2.63329	2.36921
6	0	-0.26229	2.10682	-1.17496
6	0	2.34286	2.12611	-1.12931
1	0	1.69846	3.28514	2.55011
1	0	2.71142	2.08959	1.7139
1	0	1.40379	1.55398	2.78453
1	0	0.12619	-2.17205	0.21713
1	0	-0.46418	-2.01103	4.22854
1	0	-1.00867	-3.48632	3.36829
1	0	0.71758	-3.19631	3.58384
1	0	-3.32945	-2.81524	-2.44625
6	0	-4.48451	-1.98796	-0.82247
1	0	-5.36407	-1.05223	0.90858
6	0	-2.74377	2.64516	-0.56643
1	0	-1.07327	1.89723	3.11788
1	0	-2.48528	2.55864	2.2797
1	0	-1.16219	3.63339	2.76532
6	0	-0.39056	2.02666	-2.6573
1	0	2.35569	1.41584	-1.96682
1	0	3.14845	1.84574	-0.43989
1	0	2.59043	3.11357	-1.54808
1	0	-5.38228	-2.55149	-1.07318
1	0	-2.91159	3.71431	-0.76195
1	0	-3.41133	2.34161	0.24813
1	0	-3.05508	2.08477	-1.45577
1	0	-1.40973	1.75016	-2.95438
1	0	0.29147	1.27332	-3.07564
1	0	-0.15187	2.99208	-3.12939

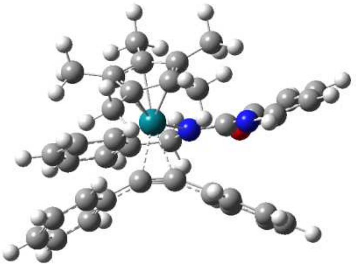

IVAb

E(M06 / B1) = -1723.97033679
H(correction)= 0.658240
G(correction)= 0.550280
E(M06 / B2) = -1725.45692285
Imaginary frequencies: 0

6	0	-4.01412	0.45349	1.14257
1	0	-3.78963	1.00586	2.05913
6	0	-5.32305	0.08908	0.85462
7	0	-2.97113	0.17518	0.35898
1	0	-6.12991	0.33789	1.54105
6	0	-5.56042	-0.59052	-0.33602
6	0	-3.20718	-0.44168	-0.79982
1	0	-6.56805	-0.90227	-0.60785
6	0	-4.49438	-0.84058	-1.18333
6	0	-2.12057	-0.67607	-1.82805
1	0	-4.61704	-1.31927	-2.15032
7	0	-0.8047	-0.69468	-1.53042
8	0	-2.52803	-0.83999	-2.99102
6	0	-0.01758	-0.89579	-2.74448
6	0	1.43507	-0.94707	-2.44096
1	0	-0.23489	-0.09361	-3.47433
1	0	-0.34239	-1.81548	-3.26306
6	0	1.86055	-0.82643	-1.11791
6	0	2.37676	-1.11229	-3.46067
45	0	0.35937	-0.6332	0.26162
6	0	3.22615	-0.86563	-0.83655
1	0	2.02817	-1.20035	-4.49166
6	0	3.73497	-1.1674	-3.17409
6	0	1.23145	1.49094	0.35338
6	0	4.16033	-1.04566	-1.85486
1	0	3.58766	-0.7169	0.18109
1	0	4.45984	-1.29889	-3.97638
6	0	0.01925	1.6379	0.10489

6	0	2.60425	1.91539	0.44672
1	0	5.2229	-1.06336	-1.61215
6	0	-1.17788	2.37864	-0.16971
6	0	3.20649	2.19328	1.68071
6	0	3.34491	2.10748	-0.72952
6	0	-1.78847	3.10763	0.85953
6	0	-1.70937	2.43639	-1.46398
6	0	4.52478	2.62948	1.73874
1	0	2.62413	2.08544	2.59338
6	0	4.65896	2.54789	-0.66502
1	0	2.87845	1.89349	-1.69005
6	0	-2.9274	3.85809	0.60387
1	0	-1.35634	3.07424	1.85969
6	0	-2.85192	3.18554	-1.71077
1	0	-1.22657	1.87765	-2.26381
6	0	5.25635	2.80154	0.56729
1	0	4.97949	2.84396	2.70458
1	0	5.22214	2.68838	-1.58611
6	0	-3.46911	3.88906	-0.67941
1	0	-3.39825	4.4191	1.40997
1	0	-3.26624	3.21416	-2.71691
1	0	6.28937	3.14221	0.61365
1	0	-4.36764	4.47079	-0.87872
1	0	-2.60081	-0.75619	3.18039
6	0	-1.56409	-0.4091	3.23984
6	0	-0.65945	-1.2292	2.38797
1	0	-1.25599	-0.45124	4.29468
1	0	-1.55751	0.6484	2.93571
6	0	0.78639	-1.14637	2.44382
6	0	-0.99099	-2.21378	1.45077
6	0	1.33611	-2.16299	1.61092
6	0	1.53051	-0.31118	3.42458
6	0	0.24558	-2.73066	0.8759
6	0	-2.32513	-2.7843	1.12801
6	0	2.73333	-2.67159	1.64494
1	0	1.05422	0.67081	3.55003
1	0	2.5698	-0.1464	3.11501
1	0	1.54972	-0.79383	4.41404
6	0	0.32805	-3.85328	-0.10086
1	0	-2.51718	-2.79026	0.04799
1	0	-3.14498	-2.2405	1.60874
1	0	-2.36494	-3.8312	1.466
1	0	2.79403	-3.50686	2.35925
1	0	3.44812	-1.90975	1.97935
1	0	3.07122	-3.04307	0.67163
1	0	1.30776	-3.87447	-0.5934
1	0	-0.4281	-3.74101	-0.88819
1	0	0.16817	-4.82853	0.38443

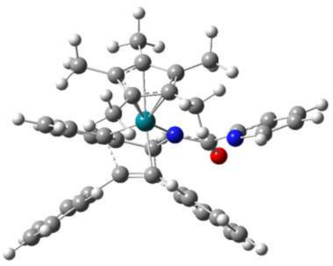

TS(IV-V)Ab

E(M06 / B1) = -1723.95448524

H(correction)= 0.657677

G(correction)= 0.554484

E(M06 / B2) = -1725.43903785

Imaginary frequencies: 1 (-252.4939 cm^{-1})

6	0	-4.27583	0.57878	0.54673
1	0	-4.22733	1.16697	1.46749
6	0	-5.49914	0.11271	0.08537
7	0	-3.11021	0.35342	-0.06572
1	0	-6.41328	0.31813	0.63888
6	0	-5.51411	-0.60913	-1.10603
6	0	-3.13419	-0.30179	-1.22777
1	0	-6.44789	-0.99966	-1.50848
6	0	-4.32273	-0.79268	-1.78561
6	0	-1.90229	-0.43385	-2.09378
1	0	-4.27849	-1.2887	-2.75137
7	0	-0.68759	-0.74883	-1.60303
8	0	-2.1119	-0.26701	-3.30774
6	0	0.30278	-0.75918	-2.67679
45	0	0.20227	-0.73483	0.3643
6	0	1.63929	-1.14277	-2.15727
1	0	0.36324	0.24281	-3.14821
1	0	-0.01614	-1.42544	-3.49327
6	0	-0.05412	-2.81505	1.20173
6	0	0.95686	-2.1812	1.964
6	0	0.22119	1.33243	-0.05265
6	0	1.94397	-0.85481	-0.8212
6	0	2.59827	-1.76417	-2.95956
6	0	-1.28944	-2.09658	1.43777
6	0	0.09715	-4.03626	0.36236
6	0	0.38063	-1.017	2.58945
6	0	2.31999	-2.71884	2.22158
6	0	1.45868	1.04452	-0.20331
6	0	-0.80407	2.33827	-0.04759
6	0	3.21222	-1.16801	-0.32295
1	0	2.34667	-2.00014	-3.99465
6	0	3.84807	-2.09892	-2.45011
6	0	-1.02948	-1.02279	2.33457
6	0	-2.60786	-2.58276	0.94814
1	0	1.1167	-4.11437	-0.03927
1	0	-0.58519	-4.0081	-0.49706
1	0	-0.1152	-4.95718	0.92711
6	0	1.10463	-0.06454	3.47857
1	0	2.26203	-3.46729	3.02645
1	0	3.02071	-1.94423	2.55568
1	0	2.75028	-3.21273	1.34332
6	0	2.74456	1.7229	-0.07646
6	0	-1.17387	2.96514	1.14989
6	0	-1.43825	2.70973	-1.24187
6	0	4.15546	-1.79881	-1.12405
1	0	3.47401	-0.88519	0.69691
1	0	4.58273	-2.59021	-3.08623
6	0	-2.01154	-0.07182	2.92694
1	0	-2.59052	-2.75809	-0.13508
1	0	-3.41844	-1.87696	1.1547
1	0	-2.84753	-3.54239	1.43073
1	0	0.57815	0.89733	3.53913
1	0	2.11703	0.13924	3.103
1	0	1.20346	-0.45478	4.50301
6	0	3.00071	2.46018	1.08564

6	0	3.68978	1.72778	-1.10893
6	0	-2.16987	3.93445	1.15541
1	0	-0.65818	2.69235	2.07197
6	0	-2.42921	3.68012	-1.22793
1	0	-1.1731	2.20077	-2.16877
1	0	5.13657	-2.0406	-0.71665
1	0	-3.0097	-0.52178	2.98965
1	0	-1.71381	0.211	3.94549
1	0	-2.10506	0.84567	2.32753
6	0	4.18265	3.18084	1.21791
1	0	2.25403	2.46472	1.88008
6	0	4.86144	2.46024	-0.97953
1	0	3.48976	1.15609	-2.01444
6	0	-2.80336	4.29031	-0.03187
1	0	-2.4474	4.41947	2.09036
1	0	-2.92265	3.95522	-2.15852
6	0	5.11465	3.18207	0.18554
1	0	4.37259	3.74758	2.12804
1	0	5.58421	2.46797	-1.79362
1	0	-3.58537	5.04788	-0.02699
1	0	6.03897	3.74892	0.285

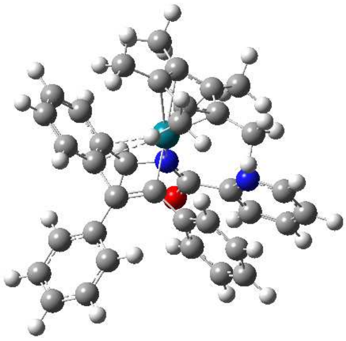

VAb

E(M06 / B1) = -1724.01299798
H(correction)= 0.659592
G(correction)= 0.555042
E(M06 / B2) = -1725.49310124
Imaginary frequencies: 0

6	0	4.24683	0.33434	-0.19367
1	0	4.38254	0.06295	-1.24363
6	0	5.27703	0.9762	0.48361
7	0	3.07079	0.01978	0.34913
1	0	6.21052	1.20693	-0.02576
6	0	5.07154	1.30353	1.81789
6	0	2.86416	0.37262	1.62265
1	0	5.8504	1.79692	2.39788
6	0	3.8469	1.00474	2.39258
6	0	1.5186	0.21265	2.29485
1	0	3.60234	1.25871	3.41979
7	0	0.48935	-0.40528	1.68861
8	0	1.40155	0.71673	3.42393
6	0	-0.75272	-0.22132	2.41853
45	0	-0.16757	-1.18521	-0.15519
6	0	-1.92963	-0.73894	1.61947
1	0	-0.90753	0.84701	2.65859
1	0	-0.71621	-0.73774	3.39097
6	0	1.25226	-2.9157	-0.49327
6	0	-0.48324	0.82968	-0.50507
6	0	-2.44907	-0.03189	0.50335
6	0	-2.64985	-1.84783	2.10425
6	0	-0.01738	-3.48487	-0.30711
6	0	1.18913	-2.02023	-1.64932
6	0	2.44145	-3.18904	0.35816
6	0	-1.71842	1.13843	-0.06972
6	0	0.56299	1.74804	-0.94306
6	0	-3.68062	-0.43335	-0.04683
1	0	-2.25289	-2.37995	2.97005
6	0	-3.85733	-2.22985	1.54947
6	0	-0.89109	-2.94555	-1.33254
6	0	-0.40464	-4.44541	0.76416
6	0	-0.14227	-2.09084	-2.18561
6	0	2.34344	-1.39507	-2.34406
1	0	2.23142	-2.95526	1.41101
1	0	3.3024	-2.58265	0.06288
1	0	2.71993	-4.25116	0.2996
6	0	-2.38412	2.45681	-0.07192
6	0	1.00248	1.80833	-2.2714
6	0	1.17359	2.58874	0.00009
6	0	-4.38349	-1.50427	0.47223
1	0	-4.08486	0.14342	-0.87943
1	0	-4.40811	-3.07315	1.96397
6	0	-2.30732	-3.33978	-1.53665
1	0	-1.4894	-4.44835	0.92968
1	0	0.07364	-4.18848	1.71914
1	0	-0.10777	-5.4751	0.51283
6	0	-0.64266	-1.44427	-3.43367
1	0	3.28308	-1.61338	-1.82884
1	0	2.41857	-1.7871	-3.36948
1	0	2.24326	-0.30254	-2.40583
6	0	-3.31928	2.76426	0.92647
6	0	-2.11286	3.42884	-1.04457
6	0	2.03523	2.66319	-2.64447
1	0	0.50549	1.18721	-3.01838
6	0	2.21022	3.43367	-0.37167
1	0	0.82769	2.56218	1.03459
1	0	-5.34483	-1.78477	0.04343
1	0	-2.35673	-4.36242	-1.94022
1	0	-2.81473	-2.67235	-2.24291
1	0	-2.8757	-3.32968	-0.59764
1	0	0.11083	-0.76911	-3.85646
1	0	-1.54865	-0.85057	-3.24645
1	0	-0.88459	-2.19129	-4.204
6	0	-3.949	4.0026	0.96171
1	0	-3.54308	2.02181	1.69389
6	0	-2.74393	4.6652	-1.00956
1	0	-1.39946	3.20631	-1.83666
6	0	2.65163	3.47004	-1.69293
1	0	2.35693	2.70023	-3.68485
1	0	2.6765	4.07291	0.37689
6	0	-3.66465	4.9596	-0.00713
1	0	-4.66474	4.22069	1.75324
1	0	-2.51967	5.40443	-1.77747
1	0	3.46608	4.13286	-1.98114
1	0	-4.15874	5.92966	0.01724

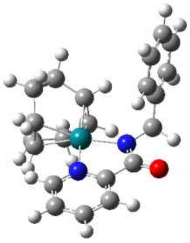

complex B

E(M06 / B1) = -1107.6074958
H(correction)= 0.420312
G(correction)= 0.345186
E(M06 / B2) = -1108.93040443
Imaginary frequencies: 0

45	0	0.62846	-0.60262	-0.14331
6	0	-0.43752	-2.02509	-1.35605
6	0	1.92627	-2.31766	0.14711
6	0	1.30655	-1.97315	1.35713
6	0	-1.09082	-1.94232	-0.12273
7	0	2.08605	0.90973	0.25755
7	0	-0.31428	1.06624	-0.93798
1	0	-0.84542	-1.43372	-2.18001
6	0	0.51203	-3.11355	-1.79014
6	0	1.52769	-3.50269	-0.7096
1	0	2.94346	-1.9493	-0.01792
1	0	1.88868	-1.38247	2.0728
6	0	0.11684	-2.66459	1.97588
6	0	-0.9866	-2.99285	0.96346
1	0	-1.95441	-1.27575	-0.05864
6	0	3.28516	0.78633	0.83665
6	0	1.70593	2.11404	-0.2047
6	0	-1.64914	1.15863	-1.49663
6	0	0.34567	2.23724	-0.84004
1	0	1.05537	-2.73883	-2.66897
1	0	-0.04892	-4.00004	-2.13452
1	0	1.13263	-4.30718	-0.07399
1	0	2.42206	-3.92136	-1.19005
1	0	-0.29456	-1.98417	2.73508
1	0	0.42951	-3.5741	2.51843
1	0	-0.8218	-3.98041	0.51096
1	0	-1.9483	-3.06356	1.48819
1	0	3.55209	-0.20707	1.19625
6	0	4.15807	1.85501	0.97751
6	0	2.52189	3.23239	-0.09662
1	0	-1.8049	0.35273	-2.23118
1	0	-1.72605	2.10745	-2.05025
6	0	-2.75644	1.1047	-0.47184
8	0	-0.03712	3.35602	-1.18943
1	0	5.12416	1.70558	1.45318
6	0	3.76712	3.10215	0.50007
1	0	2.13864	4.17131	-0.4888
6	0	-2.60188	1.67418	0.79253
6	0	-3.97698	0.50697	-0.79145
1	0	4.42882	3.96121	0.59405
1	0	-1.65115	2.13804	1.05322
6	0	-3.64604	1.65259	1.71104
6	0	-5.02422	0.48425	0.1245
1	0	-4.10325	0.05031	-1.77537
1	0	-3.51016	2.10488	2.69287
6	0	-4.86144	1.05877	1.3811
1	0	-5.96826	0.01092	-0.14287
1	0	-5.67741	1.04144	2.10208

COD

E(M06 / B1) = -311.766659673
H(correction)= 0.188764
G(correction)= 0.149854
E(M06 / B2) = -311.847506425
Imaginary frequencies: 0

6	0	-1.73844	0.62448	0.23496
6	0	-1.73844	-0.62448	-0.23496
6	0	-0.5658	1.51911	0.5221
1	0	-2.71682	1.0755	0.41589
1	0	-2.71682	-1.0755	-0.41589
6	0	-0.5658	-1.51911	-0.5221
6	0	0.5658	1.51911	-0.5221
1	0	-0.13881	1.29858	1.51426
1	0	-0.95212	2.54453	0.60237
1	0	-0.13881	-1.29858	-1.51426
1	0	-0.95212	-2.54453	-0.60237
6	0	0.5658	-1.51911	0.5221
6	0	1.73844	0.62448	-0.23496
1	0	0.13881	1.29858	-1.51426
1	0	0.95212	2.54453	-0.60237
1	0	0.13881	-1.29858	1.51426
1	0	0.95212	-2.54453	0.60237
6	0	1.73844	-0.62448	0.23496
1	0	2.71682	1.0755	-0.41589
1	0	2.71682	-1.0755	0.41589

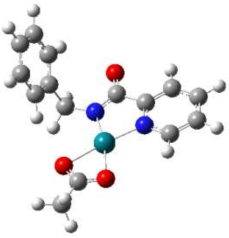

modB

E(M06 / B1) = -1024.21939962
H(correction)= 0.286011
G(correction)= 0.215838
E(M06 / B2) = -1025.53245113
Imaginary frequencies: 0

45	0	-0.77648	0.85499	-0.22861
7	0	0.26536	-0.73502	-0.94381
7	0	-1.99295	-0.6636	0.21984
8	0	0.35899	2.68022	-0.57131
6	0	1.5764	-0.60739	-1.54136
6	0	-0.21499	-1.97018	-0.76425
6	0	-3.19165	-0.54931	0.82295

6	0	-1.55353	-1.90838	-0.10613
6	0	-0.55374	3.3627	-0.01712
1	0	1.60443	0.3518	-2.07958
1	0	1.73357	-1.42415	-2.2644
6	0	2.66733	-0.63596	-0.50162
8	0	0.30194	-3.0596	-1.07462
1	0	-3.48979	0.47062	1.0644
6	0	-3.98433	-1.6473	1.11723
6	0	-2.29663	-3.04558	0.16363
8	0	-1.60135	2.82308	0.43095
6	0	-0.38702	4.8575	0.1116
6	0	3.33074	-1.826	-0.19955
6	0	2.99331	0.52224	0.20939
1	0	-4.94594	-1.49604	1.60525
6	0	-3.53458	-2.92531	0.78294
1	0	-1.85982	-3.99847	-0.13032
1	0	-0.42648	5.14437	1.16981
1	0	0.56284	5.18494	-0.32254
1	0	-1.21784	5.36743	-0.3918
1	0	3.03632	-2.7313	-0.7306
6	0	4.3181	-1.85748	0.78116
6	0	3.97818	0.49025	1.19146
1	0	2.4488	1.44319	-0.0094
1	0	-4.13808	-3.80422	1.00663
1	0	4.83013	-2.79385	1.00648
6	0	4.64808	-0.69764	1.47709
1	0	4.22449	1.39965	1.74068
1	0	5.42154	-0.72041	2.24569

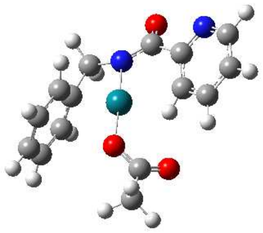

IBa

E(M06 / B1) = -1024.17670571
H(correction)= 0.283671
G(correction)= 0.212092
E(M06 / B2) = -1025.49263751
Imaginary frequencies: 0

45	0	0.28414	-0.02611	0.18582
7	0	-0.67699	-1.78753	0.01911
8	0	1.36704	1.77476	0.37224
6	0	0.37434	-2.72452	-0.25359
6	0	-1.95975	-1.94106	-0.32714
6	0	1.05917	2.76089	-0.38303
1	0	0.318	-3.12778	-1.28061
1	0	0.38454	-3.59337	0.43172
6	0	1.59511	-1.83365	-0.07333
6	0	-2.66604	-0.61434	-0.14738
8	0	-2.51045	-2.96819	-0.72776
8	0	0.15413	2.81224	-1.22492
6	0	1.94016	3.98623	-0.15107
6	0	1.85496	-1.27144	1.21377
6	0	2.56077	-1.65712	-1.09612
6	0	-1.96839	0.57067	-0.4655
7	0	-3.89521	-0.60904	0.36933
1	0	2.99905	3.70093	-0.18012
1	0	1.73956	4.75914	-0.90089
1	0	1.74789	4.39656	0.8493
6	0	3.07111	-0.5806	1.43583
1	0	1.25255	-1.58806	2.06825
6	0	3.7309	-0.97324	-0.85212
1	0	2.36032	-2.08421	-2.08
6	0	-2.55515	1.79803	-0.17087
1	0	-1.07751	0.58255	-1.14521
6	0	-4.4459	0.57928	0.62904
1	0	3.26388	-0.1556	2.42033
6	0	3.98806	-0.42755	0.42203
1	0	4.45998	-0.84348	-1.65207
6	0	-3.82066	1.80232	0.40754
1	0	-2.00066	2.70365	-0.4151
1	0	-5.45438	0.5525	1.05276
1	0	4.91255	0.12163	0.59843
1	0	-4.32455	2.73261	0.66728

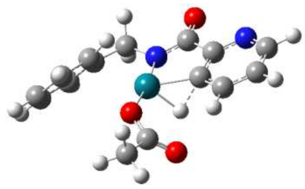

TS(I-II)Ba

E(M06 / B1) = -1024.16640277
H(correction)= 0.279356
G(correction)= 0.208904
E(M06 / B2) = -1025.4850604
Imaginary frequencies: 1 (-522.7850 cm^{-1})

45	0	0.128	0.21175	-0.03214
7	0	-0.1117	-1.80254	-0.22121
6	0	-1.89083	0.07515	-0.10582
1	0	-0.90115	0.48829	-1.21741
8	0	0.59383	2.22521	0.31753
6	0	1.05211	-2.64278	-0.11572
6	0	-1.33439	-2.35237	-0.08768
6	0	-2.36383	-1.25517	-0.02535
6	0	-2.82463	1.10667	0.00531
6	0	0.06362	3.24171	-0.26051
1	0	1.19582	-3.27162	-1.01275
1	0	0.95316	-3.34971	0.73082
6	0	2.23606	-1.72934	0.06205
8	0	-1.57795	-3.55734	-0.00151
7	0	-3.64501	-1.57425	0.16415
6	0	-4.1607	0.77369	0.20762
1	0	-2.48714	2.13729	-0.10715
8	0	-0.86395	3.25199	-1.07115
6	0	0.71073	4.55868	0.16523
6	0	2.1514	-0.6748	0.9926
6	0	3.37752	-1.82173	-0.73012
6	0	-4.51532	-0.57067	0.27429
1	0	-4.92264	1.54804	0.30177
1	0	1.78228	4.54347	-0.07188
1	0	0.23483	5.40503	-0.34121

1	0	0.62613	4.68556	1.25224
6	0	3.16515	0.27986	1.08353
1	0	1.32	-0.65488	1.71648
6	0	4.40572	-0.88972	-0.61114
1	0	3.44529	-2.62112	-1.46914
1	0	-5.56109	-0.85505	0.4263
1	0	3.04841	1.11425	1.7724
6	0	4.29629	0.17357	0.28034
1	0	5.28838	-0.97745	-1.24487
1	0	5.08547	0.92158	0.3434

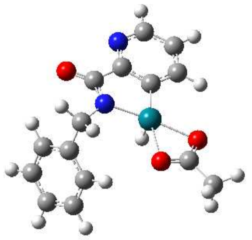

IIBa

E(M06 / B1) = -1024.19197306
H(correction)= 0.282001
G(correction)= 0.211610
E(M06 / B2) = -1025.50814015
Imaginary frequencies: 0

45	0	0.65657	0.84029	-0.24564
7	0	-0.15793	-0.82275	-1.03591
6	0	1.99972	-0.51185	0.26547
1	0	0.05883	0.51836	1.12459
8	0	1.32938	2.81365	0.4248
6	0	-1.49911	-0.7704	-1.57668
6	0	0.40828	-2.03596	-0.81101
6	0	1.7251	-1.83925	-0.11282
6	0	3.19786	-0.2701	0.93214
6	0	0.31092	3.39614	-0.05876
1	0	-1.63164	-1.61568	-2.26961
1	0	-1.60294	0.16496	-2.14889
6	0	-2.55781	-0.82006	-0.50463
8	0	-0.0634	-3.12854	-1.13031
7	0	2.53409	-2.87491	0.11311
6	0	4.05762	-1.34197	1.17158
1	0	3.44963	0.74241	1.25487
8	0	-0.58079	2.7753	-0.69374
6	0	0.16119	4.88076	0.17475
6	0	-3.0449	0.35629	0.06982
6	0	-3.03076	-2.04911	-0.03942
6	0	3.67974	-2.61267	0.74559
1	0	5.00885	-1.19811	1.6862
1	0	1.13904	5.3527	0.31701
1	0	-0.37292	5.34828	-0.65931
1	0	-0.43038	5.04309	1.08538
6	0	-4.00313	0.30319	1.07819
1	0	-2.64472	1.31501	-0.26841
6	0	-3.9885	-2.10176	0.96809
1	0	-2.61163	-2.95924	-0.46875
1	0	4.33737	-3.46793	0.92833
1	0	-4.37711	1.2282	1.51816
6	0	-4.4824	-0.92511	1.52614
1	0	-4.35083	-3.06721	1.3227

1	0	-5.23476	-0.96617	2.31442

TS(IV-V)Ba

E(M06 / B1) = -1563.20831399
H(correction)= 0.485360
G(correction)= 0.388793
E(M06 / B2) = -1564.67059217
Imaginary frequencies: 1 (-346.0721 cm^{-1})

7	0	-1.34961	-1.91901	-0.69566
6	0	-2.1514	-1.73029	-1.87727
6	0	-1.81319	-2.70271	0.29664
1	0	-2.77733	-2.62508	-2.02546
1	0	-1.48175	-1.63007	-2.74736
6	0	-3.02694	-0.50663	-1.7616
6	0	-0.84532	-2.63442	1.44061
8	0	-2.85295	-3.36913	0.29836
6	0	-2.78623	0.63969	-2.5187
6	0	-4.06375	-0.48615	-0.82041
6	0	0.18745	-1.69961	1.31526
7	0	-0.97583	-3.43167	2.50423
6	0	-3.56293	1.78496	-2.35107
1	0	-1.96739	0.63323	-3.24114
6	0	-4.84229	0.65376	-0.65383
1	0	-4.23273	-1.38301	-0.22176
6	0	1.14967	-1.6012	2.30793
6	0	-0.0613	-3.3182	3.46545
1	0	-3.35297	2.67489	-2.94429
6	0	-4.59561	1.79446	-1.41946
1	0	-5.64846	0.65502	0.08025
6	0	1.01311	-2.43338	3.41732
1	0	1.99056	-0.91702	2.20772
1	0	-0.18523	-3.97817	4.32875
1	0	-5.20257	2.68981	-1.28301
1	0	1.73904	-2.40215	4.22978
45	0	0.2046	-0.56663	-0.31375
1	0	-0.88727	0.36388	0.4189
6	0	1.43725	1.08473	-0.08934
8	0	1.36051	-1.85812	-1.5199
6	0	0.25636	1.4368	0.31978
6	0	2.77462	1.56315	-0.35342
6	0	2.42437	-2.47372	-1.15029
6	0	-0.5118	2.52946	0.89395
6	0	3.003	2.91553	-0.66117
6	0	3.86436	0.68277	-0.30527
8	0	3.05554	-2.32954	-0.09972
6	0	2.90154	-3.48571	-2.18615
6	0	-1.91188	2.53146	0.89998
6	0	0.16401	3.63703	1.43051

6	0	4.28912	3.37704	-0.90445
1	0	2.15272	3.59638	-0.7172
6	0	5.15103	1.15845	-0.53125
1	0	3.67231	-0.37234	-0.0895
1	0	3.0174	-3.00175	-3.16393
1	0	3.85076	-3.93592	-1.87792
1	0	2.14454	-4.27077	-2.30531
6	0	-2.61547	3.61394	1.41122
1	0	-2.45244	1.67913	0.48434
6	0	-0.54321	4.71356	1.94737
1	0	1.25386	3.63292	1.44404
6	0	5.37021	2.50005	-0.83227
1	0	4.45093	4.42707	-1.14827
1	0	5.99145	0.46672	-0.48198
6	0	-1.93694	4.70976	1.9369
1	0	-3.70484	3.59444	1.39057
1	0	-0.00237	5.56147	2.36685
1	0	6.38091	2.8638	-1.01713
1	0	-2.48998	5.55747	2.33995

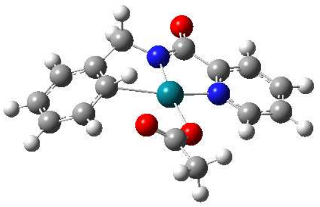

IBb

E(M06 / B1) = -1024.2091306
H(correction)= 0.284892
G(correction)= 0.214932
E(M06 / B2) = -1025.52585121
Imaginary frequencies: 0

45	0	0.00795	0.33008	0.20088
7	0	-0.32843	-1.63792	0.47754
7	0	-1.99039	0.23365	-0.14363
8	0	0.03242	2.42128	-0.13631
6	0	0.7791	-2.50418	0.7972
6	0	-1.56129	-2.12343	0.32137
6	0	-2.79423	1.25825	-0.46396
6	0	-2.50838	-1.01641	-0.04614
6	0	1.00699	3.15352	0.25385
1	0	0.62599	-3.51297	0.38039
1	0	0.86882	-2.63	1.89447
6	0	2.0246	-1.86533	0.2426
8	0	-1.95863	-3.29503	0.44153
1	0	-2.29271	2.22444	-0.52291
6	0	-4.14974	1.07886	-0.69987
6	0	-3.85603	-1.25905	-0.26741
8	0	2.05133	2.78747	0.80351
6	0	0.78213	4.6409	-0.01584
6	0	2.18751	-0.46644	0.3425
6	0	2.97182	-2.59687	-0.464
1	0	-4.76806	1.93655	-0.9593
6	0	-4.69247	-0.20197	-0.60121
1	0	-4.19605	-2.28798	-0.16687
1	0	-0.08855	4.99386	0.55271
1	0	1.6624	5.22668	0.27008
1	0	0.5571	4.80464	-1.07758

1	0	1.64944	0.14363	1.13758
6	0	3.27955	0.1628	-0.25612
6	0	4.06964	-1.96791	-1.05334
1	0	2.83402	-3.67359	-0.57861
1	0	-5.75383	-0.36874	-0.78365
1	0	3.35313	1.24425	-0.14581
6	0	4.21914	-0.58859	-0.9586
1	0	4.80262	-2.56221	-1.60045
1	0	5.06867	-0.09682	-1.43329

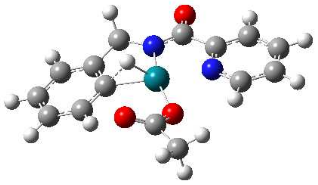

TS(I-II)Bb

E(M06 / B1) = -1024.19249926
H(correction)= 0.279821
G(correction)= 0.210364
E(M06 / B2) = -1025.51264934
Imaginary frequencies: 1 (-502.7683 cm^{-1})

45	0	0.09666	0.25739	0.16502
7	0	-0.28846	-1.71194	0.31485
7	0	-2.068	0.20042	-0.11503
8	0	0.03505	2.33743	-0.1564
6	0	1.98059	-0.4392	0.0023
6	0	0.82962	-2.62579	0.39079
6	0	-1.53143	-2.1741	0.17772
6	0	-2.91175	1.21495	-0.30504
6	0	-2.53264	-1.06022	-0.02639
6	0	0.88074	3.2374	0.20029
6	0	2.07104	-1.84955	0.04974
6	0	3.13056	0.30297	-0.28244
1	0	0.67587	-3.48006	-0.29118
1	0	0.90559	-3.06525	1.40394
8	0	-1.91633	-3.35461	0.18331
1	0	-2.43708	2.19623	-0.3688
6	0	-4.28299	1.01613	-0.41472
6	0	-3.89102	-1.33635	-0.13119
8	0	1.95909	3.08452	0.77021
6	0	0.41777	4.64558	-0.17897
6	0	3.27935	-2.47845	-0.24191
1	0	3.06539	1.39068	-0.2406
6	0	4.33142	-0.34011	-0.57806
1	0	-4.94822	1.8632	-0.5704
6	0	-4.7756	-0.28421	-0.32589
1	0	-4.19976	-2.37668	-0.05609
1	0	-0.58052	4.84084	0.23426
1	0	1.12322	5.39771	0.1898
1	0	0.33368	4.72973	-1.27052
6	0	4.41026	-1.73062	-0.56121
1	0	3.33215	-3.57037	-0.22606
1	0	5.21789	0.25192	-0.81275
1	0	-5.84595	-0.47229	-0.41064
1	0	5.35172	-2.23313	-0.78806
1	0	1.09362	0.39624	1.35543

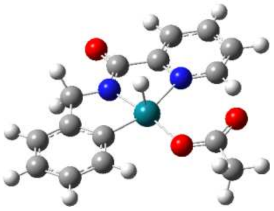

IIBb

E(M06 / B1) = -1024.20249518
H(correction)= 0.281559
G(correction)= 0.210417
E(M06 / B2) = -1025.52133336
Imaginary frequencies: 0

45	0	0.19716	0.27202	0.01781
7	0	-0.02878	-1.72075	-0.0119
1	0	0.32872	0.21644	1.54019
8	0	0.68691	2.3271	-0.12395
6	0	2.12357	-0.20977	0.03139
6	0	1.17532	-2.52257	0.07446
6	0	-1.22269	-2.3118	-0.01979
6	0	-0.10874	3.31849	0.01788
6	0	2.36462	-1.60254	0.02555
6	0	3.21679	0.66085	0.00018
1	0	1.2021	-3.26443	-0.74394
1	0	1.17109	-3.12228	1.00446
6	0	-2.363	-1.32115	-0.04369
8	0	-1.45579	-3.53073	-0.01175
8	0	-1.32018	3.30169	0.25284
6	0	0.60274	4.66252	-0.13351
6	0	3.67101	-2.08626	-0.02829
1	0	3.02273	1.7339	0.00691
6	0	4.51912	0.16761	-0.05106
7	0	-2.07891	-0.00363	-0.03497
6	0	-3.66978	-1.79456	-0.05728
1	0	1.1246	4.70523	-1.09793
1	0	-0.10897	5.49168	-0.05998
1	0	1.36856	4.76656	0.64593
6	0	4.75047	-1.20678	-0.06508
1	0	3.84482	-3.16596	-0.04133
1	0	5.36316	0.85983	-0.0803
6	0	-3.08495	0.87714	-0.02901
6	0	-4.71489	-0.88099	-0.06136
1	0	-3.81268	-2.87273	-0.06078
1	0	5.76956	-1.59411	-0.10575
1	0	-2.76841	1.92162	0.00319
6	0	-4.41872	0.47713	-0.04504
1	0	-5.75008	-1.22307	-0.07279
1	0	-5.2062	1.22872	-0.04073

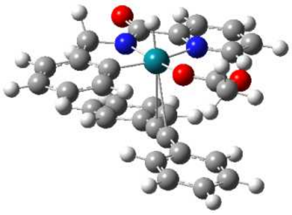

IIIBb

E(M06 / B1) = -1563.24159061
H(correction)= 0.487495
G(correction)= 0.392997
E(M06 / B2) = -1564.7047563
Imaginary frequencies: 0

Rh	0.10988	0.17622	-1.06358
N	-1.7143	1.01957	-0.92762
H	0.0202	0.49698	-2.56393
C	0.70345	2.06603	-0.91345
O	2.11197	-0.45826	-1.41215
C	1.02851	-0.39815	1.62987
C	-1.76364	2.46537	-0.95288
C	-2.84262	0.31665	-1.00346
C	-0.36175	2.98896	-0.79645
C	2.01536	2.54103	-0.82533
C	2.53705	-1.66301	-1.39925
C	-0.16456	-0.20408	1.7858
C	2.43414	-0.63337	1.65445
H	-2.43836	2.84163	-0.16119
H	-2.21182	2.81775	-1.90206
C	-2.59585	-1.17318	-1.00702
O	-4.00147	0.75964	-1.04196
C	-0.09621	4.33504	-0.55022
H	2.83344	1.82754	-0.94296
C	2.27029	3.892	-0.58556
O	1.89741	-2.69791	-1.17115
C	4.03069	-1.76066	-1.69766
C	-1.53442	0.03323	2.08823
C	2.92795	-1.94259	1.73732
C	3.33375	0.44203	1.65369
N	-1.32615	-1.61773	-1.06562
C	-3.67874	-2.04341	-0.92884
C	1.21619	4.79198	-0.44286
H	-0.92834	5.03703	-0.44569
H	3.3006	4.24688	-0.51262
H	4.58955	-1.10434	-1.01633
H	4.38224	-2.79249	-1.5859
H	4.23027	-1.41323	-2.71985
C	-2.42879	-1.03511	2.23515
C	-2.00671	1.34664	2.23267
C	4.29396	-2.16726	1.83904
H	2.22655	-2.77253	1.69258
C	4.69855	0.20786	1.75107
H	2.94094	1.45326	1.55723
C	-1.09244	-2.93381	-1.02432
C	-3.43899	-3.40921	-0.88801
H	-4.67304	-1.60334	-0.8858
H	1.41424	5.8472	-0.2496
C	-3.765	-0.79277	2.52689
H	-2.06414	-2.05319	2.10078
C	-3.34088	1.579	2.52942
H	-1.31253	2.17167	2.0729
C	5.18272	-1.09494	1.85172
H	4.6685	-3.18856	1.89504
H	5.39014	1.04953	1.74525
H	-0.03374	-3.19897	-1.06566
C	-2.12447	-3.86378	-0.93228
H	-4.2671	-4.11541	-0.81918
C	-4.2245	0.51152	2.67664
H	-4.45534	-1.63062	2.61931
H	-3.70213	2.60253	2.62033

H	6.25483	-1.27469	1.92773
H	-1.89185	-4.92682	-0.89889
H	-5.2761	0.69967	2.88819

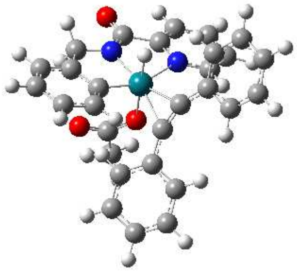

IVBb

E(M06 / B1) = -1563.23092033
H(correction)= 0.486576
G(correction)= 0.390818
E(M06 / B2) = -1564.69535929
Imaginary frequencies: 0

45	0	-0.10558	-0.36882	0.38934
7	0	0.00679	-2.32547	0.84138
1	0	-0.79867	-0.1721	1.78364
6	0	1.43609	-0.26221	1.68315
8	0	0.70551	-0.88068	-1.65563
6	0	-0.8306	1.6594	0.25196
6	0	1.14949	-2.74953	1.62341
6	0	-0.80088	-3.22229	0.28295
6	0	1.84724	-1.52925	2.14647
6	0	2.05859	0.87556	2.19493
6	0	1.82638	-1.46521	-1.83877
6	0	0.2771	1.64032	-0.34526
6	0	-2.03635	2.27249	0.73262
1	0	1.83286	-3.33003	0.98
1	0	0.83669	-3.42305	2.44137
6	0	-1.86714	-2.60006	-0.59128
8	0	-0.75474	-4.45476	0.40595
6	0	2.88543	-1.62366	3.06947
1	0	1.72902	1.86522	1.87608
6	0	3.10272	0.76882	3.11464
8	0	2.68499	-1.74042	-0.99264
6	0	2.12891	-1.75117	-3.31035
6	0	1.37971	2.17007	-1.09557
6	0	-3.026	1.56065	1.4262
6	0	-2.25063	3.63972	0.47841
7	0	-1.92071	-1.26298	-0.70622
6	0	-2.7506	-3.42728	-1.27859
6	0	3.52296	-0.48253	3.55063
1	0	3.19935	-2.61132	3.41653
1	0	3.58401	1.67227	3.49304
1	0	2.51286	-0.82514	-3.76337
1	0	2.89617	-2.52747	-3.40695
1	0	1.2237	-2.036	-3.85901
6	0	1.22637	3.42149	-1.71547
6	0	2.60526	1.49758	-1.21161
6	0	-4.18687	2.19602	1.84828
1	0	-2.85999	0.50302	1.62547
6	0	-3.41099	4.26865	0.90368
1	0	-1.47968	4.19788	-0.05321
6	0	-2.82287	-0.71452	-1.51651
6	0	-3.70194	-2.85433	-2.10913
1	0	-2.64381	-4.50032	-1.1366
1	0	4.33524	-0.57261	4.27281
6	0	2.26834	3.98817	-2.43629
1	0	0.26827	3.93593	-1.63078
6	0	3.64148	2.07277	-1.93604
1	0	2.73216	0.52174	-0.74156
6	0	-4.38805	3.5492	1.59009
1	0	-4.94387	1.62566	2.38564
1	0	-3.55554	5.32937	0.69915
1	0	-2.8035	0.37437	-1.5864
6	0	-3.7406	-1.46922	-2.23755
1	0	-4.4043	-3.47943	-2.66033
6	0	3.48173	3.31341	-2.54925
1	0	2.13236	4.95893	-2.91306
1	0	4.58601	1.5371	-2.02215
1	0	-5.30071	4.04272	1.92243
1	0	-4.46305	-0.97744	-2.88593
1	0	4.30143	3.75514	-3.1158

TS(IV-V)Bb

E(M06 / B1) = -1563.21480284
H(correction)= 0.484389
G(correction)= 0.388053
E(M06 / B2) = -1564.68002923
Imaginary frequencies: 1 (-581.5562 cm^{-1})

45	0	0.3803	-0.22143	-0.11605
7	0	1.94413	-1.47963	-0.52553
1	0	-0.56657	-0.76384	-1.34279
6	0	-0.19417	-1.774	1.00919
8	0	1.57809	0.77418	1.36469
7	0	1.65467	0.98922	-1.60637
6	0	-1.2481	1.07904	0.0389
6	0	1.98028	-2.7304	0.19829
6	0	2.96762	-1.08854	-1.27133
6	0	0.69183	-2.87079	0.95738
6	0	-1.35895	-1.86706	1.76743
6	0	2.31235	0.28383	2.30432
6	0	2.77671	0.29429	-1.86801
6	0	1.50374	2.20655	-2.12342
6	0	-1.63777	0.15725	-0.77591
6	0	-1.53381	2.26987	0.78422
1	0	2.82138	-2.71922	0.91204
1	0	2.14969	-3.58256	-0.48564
8	0	4.01161	-1.71208	-1.52382
6	0	0.37429	-4.03407	1.65336
1	0	-2.04453	-1.01917	1.81683
6	0	-1.6581	-3.03742	2.46808
8	0	2.51404	-0.89016	2.59476

6	0	2.97871	1.39389	3.1236
6	0	3.78027	0.81959	-2.6787
1	0	0.57649	2.72224	-1.86319
6	0	2.45775	2.79958	-2.9416
6	0	-2.81343	-0.48143	-1.32987
6	0	-2.3777	3.27452	0.27881
6	0	-0.94847	2.44934	2.04899
6	0	-0.79499	-4.12522	2.40605
1	0	1.06415	-4.88079	1.6134
1	0	-2.57224	-3.09436	3.0614
1	0	2.2186	2.07532	3.53091
1	0	3.56821	0.96751	3.94229
1	0	3.62901	1.99667	2.47691
6	0	3.61886	2.08444	-3.22283
1	0	4.65793	0.19948	-2.84589
1	0	2.29236	3.79784	-3.34241
6	0	-2.79379	-1.80143	-1.79931
6	0	-4.02407	0.22936	-1.37621
6	0	-2.64296	4.41533	1.02471
1	0	-2.80684	3.15138	-0.71663
6	0	-1.23389	3.58466	2.79478
1	0	-0.25386	1.6874	2.39932
1	0	-1.02711	-5.04401	2.94605
1	0	4.39085	2.51535	-3.86041
6	0	-3.94558	-2.38759	-2.30724
1	0	-1.8629	-2.36629	-1.73692
6	0	-5.17025	-0.35914	-1.88909
1	0	-4.05033	1.2492	-0.99232
6	0	-2.07897	4.57095	2.2894
1	0	-3.29727	5.18689	0.61887
1	0	-0.77838	3.70842	3.77718
6	0	-5.13751	-1.67044	-2.3612
1	0	-3.91169	-3.41783	-2.65972
1	0	-6.09963	0.20937	-1.91839
1	0	-2.2925	5.46409	2.87614
1	0	-6.03912	-2.13122	-2.76332

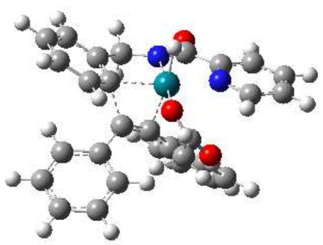

TS(IV-V)Bb'[13]

E(M06 / B1) = -1563.19374287
H(correction)= 0.484199
G(correction)= 0.393527
E(M06 / B2) = -1564.6548415
Imaginary frequencies: 1 (-246.4010 cm^{-1})

45	0	0.14216	-0.60496	-0.99193
7	0	0.9366	-2.08415	0.14321
1	0	0.18144	-1.54257	-2.30976
6	0	-1.67848	-1.41613	-0.20959
8	0	-0.83352	0.82107	-2.24353
6	0	0.02343	-2.76251	1.03608
6	0	2.24809	-2.09107	0.38501
6	0	-1.3572	-2.62611	0.47292
6	0	-2.90745	-1.35484	-0.89848
6	0	-0.55659	2.06657	-2.25876
1	0	0.07965	-2.29657	2.04236
1	0	0.3023	-3.81934	1.17468
6	0	2.96783	-1.07332	-0.4617
8	0	2.86309	-2.78741	1.204
6	0	-2.28371	-3.66033	0.52505
1	0	-3.12998	-0.45651	-1.47475
6	0	-3.81345	-2.40261	-0.85186
8	0	0.38484	2.64319	-1.69389
6	0	-1.56109	2.88928	-3.05916
7	0	2.2198	-0.25261	-1.22658
6	0	4.34757	-0.93902	-0.38631
6	0	-3.51676	-3.5515	-0.11715
1	0	-2.01907	-4.58411	1.04368
1	0	-4.75519	-2.33113	-1.39696
1	0	-1.77475	2.41016	-4.02186
1	0	-2.50768	2.93117	-2.50195
1	0	-1.19264	3.90874	-3.21499
6	0	2.80802	0.72144	-1.92792
6	0	4.96649	0.06042	-1.12308
1	0	4.8808	-1.62145	0.27183
1	0	-4.23013	-4.37445	-0.07075
1	0	2.13188	1.38762	-2.46223
6	0	4.18498	0.90424	-1.90693
1	0	6.04751	0.19052	-1.08048
1	0	4.62832	1.71236	-2.48496
1	0	-3.68753	-0.66141	1.70408
6	0	-3.65857	0.39425	1.43269
6	0	-2.49434	0.91246	0.85603
6	0	-4.75651	1.21486	1.66062
6	0	-1.28105	0.09165	0.68481
6	0	-2.45565	2.26423	0.49492
6	0	-4.71584	2.55771	1.2916
1	0	-5.6524	0.80315	2.12551
6	0	-0.06238	0.39572	0.98775
6	0	-3.56447	3.07759	0.70587
1	0	-1.54409	2.6608	0.04098
1	0	-5.58156	3.19783	1.46149
6	0	0.99429	0.97667	1.72836
1	0	-3.52582	4.126	0.41029
6	0	1.3448	0.48642	3.00331
6	0	1.77953	2.01056	1.16988
6	0	2.43619	1.0047	3.68543
1	0	0.75796	-0.32304	3.43683
6	0	2.86426	2.51735	1.86705
1	0	1.50399	2.38407	0.18287
1	0	2.69429	0.5994	4.6641
6	0	3.20752	2.02049	3.12545
1	0	3.45788	3.31351	1.41608
1	0	4.07082	2.41609	3.65995

[13] Model transition state in which a C-C bond instead of C-H one is formed (affording species **J** instead of **K**, scheme 9)

2-butyne

E(M06 / B1) = -155.846407557
H(correction)= 0.091058
G(correction)= 0.056887
E(M06 / B2) = -155.890398747
Imaginary frequencies: 0

6	0	0.60405	-0.00024	-0.00011
6	0	-0.60405	-0.00019	-0.00004
6	0	2.05825	0.00011	0.00006
6	0	-2.05825	0.00011	0.00001
1	0	2.46125	-0.10526	-1.01586
1	0	2.46018	0.93307	0.41683
1	0	2.46058	-0.82714	0.59949
1	0	-2.4603	0.93359	-0.41545
1	0	-2.46066	-0.82639	-0.60041
1	0	-2.46105	-0.10661	1.01588

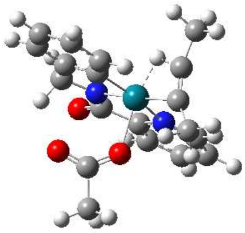

TS(IV-V)Bb(2-butyne)

E(M06 / B1) = -1180.02788568
H(correction)= 0.371784
G(correction)= 0.287877
E(M06 / B2) = -1181.39898363
Imaginary frequencies: 1 (-575.4720 cm^{-1})

45	0	-0.12809	0.55652	-0.12748
7	0	0.2142	-1.30041	-0.95238
1	0	-0.32796	1.46309	-1.51562
6	0	-2.02685	-0.04136	-0.27912
6	0	-0.38023	2.49296	0.56584
8	0	0.31557	-0.20325	1.83255
6	0	-0.94141	-2.15428	-1.10354
6	0	1.43965	-1.76858	-1.12672
6	0	-2.17277	-1.33835	-0.82143
6	0	-3.16873	0.69474	0.03401
6	0	-0.59435	2.64866	-0.68468
6	0	-0.33439	2.9796	1.94792
6	0	-0.1091	-1.27761	2.39776
1	0	-0.88392	-2.9835	-0.37721
1	0	-0.97678	-2.61125	-2.10997
6	0	2.5134	-0.74643	-0.79551
8	0	1.7888	-2.89847	-1.51244
6	0	-3.44955	-1.84571	-1.04362
1	0	-3.06713	1.69576	0.45953
6	0	-4.44411	0.1676	-0.18419
6	0	-1.19384	3.50177	-1.73914
1	0	-0.43851	4.0721	2.03725
1	0	-1.14188	2.49732	2.51786
1	0	0.59872	2.66438	2.43566
8	0	-0.92261	-2.09937	1.987
6	0	0.55127	-1.49427	3.76493
7	0	2.16531	0.48243	-0.3709
6	0	3.852	-1.1134	-0.92605
6	0	-4.5869	-1.10254	-0.72941
1	0	-3.55499	-2.84941	-1.4639
1	0	-5.32656	0.75644	0.0746
1	0	-1.40043	4.50384	-1.3401
1	0	-0.5417	3.60431	-2.61773
1	0	-2.14358	3.06732	-2.08635
1	0	0.50705	-0.5774	4.36648
1	0	0.06548	-2.31781	4.29965
1	0	1.61328	-1.73511	3.62345
6	0	3.11763	1.35803	-0.05807
6	0	4.84188	-0.19724	-0.60592
1	0	4.05405	-2.12414	-1.2738
1	0	-5.57926	-1.51916	-0.90764
1	0	2.76657	2.33345	0.28703
6	0	4.47305	1.06931	-0.15943
1	0	5.89483	-0.46456	-0.69766
1	0	5.21625	1.81796	0.10937

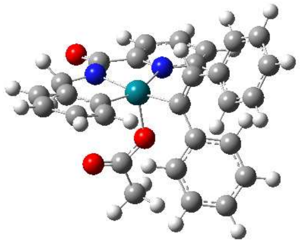

VBb

E(M06 / B1) = -1563.23830539
H(correction)= 0.489574
G(correction)= 0.393609
E(M06 / B2) = -1564.69557364
Imaginary frequencies: 0

45	0	0.76929	-0.04836	-0.35298
7	0	2.75943	-0.17341	-0.93576
6	0	0.82212	-1.99848	-0.72755
8	0	1.05264	0.03982	1.63705
6	0	-1.23146	0.1554	-0.03297
6	0	3.27028	-1.51567	-1.07679
6	0	3.57106	0.87648	-0.87474
6	0	2.0945	-2.45041	-1.15989
6	0	-0.26261	-2.87792	-0.79827
6	0	1.59614	-0.92046	2.32738
6	0	-1.9184	0.51192	-1.14372
6	0	-1.83175	0.0015	1.29769
1	0	3.8942	-1.77701	-0.2016
1	0	3.92532	-1.61268	-1.96105
6	0	2.82342	2.16804	-0.57786
8	0	4.80123	0.91686	-1.02709
6	0	2.2317	-3.74208	-1.66055
1	0	-1.25337	-2.54312	-0.488
6	0	-0.1071	-4.16986	-1.2996
8	0	1.97229	-2.01399	1.94055
6	0	1.67787	-0.52013	3.79508

6	0	-3.36128	0.56741	-1.40182
1	0	-1.31736	0.75602	-2.03526
6	0	-2.55319	1.06082	1.8705
6	0	-1.66222	-1.16221	2.06447
7	0	1.49488	2.12968	-0.36181
6	0	3.53185	3.36444	-0.51489
6	0	1.14202	-4.60741	-1.72645
1	0	3.21401	-4.07622	-2.004
1	0	-0.97097	-4.83374	-1.35894
1	0	0.66062	-0.44904	4.20322
1	0	2.24719	-1.2697	4.35355
1	0	2.14242	0.46645	3.90465
6	0	-3.82911	1.30029	-2.50533
6	0	-4.31842	-0.12234	-0.63606
6	0	-3.10684	0.95449	3.14059
1	0	-2.68655	1.9732	1.28795
6	0	-2.23239	-1.27518	3.32653
1	0	-1.06546	-1.98049	1.66137
6	0	0.84329	3.25615	-0.07946
6	0	2.85163	4.53873	-0.22559
1	0	4.60385	3.31938	-0.69337
1	0	1.26771	-5.61585	-2.12217
6	0	-5.18113	1.37002	-2.81567
1	0	-3.10072	1.82525	-3.12614
6	0	-5.66951	-0.0523	-0.9456
1	0	-3.98851	-0.73002	0.20536
6	0	-2.95765	-0.21872	3.87483
1	0	-3.66337	1.7939	3.55859
1	0	-2.09457	-2.1958	3.89403
1	0	-0.23033	3.15025	0.08836
6	0	1.47899	4.49025	-0.00242
1	0	3.38526	5.48758	-0.17093
6	0	-6.11526	0.69688	-2.03328
1	0	-5.507	1.95242	-3.67816
1	0	-6.38525	-0.60168	-0.33355
1	0	-3.39481	-0.30738	4.86928
1	0	0.90801	5.38637	0.23243
1	0	-7.17689	0.7448	-2.27418

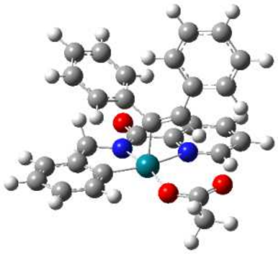

VIBb

E(M06 / B1) = -1563.28556966
H(correction)= 0.490206
G(correction)= 0.393334
E(M06 / B2) = -1564.74241359
Imaginary frequencies: 0

45	0	0.66699	1.02491	0.61539
6	0	2.29146	0.00006	1.1372
7	0	-0.73602	2.32228	-0.6909
6	0	-0.34839	-0.61547	0.1239
8	0	0.02347	1.06187	2.64543
6	0	3.21827	-0.16564	0.08481
6	0	2.5833	-0.5392	2.3917
6	0	-0.22291	2.39978	-1.93302
6	0	-1.92565	2.87632	-0.44186
6	0	-1.62759	-0.66547	0.53956
6	0	0.30733	-1.59637	-0.75622
6	0	-1.0846	1.50586	3.10308
6	0	2.85756	0.37775	-1.26836
6	0	4.40361	-0.86434	0.30681
1	0	1.86284	-0.4028	3.1978
6	0	3.76779	-1.24536	2.59977
6	0	1.1072	1.73824	-2.21414
6	0	-0.88897	3.04793	-2.9664
1	0	-2.2783	2.75493	0.58426
6	0	-2.65069	3.54473	-1.42509
6	0	-2.66867	-1.67216	0.30471
1	0	-1.97922	0.18257	1.13316
6	0	1.12492	-2.61218	-0.24722
6	0	0.09496	-1.53541	-2.13975
8	0	-2.0456	1.95411	2.46305
6	0	-1.17763	1.42264	4.62251
7	0	1.62736	1.13562	-1.14302
1	0	3.65893	1.01259	-1.68455
1	0	2.72308	-0.44494	-1.9978
6	0	4.67836	-1.41116	1.55784
1	0	5.1144	-0.99233	-0.51366
1	0	3.98169	-1.67152	3.58178
8	0	1.5979	1.78989	-3.35063
6	0	-2.12186	3.63201	-2.70717
1	0	-0.41087	3.06369	-3.94306
1	0	-3.61661	3.98206	-1.1798
6	0	-3.96638	-1.34387	0.73917
6	0	-2.49284	-2.93202	-0.29565
6	0	1.69649	-3.55161	-1.09783
1	0	1.31819	-2.64645	0.82461
6	0	0.66803	-2.47403	-2.98931
1	0	-0.53106	-0.73568	-2.5376
1	0	-0.30491	1.90211	5.08261
1	0	-2.09764	1.8941	4.98352
1	0	-1.16416	0.36965	4.93177
1	0	5.6047	-1.96367	1.72036
1	0	-2.66676	4.14673	-3.49864
6	0	-5.03522	-2.21293	0.57366
1	0	-4.11635	-0.37577	1.22049
6	0	-3.5644	-3.80147	-0.45805
1	0	-1.50605	-3.24245	-0.63167
6	0	1.46899	-3.48902	-2.47055
1	0	2.33435	-4.33237	-0.68387
1	0	0.49327	-2.40777	-4.06266
6	0	-4.84314	-3.45248	-0.03107
1	0	-6.02631	-1.92042	0.92189
1	0	-3.39386	-4.77188	-0.92546
1	0	1.92231	-4.22331	-3.13625
1	0	-5.6775	-4.14104	-0.16313

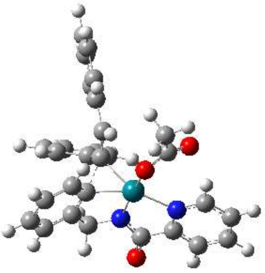

TS(VI-VII)Bb

E(M06 / B1) = -1563.25907066
H(correction)= 0.488855
G(correction)= 0.394756
E(M06 / B2) = -1564.71408677
Imaginary frequencies: 1 (-245.6961 cm^{-1})

45	0	0.98991	0.3844	0.4788
6	0	-0.22747	-0.8005	1.69696
7	0	1.75754	-1.42561	-0.00323
6	0	0.26172	-2.13551	1.70717
6	0	-0.91294	-0.34503	2.83811
6	0	1.16003	-2.59773	0.59514
6	0	2.98228	-1.51698	-0.52613
6	0	-0.01773	-2.96861	2.78836
1	0	-1.25737	0.68753	2.85958
6	0	-1.17046	-1.18248	3.91107
1	0	1.9494	-3.26195	0.98376
1	0	0.60559	-3.19719	-0.15082
6	0	3.53627	-0.16368	-0.87132
8	0	3.6539	-2.54165	-0.7209
6	0	-0.74651	-2.51244	3.88042
1	0	0.37726	-3.98679	2.78257
1	0	-1.71136	-0.80014	4.77734
7	0	2.8201	0.92282	-0.50933
6	0	4.75299	-0.05543	-1.53088
1	0	-0.9619	-3.18015	4.7146
6	0	3.27729	2.14355	-0.82364
6	0	5.23867	1.2064	-1.84151
1	0	5.27206	-0.97986	-1.7747
1	0	2.6117	2.96756	-0.55664
6	0	4.48496	2.32155	-1.48916
1	0	6.1919	1.32232	-2.35738
1	0	4.81965	3.32922	-1.72705
8	0	0.75708	3.67562	-0.32399
6	0	0.31834	3.36936	0.79018
8	0	0.38455	2.23322	1.37601
6	0	-0.44259	4.41785	1.59692
1	0	-0.27771	4.29147	2.67307
1	0	-0.16332	5.42922	1.28154
1	0	-1.51886	4.28973	1.4098
6	0	-0.96975	-0.21354	0.1165
6	0	-1.90207	0.78189	0.19813
6	0	-1.22682	-1.37561	-0.77417
6	0	-3.16382	0.99487	-0.5011
1	0	-1.68615	1.56546	0.92606
6	0	-2.15219	-2.35248	-0.39356
6	0	-0.60478	-1.48415	-2.02018
6	0	-4.02597	1.97019	0.04131
6	0	-3.59943	0.35457	-1.67965
6	0	-2.45948	-3.40869	-1.24358
1	0	-2.63124	-2.26425	0.58328
6	0	-0.91641	-2.5363	-2.87431
1	0	0.13233	-0.7317	-2.29849
6	0	-5.25268	2.27271	-0.52985
1	0	-3.70952	2.49386	0.94467
6	0	-4.8259	0.66261	-2.25261
1	0	-2.96357	-0.38016	-2.1658
6	0	-1.84435	-3.50214	-2.48969
1	0	-3.18296	-4.16217	-0.93248
1	0	-0.42508	-2.60862	-3.84427
6	0	-5.66795	1.61615	-1.68537
1	0	-5.88836	3.03028	-0.07069
1	0	-5.1249	0.14819	-3.16633
1	0	-2.08189	-4.33022	-3.15732
1	0	-6.62884	1.85003	-2.14294

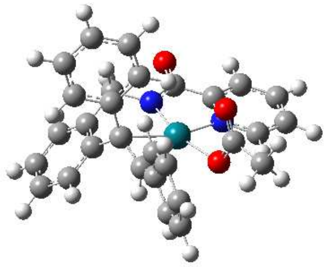

VIIBb

E(M06 / B1) = -1563.31651888
H(correction)= 0.492086
G(correction)= 0.399155
E(M06 / B2) = -1564.76788558
Imaginary frequencies: 0

45	0	0.62185	0.41239	0.33699
7	0	1.90762	-1.15896	-0.22005
8	0	-0.11721	2.35433	0.7194
6	0	1.55839	-2.54955	-0.45583
6	0	3.17178	-0.84562	-0.53678
6	0	-0.53964	3.00959	-0.31244
6	0	0.68806	-3.08168	0.63669
1	0	2.47988	-3.14341	-0.53283
1	0	1.04274	-2.64259	-1.43011
6	0	3.48835	0.59089	-0.27318
8	0	4.061	-1.58799	-0.98634
8	0	-0.55234	2.62998	-1.48185
6	0	-1.08609	4.38204	0.05571
6	0	-0.46849	-2.3741	1.00188
6	0	1.0302	-4.24241	1.32399
7	0	2.4824	1.37378	0.15606
6	0	4.77585	1.07792	-0.45543
1	0	-0.41627	4.89827	0.75587
1	0	-1.23767	4.99149	-0.84132
1	0	-2.04985	4.24891	0.56773
6	0	-1.25615	-2.87014	2.0456
6	0	-0.8927	-1.15258	0.24667
6	0	0.23849	-4.72875	2.36123
1	0	1.94533	-4.76613	1.0428
6	0	2.73619	2.65816	0.44175
6	0	5.04073	2.40831	-0.16885
1	0	5.52304	0.37561	-0.81777
1	0	-2.1667	-2.33297	2.31502

6	0	-0.91063	-4.03616	2.72338
6	0	-1.36562	-0.07612	1.03797
6	0	-1.34624	-1.43135	-1.15597
1	0	0.52374	-5.63975	2.88756
1	0	1.87916	3.22782	0.79687
6	0	4.00081	3.20994	0.29469
1	0	6.04135	2.81906	-0.30065
1	0	-1.54485	-4.40291	3.53081
6	0	-2.47958	0.87001	0.8401
1	0	-1.1497	-0.20305	2.1046
6	0	-2.0837	-2.59236	-1.41901
6	0	-1.04521	-0.57942	-2.22868
1	0	4.15908	4.25871	0.5382
6	0	-2.85953	1.63229	1.95696
6	0	-3.21842	1.04861	-0.338
6	0	-2.51667	-2.89503	-2.70618
1	0	-2.31616	-3.27003	-0.5969
6	0	-1.47852	-0.88529	-3.51427
1	0	-0.49593	0.34527	-2.03066
6	0	-3.92862	2.51633	1.91459
1	0	-2.2794	1.52799	2.8747
6	0	-4.28965	1.93424	-0.38314
1	0	-2.94793	0.50353	-1.23735
6	0	-2.21239	-2.043	-3.76235
1	0	-3.09094	-3.80493	-2.88276
1	0	-1.23489	-0.20673	-4.33125
6	0	-4.65734	2.67179	0.73788
1	0	-4.19104	3.09008	2.80394
1	0	-4.83954	2.05078	-1.31695
1	0	-2.54283	-2.28006	-4.77386
1	0	-5.49802	3.36414	0.694

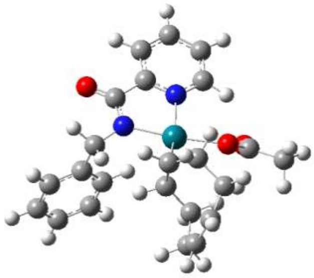

B(COD)

E(M06 / B1) = -1336.02189142
H(correction)= 0.476627
G(correction)= 0.388084
E(M06 / B2) = -1337.42688825
Imaginary frequencies: 0

45	0	-0.36336	0.54048	-0.31283
7	0	1.53138	0.01722	-0.98018
8	0	-2.30265	1.24063	0.2551
6	0	-1.38572	-0.95576	-1.44617
6	0	2.02641	-1.29107	-1.33169
6	0	2.46152	0.98262	-1.0339
6	0	-2.58757	1.78071	1.38027
6	0	-1.05552	-1.46682	-0.17458
1	0	-0.67435	-1.20555	-2.24137
6	0	-2.73834	-0.56859	-2.00329
1	0	2.79329	-1.19912	-2.11747
1	0	1.20219	-1.88937	-1.75418
6	0	2.62729	-2.06262	-0.17487
6	0	1.96667	2.30819	-0.52953
8	0	3.64058	0.88362	-1.41054
8	0	-1.80791	2.1549	2.26293
6	0	-4.09177	1.92723	1.61105
6	0	-1.95922	-1.74065	0.99797
1	0	-0.16035	-2.10038	-0.14208
1	0	-2.81524	-1.00945	-3.01362
1	0	-2.77973	0.52191	-2.14029
6	0	-3.97558	-0.99767	-1.20772
6	0	3.45843	-3.15551	-0.4303
6	0	2.34531	-1.7362	1.15266
6	0	2.79931	3.42033	-0.56162
7	0	0.73123	2.35846	0.00062
1	0	-4.48252	0.98359	2.01975
1	0	-4.29567	2.72004	2.33951
1	0	-4.62324	2.12196	0.6717
6	0	-2.64021	-3.10754	0.9105
1	0	-2.69185	-0.9414	1.13596
1	0	-1.33416	-1.72629	1.90581
1	0	-3.9472	-0.50411	-0.23195
1	0	-4.86143	-0.59423	-1.72357
6	0	-4.13035	-2.4886	-1.10197
1	0	3.69269	-3.40931	-1.4665
6	0	3.99373	-3.90934	0.60799
6	0	2.87994	-2.48871	2.19466
1	0	1.69559	-0.88202	1.35255
6	0	2.34029	4.61592	-0.0292
1	0	3.78694	3.29379	-0.99905
6	0	0.29777	3.50138	0.54652
6	0	-3.57548	-3.34583	-0.23851
1	0	-1.85535	-3.88369	0.86322
1	0	-3.17757	-3.31157	1.85481
1	0	-4.76492	-2.93692	-1.87424
1	0	4.64501	-4.7553	0.38547
6	0	3.70447	-3.57799	1.92937
1	0	2.64787	-2.21924	3.22519
6	0	1.07311	4.65514	0.54438
1	0	2.96814	5.50698	-0.04495
1	0	-0.67653	3.44346	1.03064
1	0	-3.83359	-4.40153	-0.37736
1	0	4.12408	-4.16368	2.74748
1	0	0.6838	5.56505	0.99676

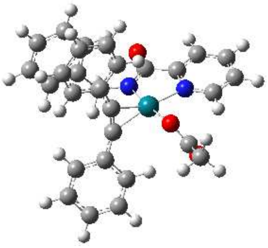

B(diphenylacetylene)

E(M06 / B1) = -1563.27922542
H(correction)= 0.490728
G(correction)= 0.391450
E(M06 / B2) = -1564.73782685
Imaginary frequencies: 0

45	0	-0.92422	0.01602	0.23647
7	0	-0.40711	-0.97815	-1.49793
6	0	0.52245	1.49043	0.04888
6	0	1.02138	0.55341	0.74236
6	0	0.73387	-0.67811	-2.3314
6	0	-1.17567	-2.00548	-1.87926
6	0	0.42954	2.73057	-0.66002
6	0	1.97814	-0.10077	1.58551
1	0	0.76937	0.41077	-2.49949
1	0	0.58616	-1.16458	-3.31008
6	0	2.06282	-1.11701	-1.7704
6	0	-2.43628	-2.11712	-1.06415
8	0	-0.99376	-2.80255	-2.81485
6	0	-0.81867	3.34331	-0.86678
6	0	1.5832	3.34746	-1.1747
6	0	1.61915	-1.23846	2.32412
6	0	3.29462	0.37774	1.68751
6	0	2.1849	-2.29708	-1.03721
6	0	3.20846	-0.35275	-2.00142
7	0	-2.64227	-1.20818	-0.09415
6	0	-3.37896	-3.09389	-1.36249
6	0	-0.89654	4.54232	-1.56251
1	0	-1.7106	2.86301	-0.45782
6	0	1.4924	4.54282	-1.87489
1	0	2.54889	2.86435	-1.02042
6	0	2.54665	-1.87402	3.13703
1	0	0.59443	-1.59938	2.23422
6	0	4.21953	-0.26354	2.49979
1	0	3.58045	1.2538	1.10522
1	0	1.28948	-2.88583	-0.83843
6	0	3.41935	-2.69424	-0.53458
6	0	4.44569	-0.7483	-1.50388
1	0	3.11606	0.57635	-2.56852
6	0	-3.80057	-1.20362	0.57259
6	0	-4.56934	-3.11377	-0.65034
1	0	-3.13981	-3.79497	-2.15852
6	0	0.25206	5.14632	-2.07225
1	0	-1.86935	5.01088	-1.71045
1	0	2.39581	5.00575	-2.27284
6	0	3.85176	-1.39318	3.22767
1	0	2.25037	-2.75574	3.70547
1	0	5.23908	0.11797	2.56083
1	0	3.49248	-3.60482	0.0603
6	0	4.55431	-1.92145	-0.76233
1	0	5.32633	-0.1304	-1.68307
1	0	-3.92255	-0.39449	1.29066
6	0	-4.79195	-2.14547	0.32456
1	0	-5.32675	-3.86926	-0.85923
1	0	0.18112	6.08442	-2.62267
1	0	4.57964	-1.89582	3.86455
1	0	5.5162	-2.22451	-0.34877
1	0	-5.72213	-2.11142	0.88792
8	0	-1.49149	0.87469	2.08926
6	0	-2.52081	1.63221	2.12569
8	0	-3.32226	1.85095	1.20654
6	0	-2.71963	2.31422	3.47646
1	0	-1.9629	3.10029	3.59808
1	0	-3.71482	2.76736	3.54229
1	0	-2.57261	1.59987	4.29546

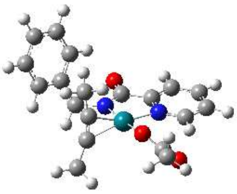

B(2-butyne)

E(M06 / B1) = -1180.09074375
H(correction)= 0.378399
G(correction)= 0.293398
E(M06 / B2) = -1181.45457601
Imaginary frequencies: 0

45	0	-0.62804	-0.48689	0.19994
7	0	0.70008	0.949	0.85372
6	0	0.42571	-2.08807	1.08963
6	0	0.82556	-1.99492	-0.09166
8	0	-1.90804	-1.9644	-0.6542
6	0	2.00808	0.70234	1.42825
6	0	0.39233	2.23193	0.63752
6	0	0.24959	-2.53617	2.47267
6	0	1.44168	-2.24799	-1.39351
6	0	-3.16106	-2.12909	-0.4851
1	0	1.93348	-0.13791	2.13403
1	0	2.31269	1.59914	1.9925
6	0	3.07569	0.3897	0.40978
6	0	-1.00953	2.41058	0.13161
8	0	1.10482	3.23824	0.82492
1	0	-0.76177	-2.93901	2.6231
1	0	0.97384	-3.31904	2.74943
1	0	0.36456	-1.69864	3.17699
1	0	2.14531	-3.09507	-1.36087
1	0	0.65676	-2.47164	-2.12992
1	0	1.99415	-1.36425	-1.74411
8	0	-3.96802	-1.38645	0.09066
6	0	-3.67149	-3.43498	-1.10031
6	0	3.35341	1.29745	-0.61758
6	0	3.80769	-0.79573	0.46893
7	0	-1.75492	1.30404	-0.05637
6	0	-1.51348	3.68485	-0.09609
1	0	-3.18636	-4.28915	-0.61019
1	0	-4.75826	-3.52092	-0.9915
1	0	-3.40007	-3.48343	-2.1627
1	0	2.78962	2.23012	-0.64879
6	0	4.33784	1.01913	-1.55931
6	0	4.79847	-1.07448	-0.46924
1	0	3.57211	-1.52151	1.24934
6	0	-3.03131	1.42993	-0.44187
6	0	-2.8293	3.81996	-0.51555
1	0	-0.84408	4.52473	0.07449
1	0	4.54142	1.73653	-2.35483
6	0	5.06546	-0.16801	-1.49013
1	0	5.35357	-2.01133	-0.41025
1	0	-3.5847	0.48985	-0.50672
6	0	-3.60265	2.67572	-0.68223
1	0	-3.25305	4.80725	-0.70139
1	0	5.83441	-0.38518	-2.23184
1	0	-4.64431	2.73597	-0.99157

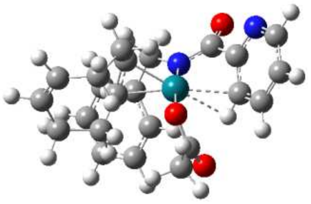

IBa(COD)

E(M06 / B1) = -1335.99827804
H(correction)= 0.475936
G(correction)= 0.385847
E(M06 / B2) = -1337.40164424
Imaginary frequencies: 0

45	0	0.5936	-0.11759	-0.05644
6	0	-0.93072	1.28756	-0.44695
6	0	-0.05114	1.21598	-1.55995
7	0	-0.52429	-1.71974	-0.735
8	0	1.84567	1.42815	0.69321
6	0	-1.06747	2.38485	0.57522
1	0	-1.86595	0.72513	-0.55732
1	0	-0.3969	0.56087	-2.36764
6	0	0.96356	2.231	-2.04215
6	0	-1.87708	-1.78316	-1.21194
6	0	0.27992	-2.76526	-1.01139
6	0	2.00978	1.45805	1.96524
6	0	-1.99243	3.51114	0.11103
1	0	-0.09176	2.77313	0.87718
1	0	-1.50169	1.93736	1.4841
1	0	0.85243	2.31095	-3.13793
1	0	1.97902	1.84672	-1.86702
6	0	0.8784	3.64554	-1.45635
1	0	-2.02272	-2.77	-1.68329
1	0	-2.04285	-1.03647	-2.01328
6	0	-2.94296	-1.57209	-0.1624
6	0	1.67324	-2.46147	-0.50625
8	0	0.00719	-3.82463	-1.57649
8	0	1.65055	0.60561	2.78491
6	0	2.69251	2.7317	2.45306
6	0	-1.59359	4.27175	-1.11969
1	0	-2.99387	3.08291	-0.07279
1	0	-2.14294	4.22703	0.93971
1	0	1.09697	3.60039	-0.38463
1	0	1.69437	4.23667	-1.90131
6	0	-0.42094	4.3367	-1.75905
6	0	-2.65991	-1.54919	1.20275
6	0	-4.26931	-1.39999	-0.56888
7	0	2.70755	-2.74667	-1.31834
6	0	1.84952	-1.90225	0.77809
1	0	1.93419	3.52286	2.54874
1	0	3.14753	2.57736	3.43776
1	0	3.44217	3.08349	1.7346
1	0	-2.40794	4.86515	-1.54925
1	0	-0.40666	4.95522	-2.66293
1	0	-1.6235	-1.65927	1.52009
6	0	-3.67684	-1.36189	2.13531
6	0	-5.28833	-1.21924	0.3584
1	0	-4.49893	-1.40765	-1.63697
6	0	3.92211	-2.43637	-0.88793
6	0	3.14461	-1.57199	1.19509
1	0	1.01795	-1.83236	1.49074
1	0	-3.43263	-1.33911	3.19729
6	0	-4.99476	-1.19874	1.71998
1	0	-6.31567	-1.08733	0.01757
1	0	4.74431	-2.67587	-1.5692
6	0	4.20178	-1.83295	0.34294
1	0	3.27474	-1.09641	2.16539
1	0	-5.78837	-1.05073	2.4523
1	0	5.22685	-1.58495	0.61329

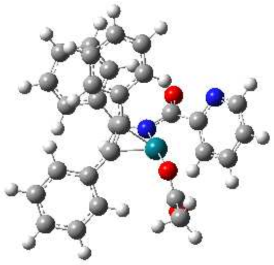

IBa(diphenylacetylene)

E(M06 / B1) = -1563.25587086
H(correction)= 0.489917
G(correction)= 0.391113
E(M06 / B2) = -1564.71203821
Imaginary frequencies: 0

45	0	-1.13831	-0.0214	-0.13726
7	0	-0.15313	-0.69391	-1.81045
6	0	0.26125	1.40182	0.38109
6	0	0.61837	0.28428	0.88181
8	0	-2.26794	0.65567	1.52649
6	0	0.97636	-0.12041	-2.5082
6	0	-0.52982	-1.94911	-2.09369
6	0	0.34248	2.79748	0.0764
6	0	1.46626	-0.59616	1.63142
6	0	-3.27366	1.3455	1.14297
1	0	0.79993	0.95927	-2.63332
1	0	1.01547	-0.57651	-3.51189
6	0	2.30618	-0.32732	-1.82448
6	0	-1.70611	-2.29385	-1.20292
8	0	-0.05073	-2.74472	-2.90595
6	0	-0.76768	3.48033	-0.45059
6	0	1.53634	3.51019	0.2919
6	0	1.21701	-1.97592	1.68351
6	0	2.58181	-0.08537	2.31606
8	0	-3.63706	1.51974	-0.03255
6	0	-4.03439	2.02613	2.27062
6	0	2.71126	-1.61018	-1.4449
6	0	3.15378	0.74814	-1.55819
7	0	-1.66318	-3.45791	-0.52585
6	0	-2.78608	-1.39019	-1.08912
6	0	-0.67989	4.83518	-0.74086
1	0	-1.6956	2.92617	-0.60855
6	0	1.61464	4.86311	-0.00739
1	0	2.4004	2.97638	0.68941
6	0	2.05554	-2.81384	2.40602
1	0	0.36216	-2.37343	1.13466
6	0	3.41291	-0.9273	3.04057

1	0	2.78131	0.98531	2.26881
1	0	-3.53635	2.9772	2.50335
1	0	-5.0634	2.24595	1.96536
1	0	-4.02483	1.41708	3.18139
1	0	2.05394	-2.45275	-1.66276
6	0	3.92439	-1.80252	-0.79454
6	0	4.37061	0.55745	-0.90865
1	0	2.84098	1.7554	-1.84202
6	0	-2.66014	-3.7132	0.30963
6	0	-3.81196	-1.68374	-0.1821
1	0	-2.87584	-0.52849	-1.75795
6	0	0.50703	5.53336	-0.52422
1	0	-1.55039	5.35269	-1.14347
1	0	2.54819	5.39998	0.16268
6	0	3.15357	-2.29591	3.08971
1	0	1.84992	-3.8835	2.43267
1	0	4.2732	-0.5142	3.56751
1	0	4.21486	-2.80621	-0.48385
6	0	4.75761	-0.72098	-0.51868
1	0	5.00971	1.41476	-0.69276
1	0	-2.5966	-4.66182	0.85081
6	0	-3.74556	-2.86088	0.53993
1	0	-4.62227	-0.96813	-0.05297
1	0	0.56997	6.59592	-0.7581
1	0	3.80653	-2.95635	3.66015
1	0	5.69859	-0.87456	0.00982
1	0	-4.50847	-3.12656	1.26917

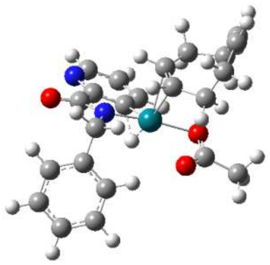

TS(I-II)Ba(COD)[14]

E(M06 / B1) = -1335.96826246
H(correction)= 0.470693
G(correction)= 0.383297
E(M06 / B2) = -1337.3740232
Imaginary frequencies: 1 (-598.3530 cm^{-1})

45	0	-0.18941	-0.0208	0.33828
7	0	1.24421	0.38425	-1.04841
6	0	0.26414	1.90122	0.7957
8	0	-1.57053	-0.60012	1.90564
6	0	-1.82394	0.61861	-1.12179
6	0	1.92967	-0.69881	-1.72133
6	0	1.79851	1.61899	-1.11201
6	0	1.11253	2.53284	-0.13922
6	0	-0.38215	2.71321	1.7311
1	0	0.83858	0.61283	1.45265
6	0	-1.24184	-1.83138	1.9839
6	0	-1.60183	-0.72991	-1.34826
1	0	-1.21769	1.29528	-1.72943
6	0	-2.96511	1.31026	-0.42617
1	0	2.27998	-0.35261	-2.70652
1	0	1.20558	-1.51397	-1.88015
6	0	3.09782	-1.22208	-0.92145
8	0	2.74042	1.95164	-1.83529
7	0	1.32478	3.8483	-0.18454
6	0	-0.16803	4.08749	1.68433
1	0	-1.03832	2.26438	2.47901
8	0	-0.36754	-2.35512	1.26388
6	0	-2.02242	-2.67076	2.97205
6	0	-2.45964	-1.91317	-0.98519
1	0	-0.87795	-0.94202	-2.14109
1	0	-3.35355	2.07461	-1.1226
1	0	-2.56856	1.87637	0.43245
6	0	-4.1414	0.44829	0.04013
6	0	2.88162	-2.08562	0.15662
6	0	4.40045	-0.81659	-1.21504
6	0	0.69105	4.60088	0.71502
1	0	-0.65711	4.75603	2.39363
1	0	-3.03596	-2.83457	2.57886
1	0	-1.54228	-3.64252	3.1243
1	0	-2.12691	-2.14466	3.92793
6	0	-3.62436	-2.12837	-1.94946
1	0	-2.83828	-1.84624	0.0391
1	0	-1.82211	-2.80806	-0.99617
1	0	-3.77044	-0.32802	0.71893
1	0	-4.79514	1.08624	0.65382
6	0	-4.95793	-0.08687	-1.10632
1	0	1.86052	-2.38008	0.41094
6	0	3.95385	-2.54427	0.91481
6	0	5.47327	-1.27931	-0.45835
1	0	4.55567	-0.10734	-2.02753
1	0	0.87656	5.67753	0.66183
6	0	-4.73137	-1.118	-1.92723
1	0	-3.22977	-2.17966	-2.98026
1	0	-4.06519	-3.12591	-1.76928
1	0	-5.85163	0.50384	-1.33286
1	0	3.77175	-3.217	1.75313
6	0	5.25358	-2.14859	0.60625
1	0	6.48615	-0.95403	-0.69773
1	0	-5.47391	-1.27094	-2.71792
1	0	6.09303	-2.51102	1.20052

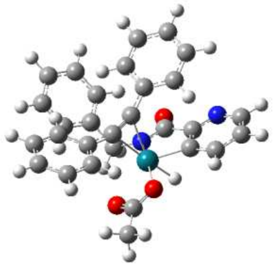

---

[14] Differences in energy depending on the orientation of benzyl ring are usually bellow 1 kcal·$mol^{-1}$and both conformers can be normally found. However, in the case of **TS(I-II)Ba(cod**) that with benzyl ring parallel to the Rh-H bond that is being formed could only be optimized, whereas that with benzyl group parallel to the ligand could only be optimized in the case of **TS(I-II)Ba(diphenylacetylene**).

TS(I-II)Ba(diphenylacetylene)

E(M06 / B1) = -1563.21492502
H(correction)= 0.486091
G(correction)= 0.390363
E(M06 / B2) = -1564.67391194
Imaginary frequencies: 1 (-543.9700 cm^{-1})

45	0	-0.17346	-1.28156	-0.56067
7	0	0.98738	0.1047	-1.4967
6	0	1.68307	-2.06876	-0.31321
8	0	-1.5138	-2.90427	0.08143
6	0	0.36699	1.05987	-2.38652
6	0	2.33429	-0.01478	-1.49767
6	0	2.73875	-1.22166	-0.70682
6	0	2.00957	-3.21761	0.41098
1	0	0.67014	-2.34395	-1.46411
6	0	-2.40676	-2.68409	-0.80311
1	0	-0.60044	0.64026	-2.7066
1	0	1.00619	1.16458	-3.27951
6	0	0.14759	2.42492	-1.7835
8	0	3.12484	0.72796	-2.08955
7	0	4.02355	-1.44616	-0.4253
6	0	3.34491	-3.4466	0.7258
1	0	1.22314	-3.90595	0.72381
8	0	-2.34335	-1.73404	-1.61132
6	0	-3.609	-3.60184	-0.83652
6	0	1.24167	3.23844	-1.46696
6	0	-1.13835	2.90575	-1.54226
6	0	4.307	-2.53707	0.28392
1	0	3.64308	-4.32312	1.30211
1	0	-4.50693	-3.0169	-0.59697
1	0	-3.74383	-4.00326	-1.84803
1	0	-3.507	-4.42421	-0.12075
1	0	2.2422	2.84014	-1.6429
6	0	1.04536	4.50534	-0.92901
6	0	-1.33702	4.17345	-0.99688
1	0	-1.99451	2.27024	-1.77929
1	0	5.36341	-2.705	0.51309
1	0	1.90771	5.12762	-0.68688
6	0	-0.24453	4.97883	-0.68957
1	0	-2.35141	4.53157	-0.81401
1	0	-0.39619	5.97139	-0.26489
1	0	-2.60967	2.37871	0.71513
6	0	-3.27872	1.54766	0.93486
6	0	-2.73166	0.26025	1.03944
6	0	-4.63983	1.75434	1.11797
6	0	-1.3166	0.07702	0.88314
6	0	-3.58137	-0.81083	1.35315
1	0	-5.04766	2.76145	1.03085
6	0	-5.47914	0.68397	1.41582
6	0	-0.10805	0.18292	1.16838
6	0	-4.94088	-0.59511	1.53724
1	0	-3.14755	-1.80524	1.44732
1	0	-6.5472	0.84646	1.55826
6	0	1.10916	0.70253	1.71593
1	0	-5.5874	-1.43852	1.78115
6	0	2.08767	-0.12714	2.27887
6	0	1.32895	2.08748	1.67511
6	0	3.25636	0.41932	2.7915
1	0	1.92893	-1.20446	2.28288
6	0	2.50383	2.62523	2.18071
1	0	0.57501	2.72845	1.21844
6	0	3.47072	1.79425	2.74176
1	0	4.01688	-0.24088	3.20725
1	0	2.66797	3.70111	2.12159
1	0	4.39869	2.21633	3.12654

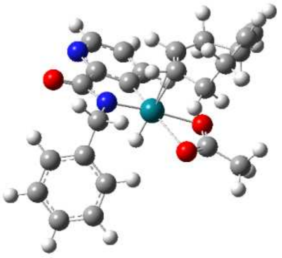

IIBa(COD)

E(M06 / B1) = -1335.98216277
H(correction)= 0.472953
G(correction)= 0.383768
E(M06 / B2) = -1337.38741665
Imaginary frequencies: 0

45	0	0.06106	0.02932	-0.49488
7	0	-1.28556	0.40559	0.97181
6	0	-0.17071	1.98804	-0.67013
1	0	-1.10265	0.01207	-1.52564
6	0	1.71953	-0.46164	1.46272
6	0	-1.92378	-0.69986	1.64793
6	0	-1.76129	1.6581	1.16775
6	0	-1.05096	2.60143	0.24011
6	0	0.45851	2.80647	-1.60462
6	0	2.08875	0.7567	1.00463
6	0	2.36869	-1.80534	1.27316
1	0	0.89927	-0.45102	2.18427
1	0	-2.24904	-0.36299	2.64538
1	0	-1.17586	-1.50082	1.77907
6	0	-3.10528	-1.24636	0.88732
8	0	-2.64041	1.98318	1.97034
7	0	-1.29241	3.91296	0.29079
6	0	0.21449	4.17913	-1.56427
1	0	1.12258	2.37268	-2.35621
1	0	1.51011	1.59783	1.39348
6	0	3.22982	1.17495	0.12873
6	0	3.57875	-2.0154	2.18066
1	0	2.64841	-1.9842	0.23105
1	0	1.61871	-2.57742	1.49304
6	0	-2.95062	-2.30884	-0.00504
6	0	-4.36546	-0.66178	1.03752
6	0	-0.6565	4.67698	-0.59851
1	0	0.68761	4.85672	-2.27646
1	0	3.76158	1.99152	0.64859
1	0	2.81326	1.63475	-0.78167
6	0	4.2449	0.10284	-0.26847
6	0	4.8047	-1.20514	1.88925
1	0	3.28269	-1.82119	3.22692
1	0	3.86005	-3.08358	2.16287
6	0	-4.04323	-2.79418	-0.71828
1	0	-1.95669	-2.73589	-0.15509
6	0	-5.45639	-1.14734	0.32448

1	0	-4.45944	0.19618	1.704
1	0	-0.85928	5.75094	-0.546
1	0	3.71948	-0.70726	-0.78588
1	0	4.91454	0.54145	-1.02269
6	0	5.08747	-0.36501	0.88808
1	0	5.59655	-1.34279	2.63333
1	0	-3.91011	-3.62329	-1.41374
6	0	-5.30013	-2.21983	-0.55096
1	0	-6.43578	-0.68465	0.45074
1	0	6.07518	0.1042	0.939
1	0	-6.1563	-2.6015	-1.10817
8	0	1.45489	-0.63849	-2.06536
6	0	1.2005	-1.86877	-1.87514
8	0	0.41629	-2.26304	-0.97711
6	0	1.93088	-2.87848	-2.72965
1	0	2.92462	-3.06159	-2.29602
1	0	1.38832	-3.82887	-2.75499
1	0	2.07802	-2.49349	-3.74445

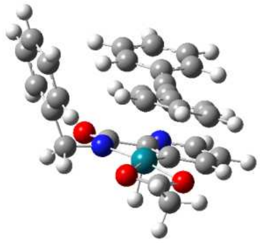

IIBa(diphenylacetylene)

E(M06 / B1) = -1563.23371794
H(correction)= 0.488006
G(correction)= 0.393395
E(M06 / B2) = -1564.69367517
Imaginary frequencies: 0

45	0	-0.38889	-0.88502	-1.08013
7	0	1.15174	0.35887	-1.5279
6	0	1.16282	-2.00381	-0.59045
1	0	-0.16455	-1.45781	-2.48936
8	0	-2.20564	-2.08706	-0.85365
6	0	0.91446	1.58393	-2.25917
6	0	2.43195	-0.04716	-1.35369
6	0	2.43582	-1.43025	-0.76817
6	0	1.10512	-3.29264	-0.06839
6	0	-2.94655	-1.1932	-1.37078
1	0	-0.03262	1.46907	-2.80915
1	0	1.72724	1.73708	-2.98737
6	0	0.82288	2.77842	-1.34633
8	0	3.44133	0.60573	-1.6362
7	0	3.58576	-2.02941	-0.45697
6	0	2.2997	-3.93023	0.26785
1	0	0.13937	-3.78319	0.07303
8	0	-2.49725	-0.07194	-1.72119
6	0	-4.40777	-1.5049	-1.58504
6	0	1.97283	3.48714	-0.98814
6	0	-0.40203	3.14934	-0.7877
6	0	3.50141	-3.25888	0.05528
1	0	2.30299	-4.93927	0.68342
1	0	-4.71016	-2.38363	-1.00565
1	0	-5.01908	-0.63937	-1.30306
1	0	-4.58342	-1.70342	-2.65065
6	0	1.898	4.54579	-0.08822
1	0	2.92721	3.1552	-1.39849
6	0	-0.47745	4.20569	0.11638
1	0	-1.29694	2.58917	-1.07051
1	0	4.45002	-3.74297	0.30849
1	0	2.80243	5.0894	0.18707
6	0	0.67262	4.90817	0.46823
1	0	-1.4396	4.48762	0.5464
1	0	0.61445	5.73479	1.17633
1	0	1.3345	-1.93712	2.91433
6	0	1.92054	-1.07079	2.6114
6	0	1.28077	-0.0074	1.95929
6	0	3.29009	-1.02701	2.82611
6	0	-0.11533	-0.04348	1.68659
6	0	2.03085	1.10165	1.54668
1	0	3.78656	-1.86816	3.30882
6	0	4.03512	0.0645	2.38429
6	0	-1.32077	-0.02114	1.50396
6	0	3.40252	1.12844	1.74903
1	0	1.52666	1.91696	1.03317
1	0	5.11557	0.07542	2.52022
6	0	-2.74087	0.09203	1.48608
1	0	3.97773	1.97297	1.37244
6	0	-3.34732	1.30051	1.11459
6	0	-3.5502	-0.98781	1.86605
6	0	-4.72822	1.43212	1.15038
1	0	-2.71195	2.12505	0.79402
6	0	-4.9314	-0.8481	1.90254
1	0	-3.07566	-1.93468	2.1158
6	0	-5.52528	0.36152	1.55048
1	0	-5.18702	2.37576	0.85773
1	0	-5.55	-1.69471	2.19874
1	0	-6.60937	0.46713	1.57773
